# N_2_Phos – an easily made, highly effective ligand designed for ppm level Pd-catalyzed Suzuki–Miyaura cross couplings in water†

**DOI:** 10.1039/d0sc00968g

**Published:** 2020-05-07

**Authors:** Nnamdi Akporji, Ruchita R. Thakore, Margery Cortes-Clerget, Joel Andersen, Evan Landstrom, Donald H. Aue, Fabrice Gallou, Bruce H. Lipshutz

**Affiliations:** Department of Chemistry & Biochemistry, University of California Santa Barbara Santa Barbara CA 93106 USA lipshutz@chem.ucsb.edu; Novartis Pharma AG Basel Switzerland; Department of Chemistry, University of Cincinnati, Cincinnati OH 45221 USA

## Abstract

A new biaryl phosphine-containing ligand from an active palladium catalyst for ppm level Suzuki–Miyaura couplings, enabled by an aqueous micellar reaction medium. A wide array of functionalized substrates including aryl/heteroaryl bromides are amenable, as are, notably, chlorides. The catalytic system is both general and highly effective at low palladium loadings (1000–2500 ppm or 0.10–0.25 mol%). Density functional theory calculations suggest that greater steric congestion in N_2_Phos induces increased steric crowding around the Pd center, helping to destabilize the 2 : 1 ligand–Pd(0) complex more for N_2_Phos than for EvanPhos (and less bulky ligands), and thereby favoring formation of the 1 : 1 ligand–Pd^o^ complex that is more reactive in oxidative addition to aryl chlorides.

## Abstract 2

A new, biaryl phosphine-containing ligand, N_2_Phos, forms a 1 : 1 complex with Pd resulting in an active catalyst at the ppm level for Suzuki–Miyaura couplings in water, enabled by an aqueous micellar medium. Notably, aryl chlorides are shown to be amenable substrates.

## Graphical abstract

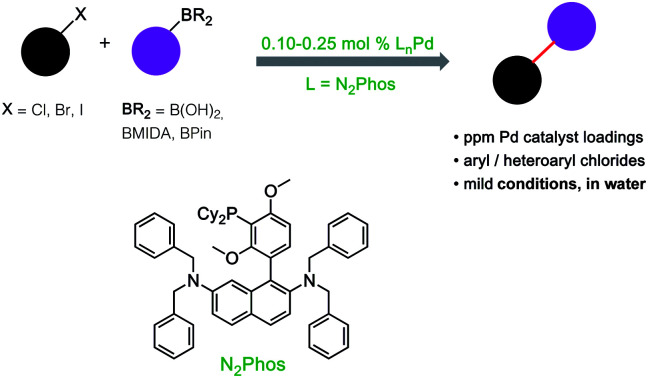

## Introduction

**Scheme 1:**
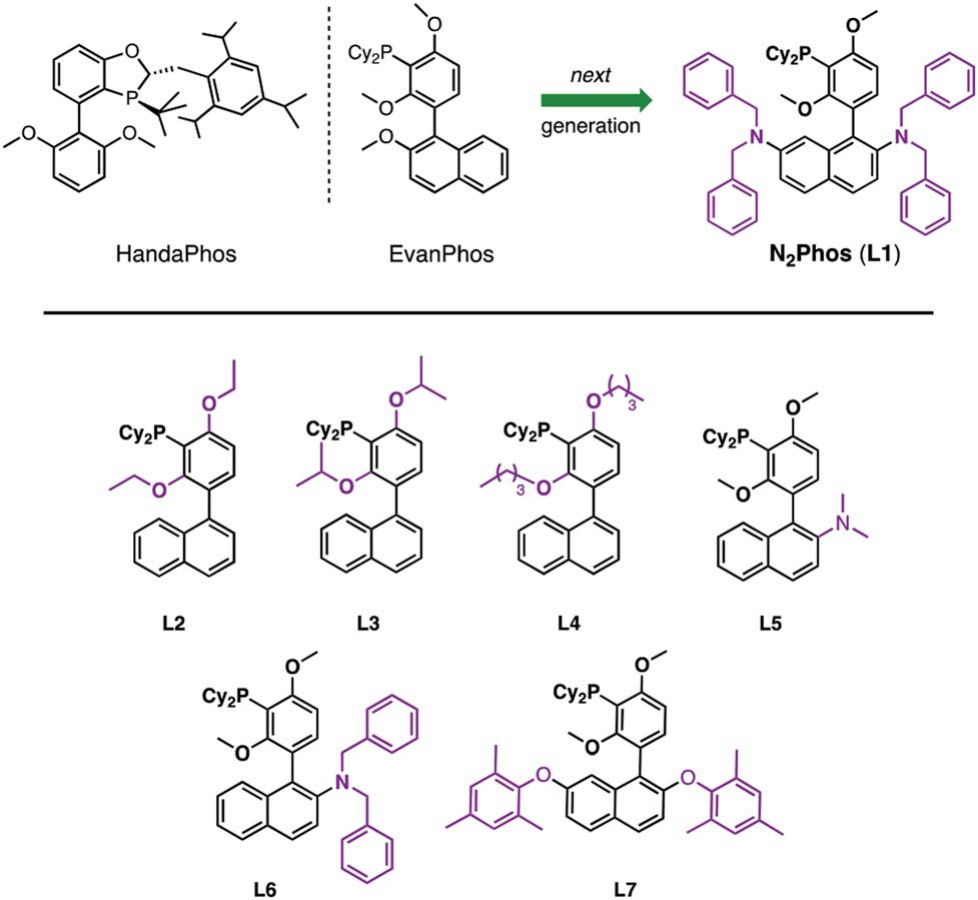
HandaPhos, EvanPhos, and next generation ligand, N_2_Phos (**L1**), for SMCs (top). Ligands prepared and screened based on the EvanPhos biaryl skeleton (bottom).

Since the seminal paper on Suzuki Miyaura cross-coupling (SMC) appeared in 1979,^1^ this C–C bond-forming chemistry has been integral to the field of organometallics, with seemingly an almost unlimited number of applications. As a Nobel Prize-winning reaction, it has been studied extensively and is used widely in both academic and industrial settings.^2–5^ However, and notwithstanding its award-winning status, the world's limited access to palladium^6^ places it not only as an endangered metal, but also of considerable cost, being far more expensive today than gold. Hence, it has become essential to focus on developing new catalysts that rely on far less palladium per reaction, while maintaining mild conditions, functional group tolerance, and high reactivity and efficiency. This is especially relevant to the pharmaceutical industry, where pressure to reduce the price of drugs seems never-ending. Moreover, every active pharmaceutical ingredient (API) must adhere to strict FDA guidelines that require very low levels of residual Pd (≤10 ppm per dose),^7^ which only adds cost in the form of clean-up (*e.g.*, metal scavenging). One alternative to precious metal catalysis envisions replacement of Pd by more readily available and inexpensive base metals, such as Ni, Co, Fe, and Cu, among others.^8–11^ And while processes have been uncovered in this regard, most accomplish their goals at great expense to the environment, typically relying on high loadings leading to an even greater need for removal of residual metal in the product, along with a considerable investment in energy (*i.e.*, heating reaction mixtures). Typical conditions for Pd-catalyzed SMCs involve moist organic media and tend to rely on unsustainably high Pd catalyst loadings in the 1–5 mol% range. Although there are isolated cases describing ppm levels of catalysis, or lower,^12,13*a*^ and an associated recent review,^13*b*^ most ligand–metal systems are often not sufficiently general, while the need to synthesize the ligand and/or catalyst along with its use in organic solvents far outweigh the anticipated benefits to be realized. One example from our lab includes the recently introduced ligand HandaPhos (Scheme 1).^14^ Although its Pd complex exhibits impressive reactivity at 1000 ppm applied to “real” (*i.e.*, highly functionalized) substrates, the preparation of HandaPhos follows essentially the literature route to an oxaphosphole-containing skeleton, involving a lengthy 10-step synthetic sequence.^14^ To minimize step count, EvanPhos^15^ was subsequently introduced to showcase the new *meta*-oriented biaryl framework that both facilitates ppm level Pd-catalyzed couplings as well as its ease of synthesis. Although prepared in only two steps from readily available starting materials, reactivity is achieved with loadings of Pd in most cases within the 0.25–0.50 mol% range (*i.e.*, 2500–5000 ppm). Therefore, we looked to create a new, easily fashioned ligand that exhibits HandaPhos reactivity at similar 1000 ppm loadings, and may even extend the range of suitable reaction partners to both aryl and heteroaryl chlorides for use under mild, aqueous micellar conditions,^16–20^ using nanoreactors derived from the designer surfactant TPGS-750-M.^16*b*,21^ In this report we describe such a new ligand, N_2_Phos (**L1**), that upon complexation with Pd, meets these demanding guidelines (Scheme 1).

Typically, ligand design for Pd-catalyzed cross coupling reactions takes, in large measure, steric and electronic factors into consideration, as they can weigh heavily on the activity of the active palladium catalyst.^22–25^ While these factors are surely at play as well under micellar catalysis conditions, the switch from a traditional organic solvent medium to water presents new rules that must also be taken into account (*e.g.*, ligand lipophilicity).^26^ Thus, further derivatization of the biaryl skeletal of EvanPhos (Scheme 1), with the goal of increasing the activity of the resulting ligated Pd complex, focused on modifications ranging from increasing steric bulk around the phosphine moiety by altering the alkoxy residues (**L2–L4**) to enhancing steric interactions based on additional substitution on the naphthyl ring. Results from the former changes showed no significant rate increase, while replacement of an alkoxy group at C-2 in the naphthyl ring showed significant improvements in reaction rates relative to those observed upon chelation of Pd with EvanPhos. Additional steric crowding by placement of two dialkylamino groups at the C-2 and C-7 locations afforded a catalyst, N_2_Phos, with the highest activity. The final ratio of N_2_Phos (**L1**) to Pd(OAc)_2_ of 1.8 : 1 led to an active pre-catalyst capable of mediating SMCs at 1000 ppm of Pd under mild, aqueous conditions.

## Results and discussion

**Scheme 2:**
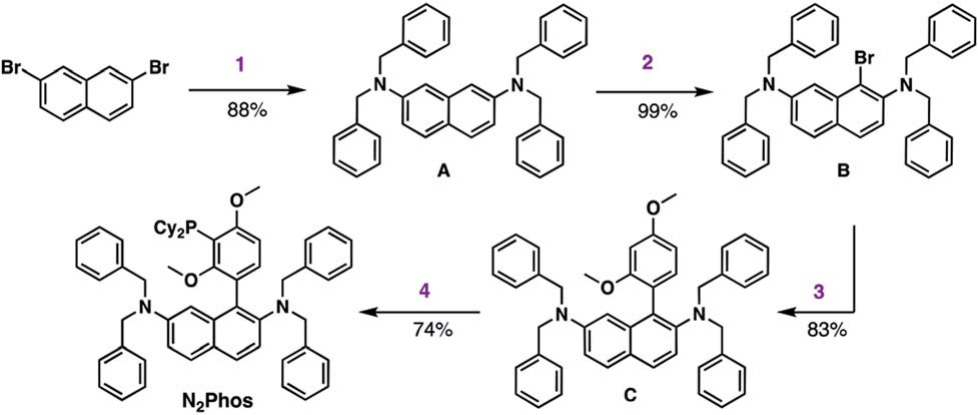
Synthesis of N_2_Phos. Step 1. 2,7-dibromonaphthalene, Pd-PEPPSI-iPent (2 mol%), dibenzylamine, KO-*t*-Bu, in toluene at 80 °C. Step 2. **A**, NBS, in DMF at 0 °C to rt. Step 3. **B**, 2,4-dimethoxyphenylboronic acid, Pd(OAc)_2_ (2 mol%), BI-DIME, K_3_PO_4_·H_2_O, dioxane, 80 °C. Step 4. **C**, *n*-BuLi, THF, 0 °C; then Cy_2_PCl, 0 °C to rt.

The synthesis of N_2_Phos (**L1**) is short, robust, and attractive (Scheme 2). Starting with commercially available 2,7-dibromonaphthalene, double amination gives the *N*-2,*N*-7-dibenzylamine intermediate **A** (88%; step 1). Subsequent bromination with NBS at 0 °C yields intermediate **B** that can be isolated without chromatography (99%; step 2). Alternatively, these first two steps can also be achieved using solventless mechanochemistry.^27^ The penultimate Pd-catalyzed SMC leads to biaryl intermediate **C** (83%; step 3). Finally, lithiation at 0 °C with *n*-BuLi in THF followed by phosphine insertion leads to the targeted ligand (74%; step 4) in good overall chemical yield (4-steps, 54%). This synthesis of N_2_Phos avoids both cryogenic conditions and unstable intermediates.

**Scheme 3:**
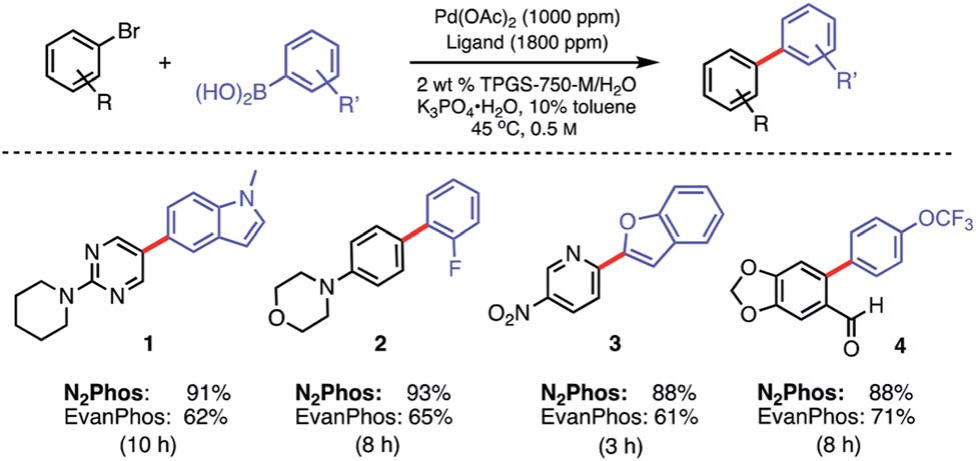
Comparison reaction between N_2_Phos and EvanPhos in aqueous media. Ligand : Pd (1.8 : 1) pre-complexed in toluene. Aryl halide (0.5 mmol), aryl boronic acid (0.75 mmol), K_3_PO_4_·H_2_O (0.75 mmol); 2 wt% TPGS-750-M/H_2_O : toluene (9 : 1) at 45 °C. Isolated yields reported.

Initial screening of the catalyst derived from N_2_Phos complexed with Pd(OAc)_2_ (1.8 : 1) *versus* that formed using EvanPhos under otherwise identical micellar conditions showed a significant increase in reactivity for the former in all substrates tested (Scheme 3). At 45 °C, the new ligand system led to good isolated yields of biaryl products, while the extent of conversion, and therefore, isolated yields of the same reactions, using EvanPhos was *ca.* 25% lower over the same time period. EvanPhos, however, showed comparable yields to N_2_Phos when given longer reaction times (16–24 h) for the substrates tested. One notable advantage of the N_2_Phos/Pd(OAc)_2_-derived catalyst is that pre-activation using commercially available DIBAL in toluene is no longer needed, as is the case with the corresponding EvanPhos-derived catalyst.^15^

**Scheme 4:**
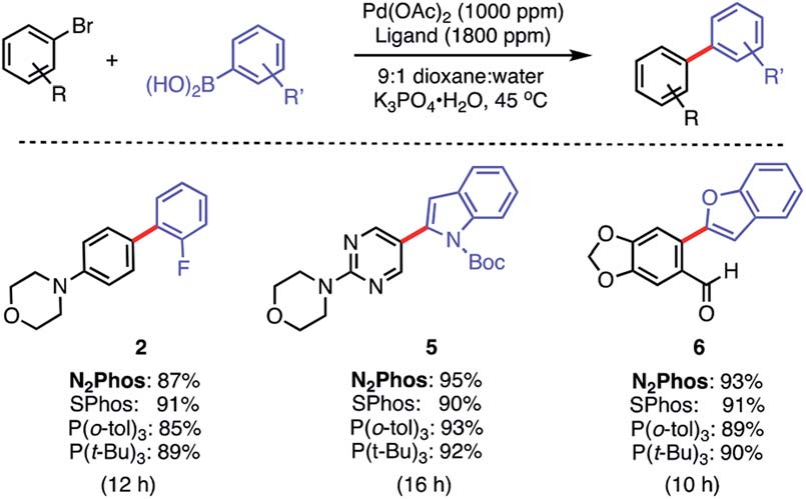
Comparisons of ligands N_2_Phos *vs.* SPhos, P(*o*-tol)_3_ and P(*t*-Bu)_3_ in *organic solvent*. Ligand : Pd (1.8 : 1) pre-complexed in toluene. Aryl halide (0.5 mmol), arylboronic acid (0.75 mmol), K_3_PO_4_·H_2_O (0.75 mmol); dioxane : water (9 : 1) [0.5 M] at 45 °C. Isolated yields reported.

The same pre-catalyst combination of N_2_Phos/Pd(OAc)_2_ could also be applied to SMCs at the 1000 ppm level, run under traditional conditions involving an organic solvent such as dioxane^28^ (Scheme 4). Relative to the corresponding reaction run under micellar catalysis conditions, these tended to require longer reaction times to reach completion (*e.g.*, formation of biaryl **2**; 8 h *vs.* 12 h; see Scheme 3*vs.*Scheme 4). When compared to commercially available SPhos,^29*a*^ tri-*t*-butylphosphine (P(*t*-Bu)_3_), and tri(*o*-tolyl)phosphine (P(*o*-tol)_3_), N_2_Phos shows similar reactivity in dioxane for the substrates tested. Hence, under such conditions in organic media, there appears to be no real benefit to using this ligand over those currently readily available.

**Scheme 5:**
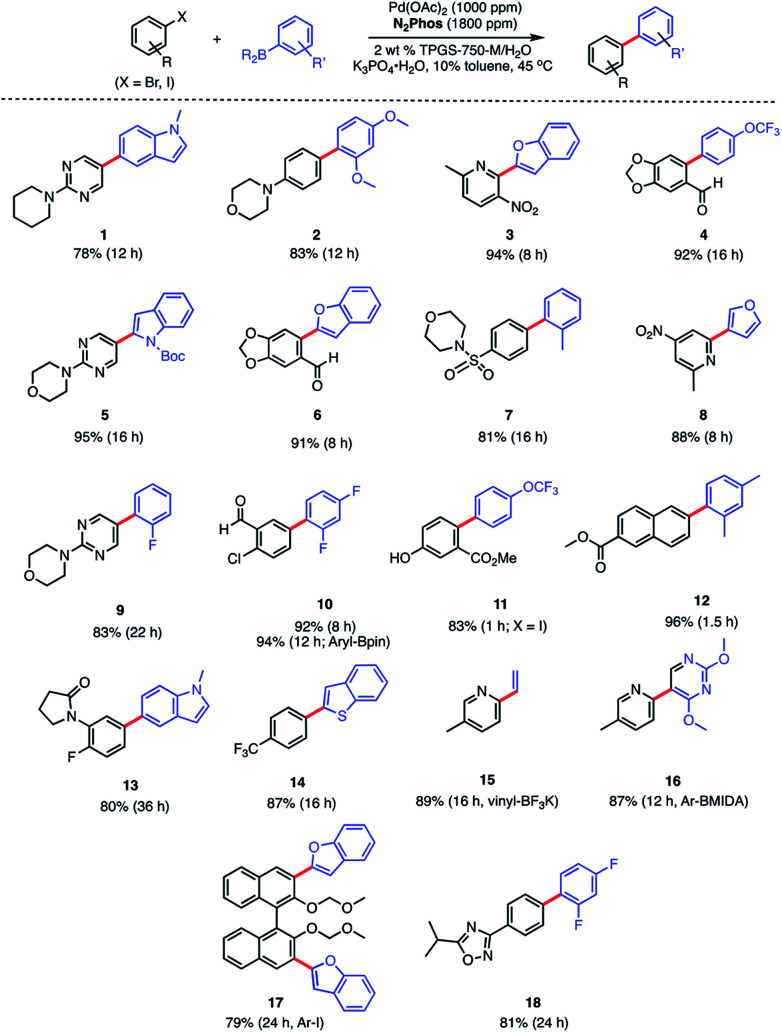
Substrate scope using N_2_Phos. Conditions: ligand : Pd (1.8 : 1) pre-complexed in toluene. Aryl halide (0.5 mmol), arylboronic acid (0.75 mmol), K_3_PO_4_·H_2_O (0.75 mmol); 2 wt% TPGS-750-M/H_2_O : toluene (9 : 1) [0.5 M] at 45 °C. Isolated yields reported.

In water, however, the choice of a 2 wt% aqueous solution of designer surfactant TPGS-750-M^21^ was based on prior efforts that showed it to be an especially enabling and recyclable medium for several types of reactions, such as peptide couplings,^32,33^ S_N_Ar,^34^ bio-catalytic processes,^35^ as well as a wide variety of metal-catalyzed cross coupling reactions. In the case of SMCs, high reactivity can be achieved using this new catalytic system (**L1**/Pd(OAc)_2_), with inclusion of K_3_PO_4_·H_2_O (1.5 equiv). This salt exhibited superior performance compared to other commonly employed bases, such as Et_3_N or K_2_CO_3_. Toluene was selected as co-solvent (10% by volume),^36^ given the poor solubility of some of the chosen substrates. Reactions run in the presence of this co-solvent appeared as nicely stirring emulsions. Broad functional group tolerance associated with either reaction partner is apparent, leading to good yields of coupled products. Hence, an assortment of challenging bromide partners can be coupled using this system at a loading of only 1000 ppm Pd (Scheme 5) including those bearing an ester (**12**), aldehyde (**4**), nitro (**3**, **8**), lactam (**13**), and sulfonamide (**7**) residues. Heteroaromatic bromides and boronic acids are also amenable, such as those containing pyridine, pyrimidine, benzofuran, indoles, furans, and benzothiophenes. Not surprisingly, aryl iodides are also converted quickly to the corresponding biaryls in excellent yields. Alternative boron-containing partners beyond boronic acids, including a Bpin derivative (leading to product **10**), a potassium trifluoroborate^30^ (affording product **15**), and a MIDA boronate^31^ (giving product **16**), could be used as well.

**Scheme 6:**
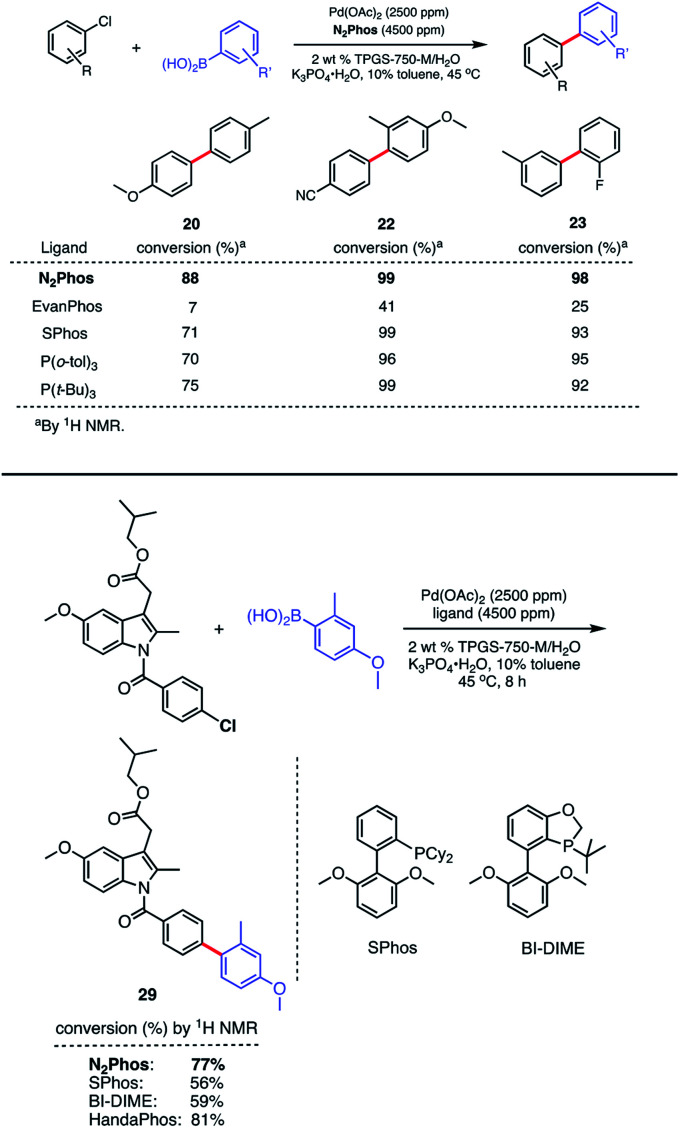
Comparison reactions between ligands and N_2_Phos (top). Comparison reactions between ligands on Pd involving a highly functionalized aryl chloride (bottom). Conditions: ligand : Pd (1.8 : 1) pre-complexed in toluene. Aryl halide (0.5 mmol), arylboronic acid (0.75 mmol), K_3_PO_4_·H_2_O (0.75 mmol); 2 wt% TPGS-750-M/H_2_O : toluene (9 : 1) [0.5 M] at 45 °C. ^1^H NMR conversion reported.

Although EvanPhos was designed as a quickly-synthesized, alternative ligand to HandaPhos for Pd-catalyzed SMCs, it has shown limited, if any, applicability to aryl chlorides, especially at the ppm level of precious metal.^14,15^ It is known to exist as a four-coordinate complex: (EvanPhos)_2_PdX_2,_ based on both an X-ray crystal structure and computational work.^15^ However, N_2_Phos-complexed palladium can be used for couplings with aryl chlorides regardless of electronic influences on the ring. This may be a result of the differential modes of complexation, where EvanPhos can spatially form 2 : 1 complexes with Pd. As shown, however, by modeling and calculations (*vide infra*), the far greater size of N_2_Phos makes 2 : 1 complexation significantly less favorable. Thus, aryl chloride precursors reflecting both electron-rich, electron-poor, and neutral educts afford product biaryls **20**, **22**, and **23**, respectively, although an increase in Pd loading to 2500 ppm (0.25 mol%) was needed (Scheme 6, top). Comparisons in each of these three cases were also made with EvanPhos, SPhos, P(*t*-Bu)_3_, and P(*o*-tol)_3_, the results from which indicate that N_2_Phos affords a catalyst that is equal to or better than these others in terms of reactivity at this loading of Pd under micellar catalysis conditions. The difference in conversion of aryl chlorides is quite noticeable, leading, *e.g.*, to biaryl **20**. Thus, while N_2_Phos gave 88% of product **20** after 8 hours, EvanPhos led to only 7% conversion after 8 h, and only 9% after 16 h (96% with N_2_Phos), after which time there was both unreacted aryl chloride present, as well as the product of protodeborylation.

The low yields with aryl chlorides appears, then, to be the result of a slow oxidative addition with EvanPhos compared to N_2_Phos. How these two ligands differ in their behavior and the possible role of steric effects is explored computationally (*vide infra*).

**Scheme 7:**
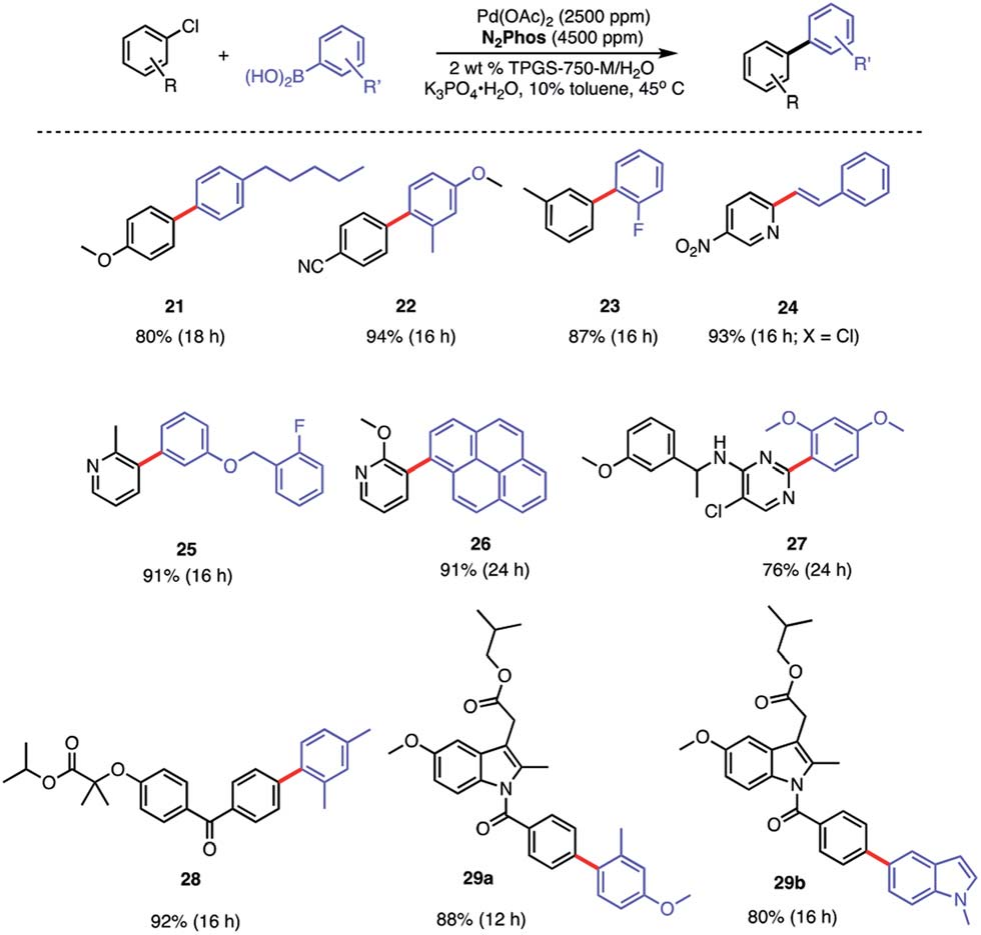
Substrate scope using N_2_Phos with aryl chlorides Conditions: ligand : Pd (1.8 : 1) pre-complexed in toluene. Aryl halide (0.5 mmol), arylboronic acid (0.75 mmol), K_3_PO_4_·H_2_O (0.75 mmol); 2 wt% TPGS-750-M/H_2_O : toluene (9 : 1) [0.5 M].

A similar outcome was noted in the case of the ester of indomethacin (Scheme 6, bottom), where a better result was observed in comparisons with catalysts derived from either SPhos or BI-DIME.^24,37^ Moreover, that the results between N_2_Phos and HandaPhos were within experimental error further suggests that the derived catalysts in each case are roughly comparable in activity imparted to their 1 : 1 complexes with Pd. The biaryls prepared and isolated from a more general study on the scope of these couplings with various aryl/heteroaryl chlorides are illustrated in Scheme 7. Several heterocyclic arrays, such as those in products **24–27** were formed under the same coupling conditions used for analogous bromides, although somewhat increased reaction times and catalyst loadings were needed to achieve high levels of conversion, and hence, good isolated yields.

**Scheme 8:**
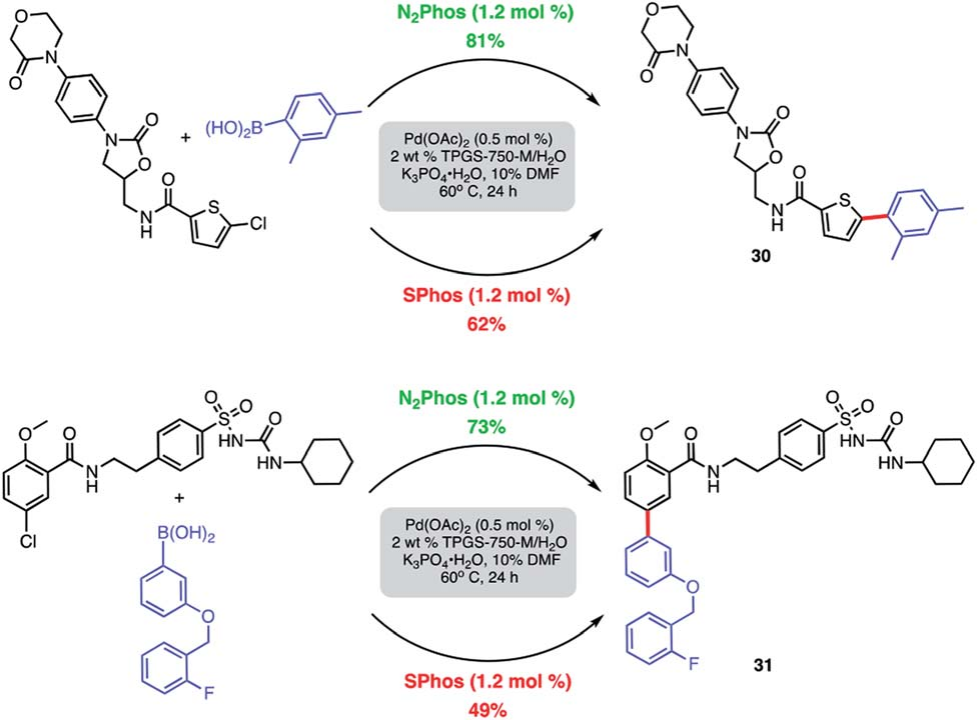
Comparison of N_2_Phos and SPhos with highly functionalized aryl/heteroaryl chlorides. Isolated yields.

Modified known targets that coupled smoothly included fenofibrate (leading to **28**), used to treat high cholesterol, and previously mentioned indomethacin (giving products **29a-b**), an anti-inflammatory drug. Very highly functionalized and especially challenging chloroarenes also participated, including rivaroxaban (a medication used to prevent blood clots) affording product **30**, and glibenclamide (used to treat type 2 diabetes) leading to biaryl **31** (Scheme 8). Palladium loadings of 5000 ppm (0.50 mol%) together with a reaction temperature of 60 °C were required, along with water-miscible DMSO (10%) as co-solvent. Direct comparisons with the most commonly used, state-of-the-art Pd/ligand combination (*i.e.*, SPhos),^29*b*^ clearly indicated that under otherwise identical micellar conditions and ppm levels of Pd, (N_2_Phos)Pd gave far better results.

**Scheme 9:**
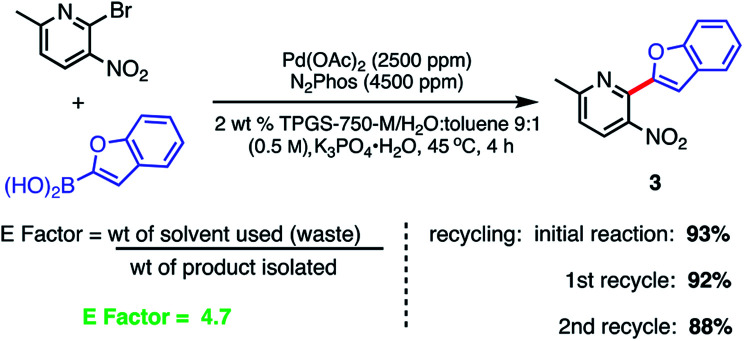
Recycling study and E Factor determination. Conditions: ligand : Pd (1.8 : 1) pre-complexed in toluene. Aryl halide (0.5 mmol), aryl boronic acid (0.75 mmol), K_3_PO_4_·H_2_O (0.75 mmol); 2 wt% TPGS-750-M/H_2_O : toluene (9 : 1) at 45 °C.

Recycling of the 2 wt% aqueous surfactant medium could be readily achieved as a means of reducing the amount of organic waste produced, as measured by E factors (Scheme 9).^38,39^ Each recycle was carried out starting with an in-flask extraction of the aqueous reaction with ethyl acetate, followed by introduction of additional catalyst (*i.e.*, an additional 1000 ppm Pd) into the reaction medium. Based on this representative biaryl coupling, an E factor of only 4.7 is indicative of the limited amounts of solvent waste generated. This value is far below those typically seen based on usage of organic solvents as both reaction medium and for extraction purposes.

**Fig. 1:**
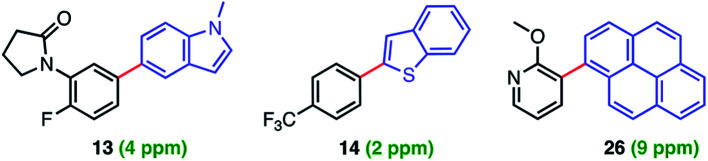
Residual levels of Pd in products **13**, **14**, and **26**.

Levels of residual Pd found in the products using ppm loadings of Pd catalysts enabled by aqueous nanomicellar technology are typically below the 10 ppm FDA allowed limit. This is yet another major advantage associated with this chemistry in water which avoids the time and expense of scavenging metal impurities. In this case, analyses of three products *via* ICP-MS led to observed levels all within the targeted range (Fig. 1).^7^

**Scheme 10:**
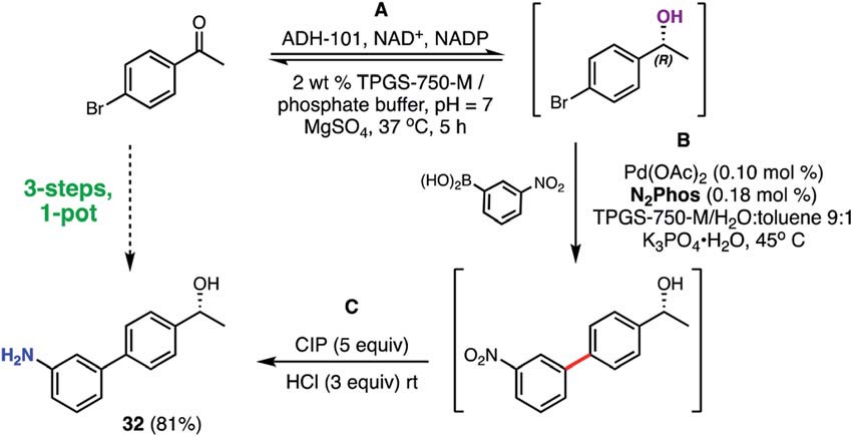
3-Step, 1-pot sequence involving an initial enzymatic reduction followed by a SMC, and then a nitro group reduction.

A representative 3-step, 1-pot sequence illustrative of the infinite opportunities now available for combining chemo- and bio-catalysis in water^35^ is illustrated in Scheme 10. Thus, following an enzymatic reduction of *p*-bromoacetophenone in an aqueous buffered medium (step **A**), the product (without isolation) is then used for a 1000 ppm N_2_Phos Pd-catalyzed Suzuki–Miyaura coupling (step **B**). Again, without processing the newly formed biaryl, nitro group reduction utilizing carbonyl iron powder (CIP)^40^ (step **C**) ultimately affords nonracemic aminoalcohol **32** in 81% overall yield.

Density functional theory (DFT) calculations and an X-ray crystal structure were obtained to gain insight into the structural factors that contribute to the greater reactivity of the N_2_Phos/Pd(OAc)_2_-derived species formed in solution relative to the corresponding catalyst derived from EvanPhos.

**Fig. 2:**
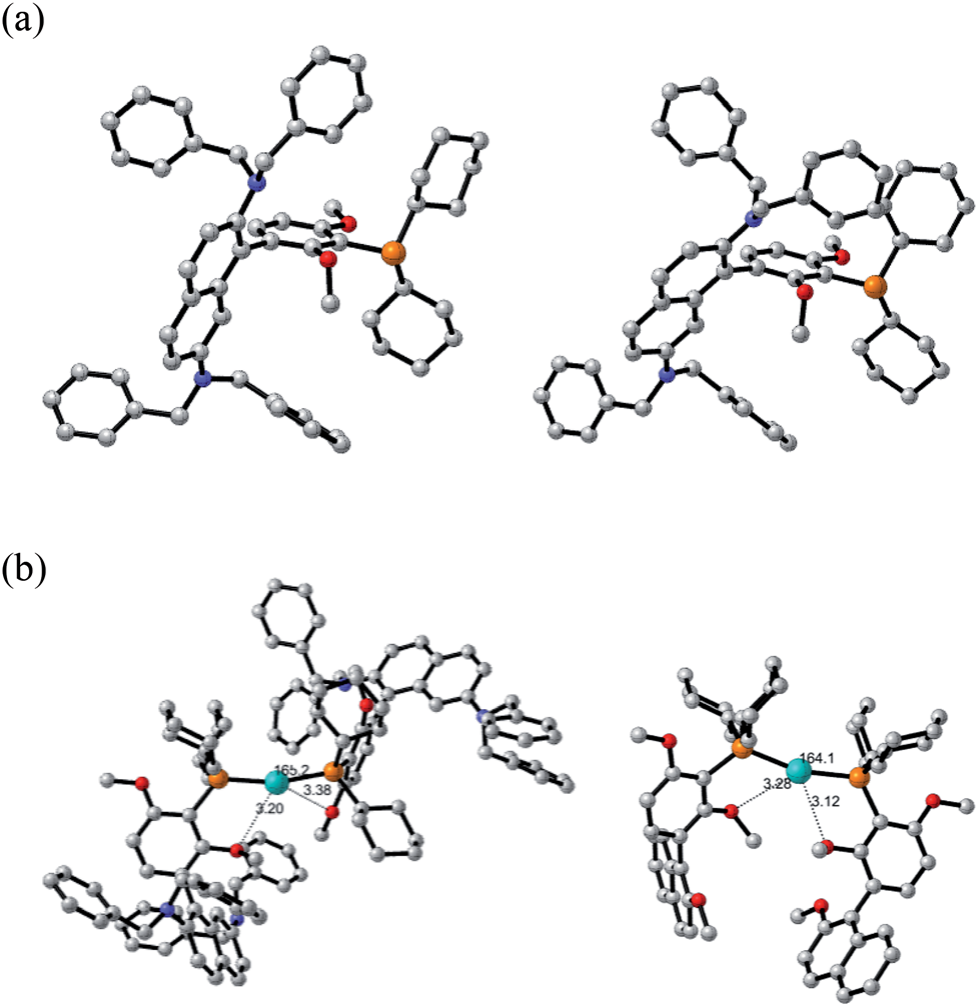
(a) X-ray (left) and B3LYP/6-31G(d) calculated (right) structures of N_2_Phos. Atom colors: nitrogen, blue; oxygen, red. Hydrogens are omitted for clarity. (b) B3LYP/6-31G(d)-SDD(Pd) calculated structures of sterically hindered, but lowest-energy palladium zero intermediates for (N_2_Phos)_2_Pd (left, P–Pd–P angle = 165.2°); (EvanPhos)_2_Pd (right, P–Pd–P angle = 164.1°). Atom colors: nitrogen, blue; oxygen, red; palladium, teal. Hydrogens are omitted clarity.

The X-ray crystal structure of N_2_Phos is shown in Fig. 2a. Two notable features include the dihedral angle between the biaryl groups of 79.47° and the orientation of the two *N*,*N*-dibenzyl moieties, the steric requirements for each forcing the aromatic rings of the biaryl unit to be almost perpendicular. Quantum-calculated geometry optimizations of the free ligand N_2_Phos at the B3LYP/6-31G(d) level were completed for seventeen of the most reasonable conformations. The next most stable, by 0.60 kcal mol^−1^, of these calculated structures had the same basic conformation as in the X-ray crystal structure. At the B3LYPD3/6-31+G(d,p) level with D3 empirical dispersion corrections and a larger basis set and at the M06/6-31+G(d,p) level, this structure was not always the lowest in energy but was within about 2 kcal mol^−1^ of being the lowest energy. Small computational inconsistencies and/or small crystal-packing effects could account for these differences between experiment and theory. Structural parameters for the X-ray structure and for the calculated structures at the three levels for the same basic conformation gave a close correlation between experiment and theory at all levels for bond distances and angles. Average errors were about 0.010 Å in some selected distances and 2.0–2.1° for selected angles. For selected dihedral angles that determine the exact conformation, the differences were larger, averaging 10–14°, as expected since these bond rotations have shallow energy wells. The B3LYPD3/6-31+G(d,p) level of theory gave slightly smaller geometry errors than the other two levels of theory. These geometry comparisons between theory and experiment suggest that the levels of theory chosen might also be expected to give reliable reaction energies for interconversion of the conformers (see ESI-2 for details†).

The structures and relative energies of the pre-reductive elimination intermediates, Ph_2_PdL, for SMCs between two simple phenyl rings to form biphenyl were calculated at the B3LYP/6-31G(d)-SDD(Pd) level using a conformation for the ligand close to the experimental conformation of the free ligand from the X-ray structure for N_2_Phos and for a low-energy conformer for EvanPhos.^15^ The calculations reveal that intermediates from both ligands form a square planar complex, as expected for Pd(ii). Key distinguishing features, however, show a C–Pd–C angle of 82.17°, along with a bond length of 2.453 Å between palladium and the methoxy oxygen on the resorcinol ring of N_2_Phos, well in the range of the van der Wall's radii of the two elements indicative of a weak interaction (Fig. 1b). The former observation highlights a notable difference as compared with the corresponding EvanPhos-containing intermediate possessing a greater C–Pd–C angle of 87.68°. The enhanced proximity of the two phenyl rings could translate into an increased rate of reductive elimination for the N_2_Phos-containing intermediate compared with that with EvanPhos in this complex.^41^ The explanation can be attributed to the *N*,*N*-dibenzyl moieties of the naphthyl ring, such that the –NBn_2_ residues significantly crowd the available space around palladium, thereby forcing the two phenyl rings into closer proximity and increasing their rate of reductive elimination. No such phenomenon is in play with EvanPhos. The steric requirements of the N_2_Phos ligand make the formation of a 2 : 1 complex with diphenylpalladium impossible, with no energy minimum found for the Ph_2_Pd(N_2_Phos)_2_ complex, though less hindered versions lacking dibenzylamino substituents were able to form such 2 : 1 complexes.

**Scheme 11:**
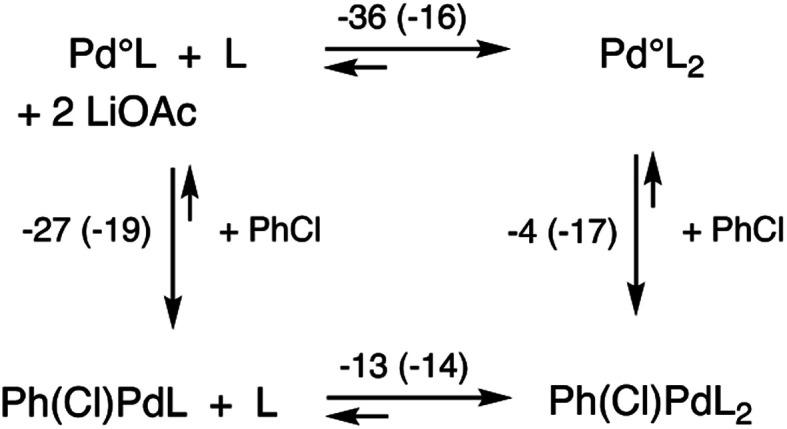
Reaction scheme for oxidative addition with free energies at 298 K in kcal mol^−1^ with EvanPhos (and N_2_Phos energies in parentheses) ligands at the M06D3/6-31+G(d,p)-SDD(Pd) level of theory.

More important, however, than the reductive elimination step in our analysis as to why the N_2_Phos ligand gives improved yields over EvanPhos with aryl chlorides is the possibly rate-limiting oxidative addition step.^42^ Oxidative additions of ligated Pd^o^ (LPd^o^ and L_2_Pd^o^; Fig. 2b) to chlorobenzene forming PhPd(Cl)L and PhPd(Cl)L_2_, respectively, were studied with both EvanPhos and N_2_Phos ligands. Oxidative addition was found to be much more downhill in free energy when carried out with monoligated species LPd^o^ in accord with literature expectations.^43,44^ The oxidative addition reaction summarized in Scheme 11 the computational results with M06D3/6-31+G(d,p)-SDD(Pd) level free energies. The free energies for the oxidative addition are 8 kcal mol^−1^ more downhill for the monoligated species LPd^o^ for EvanPhos than for N_2_Phos. This seems to contradict our presumption that catalysis by the Pd-complexing N_2_Phos ligand would be favored, although it does follow based on steric arguments. The equilibria for further ligation of LPd^o^ to form L_2_Pd^o^ have free energies of reaction of −36 and −16 kcal mol^−1^ for the EvanPhos and N_2_Phos ligands, respectively. Thus, the di-ligated L_2_Pd^o^ is the predominant form of the Pd^o^ catalyst, and the overall reaction to form PhPd(Cl)L from L_2_Pd^o^ is predicted to be downhill by 3 kcal mol^−1^ for N_2_Phos and uphill by 9 kcal mol^−1^ for EvanPhos, leading to a strong 12 kcal mol^−1^ overall preference for the N_2_Phos reaction. Inclusion of solvation by toluene computationally using a continuum model makes little difference, showing the same 12 kcal mol^−1^ preference (ESI for details†).

Electron-donating effects of the dibenzylamino nitrogens were also considered as a possible influence on the electron density in the vicinity of the phosphine group and perhaps, catalyst reactivity. Natural population analysis calculations at several levels of theory on the free ligands and diphenylpalladium complexes for EvanPhos, N_2_Phos, and an N_2_Phos with the nitrogens replaced with CH groups showed little or no regular variation of charge densities at phosphorus or palladium. The fact that the dihedral angles between the planes of the biaryl groups in the ligands is near 80° for the free ligands and palladium complexes would certainly be expected to seriously diminish any putative π-donating electronic effect. This suggests that the efficacy of N_2_Phos is likely the result of steric, rather than any significant electronic effects.

## Conclusions

In summary, a next-generation ligand, N_2_Phos, featuring judiciously positioned *N*,*N*-dibenzylamine substituents on a naphthalene ring as part of a biaryl array, has been found to impart considerable catalytic activity to its derived palladium complex. This new catalyst facilitates Suzuki–Miyaura reactions at low loadings of palladium of both aryl bromides and iodides, and, most notably, on aryl chlorides. This represents a significant advance in cross-coupling chemistry, given that such critical reactions can involve complex educts, take place at the ppm level of Pd, and are uniquely positioned for use under environmentally responsible conditions (*i.e.*, in water under ambient-like conditions). Modeling studies suggest that the observed facile participation of aryl chlorides towards a Pd catalyst containing the N_2_Phos ligand may be due to steric effects stemming from the locations of the two NBn_2_ residues within this biaryl ligand, thereby destabilizing the 2 : 1 L_2_Pd^o^ complex and making formation of the more reactive 1 : 1 LPd^o^ complex more accessible when compared to EvanPhos. The monoligated 12-electron (N_2_Phos)Pd catalyst is then especially prone towards oxidative addition to aryl chlorides.^45^

## Conflicts of interest

The authors have no conflicts of interest to declare.

## Supplementary Material

SC-011-D0SC00968G-s001

SC-011-D0SC00968G-s002
