# Supplementary material for: N2Phos – an easily made, highly effective ligand designed for ppm level Pd-catalyzed Suzuki–Miyaura cross couplings in water
Source: Chem Sci. 2020 May 7;11(20):5205–12. doi: 10.1039/d0sc00968g (PMC8159421; doi:10.1039/d0sc00968g)
Supplement: SC-011-D0SC00968G-s001 [file SC-011-D0SC00968G-s001.pdf]

Electronic Supplementary Material (ESI) for Chemical Science.
This journal is © The Royal Society of Chemistry 2020

N_2Phos. An easily made, highly effective ligand designed for ppm level Pd-catalyzed Suzuki-Miyaura cross couplings in water

Nnamdi Akporji,[1] Ruchita Thakore,[1] Margery Cortes-Clerget,[1] Joel Anderson,[1] Donald H. Aue,[1*] Fabrice Gallou,[2] and Bruce H. Lipshutz[1*]

[1]Department of Chemistry and Biochemistry, University of California, Santa Barbara, CA 93106 USA
[2]Novartis Pharma, Basel, Switzerland

Phone: 805-893-2521
Fax: 805-893-8265
Email: aue@chem.ucsb.edu
Email: lipshutz@chem.ucsb.edu
Website: http//:www.chem.uscb.edu/lipshutzgroup

Supporting Information 2. Computational Studies

Computational Results

Theoretical Methods. For molecules with no palladium, complete geometry optimizations were carried out using density functional theory (DFT) methods at B3LYP/6-31G(d), B3LYPD3/6-31+G(d,p) with Grimme's D3 empirical dispersion corrections, and M06/6-31+G(d,p) levels[1-3]. The basis set used for palladium was the SDD[4,5] effective core potential. B3LYP/6-31G(d)(C,H,N,O,Cl)-SDD(Pd) level geometry optimizations along with frequency calculations were carried out in order to verify that the stationary points thus obtained were true minima and to determine thermodynamic parameters for the determination of reaction energetics. B3LYPD3/6-31+G(d,p)(C,H,N,O,Cl)-SDD(Pd) with D3 empirical dispersion corrections single-point calculations, scf=tight, were also done to get reaction energies. B3LYP/6-31G(d) and M06/6-31+G(d,p) calculations were done with the 5d option to match the SDD calculations with 5 d orbitals and done with the default Gaussian 'int=ultrafine' option.

Thermochemical data were calculated with zero-point energy corrections from scaled frequencies using a scaling factor of 0.99 for zero-point energies.[6] A scaling factor of 1.00 for frequencies for the thermal and entropy terms was used.[6ab] The quasiharmonic approximation was used for low frequencies to calculate entropies to

avoid the large distortions found when many low-frequency vibrations are present in organometallic compounds.[6cd] Thus, frequencies below 100 cm^{-1} were treated as free rotors rather than by the harmonic approximation in calculating entropies. We have chosen a polarized continuum model in toluene (to mimic the interior of micelles) solvent for our calculations using the SMD method of Truhlar and Cramer.[7] In prior work we found that toluene solvent best mimics the micellar environment of our palladium complexes.[8] All calculations were performed using the Gaussian 16 program suite.[9]

Thermochemical data and Cartesian coordinates are found in Tables S3 and S4 at the end of this document.

Theoretical Results. Density functional theory (DFT) calculations and an X-ray crystal structure were obtained to gain insight into the structural factors that contribute to the greater reactivity of the N_2Phos/Pd$(OAc)_2$-derived species formed in solution relative to the corresponding catalyst derived from EvanPhos. The X-ray crystal structure of N_2Phos is shown in Figure S1. Two features that are notable from the crystal structure are the dihedral angle between the biaryl groups of 79.47° and the orientation of the two *N*,*N*-dibenzyl moieties whose steric requirements force the aromatic rings of the biaryl system to be almost perpendicular. Geometry optimizations of the free ligand N_2Phos at the B3LYP/6-31G(d) level were completed for 18 of the most reasonable-looking conformations,. **A** to **O** and **AA, AB** and **AC**. The conformer **A** that was the second most stable, only 0.60 kcal/mol higher in energy than the conformational varient **AB**, of these calculated structures had the same basic conformation **A** as in the X-ray crystal structure and was chosen as the best ligand structure for later energy comparisons. See Table S1 for a quantitative comparison of structural parameters. At the B3LYPD3/6-31+G(d,p) level with D3 empirical dispersion corrections and a larger basis set and at the M06/6-31+G(d,p) level, structure **A** was not always the lowest in energy, but within about 2 kcal/mol as shown in Table S2. Three other conformers, **B**, **C**, **D** and **H** were as much as 2.4 kcal/mol lower in energy than conformer **A**. Small computational inconsistencies and/or small crystal-packing effects could account for these differences between experiment and theory.

Comparison of the drawings for the the X-ray structure and the B3LYP/6-31G(d) optimized structure for conformer **A** are shown in Figure S1. The geometries are very similar, but differ some in the rotational angles about some sigma bonds of the benzyl and cyclohexyl groups. Structural parameters for the X-ray structure and for the calculated structure are shown in Table S1. At the three levels of theory for the same basic conformation **A**, close correlation between experiment and theory was found at all levels for bond distances and angles. Average errors were about 0.010 Å in some selected distances and 2.0-2.1° for selected angles. For selected dihedral angles that determine the exact conformation, the differences were larger, averaging 10-14°, as expected since these bond rotations have shallow energy wells. The B3LYPD3/6-31+G(d,p) level of theory gave slightly smaller geometry errors than the other two levels of theory. These geometry comparisons with between theory and experiment suggest that the levels of theory chosen might also be expected to give reliable reaction energies for the interconversion of the conformers in Table S2.

Table S1. Structural parameters for X-ray and optimized structures at B3LYP/6-31G(d) [5d], B3LYPD3/6-311+G(d,p), and M06/6-311+G(d,p) [5d] levels for conformer **A** of N_2Phos.

	B3LYP	B3LYPD3	M06	X-Ray
distances:				
C1-C1' (biaryl)	1.496	1.491	1.483	1.489
=C-P	1.870	1.865	1.856	1.840
=C2-N	1.423	1.422	1.414	1.426
=C7-N	1.397	1.393	1.389	1.394
angles:				
CH-P-CH	101.69	102.57	103.17	105.64
=C-P-CH	100.75	98.28	98.08	101.24
=C-P-CH	104.23	103.70	103.18	103.30
CH2-N-CH2	114.08	113.70	113.32	111.86
=C2-N-CH2	118.00	117.65	118.88	115.39
=C2-N-CH2	116.32	115.99	116.56	113.17
CH2-N-CH2	115.01	115.36	115.54	115.15
=C7-N-CH2	122.78	122.39	122.21	120.35
=C7-N-CH2	122.18	121.77	121.70	119.40
dihedral angles:				
(N)C-C-C-C(O)	69.12	75.76	74.64	79.47
=C1-C2-N-CH2	68.36	69.36	64.70	72.90
=C-CH2-N-C2=	151.82	155.47	153.61	163.61
=C-CH2-N-C2=	77.00	74.35	76.70	61.21
=C-CH2-N-C7=	103.82	95.05	93.43	78.69
=C-CH2-N-C7=	98.37	94.54	94.98	70.13
H-C-P-C=	-61.99	-50.08	-55.31	-27.58
H-C-P-C=	83.16	79.85	79.41	78.23
(P)C-C2'-O-CH3	-93.40	-97.63	-93.45	-89.82
(P)C-C4'-O-CH3	-179.96	-175.26	164.21	-175.54

Table S2. Calculated relative electronic energies, enthalpies, and free energies at 298 K for selected conformers of N_2Phos optimized at B3LYP/6-31G(d) [5d], B3LYPD3/6-31+G(d,p), and M06/6-31+G(d,p) [5d] levels of theory.

Conformer:	B3LYP			B3LYPD3			M06
	$\Delta E^o e$	ΔH^o_{298K}	ΔG^o_{298K}	$\Delta E^o e$	ΔH^o_{298K}	ΔG^o_{298K}	$\Delta E^o e$
**A**	0.00	0.00	0.00	0.00	0.00	0.00	0.00
**B**	0.24	0.06	0.05	-1.16			-2.38
**C**	1.13	0.80	0.90	-1.58	-1.92	-1.98	-1.89
**D**	1.29	1.12	1.14	0.50	0.24	0.06	-0.46
**E**	1.51	1.39	1.53	1.56	1.36	1.29	
**F**	1.60	1.39	1.48	1.85			
**G**	1.75	1.73	1.57	0.00	0.00	0.00	-0.08
**H**	2.11	1.90	2.12	-1.46	-1.66	-1.45	-1.51
**I**	3.85	3.62	3.64	2.24			
**J**	3.91	3.78	3.81	4.01	3.69	3.68	3.15
**K**	3.91	3.78	3.81	4.29			
**L**	4.46	4.29	4.17	2.01	1.90	1.62	
**M**				2.24	2.15	2.03	
**N**				4.88	4.34	3.91	
**O**	8.54	8.25	8.25	6.01			
**AA**	0.83	0.79	0.91	-0.20			-0.52(D3)
**AB**	-0.60	-0.66	-0.61	-1.71			-1.07(D3)
**AC**	1.36	1.27	1.19	-0.12	-0.17	-0.53	

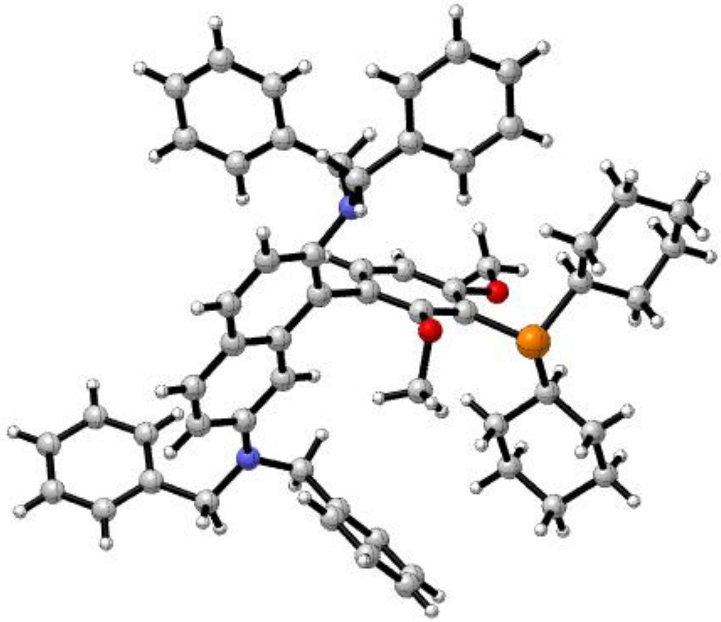

X-ray crystal structure of N_2Phos.

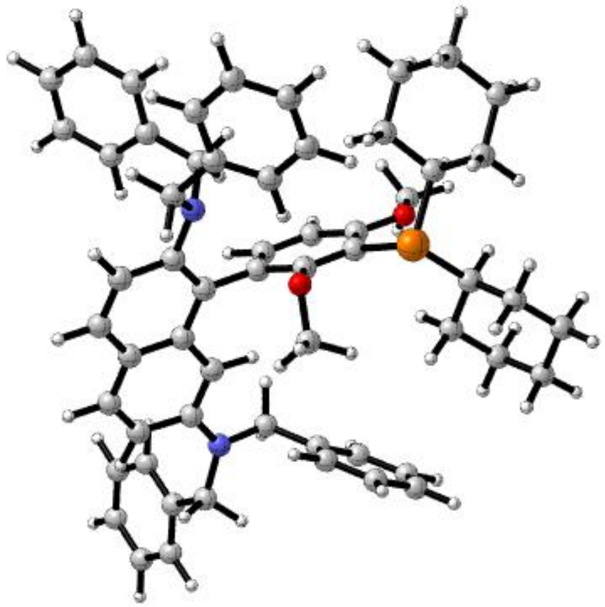

B3LYP/6-31G(d) [5d] optimized structure for conformer **A** of N_2Phos.

Figure S1. Structures of ligands. Atom colors: nitrogen, blue; oxygen, red; palladium, teal.

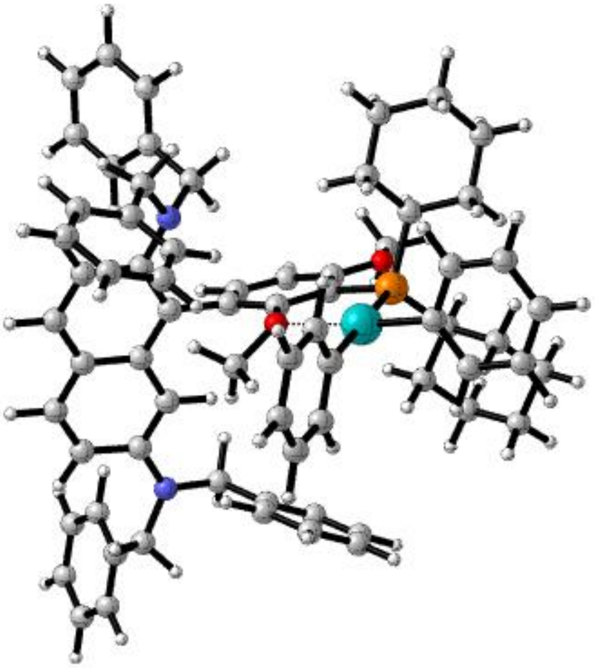

B3LYP/6-31G(d)(SDD) [5d] optimized structure for conformer **APdPh2** of N_2Phos. O-Pd distance=2.453 Å, C-Pd-C angle=82.17^o.

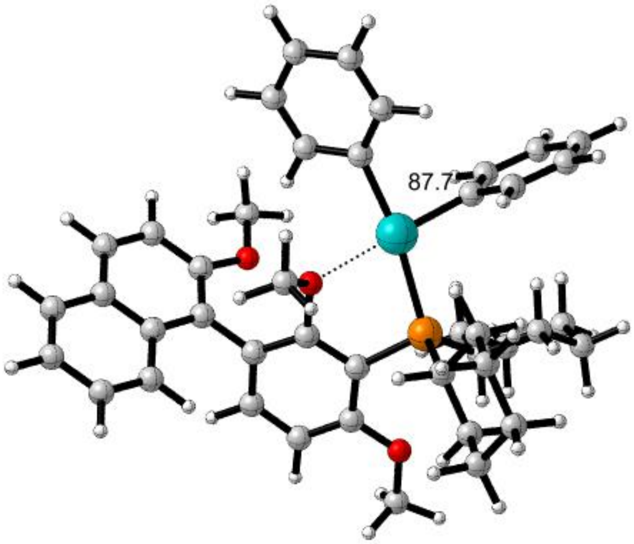

B3LYP/6-31G(d)(SDD) [5d] optimized structure for a low-energy conformer of EvanPhos., **EvanPhosPdPh$_2$**, O-Pd distance=2.330 Å, C-Pd-C angle=87.68^o.

Figure S2. Structures for $PdPh_2$ pre-reductive elimination intermediates. Atom colors: nitrogen, blue; oxygen, red; palladium, teal.

The structures and relative energies of the pre-reductive elimination intermediates for Suzuki-Miyaura couplings between two simple phenyl rings to form biphenyl were examined. The first calculation at the B3LYP/6-31G(d)-SDD(Pd) level was optimized using conformation **A** for the ligand N_2Phos, close to the X-ray structure of the free ligand. The resulting optimized structure, **APdPh$_2$** for N_2Phos, is shown in Figure S2. **APdPh$_2$** has a C-Pd-C angle of 82.17°, along with a bond length of 2.453 Å between palladium and a methoxy oxygen on the resorcinol ring of N_2Phos, well in the range of the van der Wall's radii of the two elements indicative of a weak bonding interaction. This structure was the lowest energy found for a diphenylpalladium complex with N2Phos, but no systematic search was carried out for all conformers. Two other higher-energy structures were found with ligand conformations close to that for the calculated structure **J** for N2Phos and are shown in Table S3. The first of these was 2.95 kcal/mol higher in electronic energy than **APdPh$_2$**, and the other 17.67 kcal/mol higher. The first had a C-Pd-C angle of 82.45° and the second was a dramatically different structure with a C-Pd-C angle of 160.0° and an O-Pd distance of 3.065 Å, very near the sum of the van der Waals radii.

A corresponding calculation was done for the low-energy conformer shown in Figure S2 for EvanPhos, **EvanPhosPdPh$_2$**.[10] The calculations reveal that intermediates from both ligands form a square planar complexes, as expected for Pd(II). The more sterically encumbered N_2Phos intermediate has a C-Pd-C angle substantially smaller than the corresponding angle of 87.68° in the EvanPhos-containing intermediate (Figure S2). Both show coordination to a methoxyl group, but with a shorter O-Pd distance of 2.330 Å. The enhanced proximity of the two phenyl rings in **APdPh$_2$** would be expected to result in an increased rate of reductive elimination as previously noted in our prior work.[10] This difference between the ligands can be attributed to the two *N,N*-dibenzyl moieties of the naphthyl ring, such that one -NBn_2 residue significantly crowds the available space around palladium, thereby forcing the two phenyl rings into closer proximity and likely increasing their rate of reductive elimination.

Several alternative ligands seen in Figure S3 were screened for use as potential catalysts by calculation of structures for additional pre-reductive elimination intermediates with optimizations at the B3LYP/6-31G(d)-SDD(Pd) level. The first of those structures, **QPdPh$_2$** (**N,N-dibenzyl-2-aminoNPhosPdPh$_2$**) has an NPhos ligand with a single dibenzylamino group in the 2 position of the naphthyl ring. The most stable structure found for this intermediate, shown in Figure S4 is similar to that of the N_2Phos intermediate **APdPh2** in Figure S2, but not exactly the same conformation as **A**. It has a larger 86.28° C-Pd-C bond angle and a shorter O-Pd distance, 2.338 Å, akin to that in **EvanPhosPdPh$_2$**. This suggests again that the rate of the coupling reaction between the phenyl groups might be more like that for the EvanPhos than for the N2Phos ligands. Another much higher energy structure calculated for **QPdPh$_2$** (**N,N-dibenzyl-2-aminoNPhosPdPh$_2$**) a adopted the same new T-shaped square planar Pd shape, see Figure S5, with a C-Pd-C angle of 161.23° and an O-Pd distance of 3.055 Å, as found above for N_2Phos and now 19.38 kcal/mol less stable in its electronic energy. Two other similar structures with electronic energies now higher by 21.20 and 25.26 kcal/mol and with somewhat different conformations also had

large C-Pd-C angles (161.02° and 160.59°, respectively). Curiously, the extra steric bulk of the extra 7-NBn_2 group in **$APdPh_2$** with the N_2Phos ligand does not make formation of these near 160° forms substantially less stable than for the **$QPdPh_2$** structures, apparently because the 7-NBn_2 is far enough from the phenyl groups in the 160° forms that it has little steric effect.

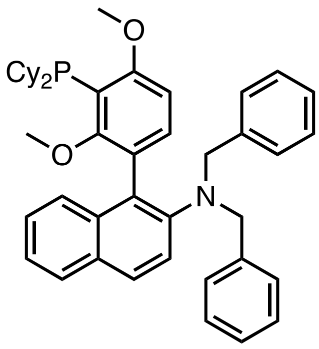

Q

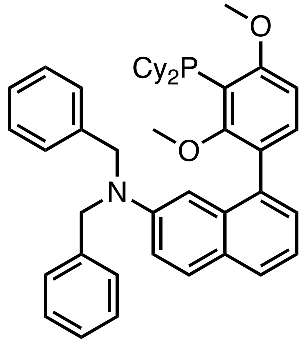

R

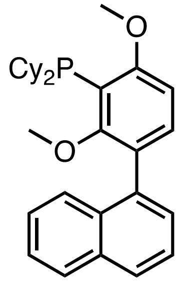

X

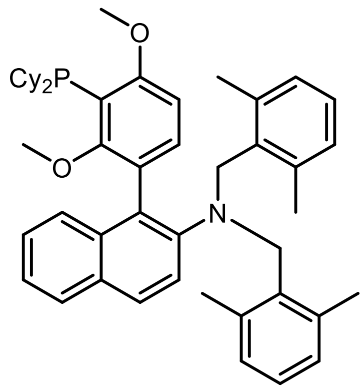

S

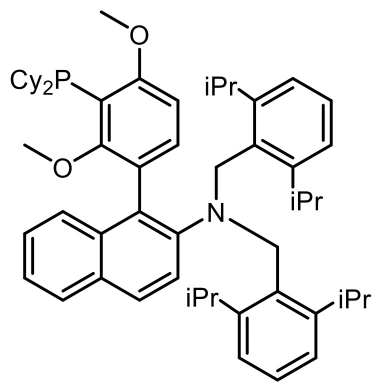

T

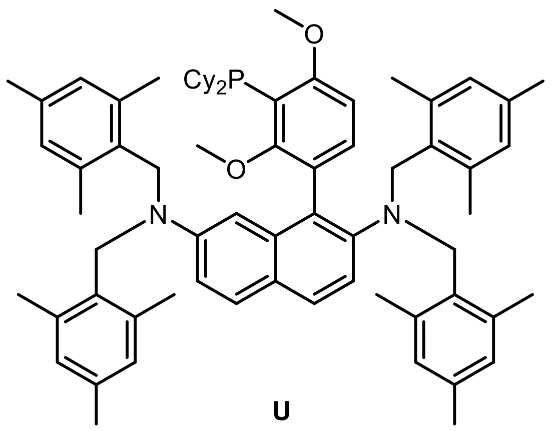

U

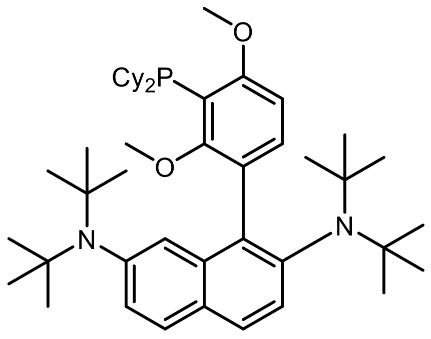

V

Figure S3. Structures for various alternative ligands.

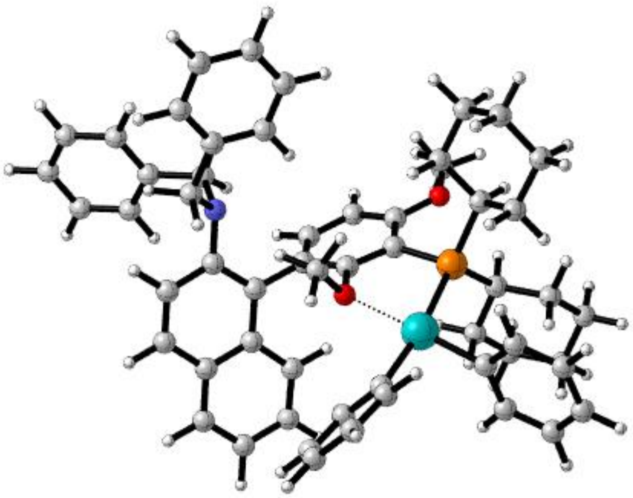

Figure S4. B3LYP/6-31G(d)(SDD) [5d] optimized structure (two viewpoints) for the more stable conformer **QPdPh$_2$** of **N,N-dibenzyl-2-aminoNPhosPdPh$_2$**. O-Pd distance=2.338 Å, C-Pd-C angle=86.28°. Atom colors: nitrogen, blue; oxygen, red; palladium, teal. Cartesian coordinates in Table S4 below are for enantiomer.

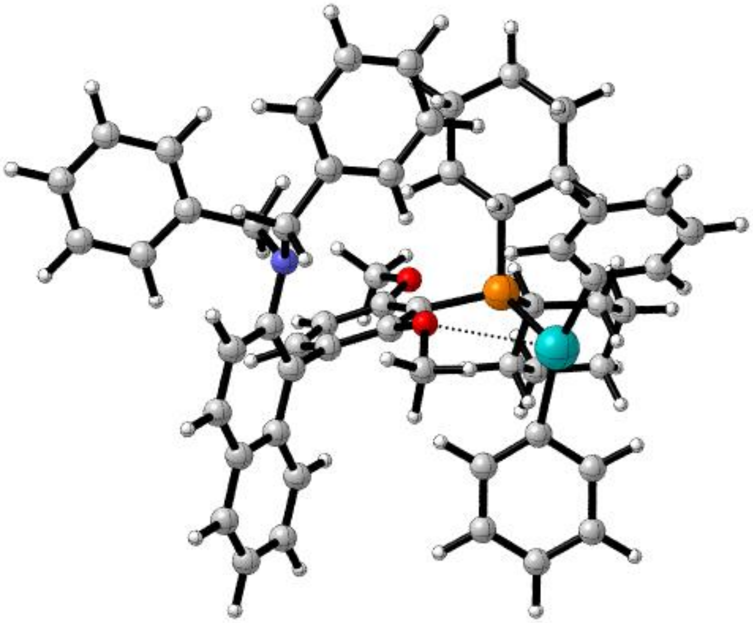

Figure S5. B3LYP/6-31G(d)(SDD) [5d] optimized structure for a less stable form of **QPdPh$_2$** of **N,N-dibenzyl-2-aminoNPhosPdPh$_2$**. O-Pd distance=3.055 Å, C-Pd-C angle=161.23°. Atom colors: nitrogen, blue; oxygen, red; palladium, teal. Cartesian coordinates in Table S4 below are for enantiomer.

As with **QPdPh$_2$** with a single 2-NBn$_2$ group, the single 7-NBn$_2$ group in **RPdPh$_2$** (**N,N-dibenzyl-7-aminoNPhosPdPh$_2$**) leads to a structures with an 87.36° C-Pd-C bond angle substantially larger than for the N$_2$Phos intermediate **APdPh2**. Two higher-energy structures calculated for **RPdPh$_2$** are 0.46 and 10.04 kcal/mol above the lowest energy form found and have similar C-Pd-C angles (85.60° and 85.48°, respectively). A third form is 22.78 kcal/mol higher with a C-Pd-C angle of 161.67°. This suggests that NBn$_2$ groups in both the 2- and 7-positions are needed to maximally compress the C-Pd-C angle in N$_2$Phos.

Other, rather different, substitutions in **S**, **T**, **U,** and **V** in Figure S3 lead to C-Pd-C angles of 82.23, 83.63, 82.64, and 83.28°, almost as low as for N$_2$Phos. For **S** and **T**, high-energy (by 16-25 kcal/mole) near-160° forms were also found. To test the effect of aryl groups larger than phenyl for the coupling partners, a structure was calculated for N$_2$Phos ligated to 2-naphthyl, o-tolylpalladium. In this case the C-Pd-C angle was 82.87°, only slightly larger than for diphenylpalladium.

Experimental data reflecting upon the rates of reaction that might be interpreted in the light of the compoutational results are discussed briefly here. Coupling reactions of aryl bromides generally occur smoothly and rapidly with a number of ligands studied at low catalyst concentrations. Some rather qualitative 'rate' data on this point are shown in Figure S6. In these two cases, the conversions appear to be slightly faster for N$_2$Phos

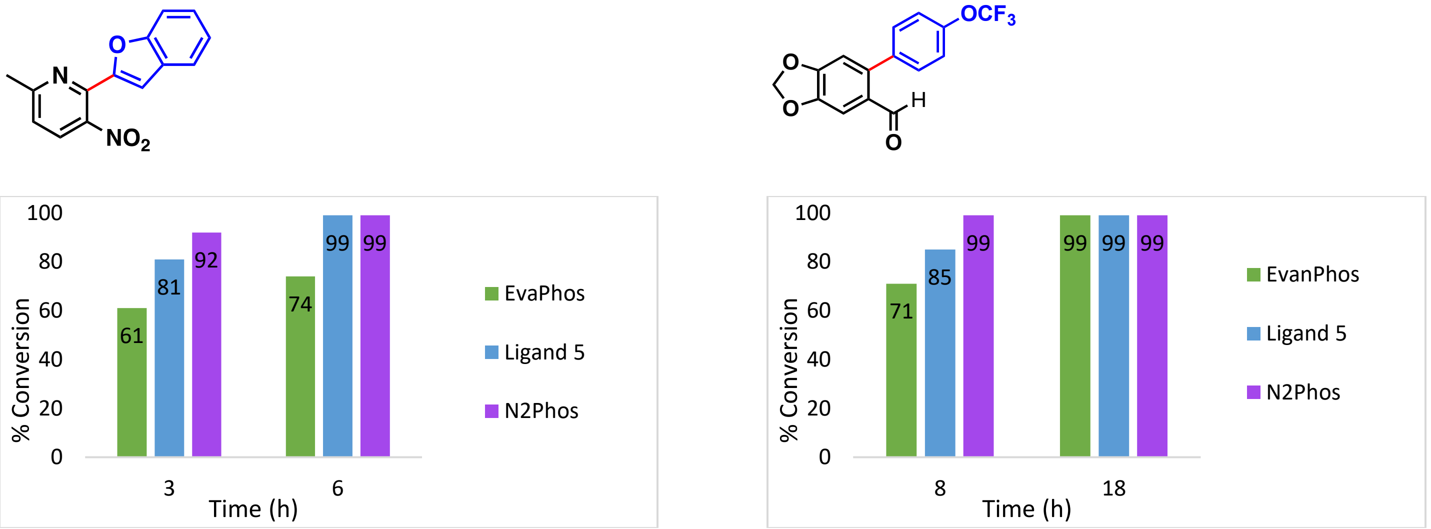

Figure S6. Relative Rates of Conversion between ligands [EvanPhos, Ligand **5** (NPhos with a 2-*N,N*-dimethylamino substituent in main text), and N$_2$Phos]. Conditoins: Pd (0.25 mol %) ligand (0.5 mol %) Ar-Br (0.5 mmol) Ar-B(OH)$_2$ (0.75 mmol) K_3PO_4 (0.75 mol %) 2 wt % TPGS-750-M/H_2O with toleuene 10% co-solvent), 45 °C.

than for EvanPhos or NPhos with a single 2-N,N-dimethylamino substituent. These data would be consistent with the C-Pd-C bond angle data in the pre-reductive intermediate structure if that were the rate-determining step, but again the effect appears to be small. For aryl chlorides, the conversions for are much slower than for aryl bromides, requiring higher catatyst concentrations. Furthermore, the difference in conversion is much more pronounced when changing from the EvanPhos to the N$_2$Phos ligand. For compound **20** for the coupling of p-

chloroanisole and p-tolylboronic acid in the main text**,** the reaction is 88% complete with N_2Phos after 8 h, while only 7% with EvanPhos over the same 8 h reaction time. After 16 h, N_2Phos went to 96% while EvanPhos went to approximately 9%. The concentration of catalyst and surfactant were identical for both examples (0.25 mol% Pd, and 0.45 mol % ligand and 2 wt % TPGS-750-M/H2O). For the aryl chlorides showing low conversions with EvanPhos, the final reaction mixtures showed substantial amounts of unreacted aryl chlorides and often protodeborylation of the boronic acid. In the case of aryl chlorides, the C-Pd-C bond angle data does not provide a very good explanation for why N_2Phos gives better conversions, since rate difference in the reductive elimination steps should be the same for aryl chlorides or bromides. A possibly better explanation for the increased efficacy of N_2Phos might lie in the considering that the palladium catalyst might be ligated to two ligands tying up a binding site on Pd needed for oxidative addition to the aryl chloride.

Thus, we tested whether two phosphine ligands might fit around a palladium using the prereductive elimination intermediates as models. When we tried to find an energy minimum for such a structure with two N_2Phos ligands **(N_2Phos)PdPh$_2$** at the B3LYP/6-31G(d)-SDD(Pd) level, one ligand separated from the palladium with a Pd-P distance of 6.3 Å before the geometry optimization was terminated unconverged, indicating that a structure with two ligands is too sterically hindered to stay bonded. The electronic energy of this separated-ligand structure was about 12 kcal/mol higher in energy than if the ligand were fully dissociated. For the 2-substituted ligand **Q** and diphenyl palladium, a 2:1 complex **Q$_2$PdPh$_2$**, was found as an energy minimum with Pd-P distances of 2.547 and 2.625 Å. A more stable minimum was found, however, for a structure **Q--QPdPh2** with one ligand only loosely bound with one Pd-P distance at 6.396 Å. This structure was lower in electronic energy by 5.98 kcal/mole and in free energy by 8.99 kcal/mole. Incidentally, a remarkably larger difference in the free energy (17.45 kcal/mol!) was calculated when the distorted harmonic energy approximation was used instead of the preferred quasiharmonic approximation.[11] A second conformation of the loose complex **Q--QPdPh2** was also found that was to be higher energy than the first loose complex by 3.81 kcal/mole in electronic energy and by 3.96 kcal/mole in free energy. When as much less hindered ligand **X** with no dibenzylamino substituents was tested, we found that the 2:1 complex **X$_2$PdPh$_2$** existed as a minimum with Pd-P distances of 2.557 and 2.621 Å. These model studies clearly show that N_2Phos is not be able to form a 2:1 complex to diphenylpalladium and formation of such a complex with the less hindered ligand **Q** would be substantially uphill in free energy. The much less hindered EvanPhos, on the other hand, is known to favor a 2:1complex with palladium dichloride based upon our X-ray crystal structure and computational work.[10] On the basis of this analysis, we postulated that the formation of an unreactive 2:1 complex between EvanPhos and other ligands much less sterically hindered than N_2Phos may well be the reason that N_2Phos is so effective as a catalyst. For EvanPhos, we think that tying the Pd up in a less reactive 2:1 complex slows the oxidative addition addition reaction with aryl chlorides to the point that irreversible protodeborylation of the boronic acid becomes faster and thereby interferes with the desired Suzuki-Miyaura cross coupling.

We tested this idea with direct calculations on the thermochemistry of the oxidative addition steps of the reactions palladium-zero complexes with chlorobenzene employing both ligands. Similar experimental and computational studies on phosphines as sterically hindered as tri-t-butyl phosphine, which is capable of Pd oxidative addition with aryl chlorides, have previously been carried out also suggesting the importance of steric effects to promote formation of the more reactive monoligated palladium-zero intermediates.[13-15] The species involved are shown in a kinetic Scheme S1 along with our computed free energy changes with the two ligands, EvanPhos and N_2Phos (energies in parentheses) from Tables S3 to S9. An artificial reduction step with lithium metal was included to complete the diagram back to palladium acetate. The free energies come from gas-phase single-point values at the M06D3/6-31+G(d,p)-SDD(Pd) level with D3 dispersion corrections, which can have have a large effect on the ligand association energies.[12] The effect of toluene solvation from the SMD continuum model is included in the tables of energies, but the effects are generally not large and are omitted in Scheme 1.

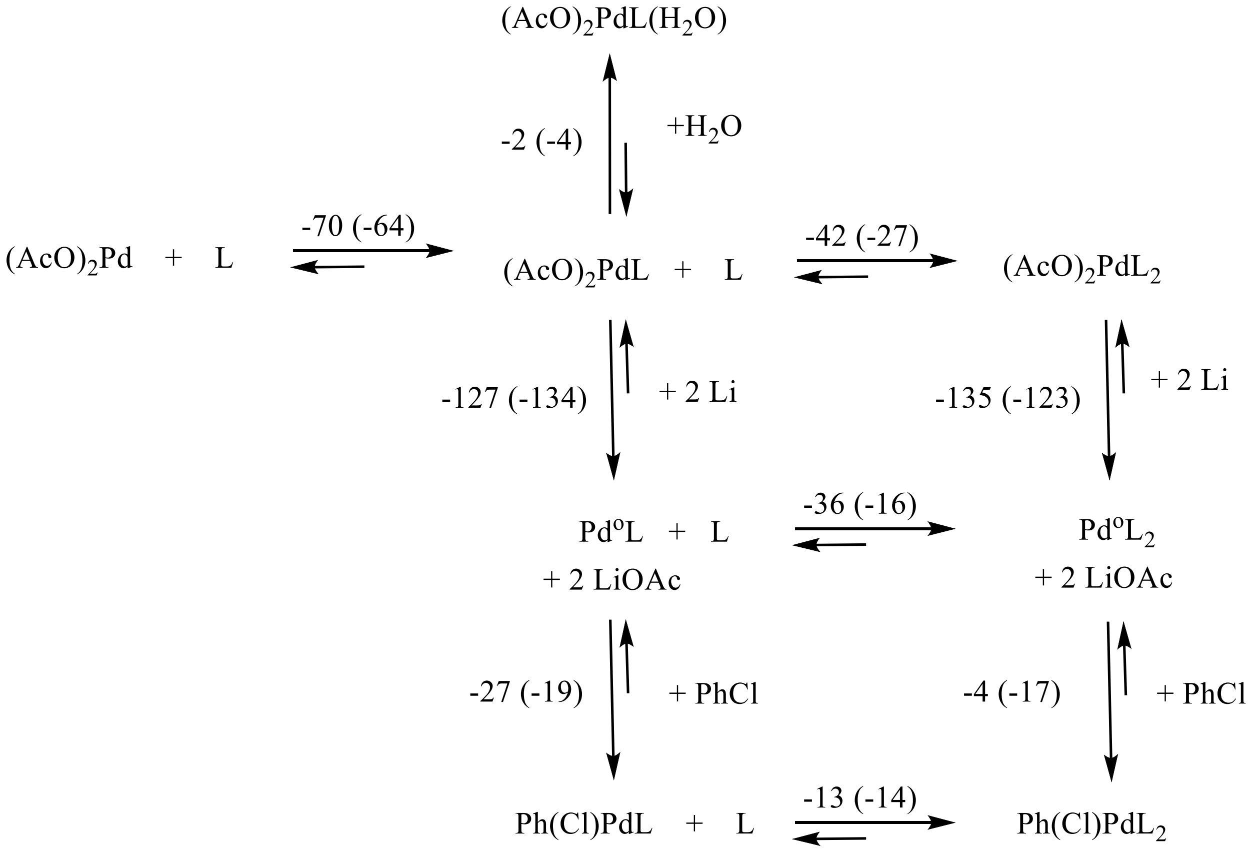

Scheme S1. Reaction scheme for oxidative addition steps of the Suzuki-Miyaura reaction with free energies at 298K in kcal/mol with EvanPhos (and N_2Phos energies in parentheses) ligands. Structures were optimized at the B3LYP/6-31G(d)/SMD level with reported single-point energies at the M06D3/6-31+G(d,p)-SDD(Pd) level of theory.

Palladium acetate is very strongly bound to its first ligand. Even with the sterically hindered N_2Phos ligand, all the palladium species in this scheme are also strongly stabilized by binding to a second ligand, though considerably less so than for EvanPhos. In looking at the structures of the monoligated species in Figures S2-5,S7-S12, one sees that methoxyl groups coordinate weakly with the open binding site of the T-shaped palladium center. Even in the diligated structures in Figures S9 and S10, there is evidence of very weak chelation by the methoxyl groups. Such binding, however, is seen seen to be weaker than for phosphorus ligands from the binding energy of water to $(AcO)_2PdL$, though the unfavorable 11 kcal/mol entropy term for this reaction is likely overstated in the free energy in solution because of water-water hydrogen bonding. We noticed also that the T-shaped structures of the oxidative addition products, Ph(Cl)PdL, preferred a configuration with the chloride ligand trans to the phosphine by 15 kcal/mol for either P-ligand. We, furthermore, chose to use the more stable (by 8-15 kcal/mol) trans-$(AcO)_2PdL_2$ complex in the scheme, but we used the less stable cis-Ph(Cl)PdL_2 product with significant steric repulsion between the bulky ligands because that is known to be the initially-formed oxidative addition product experimentally.[16] The conformations for the N_2Phos were searched exstensively, but not necessarily completely for the lowest energy form. The form chosen for our energy analysis in Scheme 1 was the form corresponding to the X-ray structure, which was 0.60 kcal/mol higher in energy than the best form, Table S2. Similarly, that same conformation for the N_2Phos ligand was used for LPd^o, though another conformer was found, again, to be 0.58 kcal/mol lower in energy. For the diligated N_2Phos species were chosen to correspond to have low-energy conformations based upon smaller molecules. Since the quantitative analysis below does not depend heavily upon having the exactly lowest energy conformer and conformational searches in such large species are impractical, we did not explore this further.

The observation of unreacted aryl chlorides and protodeborylation products in our experiments and the likelihood that oxidative addition is the overall rate-limiting step[13] indicate that the oxidative addition step is the key to our analysis of why aryl chlorides are better accommodated by the N_2Phos ligand. This reaction of palladium-zero intermediates, LPd^o and L_2Pd^o and chlorobenzene to form PhPd(Cl)L and PhPd(Cl)L_2, was studied with the EvanPhos and N_2Phos ligands. We found that the oxidative addition was much more downhill in free energy when carried out with the monoligated intermediates LPd^o in accord with expectations from the literature.[13,14] The M06D3/6-31+G(d,p)-SDD(Pd) free energies for the oxidative addition are 8 kcal/mol more negative for EvanPhos than for the N_2Phos ligand with the monoligated species LPd^o. This seems to contradict our presumption that N_2Phos reaction would be favored, though it would make sense based on the anticipated greater steric repulsion in the cis Ph(Cl)PdL_2 product with N_2Phos. The equilibria for further ligation of LPd^o to form L_2Pd^o have free energies of reaction of -36 and -16 kcal/mol for the EvanPhos and N_2Phos ligands, respectively. Thus, the diligated L_2Pd^o is the predominant form, and the overall reaction to form PhPd(Cl)L from L_2Pd^o will be downhill by 3 kcal/mol for N_2Phos and uphill by 9 kcal/mol for EvanPhos, leading to a

strong 12 kcal/mol overall preference for the N_2Phos reaction. With calcualtions in a toluene continuum, these crucial energies did not change much (see data Tables). The diligated L_2Pd^o is still the predominant form and the overall reaction to form PhPd(Cl)L from L_2Pd^o will be downhill by 6 kcal/mol for N_2Phos and uphill by 6 kcal/mol for EvanPhos, leading to the same strong 12 kcal/mol overall preference for the N_2Phos reaction. Therefore, we would expect that the difference in thermochemistry would make the reaction with N2Phos significantly faster than with EvanPhos, where a larger proportion of the catalyst remains in the lower energy diligated 'resting state', L_2Pd^o.

Under our experimental conditions, only 1.8 equivalents of ligand were added to the mixture, 10% short of the full 2 equivaents to convert all the palldium acetate into L_2Pd^o. One might ask whether that would reduce the advantage that N_2Phos would enjoy in competition with EvanPhos. Perhaps so, but it is possible that the ratios for such small quantities are not exact and/or that some of the less stable LPd^o form of Pd might be converted to palladium metal and precipitate out of solution leaving an excess of ligand if ligand oxidation is minimal.

Electron-donating effects of the dibenzylamino nitrogens were considered as a possible avenue to influence the electron density in the vicinity of the phosphine group and catalyst reactivity. Natural population analysis calculations on the free ligands and diphenyl palladium complexes for EvanPhos, N_2Phos, and an N_2Phos with the nitrogens replaced with CH groups at several levels of theory showed little or no regular variation of charge densities at phosphorus or palladium. The fact that the dihedral angles between the planes of the biaryl groups in the ligands is near 70° for these ligands and palladium complexes would certainly be expected to seriously diminish any putative pi-donating electronic effect. This suggests that the efficacy of N_2Phos is likely the result of steric effects, rather than any significant electronic effects.

Acknowledgments. Use was made of computational facilities purchased with funds from the National Science Foundation (CNS-1725797) and administered by the Center for Scientific Computing (CSC). The CSC is supported by the California NanoSystems Institute and the Materials Research Science and Engineering Center (MRSEC; NSF DMR 1720256) at UC Santa Barbara. The software used for 3-D drawings was from CYLview, 1.0b; Legault, C. Y., Université de Sherbrooke, 2009 (http://www.cylview.org).

References:

[1] A. D. Becke, *J. Chem. Phys.* **1993**, *98*, 5648. S. Grimme, S. Ehrlich, L. Goerigk, *J. Comp. Chem.* **2011**, *32*, 1456-1465.

[2] Y. Zhao; D. G. Truhlar, *Theor. Chem. Acc.* **2008**, *120*, 215-241.

[3] H. S. Yu, X. He, S. L. Li, D. G. Truhlar, *Chem. Sci.* **2016**, *7*, 5032.

[4] P. J. Hay, W. R. Wadt, *J. Chem. Phys.* **1985**, *82*, 299.

[5] From EMSL Library of Basis Set Exchange: K. L. Schuchardt, B. T. Didier, T. Elsethagen, L. Sun, V. Gurumoorthi, J. Chase, J.Li, T. L. Windus, *J. Chem. Inf. Model*. **2007**, *47*, 1045-1052.

[6] (a) A. P. Scott, L. Radom *J. Phys. Chem*. **1996**, *100*, 16502. (b) I. M. Alecu, J. Zheng, Y. Zhao, D. G. Truhlar,. *J. Chem. Theory Comput.* 2010**, 6**, 2872–2887. (c) R. F. Ribeiro, A. V. Marenich, C. J. Cramer, D. G. Truhlar *J. Phys. Chem. A* 2011, **115**, 14556-14562. (d) S. Grimme *Chem. Eur. J.* **2012**, *18*, 9955-9964. (e) D. H. Aue, to be published. We have evaluated optimum scale factors for experimental fundamental frequencies for numerous organic molecules and find that the DFT scale factors differ with basis set between 0.96 for the B3LYP/6-31G(d) and 0.954 for M06-2X/ 6-311+G(d,p) levels to 0.967 for the B3LYP/ 6-311G(d,p) level. For thermal terms and entropies, such variation has little effect, and one may argue that a scaling factor close to 1.00 might be best for the dominant low frequencies (we get 1.005 for 111 frequencies below 150 cm^{-1})(see also ref. 6a). For zero point energies determined from experimental values and CCSD(T)-F12/cc-aug-pVDZ values, the scale factors 1.00 (B3LYP/6-311G(d,p)), 0.984 (M06-2X/6-311+G(d,p)), 0.991 (M06/6-31G(d)), 0.996 (M06/6-31+G(d,p)) give the best fit for large variety of organic molecules.

[7] A. V. Marenich, C. J. Cramer, and D. G. Truhlar, *J. Phys. Chem. B* **2009**, *113*, 6378-96.

[8] E. D. Slack, R. Seupel, D. H. Aue, G. Bringmann; G.; B. H. Lipshutz, *Chem. Eur. J*. **2019**, *25*, 14237-14245.

[9] Gaussian 16, Revision A.03, M. J. Frisch, G. W. Trucks, H. B. Schlegel, G. E. Scuseria, M. A. Robb, J. R. Cheeseman, G. Scalmani, V. Barone, G. A. Petersson, H. Nakatsuji, X. Li, M. Caricato, A. V. Marenich, J. Bloino, B. G. Janesko, R. Gomperts, B. Mennucci, H. P. Hratchian, J. V. Ortiz, A. F. Izmaylov, J. L. Sonnenberg, D. Williams-Young, F. Ding, F. Lipparini, F. Egidi, J. Goings, B. Peng, A. Petrone, T. Henderson, D. Ranasinghe, V. G. Zakrzewski, J. Gao, N. Rega, G. Zheng, W. Liang, M. Hada, M. Ehara, K. Toyota, R. Fukuda, J. Hasegawa, M. Ishida, T. Nakajima, Y. Honda, O. Kitao, H. Nakai, T. Vreven, K. Throssell, J. A. Montgomery, Jr., J. E. Peralta, F. Ogliaro, M. J. Bearpark, J. J. Heyd, E. N. Brothers, K. N. Kudin, V. N. Staroverov, T. A. Keith, R. Kobayashi, J. Normand, K. Raghavachari, A. P. Rendell, J. C. Burant, S. S. Iyengar, J. Tomasi, M. Cossi, J. M. Millam, M. Klene, C. Adamo, R. Cammi, J. W. Ochterski, R. L. Martin, K. Morokuma, O. Farkas, J. B. Foresman, D. J. Fox, Gaussian, Inc., Wallingford CT, 2016.

[10] E. B. Landstrom, S. Handa, D. H. Aue, F. Gallou, B. H. Lipshutz, *Green Chem*. **2018,** *20*, 3436–3443.

[11] This is the largest such difference we have ever seen between the quasiharmonic and harmonic approxiamtions and emphasizes how important it is for workers in the field of computational chemistry to pay attention to this problem of low frequency distortions on entropy effects.[6c,d] Grimme[12] has found examples of supermolecular complexes where differences in free energies of 3-4 kcal/mole can occur. Clearly, we see that organometallic complexes with many low-frequency vibrational modes can show much larger discrepancies.

[12] S. Grimme, *Chem. Eur. J.* **2012**, *18,* 9955–9964.

[13] C. L. McMullin, N. F. and J. N. Harvey, Dalton Trans. **2014**, *43*, 13545-13556.

[14] Z. Li; Y. Fu; Q.-X. Guo; L. Liu, *Organometallics* **2008**, *27,* 4043-4049.

[15] K. Vikse, T. Naka, J. S. McIndoe, M. Besora, F. Maseras, Chem. Cat. Chem. **2013**, *5*, 3604-3609.

[16] A. L. Casado, P. Espinet, *Organometallics* **1998**, *17,* 954–959.

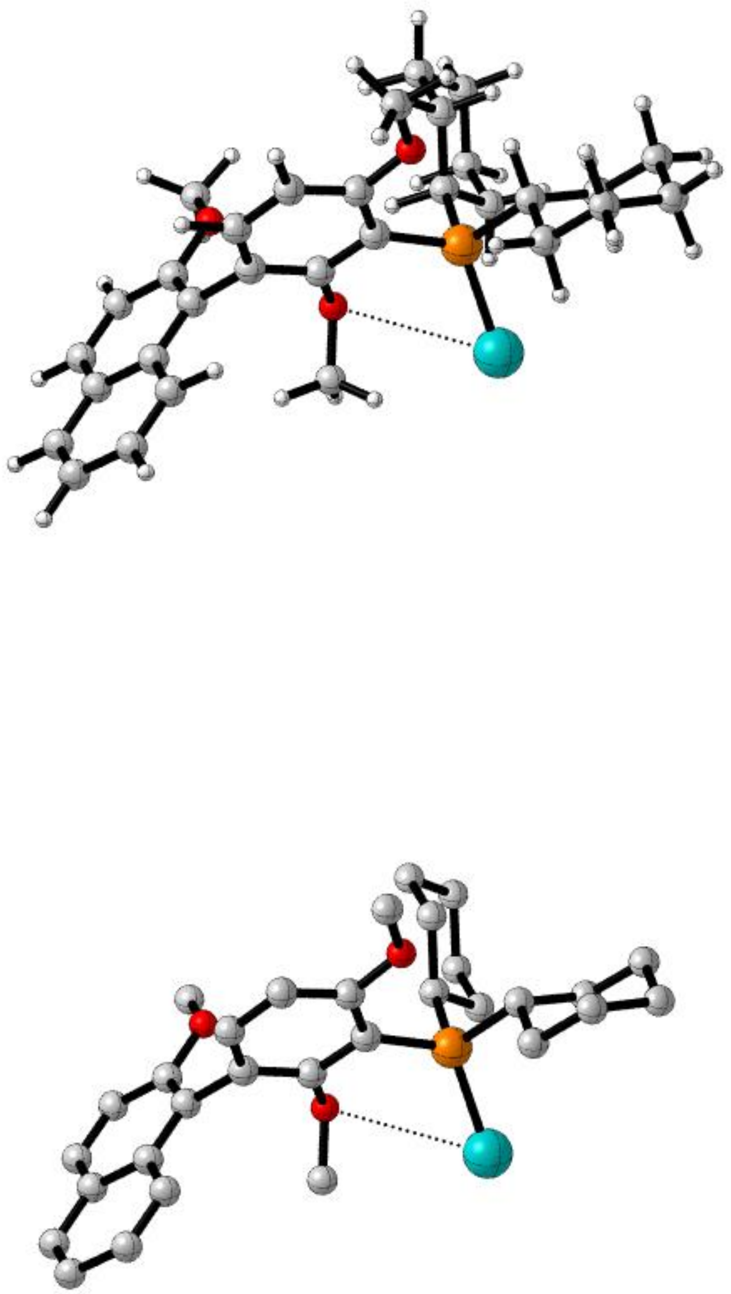

Figure S7. B3LYP/6-31G(d)(SDD) optimized structure for **Pd(EvanPhos)**. O-Pd distance=3.379 Å. Atom colors: nitrogen, blue; oxygen, red; palladium, teal.

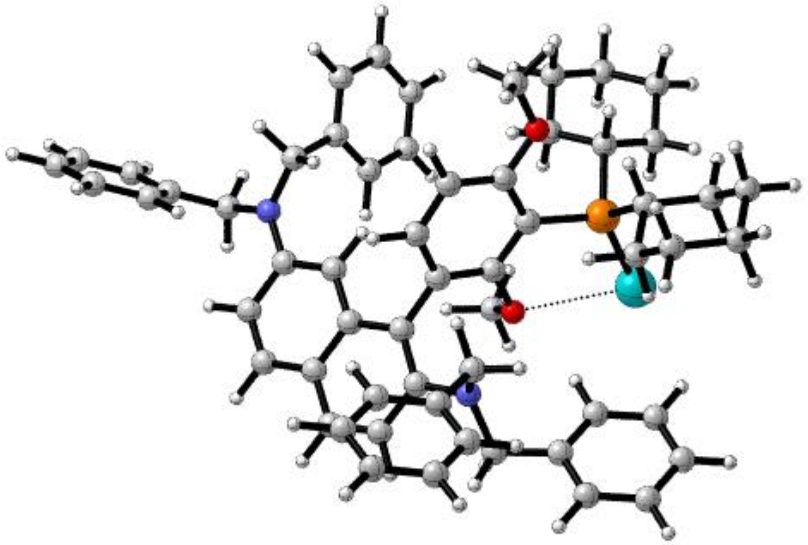

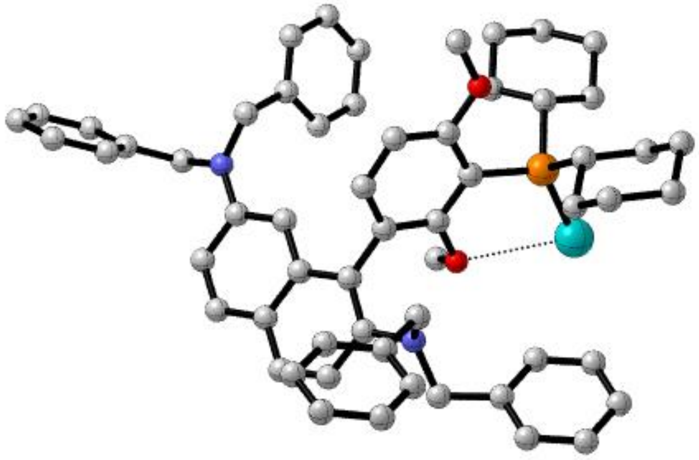

Figure S8. B3LYP/6-31G(d)(SDD) optimized structure for **Pd(N$_2$Phos)**. O-Pd distance=3.069 Å. Atom colors: nitrogen, blue; oxygen, red; palladium, teal.

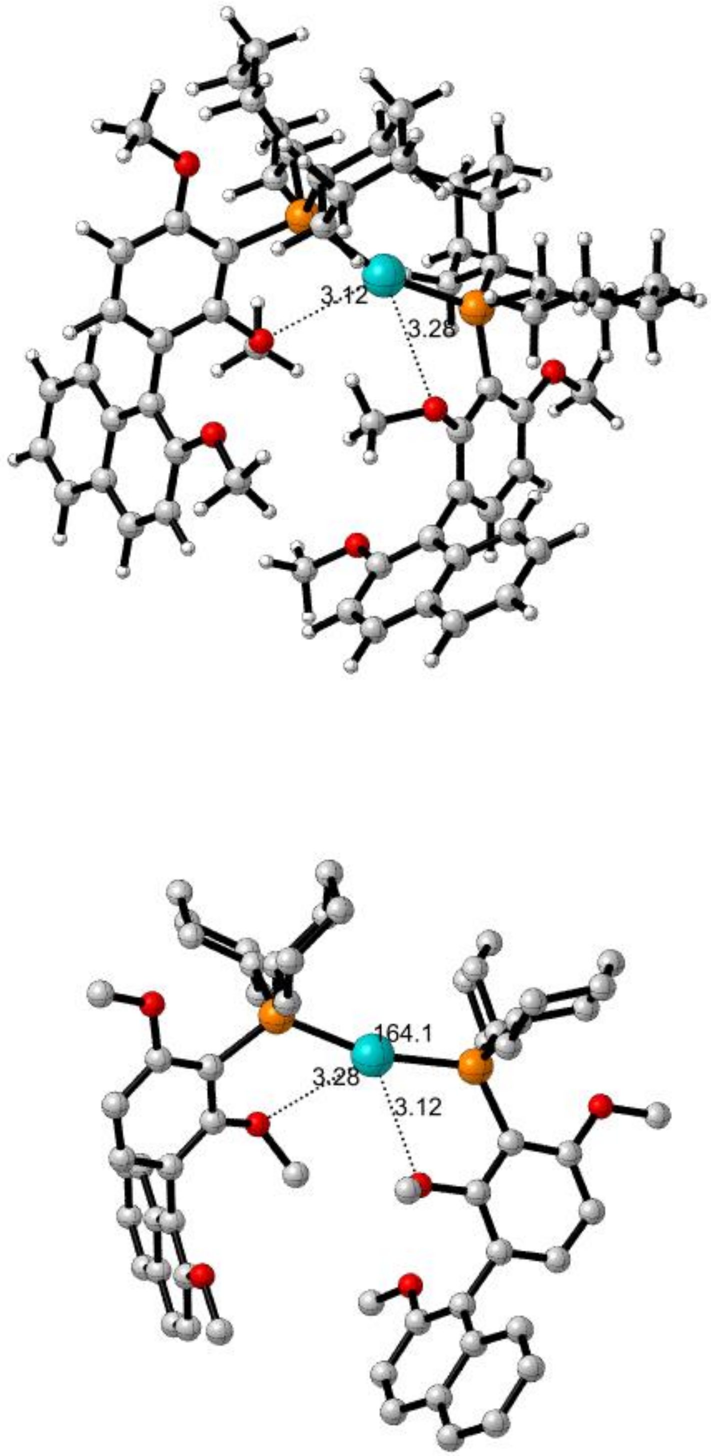

Figure S9. B3LYP/6-31G(d)(SDD) optimized structure for **Pd(EvanPhos)$_2$**. O-Pd distance=3.123, 3.277 Å, P-Pd-P angle=164.04^o. Atom colors: nitrogen, blue; oxygen, red; palladium, teal.

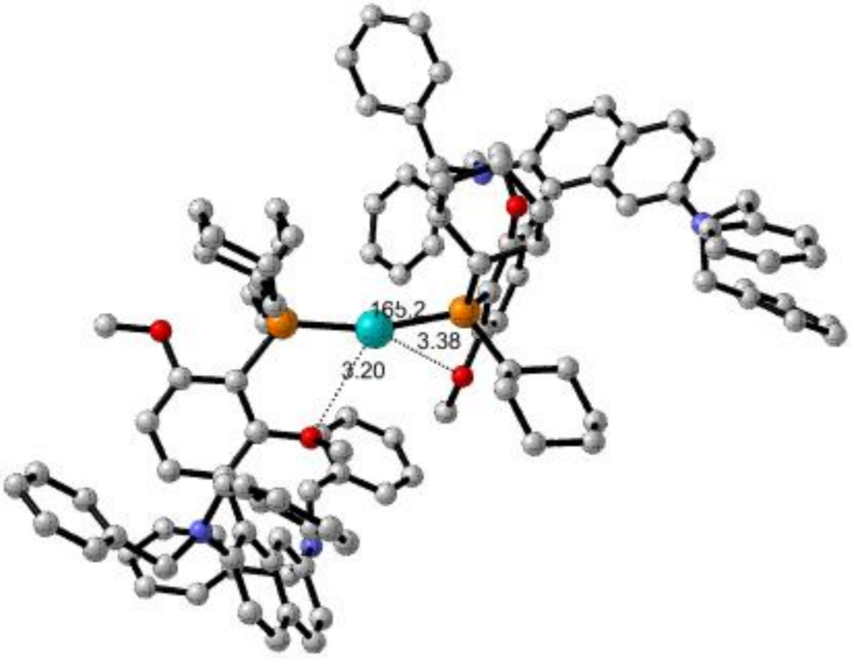

Figure S10. B3LYP/6-31G(d)(SDD) optimized structure for **Pd(N$_2$Phos)$_2$**. O-Pd distances=3.195, 3.379 Å, P-Pd-P angle=165.18^o. Atom colors: nitrogen, blue; oxygen, red; palladium, teal.

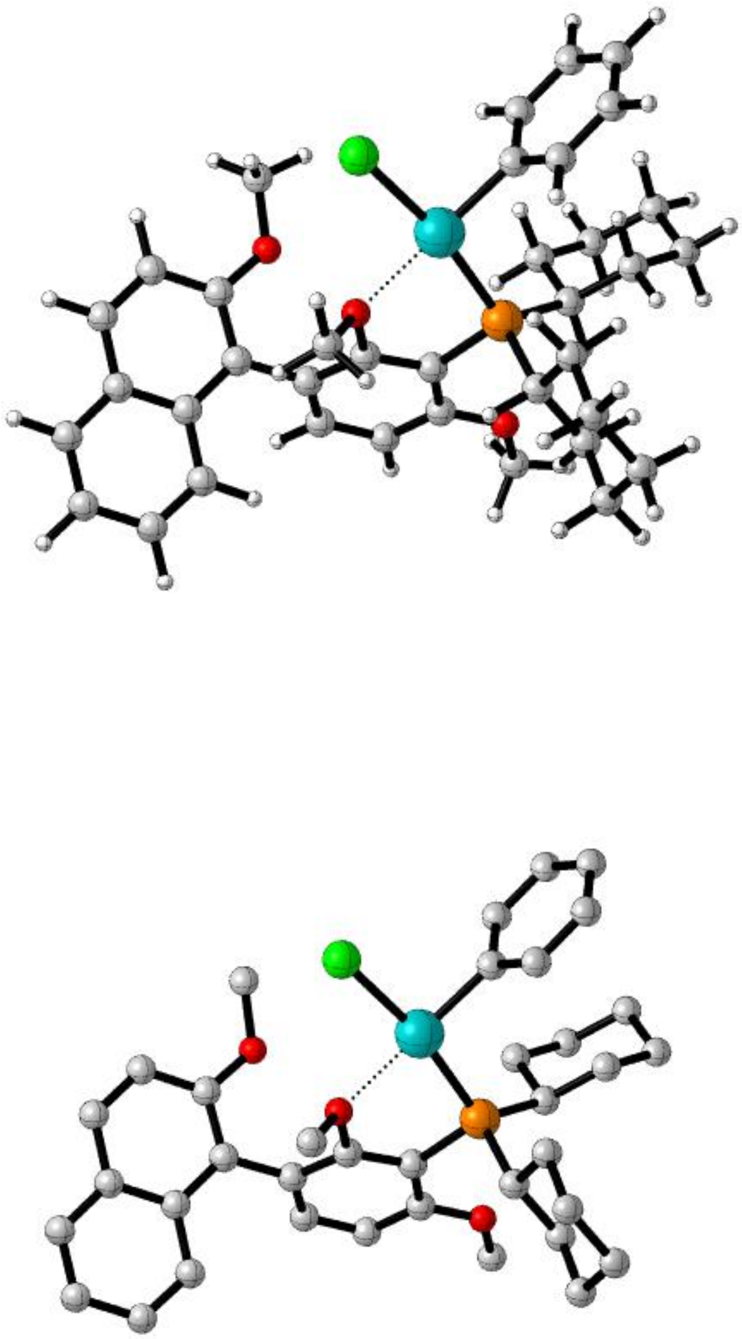

Figure S11. B3LYP/6-31G(d)(SDD) optimized structure for **PhPd(Cl)(EvanPhos)**. O-Pd distance=2.273 Å. Atom colors: nitrogen, blue; oxygen, red; palladium, teal.

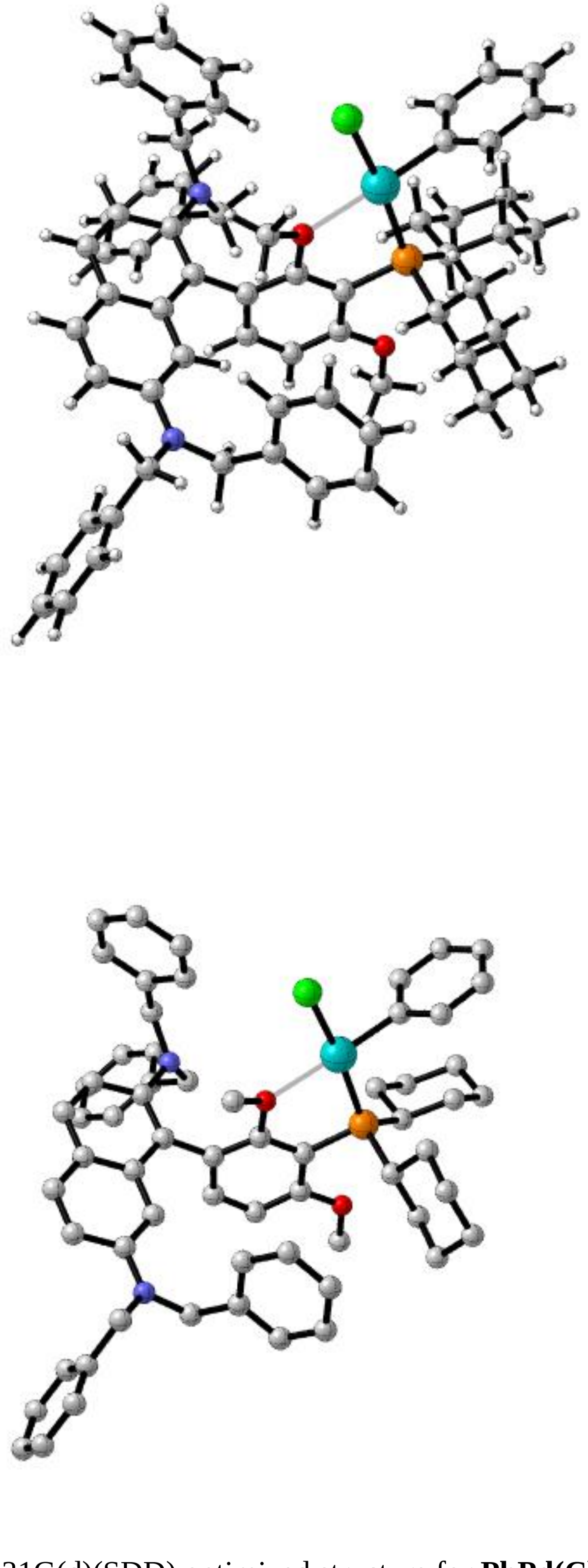

Figure S12. B3LYP/6-31G(d)(SDD) optimized structure for **PhPd(Cl)(N_2Phos)**. O-Pd distance=2.321 Å. Atom colors: nitrogen, blue; oxygen, red; palladium, teal.

Table S3. Calculated electronic energies, enthalpies, and free energies at 298 K for the oxidative addition steps of chlorobenzene with EvanPhos or N_2Phos ligands optimized at the B3LYP/6-31G(d)/SMD level and with single-point energies at the M06D3/6-31+G(d,p)-SDD(Pd) level of theory, with solvation free energies from the SMD continuum model in toluene included in parentheses.

Molecule:	B3LYP			M06D3
	$\Delta E^o e$	ΔH^o_{298K}	ΔG^o_{298K}	ΔG^o_{298K}
With 1: 1 catalyst:				
Pd^o(EvanPhos) + PhCl → PhPdCl(EvanPhos)	-41.48	-37.78	-23.55	-26.82 (-28.49)
PhPdCl(N_2Phos)	--34.47	-30.69	-16.07	-18.66 (-19.74)
Reaction energy difference: N_2Phos vs. EvanPhos	7.00	7.09	7.48	8.17 (8.55)
With 2: 1 catalyst:				
$Pd^o(EvanPhos)_2$ + PhCl → $PhPdCl(EvanPhos)_2$	1.40	5.87	20.93	-3.80 (-1.77)
$Pd^o(N_2Phos)_2$ + PhCl → $PhPdCl(N_2Phos)_2$	-0.79	3.66	18.94	-17.39 (-12.93)
Reaction energy difference: N_2Phos vs. EvanPhos	-2.19	-2.21	-1.98	-13.59 (-11.16)

Table S4. Calculated electronic energies, enthalpies, and free energies at 298 K for the combined reduction and oxidative addition steps of chlorobenzene with EvanPhos or N_2Phos ligands optimized at the B3LYP/6-31G(d)/SMD level and with single-point energies at the M06D3/6-31+G(d,p)-SDD(Pd) level of theory.

Molecule:	B3LYP			M06D3
	$\Delta E^o e$	ΔH^o_{298K}	ΔG^o_{298K}	ΔG^o_{298K}
$(OAc)_2$Pd(EvanPhos) + 2 Li + PhCl → PhPdCl(EvanPhos) + 2 LiOAc	-144.65	-142.95	-139.61	-154.21
$(OAc)_2$Pd(N_2Phos) + 2 Li +PhCl → PhPdCl(N_2Phos) + 2 LiOAc	-143.91	-142.24	-138.87	-152.69
Reaction energy difference: N_2Phos vs. EvanPhos	0.75	0.71	0.74	1.52
$(OAc)_2$Pd$(EvanPhos)_2$ + 2Li + PhCl → cis- PhPdCl$(EvanPhos)_2$ + 2 LiOAc	-119.32	-117.92	-114.57	-125.65
$(OAc)_2$Pd$(N_2Phos)_2$ + 2 Li + PhCl → cis- PhPdCl$(N_2Phos)_2$ + 2 LiOAc	-122.92	-121.13	-117.25	-139.60
Reaction energy difference: N_2Phos vs. EvanPhos	-3.60	-3.21	-2.68	-13.94

Table S5. Calculated electronic energies, enthalpies, and free energies at 298 K for attachment of a second ligand (L), EvanPhos or N_2Phos, to $(AcO)_2PdL$ or $(AcO)_2PdL(H_2O)$ optimized at the B3LYP/6-31G(d)-SDD level and with single-point energies at the M06D3/6-31+G(d,p)-SDD(Pd) level of theory, with solvation free energies from the SMD continuum model in toluene included in parentheses.

Molecule:	B3LYP			M06D3
	$\Delta E^o e$	ΔH^o_{298K}	ΔG^o_{298K}	ΔG^o_{298K}
$(AcO)_2$Pd(EvanPhos) + L → trans$(AcO)_2$Pd$(EvanPhos)_2$	-13.55	-11.21	6.63	-42.02 (-34.47)
$(AcO)_2$Pd(N_2Phos) + L → trans-$(AcO)_2$Pd$(N_2Phos)_2$	-1.96	-0.22	18.50	-27.49 (-18.57)
Complexation energy difference: N_2Phos vs. EvanPhos	11.59	10.99	11.87	14.53 (15.89)
$(AcO)_2$Pd(EvanPhos)(H_2O) + L → trans-$(AcO)_2$Pd$(EvanPhos)_2$ + H_2O	1.85	3.40	10.11	-39.69 (-34.44)
$(AcO)_2$Pd(N_2Phos)(H_2O) + L → trans-$(AcO)_2$Pd$(N_2Phos)_2$ + H_2O	16.42	17.50	25.14	-23.29 (-18.32)
Complexation energy difference: N_2Phos vs. EvanPhos	14.57	14.10	15.03	16.39 (17.11)

Table S6. Calculated electronic energies, enthalpies, and free energies at 298 K for geometrical isomerization of cis-$(AcO)_2PdL_2$ to trans-$(AcO)_2PdL_2$ with ligands (L), EvanPhos or N_2Phos, and Cl and P ligands cis- to trans in PhPdClL optimized at the B3LYP/6-31G(d)-SDD level and with single-point energies at the M06D3/6-31+G(d,p)-SDD(Pd) level of theory, with solvation free energies from the SMD continuum model in toluene included in parentheses.

Molecule:	B3LYP			M06D3
	$\Delta E^{o}e$	ΔH^{o}_{298K}	ΔG^{o}_{298K}	ΔG^{o}_{298K}
cis-$(AcO)_2$Pd$(EvanPhos)_2$ → trans$(AcO)_2$Pd$(EvanPhos)_2$	-14.90	-14.88	-15.09	-15.39 (-13.59)
cis-$(AcO)_2$Pd$(N_2Phos)_2$ → trans-$(AcO)_2$Pd$(N_2Phos)_2$	-15.17	-15.68	-16.63	-8.82 (-8.66)
Complexation energy difference: N_2Phos vs. EvanPhos	-0.27	-0.81	-1.61	6.57 (4.92)
cis-PhPdCl(EvanPhos) → trans-PhPdCl(EvanPhos)	-12.08	-11.80	-11.79	-15.12 (-14.96)
cis-PhPdCl(N2Phos) → trans-PhPdCl(N2Phos)	-14.41	-14.00	-13.88	-8.17 (-14.24)
Complexation energy difference: N2Phos vs. EvanPhos	-2.33	-2.20	-2.09	0.94 (0.72)

Table S7. Calculated electronic energies, enthalpies, and free energies at 298 K for attachment of a second ligand (L), EvanPhos or N_2Phos, to Cl_2PdL or Cl_2PdL(H_2O) optimized at the B3LYP/6-31G(d)-SDD level and with single-point energies at the M06D3/6-31+G(d,p)-SDD(Pd) level of theory.

Molecule:	B3LYP			M06D3
	$\Delta E^{o}e$	ΔH^{o}_{298K}	ΔG^{o}_{298K}	ΔG^{o}_{298K}
Cl_2Pd(EvanPhos) + L → trans-Cl_2Pd$(EvanPhos)_2$	-21.57	-19.4	-2.03	-15.58
Cl_2Pd(N_2Phos) + L → trans-Cl_2Pd$(N_2Phos)_2$	-11.02	-9.18	9.32	-28.22
Complexation energy difference: N_2Phos vs. EvanPhos	10.55	10.22	11.35	-12.64
Cl_2Pd(EvanPhos) + H_2O → Trans-Cl_2Pd$(EvanPhos)_2$ + H_2O	-4.54	-4.19	3.44	-9.75

Table S8. Calculated electronic energies, enthalpies, and free energies at 298 K for attachment of a second ligand (L), EvanPhos or N_2Phos, to cis-PhPdCl(L) or cis-PhPdCl(L)(H_2O) with release of water optimized at the B3LYP/6-31G(d)-SDD level and with single-point energies at the M06D3/6-31+G(d,p)-SDD(Pd) level of theory, with solvation free energies from the SMD continuum model in toluene included in parentheses.

Molecule:	B3LYP			M06D3
	$\Delta E^o e$	ΔH^o_{298K}	ΔG^o_{298K}	ΔG^o_{298K}
PhPdCl(EvanPhos) + L → cis- PhPdCl(EvanPhos)$_2$	11.79	13.82	31.66	-13.46 (-7.67)
PhPdCl(N_2Phos) + L → cis- PhPdCl(N_2Phos)$_2$	19.03	20.88	40.12	-14.40 (-6.70)
Complexation energy difference: N_2Phos vs. EvanPhos	7.25	7.06	8.46	-0.94 (0.97)
PhPdCl(EvanPhos)(H_2O) + L → cis-PhPdCl(EvanPhos)$_2$ + H_2O	5.53	6.12	13.66	-33.29
PhPdCl(N_2Phos)$_2$(H_2O) + L → cis- PhPdCl(N_2Phos)$_2$ + H_2O	27.34	27.72	36.8	-15.62
Complexation energy difference: N_2Phos vs. EvanPhos	21.81	21.60	23.13	17.67
PhPdOAc(EvanPhos) + L → cis-PhPdCl(EvanPhos)$_2$	4.36	6.81	25.03	-24.91
PhPdOAc(N_2Phos) + L → cis-PhPdOAc(N_2Phos)$_2$	26.81			
Complexation energy difference: N_2Phos vs. EvanPhos	22.45			
Cl_2Pd(EvanPhos) + L → cis-Cl_2Pd(EvanPhos)$_2$	2.9	5.03	22.8	-21.97
Cl_2Pd(N_2Phos) + L → cis-Cl_2Pd(N_2Phos)$_2$	5.6	7.81	26.91	-25.19
Complexation energy difference: N_2Phos vs. EvanPhos	2.70	2.78	4.11	-3.22

Table S9. Calculated electronic energies, enthalpies, and free energies at 298 K for attachment of a ligand(L) to EvanPhos or N_2Phos to $Pd(OAc)_2$, water to $(AcO)_2Pd(L)$, and L to Pd^o(EvanPhos) optimized at the B3LYP/6-31G(d)/SDD level and with single-point energies at the M06D3/6-31+G(d,p)-SDD(Pd) level of theory, with solvation free energies from the SMD continuum model in toluene included in parentheses.

Molecule:	B3LYP			M06D3
	$\Delta E^o e$	ΔH^o_{298K}	ΔG^o_{298K}	ΔG^o_{298K}
$(AcO)_2Pd$ + EvanPhos → $(AcO)_2Pd$(EvanPhos)	-78.78	-74.36	-59.28	-70.23 (-63.19)
$(AcO)_2Pd$ + N_2Phos → $(AcO)_2Pd(N_2Phos)$	-72.84	-68.39	-52.87	-64.35 (-57.98)
Complexation energy difference: N_2Phos vs. EvanPhos	5.94	5.97	6.41	5.88 (5.21)
$(AcO)_2Pd$(EvanPhos) + H_2O → $(AcO)_2Pd(EvanPhos)(H_2O)$	-15.40	-14.61	-3.48	-2.33 (0.97)
$(AcO)_2Pd(N_2Phos)$ + H_2O → $(AcO)_2Pd(N_2Phos)(H_2O)$	-18.38	-17.72	-6.64	-4.20 (-0.25)
Complexation energy difference: N_2Phos vs. EvanPhos	-2.98	-3.11	-3.16	-1.87 (-1.22)
Pd^o(EvanPhos) + EvanPhos → $Pd(EvanPhos)_2$	-31.09	-29.82	-12.82	-36.49 (-34.19)
$Pd^o(N_2Phos)$ + N_2Phos → $Pd(N_2Phos)_2$	-14.64	-13.46	5.10	-15.67 (-13.50)
Complexation energy difference: N_2Phos vs. EvanPhos	16.45	16.36	17.92	20.82 (20.69)

Table S10. Thermodynamic parameters and Cartesian coordinates from an X-ray structure and optimized structures at various levels of theory.

Conformer **A**: X-ray crystal structure:

C	-4.385022	-2.246773	0.474766
C	-4.189278	-1.730643	1.744038
C	-4.735198	-2.411401	2.809782
C	-5.476337	-3.567427	2.618240
C	-5.662001	-4.061298	1.363048
C	-5.117855	-3.403149	0.293166
C	-3.408561	-0.455560	1.983837
N	-2.442811	-0.129962	0.953397
C	-1.212563	-0.925344	0.979279
C	0.035305	-0.112267	0.809497
C	1.166804	-0.680843	0.242654
C	2.319570	0.060709	0.086731
C	2.380768	1.360290	0.495444
C	1.266061	1.926787	1.076162
C	0.108843	1.206246	1.226834
C	-2.859217	0.447482	-0.244827
C	-2.081254	0.288011	-1.417162
C	-2.480931	0.820130	-2.599568
C	-3.666691	1.580632	-2.713526
C	-4.448705	1.772753	-1.556857
C	-4.032646	1.180760	-0.338225
C	-5.653428	2.540494	-1.654190
C	-5.999676	3.119044	-2.865911
C	-5.210760	2.881629	-4.017005
C	-4.082898	2.122217	-3.937777
C	-6.512175	2.697775	-0.447645
C	-7.661471	1.925846	-0.268651
C	-8.441562	1.996068	0.884939
C	-8.050164	2.920031	1.873852
C	-6.935415	3.717711	1.714764
C	-6.185300	3.586065	0.558412
P	-9.899087	0.884931	1.051488
C	-9.635144	0.079003	2.703117
C	-8.183943	-0.344803	2.916293
C	-8.001013	-1.072728	4.242482
C	-8.887604	-2.300762	4.328044
C	-10.341410	-1.915869	4.102327
C	-10.524560	-1.166943	2.788126
O	-8.041522	1.078786	-1.291314
C	-7.496647	-0.240771	-1.179130
O	-8.826897	2.960798	2.999042
C	-8.516944	3.954218	3.980272
N	-7.134702	3.980052	-2.928672
C	-7.746403	4.113265	-4.245930
C	-9.132794	4.698324	-4.148534
C	-10.019445	4.239092	-3.198329

C	-11.294380	4.759888	-3.120651
C	-11.692477	5.755002	-3.992051
C	-10.819189	6.225380	-4.923253
C	-9.538010	5.701601	-5.010678
C	-6.903246	5.295399	-2.293487
C	-5.813410	6.119194	-2.897679
C	-4.488050	5.957527	-2.507925
C	-3.488854	6.721614	-3.061240
C	-3.787012	7.672622	-4.015239
C	-5.090733	7.841562	-4.423346
C	-6.090858	7.065583	-3.871564
C	-11.270379	2.120730	1.308849
C	-12.391705	1.624358	2.202663
C	-13.457783	2.709353	2.346979
C	-14.052215	3.040855	0.999609
C	-12.999669	3.494312	0.014173
C	-11.828046	2.521702	-0.064538
H	-10.874408	2.935841	1.732019
H	-12.030079	1.391994	3.095651
H	-12.795400	0.810060	1.811535
H	-14.172075	2.396351	2.957987
H	-13.052530	3.523120	2.739490
H	-14.728301	3.757576	1.107931
H	-14.513694	2.240868	0.640421
H	-12.662967	4.385335	0.284471
H	-13.408385	3.583559	-0.882266
H	-11.102125	2.936121	-0.596366
H	-12.121403	1.705290	-0.541636
H	-9.898750	0.719503	3.423864
H	-7.903185	-0.939260	2.175987
H	-7.603666	0.457052	2.901914
H	-7.054113	-1.345640	4.339510
H	-8.220865	-0.458958	4.987309
H	-8.789061	-2.720903	5.219717
H	-8.608748	-2.960718	3.644357
H	-10.648882	-1.344126	4.850619
H	-10.900698	-2.732865	4.093417
H	-11.472844	-0.895883	2.697047
H	-10.307922	-1.772371	2.035996
H	-5.412147	4.130187	0.452826
H	-6.687608	4.343614	2.385672
H	-6.523882	-0.203727	-1.298390
H	-7.887633	-0.816753	-1.867817
H	-7.702491	-0.604801	-0.292541
H	-7.610137	3.804323	4.321064
H	-9.157146	3.893847	4.720600
H	-8.569777	4.843996	3.574013
H	-5.472082	3.253742	-4.851203
H	-3.571767	1.958162	-4.721571
H	-1.949846	0.676598	-3.373230
H	-1.265948	-0.200059	-1.373875

H	-4.573188	1.288607	0.434544
H	-6.689321	5.147563	-1.337509
H	-7.745359	5.813183	-2.331922
H	-4.270019	5.310153	-1.848347
H	-2.588544	6.593108	-2.785411
H	-3.095331	8.207240	-4.387728
H	-5.303749	8.492032	-5.083074
H	-6.986028	7.181599	-4.166004
H	-7.794142	3.223222	-4.677164
H	-7.183001	4.697007	-4.812836
H	-9.751114	3.558370	-2.592591
H	-11.900982	4.434371	-2.465795
H	-12.572428	6.108974	-3.940851
H	-11.088671	6.917077	-5.516604
H	-8.934450	6.033077	-5.665507
H	-1.253177	-1.602411	0.257967
H	-1.162021	-1.408186	1.842097
H	1.145938	-1.587819	-0.040922
H	3.083794	-0.340667	-0.310914
H	3.176155	1.867148	0.383470
H	1.299741	2.827615	1.374041
H	-0.651758	1.615454	1.622734
H	-4.048077	0.295316	2.065133
H	-2.931454	-0.537272	2.848250
H	-4.600710	-2.083398	3.692158
H	-5.855606	-4.016686	3.364063
H	-6.165165	-4.856276	1.230801
H	-5.245480	-3.745697	-0.583835
H	-4.012755	-1.801211	-0.276398

Conformer **A**: B3LYP/6-31G(d) [5d]
Processing: namphos-h5dbe.log
PG=C01

Method	BasisSet	Imaginary Freqs
RB3LYP	6-31G(d)	0

HF Energy
-2849.3619586

ZPE	E298	S298	Squasihar	Equasihar	Strans	Srot
678.17688	714.264	336.475	285.949	714.471	46.099	40.619

Processing: namphos-h5dbe.log
126

C	3.894368	-2.272526	-1.227324
C	4.494421	-2.122183	0.029652
C	4.906855	-3.268955	0.719884
C	4.718111	-4.539803	0.174233
C	4.119045	-4.679868	-1.079663

C	3.707763	-3.541796	-1.777811
C	4.701181	-0.746251	0.654819
N	4.987120	0.322959	-0.290166
C	6.326092	0.299874	-0.858028
C	7.418049	0.956148	-0.016471
C	8.761393	0.662863	-0.290734
C	9.787216	1.264882	0.436753
C	9.483646	2.167509	1.459816
C	8.149811	2.460030	1.744839
C	7.123626	1.858623	1.010748
C	4.035623	1.273793	-0.666895
C	4.459748	2.473272	-1.322175
C	3.540683	3.409949	-1.720458
C	2.153418	3.253099	-1.473622
C	1.705130	2.086422	-0.771756
C	2.673886	1.105421	-0.415573
C	0.300470	1.921734	-0.496969
C	-0.610791	2.898629	-0.926682
C	-0.129741	4.042494	-1.629809
C	1.206152	4.213931	-1.891843
C	-0.143775	0.724556	0.283330
C	-0.924513	-0.312430	-0.274451
C	-1.357343	-1.426450	0.474182
C	-0.956728	-1.494788	1.830651
C	-0.167786	-0.492555	2.401926
C	0.221862	0.598584	1.627019
P	-2.474706	-2.664380	-0.370996
C	-1.661862	-4.351065	-0.011466
C	-0.120733	-4.313696	-0.022789
C	0.482020	-5.708324	0.221555
C	-0.026048	-6.739651	-0.795426
C	-1.560187	-6.773548	-0.826567
C	-2.148576	-5.372795	-1.066883
O	-1.309021	-0.194842	-1.592769
C	-0.391533	-0.729222	-2.544710
O	-1.374560	-2.591737	2.533564
C	-1.004044	-2.713603	3.897420
N	-2.008182	2.768139	-0.689778
C	-2.896047	3.397081	-1.673323
C	-4.250202	2.717767	-1.798984
C	-4.338946	1.341141	-2.051935
C	-5.582663	0.742090	-2.256337
C	-6.754127	1.504296	-2.204262
C	-6.674135	2.872779	-1.946247
C	-5.427705	3.472954	-1.746036
C	-2.487110	2.865965	0.703658
C	-2.555171	4.278367	1.269972
C	-1.390236	4.955135	1.663826
C	-1.454315	6.252295	2.173384
C	-2.687641	6.896646	2.302686
C	-3.854348	6.233763	1.919828

C	-3.785143	4.935668	1.407634
C	-3.952507	-2.685117	0.827684
C	-5.049993	-3.646371	0.322824
C	-6.291656	-3.632968	1.232734
C	-6.853687	-2.216187	1.409122
C	-5.768740	-1.258275	1.919426
C	-4.531143	-1.266145	1.006208
H	-3.601401	-3.040761	1.803573
H	-4.665336	-4.670836	0.260832
H	-5.344497	-3.359471	-0.697660
H	-7.059010	-4.302377	0.821658
H	-6.019758	-4.038598	2.218855
H	-7.710804	-2.227762	2.095245
H	-7.230524	-1.851929	0.441856
H	-5.472784	-1.556449	2.936708
H	-6.165484	-0.237527	1.995409
H	-3.767730	-0.595307	1.415836
H	-4.808093	-0.855510	0.025643
H	-1.984864	-4.685729	0.983929
H	0.231729	-3.937806	-0.995522
H	0.254190	-3.618011	0.732438
H	1.576927	-5.645712	0.190082
H	0.214424	-6.039849	1.236357
H	0.375222	-7.735602	-0.565414
H	0.348158	-6.474557	-1.795810
H	-1.934467	-7.164508	0.131536
H	-1.912253	-7.464763	-1.603848
H	-3.242632	-5.430412	-1.084678
H	-1.848314	-5.018684	-2.063879
H	0.824712	1.380820	2.080861
H	0.137898	-0.542662	3.440261
H	0.603498	-0.282412	-2.429068
H	-0.791752	-0.477835	-3.529523
H	-0.320906	-1.820672	-2.451241
H	0.086079	-2.762958	4.016997
H	-1.446868	-3.649511	4.243086
H	-1.396078	-1.883120	4.498769
H	-0.832025	4.812718	-1.926557
H	1.551762	5.105347	-2.411024
H	3.879079	4.312299	-2.225175
H	5.514644	2.663710	-1.482348
H	2.322046	0.191273	0.043730
H	-1.836720	2.247761	1.324606
H	-3.483878	2.413361	0.735483
H	-0.427068	4.460301	1.571515
H	-0.541299	6.759025	2.475975
H	-2.737697	7.905894	2.703169
H	-4.819208	6.724389	2.021614
H	-4.697470	4.420913	1.114580
H	-2.390991	3.331927	-2.642719
H	-3.057304	4.469195	-1.469467

H   -3.429714   0.746988 -2.081320
H   -5.635959 -0.323970 -2.462787
H   -7.720721   1.033564 -2.364927
H   -7.578172   3.474954 -1.901089
H   -5.367619   4.542025 -1.552674
H   6.297182   0.758762 -1.852574
H   6.598853 -0.749592 -1.032053
H   9.004799 -0.045540 -1.080725
H   10.823054   1.025154   0.210576
H   10.281331   2.634813   2.031016
H   7.903321   3.158488   2.540478
H   6.086443   2.088336   1.236042
H   3.826553 -0.461007   1.247979
H   5.537174 -0.807233   1.365736
H   5.388146 -3.165265   1.690570
H   5.050736 -5.418156   0.721329
H   3.981603 -5.666877 -1.512998
H   3.245848 -3.641794 -2.756646
H   3.582297 -1.389736 -1.777906

Conformer **A**: B3LYPD3/6-31+G(d,p) [5d]
Processing: namphos-h6pbed3.log
PG=C01

Method BasisSet Imaginary Freqs
RB3LYP 6-31+G(d,p) 0

HF Energy
-2849.7494763

ZPE	E298	S298	Squasihar	Equasihar	Strans	Srot
676.51623	712.182	328.162	283.537	712.375	46.099	40.530

Processing: namphos-h6pbed3.log
126
C   3.434859 -1.703811 -1.620412
C   4.125394 -1.844376 -0.408724
C   4.488591 -3.129874   0.013409
C   4.165741 -4.253108 -0.752402
C   3.471869 -4.103911 -1.955405
C   3.107732 -2.825037 -2.386190
C   4.487496 -0.631091   0.441723
N   4.772492   0.577352 -0.317088
C   6.080138   0.620920 -0.950477
C   7.173462   1.297331 -0.131404
C   8.506440   1.206285 -0.557316
C   9.529036   1.826809   0.161988
C   9.230886   2.547740   1.323800
C   7.905946   2.640363   1.755241
C   6.883071   2.019212   1.030685

C	3.805309	1.548162	-0.564579
C	4.186009	2.801833	-1.144202
C	3.233122	3.737082	-1.468909
C	1.852288	3.518085	-1.221906
C	1.462291	2.307378	-0.564468
C	2.460566	1.341223	-0.265735
C	0.073790	2.058077	-0.302811
C	-0.894589	2.964030	-0.742302
C	-0.479910	4.164420	-1.392972
C	0.849931	4.436313	-1.614170
C	-0.284985	0.827647	0.459316
C	-0.847032	-0.311371	-0.147353
C	-1.126649	-1.488103	0.573563
C	-0.898766	-1.468869	1.970130
C	-0.333623	-0.352346	2.594719
C	-0.020509	0.770257	1.829989
P	-1.757326	-2.965669	-0.374283
C	-0.790319	-4.419420	0.364901
C	0.693096	-4.103301	0.643722
C	1.421325	-5.330736	1.215832
C	1.313718	-6.542574	0.279970
C	-0.152831	-6.852699	-0.053548
C	-0.871983	-5.614192	-0.614126
O	-1.172941	-0.256143	-1.486056
C	-0.099056	-0.530943	-2.393597
O	-1.246130	-2.600351	2.657658
C	-0.951693	-2.689515	4.045165
N	-2.278848	2.694388	-0.560154
C	-3.183307	3.281260	-1.552132
C	-4.490366	2.522465	-1.675242
C	-4.489075	1.137780	-1.900189
C	-5.694286	0.459762	-2.092182
C	-6.912541	1.146789	-2.045155
C	-6.920333	2.522344	-1.805203
C	-5.712237	3.203494	-1.623107
C	-2.808102	2.727263	0.818991
C	-2.957058	4.128553	1.383421
C	-1.827455	4.848205	1.803812
C	-1.950063	6.155344	2.278731
C	-3.208105	6.764635	2.342041
C	-4.339882	6.057176	1.928367
C	-4.211540	4.749098	1.450491
C	-3.446461	-3.126259	0.463470
C	-4.169640	-4.435442	0.095028
C	-5.581498	-4.492268	0.706286
C	-6.424804	-3.271908	0.308853
C	-5.709534	-1.966659	0.684947
C	-4.303928	-1.908245	0.070691
H	-3.286837	-3.115009	1.548242
H	-3.592779	-5.300618	0.440312
H	-4.245354	-4.518820	-0.999506

H	-6.081122	-5.420619	0.401385
H	-5.494880	-4.526143	1.802335
H	-7.413764	-3.320553	0.781553
H	-6.593035	-3.289052	-0.778434
H	-5.628508	-1.900215	1.779828
H	-6.291574	-1.097291	0.356574
H	-3.807095	-0.976961	0.362850
H	-4.388918	-1.880783	-1.023552
H	-1.264974	-4.702812	1.313016
H	1.186393	-3.794574	-0.287702
H	0.791802	-3.267636	1.339550
H	2.473243	-5.082360	1.399052
H	0.979395	-5.585879	2.190637
H	1.800299	-7.419062	0.726699
H	1.852640	-6.319196	-0.651683
H	-0.669680	-7.187434	0.857980
H	-0.215240	-7.679723	-0.772283
H	-1.912286	-5.863676	-0.848258
H	-0.404671	-5.323546	-1.565853
H	0.426397	1.635580	2.311677
H	-0.139727	-0.340481	3.660045
H	0.715888	0.190408	-2.267228
H	-0.519374	-0.439320	-3.396957
H	0.279460	-1.549562	-2.245781
H	0.125866	-2.595387	4.232384
H	-1.286902	-3.680842	4.354014
H	-1.491926	-1.928078	4.622100
H	-1.225318	4.894837	-1.683671
H	1.142904	5.367400	-2.093570
H	3.538957	4.675131	-1.926016
H	5.231625	3.027194	-1.317184
H	2.138497	0.397326	0.151588
H	-2.148978	2.134023	1.451966
H	-3.778885	2.222452	0.807274
H	-0.848104	4.381207	1.749398
H	-1.065693	6.698137	2.601049
H	-3.304297	7.781005	2.713381
H	-5.320968	6.521865	1.976937
H	-5.092456	4.200727	1.126102
H	-2.671190	3.241504	-2.518878
H	-3.404975	4.341939	-1.346824
H	-3.541525	0.604646	-1.914204
H	-5.686748	-0.609286	-2.279058
H	-7.846372	0.611383	-2.192288
H	-7.860726	3.064720	-1.759564
H	-5.717832	4.275506	-1.440724
H	5.997649	1.108842	-1.928745
H	6.382436	-0.411185	-1.167666
H	8.744658	0.642860	-1.457412
H	10.556953	1.745744	-0.180300
H	10.025040	3.030081	1.886237

H 7.664199 3.198404 2.655504
H 5.852988 2.093928 1.364648
H 3.689139 -0.420788 1.160626
H 5.374478 -0.868311 1.042729
H 5.035319 -3.253490 0.945861
H 4.454109 -5.242489 -0.409711
H 3.215663 -4.975351 -2.550474
H 2.566700 -2.699002 -3.319745
H 3.157293 -0.714167 -1.966539

Conformer **A**: M06/6-31+G(d,p) [5d] optimized

Method Basis Set Imaginary Freqs
RM06 6-31+G(d,p)

HF
-2847.60921910

Processing: namphos-h5pm6.log
Frequency job incomplete: namphos-h5pm6.log
126
C 3.544503 -1.688400 -1.683081
C 4.112939 -1.840886 -0.416384
C 4.336010 -3.132208 0.068190
C 3.991877 -4.247546 -0.690536
C 3.413560 -4.086111 -1.947765
C 3.192946 -2.802660 -2.441534
C 4.502472 -0.643399 0.426169
N 4.798628 0.556461 -0.325570
C 6.102434 0.595701 -0.948706
C 7.188584 1.261181 -0.131570
C 8.504882 1.231709 -0.601309
C 9.530173 1.833888 0.119360
C 9.251483 2.474402 1.326520
C 7.943844 2.506400 1.801085
C 6.917183 1.903305 1.075450
C 3.836894 1.524501 -0.583933
C 4.219415 2.765712 -1.175006
C 3.269562 3.697291 -1.503313
C 1.893981 3.482290 -1.251203
C 1.503046 2.282346 -0.590642
C 2.497350 1.319535 -0.285392
C 0.120076 2.036379 -0.327977
C -0.847633 2.938228 -0.768707
C -0.430062 4.132473 -1.421256
C 0.896443 4.399490 -1.642560
C -0.229234 0.816111 0.439154
C -0.810299 -0.314936 -0.151366
C -1.112006 -1.471276 0.582277
C -0.865464 -1.449697 1.969019

C	-0.258151	-0.349982	2.575449
C	0.057803	0.760617	1.801900
P	-1.811257	-2.918513	-0.346389
C	-0.910113	-4.388933	0.408450
C	0.578361	-4.124288	0.649867
C	1.259707	-5.357047	1.236880
C	1.093087	-6.574567	0.334850
C	-0.377917	-6.830448	0.025792
C	-1.044637	-5.582663	-0.546469
O	-1.114611	-0.277063	-1.486709
C	-0.053429	-0.668977	-2.339927
O	-1.239335	-2.552746	2.670453
C	-0.725952	-2.734045	3.969020
N	-2.226646	2.680470	-0.593532
C	-3.132935	3.313496	-1.541452
C	-4.422061	2.551917	-1.715668
C	-4.397760	1.183595	-2.000750
C	-5.584251	0.497788	-2.239821
C	-6.809176	1.162306	-2.181408
C	-6.840672	2.521694	-1.884252
C	-5.650271	3.210871	-1.655351
C	-2.767245	2.610833	0.769385
C	-2.951784	3.960814	1.417925
C	-1.842477	4.715383	1.816389
C	-2.008158	5.973697	2.386950
C	-3.287676	6.496490	2.571147
C	-4.398554	5.752800	2.182344
C	-4.228018	4.494458	1.607895
C	-3.497313	-2.976313	0.485074
C	-4.318394	-4.173708	0.004728
C	-5.741167	-4.144876	0.560005
C	-6.448745	-2.837856	0.220375
C	-5.644487	-1.645627	0.724191
C	-4.226584	-1.669460	0.167271
H	-3.352754	-3.055034	1.575169
H	-3.829562	-5.115041	0.294369
H	-4.356354	-4.165286	-1.099389
H	-6.309570	-5.004690	0.179705
H	-5.701222	-4.256642	1.656291
H	-7.463684	-2.828786	0.640194
H	-6.561401	-2.760184	-0.874751
H	-5.603172	-1.675001	1.825672
H	-6.133798	-0.699221	0.451860
H	-3.657955	-0.806854	0.545433
H	-4.265360	-1.548421	-0.929588
H	-1.383185	-4.644179	1.371928
H	1.063010	-3.856470	-0.307452
H	0.734145	-3.266029	1.315876
H	2.325032	-5.149295	1.412807
H	0.814749	-5.569841	2.224681
H	1.553157	-7.461881	0.791236

H	1.630057	-6.392620	-0.611544
H	-0.899067	-7.124310	0.952231
H	-0.483917	-7.672539	-0.671691
H	-2.099003	-5.788174	-0.778529
H	-0.569090	-5.317756	-1.507112
H	0.525320	1.626737	2.271401
H	-0.047262	-0.339851	3.640856
H	0.809945	0.005481	-2.233019
H	-0.433654	-0.615564	-3.363268
H	0.260131	-1.703196	-2.124041
H	0.373125	-2.675479	3.972484
H	-1.033887	-3.734820	4.282949
H	-1.131134	-1.998324	4.677899
H	-1.176013	4.867485	-1.711559
H	1.194481	5.330112	-2.124994
H	3.572634	4.633966	-1.970597
H	5.268623	2.984614	-1.356703
H	2.175041	0.372160	0.137815
H	-2.109028	1.983186	1.378248
H	-3.733604	2.088537	0.717456
H	-0.839878	4.309919	1.675504
H	-1.136023	6.547500	2.693579
H	-3.416742	7.479178	3.019597
H	-5.400461	6.152295	2.326024
H	-5.097103	3.910445	1.302326
H	-2.619420	3.343962	-2.511856
H	-3.367612	4.361890	-1.270251
H	-3.436975	0.666549	-2.022632
H	-5.555492	-0.565671	-2.473453
H	-7.734195	0.619415	-2.364863
H	-7.791418	3.048103	-1.830555
H	-5.672547	4.277131	-1.427431
H	6.026178	1.080442	-1.933408
H	6.403733	-0.441191	-1.167948
H	8.724960	0.727528	-1.543297
H	10.549765	1.802246	-0.258897
H	10.051590	2.945329	1.893080
H	7.717556	3.005400	2.741007
H	5.892257	1.931901	1.443233
H	3.716303	-0.421794	1.161937
H	5.394060	-0.900688	1.020336
H	4.794079	-3.263763	1.049416
H	4.180080	-5.247024	-0.301663
H	3.141522	-4.956445	-2.541627
H	2.746965	-2.665636	-3.424945
H	3.383670	-0.686939	-2.080558

Conformer **B**: B3LYP/6-31G(d) [5d]

Processing: namphos-n5dbe.log

PG=C01

Method BasisSet Imaginary Freqs
RB3LYP 6-31G(d) 0

HF Energy
-2849.3615792

ZPE	E298	S298	Squasihar	Equasihar	Strans	Srot
677.88400	714.083	340.586	285.997	714.297	46.099	40.581

ccl00:/aue/chem126/aue/ark/pj/nam> gtg namphos-n5dbe.log
Processing: namphos-n5dbe.log
126

C	1.398856	3.795292	-1.083479
C	-0.064830	4.285387	-1.059588
C	-0.252323	5.441706	-2.063916
C	0.748832	6.584904	-1.817489
C	2.200280	6.086868	-1.833384
C	2.395882	4.939276	-0.833205
P	-1.233063	2.840290	-1.456513
C	-2.954892	3.568326	-1.092411
C	-3.912375	2.544492	-0.447935
C	-5.319503	3.130680	-0.237149
C	-5.920072	3.670555	-1.542332
C	-4.972222	4.678388	-2.206804
C	-3.572037	4.077361	-2.416755
C	-0.929912	1.706105	-0.003182
C	-0.683022	0.344880	-0.276887
C	-0.460974	-0.609430	0.737822
C	-0.470462	-0.158795	2.058972
C	-0.717955	1.174928	2.379564
C	-0.960619	2.097035	1.357196
C	-0.203003	-2.058266	0.456217
C	1.153761	-2.521273	0.349109
C	1.405575	-3.920482	0.171916
C	0.314522	-4.818994	0.108988
C	-0.975951	-4.361756	0.220939
C	-1.253507	-2.976402	0.400164
C	2.263704	-1.640015	0.465207
C	3.580554	-2.087943	0.376780
C	3.807434	-3.484735	0.164738
C	2.752330	-4.357996	0.078002
N	4.664414	-1.212269	0.471621
C	6.003213	-1.667373	0.813275
C	6.970686	-1.823755	-0.357108
C	8.349211	-1.864467	-0.105451
C	9.260441	-2.040180	-1.146137
C	8.804992	-2.170125	-2.461041
C	7.435372	-2.122980	-2.722114
C	6.524065	-1.951428	-1.676484
N	-2.605353	-2.531984	0.561578

C	-3.280665	-2.023829	-0.656734
C	-3.726976	-3.086193	-1.650907
C	-2.837855	-3.574617	-2.620409
C	-3.235423	-4.558960	-3.526102
C	-4.534311	-5.071828	-3.479785
C	-5.432169	-4.589841	-2.525794
C	-5.029800	-3.604027	-1.621467
O	-0.765715	-0.109488	-1.579758
C	0.431719	-0.078236	-2.357038
O	-1.266213	3.407632	1.608503
C	-1.313910	3.853203	2.954380
C	4.508728	0.224500	0.317632
C	4.375973	1.028470	1.609018
C	3.979533	0.439259	2.814433
C	3.847954	1.209877	3.972731
C	4.109073	2.580140	3.940580
C	4.507393	3.177093	2.741518
C	4.643756	2.404478	1.588418
C	-3.447385	-3.368544	1.418242
C	-4.576722	-2.590564	2.079260
C	-4.375713	-1.290116	2.562241
C	-5.404728	-0.608738	3.212989
C	-6.649936	-1.216557	3.395929
C	-6.859658	-2.510738	2.918089
C	-5.829717	-3.188479	2.260970
H	-0.287982	4.659818	-0.055043
H	-1.270976	5.840902	-2.000461
H	-0.126107	5.062743	-3.089322
H	0.606125	7.371851	-2.569789
H	0.536282	7.044575	-0.840685
H	2.889975	6.911804	-1.611532
H	2.450460	5.732250	-2.844476
H	2.259309	5.320368	0.189644
H	3.423841	4.556110	-0.887567
H	1.546895	3.007033	-0.335660
H	1.612742	3.344914	-2.065147
H	-2.840440	4.414027	-0.399819
H	-3.983586	1.656967	-1.094391
H	-3.515688	2.198511	0.510696
H	-5.975901	2.365993	0.198646
H	-5.260548	3.947811	0.497882
H	-6.898601	4.130809	-1.352323
H	-6.096226	2.832598	-2.232948
H	-4.892187	5.575110	-1.573792
H	-5.382257	5.012041	-3.169248
H	-2.920396	4.816686	-2.895915
H	-3.641284	3.233455	-3.118084
H	-0.290339	-0.872789	2.857855
H	-0.727883	1.477021	3.419937
H	0.765753	0.953827	-2.515875
H	0.175296	-0.521831	-3.321952

H	1.226885	-0.664390	-1.883228
H	-2.081685	3.319587	3.529617
H	-1.570957	4.913141	2.907397
H	-0.343058	3.736910	3.453214
H	-1.805184	-5.058344	0.157927
H	0.511824	-5.879442	-0.033051
H	2.945070	-5.416671	-0.081551
H	4.817856	-3.857894	0.039262
H	2.062399	-0.596791	0.671987
H	-2.593555	-1.333852	-1.147421
H	-4.150402	-1.447323	-0.323147
H	-1.830045	-3.170348	-2.666607
H	-2.533822	-4.920713	-4.273729
H	-4.845999	-5.835773	-4.187505
H	-6.448033	-4.975665	-2.489144
H	-5.737479	-3.221771	-0.888777
H	-2.797324	-3.780089	2.200173
H	-3.881584	-4.233233	0.887845
H	-3.410153	-0.817065	2.408848
H	-5.234647	0.400932	3.579059
H	-7.450595	-0.683606	3.902265
H	-7.825848	-2.991560	3.048921
H	-6.000021	-4.195376	1.884886
H	5.923540	-2.615170	1.357531
H	6.430921	-0.955299	1.532352
H	8.711262	-1.755527	0.915425
H	10.325891	-2.068519	-0.932713
H	9.513484	-2.302491	-3.274318
H	7.071590	-2.219175	-3.741904
H	5.458685	-1.912462	-1.883391
H	3.641307	0.410369	-0.326104
H	5.377862	0.598417	-0.240587
H	4.966316	2.874119	0.660943
H	4.721394	4.242205	2.706344
H	4.009797	3.178267	4.842589
H	3.541855	0.735269	4.901610
H	3.772814	-0.626256	2.844293

Conformer **C**: B3LYP/6-31G(d) [5d]

Processing: namphos-m5dbe.log
PG=C01

Method	BasisSet	Imaginary Freqs
RB3LYP	6-31G(d)	0

HF Energy
-2849.3601616

ZPE E298 S298 Squasihar Equasihar Strans Srot

677.82580 713.937 338.261 285.613 714.148 46.099 40.436

Processing: namphos-m5dbe.log
126

C	2.595597	3.730532	2.223319
C	2.439705	4.631334	1.159920
C	1.814180	5.857730	1.407734
C	1.355862	6.184483	2.686789
C	1.517332	5.281799	3.737816
C	2.139153	4.051884	3.501306
C	2.978758	4.291773	-0.214828
N	2.372571	3.082037	-0.797878
C	3.092795	2.621890	-2.003104
C	4.530206	2.222840	-1.727228
C	5.565116	2.675586	-2.553817
C	6.887547	2.284155	-2.329678
C	7.193968	1.440474	-1.261879
C	6.168970	0.987717	-0.425933
C	4.848037	1.370508	-0.659706
C	0.950446	3.194616	-1.033202
C	0.083855	2.200129	-0.594074
C	-1.335534	2.358447	-0.795429
C	-1.824386	3.519990	-1.475221
C	-0.903730	4.485652	-1.947119
C	0.442236	4.327622	-1.728990
C	-3.225319	3.661179	-1.651524
C	-4.114375	2.726748	-1.181663
C	-3.649720	1.571662	-0.478216
C	-2.275726	1.411013	-0.308780
C	0.562590	0.975511	0.124414
C	0.987737	-0.183197	-0.561587
C	1.310992	-1.385824	0.102774
C	1.230496	-1.384946	1.516915
C	0.834320	-0.242685	2.217497
C	0.497179	0.914338	1.516792
P	1.752434	-2.867890	-0.946265
C	3.396974	-3.500637	-0.227123
C	4.330833	-2.375426	0.259246
C	5.671749	-2.934282	0.768290
C	6.380700	-3.787149	-0.292870
C	5.457389	-4.898353	-0.810815
C	4.122319	-4.326663	-1.316596
O	1.157371	-0.098492	-1.929998
C	0.032672	-0.424531	-2.748136
O	1.574473	-2.551015	2.148025
C	1.434546	-2.629338	3.558144
N	-4.562250	0.631126	0.004903
C	-4.146090	-0.702323	0.404992
C	-3.816154	-0.894537	1.884316
C	-3.566638	0.183898	2.739512
C	-3.242325	-0.032760	4.081624

C	-3.162044	-1.331203	4.586165
C	-3.413511	-2.414992	3.739722
C	-3.742049	-2.195455	2.402159
C	0.511648	-4.149817	-0.283056
C	-0.929010	-3.724945	-0.636612
C	-1.968571	-4.756968	-0.167334
C	-1.671460	-6.153054	-0.731645
C	-0.241096	-6.588797	-0.387067
C	0.800966	-5.554900	-0.850995
C	-5.961536	0.954631	0.236367
C	-6.937117	0.491201	-0.842378
C	-8.303226	0.405200	-0.539594
C	-9.226742	0.017684	-1.509728
C	-8.794893	-0.298597	-2.800788
C	-7.436457	-0.223139	-3.109223
C	-6.513525	0.169798	-2.136055
H	0.602708	-4.193412	0.808085
H	1.797465	-5.893036	-0.545464
H	0.805765	-5.513003	-1.950732
H	-0.020290	-7.565858	-0.836733
H	-0.156688	-6.720190	0.702093
H	-2.396655	-6.883899	-0.350479
H	-1.791025	-6.133603	-1.825168
H	-1.963305	-4.800449	0.931989
H	-2.975865	-4.433415	-0.463737
H	-1.152606	-2.746402	-0.194185
H	-1.013658	-3.605871	-1.727508
H	3.174657	-4.152358	0.629393
H	4.521060	-1.674356	-0.565687
H	3.850575	-1.800471	1.056130
H	6.321921	-2.108294	1.087113
H	5.488134	-3.548017	1.663229
H	7.305849	-4.215330	0.115056
H	6.676233	-3.142981	-1.134013
H	5.261967	-5.616870	-0.000364
H	5.950529	-5.461443	-1.614255
H	3.486025	-5.140246	-1.682908
H	4.310742	-3.675349	-2.181993
H	0.175915	1.793826	2.066233
H	0.770805	-0.243423	3.298910
H	-0.183828	-1.497940	-2.697765
H	0.318003	-0.164787	-3.770237
H	-0.852690	0.151111	-2.457736
H	2.096222	-1.918917	4.070676
H	1.723163	-3.646855	3.828899
H	0.397631	-2.447963	3.869503
H	1.136415	5.077772	-2.094630
H	-1.278787	5.357157	-2.479621
H	-3.596873	4.531949	-2.187273
H	-5.173332	2.854417	-1.377846
H	-1.900245	0.566366	0.252627

H 2.540528 1.756296 -2.382331
H 3.082495 3.390233 -2.799366
H 5.333082 3.342805 -3.381380
H 7.676522 2.646111 -2.984137
H 8.222287 1.139638 -1.078984
H 6.399891 0.333137 0.410607
H 4.049867 1.019025 -0.012637
H 4.051676 4.086345 -0.136355
H 2.886143 5.174364 -0.872926
H 1.682967 6.563923 0.591145
H 0.871620 7.142365 2.858934
H 1.163571 5.533169 4.734447
H 2.273665 3.344978 4.316539
H 3.073871 2.773025 2.038358
H -6.052831 2.037618 0.377629
H -6.259181 0.511175 1.196491
H -8.645548 0.642370 0.466258
H -10.282136 -0.044541 -1.257129
H -9.512241 -0.605278 -3.557263
H -7.090359 -0.470884 -4.109420
H -5.456343 0.224616 -2.378365
H -3.282226 -0.992322 -0.205028
H -4.949517 -1.398784 0.130045
H -3.944867 -3.044068 1.751716
H -3.365007 -3.430692 4.124222
H -2.915224 -1.498924 5.631307
H -3.053780 0.817394 4.732296
H -3.622214 1.196270 2.351401

Conformer **D**: B3LYP/6-31G(d) [5d]

Processing: namphos-o5dbe.log
PG=C01

Method	BasisSet	Imaginary Freqs
RB3LYP	6-31G(d)	0

HF Energy
-2849.3599074

ZPE	E298	S298	Squasihar	Equasihar	Strans	Srot
677.93302	714.097	339.798	285.883	714.311	46.099	40.599

Processing: namphos-o5dbe.log
126
C -0.819401 5.446083 -2.116365
C -0.557724 4.474803 -0.943104
C 0.952879 4.435880 -0.625367
C 1.514339 5.838459 -0.337328
C 1.237633 6.808261 -1.494716

C	-0.259644	6.850868	-1.830465
P	-1.148103	2.745692	-1.422820
C	-3.049755	2.799078	-1.392513
C	-3.720568	3.724017	-0.359050
C	-5.253912	3.584608	-0.390479
C	-5.818270	3.831019	-1.797289
C	-5.149591	2.913716	-2.831873
C	-3.617455	3.051085	-2.807289
C	-0.827195	1.659459	0.068922
C	-0.608432	0.295195	-0.222982
C	-0.356256	-0.674116	0.766835
C	-0.315383	-0.238132	2.093364
C	-0.532188	1.094081	2.433285
C	-0.795445	2.035900	1.432323
C	-0.077897	-2.115422	0.465847
C	-1.102054	-3.063430	0.431108
C	-0.786091	-4.440385	0.246042
C	0.515311	-4.861276	0.117174
C	1.581576	-3.932212	0.163369
C	1.289801	-2.540569	0.336638
C	2.373369	-1.625259	0.428502
C	3.701369	-2.033317	0.327943
C	3.969851	-3.424250	0.128548
C	2.940200	-4.329678	0.058699
N	-2.461368	-2.661060	0.626862
C	-3.174520	-2.138054	-0.562380
C	-3.626920	-3.187897	-1.567250
C	-4.924976	-3.716595	-1.526428
C	-5.333455	-4.691124	-2.440220
C	-4.446367	-5.151040	-3.414900
C	-3.152084	-4.627671	-3.472081
C	-2.748501	-3.654465	-2.557083
N	4.752779	-1.116117	0.396682
C	4.531185	0.307849	0.212667
C	4.282020	1.128295	1.477232
C	4.030637	0.533572	2.717215
C	3.792494	1.319956	3.848245
C	3.800241	2.711554	3.752526
C	4.051038	3.315182	2.516422
C	4.292970	2.528045	1.391314
O	-0.748387	-0.125501	-1.535602
C	0.442035	-0.165896	-2.328637
O	-1.043859	3.350539	1.723249
C	-0.908200	3.792954	3.064260
C	-3.265748	-3.535782	1.481478
C	-4.391695	-2.797356	2.191677
C	-4.199765	-1.509354	2.710272
C	-5.223072	-0.866300	3.407253
C	-6.453250	-1.500679	3.601101
C	-6.654060	-2.782547	3.087499
C	-5.629970	-3.421897	2.384446

C	6.113248	-1.513477	0.723609
C	7.069823	-1.643852	-0.458944
C	8.452316	-1.612448	-0.229130
C	9.354956	-1.763435	-1.281217
C	8.886161	-1.940697	-2.585793
C	7.511952	-1.965404	-2.825282
C	6.609596	-1.818337	-1.768332
H	-0.498974	1.385238	3.476078
H	-0.111230	-0.961751	2.877541
H	-1.087570	4.840843	-0.059524
H	-0.354487	5.048472	-3.030628
H	-1.893737	5.520389	-2.322179
H	-0.438751	7.505588	-2.693525
H	-0.807514	7.292482	-0.984640
H	1.797571	6.479333	-2.382909
H	1.603206	7.813993	-1.249073
H	2.593561	5.775899	-0.141830
H	1.052969	6.229897	0.581735
H	1.489564	4.005504	-1.484944
H	1.147739	3.777469	0.227741
H	-3.303070	1.761480	-1.128310
H	-3.344447	4.061024	-3.145234
H	-3.163019	2.352887	-3.520273
H	-5.529246	3.132867	-3.838617
H	-5.419571	1.869139	-2.616795
H	-5.643318	4.880756	-2.078003
H	-6.906147	3.683600	-1.803480
H	-5.707069	4.279879	0.329078
H	-5.530134	2.571619	-0.062041
H	-3.456580	4.769156	-0.576598
H	-3.340957	3.510356	0.645023
H	0.133656	-0.524716	-3.313378
H	0.870644	0.837643	-2.431050
H	1.179023	-0.852322	-1.899942
H	-1.099280	4.867687	3.042431
H	-1.638981	3.311188	3.727003
H	0.104229	3.609734	3.446599
H	0.739990	-5.916287	-0.024808
H	-1.595441	-5.161028	0.195973
H	2.144789	-0.586245	0.625604
H	3.163201	-5.383315	-0.094714
H	4.990194	-3.767755	-0.002550
H	-2.588882	-3.955288	2.235780
H	-3.697080	-4.393914	0.938406
H	-4.046048	-1.584504	-0.195825
H	-2.510816	-1.426324	-1.054497
H	-5.623938	-3.352406	-0.776368
H	-6.345575	-5.085661	-2.394292
H	-4.762740	-5.906249	-4.129860
H	-2.458861	-4.972525	-4.235335
H	-1.744233	-3.242562	-2.611839

H -5.793071 -4.419310 1.980915
H -7.608862 -3.283427 3.226470
H -7.249505 -0.997534 4.143587
H -5.060427 0.134313 3.800462
H -3.246161 -1.015167 2.548419
H 6.523415 -0.778589 1.429959
H 6.077848 -2.458582 1.277374
H 8.824361 -1.466313 0.783476
H 10.423671 -1.735382 -1.084805
H 9.587625 -2.053745 -3.408022
H 7.137665 -2.098591 -3.837071
H 5.540569 -1.834862 -1.958794
H 5.409404 0.716653 -0.304991
H 3.691103 0.440575 -0.479647
H 4.494252 3.005175 0.433815
H 4.065763 4.398715 2.430660
H 3.620267 3.322807 4.633085
H 3.601070 0.840496 4.804921
H 4.015086 -0.549332 2.794656

Conformer **E**: B3LYP/6-31G(d) [5d]

Processing: namphos-i5dbe.log
PG=C01

Method	BasisSet	Imaginary Freqs
RB3LYP	6-31G(d)	0

HF Energy
-2849.3595476

ZPE	E298	S298	Squasihar	Equasihar	Strans	Srot
678.06249	714.138	337.337	285.485	714.348	46.099	40.626

ccl00:/aue/chem126/aue/ark/pj/nam> gtg namphos-i5dbe.log
Processing: namphos-i5dbe.log
126
C 4.878139 -0.977535 -0.343118
C 4.324580 -2.399102 -0.583240
C 5.395288 -3.451051 -0.231927
C 6.711675 -3.202778 -0.991476
C 7.248868 -1.783912 -0.759802
C 6.187678 -0.731819 -1.110926
P 2.724887 -2.612669 0.422614
C 2.141674 -4.366378 -0.021758
C 0.604105 -4.485747 -0.061807
C 0.151087 -5.929815 -0.338508
C 0.730253 -6.919148 0.682185
C 2.258292 -6.798100 0.761432
C 2.693504 -5.349148 1.039370
C 1.616954 -1.483110 -0.573720

C	1.114433	-0.336553	0.072507
C	0.302327	0.617191	-0.579446
C	-0.041692	0.357087	-1.909536
C	0.434184	-0.761764	-2.592532
C	1.280217	-1.661494	-1.937075
C	-0.179351	1.863762	0.097130
C	-1.576631	1.970968	0.426064
C	-2.062877	3.161354	1.058195
C	-1.155353	4.202319	1.354625
C	0.173477	4.091595	1.031695
C	0.688701	2.930374	0.386074
C	-2.499307	0.912692	0.188257
C	-3.855138	1.021372	0.495661
C	-4.321018	2.238891	1.085732
C	-3.443756	3.255597	1.365514
N	-4.762098	-0.008546	0.232534
C	-6.060974	-0.082576	0.882929
C	-7.244280	0.432726	0.067238
C	-8.543815	0.053835	0.432242
C	-9.650706	0.529728	-0.269534
C	-9.473491	1.389239	-1.357289
C	-8.183758	1.765838	-1.732676
C	-7.076356	1.291079	-1.024386
N	2.073367	2.874167	0.049255
C	2.418833	2.815949	-1.388151
C	2.356641	4.144294	-2.131561
C	1.123677	4.726153	-2.466322
C	1.069140	5.946378	-3.140271
C	2.248071	6.606317	-3.497445
C	3.480577	6.036860	-3.175470
C	3.529929	4.816791	-2.497006
O	1.477388	-0.101858	1.378866
C	0.542605	-0.534084	2.367214
O	1.817564	-2.754870	-2.560488
C	1.502268	-2.990036	-3.923357
C	-4.455018	-1.113088	-0.662761
C	-4.087572	-2.426119	0.022120
C	-3.447398	-2.451349	1.267491
C	-3.109278	-3.665846	1.868117
C	-3.407198	-4.873144	1.232248
C	-4.046762	-4.857852	-0.009647
C	-4.386683	-3.642402	-0.605268
C	3.002716	3.738079	0.788361
C	3.045690	3.529573	2.291611
C	3.113841	4.637178	3.145336
C	3.244107	4.472722	4.526126
C	3.305490	3.190047	5.071637
C	3.236302	2.078902	4.226776
C	3.109201	2.244617	2.846855
H	4.076892	-2.505806	-1.646980
H	5.032966	-4.458410	-0.466577

H	5.592101	-3.428839	0.850613
H	7.459137	-3.949055	-0.691188
H	6.537725	-3.351029	-2.067819
H	8.161724	-1.621004	-1.347614
H	7.532124	-1.671886	0.297360
H	5.987804	-0.765195	-2.192550
H	6.563804	0.276727	-0.893610
H	4.131197	-0.227067	-0.628087
H	5.060389	-0.840258	0.732763
H	2.533678	-4.645104	-1.008414
H	0.185993	-4.158319	0.902284
H	0.183879	-3.821404	-0.821435
H	-0.945367	-5.976271	-0.337899
H	0.478051	-6.220188	-1.348534
H	0.439018	-7.947134	0.428659
H	0.301428	-6.707296	1.673260
H	2.699495	-7.133920	-0.189123
H	2.654675	-7.462652	1.540627
H	3.786237	-5.297026	1.095452
H	2.323592	-5.043233	2.028807
H	-0.680693	1.063620	-2.433310
H	0.157360	-0.907202	-3.630007
H	-0.456182	-0.127303	2.172495
H	0.910259	-0.149913	3.321051
H	0.498988	-1.630257	2.404301
H	0.425480	-3.147156	-4.067846
H	2.038978	-3.899443	-4.199889
H	1.835539	-2.163173	-4.563956
H	0.839388	4.915498	1.252558
H	-1.526244	5.105719	1.833968
H	-3.812800	4.172451	1.820131
H	-5.376986	2.377845	1.287444
H	-2.114938	-0.011706	-0.222294
H	1.757654	2.094722	-1.867007
H	3.435847	2.410821	-1.460987
H	0.201566	4.218981	-2.195903
H	0.104869	6.380490	-3.392295
H	2.204898	7.555020	-4.026047
H	4.403719	6.539787	-3.452349
H	4.494479	4.375767	-2.253031
H	3.996640	3.504404	0.383116
H	2.848588	4.808723	0.573877
H	3.067096	5.640318	2.725273
H	3.293595	5.345511	5.172386
H	3.405523	3.056461	6.145817
H	3.287671	1.075843	4.643640
H	3.035268	1.379555	2.196711
H	-6.008303	0.450970	1.838502
H	-6.246191	-1.132421	1.147474
H	-8.688625	-0.622033	1.273300
H	-10.650968	0.224882	0.027352

H -10.334372 1.758027 -1.908509
H -8.035339 2.431449 -2.579196
H -6.073776 1.585535 -1.320058
H -3.651184 -0.798915 -1.336729
H -5.330996 -1.286276 -1.303562
H -4.897830 -3.637576 -1.566244
H -4.293092 -5.791920 -0.508034
H -3.150961 -5.817971 1.703906
H -2.617547 -3.668545 2.837510
H -3.221406 -1.515047 1.769320

Conformer **F**: B3LYP/6-31G(d) [5d]

Processing: namphos-p5dbe.log
PG=C01

Method	BasisSet	Imaginary Freqs
RB3LYP	6-31G(d)	0

HF Energy
-2849.3594087

ZPE	E298	S298	Squasihar	Equasihar	Strans	Srot
677.88534	714.048	340.168	285.625	714.263	46.099	40.569

Processing: namphos-p5dbe.log
126
C -2.944930 -2.845887 3.239925
C -3.204961 -3.827901 2.273385
C -3.010864 -5.170574 2.612607
C -2.567722 -5.531242 3.888054
C -2.311004 -4.546788 4.842090
C -2.501016 -3.201001 4.513180
C -3.737202 -3.436385 0.907652
N -2.897045 -2.455059 0.213979
C -3.608703 -1.736963 -0.868628
C -4.105048 -2.600697 -2.020512
C -3.253130 -2.917666 -3.089566
C -3.691715 -3.725113 -4.139743
C -4.995241 -4.227984 -4.141742
C -5.856871 -3.913583 -3.089420
C -5.412384 -3.105707 -2.040436
C -1.549707 -2.855646 -0.076667
C -0.500880 -1.945601 0.072214
C 0.850872 -2.370425 -0.174435
C 1.098711 -3.717376 -0.593506
C 0.008159 -4.603874 -0.752326
C -1.275244 -4.187541 -0.497728
C 2.439865 -4.118485 -0.825135
C 3.495806 -3.260483 -0.645630
C 3.275562 -1.921663 -0.191753

C	1.963328	-1.508732	0.032185
C	-0.740230	-0.554912	0.577729
C	-0.989091	0.540616	-0.275751
C	-1.199409	1.850182	0.204615
C	-1.165620	2.037166	1.607575
C	-0.895512	0.972200	2.471573
C	-0.683594	-0.302456	1.949404
P	-1.526195	3.193654	-1.051401
C	-3.201539	3.915295	-0.505590
C	-4.173381	2.838676	0.020092
C	-5.546299	3.434787	0.378507
C	-6.174812	4.187301	-0.802561
C	-5.213117	5.250559	-1.350183
C	-3.848574	4.639165	-1.710308
O	-1.136702	0.284572	-1.626677
C	0.024753	0.413976	-2.447638
O	-1.436526	3.300652	2.061384
C	-1.417073	3.543967	3.458740
N	4.361186	-1.063322	-0.001454
C	4.196649	0.377232	0.099972
C	4.136144	0.947397	1.515149
C	3.771194	0.164576	2.616132
C	3.703132	0.724279	3.894598
C	3.997267	2.074429	4.089222
C	4.365024	2.863344	2.996182
C	4.438171	2.300601	1.721996
C	-0.287158	4.525538	-0.500367
C	1.153808	4.011105	-0.705708
C	2.205272	5.070469	-0.334671
C	1.988074	6.376454	-1.111112
C	0.559296	6.899771	-0.912471
C	-0.495657	5.840514	-1.279986
C	5.715042	-1.556488	0.197873
C	6.627878	-1.502764	-1.024501
C	8.016764	-1.569830	-0.846136
C	8.879173	-1.557463	-1.941760
C	8.363499	-1.469098	-3.237734
C	6.983033	-1.394108	-3.424734
C	6.120832	-1.411472	-2.324948
H	-0.433985	4.729122	0.565586
H	-1.493202	6.248126	-1.080735
H	-0.447298	5.639817	-2.360993
H	0.397709	7.807144	-1.509300
H	0.425067	7.190420	0.140199
H	2.719426	7.134234	-0.800799
H	2.161179	6.193635	-2.182213
H	2.148246	5.274123	0.744800
H	3.213164	4.677119	-0.524182
H	1.319541	3.103472	-0.112765
H	1.289265	3.729546	-1.761358
H	-3.027319	4.641828	0.300336

H	-4.305184	2.062469	-0.748748
H	-3.752613	2.339213	0.897384
H	-6.217957	2.637230	0.723265
H	-5.425502	4.127782	1.224870
H	-7.125372	4.646914	-0.501496
H	-6.412226	3.470635	-1.602739
H	-5.071456	6.037540	-0.594055
H	-5.646667	5.740223	-2.232207
H	-3.186121	5.418552	-2.103072
H	-3.980543	3.913422	-2.525630
H	-0.484142	-1.128650	2.625165
H	-0.855085	1.118882	3.544292
H	0.368532	1.454584	-2.474193
H	-0.283273	0.115444	-3.452484
H	0.830681	-0.241498	-2.100404
H	-2.168591	2.940551	3.984306
H	-1.656553	4.602235	3.579365
H	-0.427576	3.344255	3.889959
H	-2.099771	-4.877993	-0.634222
H	0.200188	-5.623472	-1.079749
H	2.626892	-5.133819	-1.167990
H	4.499767	-3.596310	-0.880679
H	1.770359	-0.516661	0.419362
H	-2.934198	-0.973792	-1.257261
H	-4.461412	-1.224468	-0.404935
H	-2.242183	-2.519198	-3.096920
H	-3.018117	-3.956095	-4.961245
H	-5.338902	-4.853434	-4.961625
H	-6.876297	-4.291439	-3.087362
H	-6.094132	-2.853924	-1.230344
H	-4.714062	-2.950992	1.037883
H	-3.937907	-4.345894	0.317331
H	-3.207694	-5.942308	1.871746
H	-2.420431	-6.580161	4.132364
H	-1.965721	-4.823648	5.834865
H	-2.307652	-2.428430	5.253605
H	-3.085355	-1.800769	2.978987
H	5.662581	-2.583502	0.576464
H	6.173950	-0.972201	1.007274
H	8.425569	-1.630352	0.161016
H	9.953508	-1.609382	-1.784670
H	9.033828	-1.454542	-4.092938
H	6.572485	-1.321288	-4.428599
H	5.046795	-1.350481	-2.473661
H	3.294645	0.661045	-0.454173
H	5.033202	0.849585	-0.432900
H	4.737409	2.918316	0.877273
H	4.605172	3.914078	3.137134
H	3.947456	2.508197	5.084535
H	3.420640	0.100930	4.739218
H	3.539301	-0.886055	2.470146

Conformer **G**: B3LYP/6-31G(d) [5d]

Processing: namphos-k5dbe.log
PG=C01

Method	BasisSet	Imaginary Freqs
RB3LYP	6-31G(d)	0

HF Energy
-2849.3591746

ZPE	E298	S298	Squasihar	Equasihar	Strans	Srot
678.10952	714.245	339.037	286.491	714.452	46.099	40.669

Processing: namphos-k5dbe.log
126

C	4.249059	2.341319	0.922584
C	3.295155	3.543637	0.763138
C	4.056967	4.750001	0.173477
C	5.305605	5.103673	1.001338
C	6.243408	3.899705	1.160421
C	5.494079	2.698127	1.752035
P	1.815646	3.079267	-0.338419
C	0.566601	4.465635	0.058042
C	-0.895522	3.975288	0.094545
C	-1.880292	5.129781	0.351707
C	-1.726996	6.256651	-0.679068
C	-0.273246	6.743525	-0.748471
C	0.697753	5.579201	-1.008203
C	1.135437	1.598930	0.578079
C	0.956887	0.401722	-0.144550
C	0.482189	-0.786241	0.455237
C	0.184366	-0.741601	1.819955
C	0.342307	0.420006	2.574462
C	0.811879	1.582110	1.956920
C	0.255334	-2.045589	-0.320383
C	-1.101146	-2.471590	-0.557495
C	-1.347592	-3.678076	-1.294245
C	-0.246166	-4.424566	-1.775311
C	1.039448	-4.008922	-1.540058
C	1.319099	-2.813308	-0.811028
C	-2.225422	-1.729524	-0.105862
C	-3.535523	-2.127972	-0.352819
C	-3.753251	-3.334915	-1.079556
C	-2.686184	-4.079807	-1.524645
N	-4.614053	-1.352516	0.137816
C	-5.925437	-1.985398	0.267852
C	-6.765234	-1.378453	1.381538
C	-8.110679	-1.060510	1.166311

C	-8.901411	-0.556797	2.202891
C	-8.350276	-0.359141	3.469179
C	-7.005311	-0.668789	3.692543
C	-6.220521	-1.175814	2.657396
N	2.670767	-2.415439	-0.617347
C	3.213429	-2.451048	0.755496
C	3.580455	-3.835882	1.272794
C	2.588021	-4.740319	1.681822
C	2.926401	-6.011586	2.146286
C	4.266721	-6.401357	2.214317
C	5.264045	-5.510471	1.815796
C	4.920366	-4.239203	1.348954
O	1.296025	0.360222	-1.479942
C	0.241996	0.653714	-2.394878
O	0.971833	2.760755	2.632884
C	0.665897	2.803359	4.017611
C	-4.652241	0.082464	-0.201261
C	-5.283917	0.403983	-1.550478
C	-4.637479	0.064503	-2.749271
C	-5.222155	0.356323	-3.981504
C	-6.463701	0.996152	-4.037212
C	-7.115517	1.339883	-2.852633
C	-6.528197	1.043063	-1.619857
C	3.627494	-2.822862	-1.651583
C	4.806622	-1.874375	-1.796217
C	4.598342	-0.504417	-2.013378
C	5.683062	0.345937	-2.230821
C	6.988905	-0.154865	-2.226635
C	7.204036	-1.514958	-2.004621
C	6.116679	-2.367293	-1.792103
H	2.918722	3.820434	1.754861
H	3.404313	5.628801	0.120912
H	4.358465	4.521115	-0.859474
H	5.834984	5.943649	0.532294
H	4.991100	5.450198	1.997261
H	7.102893	4.165093	1.789935
H	6.649046	3.623351	0.175777
H	5.188767	2.934687	2.782607
H	6.158418	1.826485	1.814691
H	3.724269	1.500800	1.389939
H	4.568110	1.991318	-0.068999
H	0.809625	4.884191	1.044125
H	-1.144619	3.501187	-0.867409
H	-1.024117	3.207949	0.863249
H	-2.909709	4.747414	0.348271
H	-1.700848	5.534229	1.359232
H	-2.400542	7.089580	-0.438574
H	-2.030156	5.884443	-1.668998
H	-0.007537	7.232984	0.200583
H	-0.161962	7.503746	-1.532892
H	1.724036	5.959583	-1.055812

H	0.488936	5.146383	-1.997399
H	-0.184899	-1.640997	2.305856
H	0.097964	0.404634	3.629903
H	-0.621237	-0.001649	-2.227299
H	0.646281	0.475333	-3.393806
H	-0.067315	1.703517	-2.313678
H	-0.390187	2.569924	4.205365
H	0.870577	3.827271	4.335723
H	1.296102	2.112290	4.592197
H	1.866004	-4.618727	-1.886301
H	-0.431400	-5.346498	-2.322254
H	-2.864069	-4.992183	-2.089859
H	-4.761726	-3.656246	-1.314464
H	-2.055616	-0.831556	0.475120
H	2.478635	-1.989037	1.416892
H	4.101814	-1.810846	0.768715
H	1.543218	-4.444455	1.637432
H	2.143697	-6.696915	2.461838
H	4.530361	-7.390462	2.579819
H	6.309943	-5.802225	1.870213
H	5.701312	-3.546164	1.043963
H	3.081544	-2.836936	-2.600708
H	4.012723	-3.843778	-1.489708
H	3.584392	-0.113598	-2.007188
H	5.507087	1.403412	-2.411479
H	7.830776	0.511402	-2.396884
H	8.214818	-1.915157	-1.997353
H	6.286661	-3.429277	-1.627255
H	-6.501412	-1.960584	-0.672153
H	-5.752030	-3.040064	0.506673
H	-8.542235	-1.207513	0.178685
H	-9.944894	-0.315145	2.017763
H	-8.961690	0.035329	4.276462
H	-6.568941	-0.517251	4.676645
H	-5.172982	-1.410985	2.825989
H	-3.628207	0.460265	-0.172019
H	-5.205269	0.600960	0.588495
H	-7.040824	1.311213	-0.698772
H	-8.080453	1.839474	-2.885546
H	-6.916660	1.226674	-4.997911
H	-4.705862	0.089789	-4.900229
H	-3.670535	-0.430255	-2.715091

Conformer **H**: B3LYP/6-31G(d) [5d]

Processing: namphos-j5dbe.log
PG=C01

Method	BasisSet	Imaginary Freqs
RB3LYP	6-31G(d)	0

HF Energy
-2849.3586009

ZPE	E298	S298	Squasihar	Equasihar	Strans	Srot
678.06400	714.052	333.824	285.208	714.256	46.099	40.508

Processing: namphos-j5dbe.log
126

C	-4.698431	1.133198	0.474168
C	-3.999242	2.447875	0.064619
C	-4.817890	3.657947	0.557963
C	-6.279908	3.597611	0.079255
C	-6.961518	2.285348	0.489000
C	-6.157775	1.076052	-0.007264
P	-2.226336	2.434394	0.751777
C	-1.530223	4.128846	0.238824
C	-0.021448	4.087752	-0.080336
C	0.526532	5.485615	-0.417559
C	0.255437	6.494898	0.706418
C	-1.235096	6.529091	1.070463
C	-1.765209	5.123663	1.401830
C	-1.427978	1.231698	-0.437522
C	-0.927255	0.027192	0.096783
C	-0.241939	-0.930774	-0.684649
C	-0.049687	-0.631200	-2.035179
C	-0.562365	0.529131	-2.617360
C	-1.269558	1.441504	-1.829153
C	0.280383	-2.212994	-0.107764
C	1.683427	-2.327424	0.193367
C	2.185009	-3.559769	0.725064
C	1.292963	-4.631075	0.955014
C	-0.039740	-4.512304	0.647602
C	-0.562610	-3.309594	0.097890
C	2.594823	-1.250743	0.004435
C	3.959321	-1.373809	0.269412
C	4.439215	-2.629375	0.762799
C	3.573297	-3.667190	0.993201
N	4.862611	-0.329936	0.059116
C	6.197170	-0.332965	0.638151
C	7.318600	-0.827095	-0.272811
C	8.649371	-0.551366	0.072369
C	9.702104	-1.006801	-0.720093
C	9.438725	-1.742052	-1.879332
C	8.117816	-2.015734	-2.234192
C	7.064421	-1.561694	-1.435364
N	-1.939715	-3.245117	-0.318256
C	-2.069233	-3.511529	-1.769858
C	-3.401459	-3.102440	-2.366965
C	-4.215395	-4.043607	-3.008142
C	-5.416552	-3.661946	-3.611617

C	-5.818422	-2.326679	-3.579163
C	-5.013630	-1.378830	-2.939821
C	-3.816008	-1.762979	-2.337311
O	-1.183132	-0.261407	1.416661
C	-0.154132	0.047477	2.356624
O	-1.829378	2.580893	-2.340618
C	-1.699012	2.842837	-3.728735
C	4.530003	0.862988	-0.706310
C	4.272796	2.110548	0.132820
C	3.652364	2.032708	1.386521
C	3.422552	3.186554	2.138304
C	3.810665	4.435781	1.647665
C	4.430587	4.523174	0.399238
C	4.662100	3.366850	-0.348297
C	-2.894615	-4.080608	0.429397
C	-2.956715	-3.832763	1.923075
C	-2.939393	-4.912053	2.815049
C	-3.072264	-4.710350	4.190679
C	-3.219889	-3.417422	4.693721
C	-3.235866	-2.333454	3.811682
C	-3.109686	-2.538501	2.437435
H	-3.943672	2.496623	-1.030540
H	-4.365948	4.593180	0.208742
H	-4.801686	3.688659	1.657995
H	-6.834470	4.458646	0.475429
H	-6.303008	3.689204	-1.017083
H	-7.988082	2.250625	0.101272
H	-7.038803	2.243766	1.585720
H	-6.178918	1.057319	-1.107053
H	-6.623139	0.140182	0.327525
H	-4.145336	0.270017	0.085788
H	-4.679013	1.045542	1.570249
H	-2.059705	4.487169	-0.653375
H	0.526656	3.688733	0.786060
H	0.180786	3.409585	-0.913403
H	1.603434	5.419181	-0.617136
H	0.053509	5.841735	-1.345450
H	0.603083	7.495416	0.416392
H	0.836579	6.205899	1.594871
H	-1.806662	6.942769	0.225926
H	-1.405867	7.202272	1.921172
H	-2.826857	5.183186	1.663613
H	-1.251871	4.744568	2.297456
H	0.500306	-1.333416	-2.656704
H	-0.415431	0.699731	-3.677222
H	0.793863	-0.425609	2.079115
H	-0.493592	-0.353600	3.313971
H	-0.022279	1.133646	2.439886
H	-0.647335	2.958234	-4.021702
H	-2.226502	3.782164	-3.904776
H	-2.157243	2.049762	-4.333756

H	-0.699840	-5.355260	0.811948
H	1.680567	-5.561294	1.364847
H	3.958023	-4.611621	1.371823
H	5.499578	-2.780384	0.927590
H	2.193136	-0.307587	-0.342714
H	-1.271181	-2.959677	-2.271499
H	-1.893941	-4.583200	-1.980250
H	-3.903918	-5.085706	-3.037083
H	-6.035670	-4.407781	-4.103585
H	-6.751606	-2.024579	-4.047470
H	-5.318886	-0.336145	-2.914353
H	-3.190653	-1.025977	-1.842812
H	-3.876778	-3.861152	-0.005353
H	-2.727480	-5.159037	0.247247
H	-2.824431	-5.923261	2.429215
H	-3.055935	-5.562146	4.865996
H	-3.321058	-3.254834	5.763819
H	-3.352352	-1.321958	4.192932
H	-3.104182	-1.690863	1.762871
H	6.183382	-0.920420	1.563053
H	6.425946	0.693653	0.952999
H	8.861516	0.027436	0.969699
H	10.727589	-0.783145	-0.437356
H	10.257497	-2.094766	-2.500734
H	7.902433	-2.584750	-3.134995
H	6.037458	-1.776960	-1.714908
H	3.666017	0.641921	-1.340361
H	5.360282	1.070020	-1.396011
H	5.159335	3.439780	-1.313676
H	4.747276	5.488818	0.013616
H	3.638399	5.332584	2.236588
H	2.945135	3.108921	3.111716
H	3.359150	1.062460	1.776986

Conformer **I**: B3LYP/6-31G(d) [5d]

Processing: namphos-l5dbe.log
PG=C01

Method	BasisSet	Imaginary Freqs
RB3LYP	6-31G(d)	0

HF Energy
-2849.3558265

ZPE	E298	S298	Squasihar	Equasihar	Strans	Srot
677.99608	714.038	334.160	285.887	714.243	46.099	40.560

Processing: namphos-l5dbe.log
126

C	-4.862286	3.647091	0.662252
C	-3.933170	2.841136	-0.274323
C	-4.732768	1.713535	-0.956996
C	-5.958113	2.262240	-1.708689
C	-6.872180	3.081319	-0.786622
C	-6.087868	4.199588	-0.086182
P	-2.508946	2.132467	0.748049
C	-1.339031	3.616467	1.053251
C	-1.375787	4.794617	0.059571
C	-0.356339	5.887068	0.431704
C	-0.557201	6.387072	1.869227
C	-0.517889	5.221001	2.867247
C	-1.533047	4.125169	2.501235
C	-1.506313	1.086348	-0.441906
C	-0.977611	-0.117525	0.077358
C	-0.195006	-1.009461	-0.690318
C	0.063409	-0.652485	-2.015620
C	-0.444470	0.517817	-2.577110
C	-1.224341	1.378500	-1.799184
C	0.391765	-2.266309	-0.120059
C	-0.385072	-3.408533	0.111166
C	0.222983	-4.573768	0.657307
C	1.566094	-4.613986	0.936168
C	2.390794	-3.496256	0.681925
C	1.806187	-2.299826	0.153018
C	2.652611	-1.174293	-0.056455
C	4.027515	-1.221258	0.174024
C	4.589599	-2.440773	0.668808
C	3.787550	-3.523024	0.925096
N	-1.775672	-3.432077	-0.249000
C	-1.969688	-3.559100	-1.709547
H	-1.690469	-4.574136	-2.048759
N	4.862892	-0.129952	-0.076523
C	4.450529	1.005139	-0.887554
C	4.098306	2.265656	-0.103872
C	3.566838	2.200031	1.190090
C	3.253596	3.367672	1.889058
C	3.463659	4.617766	1.302195
C	3.989036	4.692618	0.009990
C	4.308133	3.523944	-0.682649
O	-1.277869	-0.472578	1.371687
C	-0.306026	-0.152379	2.364879
O	-1.747826	2.538180	-2.301773
C	-1.542965	2.847945	-3.671107
C	-2.625835	-4.401085	0.461281
C	-2.680480	-4.251467	1.969077
C	-2.649159	-5.390286	2.783545
C	-2.785990	-5.287192	4.169508
C	-2.953099	-4.034915	4.761598
C	-2.982773	-2.892699	3.957046
C	-2.850141	-2.997564	2.571589

C	6.202137	-0.033721	0.482947
C	7.342444	-0.461181	-0.438184
C	8.651766	-0.061898	-0.134777
C	9.722853	-0.456419	-0.935443
C	9.498839	-1.253261	-2.061670
C	8.198705	-1.649428	-2.375702
C	7.127408	-1.256435	-1.568529
H	-0.226779	0.744980	-3.613760
H	0.677527	-1.309128	-2.626834
H	-3.552481	3.508469	-1.053253
H	-5.199383	2.997244	1.482871
H	-4.316808	4.476109	1.129452
H	-6.738027	4.742007	0.612878
H	-5.754379	4.932802	-0.835757
H	-7.308649	2.416153	-0.026721
H	-7.712506	3.501299	-1.354821
H	-6.519315	1.435894	-2.165367
H	-5.615140	2.899098	-2.537831
H	-5.068468	0.994239	-0.194267
H	-4.091730	1.159456	-1.650701
H	-0.337028	3.165358	0.997351
H	-2.550593	4.526314	2.616950
H	-1.455622	3.287898	3.205715
H	-0.707899	5.582075	3.886832
H	0.492421	4.785505	2.870877
H	-1.530361	6.895417	1.944623
H	0.205121	7.135043	2.125113
H	-0.434737	6.723513	-0.276247
H	0.660771	5.482558	0.328015
H	-2.380374	5.240991	0.055752
H	-1.188304	4.434625	-0.955281
H	-0.633949	-0.650084	3.279842
H	0.687190	-0.521415	2.086738
H	-0.263032	0.931872	2.534585
H	-2.069321	3.788472	-3.844179
H	-0.478011	2.982470	-3.901216
H	-1.959498	2.072114	-4.326099
H	2.013168	-5.518385	1.343151
H	-0.379441	-5.453028	0.845293
H	2.195560	-0.253193	-0.393050
H	4.230943	-4.441281	1.303971
H	5.660226	-2.528920	0.812770
H	-2.362768	-5.444510	0.205863
H	-3.635372	-4.251493	0.063079
C	-3.378603	-3.253957	-2.182697
H	-1.276157	-2.866881	-2.192549
H	-2.853354	-2.104526	1.956970
H	-3.116830	-1.912642	4.408625
H	-3.058551	-3.948985	5.840123
H	-2.758053	-6.183908	4.783539
H	-2.519706	-6.370496	2.328139

H 6.365516 1.009596 0.784188
H 6.239553 -0.611217 1.413390
H 8.832454 0.565658 0.736253
H 10.731270 -0.136991 -0.685185
H 10.331519 -1.558557 -2.689659
H 8.013796 -2.266830 -3.251052
H 6.116757 -1.566666 -1.816684
H 5.267326 1.243436 -1.583459
H 3.607138 0.697382 -1.513914
H 4.734066 3.589071 -1.682173
H 4.164366 5.660531 -0.452375
H 3.228265 5.525905 1.850370
H 2.851931 3.300876 2.896904
H 3.410704 1.229886 1.652934
C -4.065587 -2.117166 -1.733974
C -5.345204 -1.830398 -2.209837
C -5.953993 -2.666509 -3.150027
C -5.276031 -3.797217 -3.606179
C -4.000161 -4.089546 -3.118481
H -3.593926 -1.469278 -1.001184
H -5.869589 -0.952030 -1.843972
H -6.950863 -2.439788 -3.519178
H -5.741717 -4.458506 -4.332535
H -3.480363 -4.979579 -3.467254

Conformer **J**: B3LYP/6-31G(d) [5d]

Processing: namphos5dbe.log
PG=C01

Method	BasisSet	Imaginary Freqs
RB3LYP	6-31G(d)	0

HF Energy
-2849.3557236

ZPE	E298	S298	Squasihar	Equasihar	Strans	Srot
678.02005	714.125	337.717	285.838	714.334	46.099	40.652

Processing: namphos5dbe.log
126
C -3.075884 -2.731290 1.102888
C -3.658502 -2.736288 -0.169831
C -3.680344 -3.935185 -0.895223
C -3.125366 -5.101193 -0.367727
C -2.546633 -5.087098 0.904130
C -2.524445 -3.898698 1.636758
C -4.255763 -1.474793 -0.787896
N -4.701087 -0.460269 0.153042
C -5.974169 -0.728114 0.803318

C	-7.220265	-0.357675	0.002827
C	-8.451192	-0.934816	0.344904
C	-9.615741	-0.594316	-0.342339
C	-9.565051	0.326439	-1.392490
C	-8.343276	0.900140	-1.745357
C	-7.178519	0.560617	-1.051647
C	-3.930326	0.665721	0.455884
C	-4.542086	1.799590	1.077451
C	-3.794674	2.907987	1.386586
C	-2.412059	2.990179	1.082546
C	-1.783120	1.883990	0.424047
C	-2.571172	0.729439	0.153553
C	-1.636485	4.124758	1.409457
C	-0.302596	4.181621	1.093257
C	0.352556	3.105999	0.425326
C	-0.381021	1.951099	0.104793
C	0.244456	0.779385	-0.587628
C	1.153275	-0.087756	0.058120
C	1.768953	-1.179953	-0.592011
C	1.426372	-1.396552	-1.949075
C	0.504136	-0.573122	-2.602377
C	-0.072990	0.495126	-1.918538
P	2.978677	-2.178599	0.431148
C	4.436382	-2.494413	-0.730625
C	4.977626	-1.160908	-1.287613
C	6.235181	-1.370771	-2.148455
C	7.334002	-2.117451	-1.378696
C	6.806354	-3.442028	-0.809538
C	5.550405	-3.225241	0.052383
O	1.482831	0.182683	1.365581
C	0.595889	-0.336343	2.354056
O	2.040114	-2.443368	-2.580450
C	1.759597	-2.679604	-3.950830
N	1.735005	3.222999	0.103343
C	2.108275	3.208806	-1.327073
C	1.915117	4.527562	-2.065320
C	3.016129	5.314259	-2.427276
C	2.846460	6.525854	-3.101616
C	1.563815	6.971678	-3.422455
C	0.455838	6.197004	-3.068268
C	0.630658	4.985806	-2.398652
C	2.084341	-3.861095	0.605058
C	2.277040	-4.923464	-0.495251
C	1.458059	-6.195116	-0.205020
C	1.789743	-6.784560	1.173039
C	1.605901	-5.735136	2.278536
C	2.416596	-4.459963	1.992259
C	2.549657	4.178090	0.864166
C	2.613052	3.942390	2.362807
C	2.529397	5.027654	3.243215
C	2.673554	4.847666	4.620771

C	2.902711	3.571454	5.135774
C	2.986556	2.482207	4.263971
C	2.844852	2.663769	2.887506
H	0.236217	-0.745577	-3.637863
H	-0.780455	1.136339	-2.438268
H	4.128948	-3.119372	-1.574994
H	5.816460	-2.631827	0.939214
H	5.190640	-4.192447	0.424972
H	7.584659	-3.939523	-0.215902
H	6.563565	-4.122460	-1.639474
H	7.688384	-1.485233	-0.550994
H	8.200887	-2.298233	-2.027729
H	6.610140	-0.401592	-2.503501
H	5.965634	-1.948671	-3.045282
H	5.220657	-0.489789	-0.450097
H	4.205272	-0.655424	-1.877704
H	1.019929	-3.582199	0.615944
H	3.489757	-4.697522	2.040866
H	2.235615	-3.713913	2.775665
H	1.892719	-6.151044	3.253578
H	0.539810	-5.471824	2.351204
H	2.833282	-7.133854	1.175980
H	1.165866	-7.665619	1.374148
H	1.638395	-6.940880	-0.991279
H	0.386561	-5.949308	-0.245890
H	3.339719	-5.197928	-0.560134
H	1.998070	-4.510523	-1.467322
H	0.915191	0.095494	3.304848
H	-0.440902	-0.044549	2.152412
H	0.667724	-1.431092	2.407208
H	2.370057	-3.538906	-4.234651
H	0.700578	-2.919585	-4.111954
H	2.033674	-1.817895	-4.572996
H	-2.114270	4.965487	1.907848
H	0.258328	5.073911	1.338516
H	-2.079199	-0.132539	-0.276857
H	-4.272488	3.761046	1.863554
H	-5.607310	1.802422	1.279462
H	3.565771	4.075987	0.459948
H	2.266851	5.226334	0.671194
H	3.163684	2.915329	-1.385296
H	1.536146	2.423627	-1.819645
H	4.019503	4.970276	-2.183721
H	3.715380	7.118826	-3.375772
H	1.427094	7.913409	-3.947597
H	-0.546601	6.534573	-3.319211
H	-0.236805	4.388974	-2.130411
H	2.352000	6.025864	2.846917
H	2.603335	5.702949	5.288173
H	3.014764	3.425778	6.207200
H	3.170355	1.485004	4.656794

H 2.890622 1.812818 2.216329
H -6.014488 -1.799712 1.042119
H -5.989143 -0.215363 1.771558
H -8.496398 -1.659166 1.156316
H -10.561351 -1.052357 -0.063774
H -10.470480 0.590007 -1.932572
H -8.293010 1.614716 -2.562980
H -6.228856 1.007774 -1.330143
H -5.116044 -1.762983 -1.408009
H -3.535137 -1.016322 -1.474093
H -4.142534 -3.956356 -1.880581
H -3.156097 -6.023170 -0.942640
H -2.125827 -5.996839 1.323648
H -2.082466 -3.880042 2.629718
H -3.061078 -1.810036 1.677694

Conformer **K**: B3LYPD3/6-31+G(d,p)

Processing: namphos-e6pbed3.log
Method Basis Set Imaginary Freqs
RB3LYP 6-31+G(d,p)

HF
-2849.74264110

Processing: namphos-e6pbed3.log
Frequency job incomplete: namphos-e6pbed3.log
126
C -6.844865 -1.360505 -1.378844
C -6.989198 -2.176486 -0.252684
C -8.265280 -2.652156 0.080988
C -9.375870 -2.321405 -0.697012
C -9.223555 -1.506012 -1.824066
C -7.955797 -1.027241 -2.160914
C -5.797349 -2.568163 0.616586
N -4.512424 -2.035260 0.204368
C -3.688965 -2.833437 -0.693055
C -2.412747 -3.380299 -0.062344
C -2.400308 -3.799683 1.273102
C -1.233783 -4.310313 1.845612
C -0.061194 -4.398567 1.089426
C -0.060888 -3.967411 -0.239329
C -1.232001 -3.462732 -0.809452
C -4.114523 -0.739937 0.534559
C -2.852078 -0.270097 0.181810
C -2.406385 1.032386 0.522001
C -3.279512 1.887855 1.266638
C -4.574649 1.405236 1.592829
C -4.993053 0.144114 1.238933
C -1.087533 1.483237 0.187520

C	-0.663122	2.754073	0.584994
C	-1.548594	3.586741	1.325095
C	-2.815950	3.167213	1.655629
C	-0.172755	0.587813	-0.578691
C	0.982671	0.048546	0.019848
C	1.960555	-0.664387	-0.703216
C	1.668279	-0.953452	-2.057333
C	0.492215	-0.490903	-2.659540
C	-0.400697	0.288709	-1.922382
P	3.562376	-0.973285	0.210902
C	3.465348	-2.831729	0.590070
C	3.825763	-3.811804	-0.542617
C	3.654424	-5.278247	-0.107505
C	4.454620	-5.588591	1.165807
C	4.084804	-4.620689	2.299442
C	4.265646	-3.155008	1.871414
O	1.195625	0.272366	1.355303
C	0.451610	-0.563234	2.249435
O	2.585607	-1.711708	-2.735413
C	2.409786	-1.942614	-4.126452
N	0.636837	3.217817	0.214284
C	1.292445	4.183000	1.107958
C	1.550964	3.632098	2.495151
C	2.420769	2.545697	2.660101
C	2.679936	2.030319	3.929783
C	2.077241	2.600387	5.057304
C	1.215310	3.688972	4.904084
C	0.956273	4.199274	3.627629
C	4.881086	-0.817470	-1.127089
C	6.275858	-1.013262	-0.494927
C	7.394490	-0.827057	-1.534457
C	7.301994	0.543253	-2.221325
C	5.913240	0.755293	-2.841727
C	4.796380	0.567051	-1.802208
C	0.795046	3.565241	-1.215476
C	0.056783	4.825368	-1.629571
C	0.707844	6.065518	-1.675182
C	0.008250	7.232568	-1.996639
C	-1.360018	7.172622	-2.275076
C	-2.020990	5.940512	-2.233374
C	-1.316959	4.777959	-1.913853
H	0.271155	-0.710279	-3.696738
H	-1.292932	0.678700	-2.404652
H	4.738212	-1.579200	-1.897718
H	6.412658	-0.289056	0.320944
H	6.358736	-2.009059	-0.045053
H	8.374053	-0.949657	-1.055351
H	7.313686	-1.618117	-2.294589
H	7.486426	1.331343	-1.476596
H	8.083138	0.642076	-2.985452
H	5.843263	1.754367	-3.290530

H	5.767959	0.031819	-3.657754
H	4.880354	1.350844	-1.034781
H	3.819640	0.699259	-2.278806
H	2.402628	-2.968818	0.830016
H	5.334084	-2.964973	1.698833
H	3.959316	-2.482065	2.680656
H	4.687890	-4.828088	3.192289
H	3.035492	-4.784055	2.586569
H	5.528604	-5.491862	0.948259
H	4.288781	-6.626968	1.478738
H	3.960912	-5.945717	-0.923196
H	2.590739	-5.482963	0.076862
H	4.872451	-3.657222	-0.837728
H	3.220660	-3.600471	-1.428237
H	0.708888	-0.221602	3.253630
H	-0.626888	-0.460015	2.089052
H	0.735089	-1.616178	2.124116
H	3.283224	-2.515624	-4.442292
H	1.502423	-2.526799	-4.327904
H	2.369923	-1.000188	-4.687378
H	-3.479130	3.824135	2.213417
H	-1.229046	4.581094	1.608478
H	-2.154602	-0.916444	-0.326347
H	-5.254151	2.060285	2.132765
H	-6.003528	-0.162004	1.480489
H	2.255847	4.414528	0.638838
H	0.755345	5.143134	1.174476
H	1.868518	3.676127	-1.405493
H	0.452911	2.718134	-1.811221
H	1.772655	6.116737	-1.459382
H	0.530183	8.184970	-2.030215
H	-1.906633	8.077480	-2.525191
H	-3.084383	5.885483	-2.449408
H	-1.835728	3.824310	-1.871691
H	0.280946	5.043549	3.513474
H	0.742130	4.138484	5.772717
H	2.279275	2.199388	6.046898
H	3.350405	1.181925	4.035177
H	2.867741	2.079867	1.789168
H	-5.995739	-2.281109	1.656967
H	-5.715460	-3.661074	0.629693
H	-8.389797	-3.285964	0.957043
H	-10.358064	-2.698037	-0.425411
H	-10.086088	-1.246049	-2.430738
H	-7.828208	-0.389130	-3.030816
H	-5.863005	-0.976943	-1.636940
H	-4.303905	-3.666922	-1.048250
H	-3.427964	-2.247276	-1.585920
H	-1.215283	-3.096128	-1.833026
H	0.851856	-3.999819	-0.825683
H	0.847826	-4.785349	1.538441

H -1.235966 -4.629384 2.884147
H -3.301951 -3.700281 1.870658

Conformer **L**: B3LYP/6-31G(d) [5d]

Processing: namphos-b5dbe.log
PG=C01

Method	BasisSet	Imaginary Freqs
RB3LYP	6-31G(d)	0

HF Energy
-2849.3548478

ZPE	E298	S298	Squasihar	Equasihar	Strans	Srot
677.99792	714.094	335.717	286.374	714.298	46.099	40.523

Processing: namphos-b5dbe.log
126
C 3.636620 4.665278 -0.369158
C 2.816858 3.642226 0.449956
C 3.751810 2.548865 1.006615
C 4.903265 3.153374 1.829631
C 5.702748 4.187451 1.024188
C 4.781642 5.276309 0.456187
P 1.477358 2.913705 -0.678363
C 0.003630 4.142357 -0.588104
C 0.126518 5.352949 0.357197
C -1.130759 6.240764 0.307158
C -1.439092 6.713399 -1.120308
C -1.554508 5.521766 -2.081239
C -0.307402 4.623972 -2.024168
C 0.830537 1.455330 0.292132
C 0.782529 0.204119 -0.355411
C 0.352943 -0.975015 0.294234
C -0.046753 -0.862537 1.628376
C -0.034823 0.356729 2.303599
C 0.398429 1.506832 1.638991
C 0.285255 -2.292483 -0.410022
C 1.444989 -2.966878 -0.815553
C 1.326828 -4.204227 -1.516688
C 0.102010 -4.751520 -1.803119
C -1.090217 -4.110123 -1.393812
C -1.007560 -2.869593 -0.679792
C -2.222455 -2.234368 -0.302116
C -3.474420 -2.788423 -0.567010
C -3.526806 -4.034176 -1.264304
C -2.368631 -4.655524 -1.665135
N 2.736705 -2.432552 -0.554732
C 3.185546 -2.371505 0.850576

C	3.642284	-3.697754	1.444721
C	5.005084	-3.968394	1.627309
C	5.431775	-5.185253	2.165310
C	4.496117	-6.154310	2.529437
C	3.133554	-5.896833	2.355713
C	2.711951	-4.679383	1.820581
N	-4.647330	-2.131251	-0.147810
C	-4.588675	-1.362014	1.097757
C	-4.427706	0.147376	0.955898
C	-4.093871	0.759102	-0.255317
C	-3.941583	2.146389	-0.333864
C	-4.120895	2.940142	0.799410
C	-4.451667	2.336535	2.016218
C	-4.602825	0.952618	2.090394
O	1.201753	0.104899	-1.664868
C	0.175419	0.241803	-2.645147
O	0.437872	2.733198	2.242134
C	-0.068788	2.859084	3.561655
C	3.795048	-2.774842	-1.510164
C	4.891590	-1.725794	-1.602266
C	4.577416	-0.387018	-1.879426
C	5.594138	0.555125	-2.040482
C	6.935833	0.178293	-1.921518
C	7.255524	-1.150132	-1.641320
C	6.236736	-2.094353	-1.484043
C	-5.943517	-2.739901	-0.473480
C	-7.128259	-1.802779	-0.331069
C	-7.253674	-0.673711	-1.152847
C	-8.365844	0.160101	-1.050714
C	-9.375012	-0.124077	-0.125957
C	-9.263149	-1.246462	0.694260
C	-8.145194	-2.078509	0.590115
H	-0.357897	0.394198	3.336947
H	-0.380427	-1.754024	2.153220
H	2.356034	4.156718	1.299926
H	4.055316	4.160384	-1.251944
H	2.989658	5.464162	-0.753730
H	5.355349	5.978457	-0.163295
H	4.359043	5.863907	1.284983
H	6.215526	3.679159	0.194143
H	6.486528	4.636292	1.648517
H	5.565504	2.352324	2.183382
H	4.489818	3.637075	2.727629
H	4.170836	1.960856	0.177806
H	3.185726	1.847447	1.628436
H	-0.846714	3.537826	-0.243478
H	0.558794	5.180440	-2.413035
H	-0.434810	3.762386	-2.691739
H	-1.718053	5.872926	-3.108724
H	-2.439882	4.925920	-1.811160
H	-0.630727	7.376594	-1.462932

H	-2.360542	7.310202	-1.136724
H	-1.004115	7.103524	0.975114
H	-1.988766	5.670089	0.693718
H	0.994347	5.963844	0.071587
H	0.298295	5.012622	1.381303
H	0.648918	0.056377	-3.611889
H	-0.627152	-0.487753	-2.483725
H	-0.241817	1.257290	-2.637367
H	0.021606	3.917017	3.815629
H	-1.123587	2.560351	3.614384
H	0.514108	2.265629	4.277985
H	0.036578	-5.700477	-2.331134
H	2.226915	-4.737211	-1.800772
H	-2.165669	-1.273343	0.191030
H	-2.426036	-5.605143	-2.192901
H	-4.473721	-4.519496	-1.464808
H	3.319000	-2.861081	-2.492558
H	4.258690	-3.752046	-1.292708
H	4.008045	-1.650160	0.896715
H	2.367817	-1.959077	1.444082
H	5.737654	-3.213862	1.349512
H	6.493818	-5.373579	2.301835
H	4.824164	-7.101461	2.949886
H	2.397672	-6.643480	2.643720
H	1.650014	-4.486195	1.694177
H	6.489202	-3.131347	-1.272418
H	8.294820	-1.454455	-1.545422
H	7.724516	0.915572	-2.048535
H	5.336759	1.586724	-2.267310
H	3.535042	-0.092621	-1.966200
H	-6.127523	-3.642578	0.135258
H	-5.896673	-3.067163	-1.516701
H	-6.471257	-0.448505	-1.872053
H	-8.447216	1.032080	-1.694157
H	-10.242429	0.525885	-0.047933
H	-10.043260	-1.477131	1.414961
H	-8.063779	-2.955541	1.228820
H	-5.506411	-1.555088	1.664889
H	-3.763730	-1.751068	1.710514
H	-4.870355	0.490511	3.039269
H	-4.603459	2.945730	2.903905
H	-4.011840	4.019464	0.735448
H	-3.686398	2.606587	-1.285193
H	-3.958104	0.141200	-1.137465

Conformer **M**: B3LYPD3/6-31+G(d,p)

Processing: namphos-c6pbed3.log
PG=C01

Method	BasisSet	Imaginary Freqs
RB3LYP	6-31+G(d,p)	0

HF Energy
-2849.7459130

ZPE	E298	S298	Squasihar	Equasihar	Strans	Srot
676.38169	712.094	328.177	283.952	712.282	46.099	40.332

Processing: namphos-c6pbed3.log
126

C	-4.138969	-3.722487	-1.020010
C	-3.226800	-2.985514	-0.015374
C	-3.910422	-1.689848	0.472104
C	-5.308345	-1.968677	1.048992
C	-6.202758	-2.709305	0.044950
C	-5.530422	-4.005140	-0.430602
P	-1.566009	-2.624039	-0.859901
C	-0.459392	-4.148565	-0.526869
C	-1.140198	-5.396816	0.060268
C	-0.126946	-6.534534	0.277323
C	0.613909	-6.890385	-1.019882
C	1.289074	-5.651674	-1.626581
C	0.282152	-4.507836	-1.832314
C	-0.832111	-1.400166	0.333820
C	-0.478908	-0.126437	-0.141353
C	-0.057723	0.912044	0.712856
C	0.049037	0.625592	2.075246
C	-0.236662	-0.641350	2.585072
C	-0.677750	-1.645186	1.718243
C	0.316135	2.242517	0.156209
C	-0.634723	3.072986	-0.446676
C	-0.203177	4.289548	-1.059530
C	1.127524	4.634529	-1.111510
C	2.117395	3.802392	-0.533129
C	1.703000	2.608089	0.138446
C	2.698715	1.762399	0.689357
C	4.058600	2.034439	0.581119
C	4.456494	3.223937	-0.105088
C	3.507137	4.073785	-0.627261
N	-1.995930	2.698860	-0.497093
C	-2.744200	2.499138	0.752709
C	-3.379325	3.769798	1.294816
C	-4.770178	3.892784	1.395496
C	-5.352824	5.069109	1.879354
C	-4.545664	6.143013	2.261997
C	-3.153925	6.031895	2.161112
C	-2.576936	4.854414	1.682508
N	4.996994	1.149295	1.131836
C	4.590797	0.323324	2.266067
C	4.005807	-1.050877	1.954225

C	3.566734	-1.419367	0.677787
C	2.968288	-2.664657	0.462751
C	2.810130	-3.562585	1.519720
C	3.254607	-3.207569	2.797022
C	3.845313	-1.960853	3.009436
O	-0.558707	0.136220	-1.493532
C	0.659402	-0.089065	-2.209911
O	-1.001316	-2.905161	2.143471
C	-0.732330	-3.266664	3.492423
C	-2.804645	3.196265	-1.611814
C	-3.931384	2.245248	-1.974054
C	-3.636729	0.933854	-2.375347
C	-4.662097	0.065598	-2.752178
C	-5.995627	0.489018	-2.720394
C	-6.296515	1.790449	-2.311585
C	-5.266465	2.663113	-1.942272
C	6.426677	1.392063	0.950395
C	6.928303	1.201679	-0.473315
C	7.830689	2.111717	-1.035147
C	8.329806	1.919357	-2.327030
C	7.925449	0.810454	-3.073967
C	7.024960	-0.104785	-2.518341
C	6.532211	0.089483	-1.226961
H	-0.121916	-0.825138	3.645949
H	0.376715	1.410351	2.751466
H	-3.065623	-3.626149	0.857704
H	-4.250401	-3.102236	-1.920141
H	-3.675754	-4.660417	-1.347810
H	-6.155807	-4.511683	-1.176798
H	-5.430537	-4.695987	0.419637
H	-6.383247	-2.056812	-0.821082
H	-7.181973	-2.925776	0.490242
H	-5.776023	-1.023131	1.351469
H	-5.207886	-2.577827	1.959786
H	-4.000435	-0.982684	-0.360904
H	-3.295451	-1.198912	1.232095
H	0.283412	-3.813158	0.203609
H	-0.452363	-4.801222	-2.596313
H	0.795239	-3.623174	-2.231025
H	1.769454	-5.905724	-2.579809
H	2.090204	-5.313423	-0.953934
H	-0.107410	-7.297451	-1.743546
H	1.354216	-7.678716	-0.835113
H	-0.638096	-7.418458	0.679914
H	0.604092	-6.222099	1.037870
H	-1.925958	-5.752109	-0.620041
H	-1.623743	-5.148956	1.008898
H	0.464941	0.201365	-3.244129
H	1.473332	0.521657	-1.801428
H	0.938726	-1.150389	-2.175859
H	-0.996297	-4.322715	3.572000

H	0.330334	-3.135205	3.731621
H	-1.345273	-2.687206	4.194665
H	1.430645	5.559244	-1.597052
H	-0.942694	4.960780	-1.480822
H	2.373973	0.851405	1.166360
H	3.827411	4.976419	-1.142268
H	5.502755	3.468269	-0.229169
H	-2.143303	3.289703	-2.477883
H	-3.224119	4.195492	-1.408887
H	-3.519050	1.746624	0.571770
H	-2.067141	2.078163	1.494918
H	-5.400505	3.063675	1.084202
H	-6.434265	5.146986	1.951847
H	-4.994702	7.059037	2.635331
H	-2.519089	6.862225	2.458194
H	-1.496693	4.774416	1.600316
H	-5.501929	3.674327	-1.620107
H	-7.329041	2.127412	-2.277998
H	-6.792197	-0.191494	-3.008394
H	-4.417458	-0.946818	-3.059981
H	-2.605369	0.594499	-2.364564
H	6.711388	2.394863	1.307856
H	6.952508	0.678682	1.591663
H	8.140650	2.983398	-0.462444
H	9.025280	2.638629	-2.750244
H	8.305668	0.661055	-4.080342
H	6.707197	-0.971377	-3.091428
H	5.833258	-0.620329	-0.797218
H	5.460116	0.186138	2.918043
H	3.852631	0.878945	2.863303
H	4.183692	-1.689093	4.007578
H	3.144965	-3.902575	3.625226
H	2.342271	-4.527286	1.352334
H	2.613987	-2.926167	-0.529801
H	3.670384	-0.718949	-0.143481

Conformer **N**: B3LYPD3/6-31+G(d,p)

Processing: namphos-f6pbed3.log
PG=C01

Method	BasisSet	Imaginary Freqs
RB3LYP	6-31+G(d,p)	0

HF Energy
-2849.7417005

ZPE	E298	S298	Squasihar	Equasihar	Strans	Srot
675.79827	711.634	326.542	284.986	711.832	46.099	40.534

Processing: namphos-f6pbed3.log
126

C	-7.311688	-2.384395	0.742818
C	-7.560197	-1.417108	-0.236922
C	-8.879021	-1.213642	-0.667364
C	-9.927768	-1.966287	-0.136080
C	-9.670718	-2.933341	0.841926
C	-8.360438	-3.138103	1.279793
C	-6.435809	-0.590370	-0.854503
N	-5.157986	-0.650431	-0.169310
C	-5.016598	0.150186	1.037004
C	-4.265376	1.471752	0.903859
C	-3.664313	1.879255	-0.290823
C	-2.942114	3.075975	-0.350482
C	-2.821721	3.882181	0.782832
C	-3.426970	3.485451	1.979639
C	-4.140590	2.287960	2.037404
C	-4.177736	-1.586065	-0.510244
C	-2.861864	-1.396560	-0.101926
C	-1.831066	-2.314652	-0.419972
C	-2.159828	-3.483987	-1.175715
C	-3.509264	-3.667858	-1.577685
C	-4.493543	-2.755443	-1.268294
C	-0.458825	-2.041588	-0.086223
C	0.532775	-2.959197	-0.428756
C	0.175790	-4.139222	-1.146161
C	-1.121927	-4.387950	-1.522724
C	-0.133732	-0.697096	0.473647
C	0.446750	0.280554	-0.357407
C	0.786955	1.565039	0.100669
C	0.505150	1.863973	1.453235
C	-0.105680	0.923607	2.290287
C	-0.412555	-0.342707	1.793473
P	1.636791	2.714578	-1.095590
C	0.704064	4.350549	-0.784430
C	1.451807	5.485325	-0.062543
C	0.533609	6.702123	0.147355
C	-0.037755	7.212119	-1.184098
C	-0.759860	6.089468	-1.944240
C	0.144194	4.859271	-2.130891
O	0.746672	-0.069491	-1.658474
C	-0.307900	0.152431	-2.602154
O	0.877613	3.107419	1.885327
C	0.479367	3.536929	3.181387
N	1.920184	-2.803647	-0.128406
C	2.757130	-2.369624	-1.269589
C	4.107367	-3.053494	-1.358103
C	4.198897	-4.404147	-1.719596
C	5.436757	-5.043483	-1.792079
C	6.608291	-4.335818	-1.500403
C	6.529582	-2.986868	-1.147465

C	5.286094	-2.352283	-1.083227
C	3.324607	2.886025	-0.254934
C	4.293468	3.642238	-1.188579
C	5.702272	3.749396	-0.581149
C	6.270503	2.362366	-0.247071
C	5.317007	1.586535	0.672367
C	3.902578	1.493873	0.078113
C	2.287266	-2.223731	1.164955
C	3.586530	-2.757589	1.742895
C	4.483722	-1.877305	2.357673
C	5.672420	-2.346216	2.925045
C	5.977249	-3.708217	2.878237
C	5.082271	-4.595413	2.269175
C	3.894558	-4.122966	1.711178
H	-0.333004	1.157356	3.322751
H	-0.869762	-1.077353	2.450342
H	3.214369	3.442608	0.683257
H	4.353948	3.104440	-2.145609
H	3.912005	4.642847	-1.419336
H	6.368061	4.279211	-1.273926
H	5.655720	4.353333	0.337008
H	6.409413	1.796889	-1.180147
H	7.260208	2.453704	0.217353
H	5.704393	0.578096	0.864165
H	5.262909	2.093213	1.647279
H	3.932659	0.886038	-0.837791
H	3.244446	0.969308	0.776950
H	-0.145660	4.069952	-0.153866
H	0.980181	5.116799	-2.797608
H	-0.410106	4.058263	-2.636407
H	-1.108563	6.451651	-2.919499
H	-1.657721	5.794537	-1.382420
H	0.787864	7.592092	-1.803472
H	-0.717261	8.056245	-1.012713
H	1.082861	7.503477	0.658154
H	-0.294754	6.415292	0.812548
H	2.317492	5.802110	-0.658804
H	1.834166	5.135215	0.899022
H	0.058411	-0.219595	-3.560776
H	-1.213424	-0.393040	-2.313751
H	-0.527278	1.224412	-2.687338
H	0.813074	4.572901	3.263259
H	-0.611027	3.493108	3.294172
H	0.957205	2.939797	3.968502
H	-1.368079	-5.287783	-2.081386
H	0.965902	-4.844797	-1.381461
H	-2.596606	-0.496908	0.432672
H	-3.767768	-4.560153	-2.142865
H	-5.516120	-2.950911	-1.570754
H	2.893456	-1.280327	-1.236783
H	2.188257	-2.572816	-2.179877

H 2.354526 -1.127579 1.121298
H 1.478019 -2.456763 1.863557
H 4.258597 -0.813751 2.377856
H 6.362629 -1.647194 3.389762
H 6.906000 -4.076253 3.305085
H 5.317521 -5.654871 2.218839
H 3.212454 -4.802228 1.209800
H 5.227359 -1.307172 -0.791731
H 7.432575 -2.430546 -0.912315
H 7.572957 -4.833151 -1.547862
H 5.490030 -6.091866 -2.073115
H 3.289609 -4.959671 -1.933872
H -6.288741 -0.891925 -1.897988
H -6.749707 0.460894 -0.894581
H -9.086027 -0.458098 -1.422783
H -10.944068 -1.795902 -0.480169
H -10.485116 -3.518386 1.259124
H -8.150426 -3.886127 2.039092
H -6.294395 -2.550192 1.082951
H -6.022522 0.350973 1.425563
H -4.514699 -0.455810 1.803936
H -4.602129 1.979205 2.973468
H -3.343405 4.109188 2.865682
H -2.258353 4.808650 0.737658
H -2.463184 3.371679 -1.279236
H -3.745971 1.243000 -1.166636

Conformer **O**: B3LYP/6-31G(d) [5d]

Processing: namphos-g5dbe.log
PG=C01

Method BasisSet Imaginary Freqs
RB3LYP 6-31G(d) 0

HF Energy
-2849.3483494

ZPE E298 S298 Squasihar Equasihar Strans Srot
677.86646 713.968 337.224 285.928 714.176 46.099 40.590
ccl00:/aue/chem126/aue/ark/pj/nam> gtg namphos-g5dbe.log
Processing: namphos-g5dbe.log
126
C -5.766524 -1.854708 -1.841419
C -6.004270 -2.917661 -0.961847
C -6.719553 -4.028529 -1.425353
C -7.191327 -4.079245 -2.738503
C -6.947121 -3.016188 -3.609152
C -6.232761 -1.904701 -3.155451
C -5.568887 -2.853432 0.494584

N	-4.284499	-2.202210	0.744478
C	-4.265302	-1.370285	1.952945
C	-4.872577	0.010395	1.767057
C	-4.454685	0.842850	0.718419
C	-4.992718	2.120434	0.567870
C	-5.957865	2.587793	1.465533
C	-6.379995	1.768218	2.512158
C	-5.840451	0.487127	2.657778
C	-3.092584	-2.781875	0.277156
C	-1.872225	-2.119178	0.397162
C	-0.649581	-2.687155	-0.047548
C	-0.684187	-3.972095	-0.680938
C	-1.936509	-4.621125	-0.821005
C	-3.102314	-4.061002	-0.358223
C	0.610472	-2.001633	0.084982
C	1.782023	-2.626476	-0.337745
C	1.719547	-3.897092	-0.978298
C	0.521002	-4.543316	-1.155206
C	0.628524	-0.621490	0.666696
C	0.643242	0.531776	-0.149487
C	0.549640	1.839641	0.375429
C	0.564513	1.973880	1.785013
C	0.555043	0.847559	2.613597
C	0.563002	-0.426693	2.047841
P	0.211160	3.181695	-0.881707
C	-0.882349	4.448308	0.038444
C	-0.232476	5.735090	0.582499
C	-1.262556	6.630544	1.295689
C	-2.436980	6.986901	0.373603
C	-3.086723	5.722322	-0.206048
C	-2.055373	4.817205	-0.901247
O	0.822287	0.401479	-1.510382
C	-0.324323	0.059923	-2.297960
O	0.580676	3.247564	2.283774
C	0.567183	3.433368	3.689858
N	3.049207	-2.001279	-0.087453
C	4.026173	-2.115010	-1.174126
C	5.188973	-1.144625	-1.041582
C	5.010673	0.151023	-0.539087
C	6.095523	1.021779	-0.424641
C	7.370322	0.622590	-0.835228
C	7.554578	-0.659988	-1.352798
C	6.471779	-1.537598	-1.444317
C	1.858574	4.059745	-1.213969
C	2.441273	3.568806	-2.558651
C	3.669952	4.397291	-2.969264
C	4.749129	4.393075	-1.877145
C	4.178498	4.834686	-0.520347
C	2.938663	4.013670	-0.118085
C	3.589068	-2.264077	1.269895
C	4.064506	-3.683195	1.546445

C	5.404084	-4.043739	1.339434
C	5.842601	-5.347495	1.581506
C	4.945824	-6.313650	2.041101
C	3.610364	-5.966821	2.259070
C	3.176303	-4.663424	2.013748
H	0.543461	0.947723	3.692294
H	0.529082	-1.295039	2.701001
H	1.564664	5.110234	-1.358141
H	2.717583	2.508969	-2.469370
H	1.673052	3.621846	-3.339552
H	4.083644	4.014655	-3.911596
H	3.354123	5.433579	-3.165029
H	5.157826	3.377585	-1.786131
H	5.586312	5.042663	-2.165407
H	4.948428	4.755777	0.259828
H	3.901372	5.898521	-0.573022
H	3.230330	2.966710	0.045910
H	2.539863	4.374382	0.835087
H	-1.301625	3.899326	0.890014
H	-1.661975	5.329684	-1.792668
H	-2.541322	3.904694	-1.268683
H	-3.884499	5.992991	-0.910863
H	-3.565530	5.157100	0.607884
H	-2.068840	7.614841	-0.451582
H	-3.181373	7.586328	0.914001
H	-0.774217	7.544538	1.659953
H	-1.645157	6.104598	2.183463
H	0.203414	6.309494	-0.247660
H	0.584939	5.489081	1.264824
H	0.035324	-0.019418	-3.326375
H	-0.755423	-0.895262	-1.986644
H	-1.077412	0.853843	-2.240315
H	0.578527	4.513619	3.846759
H	-0.337827	3.010945	4.145528
H	1.451512	2.988764	4.164539
H	0.487814	-5.516260	-1.640751
H	2.638448	-4.369740	-1.306704
H	-1.839092	-1.126804	0.828930
H	-1.966059	-5.595435	-1.303866
H	-4.031901	-4.599388	-0.488888
H	3.481249	-1.903122	-2.102528
H	4.444150	-3.131191	-1.277434
H	4.417871	-1.567427	1.426338
H	2.804310	-1.991287	1.980067
H	6.110601	-3.292270	0.994428
H	6.885989	-5.605823	1.418331
H	5.285882	-7.327744	2.234709
H	2.906975	-6.710514	2.625112
H	2.136090	-4.400651	2.188979
H	6.624413	-2.543375	-1.830764
H	8.541576	-0.984045	-1.672979

H 8.212261 1.304088 -0.746537
H 5.946172 2.014527 -0.009054
H 4.018030 0.457365 -0.221882
H -5.600717 -3.866591 0.931406
H -6.314215 -2.264948 1.041919
H -6.908808 -4.862982 -0.752342
H -7.742690 -4.951048 -3.081199
H -7.307547 -3.054161 -4.633689
H -6.037773 -1.072699 -3.827276
H -5.213667 -0.989340 -1.489651
H -4.799325 -1.889532 2.764598
H -3.230214 -1.279286 2.289925
H -6.176303 -0.149943 3.473723
H -7.133941 2.119802 3.211707
H -6.379073 3.582223 1.344756
H -4.660272 2.753017 -0.250805
H -3.705556 0.483474 0.017619

Conformer **AA**: B3LYP/6-31G(d) [5d]

Processing: namphos-hd5dbe.log
PG=C01

Method	BasisSet	Imaginary Freqs
RB3LYP	6-31G(d)	0

HF Energy
-2849.3606312

ZPE	E298	S298	Squasihar	Equasihar	Strans	Srot
678.15117	714.222	335.956	285.550	714.431	46.099	40.561

Processing: namphos-hd5dbe.log
126
C 6.098012 -2.741874 -1.706897
C 4.798781 -2.224278 -1.767531
C 4.611406 -0.913609 -2.229044
C 5.705642 -0.149061 -2.635378
C 7.000082 -0.675165 -2.576531
C 7.194976 -1.974003 -2.106846
C 3.607350 -3.104191 -1.429111
N 2.661377 -2.484551 -0.496279
C 3.220042 -2.239945 0.848539
C 3.599667 -3.491699 1.629329
C 2.615280 -4.312500 2.201775
C 2.966094 -5.461961 2.910235
C 4.310754 -5.810652 3.063305
C 5.299910 -5.001503 2.503041
C 4.943863 -3.852769 1.791778
C 1.306443 -2.903910 -0.596828

C	0.246061	-2.067906	-0.220535
C	-1.112437	-2.527055	-0.372808
C	-1.366329	-3.833299	-0.904142
C	-0.271228	-4.649004	-1.267253
C	1.017477	-4.202531	-1.116346
C	-2.710253	-4.261071	-1.055622
C	-3.771776	-3.456002	-0.725312
C	-3.545065	-2.143931	-0.204480
C	-2.230736	-1.714111	-0.036668
C	0.447424	-0.703598	0.364838
C	0.819584	0.425943	-0.396030
C	0.866651	1.731231	0.146729
C	0.553455	1.873156	1.521370
C	0.207509	0.765205	2.300707
C	0.152272	-0.495730	1.715908
P	1.226346	3.115478	-1.061471
C	0.077913	4.525900	-0.545291
C	-1.394688	4.070673	-0.613362
C	-2.365951	5.226132	-0.317294
C	-2.127632	6.421605	-1.250717
C	-0.664069	6.881757	-1.193936
C	0.306055	5.725154	-1.492399
O	1.186874	0.239592	-1.712677
C	0.118406	0.206955	-2.661514
O	0.595120	3.136697	2.039534
C	0.212561	3.337630	3.391565
N	-4.626686	-1.314255	0.105738
C	-4.464894	0.120614	0.262017
C	-4.227129	0.626361	1.684114
C	-3.912679	-0.235041	2.739897
C	-3.690421	0.266101	4.025746
C	-3.778335	1.636817	4.272123
C	-4.092208	2.505889	3.222304
C	-4.317229	2.001829	1.941494
C	2.949373	3.754927	-0.593292
C	3.183283	4.371178	0.798749
C	4.616559	4.917241	0.934749
C	5.662414	3.829967	0.649578
C	5.434765	3.192112	-0.728389
C	3.998990	2.661734	-0.880861
C	-5.962942	-1.834718	0.347952
C	-6.942574	-1.722079	-0.817598
C	-8.319026	-1.809823	-0.566952
C	-9.240859	-1.745217	-1.611208
C	-8.798027	-1.582923	-2.926768
C	-7.430379	-1.486828	-3.185044
C	-6.508511	-1.556484	-2.137014
H	3.104186	4.551226	-1.339897
H	3.849845	2.257644	-1.888985
H	3.847320	1.822965	-0.187167
H	6.149594	2.376249	-0.894377

H	5.626841	3.943728	-1.509309
H	6.676243	4.246329	0.715127
H	5.592744	3.051295	1.423770
H	4.758070	5.750246	0.229395
H	4.763213	5.333579	1.940590
H	2.461514	5.172819	0.993841
H	3.012967	3.607797	1.566749
H	0.288704	4.843623	0.479884
H	-1.604979	3.673629	-1.618473
H	-1.570847	3.250243	0.091897
H	-3.403377	4.875287	-0.406190
H	-2.233863	5.549530	0.726082
H	-2.799396	7.250384	-0.991358
H	-2.374384	6.129074	-2.282264
H	-0.449876	7.284401	-0.192663
H	-0.494650	7.702915	-1.902864
H	1.338938	6.087037	-1.413124
H	0.170046	5.397611	-2.533771
H	-0.145114	-1.346537	2.323214
H	-0.039930	0.873760	3.349324
H	-0.632695	-0.541830	-2.387926
H	0.570863	-0.062999	-3.618411
H	-0.348436	1.195686	-2.748464
H	-0.821036	3.014457	3.569082
H	0.293470	4.412640	3.564375
H	0.882337	2.810890	4.083792
H	1.837636	-4.865745	-1.364411
H	-0.460735	-5.647986	-1.654279
H	-2.895974	-5.251013	-1.466731
H	-4.782445	-3.806007	-0.904107
H	-2.043746	-0.739212	0.390678
H	2.492212	-1.654898	1.412108
H	4.106218	-1.609368	0.721517
H	1.567131	-4.047431	2.091679
H	2.189506	-6.083509	3.349004
H	4.583890	-6.704223	3.618631
H	6.349006	-5.261622	2.620284
H	5.718439	-3.223400	1.359157
H	3.058327	-3.300082	-2.355969
H	3.978621	-4.077682	-1.066786
H	3.605702	-0.502122	-2.261711
H	5.548329	0.860151	-3.005839
H	7.848638	-0.075400	-2.895593
H	8.197082	-2.391963	-2.052671
H	6.250710	-3.758067	-1.349427
H	-6.383626	-1.305617	1.214194
H	-5.879115	-2.881512	0.661455
H	-8.671268	-1.927945	0.456365
H	-10.304582	-1.813986	-1.398343
H	-9.514678	-1.527530	-3.741784
H	-7.076375	-1.356589	-4.204486

H   -5.444806 -1.478858 -2.341447
H   -5.367860   0.603265 -0.135753
H   -3.643601   0.449961 -0.385532
H   -4.569918   2.684158   1.131794
H   -4.173329   3.574632   3.403771
H   -3.614564   2.025491   5.273885
H   -3.451533 -0.418648   4.835611
H   -3.837645 -1.301599   2.550794

Conformer **AB**: B3LYP/6-31G(d) [5d]

Processing: namphos-he5dbe.log
PG=C01

Method   BasisSet   Imaginary Freqs
RB3LYP   6-31G(d)   0

HF Energy
-2849.3629153

ZPE	E298	S298	Squasihar	Equasihar	Strans	Srot
678.10614	714.203	337.870	285.795	714.411	46.099	40.568

ccl00:/aue/chem126/aue/ark/pj/nam> gtg namphos-he5dbe.log
Processing: namphos-he5dbe.log
126
C   -6.684688 -1.012684 -2.088182
C   -7.112617 -1.216931 -0.772149
C   -8.488469 -1.220660 -0.503735
C   -9.416643 -1.036320 -1.527961
C   -8.980183 -0.836438 -2.840443
C   -7.612531 -0.823243 -3.115885
C   -6.128371 -1.460506   0.369155
N   -4.763942 -1.014542   0.135507
C   -4.504526   0.395376   0.367201
C   -4.190144   0.803506   1.806001
C   -3.945835 -0.135439   2.812870
C   -3.651565   0.277426   4.115673
C   -3.596666   1.636193   4.428071
C   -3.839636   2.582593   3.427383
C   -4.135964   2.166855   2.129553
C   -3.741095 -1.893834 -0.232727
C   -4.055256 -3.157916 -0.822583
C   -3.050361 -4.009700 -1.209004
C   -1.680229 -3.678902 -1.047676
C   -1.340823 -2.422975 -0.446793
C   -2.400936 -1.558279 -0.057045
C   -0.641835 -4.540633 -1.468680
C   0.674615 -4.189179 -1.301013
C   1.045930 -2.947184 -0.704357
C   0.044192 -2.065040 -0.278797

N	2.423303	-2.618834	-0.572343
C	2.988075	-2.536548	0.789251
C	3.281474	-3.876348	1.451928
C	2.244295	-4.669940	1.966460
C	2.514968	-5.900933	2.564539
C	3.831105	-6.360433	2.663013
C	4.872466	-5.579588	2.160037
C	4.596482	-4.348542	1.559544
C	0.354669	-0.746454	0.356804
C	0.847625	0.357255	-0.373484
C	1.047668	1.625561	0.210091
C	0.733546	1.767998	1.584208
C	0.260896	0.686928	2.332182
C	0.077466	-0.546842	1.712131
O	1.176494	0.162265	-1.698407
C	0.122455	0.376691	-2.635561
P	1.724150	2.990168	-0.872559
O	0.900271	3.014747	2.123432
C	0.532963	3.226594	3.478440
C	3.333218	-3.197100	-1.565676
C	4.569554	-2.350248	-1.820425
C	5.842993	-2.930631	-1.791478
C	6.980258	-2.179751	-2.102060
C	6.852376	-0.834508	-2.447765
C	5.583805	-0.246660	-2.476724
C	4.449211	-0.995216	-2.161925
C	0.516396	4.436870	-0.586875
C	-0.955080	3.984857	-0.484898
C	-1.910924	5.181475	-0.331789
C	-1.748284	6.198527	-1.469816
C	-0.285437	6.643248	-1.603993
C	0.655900	5.436735	-1.759572
C	3.245585	3.501539	0.144402
C	4.248797	2.329996	0.189597
C	5.527861	2.692657	0.961580
C	6.196373	3.948032	0.384168
C	5.208825	5.121734	0.335211
C	3.924577	4.760385	-0.433477
H	2.920831	3.723919	1.167407
H	3.239089	5.615074	-0.401471
H	4.170516	4.592113	-1.492851
H	5.680328	6.000907	-0.123762
H	4.942217	5.411437	1.362715
H	7.081885	4.218737	0.974113
H	6.552310	3.731890	-0.634300
H	5.277860	2.869156	2.018608
H	6.224483	1.844568	0.941656
H	3.781379	1.446775	0.639651
H	4.519739	2.044962	-0.836582
H	0.783039	4.942778	0.350689
H	-1.226497	3.425309	-1.393693

H	-1.091267	3.299766	0.356600
H	-2.949256	4.824689	-0.286009
H	-1.709687	5.678320	0.629143
H	-2.400751	7.065975	-1.304736
H	-2.072450	5.737893	-2.414967
H	0.003819	7.217405	-0.710940
H	-0.169652	7.320284	-2.460632
H	1.689723	5.785295	-1.858358
H	0.421869	4.913386	-2.698099
H	-0.310387	-1.377560	2.295647
H	0.013939	0.794372	3.381174
H	-0.759921	-0.223290	-2.383651
H	0.510229	0.064649	-3.607956
H	-0.149583	1.439089	-2.680824
H	-0.529041	3.006071	3.645398
H	0.721051	4.284062	3.674175
H	1.141288	2.619608	4.161617
H	1.451489	-4.884731	-1.596905
H	-0.898501	-5.499430	-1.914235
H	-3.303128	-4.960815	-1.672679
H	-5.087768	-3.432278	-1.008955
H	-2.147318	-0.622585	0.420873
H	2.294434	-1.960434	1.403785
H	3.913320	-1.954642	0.721648
H	1.218057	-4.318789	1.898689
H	1.698484	-6.499957	2.960101
H	4.042105	-7.317842	3.132403
H	5.900244	-5.925797	2.236471
H	5.411667	-3.741166	1.172541
H	2.771880	-3.274378	-2.502793
H	3.652992	-4.219037	-1.300250
H	3.464103	-0.536292	-2.175019
H	5.476660	0.799039	-2.753559
H	7.733122	-0.246957	-2.694239
H	7.961593	-2.646786	-2.073839
H	5.944289	-3.981644	-1.528787
H	-6.503322	-0.957324	1.271149
H	-6.108978	-2.527179	0.620093
H	-8.835130	-1.367525	0.517760
H	-10.479753	-1.041106	-1.301445
H	-9.701398	-0.687849	-3.639615
H	-7.263246	-0.664588	-4.132929
H	-5.620810	-0.999090	-2.305627
H	-5.386032	0.955762	0.027639
H	-3.683220	0.709587	-0.288168
H	-4.331117	2.909880	1.358162
H	-3.809653	3.643956	3.660711
H	-3.376228	1.957054	5.442871
H	-3.468154	-0.466639	4.886731
H	-3.980779	-1.193613	2.572033

Conformer **AC**: B3LYP/6-31G(d) [5d]

Processing: namphos-d5dbe.log
PG=C01

Method	BasisSet	Imaginary Freqs
RB3LYP	6-31G(d)	0

HF Energy
-2849.3597898

ZPE	E298	S298	Squasihar	Equasihar	Strans	Srot
678.08327	714.174	335.341	286.233	714.383	46.099	40.632

Processing: namphos-d5dbe.log
126

C	-5.042457	-3.640479	0.001789
C	-3.988679	-2.782136	0.737992
C	-4.640243	-1.484467	1.260174
C	-5.860250	-1.778602	2.150097
C	-6.897673	-2.648374	1.426018
C	-6.259973	-3.939381	0.893711
P	-2.581718	-2.390437	-0.463357
C	-1.527609	-3.985155	-0.549963
C	-1.604212	-4.984581	0.621011
C	-0.660982	-6.182664	0.405724
C	-0.937801	-6.893807	-0.926431
C	-0.870771	-5.907566	-2.101251
C	-1.807549	-4.706227	-1.889405
C	-1.456255	-1.223884	0.474097
C	-0.916719	-0.144361	-0.258673
C	-0.088344	0.840096	0.323232
C	0.219169	0.701152	1.678506
C	-0.280178	-0.352463	2.441700
C	-1.113937	-1.303670	1.846700
C	0.457990	1.989974	-0.461507
C	-0.376100	3.022745	-0.910088
C	0.181761	4.098412	-1.660874
C	1.523840	4.149680	-1.946552
C	2.397888	3.131911	-1.501051
C	1.866924	2.033168	-0.749038
C	2.754543	0.990484	-0.362002
C	4.119253	1.030917	-0.642435
C	4.631909	2.160059	-1.356260
C	3.789227	3.158374	-1.775730
N	-1.773242	3.003456	-0.638204
C	-2.200180	3.230977	0.757243
C	-2.137004	4.677128	1.231102
C	-3.302676	5.448432	1.333311
C	-3.251227	6.777937	1.759718
C	-2.026278	7.358186	2.091575

C	-0.856189	6.599921	1.997796
C	-0.912671	5.272021	1.573562
N	4.987080	0.015545	-0.232872
C	4.616888	-0.958656	0.780734
C	4.148004	-2.314313	0.258567
C	3.590036	-2.463184	-1.016533
C	3.154229	-3.714304	-1.460042
C	3.269256	-4.833850	-0.633725
C	3.825103	-4.694630	0.640775
C	4.264335	-3.445051	1.078305
O	-1.240717	-0.005661	-1.591099
C	-0.313241	-0.570594	-2.514329
O	-1.639270	-2.351241	2.552515
C	-1.368482	-2.448996	3.941540
C	-2.644644	3.620594	-1.643466
C	-4.042834	3.023717	-1.672200
C	-4.225291	1.640580	-1.819095
C	-5.510993	1.106332	-1.910000
C	-6.631593	1.941280	-1.851889
C	-6.458126	3.317377	-1.704191
C	-5.169583	3.852244	-1.615234
C	6.294753	-0.180321	-0.838953
C	7.479957	0.382188	-0.058128
C	8.770614	-0.087057	-0.339932
C	9.880008	0.427466	0.329976
C	9.713699	1.417692	1.302134
C	8.432363	1.885343	1.594882
C	7.322667	1.371148	0.918515
H	-0.019427	-0.416284	3.491287
H	0.860502	1.440837	2.150909
H	-3.614013	-3.346408	1.597546
H	-5.375891	-3.107545	-0.900688
H	-4.600840	-4.583879	-0.343410
H	-6.998026	-4.527405	0.332210
H	-5.942974	-4.564086	1.742248
H	-7.319647	-2.080504	0.583487
H	-7.735391	-2.883523	2.095653
H	-6.316439	-0.835506	2.478617
H	-5.526021	-2.298492	3.060724
H	-4.955048	-0.861753	0.410207
H	-3.909108	-0.893301	1.821665
H	-0.496259	-3.604004	-0.593371
H	-2.850606	-5.055668	-1.906348
H	-1.710813	-4.000217	-2.723272
H	-1.118787	-6.415495	-3.042748
H	0.161917	-5.541338	-2.205414
H	-1.939374	-7.348440	-0.893053
H	-0.226993	-7.717180	-1.077151
H	-0.759994	-6.888701	1.241515
H	0.379908	-5.826880	0.417735
H	-2.632968	-5.360571	0.717197

H	-1.363429	-4.480497	1.560050
H	-0.672266	-0.302148	-3.510401
H	0.693977	-0.163662	-2.365808
H	-0.283559	-1.664709	-2.425218
H	-1.903486	-3.335887	4.286226
H	-0.295665	-2.575215	4.137263
H	-1.734358	-1.569375	4.486580
H	1.932105	4.987667	-2.507590
H	-0.464121	4.908023	-1.981457
H	2.335921	0.128807	0.140817
H	4.194457	4.007375	-2.322067
H	5.696121	2.248888	-1.543545
H	-2.173517	3.450191	-2.617447
H	-2.729094	4.713293	-1.516241
H	-3.228322	2.864346	0.843941
H	-1.582636	2.601523	1.400533
H	-4.260333	4.998222	1.081685
H	-4.167920	7.357584	1.835405
H	-1.982632	8.391596	2.425712
H	0.101332	7.041937	2.261847
H	0.001965	4.688598	1.508914
H	-5.036992	4.926617	-1.505029
H	-7.321818	3.976053	-1.657453
H	-7.631570	1.520880	-1.923424
H	-5.636088	0.033621	-2.034452
H	-3.356118	0.990239	-1.862562
H	6.281529	0.243296	-1.849452
H	6.444868	-1.259484	-0.979245
H	8.906562	-0.864149	-1.090117
H	10.873343	0.051295	0.099030
H	10.576339	1.817084	1.828697
H	8.292356	2.652866	2.351800
H	6.326440	1.736090	1.150228
H	5.487529	-1.116565	1.432194
H	3.844688	-0.515726	1.419462
H	4.708882	-3.346104	2.067017
H	3.927778	-5.561426	1.288525
H	2.938216	-5.808226	-0.982583
H	2.729791	-3.814600	-2.455788
H	3.502775	-1.595811	-1.664085

More stable conformer **$APdPh_2$** [bis(N,N-dibenzyl)-2,7-diamino]: B3LYP/6-31G(d)/SDD for pre-reductive elimination step. O-Pd distance=2.453 Å, C-Pd-C angle=82.17°. ($E_{e,rel}$=0 kcal/mol)
(Near identical conformation to **A**)

Processing: pddiphnamphos-h6dsdbe.log
PG=C01

Method	BasisSet	Imaginary Freqs
RB3LYP	GenECP	0

HF Energy
-3440.5886484

ZPE	E298	S298	Squasihar	Equasihar	Strans	Srot
792.73259	836.782	397.610	334.334	837.047	46.894	41.486

Processing: pddiphnamphos-h6dsdbe.log
149

C	3.718805	1.426683	-1.909930
C	3.597143	0.005695	-2.502343
C	4.991953	-0.602385	-2.757850
C	5.849327	0.323223	-3.640949
C	5.966786	1.734799	-3.050176
C	4.579123	2.340683	-2.798625
P	2.500685	-1.046998	-1.378060
C	2.443701	-2.772489	-2.147989
C	1.103408	-3.483913	-1.863273
C	1.065335	-4.883674	-2.500898
C	2.245285	-5.755106	-2.049504
C	3.581760	-5.042042	-2.296298
C	3.607454	-3.651582	-1.638969
C	0.833872	-0.299254	-1.726571
C	0.104667	0.323888	-0.683500
C	-1.111288	1.006521	-0.943564
C	-1.635053	0.896820	-2.239536
C	-0.978074	0.249968	-3.277119
C	0.275774	-0.309178	-3.025647
C	-1.885841	1.896457	-0.012625
C	-3.161223	1.431701	0.467883
C	-3.970642	2.296750	1.271572
C	-3.504539	3.598239	1.561562
C	-2.295253	4.036570	1.084254
C	-1.459125	3.202928	0.284856
C	-3.631062	0.112673	0.217265
C	-4.859423	-0.345559	0.694228
C	-5.664605	0.551758	1.464742
C	-5.218873	1.818366	1.745289
N	-5.320831	-1.634885	0.426199
C	-6.362925	-2.268305	1.220781
C	-7.762654	-2.252207	0.612432
C	-8.722697	-3.160165	1.080734
C	-10.021171	-3.152120	0.572674
C	-10.378323	-2.237221	-0.421624
C	-9.427567	-1.334958	-0.899321
C	-8.128068	-1.342136	-0.385029
N	-0.202886	3.706009	-0.171405
C	-0.022078	3.883059	-1.630954
C	-0.676228	5.121707	-2.229966
C	-2.067848	5.192108	-2.400356
C	-2.664258	6.328617	-2.947044

C	-1.878571	7.415610	-3.339368
C	-0.493556	7.357326	-3.180086
C	0.098458	6.218869	-2.628169
O	0.652593	0.234931	0.581923
C	-0.240772	0.178635	1.722956
O	1.015116	-0.914001	-4.002814
C	0.488724	-0.985488	-5.320436
C	-4.731323	-2.470570	-0.607329
C	-3.703204	-3.493060	-0.129186
C	-3.000045	-3.331514	1.070319
C	-2.050701	-4.275416	1.471741
C	-1.792909	-5.394874	0.678769
C	-2.494891	-5.567705	-0.517416
C	-3.443426	-4.624614	-0.914100
C	0.399605	4.829020	0.565869
H	1.375383	4.992817	0.088861
H	3.078177	0.072926	-3.465665
H	4.896243	-1.575980	-3.251381
H	5.506035	-0.777519	-1.804090
H	6.844538	-0.119218	-3.776542
H	5.396845	0.388473	-4.642142
H	6.550951	2.380375	-3.718593
H	6.518142	1.685814	-2.099729
H	4.070732	2.495633	-3.762287
H	4.671960	3.329616	-2.331107
H	2.722975	1.866640	-1.775618
H	4.167180	1.363403	-0.908909
H	2.540518	-2.643208	-3.232527
H	0.965497	-3.576640	-0.776127
H	0.260916	-2.893105	-2.234610
H	0.112028	-5.368156	-2.254562
H	1.091343	-4.778852	-3.596324
H	2.224362	-6.723884	-2.565656
H	2.145806	-5.969390	-0.975049
H	3.745687	-4.939721	-3.379995
H	4.413756	-5.644587	-1.910241
H	4.575933	-3.175529	-1.817969
H	3.525602	-3.761539	-0.552247
H	-2.586665	1.380596	-2.441600
H	-1.427347	0.217433	-4.261860
H	-0.549833	1.180658	2.015463
H	0.338971	-0.279343	2.521370
H	-1.112261	-0.434528	1.481929
H	-0.449086	-1.554155	-5.347948
H	1.243991	-1.505304	-5.912247
H	0.321021	0.013747	-5.741320
H	-1.978235	5.046723	1.305159
H	-4.124250	4.260149	2.162350
H	-5.841721	2.488011	2.334180
H	-6.647617	0.246347	1.804776
H	-2.990168	-0.559370	-0.337942

H	-0.396225	2.992956	-2.133462
H	1.058235	3.923337	-1.816975
H	-2.687759	4.351337	-2.101176
H	-3.743514	6.364108	-3.072126
H	-2.343333	8.299273	-3.768674
H	0.127122	8.196040	-3.484909
H	1.179499	6.178564	-2.509924
C	0.621458	4.602895	2.048541
H	-0.156661	5.769596	0.422106
H	-6.382763	-1.802004	2.212069
H	-6.066131	-3.310943	1.397536
H	-8.449082	-3.880889	1.849310
H	-10.752410	-3.863877	0.947030
H	-11.388465	-2.232001	-0.822195
H	-9.694858	-0.621839	-1.674966
H	-7.390041	-0.639743	-0.761001
H	-4.286968	-1.818220	-1.367436
H	-5.547365	-3.003552	-1.114147
H	-3.994664	-4.772063	-1.841133
H	-2.313373	-6.444161	-1.134245
H	-1.059371	-6.131815	0.994063
H	-1.517604	-4.137399	2.408860
H	-3.203672	-2.467088	1.695333
Pd	2.808917	-0.857089	1.000516
C	0.283521	5.609686	2.961373
C	0.535836	5.455513	4.325745
C	1.127847	4.282476	4.794798
C	1.472995	3.272258	3.893144
C	1.226957	3.434416	2.528585
H	-0.182214	6.524704	2.599731
H	0.264488	6.248110	5.018622
H	1.322052	4.154053	5.856503
H	1.941123	2.359007	4.247609
H	1.501887	2.647473	1.831396
C	4.622163	-1.673651	1.260385
C	5.772413	-0.920318	0.972439
C	7.050832	-1.480301	1.085208
C	7.208126	-2.803596	1.499964
C	6.073811	-3.555703	1.813354
C	4.797448	-2.994244	1.704623
H	5.680482	0.117011	0.659312
H	7.924150	-0.873777	0.852726
H	8.200352	-3.238491	1.590612
H	6.179437	-4.582473	2.158271
H	3.933070	-3.591538	1.982577
C	2.887298	-0.718277	3.043285
C	2.114874	-1.598452	3.827178
C	2.076561	-1.503507	5.223477
C	2.829768	-0.529040	5.880418
C	3.621432	0.341814	5.127412
C	3.650523	0.242614	3.731476

H	1.532715	-2.383774	3.344999
H	1.466457	-2.199116	5.797068
H	2.810665	-0.456935	6.965235
H	4.226540	1.096196	5.627200
H	4.293732	0.920028	3.173631

Less stable conformer **JPdPh₂** [bis(N,N-dibenzyl)-2,7-diamino]: B3LYP/6-31G(d)/SDD for pre-reductive elimination step, O-Pd distance=2.417 Å, C-Pd-C angle=82.45º. ($E_{e,rel}$=2.95 kcal/mol)
(Near identical conformation to **J**)

Processing: pddiphnamphos6dsdbe.log
PG=C01

Method	BasisSet	Imaginary Freqs
RB3LYP	GenECP	0

HF Energy
-3440.5839465

ZPE	E298	S298	Squasihar	Equasihar	Strans	Srot
792.58859	836.711	396.606	335.474	836.972	46.894	41.497

Processing: pddiphnamphos6dsdbe.log
149

C	-1.287605	-4.414736	1.658780
C	-1.491689	-3.418215	0.498391
C	-2.300181	-4.064959	-0.648180
C	-1.649240	-5.374032	-1.125352
C	-1.448405	-6.364984	0.029797
C	-0.642034	-5.723407	1.167389
P	-2.183944	-1.709033	0.953986
Pd	-2.900405	-0.431280	-0.967609
C	-4.825162	-0.963815	-1.126052
C	-5.801372	-0.200238	-0.462089
C	-7.147458	-0.583010	-0.468386
C	-7.551660	-1.731154	-1.152770
C	-6.596440	-2.482702	-1.840001
C	-5.250650	-2.099488	-1.832824
C	-0.665516	-0.812113	1.567350
C	-0.053488	0.119577	0.692547
C	1.080212	0.874995	1.069881
C	1.633348	0.597154	2.327697
C	1.081419	-0.321159	3.208993
C	-0.074425	-1.011681	2.837875
C	1.757540	1.968151	0.292492
C	3.083239	1.697972	-0.208012
C	3.844780	2.752810	-0.805075
C	3.278855	4.044476	-0.883508
C	2.016197	4.289634	-0.410859
C	1.220024	3.264448	0.184657

C	5.148608	2.470088	-1.283283
C	5.694279	1.213749	-1.205826
C	4.937788	0.129947	-0.659247
C	3.655768	0.395275	-0.176921
N	-0.092370	3.589029	0.621708
C	-0.746363	4.774745	0.046725
O	-0.630129	0.292653	-0.562898
C	0.227969	0.027088	-1.698398
O	-0.670821	-1.915970	3.668457
C	-0.142766	-2.113231	4.972703
C	-3.333876	-1.926223	2.424837
C	-3.730973	-0.546584	2.993143
C	-4.703437	-0.684675	4.177312
C	-5.945046	-1.504089	3.797898
C	-5.546638	-2.875176	3.234807
C	-4.590830	-2.737619	2.036265
C	-3.299976	0.525609	-2.737448
C	-2.750880	0.002680	-3.925391
C	-2.976365	0.603721	-5.170857
C	-3.773162	1.746366	-5.262422
C	-4.332312	2.281251	-4.099020
C	-4.101527	1.674804	-2.860957
H	1.544471	-0.479108	4.175111
H	2.518407	1.150271	2.628411
H	-2.795389	-2.475389	3.200807
H	-5.114314	-2.245593	1.210710
H	-4.308830	-3.735766	1.681962
H	-6.438811	-3.434693	2.926684
H	-5.061493	-3.466691	4.026508
H	-6.521398	-0.958921	3.036971
H	-6.602976	-1.623974	4.668424
H	-4.995104	0.312154	4.532941
H	-4.186102	-1.176616	5.015033
H	-4.206278	0.045799	2.199853
H	-2.837595	0.005313	3.309237
H	-0.498862	-3.169349	0.094022
H	-3.325627	-4.270425	-0.313822
H	-2.386404	-3.358011	-1.480563
H	-2.264075	-5.824827	-1.915103
H	-0.672515	-5.145637	-1.576960
H	-2.430469	-6.680612	0.412585
H	-0.946549	-7.273348	-0.328324
H	-0.544408	-6.422470	2.008763
H	0.377728	-5.512175	0.814118
H	-2.259129	-4.649105	2.115136
H	-0.673310	-3.965518	2.443335
H	-4.572097	2.100515	-1.977456
H	-4.955486	3.171480	-4.155633
H	-3.961162	2.210833	-6.227583
H	-2.540888	0.168007	-6.068558
H	-2.151478	-0.907109	-3.893300

H	-5.515836	0.704530	0.069753
H	-7.880712	0.024773	0.058508
H	-8.597405	-2.028152	-1.161509
H	-6.897567	-3.369009	-2.395234
H	-4.532356	-2.693196	-2.391144
H	-0.390470	0.205098	-2.577131
H	1.084085	0.701081	-1.699563
H	0.563434	-1.015927	-1.673725
H	-0.799102	-2.843935	5.448282
H	0.878805	-2.512191	4.938323
H	-0.151564	-1.182858	5.553701
H	3.862928	4.852772	-1.317776
H	1.620756	5.294164	-0.470595
H	3.051401	-0.416706	0.204823
N	5.498445	-1.148060	-0.608736
H	5.732968	3.283521	-1.707579
H	6.714739	1.055934	-1.535487
H	-1.779090	4.741659	0.420143
C	-0.778504	4.836098	-1.468379
H	-0.322581	5.713869	0.439377
C	-0.441770	3.425924	2.047044
H	-1.530607	3.304645	2.103010
H	-0.002463	2.497861	2.407893
C	-0.021763	4.574671	2.956739
C	-0.981490	5.409035	3.543107
C	-0.606138	6.459059	4.384726
C	0.743736	6.692972	4.648684
C	1.712631	5.869408	4.068704
C	1.332706	4.819023	3.233141
H	-2.036025	5.231632	3.341289
H	-1.367913	7.093176	4.831066
H	1.040289	7.509291	5.302038
H	2.766410	6.043255	4.271519
H	2.094030	4.184337	2.787616
C	-0.583155	6.063315	-2.114870
C	-0.650412	6.157017	-3.505883
C	-0.908212	5.016638	-4.268464
C	-1.108712	3.789400	-3.632150
C	-1.048753	3.701007	-2.240527
H	-0.372500	6.952960	-1.523697
H	-0.494984	7.117461	-3.991186
H	-0.958481	5.083705	-5.352249
H	-1.331771	2.901266	-4.214918
H	-1.217494	2.748416	-1.748729
C	6.633861	-1.530974	-1.435100
C	4.930162	-2.216887	0.195946
H	6.445434	-2.541961	-1.820714
H	6.660241	-0.885526	-2.320016
C	7.993461	-1.515685	-0.741713
C	9.057417	-2.233217	-1.306130
C	10.322024	-2.215484	-0.719058

C	10.540469	-1.483117	0.451060
C	9.485843	-0.772071	1.024118
C	8.220425	-0.787852	0.431029
H	8.892312	-2.812193	-2.213100
H	11.135248	-2.778244	-1.170089
H	11.524134	-1.471162	0.912716
H	9.645214	-0.201927	1.935787
H	7.401333	-0.234508	0.880714
C	4.056136	-3.214530	-0.560444
H	5.759243	-2.764129	0.665212
H	4.366671	-1.768680	1.021533
C	3.912770	-4.518702	-0.068552
C	3.116105	-5.451342	-0.734048
C	2.454167	-5.093053	-1.911641
C	2.593031	-3.796757	-2.411856
C	3.388279	-2.864170	-1.740465
H	4.438742	-4.809875	0.838829
H	3.025227	-6.461500	-0.343312
H	1.845719	-5.821551	-2.440396
H	2.090751	-3.512265	-3.332975
H	3.502620	-1.860643	-2.139438

Less stable conformer **JPdPh$_2$** [bis(N,N-dibenzyl)-2,7-diamino]: B3LYP/6-31G(d)/SDD for pre-reductive elimination step, O-Pd distance=3.065 Å, C-Pd-C angle=160.0º. ($E_{e,rel}$=17.67 kcal/mol)
(Near identical conformation to **J**)

Processing: pddiphnamphos-ha6dsdbe.log
PG=C01

Method	BasisSet	Imaginary Freqs
RB3LYP	GenECP	0

HF Energy
-3440.5604886

ZPE	E298	S298	Squasihar	Equasihar	Strans	Srot
792.70890	836.795	395.928	335.284	837.053	46.894	41.369

Processing: pddiphnamphos-ha6dsdbe.log
149

C	4.966465	4.539036	-1.570957
C	3.885389	3.653844	-1.485680
C	4.144483	2.281960	-1.364097
C	5.456972	1.807924	-1.343089
C	6.530431	2.699734	-1.433504
C	6.281962	4.067824	-1.545070
C	2.461829	4.171652	-1.606032
N	1.545729	3.597307	-0.613279
C	1.885631	3.967010	0.781082
C	1.772813	5.449379	1.109006

C	2.919487	6.250159	1.200998
C	2.824088	7.612130	1.497129
C	1.573801	8.195740	1.705866
C	0.422274	7.408866	1.619992
C	0.522267	6.048612	1.326311
C	0.161056	3.594406	-0.959911
C	-0.341512	4.571039	-1.869754
C	-1.664735	4.593102	-2.237606
C	-2.581478	3.660002	-1.703034
C	-2.108658	2.664542	-0.787660
C	-0.709593	2.625920	-0.443277
C	-3.046248	1.738692	-0.256174
C	-4.402389	1.781040	-0.577658
C	-4.847696	2.773257	-1.507597
C	-3.959454	3.673197	-2.040898
C	-0.258056	1.567303	0.518690
C	0.507880	0.419752	0.195559
C	0.833524	-0.558655	1.175744
C	0.478984	-0.292891	2.520994
C	-0.294463	0.821508	2.851538
C	-0.663108	1.709142	1.850927
O	1.047097	0.194036	-1.034051
C	0.354413	0.507186	-2.250859
P	1.748414	-2.106797	0.672370
C	3.554670	-1.612898	0.982774
C	3.814835	-0.762073	2.244936
C	5.273153	-0.266263	2.280916
C	6.279814	-1.420566	2.185386
C	5.999476	-2.295469	0.956471
C	4.544238	-2.794813	0.938092
O	0.936098	-1.157952	3.471937
C	0.525612	-0.979726	4.820845
C	1.306034	-3.498779	1.875719
C	1.826582	-4.860418	1.354421
C	1.551775	-5.984409	2.370201
C	0.060342	-6.090000	2.713882
C	-0.491473	-4.735416	3.177910
C	-0.208652	-3.623407	2.151993
Pd	1.314258	-2.832954	-1.435736
C	3.202583	-2.630682	-2.234987
C	3.755101	-1.418339	-2.694788
C	4.865763	-1.387465	-3.545639
C	5.469013	-2.579283	-3.956362
C	4.952959	-3.796909	-3.508514
C	3.844679	-3.817114	-2.652700
C	-0.695701	-3.302268	-1.290909
C	-0.970341	-4.649717	-1.621396
C	-2.246723	-5.079398	-2.004541
C	-3.303360	-4.169166	-2.050314
C	-3.070687	-2.834860	-1.705378
C	-1.792416	-2.417151	-1.319361

H	-0.606765	1.006649	3.870937
H	-1.271607	2.570171	2.112654
H	1.819311	-3.259837	2.814028
H	1.320591	-5.088655	0.407671
H	2.895806	-4.824453	1.134127
H	1.919937	-6.936948	1.967592
H	2.126248	-5.791987	3.289073
H	-0.493216	-6.415438	1.821413
H	-0.102266	-6.856199	3.483100
H	-1.573354	-4.805338	3.351506
H	-0.036057	-4.467977	4.144073
H	-0.719376	-3.858479	1.212021
H	-0.625488	-2.680481	2.510586
H	3.753300	-0.984194	0.104641
H	4.384682	-3.435896	1.816299
H	4.375881	-3.401798	0.044570
H	6.681401	-3.155277	0.934620
H	6.191361	-1.722385	0.038340
H	6.208911	-2.039835	3.092473
H	7.305174	-1.029993	2.153663
H	5.438402	0.309994	3.200868
H	5.434486	0.429474	1.445214
H	3.608715	-1.358199	3.143402
H	3.142447	0.100074	2.274520
H	-0.169409	-5.389795	-1.589328
H	-2.414046	-6.123735	-2.261741
H	-4.297762	-4.494379	-2.346694
H	-3.888798	-2.117809	-1.746771
H	-1.648948	-1.373412	-1.045429
H	3.475819	-4.783643	-2.307556
H	5.416695	-4.731902	-3.817657
H	6.334074	-2.558519	-4.614961
H	5.259006	-0.431657	-3.887045
H	3.303858	-0.475722	-2.392600
H	0.699332	-0.232766	-2.976243
H	-0.726780	0.419886	-2.127710
H	0.607672	1.512671	-2.592199
H	0.974110	-1.805023	5.376692
H	0.889965	-0.028227	5.227758
H	-0.566239	-1.023774	4.916182
H	-2.025892	5.352716	-2.927538
H	0.328007	5.330845	-2.255041
H	-2.695165	1.010070	0.459667
N	-5.307449	0.878784	-0.019952
H	-4.312405	4.415985	-2.752869
H	-5.883583	2.798272	-1.826142
H	2.076101	3.897362	-2.594569
H	2.484508	5.273529	-1.570588
H	2.912483	3.632362	0.959090
H	1.242149	3.387781	1.444309
H	3.896584	5.798857	1.043777

H	3.726208	8.214690	1.567529
H	1.496005	9.254596	1.938499
H	-0.555038	7.854229	1.788168
H	-0.378886	5.444130	1.266659
H	3.308166	1.593357	-1.282259
H	5.640166	0.739097	-1.274364
H	7.551693	2.328291	-1.418440
H	7.108950	4.770110	-1.612377
H	4.776510	5.606312	-1.663628
C	-4.856633	-0.305028	0.693388
C	-6.746683	1.088640	-0.066045
H	-7.163476	0.782938	0.902738
H	-6.944715	2.164340	-0.137832
C	-7.490462	0.350196	-1.176016
H	-5.621923	-1.079736	0.557363
H	-3.957722	-0.697725	0.205101
C	-4.585355	-0.138556	2.187901
C	-4.210915	-1.265651	2.934098
C	-3.955633	-1.163848	4.301229
C	-4.071063	0.071831	4.946407
C	-4.441802	1.197971	4.211071
C	-4.697427	1.092553	2.840377
H	-4.120278	-2.230295	2.438239
H	-3.676256	-2.050425	4.865176
H	-3.881464	0.152149	6.013687
H	-4.536578	2.162896	4.702838
H	-4.978279	1.973179	2.270516
C	-8.876673	0.171251	-1.068994
C	-9.593371	-0.472680	-2.076992
C	-8.930240	-0.955263	-3.208593
C	-7.549909	-0.787184	-3.320980
C	-6.834516	-0.137798	-2.311071
H	-9.398516	0.537147	-0.186347
H	-10.667605	-0.604672	-1.976529
H	-9.485887	-1.461912	-3.993051
H	-7.024231	-1.162334	-4.195142
H	-5.760032	-0.010364	-2.403373

Low-energy conformer of **EvanPhosPdPh$_2$**: B3LYP/6-31G(d)/SDD for pre-reductive elimination step.
C-Pd-C angle=87.68°

Processing: pddiphevanphos-b6dsdbe.log
PG=C01

Method	BasisSet	Imaginary Freqs
RB3LYP	GenECP	0

HF Energy -2363.0072496

ZPE	E298	S298	Squasihar	Equasihar	Strans	Srot

516.71651 545.940 274.116 245.954 546.082 45.725 38.880

Processing: pddiphevanphos-b6dsdbe.log
97
C -4.949951 -2.001858 0.989159
C -4.925624 -0.874117 0.116343
C -6.087187 -0.030327 0.081619
C -7.209141 -0.347925 0.893726
C -7.199743 -1.448608 1.718851
C -6.053600 -2.279261 1.765017
C -6.079373 1.104155 -0.765763
C -4.991167 1.401420 -1.552572
C -3.845298 0.565486 -1.529008
C -3.795209 -0.555290 -0.699139
C -2.589936 -1.436400 -0.747183
C -1.331498 -1.045059 -0.249424
C -0.161177 -1.806413 -0.431601
C -0.301983 -3.061280 -1.073190
C -1.547517 -3.494267 -1.536742
C -2.664152 -2.677531 -1.382722
P 1.438725 -1.026895 0.129368
C 2.757770 -1.545721 -1.102171
C 2.377658 -1.055172 -2.516634
C 3.460751 -1.416044 -3.547607
C 4.837214 -0.877851 -3.132656
C 5.215784 -1.367382 -1.728179
C 4.142199 -0.993620 -0.690605
O -1.231747 0.177927 0.413606
C -1.809459 0.214468 1.740450
O 0.828278 -3.816105 -1.206042
C 0.753096 -5.058192 -1.892217
O -2.752674 0.790614 -2.312015
C -2.766995 1.870147 -3.237990
Pd 0.800436 1.312715 0.314632
C -0.041631 3.159750 0.588950
C 0.471549 4.197463 1.388932
C -0.257186 5.367242 1.629408
C -1.521476 5.542639 1.063082
C -2.050278 4.532238 0.258316
C -1.319327 3.360843 0.027435
C 2.555818 2.237807 0.079240
C 2.929813 2.731063 -1.184058
C 4.190081 3.301458 -1.395315
C 5.104289 3.407469 -0.344512
C 4.739209 2.944563 0.920964
C 3.479536 2.370862 1.130157
C 1.785755 -1.911793 1.770297
C 2.582769 -1.013297 2.741541
C 2.717310 -1.668488 4.126625
C 3.335539 -3.071096 4.036544
C 2.543030 -3.962514 3.070053

C	2.409755	-3.319214	1.677488
H	-1.654476	-4.450428	-2.034095
H	-3.620489	-3.003259	-1.781632
H	2.806431	-2.638096	-1.115907
H	4.094952	0.096359	-0.593683
H	4.435492	-1.385703	0.290441
H	6.180379	-0.942617	-1.423032
H	5.344400	-2.460584	-1.746057
H	4.812570	0.221085	-3.134059
H	5.600604	-1.179663	-3.861489
H	3.176112	-1.023266	-4.532414
H	3.514297	-2.510623	-3.650705
H	2.246901	0.034786	-2.494632
H	1.416104	-1.483949	-2.822508
H	0.774765	-2.024691	2.191647
H	3.583860	-0.819764	2.333702
H	2.091385	-0.037572	2.827949
H	3.318653	-1.026199	4.782515
H	1.721402	-1.741597	4.589364
H	4.373843	-2.987057	3.682562
H	3.380848	-3.533139	5.031014
H	3.020948	-4.946645	2.977716
H	1.538676	-4.141347	3.482646
H	3.407764	-3.247292	1.223621
H	1.812340	-3.958350	1.021039
H	-1.759347	2.582648	-0.590898
H	-3.036546	4.651519	-0.188238
H	-2.085918	6.454192	1.245931
H	0.168953	6.146665	2.258651
H	1.459998	4.099949	1.829459
H	2.228931	2.680420	-2.013410
H	4.450913	3.675163	-2.383776
H	6.082034	3.854171	-0.507245
H	5.434600	3.030352	1.753975
H	3.222340	2.021681	2.127115
H	-1.773892	1.259613	2.045244
H	-2.841556	-0.139008	1.715653
H	-1.213413	-0.400502	2.424146
H	1.769160	-5.456185	-1.893608
H	0.088106	-5.762814	-1.377183
H	0.412267	-4.925093	-2.926383
H	-6.954789	1.748525	-0.790142
H	-5.015035	2.274623	-2.193618
H	-4.079986	-2.648830	1.036327
H	-6.044759	-3.145240	2.421846
H	-8.079673	0.302735	0.851168
H	-8.063036	-1.680792	2.336024
H	-1.815343	1.812435	-3.768086
H	-3.591739	1.771397	-3.955551
H	-2.831898	2.838939	-2.728833

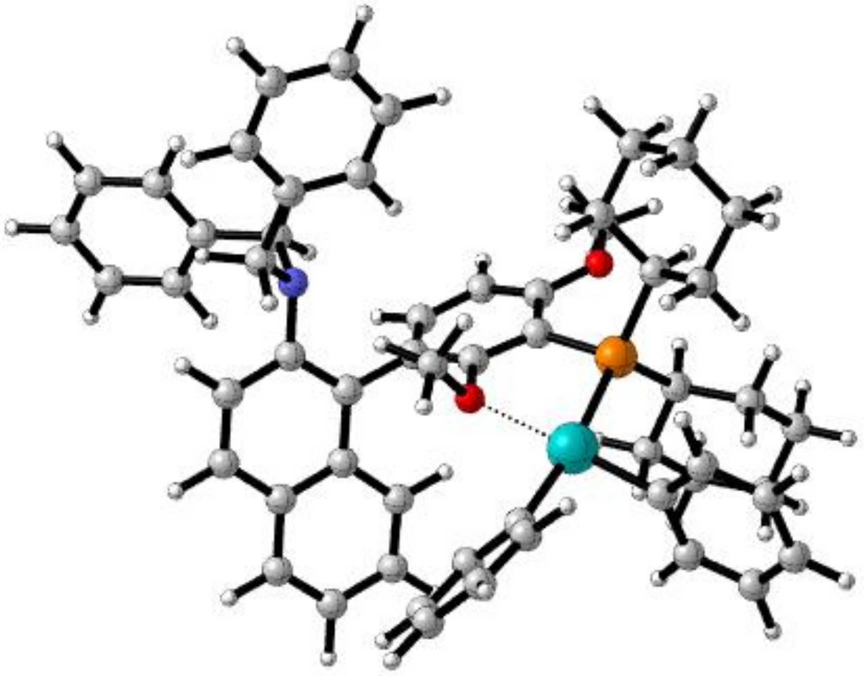

Figure S4 (repeat). B3LYP/6-31G(d)(SDD) [5d] optimized structure for more stable conformer **QPdPh$_2$** of **N,N-dibenzyl-2-aminoNPhosPdPh$_2$**. O-Pd distance=2.338 Å, C-Pd-C angle=86.28^o. Atom colors: nitrogen, blue; oxygen, red; palladium, teal. Cartesian coordinates below are for enantiomer.

Table S4. Thermodynamic parameters and Cartesian coordinates from additional optimized NPhos and N$_2$Phos structures at various levels of theory.

QPdPh$_2$, more stable conformer of **N,N-dibenzyl-2-aminoNPhosPdPh$_2$**: B3LYP/6-31G(d)/SDD for pre-reductive elimination step. O-Pd distance=2.338 Å, C-Pd-C angle=86.28^o. ($E_{e,rel}$=0.00 kcal/mol)
(Not identical conformation as **A**, cyclohexyls slightly reversed and 2-aminobenzyls rotated.)

Processing: pdnamphos-c6dsdbe.log
PG=C01

Method BasisSet Imaginary Freqs
RB3LYP GenECP 0

HF Energy
-2844.5345751

ZPE	E298	S298	Squasihar	Equasihar	Strans	Srot
644.10760	680.039	332.339	285.788	680.246	46.318	40.217

ccl00:/aue/chem126/aue/ark/pj/nam> gtg pdnamphos-c6dsdbe.log
Processing: pdnamphos-c6dsdbe.log
121

C	4.824492	-1.276383	-1.888319
C	3.344923	-0.983177	-2.222129
C	3.190669	0.424155	-2.838112
C	4.101211	0.610161	-4.063944
C	5.569528	0.319190	-3.724412
C	5.723105	-1.084366	-3.123387
P	2.248899	-1.149098	-0.696962
Pd	2.347453	0.711019	0.855862
C	2.121059	2.332242	2.075679
C	1.397021	3.431843	1.574201
C	1.064167	4.520479	2.388703
C	1.448491	4.537799	3.730910
C	2.173379	3.461255	4.246687
C	2.512192	2.379920	3.425890
C	0.528612	-0.987879	-1.388438
C	-0.348140	-0.061995	-0.794180
C	-1.592263	0.289410	-1.366603
C	-1.934911	-0.345454	-2.562071
C	-1.137981	-1.330817	-3.142562
C	0.079983	-1.664490	-2.548471
C	-2.451128	1.395976	-0.826957
C	-2.068494	2.752675	-1.111528
C	-2.846397	3.836167	-0.586004
C	-3.995499	3.539501	0.188581
C	-4.367584	2.239409	0.421121
C	-3.605529	1.145968	-0.081162
C	-0.926481	3.076521	-1.898587
C	-0.573201	4.386127	-2.140309
C	-1.341140	5.450650	-1.610915
C	-2.454884	5.175894	-0.851521
H	0.303656	4.604120	-2.743934
O	0.065169	0.590029	0.365033
C	-0.626172	0.203415	1.578850
O	0.899580	-2.643922	-3.031732
C	0.506888	-3.353086	-4.199587
C	2.418174	-2.964907	-0.188281
C	1.090943	-3.591185	0.286685
C	1.279389	-5.063668	0.693387
C	2.368082	-5.232384	1.762202

C	3.686460	-4.588363	1.311614
C	3.486510	-3.115502	0.917649
C	4.308934	0.867059	1.229922
C	5.110624	1.778156	0.518751
C	6.494188	1.836866	0.719293
C	7.110737	0.997141	1.649498
C	6.325651	0.105521	2.382559
C	4.942265	0.045239	2.177720
N	-4.002850	-0.202951	0.224233
H	3.002921	-1.724674	-2.952040
H	4.935418	-2.303931	-1.522427
H	5.163387	-0.610516	-1.085431
H	6.768408	-1.269374	-2.845482
H	5.462501	-1.835394	-3.884883
H	6.196209	0.422214	-4.619684
H	5.928455	1.062615	-2.998474
H	3.773765	-0.066609	-4.867889
H	3.991781	1.631318	-4.451588
H	2.145864	0.599883	-3.123448
H	3.440841	1.177464	-2.079508
H	2.747251	-3.508981	-1.084757
H	0.710190	-3.025082	1.150030
H	0.331470	-3.522059	-0.497868
H	0.324988	-5.472053	1.052157
H	1.552041	-5.647889	-0.198209
H	2.517058	-6.295798	1.989164
H	2.035152	-4.755872	2.696124
H	4.090101	-5.144839	0.452347
H	4.437574	-4.658069	2.108592
H	4.440159	-2.674705	0.611133
H	3.159882	-2.544731	1.797286
H	-2.846586	-0.046457	-3.070204
H	-1.466490	-1.810379	-4.056633
H	-1.701423	0.206200	1.410232
H	-0.345626	0.946058	2.324369
H	-0.295357	-0.792525	1.891695
H	-0.443152	-3.879965	-4.046734
H	1.299143	-4.080210	-4.384369
H	0.417709	-2.684556	-5.064949
H	-5.264700	2.031664	0.994180
H	-4.589503	4.359060	0.586161
H	-3.056980	5.982793	-0.440401
H	-1.050031	6.478715	-1.807417
H	-0.326944	2.275212	-2.315859
H	4.652776	2.461899	-0.191516
H	7.088042	2.552994	0.154317
H	8.185041	1.045878	1.809421
H	6.787469	-0.545921	3.122324
H	4.355777	-0.653907	2.768770
H	3.100163	1.567273	3.845959
H	2.485750	3.465360	5.289510

H	1.191167	5.382376	4.366172
H	0.501881	5.353076	1.969697
H	1.075179	3.443889	0.534932
C	-4.461068	-0.419463	1.602613
C	-4.861271	-0.868562	-0.788039
H	-4.867937	-1.935379	-0.541898
C	-6.293516	-0.367079	-0.894837
H	-4.362731	-0.767627	-1.752921
H	-5.506944	-0.107936	1.759540
C	-4.315746	-1.862587	2.065056
H	-3.852178	0.224397	2.249188
C	-3.260252	-2.674242	1.628396
C	-3.116445	-3.975149	2.113367
C	-4.022701	-4.484549	3.046300
C	-5.079593	-3.685143	3.484946
C	-5.225666	-2.386774	2.992213
H	-2.564221	-2.280274	0.894148
H	-2.293912	-4.592899	1.760994
H	-3.909148	-5.497204	3.423926
H	-5.794851	-4.072821	4.205713
H	-6.054368	-1.770203	3.334700
C	-7.327093	-1.000287	-0.189280
C	-8.642629	-0.539114	-0.275292
C	-8.945852	0.563882	-1.075300
C	-7.927618	1.199579	-1.789933
C	-6.614420	0.736226	-1.699851
H	-7.098536	-1.868890	0.424596
H	-9.430319	-1.045642	0.276572
H	-9.969407	0.921971	-1.147583
H	-8.157031	2.053154	-2.422564
H	-5.829195	1.234256	-2.263232

$QPdPh_2$, less stable conformer of **N,N-dibenzyl-2-aminoNPhosPdPh$_2$**: B3LYP/6-31G(d)/SDD for pre-reductive elimination step. O-Pd distance=3.055 Å, C-Pd-C angle=161.23^o. ($E_{e,rel}$=19.38 kcal/mol)

Processing: pdnamphos6dsdbe.log
PG=C01

Method	BasisSet	Imaginary Freqs
RB3LYP	GenECP	0

HF Energy
-2844.5036936

ZPE	E298	S298	Squasihar	Equasihar	Strans	Srot
644.35717	680.161	327.340	285.918	680.355	46.318	40.078

Processing: pdnamphos6dsdbe.log
121

C	2.967696	6.065492	-1.557220
C	3.887047	5.142795	-1.999772

C	3.809734	3.782050	-1.599838
C	2.766045	3.355125	-0.713138
C	1.826905	4.339793	-0.284884
C	1.925818	5.652022	-0.693862
C	4.743041	2.821038	-2.058059
C	4.656724	1.511171	-1.660005
C	3.623395	1.054673	-0.788037
C	2.672469	1.973441	-0.323272
C	1.583555	1.578691	0.630727
C	0.430156	0.823496	0.304357
C	-0.547136	0.497033	1.284131
C	-0.270866	0.830995	2.632112
C	0.840158	1.610519	2.963304
C	1.725989	1.986813	1.961808
P	-2.116293	-0.373763	0.767863
C	-3.497934	0.048176	1.990261
C	-4.867820	-0.422240	1.441748
C	-5.987841	-0.182499	2.470607
C	-6.070634	1.290701	2.890597
C	-4.706981	1.800078	3.376141
C	-3.602023	1.550375	2.334116
O	0.204117	0.302577	-0.932423
C	0.468499	1.050064	-2.130996
O	-1.123725	0.347679	3.579966
C	-0.912786	0.690713	4.942518
N	3.558023	-0.326106	-0.438841
C	4.123460	-1.266888	-1.414586
Pd	-2.824361	0.169836	-1.313181
C	-3.109788	2.202762	-1.058252
C	-4.341557	2.600718	-1.624734
C	-4.633019	3.941595	-1.904989
C	-3.702567	4.935058	-1.596361
C	-2.487166	4.574726	-1.008190
C	-2.204205	3.232054	-0.738902
C	-2.764226	-1.702042	-2.182804
C	-1.592212	-2.296875	-2.693948
C	-1.635487	-3.387075	-3.570342
C	-2.864603	-3.927905	-3.957331
C	-4.043953	-3.369814	-3.460191
C	-3.989272	-2.282245	-2.579664
C	-1.656472	-2.197814	1.015237
C	-0.806408	-2.508130	2.266865
C	-0.344278	-3.977839	2.268559
C	-1.521728	-4.955202	2.153635
C	-2.391271	-4.627159	0.932908
C	-2.858901	-3.161459	0.949210
H	1.034808	1.903000	3.987334
H	2.594123	2.584902	2.225521
H	-3.270817	-0.511039	2.905462
H	-5.084683	0.138525	0.522888
H	-4.850305	-1.478245	1.163765

H	-6.945591	-0.514622	2.049573
H	-5.804830	-0.806824	3.358358
H	-6.392071	1.893473	2.029076
H	-6.831548	1.424609	3.670431
H	-4.759962	2.873196	3.600711
H	-4.443433	1.295321	4.318578
H	-3.832763	2.106503	1.419441
H	-2.653243	1.939790	2.707308
H	-1.035680	-2.382645	0.128490
H	-3.495426	-3.009569	1.832016
H	-3.462997	-2.958756	0.061017
H	-3.266179	-5.288863	0.896352
H	-1.823324	-4.810838	0.009984
H	-2.138006	-4.890482	3.063215
H	-1.154748	-5.988205	2.098207
H	0.229733	-4.176567	3.183303
H	0.345219	-4.136482	1.427564
H	-1.394399	-2.308900	3.172302
H	0.071136	-1.856316	2.308153
H	-5.099440	1.850736	-1.862051
H	-5.588589	4.207898	-2.352785
H	-3.924337	5.979046	-1.804430
H	-1.754918	5.341893	-0.762175
H	-1.253097	2.987762	-0.270149
H	-4.927806	-1.880113	-2.196791
H	-5.007419	-3.784486	-3.751127
H	-2.902076	-4.776910	-4.635777
H	-0.708910	-3.813065	-3.951125
H	-0.620982	-1.895305	-2.411118
H	-0.239900	0.667749	-2.868567
H	0.289839	2.116327	-1.977688
H	1.491791	0.885293	-2.473889
H	-1.714377	0.200467	5.497357
H	0.056176	0.323297	5.302292
H	-0.973747	1.774840	5.098670
H	5.546942	3.138150	-2.718348
H	5.410178	0.808001	-1.995281
H	1.018791	4.048425	0.375987
H	1.193756	6.377563	-0.349013
H	4.691332	5.440100	-2.668892
H	3.034881	7.103732	-1.869953
C	3.520903	-2.659337	-1.334372
H	3.915076	-0.859196	-2.410103
H	5.219482	-1.351881	-1.330042
C	3.854561	-0.687558	0.966922
H	3.466279	-1.698853	1.124496
C	5.326307	-0.643796	1.353835
H	3.279603	-0.020939	1.610620
C	6.070647	-1.825825	1.469318
C	7.423224	-1.792996	1.817433
C	8.053920	-0.571155	2.055666

C	7.323330	0.615032	1.947594
C	5.972120	0.577404	1.602059
H	5.582157	-2.780921	1.289728
H	7.981646	-2.721546	1.905275
H	9.105514	-0.542159	2.328670
H	7.805074	1.570463	2.139262
H	5.411410	1.505396	1.526665
C	2.131175	-2.840375	-1.321389
C	1.584259	-4.124093	-1.346281
C	2.419133	-5.245995	-1.372353
C	3.803492	-5.075184	-1.373955
C	4.348278	-3.788209	-1.356319
H	1.485528	-1.967090	-1.288795
H	0.504737	-4.246801	-1.364358
H	1.990901	-6.244641	-1.393629
H	4.461315	-5.940372	-1.390909
H	5.428454	-3.658502	-1.366885

$QPdPh_2$, less stable conformer of **N,N-dibenzyl-2-aminoNPhos$PdPh_2$**: B3LYP/6-31G(d)/SDD for pre-reductive elimination step. O-Pd distance=3.052 Å, C-Pd-C angle=161.02°. ($E_{e,rel}$=21.20 kcal/mol)

Processing: pdnamphos-b6dsdbe.log
PG=C01

Method	BasisSet	Imaginary Freqs
RB3LYP	GenECP	0

HF Energy
-2844.5007986

ZPE	E298	S298	Squasihar	Equasihar	Strans	Srot
644.10816	679.979	330.188	286.258	680.173	46.318	40.150

Processing: pdnamphos-b6dsdbe.log
121

C	7.067464	-1.572945	-1.226996
C	5.806712	-1.019275	-1.484133
C	4.911245	-1.737089	-2.288013
C	5.271412	-2.974289	-2.823962
C	6.535200	-3.512591	-2.570218
C	7.433847	-2.806760	-1.768864
C	5.469182	0.361889	-0.940347
N	4.064097	0.515661	-0.553105
C	3.647071	-0.353578	0.571033
C	4.388172	-0.130417	1.882024
C	5.420261	-0.997027	2.268496
C	6.116652	-0.798903	3.462900
C	5.789369	0.274058	4.293038
C	4.761685	1.144551	3.921971
C	4.068490	0.942628	2.727929

C	3.561961	1.855356	-0.547899
C	4.426998	2.928326	-0.186850
C	4.001429	4.232368	-0.200079
C	2.678744	4.558406	-0.588648
C	1.786620	3.498147	-0.961055
C	2.237256	2.131612	-0.913384
C	0.484111	3.863602	-1.409154
C	0.084265	5.181139	-1.453103
C	0.959860	6.217845	-1.052132
C	2.232877	5.906633	-0.634881
C	1.325803	1.038351	-1.382148
C	0.078504	0.707078	-0.799188
C	-0.795765	-0.249403	-1.382853
C	-0.421511	-0.807509	-2.628524
C	0.826540	-0.525403	-3.192901
C	1.674047	0.377051	-2.564573
O	-0.424516	1.383287	0.277143
C	0.339443	1.535239	1.483045
P	-2.433824	-0.599834	-0.555697
C	-3.540763	0.708156	-1.369836
C	-3.323524	0.923741	-2.883715
C	-4.123030	2.143566	-3.380818
C	-5.618282	2.029618	-3.055783
C	-5.837614	1.766627	-1.560052
C	-5.046175	0.536856	-1.081714
O	-1.341657	-1.589138	-3.262936
C	-1.003446	-2.192751	-4.503791
C	-3.068345	-2.303874	-1.072542
C	-4.293739	-2.704881	-0.214617
C	-4.889684	-4.041833	-0.692039
C	-3.848249	-5.168346	-0.678172
C	-2.595059	-4.767059	-1.467605
C	-2.015808	-3.429554	-0.973179
Pd	-2.351071	-0.508235	1.701043
C	-3.681046	1.040187	2.022212
C	-3.394003	2.406628	1.822822
C	-4.190243	3.416659	2.373443
C	-5.312293	3.089049	3.139436
C	-5.631216	1.745507	3.345113
C	-4.832580	0.741529	2.783697
C	-0.978888	-2.014546	2.059019
C	-1.350827	-2.693185	3.240169
C	-0.469329	-3.543822	3.919357
C	0.812158	-3.764298	3.412485
C	1.198035	-3.130757	2.228149
C	0.312591	-2.275676	1.563620
H	1.121583	-0.962559	-4.138617
H	2.618883	0.629002	-3.033989
H	-3.382509	-2.202491	-2.118228
H	-3.969845	-2.797024	0.830799
H	-5.068397	-1.935336	-0.232337

H	-5.746294	-4.305015	-0.058220
H	-5.283323	-3.919609	-1.712581
H	-3.563003	-5.382407	0.361896
H	-4.279032	-6.093164	-1.083409
H	-1.828268	-5.548256	-1.386621
H	-2.847373	-4.685189	-2.536311
H	-1.706474	-3.532336	0.072135
H	-1.119206	-3.184436	-1.545092
H	-3.209145	1.615841	-0.848047
H	-5.421012	-0.348393	-1.614151
H	-5.215926	0.383004	-0.012934
H	-6.904647	1.619180	-1.350155
H	-5.521827	2.642355	-0.975923
H	-6.052539	1.201483	-3.636102
H	-6.145668	2.940040	-3.368410
H	-3.972911	2.262989	-4.461906
H	-3.718145	3.050476	-2.907787
H	-3.640581	0.028817	-3.435874
H	-2.263653	1.075172	-3.107782
H	-2.354065	-2.559363	3.653582
H	-0.788972	-4.040504	4.833445
H	1.500444	-4.428540	3.929368
H	2.193746	-3.301542	1.822794
H	0.640170	-1.808645	0.636578
H	-5.120214	-0.297922	2.946318
H	-6.506526	1.477193	3.933936
H	-5.933964	3.872602	3.566178
H	-3.933549	4.461466	2.206426
H	-2.526404	2.691878	1.229478
H	-0.366311	1.925824	2.217564
H	0.714617	0.567493	1.825973
H	1.158713	2.244932	1.348625
H	-1.880210	-2.771869	-4.798171
H	-0.790629	-1.438773	-5.271707
H	-0.142186	-2.864567	-4.402741
H	4.677940	5.032053	0.092504
H	5.438681	2.700333	0.129916
H	-0.200801	3.090221	-1.733571
H	-0.914979	5.426816	-1.802786
H	2.925901	6.690840	-0.338823
H	0.628507	7.252054	-1.084824
H	5.666855	1.105457	-1.722325
H	6.165031	0.584095	-0.114186
H	3.789365	-1.390559	0.250408
H	2.574483	-0.211327	0.711941
H	5.676565	-1.837880	1.627948
H	6.910306	-1.485683	3.746036
H	6.325970	0.428323	5.225475
H	4.493671	1.977354	4.567031
H	3.267829	1.623246	2.452948
H	3.924052	-1.326278	-2.474685

H 4.562339 -3.520843 -3.440813
H 6.814245 -4.476158 -2.988190
H 8.417401 -3.218516 -1.557807
H 7.769094 -1.031628 -0.595789

QPdPh₂, less stable conformer of **N,N-dibenzyl-2-aminoNPhosPdPh₂**: B3LYP/6-31G(d)/SDD for pre-reductive elimination step. O-Pd distance=3.012 Å, C-Pd-C angle=160.59°. ($E_{e,rel}$=25.26 kcal/mol)

Processing: pdnamphos-a6dsdbe.log
PG=C01

Method	BasisSet	Imaginary Freqs
RB3LYP	GenECP	0

HF Energy
-2844.4943221

ZPE	E298	S298	Squasihar	Equasihar	Strans	Srot
644.44112	680.239	327.915	285.618	680.434	46.318	40.143

Processing: pdnamphos-a6dsdbe.log
121
C -5.099034 0.716016 -0.566916
C -3.638278 0.972942 -0.991537
C -3.596334 1.407495 -2.471898
C -4.463519 2.662186 -2.693281
C -5.911562 2.455259 -2.229847
C -5.959016 1.974978 -0.773257
P -2.446427 -0.405813 -0.459315
Pd -2.235053 -0.566076 1.796175
C -3.319171 1.102174 2.346634
C -2.894229 2.442141 2.239616
C -3.521877 3.472269 2.948302
C -4.606527 3.193337 3.784412
C -5.058774 1.877821 3.902346
C -4.429892 0.854107 3.183068
C -0.884642 0.070765 -1.376532
C 0.100594 0.862204 -0.727528
C 1.273505 1.303025 -1.395451
C 1.397616 0.976102 -2.746571
C 0.446287 0.225555 -3.428192
C -0.695833 -0.211259 -2.753852
C 2.242220 2.264673 -0.769420
C 1.875007 3.655989 -0.777092
C 2.677591 4.616154 -0.078151
C 3.830436 4.157750 0.604898
C 4.198007 2.837151 0.559723
C 3.433089 1.860108 -0.147643
C 0.704843 4.134359 -1.436247
C 0.363046 5.468959 -1.410821

C	1.166047	6.409799	-0.723646
C	2.300474	5.985152	-0.071206
N	3.851038	0.499431	-0.125376
C	4.514752	-0.015871	1.075669
O	-0.222862	1.280272	0.525070
C	0.727930	1.292703	1.600656
O	-1.691424	-0.889666	-3.391957
C	-1.544557	-1.209288	-4.768511
C	-3.088846	-2.037484	-1.166292
C	-4.213156	-2.620126	-0.276696
C	-4.804336	-3.898057	-0.899730
C	-3.726426	-4.960818	-1.150588
C	-2.574163	-4.386108	-1.985400
C	-1.992991	-3.110174	-1.350749
C	-1.108373	-2.294596	1.938368
C	-1.703938	-3.279155	2.759613
C	-0.972728	-4.350910	3.286525
C	0.382121	-4.488248	2.980198
C	0.994622	-3.545959	2.149312
C	0.256250	-2.473656	1.637558
H	0.584292	0.020921	-4.482109
H	2.244828	1.369474	-3.301344
H	-3.508082	-1.789630	-2.148301
H	-3.792781	-2.852126	0.709381
H	-5.012153	-1.892758	-0.113354
H	-5.586984	-4.295559	-0.240698
H	-5.296973	-3.644987	-1.850933
H	-3.331267	-5.308033	-0.185249
H	-4.161563	-5.837446	-1.647890
H	-1.777610	-5.132803	-2.099611
H	-2.936701	-4.157909	-2.999741
H	-1.567900	-3.353279	-0.371167
H	-1.175815	-2.733988	-1.969759
H	-3.266321	1.809528	-0.385677
H	-5.517672	-0.098499	-1.174439
H	-5.142946	0.409192	0.481604
H	-6.993696	1.765091	-0.473399
H	-5.593863	2.768501	-0.106579
H	-6.394090	1.705045	-2.874530
H	-6.484913	3.383717	-2.347968
H	-4.436745	2.941967	-3.754807
H	-4.022295	3.502542	-2.137457
H	-3.961911	0.593624	-3.111697
H	-2.570590	1.622932	-2.785097
H	-2.765268	-3.212669	3.004633
H	-1.464575	-5.081455	3.926225
H	0.953169	-5.324242	3.377190
H	2.046967	-3.648744	1.892153
H	0.762647	-1.762619	0.988300
H	-4.820005	-0.160073	3.280936
H	-5.907275	1.648255	4.544387

H	-5.096832	3.993721	4.333586
H	-3.162009	4.495062	2.849510
H	-2.050155	2.686744	1.597192
H	0.150056	1.068776	2.499961
H	1.490545	0.525010	1.454078
H	1.193084	2.276441	1.693017
H	-2.444517	-1.763863	-5.039270
H	-1.475550	-0.304672	-5.385373
H	-0.663550	-1.838912	-4.942543
H	4.438536	4.872337	1.154839
H	5.098333	2.525591	1.075781
H	0.079136	3.433256	-1.976658
H	-0.532637	5.803307	-1.927706
H	2.929327	6.693018	0.464010
H	0.885003	7.459198	-0.713105
H	4.131077	-1.028997	1.251319
C	6.042410	-0.102268	1.112017
H	4.165402	0.586611	1.921997
C	4.258406	-0.149686	-1.383360
C	4.058179	-1.655233	-1.396165
H	3.675511	0.299817	-2.185680
H	5.314920	0.059386	-1.608303
C	6.621860	-1.075604	1.940250
C	8.005636	-1.186826	2.067584
C	8.845819	-0.332425	1.349299
C	8.285832	0.629751	0.509471
C	6.897344	0.745523	0.394019
H	5.976168	-1.755427	2.492517
H	8.428143	-1.946151	2.720530
H	9.925090	-0.420815	1.439603
H	8.927912	1.295787	-0.061264
H	6.484512	1.501100	-0.266572
C	2.788801	-2.219402	-1.207417
C	2.610586	-3.602275	-1.252617
C	3.700540	-4.443454	-1.496064
C	4.967745	-3.892368	-1.688003
C	5.143316	-2.507256	-1.632637
H	1.939615	-1.569492	-1.021947
H	1.621563	-4.021294	-1.089247
H	3.561393	-5.520852	-1.530918
H	5.822566	-4.538027	-1.872230
H	6.135070	-2.082458	-1.769047

$RPdPh_2$, more stable conformer of **N,N-dibenzyl-7-aminoNPhosPdPh$_2$**: B3LYP/6-31G(d)/SDD for pre-reductive elimination step. O-Pd distance=2.346 Å, C-Pd-C angle=87.36^o. ($E_{e,rel}$=0 kcal/mol)
(Near identical conformation to **A**, but naphthyl rotated near 180°)

Processing: pddiph7namphos-hb6dsdbe.log
PG=C01

Method BasisSet Imaginary Freqs
RB3LYP GenECP 0

HF Energy
-2844.5483268

ZPE	E298	S298	Squasihar	Equasihar	Strans	Srot
644.61547	680.386	328.015	284.598	680.587	46.318	39.997

Processing: pddiph7namphos-hb6dsdbe.log
121

C	-5.046358	1.780074	0.355600
C	-5.760572	1.091404	-0.628881
C	-7.018229	1.576972	-1.016056
C	-7.550183	2.724016	-0.429101
C	-6.828616	3.407249	0.554907
C	-5.576687	2.932212	0.944225
C	-5.215701	-0.167883	-1.299888
N	-3.867331	-0.562795	-0.919056
C	-2.798944	0.042053	-1.706216
C	-2.617121	-0.555865	-3.096563
C	-2.696854	-1.938640	-3.314627
C	-2.509851	-2.468281	-4.592225
C	-2.244423	-1.624499	-5.673567
C	-2.171465	-0.245651	-5.468957
C	-2.360178	0.282072	-4.189199
C	-3.627911	-1.453338	0.120790
C	-4.681703	-1.765514	1.040786
C	-4.492430	-2.674377	2.049602
C	-3.247509	-3.327149	2.248763
C	-2.162921	-2.985604	1.373836
C	-2.389830	-2.070459	0.315665
C	-3.067686	-4.295081	3.267166
C	-1.857952	-4.933346	3.423051
C	-0.778783	-4.594953	2.576032
C	-0.895224	-3.630386	1.586306
C	0.296733	-3.336886	0.733122
C	0.987080	-2.104225	0.733725
C	2.077470	-1.843708	-0.116792
C	2.557399	-2.912063	-0.912499
C	1.914313	-4.151519	-0.910040
C	0.792871	-4.339166	-0.105684
P	2.759598	-0.114402	-0.169715
C	4.549394	-0.266186	0.439118
C	4.772699	-1.422802	1.434346
C	6.243066	-1.500090	1.883015
C	6.732951	-0.174332	2.481763
C	6.489926	0.990655	1.512608
C	5.017277	1.061562	1.075450
O	0.564144	-1.092433	1.597029
C	0.825390	-1.335291	3.004206

O	3.684647	-2.663458	-1.642158
C	4.210516	-3.694526	-2.467168
Pd	1.085970	1.132433	1.068512
C	1.472881	2.998030	0.460050
C	0.723582	3.576748	-0.580106
C	1.030732	4.849654	-1.073571
C	2.086525	5.584077	-0.528978
C	2.824188	5.033907	0.520765
C	2.518022	3.758890	1.011095
C	-0.396635	1.924974	2.245038
C	-1.590615	1.183446	2.361908
C	-2.610608	1.560752	3.245202
C	-2.466460	2.700521	4.038995
C	-1.294989	3.453990	3.937513
C	-0.282097	3.073985	3.050371
C	2.943778	0.258120	-2.007228
C	1.569440	0.248530	-2.707596
C	1.702092	0.565464	-4.206991
C	2.420572	1.902067	-4.437432
C	3.789288	1.913764	-3.743841
C	3.668349	1.602807	-2.240959
H	0.181164	-5.086262	2.716788
H	3.557075	-0.541331	-2.435163
H	4.671971	1.577546	-1.800372
H	3.120199	2.412540	-1.745295
H	4.278014	2.887439	-3.875781
H	4.442898	1.166584	-4.219568
H	2.535846	2.094205	-5.512158
H	1.806398	2.719531	-4.033039
H	2.267827	-0.240168	-4.699489
H	0.706987	0.573843	-4.667510
H	1.080223	-0.724389	-2.577251
H	0.919792	0.996517	-2.234215
H	5.157165	-0.469094	-0.454126
H	4.135652	-1.270364	2.318324
H	4.474337	-2.376932	0.990140
H	6.364160	-2.315680	2.607811
H	6.867596	-1.758924	1.014894
H	7.797224	-0.243687	2.740657
H	6.193934	0.019713	3.420959
H	7.128155	0.866369	0.624844
H	6.783099	1.941170	1.976229
H	4.868227	1.902155	0.390770
H	4.386438	1.269055	1.950825
H	0.278303	-5.295448	-0.126849
H	2.267843	-4.966966	-1.529269
H	0.400062	-2.292713	3.306839
H	0.338028	-0.518841	3.534567
H	1.905113	-1.318440	3.186392
H	4.507413	-4.569052	-1.874857
H	5.091700	-3.267428	-2.948578

H 3.489343 -4.001804 -3.234630
H -1.721804 -5.684279 4.195968
H -3.907072 -4.531017 3.917449
H -5.312629 -2.898799 2.727714
H -5.635901 -1.258796 0.959956
H -1.573391 -1.879162 -0.369232
H -5.897938 -1.008236 -1.126267
H -5.213982 -0.019468 -2.387739
H -7.584423 1.052543 -1.784378
H -8.526712 3.086714 -0.740179
H -7.241638 4.302837 1.011520
H -5.001782 3.448408 1.708347
H -4.071803 1.421016 0.670658
H -1.866964 -0.012447 -1.135448
H -3.017215 1.113270 -1.808237
H -2.314727 1.358701 -4.037867
H -1.980932 0.421640 -6.305577
H -2.104298 -2.037903 -6.668826
H -2.576346 -3.542661 -4.744181
H -2.914942 -2.600084 -2.481004
H -0.118176 3.036920 -1.005853
H 0.432102 5.271662 -1.878695
H 2.322920 6.574788 -0.909176
H 3.641615 5.597810 0.966397
H 3.105279 3.358911 1.833457
H 0.609414 3.691512 2.984977
H -1.166985 4.345054 4.549605
H -3.253931 2.994929 4.729192
H -3.514371 0.957758 3.310415
H -1.731163 0.282992 1.768565

RPdPh$_2$, less stable conformer of **N,N-dibenzyl-7-aminoNPhosPdPh$_2$**: B3LYP/6-31G(d)/SDD for pre-reductive elimination step. O-Pd distance=2.334 Å, C-Pd-C angle=85.60°. ($E_{e,rel}$=0.46 kcal/mol)
(Near identical conformation to **A**)

Processing: pddiph7namphos-h6dsdbe.log
PG=C01

Method	BasisSet	Imaginary Freqs
RB3LYP	GenECP	0

HF Energy
-2844.5475982

ZPE	E298	S298	Squasihar	Equasihar	Strans	Srot
644.04613	680.055	337.916	285.684	680.266	46.318	40.569

Processing: pddiph7namphos-h6dsdbe.log
121
C -2.863728 3.952424 -0.383195

C	-3.137216	2.882015	-1.256395
C	-3.326059	3.193168	-2.614908
C	-3.216779	4.505959	-3.086999
C	-2.935558	5.550525	-2.203961
C	-2.763433	5.268858	-0.847136
Pd	-3.037412	0.978772	-0.525405
C	-4.980115	0.858534	-1.002117
C	-5.976573	1.311913	-0.119514
C	-7.334912	1.162421	-0.420782
C	-7.732352	0.569273	-1.620867
C	-6.755271	0.136647	-2.519252
C	-5.397374	0.284734	-2.214942
P	-2.600677	-1.163280	0.509833
C	-0.926568	-0.894646	1.274678
C	-0.239514	0.300077	0.992029
C	0.954969	0.667139	1.650303
C	1.482507	-0.250387	2.564024
C	0.874618	-1.475766	2.824412
C	-0.323589	-1.797563	2.182457
O	-0.782923	1.167699	0.046545
C	0.002105	1.330710	-1.161994
C	1.609372	1.998662	1.478654
C	2.966687	2.113163	1.015300
C	3.558829	3.418551	0.960972
C	2.805388	4.556839	1.341281
C	1.498957	4.425267	1.754825
C	0.909240	3.143557	1.822768
C	4.901155	3.524210	0.509755
C	5.623582	2.427209	0.113284
C	5.034696	1.122991	0.116983
C	3.721167	0.997143	0.566809
O	-0.967401	-2.988142	2.367542
C	-0.405872	-3.941503	3.259259
H	-0.117210	3.049267	2.164663
N	5.777063	0.020519	-0.307036
C	5.372782	-1.341778	-0.001843
C	4.577698	-2.065402	-1.086541
C	3.914924	-1.375959	-2.107892
C	3.182277	-2.069635	-3.075052
C	3.101943	-3.462305	-3.032545
C	3.761961	-4.159365	-2.016488
C	4.496223	-3.464576	-1.055872
C	-3.679541	-1.585942	1.997389
C	-3.768648	-0.372553	2.948394
C	-4.663850	-0.669194	4.163647
C	-6.063024	-1.131040	3.733725
C	-5.974634	-2.342137	2.795455
C	-5.088647	-2.051758	1.570426
C	-2.447413	-2.779852	-0.461074
C	-1.049904	-2.971161	-1.086114
C	-0.961261	-4.288658	-1.875974

C	-2.050758	-4.389541	-2.952367
C	-3.444586	-4.173492	-2.346768
C	-3.521297	-2.848338	-1.569997
C	6.954001	0.152857	-1.153273
C	8.298895	0.061141	-0.437586
C	9.450918	-0.231123	-1.181034
C	10.701513	-0.288524	-0.567094
C	10.817992	-0.061573	0.807002
C	9.676036	0.222260	1.556385
C	8.424356	0.283688	0.937705
H	-3.189998	-2.410246	2.527252
H	-5.019996	-2.957709	0.957103
H	-5.564078	-1.279991	0.952424
H	-6.975914	-2.639601	2.458958
H	-5.561643	-3.199425	3.348879
H	-6.672634	-1.373371	4.613811
H	-6.572696	-0.307880	3.212716
H	-4.195934	-1.453125	4.778426
H	-4.732121	0.224783	4.796886
H	-2.766617	-0.082919	3.288272
H	-4.174139	0.486516	2.397272
H	-2.615074	-3.595225	0.256173
H	-0.838406	-2.130376	-1.763899
H	-0.274735	-2.955712	-0.314725
H	0.036361	-4.376249	-2.325341
H	-1.064346	-5.131688	-1.176210
H	-1.998121	-5.362004	-3.458836
H	-1.870909	-3.624719	-3.722566
H	-3.681789	-5.008142	-1.669897
H	-4.209352	-4.183551	-3.133763
H	-4.526751	-2.710994	-1.160559
H	-3.362401	-2.011398	-2.262385
H	2.393294	0.012077	3.094351
H	1.329244	-2.155557	3.534833
H	1.023243	1.624477	-0.915381
H	-0.494349	2.117935	-1.727282
H	-0.000502	0.393286	-1.728376
H	0.593193	-4.255602	2.932173
H	-1.080085	-4.799219	3.238093
H	-0.347699	-3.549920	4.282546
H	0.917734	5.297907	2.037691
H	3.275883	5.536199	1.292202
H	5.367491	4.506612	0.491318
H	6.660136	2.552920	-0.179238
H	3.233526	0.030962	0.540318
H	6.887981	1.099500	-1.701206
H	6.907967	-0.630318	-1.922109
H	9.366381	-0.416805	-2.250326
H	11.584090	-0.517138	-1.158857
H	11.791004	-0.110039	1.288358
H	9.756045	0.396837	2.626263

H	7.537432	0.502336	1.525062
H	4.801413	-1.330469	0.933662
H	6.281509	-1.920267	0.212894
H	5.017857	-4.014835	-0.274657
H	3.713586	-5.244610	-1.979607
H	2.539160	-4.001876	-3.789536
H	2.678491	-1.519056	-3.865363
H	3.975827	-0.292503	-2.146496
H	-5.695606	1.799006	0.810927
H	-8.083044	1.523486	0.282508
H	-8.787541	0.457066	-1.857639
H	-7.047029	-0.314490	-3.465947
H	-4.659225	-0.052262	-2.938997
H	-3.575456	2.402603	-3.319596
H	-3.362992	4.713004	-4.145669
H	-2.858781	6.572666	-2.567421
H	-2.552580	6.074783	-0.146216
H	-2.722727	3.761659	0.679664

RPdPh$_2$, less stable conformer of **N,N-dibenzyl-7-aminoNPhosPdPh$_2$**: B3LYP/6-31G(d)/SDD for pre-reductive elimination step. O-Pd distance=3.207 Å, C-Pd-C angle=85.48°. ($E_{e,rel}$=10.04 kcal/mol)
(Near identical conformation to **A**)

Processing: pddiph7namphos-ha6dsdbe.log
PG=C01

Method	BasisSet	Imaginary Freqs
RB3LYP	GenECP	0

HF Energy
-2844.5323326

ZPE	E298	S298	Squasihar	Equasihar	Strans	Srot
643.49948	679.830	341.889	288.258	680.048	46.318	40.640

Processing: pddiph7namphos-ha6dsdbe.log
121

C	8.474275	-0.693548	-0.631899
C	8.293623	-0.113786	0.628242
C	9.416161	0.370019	1.314669
C	10.691478	0.266885	0.760454
C	10.863002	-0.316795	-0.497777
C	9.750878	-0.794007	-1.191788
C	6.919355	-0.016396	1.285215
N	5.779796	-0.121807	0.386238
C	5.399722	1.108567	-0.288628
C	4.601915	2.107782	0.545679
C	3.847294	1.711127	1.655890
C	3.112920	2.647718	2.387896
C	3.122841	3.994112	2.018933

C	3.874567	4.399464	0.912619
C	4.610300	3.462603	0.186878
C	5.050691	-1.302936	0.241361
C	5.634324	-2.553488	0.620673
C	4.926565	-3.722889	0.502781
C	3.606235	-3.754127	-0.018961
C	3.020937	-2.516147	-0.446771
C	3.758464	-1.314004	-0.281271
C	2.868702	-4.960047	-0.116786
C	1.584225	-4.955569	-0.612214
C	1.002892	-3.743817	-1.047033
C	1.688363	-2.541438	-0.987829
C	1.046291	-1.309569	-1.537021
C	-0.165373	-0.793130	-1.020031
C	-0.827900	0.306673	-1.603593
C	-0.215584	0.913532	-2.729663
C	0.989859	0.431126	-3.243830
C	1.598605	-0.671392	-2.648881
P	-2.467031	0.890563	-0.931488
C	-2.211584	2.748971	-0.632409
C	-0.809609	3.067501	-0.070102
C	-0.629488	4.572892	0.190431
C	-1.714337	5.129047	1.122346
C	-3.115644	4.801690	0.589631
C	-3.286049	3.293605	0.337907
O	-0.745407	-1.419701	0.056752
C	-0.116136	-1.183272	1.321259
O	-0.858689	1.995796	-3.264282
C	-0.288810	2.649409	-4.389000
Pd	-3.435715	-0.056863	1.146102
C	-4.545036	-1.627736	0.582544
C	-3.894700	-2.805499	0.184881
C	-4.630802	-3.878875	-0.331748
C	-6.019750	-3.795820	-0.449580
C	-6.671743	-2.632068	-0.036420
C	-5.942972	-1.558942	0.489876
C	-4.114561	-0.444235	2.998930
C	-3.663122	-1.520401	3.785214
C	-3.958213	-1.594178	5.149907
C	-4.734198	-0.603762	5.757383
C	-5.216752	0.457444	4.989149
C	-4.918701	0.529429	3.623523
C	-3.576439	0.781733	-2.459355
C	-3.601724	-0.665384	-3.000975
C	-4.515802	-0.792263	-4.231464
C	-5.939194	-0.306990	-3.926573
C	-5.919415	1.134063	-3.399969
C	-5.007606	1.280071	-2.167755
H	-0.004401	-3.753163	-1.452709
H	-3.129749	1.430400	-3.221262
H	-4.991252	2.331964	-1.859789

H	-5.429288	0.707372	-1.331980
H	-6.934548	1.463626	-3.143110
H	-5.564443	1.806028	-4.196248
H	-6.568529	-0.376072	-4.823429
H	-6.389955	-0.964426	-3.169594
H	-4.099426	-0.199659	-5.060580
H	-4.531035	-1.836357	-4.569229
H	-2.586816	-0.987803	-3.261272
H	-3.958002	-1.344112	-2.216519
H	-2.315810	3.254363	-1.600610
H	-0.663124	2.520839	0.874323
H	-0.029911	2.720477	-0.753210
H	0.369010	4.751679	0.609015
H	-0.667515	5.108548	-0.770153
H	-1.594793	6.213190	1.245478
H	-1.596631	4.684948	2.122078
H	-3.285534	5.346766	-0.351057
H	-3.883861	5.146395	1.293542
H	-4.294909	3.091443	-0.035032
H	-3.206901	2.759776	1.298319
H	2.521441	-1.059525	-3.070708
H	1.451964	0.891642	-4.108510
H	0.926418	-1.518385	1.316045
H	-0.686878	-1.754014	2.054604
H	-0.161206	-0.117290	1.578380
H	0.703889	3.055476	-4.157217
H	-0.967539	3.469295	-4.630230
H	-0.213351	1.974854	-5.251091
H	1.015900	-5.878639	-0.682532
H	3.334035	-5.886540	0.212157
H	5.388938	-4.660205	0.804019
H	6.656799	-2.587402	0.980131
H	3.271903	-0.381564	-0.537954
H	6.824638	-0.781062	2.064308
H	6.846667	0.944488	1.812505
H	9.288827	0.833132	2.291575
H	11.550535	0.647995	1.306549
H	11.855423	-0.394307	-0.933600
H	9.873690	-1.246413	-2.172503
H	7.610652	-1.063892	-1.176353
H	4.841068	0.846242	-1.194233
H	6.318874	1.598271	-0.638236
H	5.204241	3.787416	-0.665626
H	3.897766	5.446942	0.623223
H	2.559173	4.723566	2.594317
H	2.537495	2.324148	3.251457
H	3.839321	0.665758	1.949615
H	-2.812939	-2.878568	0.248947
H	-4.110451	-4.782340	-0.643831
H	-6.588247	-4.631379	-0.850158
H	-7.755155	-2.559186	-0.106805

H	-6.470733	-0.673018	0.830157
H	-5.321185	1.357422	3.041810
H	-5.833819	1.227053	5.448928
H	-4.970068	-0.665092	6.817091
H	-3.589605	-2.432652	5.737667
H	-3.077928	-2.315019	3.327672

RPdPh$_2$, less stable conformer of **N,N-dibenzyl-7-aminoNPhosPdPh$_2$**: B3LYP/6-31G(d)/SDD for pre-reductive elimination step. O-Pd distance=3.054 Å, C-Pd-C angle=161.67°. ($E_{e,rel}$=22.78 kcal/mol) (Near identical conformation to **J**, but naphthyl rotated near 180°)

Processing: pddiph7namphos-hc6dsdbe.log
PG=C01

Method	BasisSet	Imaginary Freqs
RB3LYP	GenECP	0

HF Energy
-2844.5120183

ZPE	E298	S298	Squasihar	Equasihar	Strans	Srot
643.93767	680.002	339.104	286.745	680.206	46.318	40.408

Processing: pddiph7namphos-hc6dsdbe.log
121

C	0.606912	0.924996	-2.009107
C	-0.563094	0.794117	-1.224428
C	-1.390643	-0.356046	-1.295905
C	-1.057307	-1.343434	-2.255154
C	0.130204	-1.259737	-2.987260
C	0.942292	-0.141145	-2.849005
O	-1.955312	-2.353251	-2.446668
H	0.410873	-2.036318	-3.687172
H	1.843323	-0.066397	-3.450404
O	-1.006564	1.802734	-0.419686
P	-2.904864	-0.442832	-0.211031
C	-0.108573	2.463715	0.490426
H	-0.689193	2.650515	1.396390
H	0.239887	3.406938	0.064994
H	0.744851	1.825733	0.731846
C	-1.638511	-3.396183	-3.357637
H	-2.483795	-4.085574	-3.322179
H	-0.724457	-3.925101	-3.060743
H	-1.523131	-3.014156	-4.379586
C	1.417608	2.178797	-2.079889
C	0.834980	3.332856	-2.577243
C	1.564867	4.534603	-2.720523
C	2.897369	4.571929	-2.374843
C	3.541953	3.416477	-1.867034
C	2.804593	2.197370	-1.701348

C	4.913124	3.421512	-1.497439
C	5.534931	2.306500	-0.993677
C	4.814413	1.082101	-0.823956
C	3.465489	1.058985	-1.174534
H	1.071052	5.419086	-3.112938
H	3.474305	5.487190	-2.487000
H	5.477277	4.345338	-1.603784
H	6.571911	2.373859	-0.684454
H	2.900469	0.141833	-1.079648
H	-0.212923	3.309783	-2.864086
N	5.449901	-0.045336	-0.305011
C	4.682609	-1.187287	0.161872
C	6.898533	-0.162035	-0.241061
C	4.297380	-2.224434	-0.892916
C	4.721819	-2.132413	-2.221667
C	4.363140	-3.111336	-3.153131
C	3.574364	-4.194879	-2.765964
C	3.141867	-4.292334	-1.440001
C	3.500882	-3.314391	-0.513335
H	5.273722	-1.679437	0.944861
H	3.776152	-0.823755	0.662325
H	5.326097	-1.284503	-2.530100
H	4.703311	-3.024513	-4.182017
H	3.300214	-4.959058	-3.488655
H	2.526324	-5.131609	-1.126398
H	3.155163	-3.395937	0.515576
H	7.162338	-1.201977	-0.474429
H	7.336767	0.433994	-1.050381
C	7.544190	0.221711	1.088524
C	6.846982	0.916433	2.081379
C	7.476911	1.265629	3.279497
C	8.811607	0.924939	3.497868
C	9.515595	0.228728	2.511035
C	8.884183	-0.121347	1.318600
H	5.807492	1.183181	1.916126
H	6.919949	1.804229	4.041708
H	9.300598	1.195425	4.429808
H	10.554610	-0.046114	2.673483
H	9.436637	-0.669449	0.557139
C	-3.427734	-2.243420	0.024857
C	-2.274854	-3.189238	0.426964
C	-2.770435	-4.642554	0.530990
C	-3.917491	-4.778295	1.541487
C	-5.051877	-3.796437	1.220172
C	-4.540517	-2.349382	1.096591
H	-3.831785	-2.567136	-0.941673
H	-1.879335	-2.870670	1.396979
H	-1.449089	-3.131929	-0.284019
H	-1.934057	-5.293850	0.816109
H	-3.112252	-4.985983	-0.457786
H	-4.296358	-5.808510	1.558582

H	-3.532897	-4.566832	2.549637
H	-5.535041	-4.091576	0.276359
H	-5.828615	-3.843206	1.994361
H	-5.379183	-1.685778	0.875794
H	-4.127651	-2.019775	2.059408
C	-4.192869	0.403758	-1.316184
C	-5.646562	0.286531	-0.815415
C	-6.578736	1.204486	-1.625430
C	-6.515038	0.887599	-3.125583
C	-5.069419	0.935885	-3.637778
C	-4.128309	0.035211	-2.814844
H	-3.894596	1.455904	-1.214365
H	-5.708594	0.542166	0.245563
H	-5.990014	-0.750681	-0.933297
H	-6.288253	2.250016	-1.451145
H	-7.606827	1.100085	-1.255854
H	-7.143198	1.585786	-3.693700
H	-6.927490	-0.117681	-3.300292
H	-5.026548	0.640412	-4.694401
H	-4.702318	1.971626	-3.588555
H	-3.108863	0.138584	-3.196947
H	-4.415123	-1.016267	-2.951977
Pd	-2.612062	0.497090	1.826171
C	-4.047629	1.975001	1.709235
C	-5.100418	1.914248	2.648785
C	-5.939982	3.008176	2.892176
C	-5.764398	4.195892	2.179702
C	-4.743325	4.279659	1.229110
C	-3.904739	3.184108	0.997636
H	-5.275609	0.994194	3.207649
H	-6.735965	2.928091	3.630333
H	-6.419124	5.045489	2.358759
H	-4.598881	5.201553	0.668162
H	-3.117349	3.276799	0.251150
C	-1.086327	-0.711205	2.537371
C	0.222686	-0.915026	2.058895
C	1.227323	-1.456493	2.867159
C	0.951795	-1.827570	4.186091
C	-0.340394	-1.658735	4.685384
C	-1.340863	-1.122321	3.865250
H	0.469407	-0.649231	1.032460
H	2.232561	-1.587890	2.468793
H	1.732973	-2.250072	4.813593
H	-0.574245	-1.952827	5.706871
H	-2.345342	-1.021876	4.282072

SPdPh$_2$, more stable stable form of *o,o,o,o*-**tetramethyl-N,N-dibenzyl-2-aminoNPhosPdPh$_2$**: B3LYP/6-31G(d)/SDD for pre-reductive elimination step. C-Pd-C angle=82.23°, ($E_{e,rel}$=0.00 kcal/mol).

Processing: pdnamtmphos-b6dsdbe.log

PG=C01

Method	BasisSet	Imaginary Freqs
RB3LYP	GenECP	0

HF Energy
-3001.7740609

ZPE	E298	S298	Squasihar	Equasihar	Strans	Srot
714.77811	754.513	352.390	309.351	754.713	46.495	40.395

Processing: pdnamtmphos-b6dsdbe.log
133

C	2.283280	1.588130	3.693509
C	2.808310	1.915022	2.429140
C	3.357228	3.201707	2.275363
C	3.345166	4.133295	3.319309
C	2.797972	3.793139	4.558410
C	2.269831	2.513660	4.742923
Pd	2.580729	0.628970	0.857312
C	4.471565	0.153811	1.324912
C	4.784428	-0.907184	2.189133
C	6.112914	-1.282781	2.418064
C	7.160066	-0.596544	1.800340
C	6.864364	0.479426	0.960721
C	5.535967	0.855720	0.733416
P	2.045887	-0.840638	-0.985632
C	3.363464	-1.158265	-2.287802
C	4.589821	-1.873835	-1.677485
C	5.681722	-2.091133	-2.740327
C	6.102177	-0.767414	-3.394062
C	4.889797	-0.040689	-3.993141
C	3.782602	0.173507	-2.946838
C	0.580796	-0.057191	-1.846166
C	-0.168298	0.929446	-1.145479
C	-1.387876	1.439924	-1.662342
C	-1.725782	1.066952	-2.970452
C	-0.987437	0.162509	-3.716529
C	0.137565	-0.429670	-3.136792
C	-2.356676	2.357757	-0.964214
C	-3.275449	1.925760	0.019137
C	-4.111812	2.923722	0.625767
C	-4.077277	4.242667	0.262924
C	-3.213541	4.695885	-0.761918
C	-2.351497	3.737362	-1.385793
C	-1.466733	4.228374	-2.392829
C	-1.458235	5.557214	-2.762074
C	-2.328681	6.487723	-2.149989
C	-3.186862	6.056047	-1.164474
N	-3.423106	0.597375	0.469123
C	-3.394342	-0.552924	-0.455252

C	-4.686836	-1.348293	-0.665348
C	-4.670874	-2.753636	-0.489048
C	-5.837452	-3.490582	-0.731230
C	-7.007954	-2.871297	-1.158715
C	-7.009899	-1.496829	-1.374028
C	-5.862892	-0.724913	-1.143945
C	-3.425627	-3.514467	-0.078680
C	-5.932027	0.754594	-1.449133
O	0.347880	1.333158	0.072678
C	0.109874	2.689741	0.533119
O	0.862447	-1.381817	-3.792925
C	0.497476	-1.743018	-5.118028
C	-4.036705	0.389323	1.799030
C	-3.696644	-0.911951	2.515074
C	-4.726918	-1.800662	2.903114
C	-4.392392	-2.965771	3.606803
C	-3.073077	-3.253148	3.944160
C	-2.067702	-2.352440	3.602186
C	-2.362817	-1.174164	2.904223
C	-6.192777	-1.530712	2.627805
C	-1.253045	-0.187500	2.634624
C	1.331677	-2.518517	-0.447159
C	1.317737	-3.644590	-1.502038
C	0.638280	-4.915042	-0.958325
C	1.283471	-5.395641	0.348576
C	1.298160	-4.275207	1.397761
C	1.983965	-3.005445	0.865939
H	-1.308840	-0.096138	-4.717401
H	-2.628577	1.491530	-3.401084
H	2.935452	-1.803455	-3.058325
H	4.999303	-1.279408	-0.854901
H	4.298441	-2.841676	-1.252851
H	6.548323	-2.579887	-2.277686
H	5.309435	-2.777878	-3.516068
H	6.570659	-0.126145	-2.633910
H	6.859757	-0.946258	-4.168086
H	5.191696	0.927252	-4.414005
H	4.487998	-0.633603	-4.828864
H	4.144128	0.861326	-2.170299
H	2.915613	0.653575	-3.416443
H	0.286880	-2.263931	-0.213428
H	3.049911	-3.210564	0.699319
H	1.939363	-2.207408	1.615134
H	1.805789	-4.613185	2.310536
H	0.263850	-4.033522	1.685437
H	2.316320	-5.716414	0.146494
H	0.752502	-6.275114	0.734849
H	0.677207	-5.706358	-1.718585
H	-0.427113	-4.706546	-0.779834
H	2.349593	-3.890913	-1.787563
H	0.812545	-3.309712	-2.411392

H	3.807140	3.487965	1.326084
H	3.772329	5.122601	3.165899
H	2.792818	4.512921	5.373465
H	1.852598	2.230835	5.707717
H	1.876318	0.594095	3.870402
H	5.334336	1.706400	0.086974
H	7.668712	1.037189	0.484871
H	8.191872	-0.887409	1.980796
H	6.326460	-2.111681	3.090211
H	3.990765	-1.451726	2.693203
H	0.895705	2.890409	1.256332
H	0.180728	3.386148	-0.304303
H	-0.867257	2.765902	1.006886
H	1.226999	-2.492437	-5.429542
H	-0.508767	-2.178555	-5.154451
H	0.547756	-0.882621	-5.796529
H	-4.741851	4.953563	0.748615
H	-4.830731	2.623294	1.376430
H	-0.784660	3.542566	-2.881585
H	-0.771065	5.891646	-3.535003
H	-3.860404	6.753727	-0.671755
H	-2.313356	7.530939	-2.452385
H	-3.687853	1.211894	2.432942
H	-5.127580	0.482763	1.736278
H	-2.608919	-1.238394	-0.129117
H	-3.077964	-0.172972	-1.423508
H	-5.818613	-4.567914	-0.585525
H	-7.905224	-3.457315	-1.340054
H	-7.909305	-1.006370	-1.739250
H	-1.039024	-2.547918	3.895570
H	-2.832352	-4.163162	4.487675
H	-5.185305	-3.650836	3.896633
H	-0.301086	-0.551012	3.031987
H	-1.117642	0.017419	1.570637
H	-1.455248	0.780159	3.114519
H	-6.802313	-2.373350	2.968114
H	-6.547961	-0.639476	3.161491
H	-6.400754	-1.382114	1.564119
H	-6.818078	0.976758	-2.052455
H	-5.991431	1.367714	-0.543430
H	-5.054735	1.106355	-2.002765
H	-3.654049	-4.578793	0.033951
H	-2.631717	-3.432047	-0.832442
H	-3.013506	-3.163899	0.872422

SPdPh$_2$, less stable stable conformer of *o,o,o,o*-**tetramethyl-N,N-dibenzyl-2-aminoNPhosPdPh$_2$**: B3LYP/6-31G(d)/SDD for pre-reductive elimination step. C-Pd-C angle=160.86°, ($E_{e,rel}$=16.37 kcal/mol).

Processing: pdnamtmphos6dsdbe.log
PG=C01

Method	BasisSet	Imaginary Freqs
RB3LYP	GenECP	0

HF Energy
-3001.7479707

ZPE	E298	S298	Squasihar	Equasihar	Strans	Srot
714.69369	754.432	351.050	310.067	754.626	46.495	40.376

Processing: pdnamtmphos6dsdbe.log
133

C	5.701479	-2.058706	-1.374496
C	4.460215	-1.416474	-1.593495
C	3.320994	-2.188231	-1.918268
C	3.417903	-3.585480	-1.939786
C	4.627462	-4.223214	-1.676152
C	5.759822	-3.458849	-1.411383
C	4.374274	0.104376	-1.614393
N	3.469970	0.732823	-0.626794
C	3.471509	0.179841	0.740125
C	4.818048	-0.020337	1.444712
C	5.113789	-1.280575	2.020367
C	6.319997	-1.455844	2.710949
C	7.227192	-0.411050	2.858080
C	6.915619	0.837939	2.330676
C	5.718424	1.054069	1.634313
C	2.971378	2.018689	-0.920695
C	3.686454	2.790486	-1.899225
C	3.320819	4.059100	-2.258915
C	2.213380	4.699030	-1.655623
C	1.464919	3.964430	-0.679931
C	1.822008	2.607120	-0.343714
C	0.343262	4.628673	-0.098769
C	0.004889	5.918359	-0.450385
C	0.759235	6.633537	-1.408265
C	1.843600	6.025274	-1.997927
C	0.919588	1.911245	0.639179
C	-0.089123	0.982187	0.286896
C	-0.897016	0.342337	1.265625
C	-0.586084	0.570962	2.627236
C	0.341295	1.552630	2.990520
C	1.051185	2.218095	1.998961
O	-0.299497	0.570997	-0.996294
C	-0.400437	1.517764	-2.076655
P	-2.271688	-0.791075	0.701169
C	-1.354367	-2.444328	0.549750
C	-0.328519	-2.731302	1.668477
C	0.495498	-3.993251	1.347517
C	-0.391980	-5.218217	1.090596
C	-1.440792	-4.921021	0.010894
C	-2.268297	-3.671815	0.359887

O	-1.209676	-0.214574	3.550786
C	-0.965741	0.012679	4.932266
C	-3.560791	-0.958236	2.074718
C	-4.812505	-1.709733	1.557211
C	-5.823361	-1.946352	2.694340
C	-6.244032	-0.633727	3.368102
C	-5.017101	0.166383	3.825534
C	-4.022450	0.389252	2.672620
Pd	-3.338191	-0.088631	-1.167295
C	-3.037665	-1.740746	-2.370318
C	-1.853491	-1.985879	-3.095519
C	-1.808353	-2.900506	-4.153272
C	-2.954649	-3.613408	-4.514489
C	-4.141051	-3.403118	-3.808924
C	-4.174425	-2.489075	-2.748593
C	-3.982224	1.767588	-0.523323
C	-5.288014	1.992334	-1.012602
C	-5.874699	3.264023	-1.011116
C	-5.174427	4.351078	-0.485863
C	-3.891419	4.152997	0.031173
C	-3.311397	2.880544	0.015236
H	0.539145	1.777045	4.031143
H	1.784717	2.965883	2.289039
H	-3.087550	-1.555036	2.863205
H	-5.279099	-1.104199	0.768473
H	-4.551214	-2.665984	1.099687
H	-6.701867	-2.470591	2.296587
H	-5.374624	-2.615492	3.444149
H	-6.818761	-0.029198	2.651718
H	-6.910998	-0.835261	4.216369
H	-5.326820	1.137605	4.232435
H	-4.515244	-0.371207	4.645008
H	-4.505203	0.976304	1.884410
H	-3.176106	0.979353	3.027829
H	-0.802729	-2.294123	-0.388088
H	-2.813968	-3.866741	1.293522
H	-3.004028	-3.485289	-0.426241
H	-2.111807	-5.779808	-0.118049
H	-0.946515	-4.762369	-0.958012
H	-0.904421	-5.497362	2.023671
H	0.224240	-6.079896	0.803236
H	1.192677	-4.192337	2.172014
H	1.113936	-3.799865	0.459118
H	-0.852190	-2.869144	2.624158
H	0.349912	-1.883031	1.799470
H	-5.872259	1.159144	-1.411782
H	-6.879208	3.400061	-1.407157
H	-5.625886	5.340182	-0.472089
H	-3.339782	4.994362	0.447255
H	-2.314169	2.758773	0.432505
H	-5.112173	-2.360723	-2.207043

H	-5.039661	-3.955287	-4.078075
H	-2.922243	-4.327329	-5.334091
H	-0.878550	-3.055729	-4.698247
H	-0.946002	-1.445025	-2.834775
H	-1.033192	1.033559	-2.822684
H	-0.876195	2.441701	-1.742327
H	0.583461	1.730888	-2.499159
H	-1.571968	-0.722920	5.463332
H	0.091731	-0.138326	5.181748
H	-1.272429	1.021607	5.234560
H	3.906525	4.599433	-2.999271
H	4.578006	2.375782	-2.350089
H	-0.257490	4.111176	0.638972
H	-0.856301	6.389479	0.016165
H	2.438575	6.551791	-2.740966
H	0.481208	7.649141	-1.675224
H	4.004892	0.395709	-2.603578
H	5.386628	0.516938	-1.524167
H	2.938042	-0.773093	0.725409
H	2.872899	0.848078	1.353063
C	4.160286	-2.457078	1.952648
H	6.542188	-2.428853	3.142461
H	8.159899	-0.564116	3.394792
H	7.601761	1.670671	2.467224
C	5.433787	2.453198	1.135851
C	2.012595	-1.541180	-2.303233
H	2.536223	-4.171725	-2.187603
H	4.691323	-5.308097	-1.697015
H	6.713454	-3.950001	-1.234272
C	6.991283	-1.297654	-1.141476
H	1.260958	-2.300423	-2.538366
H	1.609054	-0.892127	-1.522418
H	2.132156	-0.912658	-3.197223
H	7.810913	-1.995157	-0.943812
H	7.276510	-0.705504	-2.020856
H	6.928172	-0.613570	-0.290297
H	6.112115	3.169872	1.609896
H	5.563911	2.547697	0.052397
H	4.408297	2.771619	1.351655
H	4.605357	-3.331312	2.437837
H	3.214237	-2.247237	2.467992
H	3.916571	-2.740673	0.924248

SPdPh$_2$, less stable conformer of *o,o,o,o*-**tetramethyl-N,N-dibenzyl-2-aminoNPhosPdPh$_2$**: B3LYP/6-31G(d)/SDD for pre-reductive elimination step. C-Pd-C angle=160.82°, ($E_{e,rel}$=18.23 kcal/mol).

Processing: pdnamtmphos-a6dsdbe.log
PG=C01

Method BasisSet Imaginary Freqs

RB3LYP GenECP 0

HF Energy
-3001.7450045

ZPE	E298	S298	Squasihar	Equasihar	Strans	Srot
714.30133	754.257	354.800	312.127	754.454	46.495	40.502

Processing: pdnamtiprphos6dsdbe.log
133

C	-3.787278	-3.681867	0.432918
C	-3.456952	-2.603983	-0.420580
C	-3.581596	-2.756289	-1.820276
C	-4.055800	-3.965755	-2.340923
C	-4.391417	-5.025453	-1.502148
C	-4.251041	-4.880927	-0.124876
C	-2.928537	-1.298014	0.164534
N	-3.867885	-0.144831	0.125772
C	-5.295885	-0.528606	0.091614
C	-6.360757	0.465560	0.550164
C	-7.484330	0.699124	-0.283364
C	-8.503876	1.550974	0.157197
C	-8.446330	2.159382	1.407629
C	-7.364799	1.896736	2.240052
C	-6.323904	1.051850	1.833209
C	-3.429685	1.113504	-0.329596
C	-2.190783	1.699584	0.016511
C	-1.901936	3.048714	-0.409085
C	-2.814374	3.757759	-1.256300
C	-4.006467	3.103616	-1.649193
C	-4.300880	1.846599	-1.196921
C	-2.524278	5.082914	-1.673417
C	-1.380172	5.723113	-1.256328
C	-0.489553	5.048784	-0.389561
C	-0.739577	3.756067	0.017994
C	-1.167993	1.020068	0.881503
C	0.164843	0.753396	0.467110
C	1.144660	0.238178	1.355615
C	0.775957	0.062447	2.712420
C	-0.542228	0.268741	3.124131
C	-1.481431	0.737432	2.212674
P	2.887188	-0.026204	0.731130
C	3.770001	-1.306928	1.805715
C	5.118181	-1.714175	1.161808
C	5.898311	-2.678244	2.074319
C	5.079561	-3.929927	2.416270
C	3.710943	-3.548831	2.996373
C	2.948059	-2.587659	2.067605
O	0.622183	1.110635	-0.770218
C	-0.036593	0.659765	-1.964277
O	1.765251	-0.273612	3.589825

C	1.441469	-0.466992	4.959125
C	3.659989	1.668885	1.088752
C	5.190399	1.720573	0.903983
C	5.698099	3.171977	0.959826
C	5.307493	3.854081	2.277689
C	3.794792	3.762156	2.517794
C	3.279408	2.312070	2.440282
Pd	3.002119	-0.689418	-1.427649
C	2.007185	-2.501260	-1.303676
C	2.647578	-3.435185	-2.147894
C	2.036178	-4.636098	-2.530136
C	0.765429	-4.952966	-2.048054
C	0.117366	-4.063722	-1.186034
C	0.735030	-2.862644	-0.819974
C	4.016585	0.928218	-2.212467
C	3.445553	2.189281	-2.481891
C	4.069918	3.116541	-3.323159
C	5.297647	2.812724	-3.917805
C	5.895267	1.577533	-3.660520
C	5.266938	0.658330	-2.811206
H	-0.833336	0.117815	4.156236
H	-2.488207	0.936010	2.557338
H	3.967987	-0.810180	2.762884
H	4.909509	-2.202012	0.200272
H	5.738620	-0.843169	0.939500
H	6.840248	-2.959128	1.585775
H	6.173139	-2.155708	3.003215
H	4.928785	-4.522636	1.502561
H	5.631390	-4.568273	3.118496
H	3.105964	-4.448886	3.166082
H	3.850574	-3.074810	3.980394
H	2.754180	-3.084525	1.111390
H	1.976225	-2.350580	2.504117
H	3.212661	2.274005	0.288923
H	5.674230	1.149711	1.708836
H	5.476620	1.266889	-0.048380
H	6.787997	3.183601	0.831748
H	5.277155	3.728989	0.111122
H	5.836160	3.363779	3.109150
H	5.629433	4.903415	2.278587
H	3.534114	4.190949	3.494391
H	3.271056	4.366176	1.762184
H	3.708638	1.726825	3.264647
H	2.194810	2.310266	2.581401
H	3.651967	-3.228780	-2.526632
H	2.558166	-5.325349	-3.191271
H	0.286821	-5.886784	-2.333075
H	-0.871276	-4.304885	-0.801418
H	0.210707	-2.197496	-0.135391
H	5.769357	-0.289551	-2.614163
H	6.854886	1.331725	-4.111738

H	5.785447	3.533468	-4.569836
H	3.596102	4.077405	-3.516862
H	2.489871	2.453703	-2.031804
H	0.621100	0.962856	-2.780218
H	-0.124132	-0.430016	-1.960539
H	-1.015178	1.127211	-2.085267
H	2.380046	-0.730179	5.449675
H	1.039465	0.449132	5.409363
H	0.721896	-1.284553	5.091239
H	-4.694770	3.612751	-2.319813
H	-5.217270	1.379554	-1.526141
H	-0.046195	3.277345	0.698007
H	0.403499	5.556841	-0.034594
H	-3.235132	5.586039	-2.325216
H	-1.166185	6.738826	-1.577078
H	-5.371234	-1.405078	0.745458
H	-5.564604	-0.898037	-0.903758
H	-2.036374	-1.007208	-0.391367
H	-2.599477	-1.472179	1.192864
C	-7.657452	0.038127	-1.638574
H	-9.357979	1.729720	-0.491378
H	-9.246425	2.817635	1.735678
H	-7.321196	2.344559	3.230291
C	-5.194904	0.810848	2.807524
C	-3.177208	-1.656300	-2.776432
H	-4.151355	-4.075892	-3.418394
H	-4.755834	-5.960282	-1.919850
H	-4.505136	-5.707425	0.534319
C	-3.650824	-3.595489	1.940900
H	-5.547023	0.929189	3.837990
H	-4.756130	-0.185963	2.708714
H	-4.390080	1.538475	2.651464
H	-8.578525	0.389401	-2.113536
H	-6.834361	0.254059	-2.331437
H	-7.727082	-1.053780	-1.560824
H	-3.470297	-1.906482	-3.800573
H	-3.623489	-0.689281	-2.523637
H	-2.088326	-1.518374	-2.774807
H	-4.018399	-4.513468	2.409533
H	-2.607699	-3.463488	2.253895
H	-4.219707	-2.760001	2.367481

TPdPh$_2$, more stable form of *o,o,o,o*-**tetraiospropyl-N,N-dibenzyl-2-aminoNPhosPdPh$_2$**: B3LYP/6-31G(d)/SDD for pre-reductive elimination step. C-Pd-C angle=83.63º, ($E_{e,rel}$=0.00 kcal/mol).
Processing: pdnamtiprphos-b6dsdbe.log
PG=C01

Method	BasisSet	Imaginary Freqs
RB3LYP	GenECP	0

HF Energy
-3316.2563336

ZPE	E298	S298	Squasihar	Equasihar	Strans	Srot
858.45138	904.944	401.699	349.929	905.182	46.821	41.058

Processing: pdnamtiprphos-b6dsdbe.log
157

C	3.686298	2.341737	3.261270
C	3.727715	2.335934	1.854909
C	3.806607	3.582972	1.207053
C	3.806039	4.782420	1.927698
C	3.739150	4.764613	3.322728
C	3.682255	3.538015	3.987913
Pd	3.448568	0.632260	0.771398
C	5.404922	0.367462	1.101307
C	5.857134	-0.479035	2.126998
C	7.220347	-0.749906	2.291141
C	8.164350	-0.170999	1.440743
C	7.730816	0.693163	0.432865
C	6.368006	0.966929	0.271715
P	2.795231	-1.267454	-0.574849
C	4.019097	-1.889902	-1.858313
C	5.265716	-2.498720	-1.174795
C	6.298356	-2.957148	-2.220121
C	6.704592	-1.809300	-3.154159
C	5.472537	-1.195301	-3.833398
C	4.426818	-0.737557	-2.802233
C	1.290519	-0.623882	-1.482548
C	0.581573	0.469981	-0.902810
C	-0.539837	1.051691	-1.545240
C	-0.854934	0.559184	-2.821650
C	-0.210008	-0.514735	-3.410897
C	0.838344	-1.130297	-2.719522
C	-1.359578	2.236098	-1.093764
C	-2.564220	2.149564	-0.357112
C	-3.226807	3.383025	-0.037471
C	-2.750758	4.608187	-0.420582
C	-1.567944	4.725052	-1.185903
C	-0.877493	3.520522	-1.537325
C	0.319115	3.662191	-2.303653
C	0.783192	4.900952	-2.692127
C	0.085811	6.081096	-2.344382
C	-1.069479	5.986429	-1.603205
N	-3.146350	0.955415	0.141131
C	-3.226493	-0.280182	-0.667424
C	-4.595418	-0.799028	-1.147207
C	-4.973299	-2.141389	-0.847518
C	-6.187095	-2.633313	-1.344708
C	-7.028096	-1.846902	-2.126337
C	-6.639501	-0.555518	-2.452421

C	-5.425198	-0.022037	-1.994874
C	-4.062868	-3.106328	-0.076638
C	-5.036298	1.359468	-2.523905
O	1.094918	0.887345	0.309828
C	0.529786	1.966615	1.085994
O	1.472048	-2.233631	-3.211302
C	1.088419	-2.747216	-4.479739
C	-4.063807	1.098167	1.296527
C	-4.359909	-0.131759	2.160777
C	-5.705343	-0.559120	2.341195
C	-5.966511	-1.608636	3.232940
C	-4.946395	-2.233220	3.943025
C	-3.636677	-1.798824	3.782560
C	-3.326224	-0.747458	2.910117
C	-6.895766	0.132666	1.665674
C	-1.888776	-0.234833	2.870567
C	2.106548	-2.767291	0.354474
C	1.921427	-4.057189	-0.470621
C	1.267837	-5.169337	0.369653
C	2.056551	-5.448533	1.656743
C	2.241507	-4.165361	2.479391
C	2.896423	-3.045009	1.653118
H	-0.526252	-0.862313	-4.386125
H	-1.663157	1.044383	-3.362032
H	3.536888	-2.669657	-2.452133
H	5.726164	-1.761852	-0.508590
H	4.980952	-3.354877	-0.552203
H	7.179828	-3.361901	-1.707090
H	5.875865	-3.780016	-2.817173
H	7.219080	-1.035469	-2.567147
H	7.418970	-2.164247	-3.908328
H	5.765071	-0.344753	-4.462864
H	5.019000	-1.940324	-4.504973
H	4.842449	0.084595	-2.204982
H	3.545695	-0.340214	-3.319658
H	1.108802	-2.414822	0.653833
H	3.928192	-3.331574	1.410781
H	2.963744	-2.125919	2.246221
H	2.846992	-4.367348	3.372344
H	1.259923	-3.822056	2.838372
H	3.043920	-5.856802	1.394164
H	1.550169	-6.216416	2.255582
H	1.180303	-6.083603	-0.231952
H	0.241800	-4.868571	0.630769
H	2.900135	-4.410387	-0.823805
H	1.321017	-3.855998	-1.362623
H	3.866230	3.626848	0.120586
H	3.862346	5.731477	1.397594
H	3.742301	5.695378	3.885075
H	3.645510	3.509810	5.075479
H	3.665477	1.398944	3.805350

H	6.058407	1.659925	-0.506439
H	8.454849	1.168943	-0.225885
H	9.223360	-0.380424	1.569258
H	7.542373	-1.413616	3.091378
H	5.144574	-0.940745	2.806023
H	1.073599	1.940898	2.028020
H	0.703796	2.924495	0.595530
H	-0.531771	1.802966	1.249654
H	1.734547	-3.608948	-4.655155
H	0.040670	-3.072216	-4.481382
H	1.243353	-2.009164	-5.276280
H	-3.300938	5.507547	-0.153353
H	-4.162291	3.353578	0.503555
H	0.876039	2.777749	-2.589414
H	1.697511	4.968986	-3.275736
H	-1.622005	6.879755	-1.320767
H	0.462828	7.050052	-2.658998
H	-3.613942	1.852677	1.951211
H	-5.016210	1.517734	0.964374
H	-2.729021	-1.072461	-0.109159
H	-2.612080	-0.117575	-1.546145
H	-6.483468	-3.652044	-1.120826
H	-7.969454	-2.248404	-2.493037
H	-7.276810	0.046624	-3.094262
H	-2.844649	-2.269629	4.356886
H	-5.175442	-3.045879	4.627821
H	-6.987536	-1.943113	3.380100
C	-0.831335	-1.352937	2.844161
H	-1.767808	0.331467	1.945594
C	-1.630801	0.719469	4.056639
C	-8.122747	-0.773133	1.457430
C	-7.313969	1.388731	2.462437
H	-6.587251	0.446521	0.663628
C	-6.065320	2.447901	-2.162323
H	-4.090561	1.658898	-2.075965
C	-4.809232	1.316880	-4.050207
C	-4.800025	-4.267186	0.614242
C	-2.969211	-3.675152	-1.008661
H	-3.572297	-2.549908	0.727601
H	-4.100154	-4.806159	1.262789
H	-5.623070	-3.906620	1.238437
H	-5.198366	-4.994546	-0.103107
H	-2.293203	-4.337499	-0.453147
H	-3.424469	-4.259951	-1.817422
H	-2.365555	-2.886586	-1.470757
H	-5.713362	3.430653	-2.496597
H	-7.036221	2.262618	-2.636515
H	-6.229831	2.500316	-1.080456
H	-4.461363	2.291836	-4.412791
H	-4.058934	0.564153	-4.319391
H	-5.730791	1.069069	-4.589556

H 0.161678 -0.923638 2.669654
H -0.784861 -1.903820 3.790822
H -1.033766 -2.075697 2.045385
H -0.616590 1.133978 4.010910
H -2.338913 1.555993 4.065559
H -1.735358 0.188896 5.010632
H -8.856153 -0.252517 0.831333
H -7.852964 -1.704807 0.951839
H -8.623661 -1.019419 2.401118
H -8.140814 1.907557 1.961985
H -7.650171 1.108359 3.467919
H -6.489364 2.100405 2.580131

TPdPh$_2$, less stable conformer of *o,o,o,o*-**tetraiospropyl-N,N-dibenzyl-2-aminoNPhosPdPh$_2$**: B3LYP/6-31G(d)/SDD for pre-reductive elimination step. C-Pd-C angle=160.72º, ($E_{e,rel}$=15.73 kcal/mol).

Processing: pdnamtiprphos-a6dsdbe.log
PG=C01

Method BasisSet Imaginary Freqs
RB3LYP GenECP 0

HF Energy
-3316.2312720

ZPE	E298	S298	Squasihar	Equasihar	Strans	Srot
858.36920	904.782	396.946	351.202	905.004	46.821	40.982

Processing: pdnamtiprphos-a6dsdbe.log
157
C 3.830725 -2.441081 0.242663
C 3.363815 -1.293726 0.946971
C 3.425799 -1.270462 2.363580
C 3.992368 -2.357680 3.044836
C 4.457789 -3.472519 2.360834
C 4.363187 -3.512265 0.972542
C 2.671512 -0.167128 0.170381
N 3.247846 1.193285 0.225420
C 4.716357 1.374259 0.259344
C 5.566219 0.686297 -0.817629
C 6.606802 -0.209452 -0.443111
C 7.406693 -0.773399 -1.447203
C 7.216971 -0.464168 -2.789542
C 6.244042 0.461218 -3.146243
C 5.428462 1.062731 -2.178641
C 2.448921 2.299564 0.602638
C 1.126513 2.535333 0.162543
C 0.476782 3.779924 0.502393
C 1.116374 4.728231 1.365214
C 2.401667 4.412453 1.865766

C	3.036568	3.258894	1.493737
C	0.471699	5.948898	1.694994
C	-0.763614	6.260171	1.175886
C	-1.389483	5.351052	0.292099
C	-0.792439	4.151747	-0.031789
C	0.372079	1.594945	-0.732349
C	-0.878637	1.010166	-0.396852
C	-1.624017	0.236566	-1.325345
C	-1.115257	0.119358	-2.642940
C	0.130463	0.655017	-2.976892
C	0.844107	1.376058	-2.027375
P	-3.293092	-0.440139	-0.821063
C	-3.702883	-1.961293	-1.866494
C	-4.948052	-2.687749	-1.301423
C	-5.353929	-3.870314	-2.200161
C	-4.202562	-4.866831	-2.387023
C	-2.938388	-4.156573	-2.889114
C	-2.547751	-2.980283	-1.976725
O	-1.506543	1.270141	0.786970
C	-0.841339	1.077178	2.044111
O	-1.908170	-0.497061	-3.565562
C	-1.432614	-0.654781	-4.894813
C	-4.441058	0.969747	-1.363282
C	-5.940811	0.615361	-1.299774
C	-6.807902	1.871511	-1.492733
C	-6.491595	2.574823	-2.819439
C	-4.992998	2.883841	-2.934671
C	-4.121197	1.631161	-2.721423
Pd	-3.460661	-0.979314	1.371192
C	-2.110541	-2.544313	1.464776
C	-2.652588	-3.606330	2.222775
C	-1.854834	-4.634225	2.741505
C	-0.482507	-4.644694	2.488504
C	0.080676	-3.625221	1.715293
C	-0.726583	-2.599764	1.209821
C	-4.863881	0.421727	1.948327
C	-4.611807	1.793651	2.156058
C	-5.497671	2.605217	2.873132
C	-6.675268	2.068408	3.400629
C	-6.959726	0.716212	3.201356
C	-6.071285	-0.087068	2.475876
H	0.527484	0.561077	-3.979912
H	1.781955	1.831715	-2.315495
H	-3.937017	-1.581132	-2.867807
H	-4.708984	-3.053820	-0.294353
H	-5.795995	-2.007714	-1.192141
H	-6.228295	-4.372864	-1.766839
H	-5.669830	-3.487696	-3.182646
H	-3.983591	-5.349254	-1.423641
H	-4.496500	-5.665697	-3.080250
H	-2.102076	-4.864953	-2.948493

H	-3.109313	-3.786734	-3.912117
H	-2.314617	-3.359922	-0.976635
H	-1.639690	-2.508313	-2.356411
H	-4.246800	1.708716	-0.574632
H	-6.180119	-0.101680	-2.097488
H	-6.182049	0.145819	-0.342503
H	-7.868919	1.594179	-1.450553
H	-6.629998	2.558651	-0.653757
H	-6.792020	1.924107	-3.654623
H	-7.078404	3.497379	-2.915825
H	-4.766054	3.324493	-3.914442
H	-4.720445	3.638930	-2.182559
H	-4.301916	0.916988	-3.535836
H	-3.066569	1.916291	-2.777471
H	-3.726026	-3.640008	2.422797
H	-2.308397	-5.429215	3.330557
H	0.142421	-5.443126	2.881389
H	1.148538	-3.627652	1.508060
H	-0.261101	-1.829439	0.596537
H	-6.332218	-1.134742	2.320339
H	-7.877072	0.287424	3.600738
H	-7.365799	2.698733	3.956012
H	-5.266965	3.658651	3.022857
H	-3.701926	2.239408	1.757101
H	-1.639091	1.080153	2.788624
H	-0.336658	0.109083	2.066298
H	-0.136875	1.884192	2.253478
H	-2.223645	-1.182754	-5.429882
H	-1.253525	0.315523	-5.374417
H	-0.513115	-1.252207	-4.924484
H	2.883872	5.096731	2.560152
H	4.004786	3.042678	1.927199
H	-1.291260	3.489269	-0.727667
H	-2.353706	5.601380	-0.142709
H	0.982938	6.638892	2.362636
H	-1.250370	7.197560	1.430068
H	5.104606	1.101091	1.243037
H	4.887531	2.448252	0.154563
H	1.652665	-0.083408	0.550014
H	2.565892	-0.467198	-0.871803
C	6.961883	-0.501301	1.020111
H	8.197935	-1.463369	-1.176054
H	7.844201	-0.922173	-3.550260
H	6.134824	0.739438	-4.189695
C	4.515268	2.210601	-2.611061
C	2.842631	-0.141097	3.216147
H	4.044399	-2.338262	4.129820
H	4.882303	-4.313020	2.904064
H	4.714662	-4.394794	0.449278
C	3.717990	-2.583013	-1.281889
H	3.724568	2.314415	-1.865650

C	5.310174	3.535450	-2.630163
C	3.836326	1.987914	-3.975283
C	7.713718	-1.824943	1.245630
C	7.795960	0.659699	1.608482
H	6.033659	-0.582369	1.592831
C	3.914644	0.569679	4.066773
H	2.407645	0.611844	2.556727
C	1.709693	-0.663714	4.126145
C	4.775173	-3.509398	-1.910080
C	2.309069	-3.069144	-1.688544
H	3.883785	-1.597365	-1.728933
H	7.784515	-2.022515	2.321013
H	7.192874	-2.670016	0.786379
H	8.738202	-1.792200	0.855929
H	8.023864	0.474450	2.665362
H	8.746305	0.758352	1.070010
H	7.278243	1.622404	1.538232
H	4.654748	4.377105	-2.885115
H	5.768007	3.747122	-1.657570
H	6.116081	3.493387	-3.372669
H	3.094036	2.773772	-4.159263
H	4.552688	2.028242	-4.803403
H	3.328675	1.017596	-4.020479
H	2.231094	-3.149430	-2.780153
H	2.106869	-4.058860	-1.262232
H	1.518153	-2.396210	-1.342196
H	4.727029	-3.426740	-3.001879
H	5.787931	-3.236329	-1.599490
H	4.605431	-4.563461	-1.660718
H	1.218706	0.171567	4.640520
H	0.953877	-1.213562	3.556132
H	2.095780	-1.342211	4.895658
H	3.468127	1.401961	4.623587
H	4.364618	-0.114143	4.796060
H	4.724570	0.973621	3.449810

TPdPh$_2$, less stable conformer of *o,o,o,o*-**tetraiospropyl-N,N-dibenzyl-2-aminoNPhosPdPh$_2$**: B3LYP/6-31G(d)/SDD for pre-reductive elimination step. C-Pd-C angle=160.25°, ($E_{e,rel}$=25.19 kcal/mol).
Processing: pdnamtiprphos6dsdbe.log
PG=C01

Method	BasisSet	Imaginary Freqs
RB3LYP	GenECP	0

HF Energy
-3316.2161920

ZPE	E298	S298	Squasihar	Equasihar	Strans	Srot
858.94903	905.199	395.057	349.387	905.422	46.821	41.017

Processing: pdnamtiprphos6dsdbe.log
157

C	5.853757	-1.640901	-1.354037
C	4.477957	-1.336735	-1.589642
C	3.586475	-2.388987	-1.933603
C	4.065797	-3.710191	-1.926638
C	5.388647	-4.012470	-1.641433
C	6.276326	-2.975739	-1.379798
C	4.056974	0.133945	-1.644009
N	3.037559	0.643021	-0.693723
C	3.138969	0.217417	0.724009
C	4.490320	0.231376	1.465774
C	4.952275	-0.973557	2.075214
C	6.133281	-0.946790	2.828027
C	6.865318	0.224632	2.996332
C	6.396497	1.403074	2.434328
C	5.207791	1.434073	1.689410
C	2.431419	1.880531	-1.034109
C	3.065114	2.670996	-2.054778
C	2.609561	3.902594	-2.440305
C	1.493037	4.497663	-1.812066
C	0.818560	3.743707	-0.798592
C	1.246308	2.405194	-0.459976
C	-0.295097	4.377057	-0.167493
C	-0.695598	5.650330	-0.513809
C	-0.023223	6.377260	-1.522646
C	1.052682	5.801673	-2.157669
C	0.369182	1.695431	0.541024
C	-0.709674	0.833813	0.208810
C	-1.550501	0.269397	1.210577
C	-1.202955	0.489741	2.565925
C	-0.185095	1.384307	2.907029
C	0.550817	1.989819	1.897200
O	-0.970353	0.420466	-1.062441
C	-1.008080	1.335887	-2.171440
P	-3.055833	-0.734526	0.726882
C	-2.403694	-2.515847	0.772305
C	-1.443347	-2.854430	1.932904
C	-0.852256	-4.267617	1.765744
C	-1.940064	-5.340527	1.626488
C	-2.912600	-4.987513	0.493653
C	-3.513143	-3.584088	0.685330
O	-1.896996	-0.206823	3.509827
C	-1.609222	0.009224	4.884383
C	-4.360566	-0.576552	2.091936
C	-5.724118	-1.127971	1.606925
C	-6.762234	-1.114568	2.744073
C	-6.946435	0.290994	3.330635
C	-5.598762	0.886723	3.758709
C	-4.575006	0.862892	2.609418
Pd	-4.008239	-0.135126	-1.242396

C	-3.694993	-1.866742	-2.322437
C	-2.491701	-2.186249	-2.983803
C	-2.432094	-3.165136	-3.981665
C	-3.583422	-3.867900	-4.346054
C	-4.789790	-3.583373	-3.703011
C	-4.837429	-2.606440	-2.701160
C	-4.664322	1.764194	-0.759205
C	-6.006232	1.934786	-1.168154
C	-6.591887	3.201024	-1.289473
C	-5.852921	4.342105	-0.973680
C	-4.532008	4.202864	-0.539540
C	-3.954982	2.933899	-0.428513
H	0.046575	1.601672	3.942021
H	1.331242	2.695537	2.169595
H	-4.001340	-1.199953	2.919046
H	-6.077386	-0.500578	0.778746
H	-5.631919	-2.142101	1.212152
H	-7.718578	-1.500157	2.367737
H	-6.439648	-1.804068	3.539043
H	-7.400431	0.942065	2.569951
H	-7.642317	0.264033	4.179235
H	-5.732544	1.919398	4.105961
H	-5.203236	0.317124	4.614050
H	-4.941037	1.483794	1.785294
H	-3.637892	1.308735	2.947440
H	-1.833878	-2.557417	-0.164748
H	-4.096958	-3.580183	1.616190
H	-4.194575	-3.360422	-0.139577
H	-3.721858	-5.726856	0.438863
H	-2.388770	-5.023969	-0.471855
H	-2.498293	-5.417873	2.571789
H	-1.484601	-6.323701	1.451194
H	-0.199036	-4.491372	2.619851
H	-0.213851	-4.284027	0.871311
H	-1.981155	-2.796623	2.887851
H	-0.627557	-2.127335	1.984037
H	-6.618310	1.061994	-1.405827
H	-7.625113	3.292703	-1.618981
H	-6.302603	5.328546	-1.057942
H	-3.947214	5.086498	-0.290951
H	-2.927483	2.861528	-0.078874
H	-5.791488	-2.420506	-2.206639
H	-5.692928	-4.126907	-3.974592
H	-3.539825	-4.631338	-5.119200
H	-1.486268	-3.379711	-4.476817
H	-1.581320	-1.651636	-2.720746
H	-1.621542	0.838652	-2.924904
H	-1.478212	2.278761	-1.886465
H	-0.007359	1.518490	-2.567027
H	-2.280893	-0.652731	5.433362
H	-0.569751	-0.249391	5.120589

H	-1.803973	1.048071	5.178124
H	3.137979	4.452236	-3.216038
H	3.967618	2.309784	-2.524653
H	-0.833030	3.854658	0.613551
H	-1.539571	6.101325	0.001459
H	1.591099	6.339054	-2.935177
H	-0.353302	7.377541	-1.787992
H	3.661768	0.312064	-2.644443
H	4.957058	0.746045	-1.562745
H	2.725854	-0.786028	0.789073
H	2.454134	0.845846	1.282635
C	4.161797	-2.288054	2.020579
H	6.490627	-1.857814	3.295127
H	7.784283	0.218138	3.577154
H	6.947845	2.325677	2.594014
C	4.721471	2.808880	1.229400
C	2.110778	-2.273068	-2.347896
H	3.378255	-4.514912	-2.176103
H	5.733332	-5.043363	-1.652582
H	7.319323	-3.210655	-1.200362
C	6.928314	-0.559113	-1.169951
H	1.910096	-3.218939	-2.867335
C	1.160708	-2.250004	-1.137517
C	1.747871	-1.177709	-3.366373
C	8.177269	-1.021199	-0.397434
C	7.362473	0.004702	-2.542089
H	6.504139	0.257117	-0.578420
C	5.727365	3.514917	0.299752
H	3.799279	2.689136	0.664182
C	4.382917	3.706144	2.439032
C	5.013890	-3.555214	2.213671
C	3.022020	-2.275207	3.064353
H	3.715675	-2.381892	1.026033
H	2.430731	-3.197347	3.002499
H	3.435541	-2.205918	4.078087
H	2.341906	-1.427692	2.926755
H	4.401609	-4.439037	2.001790
H	5.870529	-3.572161	1.533444
H	5.381751	-3.658272	3.241502
H	3.959903	4.659657	2.100612
H	3.653077	3.223926	3.100082
H	5.271969	3.929244	3.040232
H	5.313930	4.465468	-0.056260
H	6.671031	3.732343	0.813810
H	5.960599	2.901781	-0.577571
H	8.804527	-0.151423	-0.171990
H	7.912014	-1.495506	0.551602
H	8.792347	-1.718708	-0.978305
H	8.103081	0.803987	-2.415326
H	7.818721	-0.785134	-3.150933
H	6.520230	0.411095	-3.112564

H 0.768396 -1.404978 -3.803314
H 1.669995 -0.189002 -2.908797
H 2.472693 -1.132697 -4.187737
H 0.128270 -2.401171 -1.467709
H 1.412391 -3.048523 -0.430154
H 1.205098 -1.290547 -0.619156

UPdPh₂, conformer of **2,7-bis-(N,N-dimesitylamino)N2PhosPdPh₂**: B3LYP/6-31G(d)/SDD for pre-reductive elimination step. C-Pd-C angle=82.64°

Processing: pdph2namphosme126dsdbe.log
PG=C01

Method	BasisSet	Imaginary Freqs
RB3LYP	GenECP	0

HF Energy
-3912.3513396

ZPE	E298	S298	Squasihar	Equasihar	Strans	Srot
1002.11972	1059.055	486.017	414.344	1059.372	47.314	42.322

Processing: pdph2namphosme126dsdbe.log
185
C 3.386548 -4.340834 -2.566033
C 2.740591 -2.958152 -2.810577
C 3.819091 -1.895031 -3.112035
C 4.712955 -2.318091 -4.290292
C 5.345161 -3.696921 -4.053763
C 4.268743 -4.750679 -3.759123
P 1.705872 -2.413766 -1.339296
C 0.219720 -3.587247 -1.194384
C -0.384730 -4.102715 -2.516425
C -1.650945 -4.941129 -2.261283
C -1.383449 -6.101230 -1.292560
C -0.776638 -5.589919 0.021744
C 0.492461 -4.754785 -0.220682
C 0.849014 -0.814216 -1.790098
C 0.564126 0.100879 -0.734968
C -0.171724 1.293267 -0.961980
C -0.650622 1.493175 -2.265137
C -0.377034 0.643991 -3.324119
C 0.380255 -0.505135 -3.086509
C -0.616037 2.315784 0.054224
C 0.136764 3.448921 0.436460
C -0.420148 4.316476 1.429934
C -1.653932 4.106531 1.984411
C -2.458810 3.020073 1.576213
C -1.945392 2.123632 0.587783
C -2.776258 1.039942 0.184698

C	-4.042736	0.812463	0.731805
C	-4.524762	1.728868	1.716701
C	-3.750861	2.791344	2.110976
N	1.406571	3.781548	-0.069562
C	1.725672	3.661303	-1.508984
C	2.190968	4.944573	-2.193665
C	3.510719	5.061849	-2.683921
C	3.902353	6.240235	-3.331909
C	3.027500	7.311745	-3.517738
C	1.718724	7.169624	-3.049232
C	1.285490	6.011280	-2.396574
C	4.529749	3.945456	-2.557782
C	3.481960	8.587085	-4.188799
C	-0.154919	5.932599	-1.941170
N	-4.823231	-0.274807	0.341785
C	-4.199928	-1.466453	-0.250785
C	-4.704309	-2.793096	0.304849
C	-4.413275	-3.142746	1.644797
C	-4.848505	-4.373555	2.143635
C	-5.567538	-5.280233	1.358154
C	-5.834394	-4.924900	0.035574
C	-5.416453	-3.701894	-0.506841
O	1.064315	-0.269187	0.501119
C	0.425921	0.144466	1.732903
O	0.669167	-1.389833	-4.086467
C	0.248849	-1.100478	-5.411830
C	-3.630737	-2.221294	2.554389
C	-6.049892	-6.592416	1.931434
C	-5.742214	-3.415846	-1.960351
C	2.320432	4.670409	0.681889
C	2.644472	4.266197	2.112680
C	3.211962	2.999649	2.370837
C	3.481395	2.618243	3.690065
C	3.243742	3.474019	4.767665
C	2.762305	4.754815	4.487567
C	2.472378	5.173369	3.183647
C	3.606779	2.064870	1.249728
C	2.046352	6.616328	2.982829
C	3.549566	3.044768	6.184427
C	-6.270386	-0.308429	0.577465
C	-7.081968	0.902406	0.118013
C	-8.073181	1.434401	0.974572
C	-8.847110	2.519095	0.546279
C	-8.680195	3.098716	-0.712097
C	-7.720490	2.539306	-1.557639
C	-6.924830	1.454675	-1.172662
C	-8.358502	0.860288	2.349832
C	-9.498888	4.294350	-1.139898
C	-5.935742	0.907861	-2.178573
H	-0.761181	0.873626	-4.309937
H	-1.265153	2.371378	-2.443746

H	2.084803	-3.037097	-3.679789
H	3.995100	-4.318423	-1.656953
H	2.612880	-5.101879	-2.410978
H	4.733497	-5.722680	-3.551171
H	3.639418	-4.886943	-4.652136
H	6.032662	-3.639330	-3.197978
H	5.945139	-3.995497	-4.923264
H	5.491587	-1.561398	-4.452904
H	4.109266	-2.348481	-5.210142
H	4.441570	-1.749502	-2.219165
H	3.346839	-0.930164	-3.331541
H	-0.530731	-2.939655	-0.715077
H	1.281454	-5.402978	-0.624469
H	0.874571	-4.360061	0.727174
H	-0.542301	-6.430879	0.687124
H	-1.522748	-4.974732	0.546264
H	-0.688182	-6.813243	-1.762036
H	-2.312142	-6.651014	-1.094416
H	-2.040215	-5.320710	-3.215472
H	-2.434153	-4.295277	-1.837098
H	0.354710	-4.726640	-3.037557
H	-0.615195	-3.268315	-3.184879
H	0.837304	-0.512661	2.496658
H	0.672564	1.178484	1.969091
H	-0.655586	0.018634	1.656771
H	0.623737	-1.921826	-6.025062
H	-0.844988	-1.060484	-5.489726
H	0.674063	-0.155104	-5.770593
H	-2.034739	4.796502	2.734369
H	0.146825	5.179259	1.741228
H	-2.418248	0.388897	-0.600032
H	-4.127977	3.478988	2.864985
H	-5.501097	1.587422	2.160874
H	3.251870	4.672465	0.106684
H	1.966307	5.708706	0.651888
H	2.476767	2.876899	-1.651395
H	0.827835	3.316355	-2.013997
H	4.921950	6.318244	-3.704623
H	1.009119	7.981111	-3.201351
H	2.610791	5.455704	5.306911
H	3.896878	1.630681	3.874221
H	-6.640313	-1.197871	0.055807
H	-6.474142	-0.498117	1.638183
H	-9.604849	2.918259	1.217913
H	-7.587407	2.953704	-2.555449
H	-4.300189	-1.453393	-1.343514
H	-3.128826	-1.400421	-0.042967
H	-6.382626	-5.618516	-0.598899
H	-4.614309	-4.632818	3.174535
H	-9.159704	1.424418	2.836887
H	-8.678295	-0.188057	2.305380

H	-7.485826	0.898662	3.014850
H	-6.117190	1.346465	-3.165303
H	-4.900479	1.128737	-1.896510
H	-6.016725	-0.180414	-2.283620
H	-9.675374	4.293814	-2.221217
H	-10.472192	4.315096	-0.637730
H	-8.987143	5.235228	-0.895938
H	-6.340056	-4.228834	-2.383442
H	-6.315952	-2.489744	-2.086665
H	-4.839730	-3.320921	-2.576657
H	-3.412627	-2.713112	3.507157
H	-2.673357	-1.921537	2.109429
H	-4.173387	-1.293993	2.768604
H	-5.285337	-7.059369	2.562797
H	-6.940950	-6.450043	2.557436
H	-6.315268	-7.301852	1.140831
H	-0.726121	6.785391	-2.321083
H	-0.648686	5.019094	-2.295351
H	-0.248446	5.927584	-0.849864
H	5.497389	4.266478	-2.955380
H	4.689283	3.635663	-1.518299
H	4.230121	3.047799	-3.113096
H	2.674057	9.044370	-4.770895
H	3.809086	9.331465	-3.450165
H	4.325777	8.406931	-4.863417
H	2.008425	7.135342	3.945619
H	2.751952	7.164003	2.345778
H	1.056854	6.725463	2.521919
H	4.050566	1.149440	1.646837
H	2.760463	1.782873	0.619663
H	4.349682	2.539503	0.592059
H	3.578490	1.953736	6.268786
H	4.524733	3.426732	6.516542
H	2.799504	3.422448	6.889015
Pd	2.749918	-2.039443	0.806098
C	4.186214	-3.432021	0.842090
C	5.461986	-3.110375	0.347084
C	6.470276	-4.077625	0.265860
C	6.230487	-5.385086	0.693597
C	4.975956	-5.709629	1.213841
C	3.967735	-4.742698	1.294352
H	5.677936	-2.097493	0.014914
H	7.447179	-3.802474	-0.127264
H	7.013623	-6.136708	0.634041
H	4.778684	-6.719036	1.569600
H	3.006622	-5.018811	1.718613
C	3.362710	-1.734973	2.740060
C	4.596631	-1.172333	3.114422
C	4.921317	-0.932018	4.453993
C	4.018864	-1.262752	5.468839
C	2.795464	-1.841817	5.127450

C 2.481178 -2.080684 3.784002
H 5.332838 -0.931785 2.350428
H 5.886535 -0.496111 4.705544
H 4.274271 -1.088782 6.511527
H 2.088304 -2.121586 5.906239
H 1.528724 -2.558752 3.555109

VPdPh₂, conformer of **2,7-bis-(N,N-di-t-butylamino)N2PhosPdPh₂**: B3LYP/6-31G(d)/SDD for pre-reductive elimination step. C-Pd-C angle=83.28^o
‘
Processing: pdph2namphostbu46dsdbe.log
PG=C01

Method BasisSet Imaginary Freqs
RB3LYP GenECP 0

HF Energy
-2988.1028231

ZPE	E298	S298	Squasihar	Equasihar	Strans	Srot
801.13812	843.530	363.356	322.749	843.721	46.505	40.402

Processing: pdph2namphostbu46dsdbe.log
145
C -5.729460 1.717118 1.554884
C -4.577732 2.218699 0.893956
C -3.680317 1.305232 0.254090
C -3.998703 -0.084698 0.319107
C -5.134389 -0.564255 0.954415
C -6.003113 0.370836 1.582497
C -4.288502 3.604054 0.848920
C -3.185375 4.057907 0.174930
C -2.277962 3.183924 -0.501587
C -2.502209 1.803004 -0.425919
C -1.652072 0.785241 -1.143816
C -0.448678 0.207368 -0.680454
C 0.280268 -0.748119 -1.428321
C -0.262720 -1.165431 -2.666425
C -1.483777 -0.659521 -3.115477
C -2.153359 0.287834 -2.352099
P 1.871806 -1.387921 -0.687139
C 1.333986 -3.068922 0.014059
C 2.183835 -3.471873 1.239213
C 1.637293 -4.745758 1.905936
C 1.521553 -5.907336 0.908763
C 0.677640 -5.505460 -0.308755
C 1.222726 -4.235826 -0.988633
O 0.078055 0.584484 0.556877
C -0.670964 0.160174 1.718531
O 0.441103 -2.095053 -3.376433

C	-0.044846	-2.509518	-4.645897
N	-1.179534	3.757324	-1.243492
C	-0.051045	4.344957	-0.399476
C	1.304152	3.790284	-0.901096
N	-5.477543	-1.962700	1.019763
C	-6.086103	-2.500633	-0.247353
C	-7.216773	-1.539580	-0.688596
C	3.063107	-1.708728	-2.103005
C	3.333779	-0.396668	-2.871064
C	4.343130	-0.610870	-4.011954
C	5.651476	-1.230678	-3.501211
C	5.379771	-2.535670	-2.740405
C	4.385292	-2.320603	-1.585564
C	-4.715409	-2.797316	2.017706
C	-3.593205	-3.662041	1.393924
C	-1.581903	4.382914	-2.563437
C	-2.399827	5.702729	-2.464228
C	-5.673835	-3.704672	2.833879
C	-4.047984	-1.874171	3.065147
C	-5.088853	-2.622859	-1.430257
C	-6.755961	-3.874840	-0.046474
C	-2.467888	3.393523	-3.350196
C	-0.354307	4.628396	-3.467740
C	-0.183757	3.896738	1.073081
C	0.019179	5.892088	-0.357079
H	-1.920070	-0.994171	-4.048459
H	-3.106522	0.664085	-2.707186
H	0.695699	-3.213584	-5.028965
H	-0.133871	-1.662206	-5.336779
H	-1.015205	-3.014535	-4.560866
H	-1.684822	0.558348	1.689777
H	-0.134424	0.564466	2.576112
H	-0.695094	-0.934325	1.769861
H	2.598005	-2.420018	-2.789826
H	2.397608	0.006859	-3.274650
H	3.728428	0.354262	-2.173680
H	4.541107	0.345843	-4.512483
H	3.898498	-1.274276	-4.769371
H	6.150576	-0.520639	-2.826569
H	6.339688	-1.411217	-4.337127
H	6.315818	-2.947484	-2.342693
H	4.976532	-3.286146	-3.437664
H	3.224497	-3.640655	0.932644
H	2.208225	-2.646044	1.958714
H	0.319258	-2.852405	0.382395
H	0.643734	-4.533615	2.329495
H	2.281604	-5.026941	2.748679
H	-0.359572	-5.325877	0.012252
H	1.089357	-6.789600	1.398010
H	2.527980	-6.197292	0.572065
H	0.640314	-6.326434	-1.036820

H	2.215222	-4.455658	-1.405448
H	0.584633	-3.956020	-1.831336
H	4.835824	-1.660536	-0.837215
H	4.195877	-3.279461	-1.088978
H	-2.975125	5.120473	0.151363
H	-4.955404	4.301386	1.350774
H	-6.397555	2.424137	2.041405
H	-6.886341	-0.009824	2.085889
H	-3.321301	-0.794216	-0.137988
H	-2.763167	3.855099	-4.299054
H	-1.920097	2.476437	-3.573802
H	-3.384365	3.136650	-2.811478
H	-0.705378	4.951441	-4.454452
H	0.314774	5.406832	-3.097401
H	0.220660	3.707313	-3.599024
H	-2.595865	6.081451	-3.474736
H	-3.372126	5.533654	-1.992320
H	-1.883109	6.490611	-1.915556
H	0.849963	6.183786	0.295658
H	0.201656	6.352398	-1.330338
H	-0.892321	6.328017	0.065643
H	2.107453	4.115208	-0.230756
H	1.287690	2.697093	-0.896256
H	1.557793	4.127317	-1.906765
H	0.706705	4.221520	1.618089
H	-1.055701	4.332780	1.568407
H	-0.233012	2.812531	1.158644
H	-5.607375	-3.010371	-2.316068
H	-4.666503	-1.650867	-1.698775
H	-4.263820	-3.303856	-1.204964
H	-7.715571	-1.945240	-1.575989
H	-7.963615	-1.429983	0.104801
H	-6.841597	-0.545550	-0.944903
H	-7.266484	-4.150422	-0.975803
H	-6.042341	-4.672732	0.172602
H	-7.505001	-3.841918	0.749333
H	-5.140614	-4.122555	3.695934
H	-6.517860	-3.115666	3.207136
H	-6.068177	-4.546361	2.265329
H	-3.573199	-2.499694	3.828086
H	-3.277847	-1.232758	2.631712
H	-4.781100	-1.233086	3.564237
H	-3.051380	-4.198009	2.183109
H	-3.979838	-4.413197	0.699861
H	-2.869955	-3.039897	0.854656
Pd	2.431627	0.245292	1.012105
C	4.397134	-0.031816	1.279272
C	5.311257	0.713347	0.514230
C	6.688040	0.477413	0.600279
C	7.184425	-0.498373	1.467132
C	6.289238	-1.225931	2.253861

C 4.912700 -0.990432 2.166162
H 4.952540 1.487098 -0.160761
H 7.372347 1.065920 -0.007996
H 8.253918 -0.680130 1.537872
H 6.660882 -1.976662 2.948686
H 4.240653 -1.560836 2.801612
C 2.654797 1.578985 2.546718
C 3.206629 2.862795 2.389139
C 3.216861 3.791377 3.435022
C 2.688522 3.453025 4.683585
C 2.163137 2.174684 4.875549
C 2.156875 1.251307 3.822403
H 3.645863 3.146793 1.435367
H 3.647346 4.778660 3.276687
H 2.700613 4.171623 5.499598
H 1.767621 1.887896 5.848349
H 1.765455 0.252431 4.014001

Conformer of **N2PhosPd-naphthyl, o-tolyl**: B3LYP/6-31G(d)/SDD for pre-reductive elimination step. C-Pd-C angle=82.87^{o}

Processing: pdnaptolnamphos6dsdbe.log
Method Basis Set Imaginary Freqs
RB3LYP GenECP

HF
-3633.53741200

Processing: pdnaptolnamphos6dsdbe.log
Frequency job incomplete: pdnaptolnamphos6dsdbe.log
158
C 3.763466 -2.804710 -1.647180
C 4.408500 -3.128637 -0.447141
C 4.254560 -4.421351 0.071273
C 3.469259 -5.368056 -0.587776
C 2.828985 -5.035697 -1.784879
C 2.979061 -3.751418 -2.311807
C 5.267983 -2.114810 0.304549
N 5.836963 -1.051678 -0.507076
C 6.991621 -1.431954 -1.307633
C 8.338581 -1.385850 -0.591313
C 9.422550 -2.096252 -1.125860
C 10.676288 -2.050618 -0.517360
C 10.863557 -1.296906 0.644580
C 9.788849 -0.592692 1.187989
C 8.534375 -0.636505 0.573459
C 5.258664 0.216538 -0.588956
C 6.008095 1.303101 -1.139798
C 5.444704 2.549341 -1.247645
C 4.127768 2.818812 -0.798130

C	3.372345	1.761590	-0.197913
C	3.964328	0.469456	-0.133710
C	3.542363	4.099412	-0.908526
C	2.265228	4.329478	-0.467863
C	1.473005	3.299908	0.125154
C	2.032137	2.016662	0.271255
C	1.365584	0.928291	1.066943
C	0.251546	0.131399	0.711164
C	-0.346715	-0.774219	1.625467
C	0.241200	-0.907360	2.905826
C	1.381457	-0.180376	3.254216
C	1.916977	0.711376	2.338128
P	-1.847894	-1.731559	1.057876
C	-2.984336	-1.920313	2.545776
C	-3.360073	-0.544039	3.134485
C	-4.307414	-0.691565	4.338062
C	-5.565570	-1.491968	3.972532
C	-5.193254	-2.858492	3.380896
C	-4.254511	-2.716212	2.169424
O	-0.328778	0.226057	-0.549045
C	0.554107	0.123970	-1.692295
O	-0.341583	-1.786001	3.772242
C	0.179044	-1.912589	5.088166
N	0.140825	3.604892	0.517456
C	-0.242421	3.468837	1.937678
C	0.134111	4.645352	2.830540
C	-0.854562	5.477206	3.370665
C	-0.519257	6.552894	4.196604
C	0.818983	6.815507	4.491064
C	1.816445	5.994746	3.957428
C	1.476409	4.918701	3.137475
C	-1.117487	-3.448473	0.687035
C	-0.880806	-4.375332	1.897766
C	-0.198399	-5.688958	1.474119
C	-0.991562	-6.416472	0.380115
C	-1.231496	-5.495788	-0.824467
C	-1.916995	-4.182004	-0.412583
Pd	-2.557810	-0.610061	-0.972322
C	-2.906382	0.199273	-2.835247
C	-2.420449	-0.440392	-4.003944
C	-2.615432	0.158536	-5.259627
C	-3.312779	1.359003	-5.396198
C	-3.816933	1.983797	-4.256210
C	-3.611786	1.404500	-3.000652
C	-4.483458	-1.169057	-1.131210
C	-5.505239	-0.363954	-0.520609
C	-6.874893	-0.807383	-0.547168
C	-7.200938	-2.026936	-1.196655
C	-6.212685	-2.769612	-1.799164
C	-4.862877	-2.337121	-1.770722
C	-0.518681	4.765951	-0.101235

C	-0.506079	4.796813	-1.617858
C	-0.285423	6.009338	-2.283716
C	-0.307502	6.074334	-3.677756
C	-0.544519	4.919253	-4.424716
C	-0.772157	3.706631	-3.769805
C	-0.758883	3.647718	-2.375249
H	1.842814	-0.289235	4.227771
H	2.788169	1.295639	2.619503
H	-2.442075	-2.481282	3.310692
H	-4.784914	-2.211082	1.356031
H	-3.987764	-3.712808	1.798791
H	-6.096887	-3.402978	3.079076
H	-4.702138	-3.468441	4.154761
H	-6.153221	-0.926638	3.235776
H	-6.204568	-1.620758	4.855944
H	-4.582130	0.302809	4.713300
H	-3.777217	-1.200476	5.157692
H	-3.855051	0.059161	2.363262
H	-2.457354	-0.000920	3.437634
H	-0.134710	-3.197521	0.260097
H	-2.932045	-4.400054	-0.055051
H	-2.032568	-3.527746	-1.282246
H	-1.839521	-6.006473	-1.582492
H	-0.266089	-5.263794	-1.297920
H	-1.960458	-6.741071	0.788132
H	-0.463960	-7.326934	0.067121
H	-0.074090	-6.337114	2.351960
H	0.812279	-5.466272	1.101984
H	-1.844811	-4.613395	2.368711
H	-0.278367	-3.868332	2.655316
H	-4.028357	1.904668	-2.130808
H	-4.364279	2.920169	-4.338116
H	-3.461901	1.796099	-6.380813
H	-2.228161	-0.340018	-6.147667
C	-1.742626	-1.797006	-3.963734
C	-5.224104	0.870392	0.132669
C	-7.873844	-0.012064	0.080218
H	-8.237989	-2.354295	-1.216318
H	-6.460987	-3.697693	-2.310016
H	-4.118267	-2.950024	-2.268702
H	-0.096521	0.182771	-2.563143
H	1.267600	0.946146	-1.705213
H	1.079106	-0.837172	-1.668144
H	-0.467765	-2.632795	5.591950
H	1.208065	-2.293400	5.080171
H	0.147849	-0.956722	5.625230
H	4.121887	4.910960	-1.342791
H	1.854322	5.326029	-0.551548
H	3.365812	-0.345423	0.250845
H	6.024073	3.364964	-1.674550
H	7.036646	1.155825	-1.448646

H	-1.561228	4.720037	0.242269
H	-0.125077	5.721058	0.283826
H	-1.330126	3.329846	1.968762
H	0.203213	2.556887	2.330636
H	-1.900282	5.277346	3.145331
H	-1.303042	7.184388	4.607083
H	1.084414	7.651795	5.132424
H	2.861345	6.190783	4.184464
H	2.259707	4.286243	2.728459
H	-0.090148	6.910424	-1.704831
H	-0.132725	7.023928	-4.177487
H	-0.557893	4.963676	-5.510702
H	-0.981263	2.807728	-4.341141
H	-0.950580	2.706667	-1.869674
H	6.823883	-2.451632	-1.679705
H	7.024344	-0.800595	-2.202480
H	9.281810	-2.691712	-2.026239
H	11.505370	-2.608264	-0.945269
H	11.838706	-1.263204	1.122921
H	9.923920	-0.006112	2.093130
H	7.699523	-0.088533	1.000138
H	6.096346	-2.649416	0.789196
H	4.692565	-1.660573	1.118687
H	4.762980	-4.692382	0.994765
H	3.370049	-6.369101	-0.176172
H	2.228887	-5.775211	-2.307841
H	2.493605	-3.487068	-3.247764
H	3.886845	-1.810816	-2.067038
C	-6.215267	1.621273	0.727681
H	-4.191130	1.209761	0.149273
H	-5.968103	2.561857	1.213836
C	-7.558429	1.173723	0.704323
H	-8.904021	-0.361773	0.054164
H	-8.336881	1.769157	1.174719
H	-2.485929	-2.605389	-3.933165
H	-1.124030	-1.961633	-4.854742
H	-1.108929	-1.919152	-3.079438

X_2PdPh_2, conformer of associated **bis(Phos)$PdPh_2$**, N-Phos structure without 2,7-amino substituents: B3LYP/6-31G(d)/SDD for pre-reductive elimination step. P-Pd distances=2.557, 2.621 Å, C-Pd-C angle=79.94^o.

Processing: pddiphdi0namphos6dsdbe.log
PG=C01

Method	BasisSet	Imaginary Freqs
RB3LYP	GenECP	0

HF Energy
-3905.7121378

ZPE	E298	S298	Squasihar	Equasihar	Strans	Srot
879.27856	927.234	411.638	360.324	927.465	47.076	41.499

Processing: pddiphdi0namphos6dsdbe.log
Frequency job incomplete: pddiphdi0namphos6dsdbe.log
163

C	-2.977758	-0.214903	2.985172
C	-1.757075	-0.255455	2.040855
C	-1.217458	-1.684388	1.838666
C	-0.964218	-2.398800	3.177834
C	-2.205458	-2.381308	4.080108
C	-2.684346	-0.942492	4.311771
P	-1.758463	0.764393	0.434678
Pd	0.308110	0.277158	-0.990765
C	-0.863779	-0.962751	-2.148489
C	-0.918340	-2.334171	-1.840666
C	-1.673278	-3.235882	-2.601159
C	-2.408664	-2.788242	-3.700136
C	-2.370905	-1.430474	-4.025387
C	-1.605040	-0.542072	-3.265184
C	-3.333037	0.543882	-0.565488
C	-4.294548	-0.483105	-0.454695
C	-5.491468	-0.472230	-1.217303
C	-5.639989	0.515485	-2.192625
C	-4.665626	1.480915	-2.410246
C	-3.529668	1.489857	-1.601597
C	-6.619571	-1.433884	-1.028729
C	-7.000014	-2.242840	-2.086751
C	-8.083611	-3.144925	-1.980719
C	-8.792682	-3.238609	-0.806230
C	-8.458902	-2.418373	0.304845
C	-7.366339	-1.492366	0.197979
C	-9.193491	-2.486536	1.520060
C	-8.879545	-1.674460	2.585672
C	-7.815186	-0.747960	2.476948
C	-7.080185	-0.659648	1.315038
H	-6.430607	-2.198242	-3.011070
O	-4.046542	-1.484582	0.446459
C	-4.234455	-2.848273	0.038002
O	-2.527509	2.403722	-1.766560
C	-2.581134	3.295762	-2.869469
C	-2.042001	2.484221	1.252955
C	-3.510655	2.938360	1.402369
C	-3.612302	4.170398	2.321153
C	-2.738576	5.329393	1.819108
C	-1.286168	4.879819	1.599486
C	-1.212820	3.649044	0.678332
C	1.547104	0.032594	-2.625468
C	2.625139	-0.861113	-2.720154
C	3.432680	-0.930050	-3.862295

C	3.181502	-0.102217	-4.956904
C	2.106395	0.787569	-4.895222
C	1.303642	0.843301	-3.751995
P	2.254438	1.514123	0.254881
C	2.383221	3.083206	-0.845288
C	2.518161	4.476640	-0.199116
C	2.237869	5.581713	-1.235997
C	3.150796	5.460597	-2.464464
C	3.065381	4.058198	-3.083326
C	3.351137	2.959253	-2.045428
C	1.997587	2.052812	2.069276
C	1.776147	0.802141	2.951164
C	1.324391	1.183038	4.371071
C	2.335232	2.116197	5.051325
C	2.631856	3.337830	4.171807
C	3.068277	2.934861	2.749826
C	3.927433	0.678669	0.422962
C	3.962005	-0.723763	0.648561
C	5.180996	-1.428695	0.800154
C	6.368592	-0.702727	0.667280
C	6.378756	0.673267	0.490371
C	5.163826	1.364711	0.426211
O	2.741926	-1.313501	0.796053
C	2.486956	-2.665196	0.400860
C	5.294246	-2.854127	1.243965
C	5.842913	-3.865965	0.385824
C	6.010585	-5.197765	0.896123
C	5.623421	-5.487623	2.231653
C	5.092200	-4.504593	3.033862
C	4.935845	-3.189174	2.538279
C	6.553186	-6.200606	0.047405
C	6.906141	-5.916177	-1.252112
C	6.725823	-4.608775	-1.763400
C	6.208592	-3.611075	-0.965917
O	5.120546	2.728753	0.391011
C	6.337889	3.458516	0.394976
H	4.530383	-2.418949	3.188993
H	-7.579595	-0.098497	3.315840
H	6.994071	-4.392202	-2.793888
H	7.324735	1.197946	0.449490
H	7.312352	-1.231268	0.769221
H	1.371841	3.043000	-1.275907
H	4.379585	3.063732	-1.692326
H	3.263922	1.980068	-2.516740
H	3.771584	3.965150	-3.918433
H	2.063177	3.902968	-3.508228
H	4.189941	5.658628	-2.160058
H	2.893200	6.227740	-3.206718
H	2.355969	6.567396	-0.766004
H	1.187387	5.514463	-1.557591
H	3.530224	4.604200	0.198732

H	1.824802	4.594053	0.641365
H	1.054878	2.616256	2.046587
H	4.007330	2.370467	2.809029
H	3.280226	3.832146	2.168992
H	3.411365	3.958549	4.633392
H	1.731602	3.967997	4.108396
H	3.270388	1.564349	5.228004
H	1.966671	2.431325	6.036173
H	1.180274	0.274500	4.970895
H	0.344426	1.682137	4.322404
H	2.720510	0.246784	3.017735
H	1.059950	0.120967	2.487311
H	-1.579375	0.501970	-3.564075
H	-2.931267	-1.060140	-4.882071
H	-2.989388	-3.485692	-4.299370
H	-1.676062	-4.292067	-2.335713
H	-0.360308	-2.724476	-0.993612
H	2.852064	-1.522149	-1.892395
H	4.257676	-1.640042	-3.893788
H	3.805380	-0.155411	-5.845971
H	1.887228	1.437378	-5.740839
H	0.468793	1.541098	-3.742940
H	1.486365	-2.661232	-0.032913
H	3.201789	-3.012183	-0.348106
H	2.520552	-3.330902	1.267035
H	6.051798	4.511609	0.372481
H	6.921927	3.260188	1.302437
H	6.948612	3.231691	-0.487967
H	5.754087	-6.499734	2.607258
H	4.799336	-4.728766	4.056059
H	6.070063	-2.612344	-1.367095
H	6.680378	-7.204564	0.445901
H	7.317073	-6.693944	-1.890048
H	-6.551606	0.531505	-2.782836
H	-4.803948	2.219536	-3.189777
H	-3.547772	-3.438421	0.649683
H	-5.256935	-3.182991	0.224019
H	-3.975893	-2.976240	-1.016127
H	-1.651910	3.866907	-2.833870
H	-2.636835	2.750483	-3.819261
H	-3.433973	3.982813	-2.792514
H	-1.656724	2.302945	2.267635
H	-3.912262	3.194316	0.414871
H	-4.140278	2.132008	1.790981
H	-4.659881	4.490124	2.397431
H	-3.297292	3.891092	3.337878
H	-3.146339	5.699888	0.866777
H	-2.777831	6.170259	2.523595
H	-0.695352	5.701117	1.172566
H	-0.829010	4.639526	2.571787
H	-1.596983	3.915721	-0.309287

H -0.174031 3.345216 0.532228
H -0.956063 0.295106 2.556800
H -1.925903 -2.262681 1.242864
H -0.287415 -1.636615 1.261006
H -0.641265 -3.431218 2.988059
H -0.133237 -1.905435 3.704170
H -3.011509 -2.960677 3.606215
H -1.988410 -2.870953 5.038528
H -3.585926 -0.934239 4.938396
H -1.911382 -0.392608 4.870119
H -3.836624 -0.683382 2.498688
H -3.252301 0.821714 3.208826
H -8.343472 -3.770089 -2.830767
H -9.621429 -3.936401 -0.711897
H -6.276082 0.063541 1.237631
H -10.016576 -3.194145 1.589527
H -9.450655 -1.736579 3.508155

Q--QPdPh$_2$, more stable dissociated form of **bis(N,N-dibenzyl-2-aminoNPhos)PdPh$_2$**, one phosphine ligand dissociated to loose complex: B3LYP/6-31G(d)/SDD for pre-reductive elimination step. P-Pd distance=6.396 Å, C-Pd-C angle=86.17°. ($E_{e,rel}$=0.00 kcal/mol, $G_{298,rel}$=0.00 kcal/mol)

Processing: pddiphdi2namphos6dsdbe.log
PG=C01

Method	BasisSet	Imaginary Freqs
RB3LYP	GenECP	0

HF Energy
-5097.8106317

ZPE	E298	S298	Squasihar	Equasihar	Strans	Srot
1173.68819	1239.099	570.024	464.087	1239.504	47.927	43.806

Processing: pddiphdi2namphos6dsdbe.log
219
C 10.520518 0.428964 -2.481339
C 9.680852 -0.681380 -2.660372
C 10.133469 -1.734633 -3.467126
C 11.390722 -1.686770 -4.074224
C 12.217518 -0.578967 -3.882421
C 11.777693 0.480250 -3.084313
C 8.313656 -0.745262 -1.994603
N 8.323455 -1.117485 -0.559826
C 8.799417 -2.482734 -0.308844
C 7.886797 -3.547011 -0.894712
C 8.420940 -4.636039 -1.592617
C 7.590976 -5.648815 -2.081650
C 6.212339 -5.578906 -1.880559
C 5.670131 -4.492736 -1.186672

C	6.500917	-3.485218	-0.696819
C	8.762302	-0.118932	0.358273
C	7.974846	1.005225	0.636954
C	8.459827	2.004992	1.550687
C	9.723898	1.826276	2.205082
C	10.476618	0.661329	1.917869
C	10.016526	-0.269792	1.021152
C	7.708832	3.172139	1.880000
C	8.184779	4.101082	2.779669
C	9.440255	3.923440	3.408144
C	10.189075	2.804893	3.123876
C	6.641316	1.218578	-0.014884
C	5.461026	0.525374	0.346938
C	4.230921	0.761453	-0.309496
C	4.220817	1.709686	-1.362792
C	5.365463	2.426877	-1.712309
C	6.549519	2.178424	-1.028638
P	2.536312	0.174788	0.202205
C	1.820255	-0.889698	-1.196955
C	2.851260	-1.450813	-2.195168
C	2.186353	-2.410571	-3.198237
C	1.010899	-1.744038	-3.928069
C	-0.011391	-1.176713	-2.931621
C	0.644568	-0.210761	-1.930190
O	5.468095	-0.438444	1.323406
C	6.009521	-0.138700	2.614860
O	3.028371	1.867248	-1.994823
C	2.865456	2.947332	-2.911177
H	7.588847	4.980699	3.008384
Pd	1.052233	1.816776	1.299047
C	-0.210991	2.777983	2.552858
C	0.139480	3.838093	3.406987
C	-0.738772	4.293768	4.395645
C	-1.999704	3.711248	4.542161
C	-2.374603	2.669007	3.692108
C	-1.493097	2.211643	2.704077
P	-4.036695	-2.041911	1.654669
C	-4.360390	-2.356265	3.500711
C	-4.576321	-1.004698	4.218678
C	-4.679235	-1.171704	5.743949
C	-5.778044	-2.170680	6.132720
C	-5.573183	-3.516365	5.423440
C	-5.470972	-3.348972	3.895936
C	-5.738610	-2.096382	0.865092
C	-6.284965	-0.827631	0.534895
C	-7.516960	-0.685435	-0.145143
C	-8.246364	-1.853098	-0.395771
C	-7.770112	-3.114046	-0.056291
C	-6.514252	-3.234577	0.550051
C	-8.061386	0.622296	-0.634600
C	-9.224193	1.168741	0.011283

C	-9.774465	2.416871	-0.431698
C	-9.149188	3.083007	-1.513393
C	-8.053298	2.542705	-2.135873
C	-7.488403	1.299015	-1.724456
C	-9.849129	0.537982	1.128034
C	-10.949753	1.093131	1.744000
C	-11.496986	2.314953	1.285763
C	-10.913071	2.960605	0.220235
H	-11.401864	0.586544	2.592897
N	-6.353201	0.782313	-2.411706
C	-5.507143	1.718981	-3.163279
C	-4.884987	2.837275	-2.347233
C	-4.862062	4.142398	-2.853514
C	-4.223164	5.170647	-2.156495
C	-3.601302	4.903662	-0.936063
C	-3.620865	3.603764	-0.420295
C	-4.253788	2.576479	-1.123171
O	-5.528823	0.254831	0.889096
C	-6.135337	1.355854	1.572483
O	-5.990366	-4.453130	0.879750
C	-6.756682	-5.620487	0.628948
C	-3.002152	-3.540602	1.094615
C	-3.070502	-4.910666	1.797003
C	-1.962596	-5.849386	1.282429
C	-2.021007	-6.013310	-0.243396
C	-1.960980	-4.651278	-0.950535
C	-3.053828	-3.696479	-0.439658
C	-6.516200	-0.500498	-3.130902
C	-7.199143	-0.402643	-4.488963
C	-6.460551	-0.519831	-5.673508
C	-7.080084	-0.426093	-6.921983
C	-8.456145	-0.209562	-7.003302
C	-9.206275	-0.092063	-5.830090
C	-8.583446	-0.190366	-4.585694
C	1.285597	3.582752	0.395822
C	2.459561	4.320679	0.610408
C	2.667247	5.537665	-0.052309
C	1.703865	6.038376	-0.931136
C	0.529615	5.310555	-1.142132
C	0.316532	4.096647	-0.479772
C	2.641396	-1.062382	1.629113
C	3.162967	-2.483980	1.346713
C	3.160452	-3.349571	2.620266
C	1.767828	-3.410246	3.262162
C	1.229449	-2.000676	3.545857
C	1.236410	-1.125142	2.280186
H	-8.371660	-3.986229	-0.280264
H	-9.208536	-1.767930	-0.894345
H	-2.001685	-3.135605	1.326835
H	-4.037772	-4.086389	-0.727054
H	-2.947393	-2.715907	-0.922355

H	-2.054944	-4.781241	-2.036969
H	-0.973268	-4.197966	-0.775260
H	-2.959097	-6.519924	-0.516116
H	-1.204844	-6.660529	-0.590568
H	-2.043785	-6.827824	1.774686
H	-0.979180	-5.441768	1.563712
H	-4.046459	-5.368969	1.620031
H	-2.965940	-4.789853	2.882052
H	-3.395781	-2.758404	3.851765
H	-6.430041	-2.975180	3.510926
H	-5.306043	-4.320496	3.423314
H	-6.393241	-4.205221	5.667587
H	-4.651212	-3.985934	5.798867
H	-6.758254	-1.758682	5.849176
H	-5.800888	-2.310639	7.221405
H	-4.863323	-0.197059	6.214678
H	-3.712936	-1.526865	6.132612
H	-5.503113	-0.545025	3.845219
H	-3.762616	-0.313733	3.969562
H	-0.616130	3.564671	-0.638515
H	-0.238618	5.691636	-1.811949
H	1.861601	6.987995	-1.436720
H	3.584270	6.095452	0.128075
H	3.220104	3.953296	1.294593
H	1.107781	4.320275	3.298231
H	-0.438053	5.110321	5.049338
H	-2.684804	4.069443	5.306904
H	-3.355422	2.208096	3.793464
H	-1.817365	1.399855	2.053927
H	-5.352658	1.776322	2.209436
H	-6.480469	2.120293	0.873213
H	-6.972997	1.021182	2.193470
H	-6.148193	-6.456464	0.979437
H	-7.705112	-5.607098	1.180967
H	-6.962449	-5.748039	-0.441547
H	-9.557662	4.030444	-1.857484
H	-7.614962	3.068716	-2.974102
H	-9.445020	-0.397122	1.499005
H	-11.312763	3.905874	-0.140328
H	-12.367438	2.739950	1.777980
H	-4.696923	1.104395	-3.577831
H	-6.022193	2.147086	-4.039251
H	-5.514649	-0.928349	-3.260140
H	-7.069687	-1.183148	-2.487628
H	-5.387740	-0.693729	-5.617198
H	-6.488017	-0.523431	-7.828483
H	-8.942404	-0.136936	-7.972599
H	-10.279751	0.070423	-5.884540
H	-9.176390	-0.102425	-3.679300
H	-5.346905	4.356406	-3.804463
H	-4.218227	6.178308	-2.564738

H	-3.104193	5.698605	-0.386635
H	-3.140013	3.391046	0.531075
H	-4.278052	1.570936	-0.714230
H	7.443701	2.734242	-1.298408
H	5.341524	3.165594	-2.503537
H	5.464581	-0.768812	3.323667
H	7.074985	-0.373592	2.667471
H	5.856390	0.914143	2.872994
H	1.815470	2.929241	-3.202554
H	3.087109	3.904194	-2.429565
H	3.497354	2.808378	-3.797674
H	1.392293	-1.742823	-0.650810
H	3.309235	-0.618086	-2.742167
H	3.664004	-1.964383	-1.668635
H	2.933646	-2.766771	-3.919125
H	1.820907	-3.300144	-2.662831
H	1.393907	-0.927259	-4.557887
H	0.528040	-2.460904	-4.604803
H	-0.820021	-0.661120	-3.465782
H	-0.481102	-2.007855	-2.383915
H	1.009799	0.674915	-2.458715
H	-0.096742	0.143005	-1.203714
H	3.310663	-0.586768	2.352826
H	2.525792	-2.973275	0.597070
H	4.172309	-2.433483	0.934477
H	3.516897	-4.359501	2.380235
H	3.875819	-2.932204	3.344534
H	1.077666	-3.932947	2.582558
H	1.797088	-3.997262	4.188919
H	0.212097	-2.049919	3.953129
H	1.852656	-1.521027	4.314514
H	0.499932	-1.517474	1.564525
H	0.890970	-0.112946	2.563255
H	10.633991	-1.129830	0.788207
H	11.440222	0.522339	2.402743
H	6.745103	3.329710	1.408934
H	11.153495	2.648601	3.601989
H	9.805060	4.665758	4.112617
H	9.822897	-2.651145	-0.683572
H	8.836229	-2.613337	0.778877
H	7.689792	-1.481261	-2.511232
H	7.808246	0.217399	-2.082434
H	9.495545	-4.693290	-1.752815
H	8.022482	-6.487377	-2.622166
H	5.563518	-6.363067	-2.261777
H	4.597077	-4.433691	-1.023056
H	6.089286	-2.635693	-0.159807
H	9.489428	-2.596673	-3.626000
H	11.721027	-2.512289	-4.699727
H	13.195036	-0.537192	-4.355695
H	12.411784	1.350841	-2.937158

H 10.184790 1.261229 -1.868197

Q--QPdPh$_2$, less stable conformer of dissociated form of **bis(N,N-dibenzyl-2-aminoNPhos)PdPh$_2$**: B3LYP/6-31G(d)/SDD for pre-reductive elimination step. P-Pd distance=6.009 Å, C-Pd-C angle=83.95°. ($E_{e,rel}$=3.81 kcal/mol, $G_{298,rel}$=3.96 kcal/mol)

Processing: pddiphdi2namphos-a6dsdbe.log
PG=C01

Method BasisSet Imaginary Freqs
RB3LYP GenECP 0

HF Energy
-5097.8045579

ZPE	E298	S298	Squasihar	Equasihar	Strans	Srot
1173.74174	1239.129	568.602	463.680	1239.534	47.927	43.784

Processing: pddiphdi2namphos-a6dsdbe.log
219
C -2.988695 1.852320 1.874381
C -2.281982 0.525571 1.541025
C -2.366382 -0.483309 2.704686
C -1.865116 0.128571 4.024824
C -2.580272 1.447909 4.351768
C -2.458337 2.447081 3.192198
P -2.505842 -0.302378 -0.145519
Pd -1.137244 -2.321897 0.335750
C -0.771050 -3.411967 -1.301530
C -1.541047 -4.543817 -1.609862
C -1.337765 -5.238498 -2.808478
C -0.352373 -4.826014 -3.708437
C 0.431927 -3.712269 -3.397955
C 0.230026 -3.014404 -2.200519
C -4.257158 -0.720228 -0.648977
C -5.444126 -0.653875 0.117857
C -6.669727 -1.173947 -0.369478
C -6.655120 -1.816307 -1.613476
C -5.512109 -1.905970 -2.396612
C -4.327951 -1.342266 -1.920643
C -7.992387 -1.092271 0.334784
C -8.727578 0.098841 0.382699
C -9.987859 0.111290 1.050644
C -10.502819 -1.014426 1.642903
C -9.801381 -2.244034 1.596523
C -8.533159 -2.285836 0.927332
C -10.320932 -3.420529 2.200057
C -9.620311 -4.604037 2.165178
C -8.360020 -4.650892 1.523032
C -7.832314 -3.528164 0.923037

N	-8.222279	1.300016	-0.198666
C	-8.638453	2.552122	0.444324
C	-7.673095	3.699749	0.198102
C	-8.156783	4.967796	-0.144130
C	-7.283808	6.046077	-0.312644
C	-5.911106	5.864339	-0.144334
C	-5.419049	4.600382	0.194824
C	-6.292348	3.525979	0.366007
O	-5.330965	-0.032901	1.333007
C	-6.055205	-0.501596	2.474267
O	-3.169142	-1.353396	-2.630845
C	-3.094274	-2.113944	-3.833745
C	-8.210315	1.368540	-1.679942
C	-9.570059	1.568222	-2.333710
C	-10.459690	0.494381	-2.492750
C	-11.710093	0.681958	-3.082687
C	-12.092920	1.949463	-3.528978
C	-11.215999	3.025278	-3.383183
C	-9.965746	2.833178	-2.790292
C	-1.932438	1.136382	-1.254547
C	-3.041950	1.856870	-2.044265
C	-2.479672	3.075951	-2.797725
C	-1.310969	2.686315	-3.714401
C	-0.208566	1.961684	-2.928344
C	-0.756464	0.740290	-2.170052
C	-0.065506	-3.751262	1.267704
C	-0.729087	-4.694655	2.074091
C	-0.017596	-5.542872	2.930153
C	1.376431	-5.480117	2.982373
C	2.052851	-4.567115	2.169693
C	1.339634	-3.719798	1.314930
P	3.431080	1.329377	1.717054
C	2.517981	2.994706	1.569248
C	2.229977	3.901796	2.781390
C	1.280716	5.052420	2.394857
C	1.829145	5.869665	1.215895
C	2.125776	4.970642	0.006560
C	3.064835	3.810396	0.379098
C	3.083944	0.755432	3.494096
C	3.161342	-0.786468	3.559249
C	2.717032	-1.326963	4.928118
C	3.524669	-0.700894	6.073566
C	3.462255	0.832117	6.016460
C	3.902575	1.374515	4.643681
C	5.287548	1.600717	1.663811
C	6.020466	0.575357	1.010659
C	7.415327	0.657962	0.793243
C	8.083555	1.756388	1.345619
C	7.414240	2.765041	2.029218
C	6.022875	2.696770	2.168025
O	5.289321	-0.493355	0.571187

C	5.719271	-1.822529	0.880570
C	8.196392	-0.334958	-0.012790
C	9.124379	-1.197068	0.667978
C	9.889293	-2.159197	-0.071009
C	9.708352	-2.230213	-1.473435
C	8.835931	-1.389414	-2.115595
C	8.068550	-0.415576	-1.409823
C	10.792626	-3.019288	0.608200
C	10.941233	-2.956611	1.974625
C	10.176127	-2.025209	2.715855
C	9.295980	-1.173576	2.084179
O	5.316276	3.674490	2.810435
C	6.019442	4.765031	3.383925
N	7.183492	0.436321	-2.129953
C	7.461944	1.889374	-2.106192
C	8.554868	2.357457	-3.058360
C	8.234554	3.052968	-4.231388
C	9.231661	3.481090	-5.111070
C	10.571996	3.214848	-4.828933
C	10.906837	2.523060	-3.661746
C	9.907670	2.101267	-2.784363
C	6.714061	-0.001707	-3.450689
C	5.948719	-1.312352	-3.471643
C	6.192052	-2.246920	-4.485457
C	5.441873	-3.421735	-4.573267
C	4.436890	-3.677779	-3.639553
C	4.188558	-2.751412	-2.622373
C	4.935065	-1.574635	-2.539994
H	-7.801758	-5.583106	1.501807
H	10.282538	-1.981438	3.796780
H	7.978255	3.597481	2.431353
H	9.159986	1.830978	1.213882
H	1.536113	2.606237	1.246468
H	4.050093	4.213310	0.643672
H	3.211863	3.156046	-0.490215
H	2.564361	5.560979	-0.808984
H	1.179783	4.560935	-0.379475
H	2.757101	6.372917	1.526970
H	1.122387	6.662851	0.938449
H	1.109917	5.703022	3.263135
H	0.298326	4.638316	2.120011
H	3.165614	4.315733	3.164968
H	1.780549	3.320480	3.595922
H	2.025704	1.032869	3.634690
H	4.963911	1.135780	4.487040
H	3.826307	2.464819	4.629610
H	4.087494	1.269822	6.806609
H	2.431060	1.158955	6.220184
H	4.574211	-1.022338	5.994200
H	3.159820	-1.062700	7.043891
H	2.805940	-2.420661	4.941734

H	1.649249	-1.102379	5.072776
H	4.198786	-1.100069	3.370909
H	2.551895	-1.232566	2.765645
H	0.857300	-2.155804	-1.978169
H	1.215842	-3.386363	-4.078320
H	-0.189121	-5.372767	-4.633902
H	-1.944506	-6.114991	-3.027945
H	-2.295720	-4.897093	-0.912871
H	-1.814506	-4.772524	2.040241
H	-0.554339	-6.258578	3.549917
H	1.930960	-6.143815	3.641380
H	3.140013	-4.520568	2.192644
H	1.886632	-3.026442	0.679953
H	4.805935	-2.408234	1.017820
H	6.305968	-2.249936	0.064294
H	6.306609	-1.843224	1.804212
H	5.260036	5.397558	3.847810
H	6.728385	4.429100	4.151380
H	6.557119	5.345768	2.623468
H	10.282594	-2.956542	-2.044052
H	8.740380	-1.457189	-3.191617
H	8.717688	-0.470822	2.673274
H	11.364049	-3.735523	0.021865
H	11.634559	-3.619758	2.484640
H	6.039932	0.794061	-3.794548
H	7.523176	-0.037637	-4.198937
H	6.524425	2.402715	-2.351961
H	7.716152	2.170205	-1.085150
H	7.191463	3.268115	-4.455017
H	8.960641	4.022363	-6.014022
H	11.351094	3.546844	-5.510141
H	11.949040	2.317047	-3.431321
H	10.178708	1.568327	-1.876971
H	6.975306	-2.052332	-5.216009
H	5.647479	-4.136366	-5.366313
H	3.851261	-4.591401	-3.697802
H	3.406124	-2.947840	-1.894037
H	4.747756	-0.863707	-1.741095
H	-7.584662	-2.240533	-1.983713
H	-5.551819	-2.394335	-3.362101
H	-5.492886	-0.149711	3.342726
H	-7.065817	-0.091366	2.509530
H	-6.103776	-1.594823	2.490553
H	-2.051321	-2.068805	-4.145544
H	-3.363025	-3.159022	-3.653605
H	-3.739246	-1.683609	-4.610452
H	-1.533781	1.858821	-0.527828
H	-3.483161	1.161673	-2.767462
H	-3.851766	2.172874	-1.376000
H	-3.280973	3.552034	-3.377507
H	-2.134045	3.826399	-2.070535

H	-1.682282	2.024643	-4.511160
H	-0.903790	3.576362	-4.211362
H	0.598423	1.646179	-3.602067
H	0.243151	2.663274	-2.210322
H	-1.091184	-0.019636	-2.883552
H	0.041836	0.281330	-1.574858
H	-1.208848	0.762395	1.429007
H	-3.396924	-0.832650	2.821064
H	-1.763688	-1.381386	2.480438
H	-1.996092	-0.593712	4.840436
H	-0.784063	0.315295	3.944472
H	-3.644833	1.248599	4.547338
H	-2.171247	1.880560	5.273682
H	-3.003762	3.370727	3.425006
H	-1.401930	2.730427	3.069936
H	-4.064194	1.681455	1.954720
H	-2.840779	2.575209	1.062445
H	-10.564801	1.028956	1.067082
H	-11.469799	-0.976276	2.139286
H	-6.865588	-3.586517	0.435433
H	-11.287753	-3.364410	2.695236
H	-10.026503	-5.497953	2.630293
H	-9.648270	2.871508	0.137070
H	-8.684899	2.357898	1.522342
H	-7.547394	2.194573	-1.955380
H	-7.747643	0.452091	-2.048696
H	-9.226708	5.112991	-0.277752
H	-7.677294	7.023697	-0.578987
H	-5.228226	6.699335	-0.276984
H	-4.350868	4.453057	0.331183
H	-5.913902	2.541487	0.625624
H	-9.282588	3.673175	-2.686281
H	-11.501738	4.013543	-3.734616
H	-13.065158	2.094897	-3.992509
H	-12.383414	-0.163208	-3.200913
H	-10.168363	-0.497004	-2.155761

Q_2PdPh_2, conformer of associated form of **bis(N,N-dibenzyl-2-aminoNPhos)$PdPh_2$**: B3LYP/6-31G(d)/SDD for pre-reductive elimination step. P-Pd distances=2.547, 2.625 Å, C-Pd-C angle=79.01°. ($E_{e,rel}$=5.98 kcal/mol, $G_{298,rel}$=8.99 kcal/mol; remarkably $G_{298,rel}$=17.45 kcal/mol using distorted non-quasiharmonic frequencies!)

Processing: pddiphdi2namphos-b6dsdbe.log
PG=C01

Method	BasisSet	Imaginary Freqs
RB3LYP	GenECP	0

HF Energy
-5097.8010984

ZPE	E298	S298	Squasihar	Equasihar	Strans	Srot

1176.24745 1240.480 536.098 458.547 1240.820 47.927 43.332

Processing: pddiphdi2namphos-b6dsdbe.log
219
C -4.967490 -4.033560 -1.954192
C -5.428230 -2.908683 -2.701598
C -5.766362 -3.129855 -4.077802
C -5.653671 -4.429709 -4.639207
C -5.220809 -5.492561 -3.881528
C -4.872665 -5.284052 -2.526158
C -6.222408 -2.031110 -4.842216
C -6.345101 -0.787082 -4.279126
C -6.007346 -0.531064 -2.915207
C -5.535267 -1.591884 -2.126325
C -5.261307 -1.474024 -0.647887
C -4.080040 -1.027378 0.001009
C -3.945496 -1.072435 1.421335
C -5.074258 -1.455969 2.177028
C -6.239374 -1.918706 1.557330
C -6.295872 -1.943646 0.173778
P -2.306854 -0.585789 2.206198
C -2.702783 1.201573 2.743098
C -2.889946 2.074387 1.480361
C -2.947950 3.570065 1.835461
C -4.066555 3.871324 2.841575
C -3.947106 2.973315 4.078713
C -3.882293 1.481203 3.700902
O -2.997066 -0.492901 -0.620349
C -2.801698 -0.516011 -2.032048
O -4.986470 -1.344864 3.534844
C -6.104562 -1.707941 4.330092
Pd -0.045258 -1.003574 0.939910
C -0.544966 -2.995534 0.746397
C -1.428530 -3.546133 -0.194693
C -1.681275 -4.922497 -0.250998
C -1.055907 -5.795427 0.640455
C -0.165006 -5.274164 1.581100
C 0.089067 -3.899823 1.621198
P 1.349934 1.080956 1.381384
C 1.286608 1.968180 3.086834
C 2.565606 2.722713 3.512524
C 2.293565 3.618555 4.735648
C 1.724552 2.816529 5.915314
C 0.491760 2.002032 5.494736
C 0.796465 1.115905 4.273999
C 0.741517 2.540267 0.322430
C 1.440950 3.914825 0.391081
C 0.652981 4.985346 -0.388873
C 0.396884 4.562885 -1.840849
C -0.342019 3.219228 -1.888543
C 0.411862 2.119054 -1.122574

C	3.195577	0.750471	1.209940
C	4.100372	1.293941	0.272089
C	5.506216	1.116751	0.385161
C	5.971929	0.277383	1.399643
C	5.106712	-0.379693	2.264547
C	3.734392	-0.145541	2.165220
O	3.580103	2.088522	-0.720625
C	3.865532	1.736396	-2.078404
C	6.522014	1.861393	-0.428344
C	7.519887	1.184804	-1.151632
C	8.507582	1.938241	-1.851450
C	8.522108	3.309333	-1.832492
C	7.556589	4.031303	-1.090865
C	6.554531	3.301174	-0.368494
C	5.653905	4.058263	0.436375
C	5.713466	5.433339	0.486660
C	6.677016	6.145691	-0.265972
C	7.583033	5.450888	-1.032080
O	2.830915	-0.749011	2.991774
C	3.278254	-1.751908	3.891349
C	1.418148	-1.651418	-0.358094
C	1.262346	-1.376818	-1.729187
C	2.186464	-1.820227	-2.683274
C	3.298836	-2.568001	-2.291693
C	3.470740	-2.857687	-0.936020
C	2.544026	-2.406240	0.008392
C	-2.000820	-1.674951	3.760867
C	-2.290186	-1.116593	5.168770
C	-1.654758	-2.013693	6.248287
C	-2.132376	-3.468278	6.134316
C	-1.893119	-4.020249	4.722023
C	-2.525971	-3.125105	3.642310
N	7.586745	-0.240761	-1.184739
N	-6.113171	0.806661	-2.416200
H	5.013547	5.976930	1.115969
H	-4.524974	-6.120446	-1.925844
H	-7.092471	-2.251773	2.134158
H	-7.195219	-2.319973	-0.306275
H	-0.906720	-1.749716	3.680465
H	-3.611233	-3.129838	3.770888
H	-2.309461	-3.535977	2.654947
H	-2.296315	-5.037556	4.635699
H	-0.812875	-4.100409	4.536201
H	-3.208147	-3.511529	6.363576
H	-1.629719	-4.093997	6.883722
H	-1.883550	-1.611240	7.244350
H	-0.559420	-1.982788	6.144295
H	-3.370934	-1.068167	5.334094
H	-1.902601	-0.097460	5.278397
H	-1.779730	1.529746	3.239708
H	-4.818676	1.196319	3.206502

H	-3.812924	0.881151	4.607647
H	-4.793727	3.144233	4.757044
H	-3.040409	3.244860	4.640791
H	-5.038943	3.694230	2.358975
H	-4.048403	4.930397	3.130041
H	-3.093890	4.162444	0.924368
H	-1.981971	3.881790	2.261413
H	-3.828011	1.786962	0.987039
H	-2.095292	1.881968	0.754352
H	2.694567	-2.674222	1.050160
H	4.325050	-3.448574	-0.609842
H	4.012847	-2.923176	-3.030376
H	2.024530	-1.594440	-3.736187
H	0.395841	-0.819759	-2.075958
H	-1.926423	-2.899494	-0.909814
H	-2.359688	-5.308822	-1.008190
H	-1.248812	-6.864739	0.594756
H	0.341637	-5.938264	2.279581
H	0.804638	-3.528521	2.352854
H	-1.781222	-0.158042	-2.175463
H	-2.888804	-1.526704	-2.438681
H	-3.503925	0.152073	-2.534772
H	-5.807978	-1.516982	5.363018
H	-6.984825	-1.099757	4.087238
H	-6.353707	-2.770623	4.217156
H	-6.499566	-2.187284	-5.882257
H	-6.740999	0.017688	-4.885668
H	-4.680887	-3.901799	-0.917752
H	-5.921042	-4.566205	-5.684666
H	-5.140820	-6.484518	-4.317417
H	7.042287	0.152434	1.517858
H	5.505683	-1.039103	3.025485
H	3.299859	2.442229	-2.690677
H	4.928032	1.845601	-2.307392
H	3.531357	0.715009	-2.291461
H	2.379288	-2.135589	4.376535
H	3.780668	-2.567715	3.357745
H	3.955460	-1.341014	4.651594
H	0.507627	2.724259	2.904512
H	3.345983	1.995148	3.767119
H	2.965806	3.326725	2.692674
H	3.218845	4.128414	5.034409
H	1.580095	4.407754	4.454264
H	2.497937	2.128185	6.287426
H	1.476341	3.486833	6.748358
H	0.145403	1.379892	6.330684
H	-0.333940	2.689471	5.254619
H	1.568713	0.390522	4.542022
H	-0.084531	0.534574	3.992425
H	-0.230443	2.669799	0.822508
H	1.330662	1.868346	-1.655816

H	-0.191503	1.205102	-1.095164
H	-0.499132	2.903189	-2.928415
H	-1.342799	3.347394	-1.449919
H	1.358320	4.471919	-2.367749
H	-0.178330	5.334285	-2.369497
H	1.201842	5.935683	-0.356501
H	-0.312620	5.166105	0.108154
H	2.450685	3.839106	-0.018813
H	1.533236	4.242619	1.432063
H	9.248251	1.408824	-2.440069
H	9.278627	3.856466	-2.390464
H	4.919541	3.538118	1.037818
H	8.346927	5.976854	-1.600471
H	6.706962	7.231105	-0.224725
C	6.538459	-0.960524	-1.937303
C	8.923519	-0.826743	-1.312747
H	6.462160	-1.968016	-1.519359
H	5.585210	-0.474492	-1.729142
C	6.771250	-1.067479	-3.440071
C	7.067164	-2.309834	-4.018363
C	7.277543	-2.434234	-5.393916
C	7.201479	-1.310112	-6.216834
C	6.912476	-0.064091	-5.654532
C	6.698091	0.054448	-4.280857
H	7.133139	-3.188979	-3.381736
H	7.500418	-3.409119	-5.820128
H	7.363335	-1.402688	-7.287602
H	6.847144	0.816922	-6.288165
H	6.474624	1.030024	-3.859077
C	9.020925	-2.252604	-0.789316
H	9.291424	-0.819405	-2.352535
H	9.607301	-0.193725	-0.734141
C	9.814153	-3.186359	-1.467460
C	9.979400	-4.482113	-0.972973
C	9.341031	-4.866555	0.206861
C	8.538560	-3.946079	0.885657
C	8.382452	-2.650000	0.393165
H	10.305533	-2.895812	-2.393473
H	10.599763	-5.191618	-1.514547
H	9.461794	-5.875859	0.591301
H	8.031613	-4.239120	1.801651
H	7.746004	-1.941274	0.913134
C	-6.081097	1.865244	-3.437527
C	-7.117458	1.063585	-1.355718
H	-6.858887	2.023304	-0.898073
C	-8.569103	1.104845	-1.816860
H	-6.991392	0.301837	-0.587153
C	-9.240177	-0.061891	-2.216619
C	-10.571769	-0.015239	-2.630516
C	-11.261397	1.199912	-2.646817
C	-10.609027	2.366430	-2.246186

C -9.274118 2.315980 -1.837068
H -8.717075 -1.013810 -2.203927
H -11.073959 -0.930198 -2.934390
H -12.299957 1.234876 -2.965312
H -11.137610 3.316405 -2.250120
H -8.771257 3.228358 -1.525175
C -5.722850 3.242401 -2.906189
H -5.324506 1.575093 -4.175242
H -7.037529 1.947996 -3.979040
C -4.558437 3.458873 -2.156785
C -4.195629 4.749429 -1.769828
C -4.994093 5.843267 -2.115484
C -6.159774 5.637321 -2.852771
C -6.517410 4.344948 -3.244612
H -3.938091 2.610939 -1.879335
H -3.279709 4.905517 -1.207118
H -4.706707 6.846623 -1.812746
H -6.790582 6.479206 -3.126131
H -7.423692 4.188536 -3.825074

trans-$(AcO)_2Pd(N_2Phos)$: B3LYP/6-31G(d)/SDD

Processing: pdac2evanphos-b6dsdbe.log
PG=C01

Method	BasisSet	Imaginary Freqs
RB3LYP	GenECP	0

HF Energy
-2356.7361552

ZPE	E298	S298	Squasihar	Equasihar	Strans	Srot
468.85698	497.734	270.552	246.300	497.869	45.578	38.413

Processing: pdac2evanphos-b6dsdbe.log
89
C 4.209969 -0.254047 -0.822295
C 2.888744 -0.987338 -1.152157
C 2.328522 -0.549713 -2.525682
C 3.377051 -0.730371 -3.636217
C 4.674515 0.021035 -3.310484
C 5.237347 -0.427583 -1.955229
P 1.608939 -0.706315 0.179079
C 0.094718 -1.656802 -0.307325
C -1.121419 -0.981826 -0.134847
C -2.334527 -1.399068 -0.702734
C -2.311936 -2.627827 -1.371606
C -1.150485 -3.391032 -1.482017
C 0.057100 -2.904660 -0.969146
O -1.107165 0.200617 0.617795

C	-1.705294	0.069746	1.946267
C	-3.534582	-0.517113	-0.708968
C	-4.780926	-0.916828	-0.133234
C	-5.908681	-0.030463	-0.208498
C	-5.747643	1.226701	-0.842478
C	-4.543520	1.606575	-1.386503
C	-3.427973	0.731155	-1.333059
C	-7.148816	-0.423842	0.361760
C	-7.286168	-1.635674	0.998755
C	-6.171891	-2.503538	1.097191
C	-4.956311	-2.155772	0.550371
O	1.240053	-3.575157	-1.077740
C	1.271646	-4.801125	-1.799657
O	-2.234569	1.030343	-1.901722
C	-1.940758	2.380845	-2.278519
C	2.192869	-1.422058	1.815334
C	2.965843	-2.754185	1.721328
C	3.290177	-3.298012	3.124640
C	4.049793	-2.268440	3.973733
C	3.283178	-0.940195	4.055317
C	2.959204	-0.377999	2.660842
Pd	0.670769	1.378947	0.409147
O	-0.338245	3.181856	0.617489
O	2.348017	2.463610	0.164661
H	-1.188257	-4.338801	-2.005127
H	-3.225143	-2.984243	-1.838813
H	3.080316	-2.065784	-1.177437
H	4.008231	0.810047	-0.660947
H	4.636153	-0.637965	0.111889
H	6.145605	0.136955	-1.708911
H	5.534235	-1.485625	-2.018403
H	4.466214	1.099659	-3.280154
H	5.418604	-0.137570	-4.101526
H	2.959379	-0.377992	-4.587460
H	3.599204	-1.800913	-3.766391
H	2.025569	0.503597	-2.478259
H	1.430992	-1.130617	-2.768177
H	1.241419	-1.621023	2.331976
H	3.893913	-0.102058	2.155625
H	2.369699	0.541172	2.748311
H	3.859072	-0.199207	4.623067
H	2.344631	-1.094723	4.608067
H	5.038720	-2.089701	3.525915
H	4.230331	-2.666984	4.980018
H	3.871459	-4.224689	3.033724
H	2.351837	-3.566841	3.632240
H	3.905489	-2.593509	1.175217
H	2.391992	-3.491288	1.150757
H	-1.515642	1.020981	2.443008
H	-2.771327	-0.132378	1.828700
H	-1.214164	-0.747971	2.483800

H	2.311165	-5.131244	-1.775002
H	0.637629	-5.560327	-1.325542
H	0.957253	-4.659250	-2.840641
H	-6.599720	1.900242	-0.894836
H	-4.447581	2.571169	-1.870787
H	-4.111730	-2.830595	0.644892
H	-6.275983	-3.454124	1.613978
H	-7.991619	0.259826	0.288649
H	-8.239421	-1.924882	1.432029
H	-0.879062	2.391736	-2.526708
H	-2.536838	2.682249	-3.149473
H	-2.105940	3.063746	-1.439863
C	2.419404	3.074393	-0.995061
C	-0.582753	3.516162	1.850608
O	1.725694	2.830029	-1.982042
C	3.482555	4.167425	-1.010509
O	-0.406331	2.787921	2.837689
C	-1.114407	4.937345	2.007857
H	-1.516504	5.082583	3.012849
H	-0.295365	5.647581	1.844743
H	-1.883349	5.149318	1.258329
H	3.750594	4.412465	-2.040608
H	3.071589	5.062923	-0.530006
H	4.369978	3.868205	-0.445347

trans-$(AcO)_2Pd(EvanPhos)_2$: B3LYP/6-31G(d)/SDD

Processing: pdac2evanphos-x6dsdbe.log
PG=CI

Method	BasisSet	Imaginary Freqs
RB3LYP	GenECP	0

HF Energy
-4128.5306274

ZPE	E298	S298	Squasihar	Equasihar	Strans	Srot
872.86223	923.904	432.267	381.824	924.157	47.136	41.254

Processing: pdac2evanphos-x6dsdbe.log
163

C	-2.211409	4.063073	2.945551
C	-2.203097	4.395574	1.446452
C	-1.361229	3.390802	0.639419
C	0.077126	3.306884	1.200162
C	0.034676	2.904116	2.684933
C	-0.783394	3.926584	3.494020
P	1.101933	2.179242	0.092333
Pd	0.000000	0.000000	0.000000
O	1.027783	-0.505121	1.719171

C	1.234151	3.190952	-1.517179
C	2.405808	2.725034	-2.404803
C	2.424021	3.462331	-3.753739
C	2.476402	4.983980	-3.573994
C	1.327558	5.459529	-2.676838
C	1.301460	4.723787	-1.323645
C	2.850867	2.095621	0.727794
C	3.643370	0.988470	0.321887
C	4.963055	0.801601	0.789884
C	5.489302	1.779819	1.638828
C	4.768783	2.905244	2.013604
C	3.460834	3.073396	1.545035
C	5.831588	-0.368531	0.449351
C	6.982890	-0.160047	-0.311527
C	7.882346	-1.219856	-0.589057
C	7.627406	-2.483340	-0.108868
C	6.469609	-2.756021	0.658222
C	5.555392	-1.684227	0.940269
C	4.388535	-1.984951	1.700907
C	4.154576	-3.262861	2.158827
C	5.064644	-4.314734	1.889124
C	6.196929	-4.061204	1.151223
O	7.177020	1.109470	-0.788189
C	8.331628	1.386693	-1.562006
O	3.040332	0.113793	-0.528500
C	3.733966	-0.390012	-1.677978
O	2.737655	4.200200	1.819542
C	3.291838	5.187792	2.674347
P	-1.101933	-2.179242	-0.092333
C	-1.234151	-3.190952	1.517179
C	-1.301460	-4.723787	1.323645
C	-1.327558	-5.459529	2.676838
C	-2.476402	-4.983980	3.573994
C	-2.424021	-3.462331	3.753739
C	-2.405808	-2.725034	2.404803
C	-0.077126	-3.306884	-1.200162
C	-0.034676	-2.904116	-2.684933
C	0.783394	-3.926584	-3.494020
C	2.211409	-4.063073	-2.945551
C	2.203097	-4.395574	-1.446452
C	1.361229	-3.390802	-0.639419
C	-2.850867	-2.095621	-0.727794
C	-3.460834	-3.073396	-1.545035
C	-4.768783	-2.905244	-2.013604
C	-5.489302	-1.779819	-1.638828
C	-4.963055	-0.801601	-0.789884
C	-3.643370	-0.988470	-0.321887
O	-2.737655	-4.200200	-1.819542
C	-3.291838	-5.187792	-2.674347
O	-3.040332	-0.113793	0.528500
C	-3.733966	0.390012	1.677978

C	-5.831588	0.368531	-0.449351
C	-5.555392	1.684227	-0.940269
C	-6.469609	2.756021	-0.658222
C	-7.627406	2.483340	0.108868
C	-7.882346	1.219856	0.589057
C	-6.982890	0.160047	0.311527
C	-6.196929	4.061204	-1.151223
C	-5.064644	4.314734	-1.889124
C	-4.154576	3.262861	-2.158827
C	-4.388535	1.984951	-1.700907
O	-7.177020	-1.109470	0.788189
C	-8.331628	-1.386693	1.562006
O	-1.027783	0.505121	-1.719171
H	8.321308	-3.293040	-0.322050
H	2.765783	-4.830353	-3.502404
H	6.904724	-4.857252	0.930547
H	8.772010	-1.042043	-1.181727
H	8.355926	0.795250	-2.486992
H	0.810053	-3.623780	-4.548907
H	0.285504	-4.908569	-3.460883
H	2.744267	-3.114504	-3.101711
H	9.255731	1.207505	-0.996167
H	1.791752	-5.406957	-1.303747
H	3.226039	-4.416417	-1.049862
H	4.863685	-5.315390	2.262038
H	8.268301	2.446254	-1.817613
H	-2.528952	-5.963483	-2.762037
H	4.217406	-1.344182	-1.454082
H	-3.512301	-4.782182	-3.669755
H	2.960565	-0.525058	-2.435895
H	4.483355	0.327221	-2.027489
H	3.281199	3.115873	-4.345954
H	-4.205168	-5.623179	-2.249154
H	-1.048051	-2.836073	-3.093932
H	0.425229	-1.918449	-2.790623
H	-0.533455	-4.297080	-1.141044
H	1.824270	-2.397868	-0.674972
H	1.361320	-3.686387	0.416159
H	3.254689	-3.468978	2.732375
H	1.523998	3.187585	-4.322293
H	2.319842	1.655197	-2.583926
H	2.436530	5.492137	-4.546563
H	-0.445786	-5.089821	0.749079
H	3.435645	5.263106	-3.112361
H	3.351927	2.914923	-1.879202
H	3.665345	-1.202878	1.901343
H	-5.231118	-3.645846	-2.654023
H	6.502549	1.653093	2.008326
H	-1.396617	-6.541462	2.501139
H	-2.201205	-4.979848	0.748490
H	-0.371441	-5.289149	3.194366

H	-0.300176	-2.952297	2.042554
H	0.300176	2.952297	-2.042554
H	0.371441	5.289149	-3.194366
H	1.396617	6.541462	-2.501139
H	2.201205	4.979848	-0.748490
H	-6.502549	-1.653093	-2.008326
H	5.231118	3.645846	2.654023
H	-3.435645	-5.263106	3.112361
H	-2.436530	-5.492137	4.546563
H	-3.665345	1.202878	-1.901343
H	-3.351927	-2.914923	1.879202
H	0.445786	5.089821	-0.749079
H	-1.523998	-3.187585	4.322293
H	-2.319842	-1.655197	2.583926
H	-3.254689	3.468978	-2.732375
H	-1.361320	3.686387	-0.416159
H	4.205168	5.623179	2.249154
H	-1.824270	2.397868	0.674972
H	0.533455	4.297080	1.141044
H	-3.281199	-3.115873	4.345954
H	1.048051	2.836073	3.093932
H	-0.425229	1.918449	2.790623
H	3.512301	4.782182	3.669755
H	-8.268301	-2.446254	1.817613
H	-4.483355	-0.327221	2.027489
H	-2.960565	0.525058	2.435895
H	2.528952	5.963483	2.762037
H	-4.217406	1.344182	1.454082
H	-9.255731	-1.207505	0.996167
H	-4.863685	5.315390	-2.262038
H	-1.791752	5.406957	1.303747
H	-3.226039	4.416417	1.049862
H	-8.355926	-0.795250	2.486992
H	-0.285504	4.908569	3.460883
H	-2.744267	3.114504	3.101711
H	-8.772010	1.042043	1.181727
H	-0.810053	3.623780	4.548907
H	-6.904724	4.857252	-0.930547
H	-8.321308	3.293040	0.322050
H	-2.765783	4.830353	3.502404
C	-0.478566	0.484300	-2.897733
C	0.478566	-0.484300	2.897733
O	-0.701599	-0.222600	3.156148
C	1.463845	-0.782739	4.028691
O	0.701599	0.222600	-3.156148
C	-1.463845	0.782739	-4.028691
H	-0.921599	0.967003	-4.958561
H	-2.122377	-0.083078	-4.167570
H	-2.101219	1.638986	-3.790583
H	0.921599	-0.967003	4.958561
H	2.101219	-1.638986	3.790583

H 2.122377 0.083078 4.167570

(AcO) ${}_2$Pd(N${}_2$Phos): B3LYP/6-31G(d)/SDD

Processing: pdac2namphos6dsdbe.log
PG=C01

Method	BasisSet	Imaginary Freqs
RB3LYP	GenECP	0

HF Energy
-3434.3157661

ZPE	E298	S298	Squasihar	Equasihar	Strans	Srot
745.04962	788.806	393.764	335.419	789.064	46.795	41.216

Processing: pdac2namphos6dsdbe.log
141

C	-1.948357	3.272092	-2.363686
C	-1.369364	4.446163	-1.865632
C	-1.088115	5.489063	-2.757008
C	-1.374983	5.365415	-4.117960
C	-1.950873	4.190563	-4.603480
C	-2.237734	3.143783	-3.723051
C	-1.144827	4.639493	-0.379111
N	-0.424345	3.554540	0.311254
C	-0.669733	3.610527	1.770712
C	-0.209011	4.886016	2.465282
C	-1.140835	5.823355	2.929099
C	-0.727753	6.992893	3.571611
C	0.632206	7.244132	3.756741
C	1.573301	6.318039	3.298882
C	1.155809	5.149242	2.661952
C	0.888827	3.229627	-0.132543
C	1.627116	4.186983	-0.890243
C	2.896561	3.926689	-1.340811
C	3.528688	2.693605	-1.066156
C	2.821549	1.706816	-0.308264
C	1.481317	1.982426	0.138893
C	3.454560	0.453441	-0.081604
C	4.738330	0.169414	-0.548445
C	5.438102	1.187801	-1.269649
C	4.839167	2.396529	-1.519876
N	5.348859	-1.062389	-0.315269
C	6.460892	-1.543974	-1.122665
C	7.843267	-1.407806	-0.491073
C	8.896866	-2.197603	-0.971985
C	10.181183	-2.073710	-0.443210
C	10.430143	-1.159956	0.584477
C	9.386039	-0.375256	1.074879

C	8.100811	-0.498187	0.540016
C	0.792523	0.943812	0.973989
C	-0.289154	0.128388	0.576934
C	-0.977605	-0.703700	1.487682
C	-0.509914	-0.772422	2.818877
C	0.619731	-0.051937	3.213034
C	1.239253	0.785110	2.293729
O	-0.746073	0.120322	-0.737421
Pd	-2.683733	-0.729674	-1.212153
O	-4.496723	-1.520777	-1.596433
P	-2.457743	-1.624112	0.863812
C	-1.948371	-3.433412	0.720978
C	-1.958779	-4.253343	2.029262
C	-1.431018	-5.679475	1.788023
C	-2.213815	-6.399240	0.681456
C	-2.209229	-5.578687	-0.615654
C	-2.739255	-4.151474	-0.399840
O	-1.204891	-1.576527	3.673056
C	-0.793915	-1.658503	5.032694
C	4.837801	-2.002903	0.668677
C	3.896604	-3.083502	0.140029
C	3.202570	-2.940257	-1.067182
C	2.328649	-3.937123	-1.510811
C	2.142896	-5.094557	-0.752429
C	2.835744	-5.249491	0.451884
C	3.706209	-4.251727	0.890923
C	-3.828096	-1.385965	2.112534
C	-4.157723	0.120547	2.231704
C	-5.298580	0.363063	3.234175
C	-6.549657	-0.443436	2.860769
C	-6.222225	-1.939374	2.759872
C	-5.087697	-2.206973	1.754848
C	0.233007	0.113121	-1.833102
O	-2.731312	-0.040947	-3.184547
H	1.000296	-0.111899	4.225004
H	2.090991	1.378653	2.613784
H	-3.438264	-1.742167	3.071857
H	-5.421696	-1.945983	0.746898
H	-4.856652	-3.278210	1.742918
H	-7.111599	-2.508768	2.461211
H	-5.927816	-2.315777	3.751543
H	-6.927584	-0.088598	1.892043
H	-7.346551	-0.277650	3.597070
H	-5.530573	1.434980	3.265818
H	-4.970200	0.081137	4.246586
H	-4.453310	0.500393	1.245802
H	-3.264898	0.676936	2.543773
H	-0.905319	-3.348952	0.380669
H	-3.804223	-4.194022	-0.137319
H	-2.671000	-3.582818	-1.331924
H	-2.809964	-6.075264	-1.387666

H	-1.182848	-5.522908	-1.006979
H	-3.252075	-6.552068	1.012167
H	-1.792395	-7.397392	0.505396
H	-1.477834	-6.248964	2.725679
H	-0.369223	-5.624336	1.506403
H	-2.984756	-4.313486	2.417605
H	-1.359606	-3.757386	2.796530
H	0.286652	1.117124	-2.251504
H	1.199716	-0.201273	-1.443196
H	-0.143766	-0.608206	-2.556594
H	-1.503505	-2.332922	5.514217
H	0.218147	-2.071952	5.120907
H	-0.833212	-0.677156	5.520512
H	3.435392	4.681195	-1.909625
H	1.183924	5.150493	-1.099738
H	2.897626	-0.314853	0.438017
H	5.382365	3.159987	-2.072289
H	6.458353	1.020802	-1.596038
H	-2.125369	4.685776	0.111453
H	-0.681840	5.623424	-0.201640
H	-1.749467	3.490299	1.919370
H	-0.190521	2.750679	2.234602
H	-2.203029	5.632661	2.789723
H	-1.468130	7.705596	3.925737
H	0.957845	8.152927	4.255937
H	2.634585	6.504364	3.442704
H	1.896062	4.434961	2.311595
H	-0.647306	6.410935	-2.381295
H	-1.149157	6.185963	-4.794574
H	-2.179783	4.092448	-5.661778
H	-2.692507	2.221536	-4.069071
H	-2.186676	2.454956	-1.689272
H	6.278773	-2.602391	-1.353095
H	6.441277	-1.030390	-2.090211
H	8.708273	-2.916996	-1.766873
H	10.986078	-2.694785	-0.827672
H	11.429268	-1.064851	1.001070
H	9.569139	0.335727	1.876486
H	7.289741	0.112107	0.926231
H	5.700349	-2.488701	1.144202
H	4.343200	-1.433924	1.464654
H	4.250649	-4.384001	1.824374
H	2.707977	-6.153362	1.042204
H	1.473200	-5.875072	-1.102954
H	1.796442	-3.808683	-2.449552
H	3.351863	-2.046922	-1.665988
C	-5.479802	-0.652068	-1.525561
O	-5.423471	0.455984	-0.996439
C	-6.752488	-1.165574	-2.191439
H	-7.611263	-0.582986	-1.851082
H	-6.653420	-1.049878	-3.276872

H -6.906696 -2.229027 -1.986325
C -2.205630 -0.899798 -4.008880
O -1.524087 -1.879879 -3.672028
C -2.475039 -0.597578 -5.479221
H -1.988558 -1.340598 -6.114506
H -3.554096 -0.600738 -5.667673
H -2.103663 0.401670 -5.733239

trans-(AcO) $_{2}$**Pd(N** $_{2}$**Phos)** $_{2}$**:** B3LYP/6-31G(d)/SDD

Processing: pdtac2dinamphos6dsdbe.log
PG=C01

Method	BasisSet	Imaginary Freqs
RB3LYP	GenECP	0

HF Energy
-6283.6808410

ZPE	E298	S298	Squasihar	Equasihar	Strans	Srot
1424.66947	1505.408	674.876	558.576	1505.878	48.534	44.599

Processing: pdtac2dinamphos6dsdbe.log
267
C -8.591004 3.940719 -2.349578
C -7.794628 4.535404 -3.340205
C -7.665880 5.929418 -3.349286
C -8.320376 6.717064 -2.398423
C -9.109767 6.115222 -1.418311
C -9.241474 4.723707 -1.396119
C -7.091329 3.688718 -4.395659
N -6.139753 2.704693 -3.861026
C -5.005191 3.272881 -3.138797
C -4.136099 4.130919 -4.048356
C -3.888722 5.477769 -3.761080
C -3.071096 6.248364 -4.593953
C -2.495589 5.678981 -5.729675
C -2.742898 4.335597 -6.029950
C -3.557099 3.570152 -5.196293
C -6.578293 1.396548 -3.582723
C -6.313886 0.732173 -2.388473
C -6.704568 -0.623382 -2.175283
C -7.419760 -1.290623 -3.224993
C -7.686129 -0.592973 -4.432965
C -7.278071 0.699204 -4.616486
C -6.458114 -1.320932 -0.938875
C -6.933586 -2.635852 -0.789940
C -7.620920 -3.273461 -1.858280
C -7.860892 -2.618726 -3.039523
C -5.892942 -0.615576 0.264614

C	-4.582306	-0.109184	0.462699
C	-4.186475	0.491345	1.693201
C	-5.179624	0.700252	2.675936
C	-6.485607	0.239317	2.484445
C	-6.806069	-0.420828	1.309121
P	-2.407574	1.021956	1.911436
C	-1.832134	1.017069	3.729255
C	-1.967190	2.293571	4.584794
C	-1.150755	2.146213	5.883529
C	-1.569627	0.903651	6.682050
C	-1.470915	-0.363641	5.821370
C	-2.270956	-0.238797	4.512487
O	-3.637028	-0.036789	-0.520804
C	-3.398180	-1.113260	-1.432906
O	-4.820606	1.391567	3.793643
C	-5.776552	1.577511	4.827114
N	-6.757490	-3.345849	0.441875
C	-7.858363	-4.233275	0.827707
C	-7.905478	-4.538465	2.318182
C	-8.335833	-5.799600	2.750432
C	-8.453137	-6.090913	4.111199
C	-8.128318	-5.123246	5.062988
C	-7.688576	-3.865893	4.642132
C	-7.581189	-3.573965	3.281625
Pd	-0.486214	-0.182476	0.961885
O	-1.412103	-2.020068	0.780385
P	1.597613	-1.206101	0.292565
C	1.487164	-2.440927	-1.161240
C	2.736053	-2.478200	-2.066681
C	2.660841	-3.650450	-3.062272
C	1.376017	-3.603064	-3.901277
C	0.132375	-3.532458	-3.003875
C	0.205002	-2.356318	-2.013341
C	2.858186	0.057938	-0.257624
C	4.136665	0.310247	0.292566
C	5.027348	1.255792	-0.281685
C	4.561874	2.008029	-1.364060
C	3.285009	1.844391	-1.886009
C	2.448453	0.864125	-1.347417
C	6.466123	1.412841	0.110679
C	7.373729	0.300634	-0.063054
C	8.761432	0.461162	0.266196
C	9.226101	1.719130	0.718287
C	8.364277	2.779692	0.830531
C	6.978431	2.648635	0.526154
C	9.640363	-0.637186	0.095181
C	9.205845	-1.835039	-0.420374
C	7.840820	-2.006431	-0.788363
C	6.959077	-0.950225	-0.586819
N	7.364573	-3.223218	-1.340190
C	8.149992	-4.434606	-1.105977

C	7.305428	-5.699156	-1.132951
C	7.752261	-6.838604	-1.811156
C	7.007615	-8.021349	-1.790835
C	5.800382	-8.074829	-1.093748
C	5.343416	-6.940046	-0.416674
C	6.091191	-5.762931	-0.435057
N	6.128484	3.802904	0.639482
C	6.716641	5.051743	0.144352
C	5.688236	6.109179	-0.232604
C	5.980291	7.462861	-0.020321
C	5.087529	8.461451	-0.415592
C	3.878684	8.117563	-1.022450
C	3.573263	6.770416	-1.230627
C	4.471211	5.775203	-0.841798
O	4.526611	-0.461578	1.353791
C	4.945990	0.203354	2.552986
O	1.214125	0.600319	-1.856373
C	0.611522	1.546011	-2.729376
C	6.719697	-3.153547	-2.668256
C	7.682888	-3.065700	-3.844508
C	8.324702	-1.858258	-4.160922
C	9.219238	-1.784860	-5.228826
C	9.485703	-2.918102	-6.002048
C	8.850664	-4.123475	-5.700528
C	7.956474	-4.193831	-4.629283
C	2.122966	-2.401697	1.659000
C	3.359432	-3.294182	1.414660
C	3.453457	-4.395795	2.488431
C	3.455148	-3.811894	3.907334
C	2.221528	-2.927444	4.133773
C	2.104911	-1.811880	3.081176
C	5.396591	3.945099	1.920190
C	6.219813	4.399089	3.118746
C	6.257902	5.754366	3.478475
C	7.015076	6.189520	4.568304
C	7.746998	5.270954	5.322458
C	7.713732	3.917041	4.980679
C	6.956617	3.487542	3.889823
O	0.376918	1.682024	0.917280
C	-2.400025	2.828030	1.369515
C	-2.287216	2.985885	-0.160437
C	-2.079010	4.462620	-0.539355
C	-3.190251	5.363210	0.019599
C	-3.359859	5.170032	1.531971
C	-3.567608	3.688960	1.896605
C	-5.411777	-3.916906	0.668546
C	-5.090714	-5.158297	-0.152643
C	-4.669512	-5.059008	-1.487960
C	-4.394984	-6.202496	-2.239656
C	-4.537640	-7.469308	-1.668283
C	-4.953471	-7.583342	-0.340833

C	-5.226004	-6.436337	0.408287
H	-7.253083	0.402578	3.230387
H	-7.821642	-0.775574	1.167843
H	-0.754914	0.899826	3.545307
H	-3.339161	-0.173359	4.744926
H	-2.103499	-1.137113	3.913568
H	-1.825973	-1.237523	6.382987
H	-0.417385	-0.557978	5.576888
H	-2.607401	1.029030	7.029326
H	-0.949887	0.805531	7.583173
H	-1.266949	3.052408	6.493514
H	-0.084261	2.071606	5.628493
H	-3.016436	2.478713	4.832909
H	-1.604218	3.171407	4.037997
H	-1.464390	3.203332	1.801107
H	-4.505215	3.333698	1.447301
H	-3.682174	3.586276	2.976931
H	-4.207160	5.764002	1.899621
H	-2.466227	5.547245	2.051474
H	-4.140069	5.115005	-0.476861
H	-2.982735	6.416561	-0.211482
H	-2.024837	4.565142	-1.630403
H	-1.108244	4.797391	-0.144203
H	-3.203590	2.608646	-0.632185
H	-1.457666	2.381522	-0.533211
H	-2.460148	-0.857265	-1.926978
H	-3.254664	-2.048914	-0.890489
H	-4.195501	-1.198580	-2.173473
H	-5.254088	2.114984	5.620196
H	-6.628667	2.176728	4.482651
H	-6.138229	0.618220	5.217426
H	-8.389035	-3.117719	-3.848979
H	-7.945043	-4.300735	-1.737402
H	-5.816619	1.255629	-1.581149
H	-8.209696	-1.117002	-5.229443
H	-7.446899	1.189764	-5.569600
H	5.236266	2.721506	-1.824906
H	2.967260	2.442276	-2.731550
H	5.120004	-0.589936	3.282330
H	5.877279	0.752983	2.398552
H	4.151468	0.866025	2.911545
H	-0.413321	1.202152	-2.876152
H	0.600021	2.543444	-2.276746
H	1.124337	1.582497	-3.699242
H	1.438797	-3.401745	-0.629572
H	2.805698	-1.536581	-2.625189
H	3.651633	-2.557889	-1.469930
H	3.543322	-3.636213	-3.715198
H	2.700237	-4.599876	-2.507854
H	1.402966	-2.715706	-4.551521
H	1.323179	-4.475799	-4.565132

H	-0.774327	-3.443188	-3.617292
H	0.035374	-4.474796	-2.443813
H	0.200024	-1.412388	-2.568064
H	-0.667377	-2.359721	-1.357296
H	1.240599	-3.057757	1.607392
H	2.919703	-1.098501	3.203162
H	1.176524	-1.260589	3.235835
H	2.254407	-2.477397	5.134875
H	1.312179	-3.541488	4.096138
H	4.369348	-3.215281	4.049889
H	3.491714	-4.620421	4.649531
H	4.357677	-4.995913	2.317625
H	2.598023	-5.080517	2.383773
H	4.263627	-2.679840	1.434219
H	3.313905	-3.767304	0.426740
H	8.733100	3.736912	1.181760
H	10.277880	1.834783	0.970371
H	5.916505	-1.092648	-0.838818
H	10.689758	-0.506438	0.349809
H	9.918196	-2.636589	-0.580958
H	4.592152	4.666444	1.748851
H	4.920705	2.984886	2.128690
H	5.680815	6.473585	2.901571
H	7.026819	7.244213	4.831693
H	8.333182	5.605489	6.174408
H	8.271919	3.192843	5.568575
H	6.934263	2.430810	3.638631
H	7.424970	5.505744	0.857695
H	7.301707	4.793453	-0.747306
H	6.917791	7.737163	0.459089
H	5.334087	9.505827	-0.242000
H	3.178289	8.891417	-1.325411
H	2.631159	6.492584	-1.696775
H	4.228155	4.728788	-0.994999
H	-5.334281	-4.156212	1.733590
H	-4.685245	-3.126315	0.469248
H	-4.552667	-4.078506	-1.941168
H	-4.064018	-6.103949	-3.270398
H	-4.320492	-8.359878	-2.252271
H	-5.060522	-8.564432	0.115002
H	-5.541899	-6.531308	1.444776
H	-8.788484	-3.727410	0.540060
H	-7.845865	-5.192920	0.283781
H	-7.227888	-2.600479	2.956559
H	-7.424878	-3.109082	5.376794
H	-8.210142	-5.348578	6.123005
H	-8.789331	-7.075587	4.425820
H	-8.582217	-6.561013	2.013122
H	-6.555399	4.349743	-5.085684
H	-7.838072	3.152904	-4.986351
H	-5.322591	3.864384	-2.265937

H	-4.409089	2.438050	-2.759051
H	-7.047505	6.402146	-4.109238
H	-8.210244	7.798221	-2.423323
H	-9.619525	6.724047	-0.676250
H	-9.856312	4.247717	-0.636513
H	-8.699144	2.859779	-2.324247
H	-4.341529	5.927532	-2.881127
H	-2.889750	7.293189	-4.355355
H	-1.861265	6.276041	-6.379537
H	-2.299906	3.885692	-6.914912
H	-3.759211	2.529195	-5.436259
H	8.984497	-4.544217	-1.818946
H	8.595229	-4.339618	-0.109342
H	6.090855	-4.042681	-2.776437
H	6.052170	-2.289192	-2.669011
H	8.691490	-6.800072	-2.358939
H	7.369950	-8.896901	-2.323499
H	5.217637	-8.992063	-1.078471
H	4.405110	-6.973549	0.131144
H	5.735050	-4.877765	0.084534
H	7.460288	-5.134386	-4.400343
H	9.047237	-5.008852	-6.299968
H	10.179796	-2.859222	-6.836396
H	9.704151	-0.840397	-5.462351
H	8.118165	-0.971140	-3.568258
C	1.226470	2.067293	1.829188
C	-1.376204	-2.896423	1.746770
O	1.636615	1.388611	2.774113
C	1.705296	3.502170	1.616278
O	-0.956573	-2.701045	2.889194
C	-1.873388	-4.285613	1.350289
H	2.237383	3.855963	2.501564
H	2.379357	3.538142	0.753480
H	0.862844	4.166688	1.399530
H	-2.602935	-4.638489	2.085364
H	-2.312253	-4.315016	0.351918
H	-1.025254	-4.979980	1.383651

Ph(Cl)Pd(EvanPhos), Cl trans to P: B3LYP/6-31G(d)/SDD

Processing: pdclphevanphos-s6dsdbe.log
PG=C01

Method	BasisSet	Imaginary Freqs
RB3LYP	GenECP	0

HF Energy
-2591.6226650

ZPE	E298	S298	Squasihar	Equasihar	Strans	Srot

461.21604 488.063 254.462 232.377 488.182 45.553 38.486

Processing: pdclphevanphos-s6dsdbe.log
87

Pd	0.796818	-1.437774	-0.580900
P	1.312171	0.763685	-0.056995
C	-0.318829	1.341972	0.643069
C	-0.517553	2.447720	1.505115
C	-1.780701	2.713269	2.041986
C	-2.853611	1.878474	1.740151
C	-2.723194	0.786878	0.878623
C	-1.450301	0.571082	0.316269
O	0.570457	3.228986	1.772980
C	0.433822	4.316575	2.678623
H	1.425165	4.766946	2.750047
H	0.116413	3.973815	3.670941
H	-0.278109	5.062928	2.305010
H	-1.933168	3.549810	2.712670
H	-3.818499	2.066727	2.201693
C	-1.911085	-0.413889	-1.848933
H	-2.973364	-0.195559	-1.733753
H	-1.766759	-1.395639	-2.299526
H	-1.424068	0.361754	-2.450978
O	-1.274072	-0.500856	-0.551236
C	-3.862316	-0.147414	0.647556
C	-3.748496	-1.466948	1.092395
C	-4.803249	-2.393397	0.883263
C	-5.965474	-1.991530	0.269441
C	-6.140290	-0.658901	-0.179367
C	-5.068073	0.278826	0.004813
C	-5.249786	1.599478	-0.501556
C	-6.422476	1.971800	-1.121299
C	-7.484656	1.049090	-1.277852
C	-7.337814	-0.239364	-0.817954
H	-4.696503	-3.418576	1.216910
H	-6.771305	-2.705927	0.119183
H	-8.139209	-0.964301	-0.941616
H	-8.404887	1.358007	-1.765578
H	-6.532453	2.985706	-1.497438
H	-4.444993	2.319653	-0.397308
O	-2.608828	-1.791741	1.754536
C	-2.281243	-3.167424	1.952324
H	-2.997352	-3.655725	2.625889
H	-2.218352	-3.698074	0.997042
H	-1.294822	-3.165800	2.416934
C	1.545228	1.888658	-1.560892
C	2.396464	1.234881	-2.670208
C	2.428964	2.111949	-3.933833
C	2.909934	3.537910	-3.629574
C	2.060969	4.182797	-2.525115
C	2.032776	3.319641	-1.250619

H	0.517221	1.964790	-1.946653
H	1.992211	0.245264	-2.909353
H	3.421895	1.074976	-2.312158
H	1.418540	2.155961	-4.367355
H	3.071516	1.643543	-4.689832
H	2.881848	4.151672	-4.538715
H	3.960921	3.505202	-3.306000
H	1.032523	4.321029	-2.890746
H	2.442404	5.183203	-2.282980
H	1.396419	3.785148	-0.492039
H	3.046472	3.277745	-0.829304
C	2.639398	1.171638	1.200969
C	2.355510	0.447372	2.535042
C	3.434008	0.768072	3.584054
C	4.841506	0.440717	3.065923
C	5.119623	1.163168	1.740703
C	4.054635	0.831498	0.679877
H	2.332396	-0.635018	2.358450
H	1.367917	0.728343	2.918458
H	3.224977	0.211015	4.506293
H	3.381460	1.836144	3.844529
H	5.596305	0.713012	3.814748
H	4.927019	-0.643680	2.907952
H	5.137937	2.249906	1.915147
H	6.110740	0.890253	1.357377
H	4.271083	1.391402	-0.237295
H	4.114783	-0.230857	0.424243
H	2.585651	2.249415	1.377586
Cl	-0.103921	-3.531707	-1.208259
C	2.621343	-2.230314	-0.500618
C	3.031248	-2.877057	0.674505
C	4.297530	-3.466877	0.757344
C	5.166640	-3.430032	-0.335114
C	4.753025	-2.810686	-1.515655
C	3.488170	-2.216870	-1.600591
H	2.360243	-2.937949	1.526911
H	4.596720	-3.966034	1.676656
H	6.149294	-3.890357	-0.271224
H	5.411630	-2.791773	-2.381472
H	3.182281	-1.754430	-2.534032

cis-Ph(Cl)Pd(EvanPhos)$_2$: B3LYP/6-31G(d)/SDD

Processing: pdcclphdievanphos-x6dsdbe.log
PG=C01

Method	BasisSet	Imaginary Freqs
RB3LYP	GenECP	0

HF Energy

-4363.3638978

ZPE	E298	S298	Squasihar	Equasihar	Strans	Srot
865.13030	913.980	416.659	367.065	914.204	47.121	41.572

Processing: pdcclphdievanphos-x6dsdbe.log
161

C	2.989008	0.650664	3.017767
C	1.733034	1.177690	2.697152
C	1.183255	2.115951	3.591370
C	1.873239	2.534585	4.731487
C	3.135400	2.011341	5.023986
C	3.683349	1.061564	4.164338
Pd	0.506831	0.670552	1.122782
Cl	-0.756277	-0.434354	2.863023
P	2.137274	1.598630	-0.397122
C	3.867505	0.913378	-0.216298
C	5.058497	1.661683	-0.347158
C	6.307699	1.070373	-0.130815
C	6.383415	-0.290998	0.136539
C	5.249888	-1.105160	0.177074
C	3.992306	-0.482134	0.011733
O	4.935304	2.969898	-0.719190
C	6.106160	3.763808	-0.840885
C	5.444681	-2.583033	0.313062
C	5.421510	-3.432610	-0.835868
C	5.657000	-4.840969	-0.685272
C	5.923500	-5.351663	0.608288
C	5.954384	-4.527166	1.709595
C	5.712046	-3.137774	1.562929
C	5.623885	-5.683038	-1.830090
C	5.370930	-5.168786	-3.080337
C	5.144370	-3.778658	-3.235207
C	5.169823	-2.935139	-2.147470
O	2.826376	-1.188413	-0.023807
C	2.548806	-2.246455	0.910622
O	5.691914	-2.274121	2.622703
C	5.895839	-2.780358	3.934110
C	1.998650	3.488768	-0.074797
C	2.781561	4.021794	1.146895
C	2.253417	5.404449	1.566991
C	2.313849	6.413313	0.412064
C	1.594629	5.872503	-0.831173
C	2.122017	4.484933	-1.246817
C	1.924286	1.397378	-2.285782
C	3.099165	1.934455	-3.138321
C	2.789666	1.840821	-4.643819
C	2.476243	0.401193	-5.068958
C	1.352699	-0.186351	-4.205685
C	1.660612	-0.065016	-2.702301
P	-1.538066	0.093011	-0.374496

C	-1.231595	-1.731354	-0.856033
C	-1.720616	-2.254111	-2.222014
C	-1.123499	-3.642137	-2.523851
C	-1.426846	-4.652167	-1.408857
C	-0.951630	-4.125109	-0.047910
C	-1.532842	-2.737029	0.277396
C	-1.846445	1.035574	-1.999380
C	-3.197923	0.782135	-2.703442
C	-3.283286	1.534711	-4.043978
C	-3.065087	3.043185	-3.866378
C	-1.749435	3.324253	-3.127152
C	-1.670910	2.558109	-1.795322
C	-3.169479	0.354029	0.502331
C	-3.235889	1.467775	1.374057
C	-4.365465	1.709541	2.159781
C	-5.476055	0.892838	2.014312
C	-5.517109	-0.156877	1.092704
C	-4.340801	-0.429903	0.353929
O	-2.165604	2.310475	1.372850
C	-2.148928	3.393932	2.289576
C	-6.840962	-0.837189	0.914809
C	-7.274357	-1.770784	1.854613
C	-8.557538	-2.367105	1.757879
C	-9.398681	-2.029505	0.722413
C	-9.011712	-1.085096	-0.259205
C	-7.716216	-0.473691	-0.155714
C	-7.350705	0.484746	-1.145166
C	-8.208761	0.810449	-2.171263
C	-9.483782	0.200561	-2.271641
C	-9.872557	-0.725245	-1.331715
O	-4.280511	-1.410514	-0.600605
C	-5.013716	-2.629035	-0.458732
O	-6.380944	-2.095655	2.836555
C	-6.774055	-2.989203	3.865777
H	-1.200525	3.906716	2.123499
H	-2.194954	3.038182	3.325170
H	-2.976035	4.090789	2.099773
H	-4.465973	-3.372562	-1.043347
H	-5.053155	-2.947812	0.586921
H	-6.025915	-2.534694	-0.858497
H	-4.395436	2.534569	2.859442
H	-6.366978	1.097985	2.600956
H	-6.380877	0.965015	-1.073353
H	-7.906384	1.545596	-2.912592
H	-10.149716	0.468173	-3.087428
H	-10.850295	-1.198177	-1.392991
H	-10.380472	-2.491629	0.650977
H	-8.879384	-3.094248	2.494432
H	-1.435468	-1.565482	-3.026047
H	-2.812161	-2.315316	-2.229561
H	-1.507194	-4.005480	-3.486647

H	-0.032798	-3.550502	-2.637872
H	-1.126271	-2.370234	1.223061
H	-2.612817	-2.821404	0.418752
H	0.146920	-4.067584	-0.052390
H	-1.220846	-4.828906	0.750622
H	-0.956242	-5.618630	-1.632936
H	-2.511350	-4.836357	-1.371014
H	-0.134936	-1.693504	-0.921265
H	-1.044335	0.692926	-2.666825
H	-3.370900	-0.282572	-2.863622
H	-4.005637	1.129086	-2.046215
H	-2.524653	1.135855	-4.734636
H	-4.258037	1.342963	-4.511654
H	-3.074398	3.551483	-4.839390
H	-3.900067	3.462042	-3.285261
H	-1.637873	4.401033	-2.942302
H	-0.905255	3.029491	-3.769450
H	-0.724313	2.772843	-1.289959
H	-2.458721	2.922068	-1.124505
H	2.725546	-3.216977	0.439124
H	3.157415	-2.151288	1.810813
H	1.497717	-2.134610	1.183333
H	6.647730	3.833171	0.110678
H	6.777930	3.371580	-1.614781
H	5.761204	4.757682	-1.131842
H	6.896834	-3.216642	4.048534
H	5.797706	-1.920958	4.599180
H	5.139532	-3.528960	4.201957
H	6.158777	-4.946660	2.687683
H	6.106294	-6.417041	0.726482
H	5.804460	-6.747337	-1.696358
H	5.347233	-5.821554	-3.948528
H	4.951086	-3.374209	-4.225443
H	5.006320	-1.871083	-2.280886
H	7.357473	-0.751614	0.274412
H	7.219165	1.650514	-0.199389
H	3.351737	2.961693	-2.879239
H	3.990420	1.331622	-2.922608
H	3.640909	2.232199	-5.216490
H	1.931794	2.487824	-4.881821
H	3.381268	-0.213578	-4.952675
H	2.205132	0.362349	-6.131917
H	0.413666	0.340284	-4.433032
H	1.186068	-1.241301	-4.459622
H	2.541315	-0.673381	-2.467529
H	0.842674	-0.487438	-2.119405
H	1.026893	1.984909	-2.520649
H	1.560664	4.144545	-2.123552
H	3.171589	4.574527	-1.547354
H	1.702068	6.571528	-1.671221
H	0.516404	5.799480	-0.623830

H	3.367186	6.610805	0.160966
H	1.880410	7.375748	0.714160
H	2.831236	5.770023	2.425551
H	1.213523	5.306966	1.914041
H	3.838909	4.106007	0.884748
H	2.709066	3.334209	1.988183
H	0.938140	3.494351	0.217768
H	-5.916271	-3.059391	4.536792
H	-7.640475	-2.609778	4.423396
H	-7.007192	-3.988037	3.473099
H	0.192580	2.516160	3.408253
H	1.415747	3.263096	5.398455
H	3.674771	2.332182	5.912067
H	4.662105	0.632839	4.373073
H	3.464169	-0.085308	2.383811

Ph(Cl)Pd(N$_2$Phos) Cl cis to P: B3LYP/6-31G(d)/SDD

Processing: pdclphnamphos-a6dsdbe.log
PG=C01

Method	BasisSet	Imaginary Freqs
RB3LYP	GenECP	0

HF Energy
-3669.1781196

ZPE	E298	S298	Squasihar	Equasihar	Strans	Srot
736.86273	778.681	381.793	321.796	778.926	46.779	41.271

Processing: pdclphnamphos-a6dsdbe.log
139

Pd	-2.914720	-0.841653	-1.248245
P	-2.279405	-2.125707	0.694696
C	-0.887118	-1.101508	1.387793
C	-0.331751	-1.293518	2.675663
C	0.704641	-0.481157	3.139261
C	1.167311	0.547689	2.332257
C	0.638002	0.823241	1.062969
C	-0.371395	-0.048413	0.595152
O	-0.848795	-2.306868	3.427052
H	-0.932133	-3.350305	5.144891
H	0.709215	-2.779156	4.737816
H	-0.509206	-1.630104	5.373375
H	1.140487	-0.629044	4.119541
H	1.958542	1.192657	2.702703
O	-0.909865	0.137114	-0.686687
C	0.044543	-0.000627	-1.775364
H	0.415489	-1.030454	-1.807973

H	0.867565	0.699787	-1.643537
H	-0.509669	0.236354	-2.681424
C	1.220459	2.025862	0.374410
C	0.553816	3.264254	0.300214
C	1.272691	4.397350	-0.191167
C	2.575914	4.308739	-0.604725
C	3.268205	3.078470	-0.565569
C	2.591814	1.921559	-0.063196
C	3.296019	0.684683	-0.063503
C	4.619477	0.576770	-0.491801
C	5.283644	1.758047	-0.948266
C	4.614799	2.955363	-0.990222
N	5.303226	-0.640063	-0.469656
C	4.813542	-1.790129	0.271590
C	4.031473	-2.819895	-0.541048
C	6.506566	-0.870792	-1.255680
C	7.825518	-0.760037	-0.495724
H	5.679167	-2.288568	0.728755
H	4.203818	-1.430225	1.108089
H	6.508461	-0.181323	-2.107182
H	6.435635	-1.874369	-1.696394
C	7.929381	-0.062068	0.712098
C	9.159221	0.046041	1.367034
C	10.300883	-0.541723	0.821637
C	10.205734	-1.243676	-0.383071
C	8.976541	-1.353889	-1.032131
H	7.042459	0.395048	1.140773
H	9.222609	0.591265	2.305272
H	11.256894	-0.458164	1.331433
H	11.087771	-1.711153	-0.813049
H	8.908093	-1.908949	-1.966067
C	3.912196	-4.130053	-0.057804
C	3.194652	-5.092454	-0.768688
C	2.588534	-4.758822	-1.983328
C	2.705852	-3.457778	-2.475743
C	3.422351	-2.495074	-1.758872
H	4.393470	-4.401286	0.880150
H	3.120859	-6.106035	-0.383228
H	2.040800	-5.509797	-2.545859
H	2.248201	-3.192269	-3.425376
H	3.518429	-1.486497	-2.149709
N	-0.807814	3.432961	0.662428
C	-1.252091	3.101788	2.030389
C	-1.537090	4.598319	0.138422
H	-0.738945	2.198710	2.354147
H	-2.320172	2.857107	1.977294
H	-2.583574	4.435970	0.430523
H	-1.235388	5.532925	0.639293
C	-1.045249	4.200667	3.066676
C	-2.142760	4.814223	3.682796
C	-1.964266	5.810829	4.645503

C	-0.677065	6.213740	5.002004
C	0.427501	5.612258	4.392311
C	0.244675	4.613402	3.435987
H	-3.149426	4.505441	3.408052
H	-2.830335	6.272503	5.112749
H	-0.533619	6.989930	5.749043
H	1.433829	5.919500	4.665585
H	1.110447	4.153005	2.967586
C	-1.478484	4.797906	-1.364386
C	-1.522559	3.718882	-2.253360
C	-1.498724	3.936316	-3.631997
C	-1.442151	5.236264	-4.139214
C	-1.411405	6.320054	-3.259859
C	-1.425208	6.098772	-1.882037
H	-1.578207	2.707967	-1.863234
H	-1.543646	3.087318	-4.306749
H	-1.426091	5.403191	-5.213181
H	-1.368737	7.336308	-3.643753
H	-1.389555	6.946058	-1.199367
C	-1.504420	-3.806305	0.294038
C	-2.250417	-4.477534	-0.883854
C	-1.536515	-5.761640	-1.336925
C	-1.347968	-6.749381	-0.177259
C	-0.612040	-6.084146	0.993615
C	-1.318971	-4.798486	1.461482
H	-0.504161	-3.524318	-0.069450
H	-2.343424	-3.777487	-1.720117
H	-3.278086	-4.719738	-0.584277
H	-0.751173	-4.333626	2.270477
H	-2.301485	-5.063099	1.875914
H	-0.523735	-6.781478	1.837435
H	0.413631	-5.835462	0.683097
H	-2.105487	-6.228668	-2.150724
H	-0.551272	-5.499314	-1.751091
H	-0.800728	-7.638962	-0.515582
H	-2.333255	-7.099505	0.164662
C	-3.527582	-2.325605	2.084076
C	-4.715556	-3.218206	1.658737
C	-5.749103	-3.330415	2.793688
C	-6.248136	-1.951247	3.246235
C	-5.074759	-1.049842	3.654678
C	-4.031333	-0.934907	2.530367
H	-3.018944	-2.797656	2.929256
H	-3.191791	-0.313957	2.866701
H	-4.480314	-0.423402	1.667614
H	-4.594177	-1.463444	4.554338
H	-5.435610	-0.049109	3.925452
H	-6.797711	-1.477709	2.420244
H	-6.956365	-2.054848	4.078502
H	-6.591192	-3.949302	2.459112
H	-5.297652	-3.851975	3.651873

H -5.188743 -2.801600 0.762793
H -4.364819 -4.221273 1.389397
H 5.130250 3.845732 -1.343317
H 6.328515 1.721339 -1.234525
H 3.093346 5.196595 -0.961075
H 2.765341 -0.205468 0.246270
H 0.780866 5.359562 -0.219723
Cl -4.916034 -1.914532 -1.748583
C -3.305154 0.245214 -2.917281
C -0.355633 -2.515176 4.744008
C -4.228336 1.300287 -2.905423
C -4.514704 2.012852 -4.073747
C -3.894215 1.672313 -5.278483
C -2.980326 0.617434 -5.305838
C -2.694864 -0.095512 -4.133711
H -4.747457 1.557942 -1.985456
H -5.231950 2.830084 -4.043299
H -4.128153 2.218830 -6.188839
H -2.502606 0.331379 -6.240934
H -2.014706 -0.944374 -4.188136

cis-Ph(Cl)Pd(N$_2$Phos)$_2$: B3LYP/6-31G(d)/SDD

Processing: pdclphdinamphos6dsdbe.log
PG=C01

Method	BasisSet	Imaginary Freqs
RB3LYP	GenECP	0

HF Energy
-6518.5327089

ZPE	E298	S298	Squasihar	Equasihar	Strans	Srot
1417.29183	1495.821	655.397	542.856	1496.276	48.524	44.543

Processing: pdclphdinamphos6dsdbe.log
265
C -5.786108 6.189550 0.984112
C -6.197729 6.476757 -0.324380
C -6.157607 7.806096 -0.761824
C -5.727801 8.828605 0.087565
C -5.322121 8.531475 1.389034
C -5.349779 7.206691 1.832962
C -6.740649 5.392493 -1.243314
N -5.943679 4.161994 -1.234295
C -4.547213 4.348312 -1.675257
C -4.377398 4.902043 -3.085628
C -3.828809 6.177457 -3.278838
C -3.651432 6.700482 -4.562078
C -4.029626 5.954785 -5.679024

C	-4.584621	4.684725	-5.502565
C	-4.754591	4.164128	-4.219103
C	-6.617726	2.961893	-1.604208
C	-6.239354	1.709371	-1.089435
C	-7.029091	0.546051	-1.415210
C	-8.130705	0.665482	-2.327663
C	-8.447050	1.936778	-2.860813
C	-7.726225	3.045594	-2.499881
C	-8.892751	-0.485289	-2.646345
C	-8.635120	-1.703684	-2.064437
C	-7.575120	-1.838024	-1.123385
C	-6.787782	-0.728247	-0.838429
C	-5.109081	1.585481	-0.111501
C	-3.956996	0.773999	-0.297066
C	-3.002332	0.582314	0.728938
C	-3.176246	1.329493	1.918944
C	-4.280841	2.160947	2.108814
C	-5.235058	2.259141	1.106167
P	-1.526755	-0.581945	0.745889
C	-2.068991	-1.881176	2.048520
C	-3.596735	-2.091764	2.153527
C	-3.921281	-3.337892	2.999056
C	-3.293724	-3.258827	4.397916
C	-1.786452	-2.978834	4.315978
C	-1.492833	-1.728172	3.468482
O	-3.768307	0.066948	-1.459051
C	-3.804755	0.759756	-2.710339
O	-2.215425	1.165738	2.872173
C	-2.247244	1.983670	4.033361
N	-7.304364	-3.073847	-0.476325
C	-7.786898	-4.294375	-1.123311
C	-6.970269	-5.522440	-0.752443
C	-7.604196	-6.735226	-0.460051
C	-6.858252	-7.882896	-0.177222
C	-5.464251	-7.827929	-0.177725
C	-4.821724	-6.619611	-0.463836
C	-5.569178	-5.477484	-0.750426
Pd	0.641148	0.452064	0.838116
P	2.249838	-1.254349	2.069723
C	2.065710	-0.386085	3.778703
C	2.023079	-1.225024	5.072734
C	1.532082	-0.372062	6.258515
C	2.391038	0.885240	6.452667
C	2.468380	1.705230	5.157518
C	2.958262	0.861379	3.967366
C	-0.224462	2.173268	0.180399
C	-0.440768	3.197336	1.113786
C	-0.865947	4.465723	0.707311
C	-1.078958	4.738543	-0.645987
C	-0.863252	3.728442	-1.585536
C	-0.439399	2.457622	-1.174760

Cl	2.654457	1.753997	0.553710
C	1.867792	-3.097170	2.358822
C	1.619823	-3.820632	1.017460
C	1.103984	-5.253034	1.241392
C	2.075596	-6.077076	2.096024
C	2.389261	-5.358041	3.413550
C	2.886196	-3.919320	3.180007
C	4.039627	-1.297844	1.534309
C	4.338171	-1.195735	0.145987
C	5.669599	-1.049296	-0.316484
C	6.683255	-1.048532	0.650222
C	6.440105	-1.237837	2.002218
C	5.120444	-1.377955	2.439831
O	3.261908	-1.309009	-0.678893
C	3.192656	-0.569776	-1.906157
C	6.138822	-0.846688	-1.731025
C	6.632992	0.463173	-2.075871
C	7.215888	0.684497	-3.364681
C	7.300881	-0.391586	-4.273175
C	6.823707	-1.634554	-3.936326
C	6.227247	-1.888459	-2.666216
C	7.703366	1.980341	-3.681875
C	7.624857	3.017690	-2.791093
C	7.010688	2.837143	-1.510253
C	6.517880	1.573614	-1.190662
O	4.824403	-1.597886	3.756170
C	5.886429	-1.675305	4.694049
N	5.694375	-3.189138	-2.397555
C	5.295965	-3.967307	-3.575277
C	4.218373	-5.002252	-3.301297
C	4.333691	-6.291173	-3.835553
C	3.301595	-7.222522	-3.694293
C	2.134744	-6.870698	-3.016101
C	2.015183	-5.588646	-2.471348
C	3.050550	-4.662854	-2.604516
N	6.986717	3.904932	-0.600654
C	6.719999	3.636351	0.814824
C	7.413164	4.623409	1.739267
C	8.805496	4.785522	1.690560
C	9.452011	5.657602	2.565566
C	8.714012	6.380586	3.507898
C	7.328408	6.227647	3.563010
C	6.683236	5.356554	2.680652
C	6.697066	5.269365	-1.055461
C	5.267928	5.752427	-0.829658
C	5.042082	7.090277	-0.480321
C	3.743853	7.575809	-0.313880
C	2.652667	6.719542	-0.476346
C	2.866660	5.380186	-0.809111
C	4.165977	4.903375	-0.992412
C	6.360925	-4.006021	-1.358407

C	7.683269	-4.637089	-1.772935
C	8.852324	-3.866594	-1.873259
C	10.060770	-4.447478	-2.258823
C	10.123957	-5.813208	-2.548001
C	8.970434	-6.592146	-2.448591
C	7.761788	-6.005620	-2.064974
C	-1.400594	-1.742049	-0.757615
C	-2.560096	-2.701445	-1.102569
C	-2.144967	-3.701172	-2.200325
C	-1.615359	-2.997005	-3.455099
C	-0.443036	-2.073715	-3.100135
C	-0.822657	-1.057361	-2.010042
C	-7.405025	-3.082394	0.999812
C	-8.824399	-3.123945	1.549112
C	-9.617094	-1.966296	1.584951
C	-10.922999	-2.011190	2.073576
C	-11.458791	-3.215261	2.538334
C	-10.679384	-4.372462	2.512507
C	-9.372388	-4.324205	2.021272
H	7.271259	-1.275000	2.694666
H	7.708630	-0.920989	0.314126
H	1.047415	0.006431	3.638530
H	3.989770	0.546941	4.152653
H	2.961175	1.467276	3.058701
H	3.131901	2.569418	5.291650
H	1.472454	2.114196	4.926213
H	3.406831	0.584133	6.750370
H	1.994022	1.495132	7.275120
H	1.528302	-0.979956	7.173311
H	0.487310	-0.073042	6.081141
H	3.021674	-1.609872	5.297424
H	1.363218	-2.093280	4.962220
H	0.916977	-3.088138	2.909000
H	3.836233	-3.950301	2.631109
H	3.097512	-3.445718	4.138227
H	3.142004	-5.918043	3.984266
H	1.483400	-5.331401	4.038408
H	3.008151	-6.226360	1.532659
H	1.663397	-7.075806	2.290598
H	0.942061	-5.747106	0.276102
H	0.124155	-5.211242	1.742225
H	2.556285	-3.854967	0.446047
H	0.911567	-3.257230	0.403247
H	2.128269	-0.452420	-2.111418
H	3.638988	0.418433	-1.791782
H	3.669507	-1.122737	-2.718067
H	5.415711	-1.857462	5.662131
H	6.568254	-2.503062	4.461829
H	6.455034	-0.737980	4.740401
H	7.765050	-0.230556	-5.243905
H	6.937415	-2.449528	-4.640941

H	5.993525	1.427390	-0.256735
H	8.171799	2.135276	-4.651545
H	8.063477	3.975010	-3.051090
H	-6.120165	2.863821	1.269610
H	-4.414215	2.707566	3.033944
H	-3.516033	0.022188	-3.461962
H	-4.805019	1.129128	-2.942131
H	-3.085647	1.586052	-2.714614
H	-1.346089	1.733426	4.595498
H	-2.228145	3.046446	3.766228
H	-3.130966	1.773749	4.649567
H	-1.632599	-2.800745	1.630820
H	-4.045673	-1.209060	2.626090
H	-4.060080	-2.182697	1.166767
H	-5.009304	-3.455190	3.078567
H	-3.550486	-4.234481	2.480493
H	-3.778640	-2.449893	4.964238
H	-3.482842	-4.186077	4.953617
H	-1.368019	-2.850078	5.322871
H	-1.278014	-3.850251	3.876250
H	-1.940797	-0.855512	3.950476
H	-0.419230	-1.541898	3.426566
H	-0.587100	-2.370010	-0.364744
H	-1.547547	-0.347354	-2.411785
H	0.060715	-0.479537	-1.719322
H	-0.091587	-1.538347	-3.991875
H	0.402781	-2.684922	-2.753625
H	-2.425306	-2.406912	-3.909724
H	-1.306297	-3.735458	-4.205644
H	-3.001312	-4.340669	-2.451235
H	-1.362611	-4.369348	-1.808343
H	-3.429306	-2.128170	-1.435195
H	-2.864636	-3.269339	-0.217392
H	-7.979806	4.006889	-2.931453
H	-9.274278	2.025609	-3.561475
H	-5.965613	-0.841720	-0.143250
H	-9.721561	-0.382093	-3.343234
H	-9.269919	-2.551609	-2.295811
H	-4.059251	5.023128	-0.967065
H	-4.036257	3.390574	-1.576469
H	-3.539262	6.766860	-2.412227
H	-3.218355	7.689640	-4.687013
H	-3.892493	6.357659	-6.679039
H	-4.880791	4.095481	-6.366846
H	-5.190383	3.176711	-4.098167
H	-6.844613	5.819696	-2.254615
H	-7.749862	5.123779	-0.909655
H	-6.463496	8.041962	-1.778617
H	-5.704109	9.854357	-0.271223
H	-4.982439	9.323490	2.051142
H	-5.030759	6.965836	2.843945

H	-5.793528	5.159349	1.325842
H	5.658443	-4.795738	-1.071648
H	6.506953	-3.374572	-0.481676
H	8.814566	-2.804608	-1.646765
H	10.955973	-3.834591	-2.327929
H	11.066527	-6.266071	-2.844520
H	9.009997	-7.656473	-2.666976
H	6.865867	-6.617615	-1.985883
H	4.899210	-3.257118	-4.308840
H	6.150733	-4.471673	-4.056006
H	2.965637	-3.672820	-2.164732
H	1.106209	-5.305567	-1.947987
H	1.324829	-7.587781	-2.910432
H	3.410089	-8.218415	-4.116306
H	5.238267	-6.564808	-4.374406
H	5.642701	3.617283	1.035228
H	7.105810	2.634498	1.027920
H	7.385005	5.962831	-0.557630
H	6.922552	5.323454	-2.123025
H	5.602155	5.247593	2.717642
H	6.745664	6.790265	4.287985
H	9.217397	7.059476	4.191523
H	10.532125	5.771852	2.516126
H	9.377712	4.225697	0.954503
H	5.890881	7.755813	-0.334051
H	3.587361	8.617775	-0.044392
H	1.639728	7.086879	-0.333113
H	2.027930	4.698956	-0.912075
H	4.315885	3.857050	-1.241136
H	-8.850969	-4.494859	-0.913352
H	-7.702331	-4.137314	-2.204626
H	-6.847959	-3.952625	1.360437
H	-6.888137	-2.194529	1.371042
H	-8.691116	-6.782067	-0.455038
H	-7.367788	-8.816293	0.047757
H	-4.881537	-8.717834	0.044944
H	-3.735781	-6.568711	-0.468121
H	-5.073985	-4.534874	-0.965716
H	-8.766548	-5.227609	2.008263
H	-11.085508	-5.312308	2.877943
H	-12.474908	-3.248697	2.922617
H	-11.521241	-1.103939	2.097963
H	-9.205738	-1.024737	1.231299
H	-0.249102	1.703300	-1.931693
H	-1.011023	3.930350	-2.644501
H	-1.395819	5.727724	-0.966455
H	-1.011066	5.247961	1.450018
H	-0.237988	3.020839	2.165727
