# Supplementary material for: N2Phos – an easily made, highly effective ligand designed for ppm level Pd-catalyzed Suzuki–Miyaura cross couplings in water
Source: Chem Sci. 2020 May 7;11(20):5205–12. doi: 10.1039/d0sc00968g (PMC8159421; doi:10.1039/d0sc00968g)
Supplement: SC-011-D0SC00968G-s002 [file SC-011-D0SC00968G-s002.pdf]

Electronic Supplementary Material (ESI) for Chemical Science.
This journal is © The Royal Society of Chemistry 2020

N_2Phos. An easily made, highly effective ligand designed for ppm level Pd-catalyzed Suzuki-Miyaura cross couplings in water

Nnamdi Akporji,[1] Ruchita Thakore,[1] Margery Cortes-Clerget,[1] Joel Anderson,[3] Evan Landstrom [1] Donald H. Aue,[1] Fabrice Gallou,[2] and Bruce H. Lipshutz*[1]

[1]Department of Chemistry and Biochemistry, University of California, Santa Barbara, CA 93106 USA
[2]Novartis Pharma, Basel, Switzerland
[3]Department of Chemistry, University of Cincinnati, Cincinnati OH 45221 USA

*Phone: 805-893-2521
Fax: 805-893-8265
Email: lipshutz@chem.ucsb.edu
Website: http//:www.chem.uscb.edu/liipshutzgroup

Supporting Information

Table of Contents

General Information 2
Synthesis and Analytical Data for N_2Phos 3
Reaction Optimization 7
Recycle Study 11
General procedure for catalyst solution preparation 12
General procedure for Suzuki-Miyaura couplings (SMC) 12
Analytical data for products from Suzuki-Miyaura couplings 14
General Procedure for Hydration of Alkynes 27
Analytical Data for Products from Hydration of Alkynes 28
Procedure for the 1-pot process 29
^{1}H, ^{13}C, ^{31}P and ^{19}F NMR of synthesized products 31

General Information

Unless otherwise specified, all of the reactions were run under and inert atmosphere of argon. Silica gel TLC plates (UV 254 indicator, thickness 200 mm standard grade, glass backed and 230-400 mesh from Merck) or Aluminum Oxide 60 F254 polyester basked plates (Sigma-Aldrich, 0.2 mm thick) were used. The developed TLC plate was analyzed by a UV lamp (254 nm). The plates were further analyzed with the use of an aqueous potassium permanganate stain or butanolic vanillin and developed with a heat gun. All commercially available reagents were used without further purification. A 2 wt % TPGS-750-M/H_2O solution was prepared by dissolving TPGS-750-M in degassed HPLC grade water. TPGS-750-M was made as described previously[1] and is also commercially available. Tetrahydrofuran (THF) and toluene were all taken from an Innovative Technologies Solvent Purification System (SOS) and used immediately. *N,N*-Dimethylformamide (DMF) and dimethylsulfoxide (DMSO) were stored over activated 4Å molecular sieves and were purchased from Fisher Scientific. K_3PO_4 tribasic was purchased from Sigma-Aldrich and used without further purification. The source of $Pd(OAc)_2$ was either Spectrum Chemicals or Johnson Matthey. Reagents were purchased from Sigma-Aldrich, Combi-Blocks, Alfa Aesar, or Acros Organics. *n*-Butyllithium was purchased from Sigma-Aldrich and was titrated with diphenylacetic acid prior to use. Flash chromatography was performed using Silicycle Silicaflash® P60 unbonded grade silica. ^1H and ^{13}C NMR were recorded at 25 °C on either a Varian Unity Inova 500 MHz or a Varian Unity Inova 600 MHz spectrometers in $CDCl_3$ with residual $CHCl_3$ (^1H = 7.26 ppm, ^{13}C = 77.16 ppm) as the internal standard. Chemical shifts are reported in parts per million (ppm). The data presented will be reported as follows; chemical shift, multiplicity (s = singlet, bs = broad singlet, d = doublet, dd = doublet of doublet, t = triplet, q = quartet, quin = quintet, m = multiplet), coupling constant (if applicable) and integration. HRMS data were recorded on a Waters Micromass LCT TOF ES+ Premier mass spectrometer using ESI ionization.

Synthesis and Analytical Data for N_2Phos

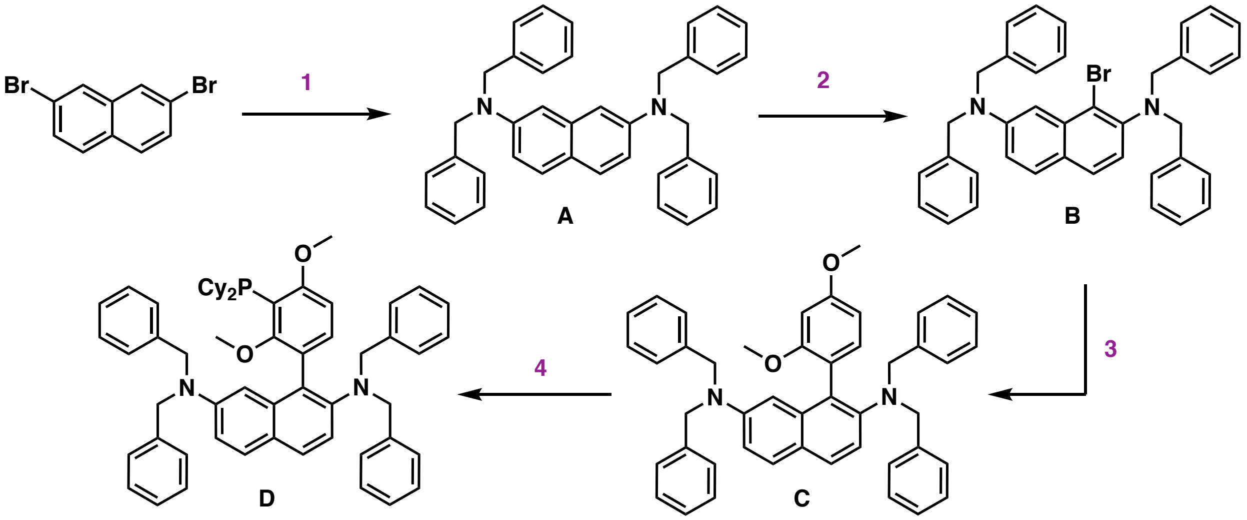

***N^2,N^2,N^7,N^7*-Tetrabenzylnaphthalene-2,7-diamine (A)**

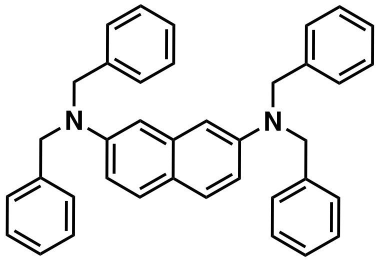

In solution. To an oven dried 3-neck round bottom flask equipped with a stir bar under argon were added 2,7-dibromonaphthalene (1 equiv), potassium *t*-butoxide (3 equiv), and Pd-PEPPSI-iPent (0.02 equiv). The reaction flask was evacuated and backfilled three times with argon. The flask was sealed with a rubber septum after which *N,N*-dibenzylamine (2.05 equiv) was added via syringe followed by anhydrous toluene (0.5 M). The reaction flask was sealed with a rubber septum and a water jacketed condenser was attached. The reaction was set to stir at 80 °C for 16 h. Upon completion, the reaction was cooled to rt. The solvent was removed under reduced pressure. The crude reaction mixture was added to a beaker and distilled H_2O was added. The suspension was set to stir for 30 min. The beakers' contents were then vacuum filtered, and the filtered material was allowed to dry. The dried mixture was transferred to a flask and sonicated with several portions of hexanes. The material was vacuum filtered and washed with hexanes and allowed to dry. The product (86% yield) was obtained as a white solid and used without any further purification.

Using Mechanochemistry. The amination was run in a 30 mL stainless steel Form Tech Scientific grinding jar (https://www.FormTechScientific.com). To the jar was added 1.72 g (6.00 mmol, 1 equiv) of 2,7-dibromonaphthalene, 2.02 g (18.0 mmol, 3 equiv) potassium *t*-butoxide, and 95.0 mg (0.120 mmol, 0.02 equiv) Pd-PEPPSI-IPent, and a 1/2" stainless steel ball. The jar was closed, leaving a slight gap where the two halves join together. The middle of the jar was wrapped with Parafilm and flushed with argon for several min. After this, 2.48 mL (12.3 mmol, 2.05 equiv) *N,N*-dibenzylamine was added by syringe through the Parafilm. The Parafilm was removed and the jar was fully sealed. The reaction vessel was placed in a modified SPEX 8000M Mixer/mill and run for 10 h at 40 °C. Afterwards, the dry, yellow-orange reaction mixture was scraped out of the vial onto a watch glass using a metal spatula. The dry reaction mixture was finely divided up using the metal spatula. This was then added to a beaker and suspended with stirring in distilled H_2O for 30 min to remove salt. The beaker's contents were then vacuum filtered and allowed to dry. The dried mixture was transferred to a recovery flask. This was sonicated in several portions of hexanes and transferred to a Buchner funnel, washed a final time with hexanes, and allowed to dry. The product (2.806 g, 90% yield) was obtained as a white solid. **^{1}H NMR** (600 MHz, $CDCl_3$) δ 7.49 (d, *J* = 9.0 Hz, 2H), 7.33-7.29 (m, 8H), 7.25-7.22 (m, 12H), 6.85 (dd, *J* = 9.0, 2.4 Hz, 2H), 6.74 (d, *J* = 2.4 Hz, 2H), 4.68 (s, 8H). **^{13}C NMR** (126 MHz, $CDCl_3$) δ 147.87, 138.82, 136.69, 128.73, 126.99, 126.96, 120.69, 112.38, 104.70, 53.96. **Chemical Formula:** $C_{38}H_{34}N_2$; EI-MS [M$^+$] calcd: 518.2722; found: 518.2720.

N^2,N^2,N^7,N^7-Tetrabenzyl-1-bromonaphthalene-2,7-diamine (B)

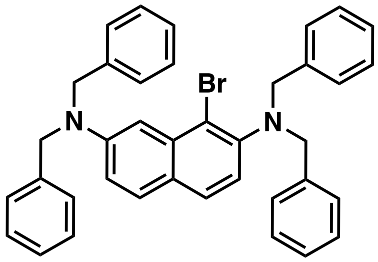

To an oven-dried round-bottom flask under a continuous flow of argon was added the substrate. The reaction vessel was evacuated and backfilled with argon two to three times. The substrate was dissolved in DMF (0.5 M) and cooled to 0 °C with an ice bath. A solution of *N*-bromosuccinimide (1 equiv) in DMF (0.5 M relative to substrate) was added dropwise slowly into the solution with the use of an addition funnel over a period of 10 min. The resulting mixture was removed from the ice bath and set to stir for an additional 3 h. Upon completion the reaction was diluted with ether and washed with

brine (4x the volume of organic solvent) to remove DMF. The resulting organic mixture was dried with anhydrous Na_2SO_4 and reduced under pressure. The compound was used without further purification. **^{1}H NMR** (500 MHz, $CDCl_3$) δ 7.42 (s, 1H) 7.43– 7.41 (d, J = 9.0 Hz, 1H), 7.36 (d, *J* = 8.6 Hz, 1H), 7.30 – 7.28 (m, 7H), 7.28 - 7.26 (m, 4H), 7.22 – 7.16 (m, 7H), 7.14 – 7.10 (m, 2H), 6.96 (dd, *J* = 9.0, 2.5 Hz, 1H), 6.82 (d, *J* = 8.6 Hz, 1H), 4.73 (s, 4H), 4.17 (s, 4H). **^{13}C NMR** (126 MHz, $CDCl_3$) δ 148.15, 147.62, 138.56, 138.44, 135.09, 129.32, 128.84, 128.83, 128.80, 128.28, 127.25, 127.21, 127.04, 127.02, 125.05, 119.09, 118.41, 115.21, 106.19, 57.03, 54.81. **Yield:** 96%, as a tan solid. **Chemical Formula**: $C_{38}H_{33}BrN_2$; EI-MS [M$^+$] calcd: 596.1827; found: 596.1824

***N^2*,*N^2*,*N^7*,*N^7*-Tetrabenzyl-1-(2,4-dimethoxyphenyl)naphthalene-2,7-diamine (C)**

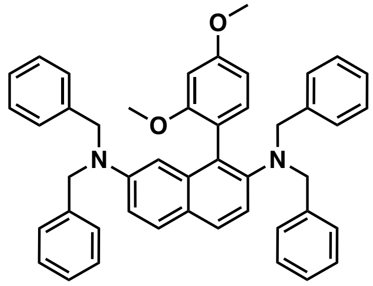

Aryl bromide (1 equiv), base (2 equiv), aryl boronic acid (1.5 equiv) and Pd(dppf)Cl_2 (2 mol %) were charged into a 3-neck round bottom flask under a continuous flow of argon. Upon addition of solids the flask was evacuated and then refilled with argon 3 times. Degassed toluene and water were then added to the reaction vessel in a 10:1 ratio of toluene to water. A reflux condenser was attached and the reaction was set to stir for 16 h at 80 ^{o}C. Upon completion, the reaction was diluted with EtOAc and the organic mixture was washed with water, and then brine. The resulting organic layer was dried over anhydrous Na_2SO_4 and the solvent was reduced under vacuum. The resulting crude mixture was purified via flash chromatography. **^{1}H NMR** (500 MHz, $CDCl_3$) δ 7.55 (dd, *J* = 12.1, 8.8 Hz, 2H), 7.26 (d, *J* = 4.5 Hz, 2H), 7.24 (d, *J* = 0.8 Hz, 2H), 7.22 – 7.13 (m, 12H), 7.06 – 7.02 (m, 5H), 6.98 (dd, *J* = 9.0, 2.6 Hz, 1H), 6.83 (d, *J* = 8.2 Hz, 1H), 6.55 (dd, *J* = 8.3, 2.4 Hz, 1H), 6.41 (d, *J* = 2.5 Hz, 1H), 6.38 (d, *J* = 2.4 Hz, 1H), 4.64 – 4.50 (m, 4H), 4.01 – 3.92 (m, 4H), 3.91 (s, 3H), 3.33 (s, 3H**).** **^{13}C NMR** (126 MHz, $CDCl_3$) δ 158.36, 139.27, 138.96, 135.72, 132.68, 129.05, 128.82, 128.64, 127.95, 127.34, 126.85, 126.80, 126.66, 118.53, 114.35, 105.45, 104.79, 98.57, 56.96, 55.62, 55.12, 55.10. **Yield:** 83%, as a yellow solid. **R_f:** 0.14 (10% EtOAc/hexanes) **Chemical Formula**: $C_{58}H_{63}N_2O_2P$ EI-MS [M$^+$] calcd: 859.4627; found: 850.4628.

N^2,N^2,N^7,N^7-Tetrabenzyl-1-(3-(dicyclohexylphosphaneyl)-2,4-dimethoxyphenyl)naphthalene-2,7-diamine L1 (D; N_2Phos)

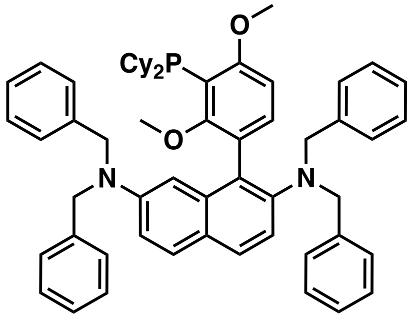

An oven dried round bottom flask was charged with substrate under a continuous flow of argon. The reaction vessel was evacuated and back-filled with argon three times. Anhydrous THF [0.2 M] was added to the reaction vessel and the reaction was cooled to 0 °C using an ice bath. *n*-Butyllithium ([2.3 M] in hexanes 1.2 equiv) was added to reaction dropwise via syringe over a period of 15 min. The resulting mixture was set to stir for an additional 30 min. Neat chlorodicyclohexylphosphine (1.05 equiv) was added dropwise via syringe over a period of 10 min. The reaction vessel was removed from the ice bath and set to stir for an additional 2 h. The solution was quenched with water and diluted with diethyl ether. The organic layer was washed with water and brine and dried over anhydrous Na_2SO_4. The resulting ether was concentrated under vacuum. The mixture was purified via flash chromatography utilizing <u>basic alumina</u> (5% EtOAc/hexanes) to afford a white solid; 75% yield; R_f = 0.28 (5% EtOAc/hexanes) on aluminum oxide plates. ^{1}H NMR (500 MHz, $CDCl_3$) δ 7.58 (d, *J* = 9.0 Hz, 1H), 7.54 (d, *J* = 8.7 Hz, 1H), 7.25 – 7.22 (m, 5H), 7.16 – 7.13 (m, 5H), 7.12 – 7.10 (m, 5H), 7.08 – 7.04 (m, 5H), 7.03 – 7.01 (m, 1H), 6.99 (d, *J* = 8.7 Hz, 1H), 6.73 (d, *J* = 8.4 Hz, 1H), 6.47 (d, *J* = 2.5 Hz, 1H), 6.36 (d, *J* = 8.5 Hz, 1H), 4.56 – 4.48 (m, 4H), 3.99 (d, *J* = 14.2 Hz, 2H), 3.92 (d, *J* = 14.3 Hz, 2H), 3.81 (s, 3H), 3.19 (s, 3H), 2.44 (m, 1H), 1.96 (d, *J* = 8.4 Hz, 2H), 1.81 – 1.72 (m, 2H), 1.63 (q, *J* = 10.7, Hz, 3H), 1.37 – 1.27 (m, 5H), 1.24 – 1.19 (m, 2H), 1.07 – 0.95 (m, 3H). **^{13}C NMR** (126 MHz, $CDCl_3$) δ 158.36, 139.27, 138.96, 135.72, 132.68, 129.05, 128.82, 128.64, 127.95, 127.34, 126.85, 126.80, 126.66, 118.53, 114.35, 105.45, 104.79, 98.57, 56.96, 55.62, 55.12, 55.10. **^{31}P NMR** (162 MHz, $CDCl_3$) δ -9.69. **Chemical Formula:** $C_{58}H_{63}N_2O_2P$. EI-MS [M^+] calcd: 851.4705; found: 851.4692.

Reaction Optimization

Palladium Source Screening

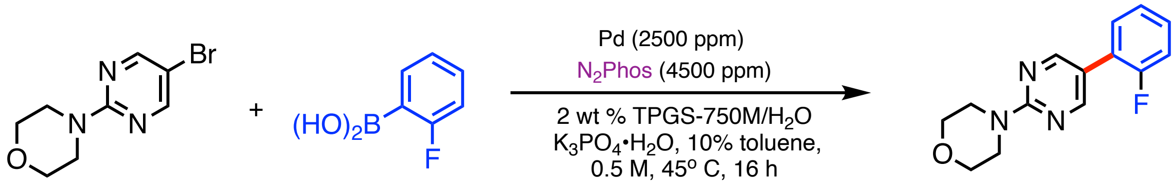

Entry	Pd Source	Conv (%)[1]
**1**	**$Pd(OAc)_2$**	**96**
2	$[Pd(cinnamyl)Cl_2]_2$	87
3	$[Pd(allyl)Cl_2]_2$	63
4	$Pd(PhCN)_2Cl_2$	71

[1]Conversion based on ^{1}H NMR.

Conditions: Ligand **L1**:Pd (1.8:1) pre-complexed in toluene. Aryl halide (0.5 mmol), arylboronic acid or equivalent (0.75 mmol), $K_3PO_4{\bullet}H_2O$ (0.75 mmol); 2 wt % TPGS-750-M/H_2O : toluene (9:1) at 45 ^oC.

Choice of Base

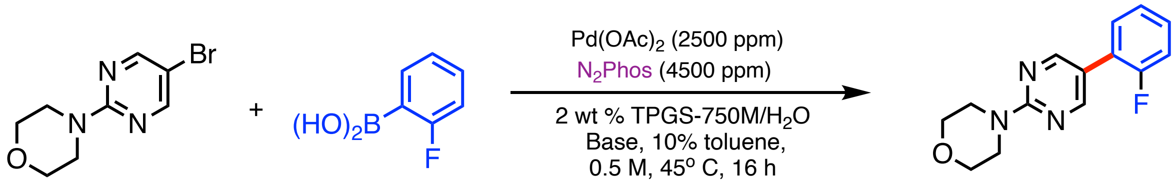

Entry	Base	Conv (%)[1]
1	Et_3N	88
**2**	**$K_3PO_4{\cdot}H_2O$**	**96**
3	K_2CO_3	79
4	DIPEA	89
5	NaO*t*-Bu	82

[1]Conversion based on ^{1}H NMR.

Conditions: Ligand **L1**:Pd (1.8:1) pre-complexed in toluene. Aryl halide (0.5 mmol), arylboronic acid or equivalent (0.75 mmol), base (0.75 mmol); 2 wt % TPGS-750-M/H_2O : toluene (9:1) at 45 ^oC.

Ligand Screening

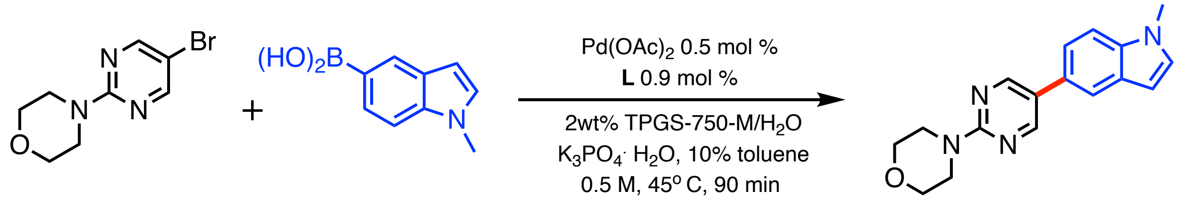

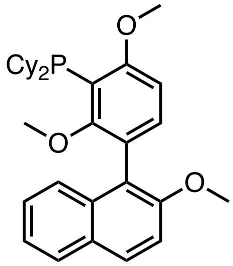

EvanPhos

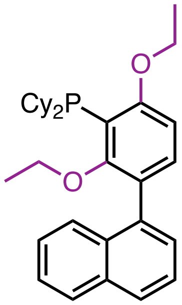

L2

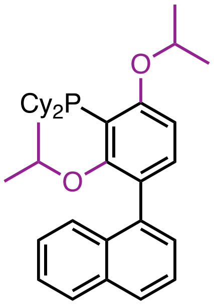

L3

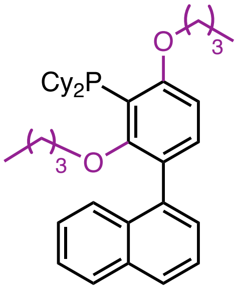

L4

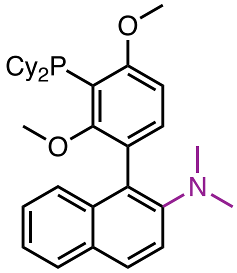

L5

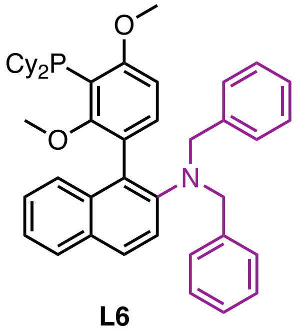

L6

Entry	Ligand	Conv (%)[1]
1	**EvanPhos**	79
2	**L2**	43
3	**L3**	45
4	**L4**	39
5	**L5**	81
6	**L6**	95

[1]Conversion based on ^1H NMR.

Conditions: Ligand **L1**:Pd (1.8:1) pre-complexed in toluene. Aryl halide (0.5 mmol), aryl boronic acid or equivalent (0.75 mmol), base (0.75 mmol); 2 wt % TPGS-750-M/H_2O : toluene (9:1) at 45 °C.

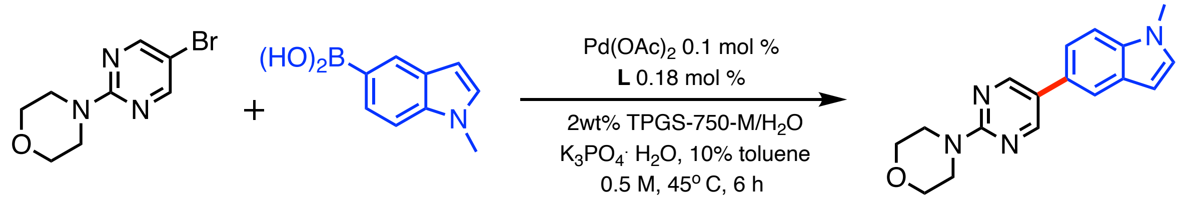

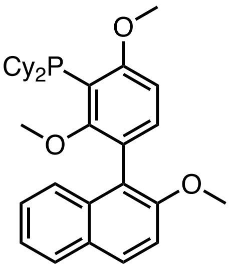

EvanPhos

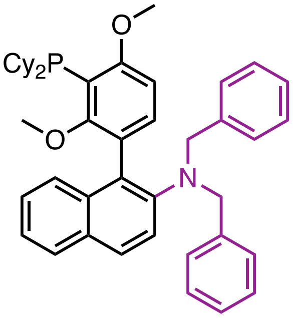

L6

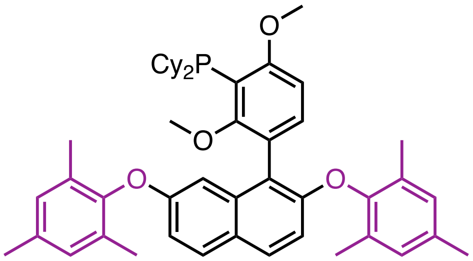

L7

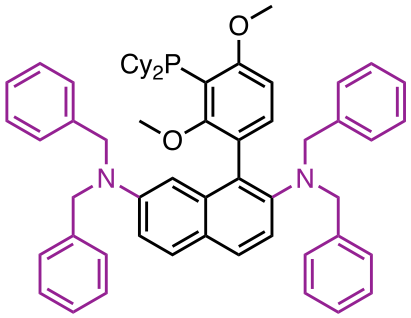

N_2Phos

Entry	Ligand	Conv (%)[1]
1	**EvanPhos**	62
2	**L6**	82
3	**L7**	59
4	**N_2Phos**	91

[1]Conversion based on ^{1}H NMR.

Conditions: Ligand **L1**:Pd (1.8:1) pre-complexed in toluene. Aryl halide (0.5 mmol), aryl boronic acid or equivalent (0.75 mmol), base (0.75 mmol); 2 wt % TPGS-750-M/H_2O : toluene (9:1) at 45 °C.

Pd:Ligand Optimization

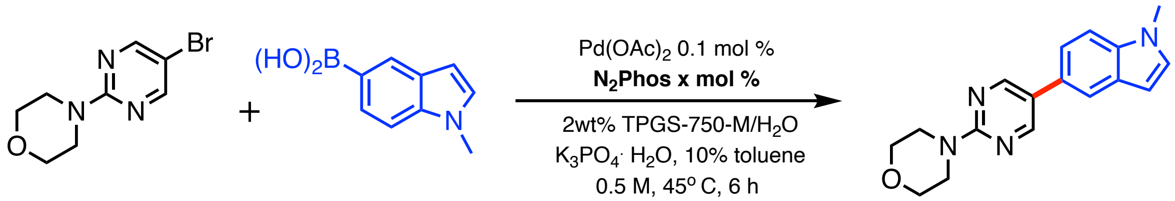

Entry	mol %	Conv (%)[1]
1	0.1	69
2	0.12	74
3	0.15	84
**4**	**0.18**	**91**
5	0.2	90

[1]Conversion based on ^{1}H NMR.

Conditions: Ligand **L1**:Pd (X:1) pre-complexed in toluene. Aryl halide (0.5 mmol), arylboronic acid or equivalent (0.75 mmol), base (0.75 mmol); 2 wt % TPGS-750-M/H_2O : toluene (9:1) at 45 ^oC.

Recycle Study

The initial reaction was set up according to the general procedure. After 4 h, the reaction media was extracted three times with MTBE and the combined organic layers were dried over anhydrous Na_2SO_4 and the solvent was removed under reduced pressure. The crude reaction mixture was purified via flash chromatography.

Recycling of the surfactant solution involved first sparging the reaction vial with argon followed by addition of aryl bromide (0.5 mmol), aryl boronic acid (0.625 mmol), the base (0.5 mmol) and fresh catalyst solution (0.1 mL). The reaction vial was then flushed with argon, capped, and sealed with Teflon tape.

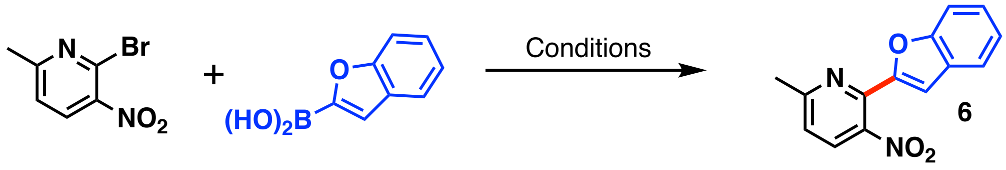

$$\text{E Factor} = \frac{\text{wt of solvent used (waste)}}{\text{wt of product isolated}}$$

E Factor:4.65

Initial Reaction: **93%**

1st Recycle: **92%**

2nd Recycle: **88%**

General procedure for catalyst solution preparation

Catalyst Preparation for SMC

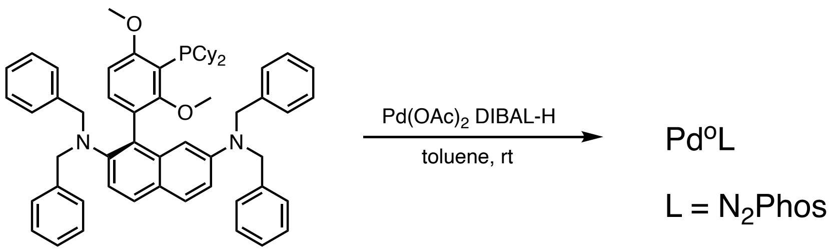

N_2Phos

To an oven dried 1-dram vile equipped with a stir bar was added $Pd(OAc)_2$ (2.25 mg, 0.01 mmol) and N_2Phos (14.8 mg, 0.018 mmol). The vial was capped with a 14/20 rubber septum sealed with Teflon tape. The vial was evacuated and backfilled with argon three times and left under a continuous flow of argon. Anhydrous toluene (1 mL) was added to the vile to achieve the desired Pd concentration (50 μL of stock solution equates to 1000 ppm loading for a 0.5 mmol reaction). The mixture was set to stir for 15 min. The resulting mixture was stirred for additional 5 min. At this point the catalyst is ready and may be added to the reaction mixture.

General procedure for Suzuki-Miyaura couplings (SMC)

General Procedure for SMC reactions in aqueous TPGS-750-M

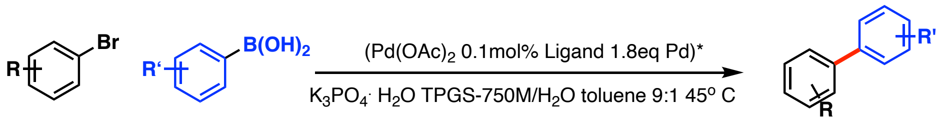

To an oven dried, 1-dram vial equipped with a magnetic stir bar was charged aryl bromide (0.5 mmol), organoboron (0.75 mmol), and potassium phosphate (0.75 mmol). The vial was fitted with a rubber septum and sealed with Teflon tape. The reaction vial was purged with argon with the use of a vent needle. At this point, a solution of 2 wt % TPGS-750-M in (0.9 mL) and toluene (50 μL) were added, followed by the catalyst solution via syringe. The vial was placed in an aluminum heating block over a stir plate with a stir rate set to >1000 rpm with a thermocouple probe in the aluminum block set to 48 ^{o}C (this gives a temperature of 45 ^{o}C in the reaction vial). The reactions were monitored by either GC/MS or TLC analysis. Upon completion of the reaction, the vial was cooled to rt. The mixture was extracted with EtOAc and the organic layer was washed with brine three times. The layers were then

separated and the organic layer was dried over anhydrous Na_2SO_4. The mixture was concentrated under vacuum and the crude mixture was purified via flash chromatography.

General Procedure for SMC Reactions in Organic Solvent

To an oven dried 1-dram vial equipped with a magnetic stir bar was charged aryl bromide (0.5 mmol), organoboron (0.75 mmol), and potassium phosphate (0.75 mmol). The vial was fitted with a rubber septum and sealed with Teflon tape. The reaction vial was purged with argon with the use of a vent needle. Degassed organic solvent (either EtOAc or toluene) (0.8 mL), DI water (0.1 mL) and the catalyst solution were added via syringe. The vial was placed in an aluminum heating block over a stir plate with a stir rate set to >1000 rpm with a thermocouple probe in the aluminum block set to 48 ^oC (this gives a temperature of 45 ^oC in the reaction vial). The reactions were monitored by either GC/MS or TLC analysis. Upon completion of the reaction, the vial was cooled to rt. The mixture was extracted with EtOAc and the organic layer was washed with brine three times. The layers were then separated and the organic layer was dried over anhydrous Na_2SO_4. The mixture was concentrated under vacuum and the crude mixture was purified via flash chromatography.

Analytical data for products from Suzuki-Miyaura couplings

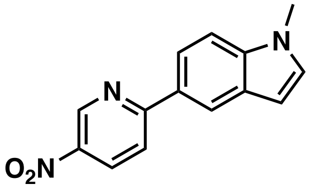

1-Methyl-5-(5-nitropyridin-2-yl)-1*H*-indole (1). Prepared from 5-bromo-5-nitropyridine and 1-methylindole-5-boronic acid according to the general procedure**.** ^{1}**H NMR** (500 MHz, $CDCl_3$) δ 9.48 (dd, *J* = 2.7, 0.7 Hz, 1H), 8.48 (dd, *J* = 8.8, 2.7 Hz, 1H), 8.41 (dd, *J* = 1.8, 0.6 Hz, 1H), 8.01 (dd, *J* = 8.7, 1.8 Hz, 1H), 7.94 (dd, *J* = 8.8, 0.8 Hz, 1H), 7.44 (dt, *J* = 8.7, 0.8 Hz, 1H), 7.13 (d, *J* = 3.1 Hz, 1H), 6.61 (dd, *J* = 3.2, 0.9 Hz, 1H), 3.85 (s, 3H**).** ^{13}**C NMR** (126 MHz, $CDCl_3$) δ 164.02, 145.40, 131.82, 130.49, 128.76, 121.53, 121.45, 119.54, 110.02, 102.66, 77.36, 33.22. **Yield**: 89%, 12 h; as a yellow solid. **R$_f$**: 0.31 (25% EtOAc/hexanes).

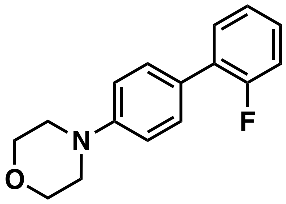

4-(2'-Fluoro-[1,1'-biphenyl]-4-yl)morpholine (2). Prepared from 4-bromophenylmorpholine and 2-fluorophenylboronic acid according to the general procedure. ^{1}**H NMR** (400 MHz, $CDCl_3$) δ 7.53 – 7.46 (m, 2H), 7.42 (td, *J* = 7.8, 1.9 Hz, 1H), 7.24 (dd, *J* = 5.0, 2.0 Hz, 1H), 7.18 (td, *J* = 7.5, 1.4 Hz, 1H), 7.13 (ddd, *J* = 10.9, 8.0, 1.3 Hz, 1H), 7.02 – 6.95 (m, 2H), 3.91 – 3.86 (m, 4H), 3.24 – 3.20 (m, 4H). ^{13}**C NMR** (101 MHz, $CDCl_3$) δ 150.76, 130.48, 129.95, 129.92, 128.37, 128.29, 124.43, 116.29, 116.06, 115.43, 67.03, 49.13. ^{19}**F NMR** (376 MHz, $CDCl_3$) δ -118.30 (dddt, *J* = 11.2, 8.3, 4.9, 1.7 Hz). **Yield**: 93%, as a white solid. **R$_f$**: 0.24 (15% EtOAc/hexanes).

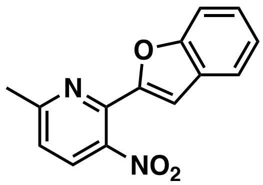

2-(Benzofuran-2-yl)-6-methyl-3-nitropyridine (3). Synthesized with 2-bromo-3-nitro-6-methyl-pyridine and benzofuran-2-phenyl boronic acid according to the general procedure. **^{1}H NMR** (500 MHz, $CDCl_3$) δ 7.94 (d, *J* = 8.3 Hz, 1H), 7.68 (ddd, *J* = 7.8, 1.3, 0.7 Hz, 1H), 7.54 – 7.51 (m, 2H), 7.38 (ddd, *J* = 8.3, 7.2, 1.3 Hz, 1H), 7.29 – 7.27 (m, 1H), 7.25 (d, *J* = 8.3 Hz, 1H), 2.71 (s, 3H). **^{13}C NMR** (126 MHz, $CDCl_3$) δ 162.12, 155.82, 151.26, 141.09, 134.18, 132.24, 128.03, 126.27, 123.60, 122.71, 122.69, 122.15, 112.01, 109.33, 24.83, 24.50. **Yield:** 94%, as a yellow solid. **R_f:** 0.35 (25% EtOAc/hexanes).

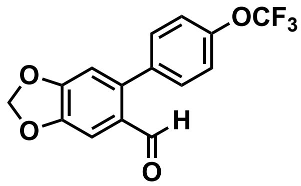

6-(4-(Trifluoromethoxy)phenyl)benzo[*d*][1,3]dioxole-5-carbaldehyde (4). Synthesized with 5-Bromopiperonal and 4-trifluoromethoxypehnyl boronic acid according to the general procedure. **^{1}H NMR** (500 MHz, $CDCl_3$) δ 9.73 (s, 1H), 7.47 (s, 1H), 7.38 – 7.36 (m, 2H), 7.31 – 7.29 (m, 2H), 6.82 (s, 1H), 6.10 (s, 2H). **^{13}C NMR** (126 MHz, $CDCl_3$) δ 190.15, 152.35, 149.35, 148.27, 142.04, 136.37, 131.62, 129.04, 121.62, 120.96, 120.95, 110.33, 106.64, 102.39, **^{19}F NMR** (376 MHz, $CDCl_3$) δ -57.85; **Yield**: 92%, as a white solid. **R_f:** 0.30 (15% EtOAc/hexanes).

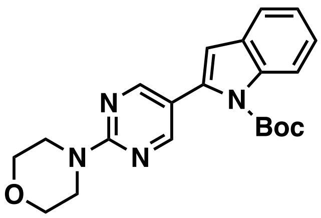

***t*-Butyl 2-(2-morpholinopyrimidin-5-yl)-1*H*-indole-1-carboxylate (5).** Synthesized with 4-(5-bromo-pyrimidin-2-yl)morpholine and (1-(*t*-butoxycarbonyl)-1H-indol-2-yl) boronic acid according to the general procedure. ^{1}**H NMR** (400 MHz, $CDCl_3$) δ 8.39 (s, 2H), 8.18 (d, *J* = 8.3 Hz, 1H), 7.55 (d, *J* = 7.6 Hz, 1H), 7.38 – 7.21 (m, 2H), 6.55 (s, 1H), 3.90 – 3.82 (m, 4H), 3.83 – 3.75 (m, 4H), 1.50 (s, 9H). ^{13}**C NMR** (101 MHz, $CDCl_3$) δ 160.84, 157.27, 150.23, 137.27, 135.08, 129.25, 124.67, 123.27, 120.56,

117.87, 115.85, 110.63, 84.28, 66.96, 44.49, 28.11. **Yield:** 78%, 24 h as a white solid; **R$_f$:** 0.20 (15% EtOAc/hexanes).

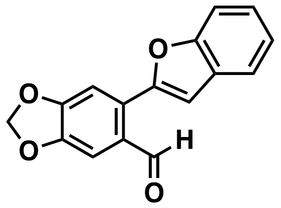

6-(Benzofuran-2-yl)benzo[*d*][1,3]dioxole-5-carbaldehyde (6). Synthesized from 5-bromopiperanal and benzofuran-2-phenylboronic acid according to the general procedure. **^{1}H NMR** (500 MHz, $CDCl_3$) δ 10.27 (s, 1H), 7.64 (ddd, *J* = 7.7, 1.4, 0.7 Hz, 1H), 7.54 (dq, *J* = 8.3, 0.9 Hz, 1H), 7.50 (s, 1H), 7.36 (ddd, *J* = 8.4, 7.3, 1.4 Hz, 1H), 7.29 (td, *J* = 7.5, 1.0 Hz, 1H), 7.23 (s, 1H), 6.87 (d, *J* = 1.0 Hz, 1H), 6.12 (s, 2H). **^{13}C NMR** (126 MHz, $CDCl_3$) δ 190.21, 155.47, 152.43, 152.36, 149.01, 130.65, 129.86, 128.69, 125.31, 123.53, 121.42, 111.54, 108.89, 108.13, 107.05, 102.55. **Yield**: 91%, 8 h, as a white solid. **R$_f$**: 0.31 (15% EtOAc/hexanes).

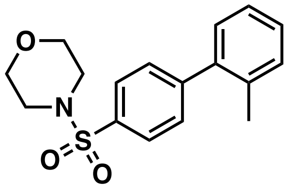

4-((2'-Methyl-[1,1'-biphenyl]-4-yl)sulfonyl)morpholine (7). Synthesized with 4-((4-bromophenyl)-sulfonyl)morpholine and methyl phenyboronic acid according to the general procedure**.** **^{1}H NMR** (500 MHz, $CDCl_3$) δ 7.82 – 7.79 (d, 2H), 7.52 – 7.49 (d, 2H), 7.34 – 7.27 (m, 3H), 7.22 (dd, *J* = 7.2, 1.3 Hz, 1H), 3.80 – 3.76 (m, 4H), 3.10 – 3.04 (m, 4H), 2.27 (s, 3H). **^{13}C NMR** (126 MHz, $CDCl_3$) δ 147.22, 140.08, 135.28, 130.79, 130.07, 129.64, 128.39, 127.88, 126.21, 105.14, 66.30, 46.20, 20.51. **Yield**: 81%, 16 h, as a white solid. **R$_f$**: 0.55 (40% EtOAc/hexanes). **Chemical Formula:** $C_{17}H_{19}NO_3S$ EI-MS $[M]^+$ calcd: 317.1086; found: 372.1253 $[M+Na+MeOH]^+$.

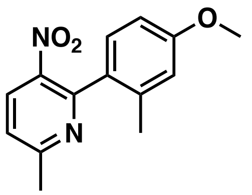

2-(4-Methoxy-2-methylphenyl)-6-methyl-3-nitropyridine (8). Synthesized with 2-Bromo-6-methyl-3-nitropyridine and 2-methyl-4-methoxyphenyl boronic acid according to the general procedure. **^{1}H NMR**

(500 MHz, $CDCl_3$) δ 8.17 (d, *J* = 8.3 Hz, 1H), 7.28 (dd, *J* = 8.3, 0.5 Hz, 1H), 7.12 (d, *J* = 8.3 Hz, 1H), 6.82 (dt, *J* = 2.8, 0.6 Hz, 1H), 6.79 (ddd, *J* = 8.3, 2.6, 0.6 Hz, 1H), 3.83 (s, 3H), 2.69 (s, 3H), 2.16 (s, 3H). **^{13}C NMR** (126 MHz, $CDCl_3$) δ 162.72, 160.17, 153.79, 137.51, 132.52, 129.69, 129.49, 122.13, 116.14, 111.47, 55.39, 24.95, 19.90. **Yield**: 91%, 8 h, as a yellow solid **R_f:** 0.20 (25% EtOAc/hexanes).

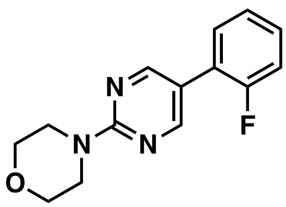

4-(5-(2-Fluorophenyl)pyrimidin-2-yl)morpholine (9). Synthesized with 4-(5-bromopyrimidin-2-yl)morpholine and 2-fluorophenyl boronic acid according to the general procedure. **^{1}H NMR** (500 MHz, $CDCl_3$) δ 8.55 (d, *J* = 2.9 Hz, 2H), 8.54 (d, *J* = 1.6 Hz, 2H), 7.39 – 7.35 (m, 1H), 7.34 – 7.30 (m, 1H), 7.23 – 7.20 (m, 1H), 7.16 (m, 1H), 3.87 (dd, *J* = 5.7, 3.9 Hz, 4H), 3.79 (dd, *J* = 5.6, 3.9 Hz, 4H). **^{13}C NMR** (126 MHz, $CDCl_3$) δ 157.48, 157.45, 129.47, 129.44, 129.32, 129.25, 124.88, 124.85, 116.49, 116.31, 105.14, 66.98, 44.46. **^{19}F NMR** (376 MHz, $CDCl_3$) δ -117.81 (dt, *J* = 7.7, 2.8 Hz). **Yield**: 83%, 22 h, as a white solid. **R_f**: 0.50 (25% EtOAc/hexanes). **Chemical Formula:** $C_{14}H_{14}FN_3O$ EI-MS $[M]^+$ calcd: 259.1121; found: 260.1196.

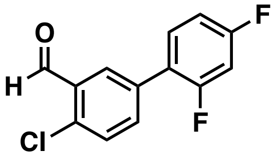

4-Chloro-2',4'-difluoro-[1,1'-biphenyl]-3-carbaldehyde (10) Synthesized with 3-bromo-6-chlorobenzalsehyde and 2,4-difluorophenyl boronic acid according to the general procedure. The pinacol ester of the boronic acid was also employed. **^{1}H NMR** (500 MHz, $CDCl_3$) δ 9.84 (d, *J* = 3.0 Hz, 1H), 8.00 (d, *J* = 2.3 Hz, 1H), 7.63 (dd, *J* = 8.2, 2.3 Hz, 1H), 7.34 (d, *J* = 8.2 Hz, 1H), 7.32 – 7.27 (m, 1H), 7.03 (m, 1H), 6.98 – 6.94 (m, 1H). ^{13}C NMR (126 MHz, $CDCl_3$) δ 190.02, 136.18, 135.47, 133.89, 132.96, 132.74, 132.71, 132.67, 132.63, 128.13, 112.24, 112.21, 112.07, 112.04, 104.74, 104.54, 104.33. **^{19}F NMR** (376 MHz, $CDCl_3$) δ -108.22 (qd, *J* = 8.3, 6.2 Hz), -110.85 (dd, *J* = 9.0, 2.9 Hz). **Yield:** 92% with the boronic acid. 94% as the pinacol ester as a white solid. **R_f**: 0.54 (15% EtOAc/hexanes).

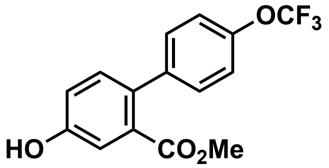

Methyl 4-hydroxy-4'-(trifluoromethoxy)-[1,1'-biphenyl]-2-carboxylate (11). Synthesized with methyl 5-hydroxy-2-iodobenzoate and 4-trifluoromethoxyphenylboronic acid according to the general procedure**.** ^{1}**H NMR** (500 MHz, MeOH-*d*$_4$) δ 7.71 – 7.68 (m, 2H), 7.58 – 7.55 (m, 2H), 7.40 – 7.37 (m, 1H), 7.31 (dq, *J* = 7.8, 1.0 Hz, 2H), 3.90 (s, 3H). ^{13}**C NMR** (126 MHz, MeOH-*d*$_4$) δ 166.90, 154.27, 136.93, 130.64, 130.33, 130.25, 120.55, 120.13, 116.34, 106.82, 51.20**.** ^{19}**F NMR** (376 MHz, $CDCl_3$) δ -57.79. **Yield:** 83%, as a pale yellow solid. **R$_f$:** 0.45 (40% EtOAc/hexanes).

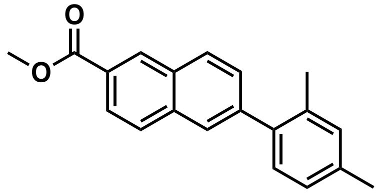

Methyl 6-(2,4-dimethylphenyl)-2-naphthoate (12). Synthesized with methyl 6-bromo-2-naphthoate and 2,4-methylphenylbornoc acid according to the general procedure. ^{1}**H NMR** (500 MHz, $CDCl_3$) δ 8.66 – 8.62 (m, 1H), 8.08 (dd, *J* = 8.6, 1.7 Hz, 1H), 7.99 – 7.96 (m, 1H), 7.89 (dt, *J* = 8.6, 0.7 Hz, 1H), 7.79 (t, *J* = 1.1 Hz, 1H), 7.53 (dd, *J* = 8.4, 1.7 Hz, 1H), 7.22 (d, *J* = 7.6 Hz, 1H), 7.16 – 7.13 (m, 1H), 7.11 (m, 1H), 4.00 (s, 3H), 2.40 (s, 3H), 2.29 (s, 3H). ^{13}**C NMR** (126 MHz, $CDCl_3$) δ 167.42, 142.21, 138.60, 137.56, 135.60, 135.39, 131.39, 130.99, 129.98, 129.04, 128.89, 128.32, 127.80, 127.39, 126.79, 125.66, 77.41, 77.16, 76.91, 52.37, 21.24, 20.57. **Yield:** 96%, 1.5 h as a white solid. **R$_f$**: 0.50 (25% EtOAc/hexanes).

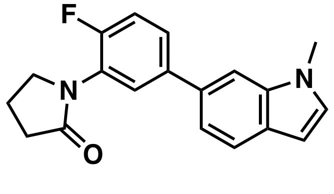

1-(2-Fluoro-5-(1-methyl-1*H*-indol-6-yl)phenyl)pyrrolidin-2-one (13). Synthesized from 1-(5-bromo-2-fluorophenyl)pyrrolidine-2-one and 1-methylindole-5-boronic acid according to the general

procedure. **^{1}H NMR** (500 MHz, $CDCl_3$) δ 7.79 – 7.77 (m, 1H), 7.65 (dd, *J* = 7.3, 2.4 Hz, 1H), 7.50 (ddd, *J* = 8.4, 4.6, 2.3 Hz, 1H), 7.41 (dd, *J* = 8.4, 1.8 Hz, 1H), 7.37 – 7.35 (m, 1H), 7.20 (dd, *J* = 10.5, 8.6 Hz, 1H), 7.08 (d, *J* = 3.1 Hz, 1H), 6.52 (d, *J* = 2.3 Hz, 1H), 3.88 (t, *J* = 7.0 Hz, 2H), 3.82 (s, 3H), 2.61 (t, *J* = 8.0 Hz, 2H), 2.24 (p, *J* = 7.5 Hz, 2H). **^{13}C NMR** (126 MHz, $CDCl_3$) δ 174.91, 139.53, 132.69, 131.36, 129.76, 129.06, 127.20, 127.14, 126.73, 126.71, 121.36, 119.56, 116.90, 109.61, 101.48, 50.30, 33.09, 31.32, 19.22. **^{19}F NMR** (376 MHz, $CDCl_3$) δ -124.22 (ddd, *J* = 11.1, 7.3, 4.7 Hz).
Yield: 80% 36 h as a tan solid. **R_f:** 0.24 (40% EtOAc/hexanes).

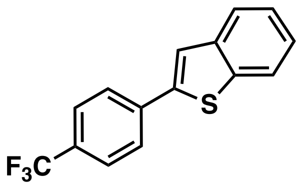

2-(4-(Trifluoromethyl)phenyl)benzo[*b*]thiophene (14) Synthesized with benzothiophene-2-bornic acid and 4-trifluoromethylphenyl boronic acid according to the general procedure. **^{1}H NMR** (500 MHz, $CDCl_3$) δ 7.87 – 7.84 (m, 1H), 7.82 (m, 3H), 7.68 (d, *J* = 8.2 Hz, 2H), 7.64 (d, *J* = 0.7 Hz, 1H), 7.37 (m, 2H). **^{13}C NMR** (126 MHz, $CDCl_3$) δ 140.57, 126.75, 126.11, 126.08, 125.14, 124.96, 124.11, 123.80, 122.52, 121.41, 121.19. **^{19}F NMR** (376 MHz, $CDCl_3$) δ -62.64. **Yield:** 87%, 16 h, as a white solid. **R_f:** 0.60 (25% EtOAc/hexanes).

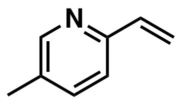

5-Methyl-2-vinylpyridine (15). Prepared from 2-boromo-5-methylpyridine and potassium vinyltrifluoroborate according to the general procedure. **^{1}H NMR** (400 MHz, $CDCl_3$) δ 8.37 (s, 1H), 7.41 (d, *J* = 9.0, 1H), 7.24 (d, *J* = 9.0 Hz, 1H), 6.78 (dd, *J* = 11.0, 17.5, 1H), 6.12 (dd, *J* = 17.5, 1H), 5.41 (d, *J* = 11.0 Hz, 1H) 2.31 (s, 3H). **^{13}C NMR** (101 MHz, $CDCl_3$) δ 153.51, 149.69, 138.03, 137.03, 132.38, 121.40, 117.15, 20.22. **Yield**: 89%, as a colorless oil. **R_f**: 0.20 (10% EtOAc/hexanes).

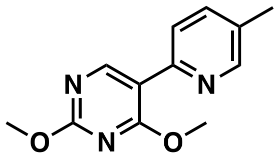

2,4-Dimethoxy-5-(5-methylpyridin-2-yl)pyrimidine (16). Prepared from 2-bromo-5-methylpyridine and (2,4-dimethoxypyrimidin-5-yl)boronic acid MIDA ester according to the general procedure. **^{1}H NMR** (400 MHz, $CDCl_3$) δ 8.83 (s, 1H), 8.52 – 8.48 (s, 1H), 7.71 (d, J = 8.1 Hz, 1H), 7.53 (d, J = 8.1, Hz, 1H), 4.07 (s, 3H), 4.05 (s, 3H), 2.37 (s, 3H). **^{13}C NMR** (101 MHz, $CDCl_3$) δ 164.96, 159.54, 150.18, 149.16, 136.94, 131.93, 123.56, 114.90, 110.16, 55.13, 54.32, 29.86. **Yield**: 87%, as a white solid. **R_f**: 0.30 (40% EtOAc/hexanes).

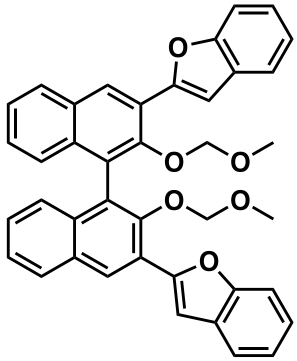

2,2'-(2,2'-bis(Methoxymethoxy)-[1,1'-binaphthalene]-3,3'-diyl)bis(benzofuran) (17) Synthesized with 3,3'-diiodo-2,2'-bis(methoxymethoxy)-1,1'-binaphthalene and benzofuran-2-boronic acid according to the general procedure. **^{1}H NMR** (400 MHz, $CDCl_3$) δ 8.68 (s, 2H), 8.01 (d, J = 8.2 Hz, 2H), 7.69 – 7.55 (m, 6H), 7.47 (t, J = 7.1 Hz, 2H), 7.29 (m, 8H), 4.81-4.53 (m, 4H), 2.56 (s, 6H); **^{13}C NMR** (101 MHz, $CDCl_3$) δ 154.6,152.3, 150.0, 134.0, 130.9, 129.2, 128.7, 128.1, 127.4, 126.7, 126.5, 125.9, 124.9, 124.2, 123.1, 121.6,111.1, 107.1, 98.7, 56.7. **Yield**: 79%, 24 h as a white solid. **R_f:** 0.40 (25% EtOAc/hexanes).

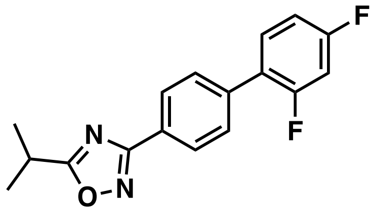

3-(2',4'-Difluoro-[1,1'-biphenyl]-4-yl)-5-isopropyl-1,2,4-oxadiazole (18). Prepared from 3-(4-bromophenyl)-5-isopropyl-1,2,4-oxadiazole and 2,4-difluorophenylbornic acid according to the general procedure**.** **^{1}H NMR** (400 MHz, $CDCl_3$) δ 8.21 – 8.13 (d, *J* = 8.4 2H), 7.64 (d, *J* = 1.7 Hz, 2H), 7.48 (td, *J* = 8.7, 6.4 Hz, 1H), 7.04 – 6.93 (m, 2H), 3.33 (hept, *J* = 7.0 Hz, 1H), 1.50 (d, *J* = 7.0 Hz, 6H). **^{13}C NMR** (101 MHz, $CDCl_3$) δ 184.17, 168.00, 131.60, 129.45, 129.42, 127.73, 126.52, 112.03, 111.81, 104.71, 27.73, 20.39. **^{19}F NMR** (376 MHz, $CDCl_3$) δ -110.51 (p, *J* = 7.8 Hz), -113.12 (q, *J* = 9.1 Hz). **Yield**: 81%, as a white solid. **R_f:** 0.25 (5% EtOAc/hexanes).

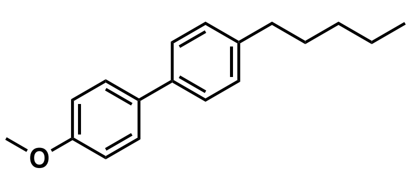

4-Methoxy-4'-pentyl-1,1'-biphenyl (21). Synthesized from 4-chloroanisole and 4-pentylphenylboronic acid according to the general procedure ($Pd(OAc)_2$ 0.25 mol%). **^{1}H NMR** (600 MHz, $CDCl_3$) δ 7.52 (d, *J* = 8.7 Hz, 2H), 7.47 (d, *J* = 8.2 Hz, 2H), 7.23 (d, *J* = 8.3 Hz, 2H), 6.97 (d, *J* = 8.7 Hz, 2H), 3.85 (s, 3H), 2.65 – 2.62 (m, 2H), 1.67 – 1.63 (m, 2H), 1.38 – 1.34 (m, 4H), 0.93 – 0.90 (m, 3H). **^{13}C NMR** (151 MHz, $CDCl_3$) δ 141.60, 138.29, 133.92, 128.92, 128.11, 126.70, 114.28, 108.93, 77.37, 77.16, 76.95, 55.48, 35.70, 31.72, 31.37, 22.73, 14.20. **Yield**: 83%, as a white solid. **R_f**: 0.30 (5% EtOAc/hexanes).

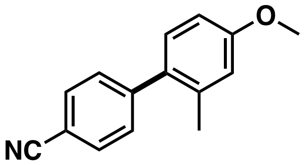

4'-Methoxy-2'-methyl-[1,1'-biphenyl]-4-carbonitrile (22). Synthesied from 4-chlorobenzonitrole and 2-methyl-4-methoxyphenyl boronic acid according to the general procedure ($Pd(OAc)_2$ 0.25 mol%). **^{1}H NMR** (600 MHz, $CDCl_3$) δ 7.68 (d, *J* = 8.6 Hz, 2H), 7.41 (d, *J* = 8.5 Hz, 2H), 7.13 (d, *J* = 8.3 Hz, 1H), 6.84 – 6.81 (m, 2H), 3.84 (s, 3H), 2.26 (s, 3H). **^{13}C NMR** (151 MHz, $CDCl_3$) δ 159.63, 146.69, 136.70, 132.74, 132.04, 130.79, 130.29, 119.19, 116.20, 111.63, 110.42, 77.37, 77.16, 76.95, 55.44, 20.79. **Yield**: 94%, as a yellow solid. **R_f:** 0.25 (10% EtOAc/hexanes).

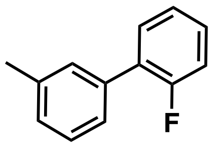

2-Fluoro-3'-methyl-1,1'-biphenyl (23). Synthesized from 3-chlorotoluene and 2-fluorophenyl boronic acid according to the general procedure ($Pd(OAc)_2$ 0.25 mol%). **^{1}H NMR** (500 MHz, $CDCl_3$) δ 7.44 (m, J = 7.8, 1.8 Hz, 1H), 7.39 – 7.34 (m, 3H), 7.33 – 7.29 (m, 1H), 7.21 (m, J = 7.5, 1.4 Hz, 2H), 7.15 (m, J = 11.0, 8.2, 1.5 Hz, 1H), 2.43 (s, 3H). **^{13}C NMR** (126 MHz, $CDCl_3$) δ 138.16, 135.90, 130.96, 129.91, 129.00, 128.46, 126.28, 124.42, 116.27, 116.09, 21.65. **^{19}F NMR** (376 MHz, $CDCl_3$) δ -117.96 (m). **Yield**: 87%, as a colorless oil. **R_f:** 0.35 (5% Et_2O/hexanes).

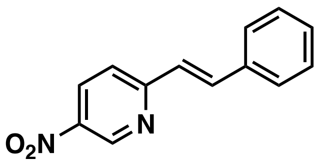

(*E*)-5-Nitro-2-styrylpyridine (24). Synthesized with 2-chloro-5-nitropyridine and (*E*)-phenethylboronic acid according to the general procedure. **^{1}H NMR** (500 MHz, $CDCl_3$) δ 9.43 – 9.40 (m, 1H), 8.44 (dd, J = 8.6, 2.7 Hz, 1H), 7.87 (d, J = 16.0 Hz, 1H), 7.64 – 7.61 (m, 2H), 7.50 (dd, J = 8.7, 0.7 Hz, 1H), 7.44 – 7.37 (m, 3H), 7.24 (d, J = 15.9 Hz, 1H). **^{13}C NMR** (126 MHz, $CDCl_3$) δ 161.09, 145.59, 138.26, 131.92, 129.78, 129.10, 127.89, 125.92, 121.78, 105.15. **Yield**: 93%, 16 h, as a yellow solid. **R_f**: 0.40 (25% EtOAc/hexanes).

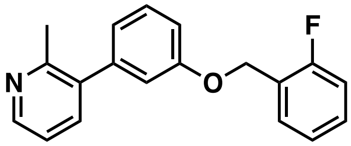

3-(3-((2-Fluorobenzyl)oxy)phenyl)-2-methylpyridine (25). Synthesized from 3-chloro-2-methyl-pyridine and 3-((2-fluorobenzyl)oxy)phenyl boronic acid according to the general procedure ($Pd(OAc)_2$ 0.25 mol%). **^{1}H NMR** (400 MHz, $CDCl_3$) δ 8.50 (dd, J = 5.0, 1.7 Hz, 1H), 7.51 (dd, J = 7.7, 1.8 Hz, 2H), 7.39 – 7.29 (m, 2H), 7.20 – 7.15 (m, 2H), 7.09 (ddd, J = 9.6, 8.2, 1.1 Hz, 1H), 7.04 – 7.00 (m, 1H), 6.95 – 6.91 (m, 2H), 5.17 (s, 2H), 2.49 (s, 3H). ^{13}C **NMR** (101 MHz, $CDCl_3$) δ 161.81, 158.58, 155.96, 148.16, 141.55, 137.20, 136.87, 129.97, 124.45, 124.41, 122.06, 121.09, 115.83, 113.94, 110.15, 63.94, 23.51. **^{19}F NMR** (376 MHz, $CDCl_3$) δ -118.53 (ddd, J = 10.3, 7.4, 5.5 Hz). **Yield**: 91%, 16 h, as a yellow oil. **R_f**: 0.21 (25% EtOAc/hexanes).

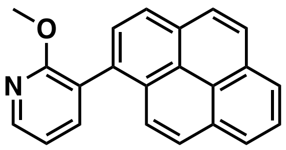

2-Methoxy-3-(pyren-1-yl)pyridine (26). Synthesized from and pyrene boronic acid according to the general procedure ($Pd(OAc)_2$ 0.25 mol%). **^{1}H NMR** (600 MHz, $CDCl_3$) δ 8.34 (dd, *J* = 5.1, 1.9 Hz, 1H), 8.22 (d, *J* = 7.8 Hz, 1H), 8.19 (dd, *J* = 7.6, 1.1 Hz, 1H), 8.16 (dd, *J* = 7.6, 1.0 Hz, 1H), 8.09 (s, 2H), 8.02 – 7.98 (m, 2H), 7.93 (d, *J* = 7.8 Hz, 1H), 7.80 (d, *J* = 9.2 Hz, 1H), 7.71 (dd, *J* = 7.1, 1.9 Hz, 1H), 7.10 (dd, *J* = 7.1, 5.1 Hz, 1H), 3.89 (s, 3H). **^{13}C NMR** (151 MHz, $CDCl_3$) δ 161.91, 146.59, 140.74, 132.31, 131.51, 131.17, 131.07, 129.24, 127.99, 127.72, 127.59, 127.54, 126.13, 125.36, 125.33, 125.18, 124.93, 124.70, 124.13, 116.94, 53.74. **Yield:** 91%, as a white solid. **R_f**: 0.25 (10% EtOAc/hexanes) **Chemical Formula**: $C_{22}H_{15}NO$ EI-MS $[M]^+$ calcd: 310.1232; found: 310.1227.

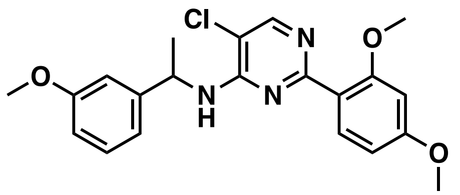

5-Chloro-2-(2,4-dimethoxyphenyl)-*N*-(1-(3-methoxyphenyl)ethyl)pyrimidin-4-amine (27). Synthesized from 2,5-dichloro-*N*-(1-(3-methoxyphenyl)ethyl)pyrimidin-4-amine and 2,4-dimethoxyphenylboronic acid according to the general procedure ($Pd(OAc)_2$ 0.25 mol %). **^{1}H NMR** (400 MHz, $CDCl_3$) δ 8.26 (s, 1H), 7.67 – 7.62 (m, 1H), 7.36 – 7.30 (m, 2H), 6.92 – 6.87 (m, 2H), 6.54 (m, 2H), 5.43 (dt, *J* = 13.6, 7.3 Hz, 2H), 3.85 (s, 3H), 3.84 (s, 3H), 3.81 (s, 3H), 1.62 (d, *J* = 6.5 Hz, 3H). **^{13}C NMR** (101 MHz, $CDCl_3$) δ 163.00, 162.09, 159.29, 158.96, 156.66, 152.59, 135.82, 133.12, 127.61, 121.32, 114.11, 111.79, 104.72, 99.40, 77.36, 56.14, 55.56, 55.45, 49.59, 22.09. **Yield**: 76%, as a tan solid. **R_f**: 0.25 (30% Et_2O in hexanes) **Chemical Formula:** $C_{21}H_{22}ClN_3O_3$ EI-MS $[M]^+$ calcd: 399.1350; found: 401.1360 [M+2H].

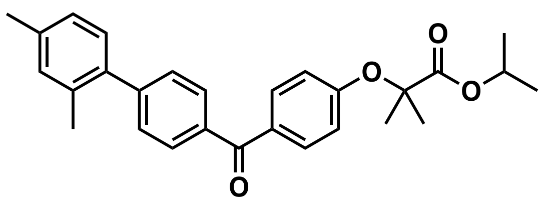

Isopropyl-2-(4-(2',4'-dimethyl-[1,1'-biphenyl]-4-carbonyl)phenoxy)-2-methylpropanoate (28). Synthesized from isopropyl 2-(4-(4-chlorobenzoyl)phenoxy)-2-methylpropanoate and 2,4-dimethyl-phenylboronic acid according to the general procedure ($Pd(OAc)_2$ 0.25 mol%). **^{1}H NMR** (500 MHz, $CDCl_3$) δ 7.83 – 7.78 (m, 4H), 7.42 – 7.39 (m, 2H), 7.19 (d, *J* = 8.2 Hz, 1H), 6.90 (d, *J* = 2.1 Hz, 1H), 6.88 (d, *J* = 2.1 Hz, 1H), 6.84 (d, *J* = 2.6 Hz, 1H), 6.82 (dd, *J* = 8.2, 2.7 Hz, 1H), 5.10 (h, *J* = 6.2 Hz, 1H), 3.85 (s, 3H), 2.30 (s, 3H), 1.67 (s, 6H), 1.21 (d, *J* = 6.3 Hz, 6H). **^{13}C NMR** (126 MHz, $CDCl_3$) δ 195.42, 173.33, 159.63, 159.31, 145.80, 136.86, 136.33, 133.69, 132.15, 130.93, 130.89, 129.84, 129.40, 117.35, 116.07, 111.45, 105.14, 79.52, 69.45, 55.43, 25.54, 21.68, 20.93. **Yield**: 92%, 12 h, as a white solid. **R_f:** 0.45 (25% EtOAc/hexanes). **Chemical Formula:** $C_{28}H_{30}O_4$ EI-MS [M^+ calcd: 430.2144. found: 430.2143.

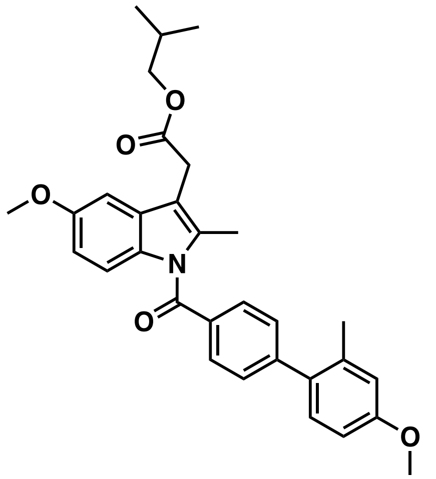

Isobutyl 2-(5-methoxy-1-(4'-methoxy-2'-methyl-[1,1'-biphenyl]-4-carbonyl)-2-methyl-1*H*-indol-3-yl)acetate (29a). Synthesized from isobutyl 2-(1-(4-chlorobenzoyl)-5-methoxy-2-methyl-1*H*-indol-3-yl)acetate and 2-methyl-4-methoxyphenyl boronic acid ($Pd(OAc)_2$ 0.25 mol %). **^{1}H NMR** (500 MHz, $CDCl_3$) δ 7.76 – 7.72 (m, 2H), 7.44 – 7.40 (m, 2H), 7.21 (d, *J* = 8.2 Hz, 1H), 7.00 (d, *J* = 6.0 Hz, 1H), 6.99 (s, 1H), 6.86 – 6.82 (m, 2H), 6.69 (dd, *J* = 9.1, 2.5 Hz, 1H), 3.89 (d, *J* = 6.6 Hz, 2H), 3.85 (d, *J* = 2.1 Hz, 6H), 3.69 (s, 2H), 2.42 (s, 3H), 2.30 (s, 3H), 1.92 (dp, *J* = 13.4, 6.7 Hz, 1H), 0.90 (d, *J* = 6.7 Hz, 6H). **^{13}C NMR** (126 MHz, $CDCl_3$) δ 171.17, 169.52, 159.46, 156.06, 146.68, 136.79, 136.04, 133.71, 133.32, 131.20, 130.88, 130.70, 129.89, 129.80, 116.14, 115.18, 112.51, 111.81, 111.54, 101.23, 71.20, 55.85, 55.44, 30.64, 27.87, 20.92, 19.18, 13.49. **Yield**: 88%, 12 h, as a yellow solid. **R_f:** 0.43 (25% EtOAc/hexanes). **Chemical Formula**: $C_{31}H_{33}NO_5$ EI-MS $[M]^+$ calcd: 499.2359, found: 499.2351.

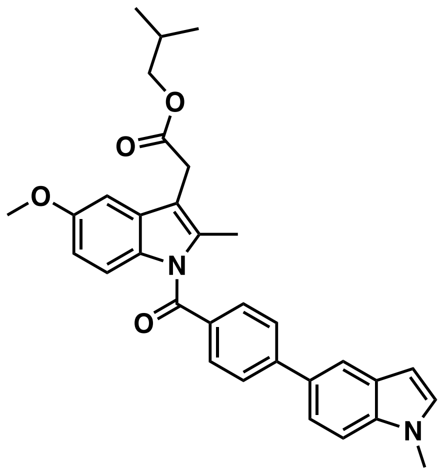

Isobutyl 2-(5-methoxy-2-methyl-1-(4-(1-methyl-1*H*-indol-5-yl)benzoyl)-1*H*-indol-3-yl)acetate (29b). Synthesized from isobutyl 2-(1-(4-chlorobenzoyl)-5-methoxy-2-methyl-1*H*-indol-3-yl)acetate and 1-methylindole-5-boronic acid ($Pd(OAc)_2$ 0.25 mol%). **^{1}H NMR** (600 MHz, $CDCl_3$) δ 7.92 (s, 1H), 7.78 – 7.73 (m, 4H), 7.53 (dt, *J* = 8.5, 1.6 Hz, 1H), 7.41 (d, *J* = 8.5 Hz, 1H), 7.11 – 7.08 (m, 1H), 7.01 – 6.97 (m, 2H), 6.67 (dt, *J* = 9.0, 1.9 Hz, 1H), 6.57 – 6.54 (m, 1H), 3.88 (dd, *J* = 6.6, 1.5 Hz, 2H), 3.83 (d, *J* = 1.5 Hz, 6H), 3.68 (s, 2H), 2.43 (d, *J* = 1.5 Hz, 3H), 1.91 (hept, *J* = 13.4, 6.7, 1.4 Hz, 1H), 0.89 (d, *J* = 6.7, 1.4 Hz, 6H). **^{13}C NMR** (151 MHz, $CDCl_3$) δ 171.24, 169.58, 155.97, 147.36, 136.15, 133.10, 131.24, 130.61, 130.03, 129.19, 127.43, 121.32, 119.99, 115.20, 112.27, 111.75, 109.88, 108.92, 101.77, 101.17, 71.19, 55.85, 33.15, 30.67, 27.88, 19.19, 13.45. **Yield**: 80%, 16 h, as a yellow solid. **R_f:** 0.40 (20% EtOAc/hexanes). **Chemical Formula:** $C_{32}H_{32}N_2O_4$ EI-MS $[M]^+$ calcd: 508.2362; found: 509.2369 [M+H].

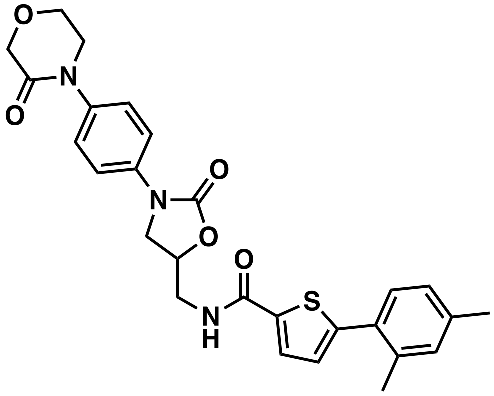

5-(2,4-Dimethylphenyl)-*N*-((2-oxo-3-(4-(3-oxomorpholino)phenyl)oxazolidin-5-yl)methyl)thiophene-2-carboxamide (30). Synthesized from rivaroxaban and 2,4-dimethylphenyl-boronic acid. DMSO 10% v/v used as a co-solvent at 60 °C with 0.5 mol % loading of palladium. **^{1}H NMR** (400 MHz, DMSO-d_6) δ 8.86 (t, *J* = 5.9 Hz, 1H), 7.76 (d, *J* = 3.8 Hz, 1H), 7.55 – 7.52 (m, 2H),

7.39 – 7.36 (m, 2H), 7.26 (d, *J* = 7.8 Hz, 1H), 7.15 (d, *J* = 3.8 Hz, 1H), 7.11 (s, 1H), 7.04 (dd, *J* = 8.0, 1.7 Hz, 1H), 4.83 (dd, *J* = 8.9, 5.5 Hz, 1H), 4.16 (s, 2H), 3.94 – 3.91 (m, 2H), 3.68 – 3.65 (m, 2H), 3.60 (t, *J* = 5.6 Hz, 2H), 2.32 (s, 3H), 2.26 (s, 3H). **^{13}C NMR** (101 MHz, DMSO-d_6) δ 165.95, 161.73, 154.14, 147.14, 138.31, 137.95, 137.04, 136.51, 135.10, 131.66, 130.00, 129.72, 128.75, 127.20, 126.92, 125.93, 118.32, 71.41, 67.72, 63.46, 49.00, 47.46, 42.18, 20.77, 20.63. **Yield**: 68%, as a white solid. **R$_f$:** 0.20 (5% methanol in DCM). **Chemical Formula:** $C_{27}H_{27}N_3O_5S$ EI-MS [M]$^+$ calcd: 505.1671; found: 538.2104 [M+MeOH+H].

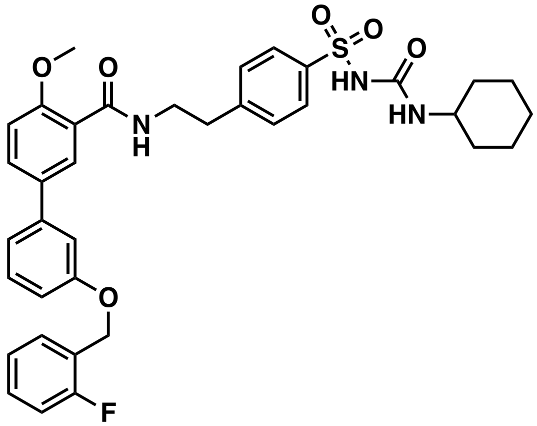

***N*-(4-(*N*-(Cyclohexylcarbamoyl)sulfamoyl)phenethyl)-3'-((2-fluorobenzyl)oxy)-4-methoxy-[1,1'-biphenyl]-3-carboxamide (31).** Prepared from glibenclamide and (3-((2-fluorobenzyl)oxy)phenyl) boronic acid. DMSO was used as a co-solvent at 55 °C with 0.5 mol % loading of palladium. **^{1}H NMR** (400 MHz, DMSO-d_6) δ 10.30 (bs, 3H), 8.23 (t, *J* = 5.7 Hz, 1H), 8.03 (s, 1H), 7.80 (d, *J* = 8.1 Hz, 2H), 7.60 (d, *J* = 2.8 Hz, 1H), 7.50 (d, *J* = 1.7 Hz, 1H), 7.45 (m, 3H), 7.42 – 7.34 (m, 2H), 7.22 (m, 2H), 7.11 (d, *J* = 8.9 Hz, 1H), 6.34 (d, *J* = 7.8 Hz, 1H), 5.09 (s, 1H), 3.76 (s, 3H), 3.51 (q, *J* = 6.7 Hz, 2H), 2.89 (t, *J* = 7.0 Hz, 2H), 1.56 (m, 4H), 1.46 – 1.41 (m, 1H), 1.23 – 0.99 (m, 6H). **^{13}C NMR** (101 MHz, DMSO-d_6) δ 163.67, 157.58, 155.70, 150.52, 145.20, 138.24, 131.53, 130.59, 130.33, 130.24, 129.52, 129.30, 128.66, 127.32, 126.86, 124.82, 124.55, 124.34, 124.00, 119.87, 116.54, 115.48, 115.28, 114.15, 109.59, 63.27, 56.23, 48.08, 40.20, 34.68, 32.30, 25.01, 24.20. **^{19}F NMR** (376 MHz, DMSO-d_6) δ -118.53 (ddd, *J* = 10.3, 7.4, 5.5 Hz). **Yield:** 61%, as a white solid. **R$_f$:** 0.15 (10% methanol in DCM). **Chemical Formula**: $C_{36}H_{38}FN_3O_6S$ EI-MS [M]$^+$ calcd: 659.2465; found: 692.3128 [M+H+MeOH].

General Procedure for Hydration of Alkynes

Precatalyst N$_2$Phos-gold(I) chloride synthesis was performed as previously described.[1]

Gold pre-catalyst synthesis. N$_2$Phos-gold(I) chloride (0.9 mg, 0.001 mmol) and silver(I)hexafluoro-antimonate (0.7 mg, 0.002 mmol) were charged under an inert atmosphere (glovebox) into a 1-dram vial containing a Teflon-coated magnetic stir bar and a rubber septum. The vial was covered with aluminum foil to protect it from light. Anhydrous DCM (1 mL) was added and the solution was stirred for 15 min.

1-Pot reaction set-up. To a dried 1-dram vial was added, under an argon atmosphere, 0.2 mL of the gold pre-catalyst solution (1000 ppm or 0.1 mol %). DCM was evaporated under argon. Alkyne (0.2 mmol, 1.0 equiv) was added to the vial, followed by toluene (20 μL), a 3 wt % TPGS-750-M/H_2O solution (0.2 mL, 1.0 M), and trifluoroacetic acid (46 mg, 0.4 mmol, 2.0 equiv). The resulting mixture was stirred at rt for 24 h. The reaction was then extracted with EtOAc. The organic layer was washed with H_2O, dried over anhydrous $MgSO_4$ and concentrated under vacuum. The products were purified by flash chromatography (100:0 → 80:20 hexanes/EtOAc).

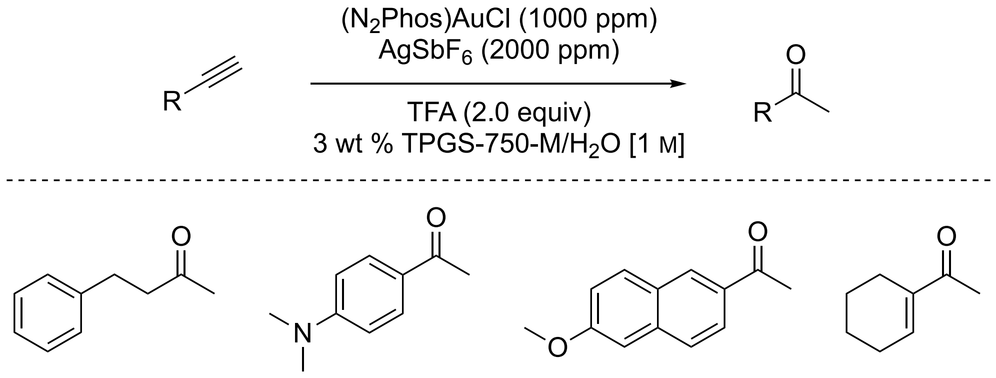

95% | 97% | 53% (39%)[1] | 90%

[1]with HandaPhos

---

[1] Klumphu, P. *et al.* Micellar catalysis-enabled sustainable ppm Au-catalyzed reactions in water at room temperature. *Chem. Sci.* **8,** 6354–6358 (2017).

Analytical Data for Products from Hydration of Alkynes

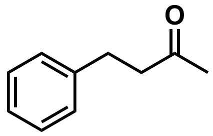

Benzylacetone. Synthesized according to general procedure B. **^{1}H NMR** (600 MHz, $CDCl_3$) δ 7.30 (dd, J = 8.6, 6.6 Hz, 2H), 7.23 – 7.18 (m, 3H), 2.91 (t, J = 7.7 Hz, 2H), 2.78 (dd, J = 8.3, 7.0 Hz, 2H), 2.15 (s, 3H). **^{13}C NMR** (151 MHz, $CDCl_3$) δ 208.1, 141.1, 128.6, 128.4, 126.2, 45.3, 30.2, 29.9, 6.6. **Yield:** 95%, as a colorless oil. **R_f:** 0.65 (20% EtOAc/hexanes) – UV + CAM stain.

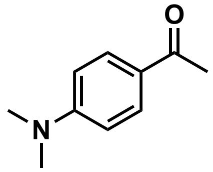

***N,N*-Dimethylaminoacetophenone.** Synthesized according to general procedure B. **^{1}H NMR** (600 MHz, $CDCl_3$) δ 7.90 – 7.84 (m, 2H), 6.69 – 6.62 (m, 2H), 3.08 – 3.04 (m, 6H), 2.51 (t, J = 0.9 Hz, 3H). **^{13}C NMR** (151 MHz, $CDCl_3$) δ 196.4, 153.5, 130.6, 125.4, 110.7, 40.1, 26.1. **Yield:** 97%, as a white powder. **R_f**: 0.35 (8:2 hexanes/EtOAc) – UV + CAM stain.

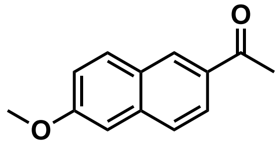

1-(6-Methoxynaphthalen-2-yl)ethan-1-one. Synthesized according to general procedure B. **^{1}H NMR** (600 MHz, $CDCl_3$) δ 8.40 (s, 1H), 8.01 (dd, J = 8.6, 1.8 Hz, 1H), 7.85 (d, J = 8.9 Hz, 1H), 7.77 (d, J = 8.6 Hz, 1H), 7.21 (dd, J = 9.0, 2.5 Hz, 1H), 7.16 (d, J = 2.5 Hz, 1H), 3.95 (s, 3H), 2.70 (s, 3H). **^{13}C NMR** (151 MHz, $CDCl_3$) δ 198.0, 159.9, 137.4, 132.8, 131.2, 130.2, 128.0, 127.2, 124.8, 119.9, 105.9, 55.6, 26.7. **Yield:** 53%, as a beige solid. **R_f:** 0.46 (20% EtOAc/hexanes) – UV + CAM stain.

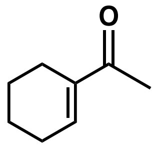

1-Acetyl-1-cyclohexene. Synthesized according to general procedure B. **^{1}H NMR** (600 MHz, $CDCl_3$) δ 6.93 – 6.87 (m, 1H), 2.27 (t, J = 1.1 Hz, 3H), 2.27 – 2.18 (m, 4H), 1.68 – 1.55 (m, 4H). **^{13}C NMR** (151 MHz, $CDCl_3$) δ 199.6, 141.1, 139.8, 26.2, 25.3, 23.1, 22.0, 21.6. **Yield:** 90%, as a colorless oil. **R_f**: 0.33 (15% EtOAc/hexanes) CAM stain.

Procedure for the 1-pot process

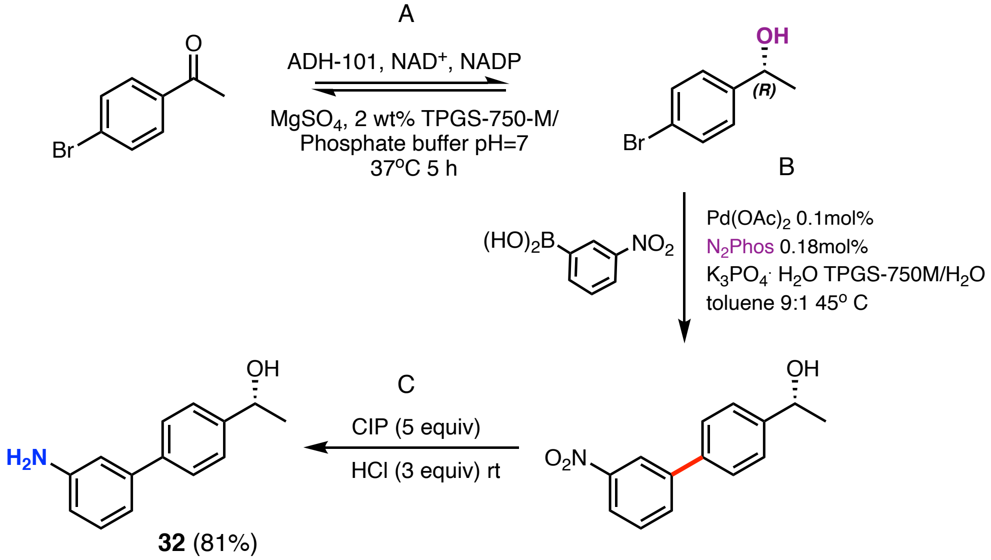

32 (81%)

ADH-101[1-3] is commercially available within the enzyme kit EZK-001 from Johnson Matthey. NAD^+ was purchased from Bioworld and $NADP^+$ was purchased from Chem-Impes. Isopropanol was purchased from VWR. All other commercially available reagents were used without further purification. To a 2-dram vial equipped with a stir bar was added the ketone (0.2 mmol, 1 equiv), $MgSO_4$ (0.8 mg), NAD^+ (2.6 mg) and $NADP^+$ (2.4 mg). *i*-PrOH (0.1 mL) and a [0.2 M] phosphate buffer solution at pH = 7 (0.7 mL) and 2 wt % aqueous TPGS-750-M were added. ADH-101 (20 mg) was then added. The reaction was set to stir at 37 °C for 16 h. The reaction was monitored by HPLC. Upon completion, the reaction was charged with the arylboronic acid (0.3 mmol, 1.5 equiv) and potassium phosphate (0.3 mmol, 1.5 equiv). The reaction vial was fitted with a rubber septum and sealed with Teflon tape. The reaction vial was purged with argon with the use of a vent needle. At this point a solution of $Pd(OAc)_2$ and N_2Phos in toluene (0.05 mL) was added via syringe. The reaction was set to stir at 45 °C and monitored by

either GC/MS or TLC analysis. Upon completion of the reaction, carbonyl Fe powder (CIP, 5 equiv) and HCl (3 equiv) was added to the reaction vial. The reaction was set to stir at rt and monitored by TLC analysis. Upon completion, the reaction was extracted with EtOAc and the organic layer was washed with brine three times. The layers were then separated and the organic layer was dried over anhydrous Na_2SO_4. The mixture was concentrated under vacuum and then purified via flash chromatography (40% EtOAc in hexanes). **^{1}H NMR** (400 MHz, $CDCl_3$) δ 7.55 (d, *J* = 8.3 Hz, 2H), 7.43 (d, *J* = 8.3 Hz, 2H), 7.22 (t, *J* = 7.8 Hz, 1H), 6.99 (d, *J* = 8.3 Hz, 1H), 6.90 (t, *J* = 2.0 Hz, 1H), 6.68 (dd, *J* = 7.9, 1.4 Hz, 1H), 4.95 (q, *J* = 6.5 Hz, 1H), 3.74 (s, 2H), 1.54 (d, *J* = 6.5 Hz, 3H). **^{13}C NMR** (101 MHz, $CDCl_3$) δ 146.84, 144.88, 142.20, 140.77, 129.83, 127.35, 125.87, 117.75, 114.24, 113.94, 70.35, 25.27. **Yield**: 81%**,** as a yellow solid. **R_f:** 0.33 (40% EtOAc in hexanes). **Chemical Formula**: $C_{14}H_{15}NO$ EI-MS $[M]^+$ calcd: 213.1154; found: 213.1147.

References:

1. Niefind, K., Müller, J., Riebel, B., Hummel, W. & Schomburg, D. The crystal structure of R-specific alcohol dehydrogenase from Lactobacillus brevis suggests the structural basis of its metal dependency. *J. Mol. Biol.* **2003***, 327*, 317–328.
2. Schlieben, N. H. et al. Atomic resolution structures of R-specific alcohol dehydrogenase from Lactobacillus brevis provide the structural bases of its substrate and cosubstrate specificity. *J. Mol. Biol.* **2005**, *349*, 801–813.
3. Cortes-Clerget, M.; Akporji, N.; Zhou, J.; Gao, F.; Guo, P.; Parmentier, M.; Gallou, F.; Berthon, J.-Y.; Lipshutz, B. H. Bridging the Gap between Transition Metal- and Bio-Catalysis via Aqueous Micellar Catalysis. *Nat. Commun.* **2019**, *10*, 2169.

^{1}H, ^{13}C, ^{31}P and ^{19}F NMR of synthesized products

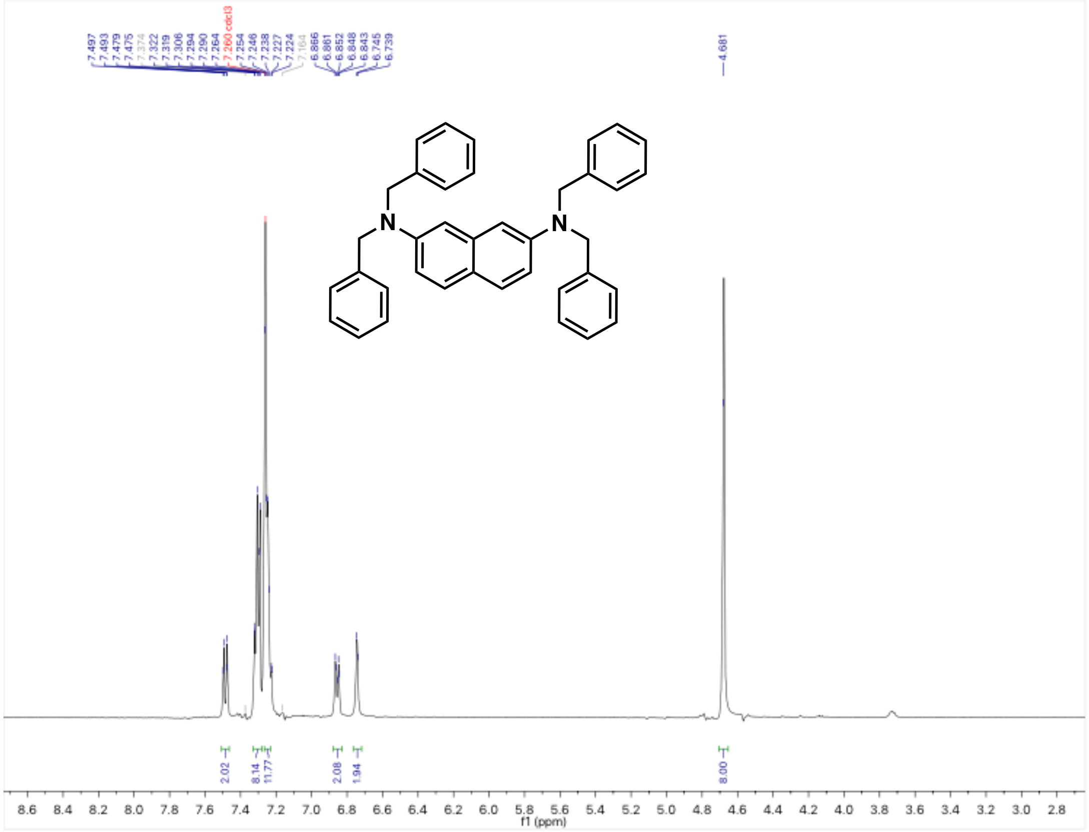

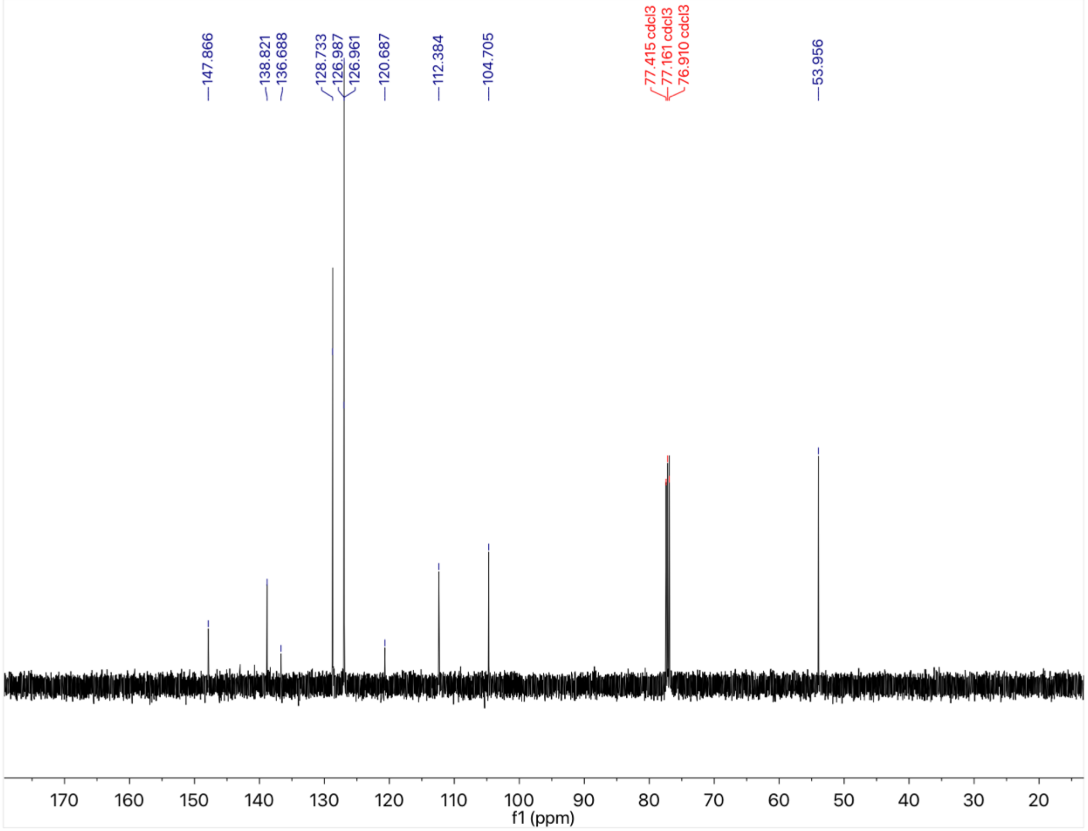

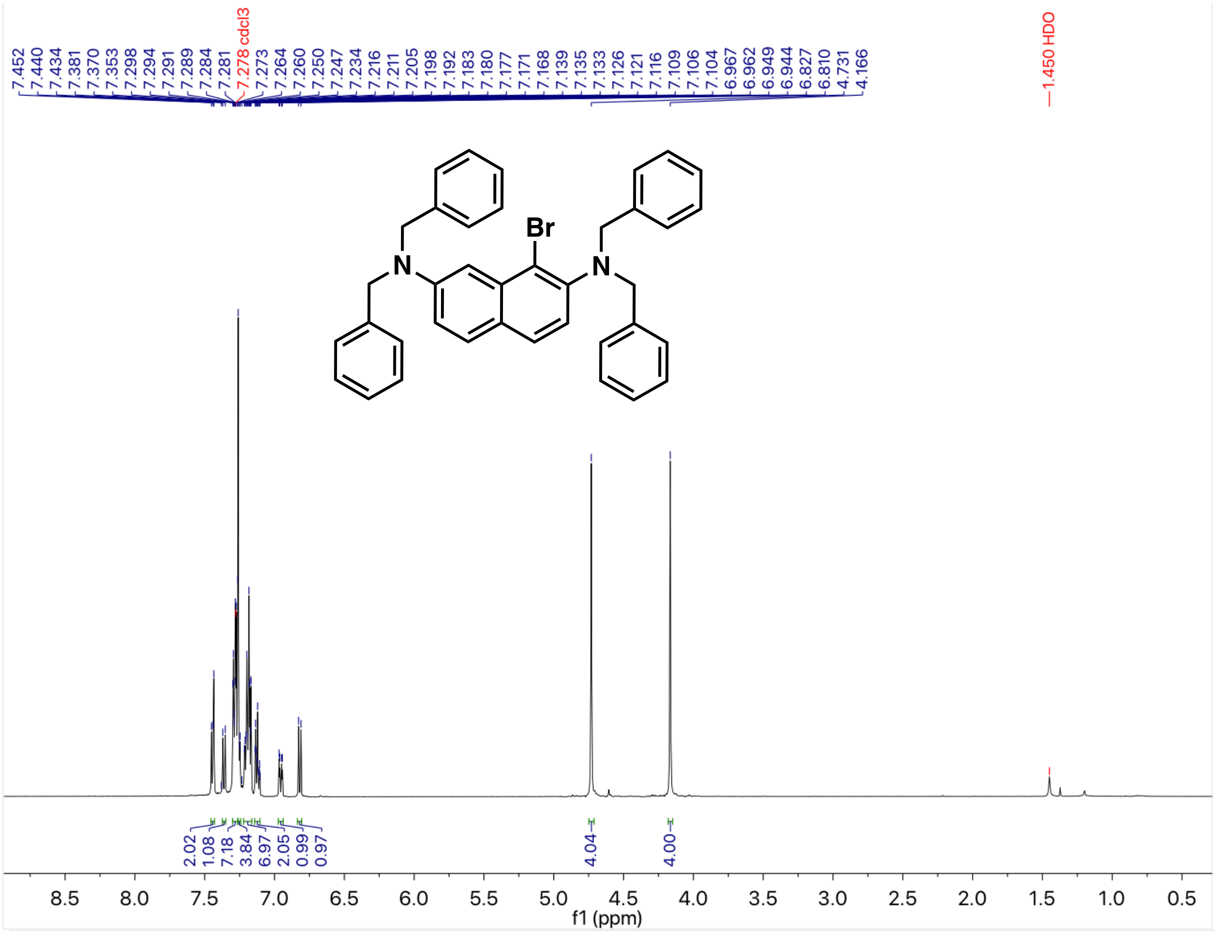
7.452
7.440
7.434
7.381
7.370
7.353
7.298
7.294
7.291
7.289
7.284
7.281
7.278 cdcl3
7.273
7.264
7.260
7.250
7.247
7.234
7.216
7.211
7.205
7.198
7.192
7.183
7.180
7.177
7.171
7.168
7.139
7.135
7.133
7.126
7.121
7.116
7.109
7.106
7.104
6.967
6.962
6.949
6.944
6.827
6.810
4.731
4.166
1.450 HDO
Br
N
N
2.02
1.08
7.18
3.84
6.97
2.05
0.99
0.97
4.04
4.00
8.5 8.0 7.5 7.0 6.5 6.0 5.5 5.0 4.5 4.0 3.5 3.0 2.5 2.0 1.5 1.0 0.5
f1 (ppm)
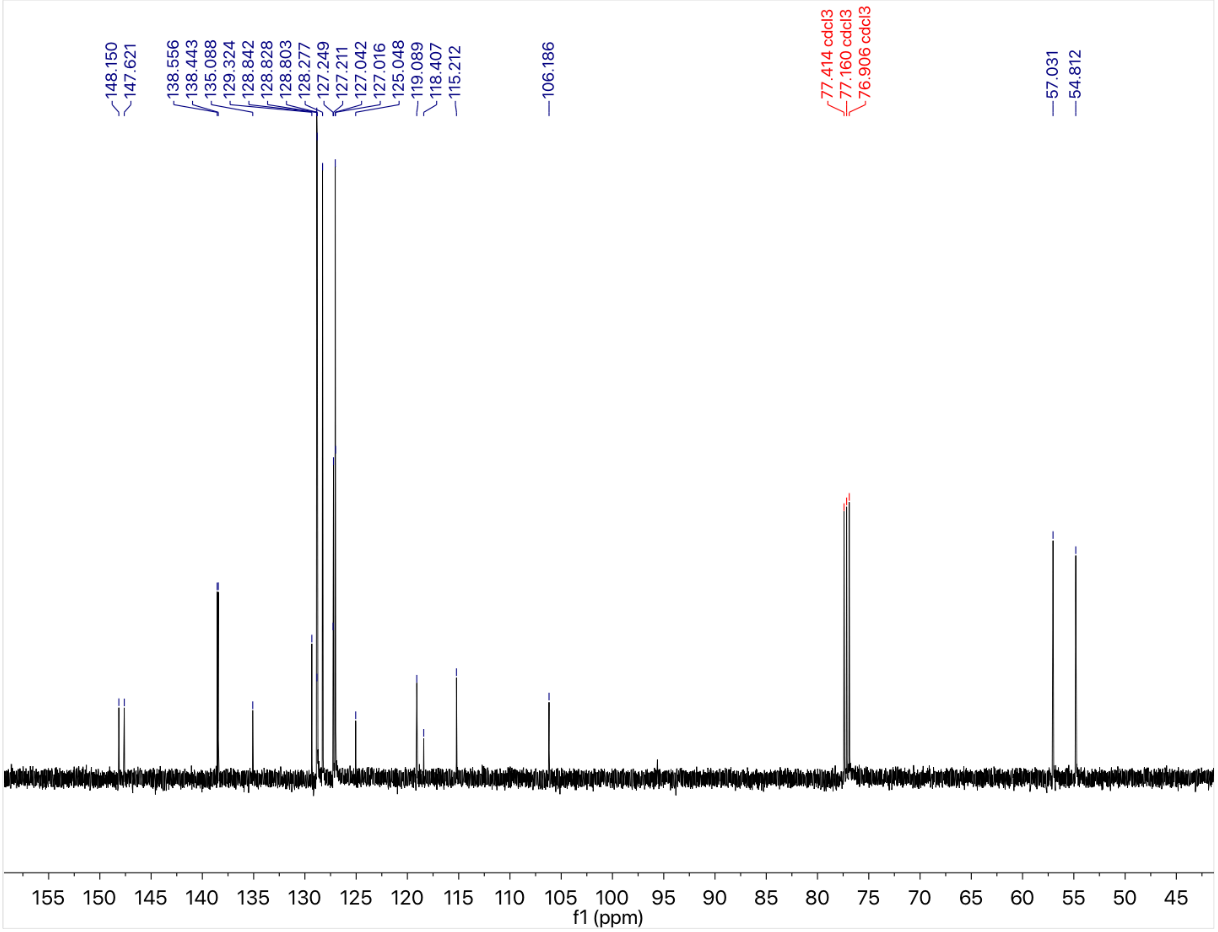
148.150
147.621
138.556
138.443
135.088
129.324
128.842
128.828
128.803
128.277
127.249
127.211
127.042
127.016
125.048
119.089
118.407
115.212
106.186
77.414 cdcl3
77.160 cdcl3
76.906 cdcl3
57.031
54.812
155 150 145 140 135 130 125 120 115 110 105 100 95 90 85 80 75 70 65 60 55 50 45
f1 (ppm)

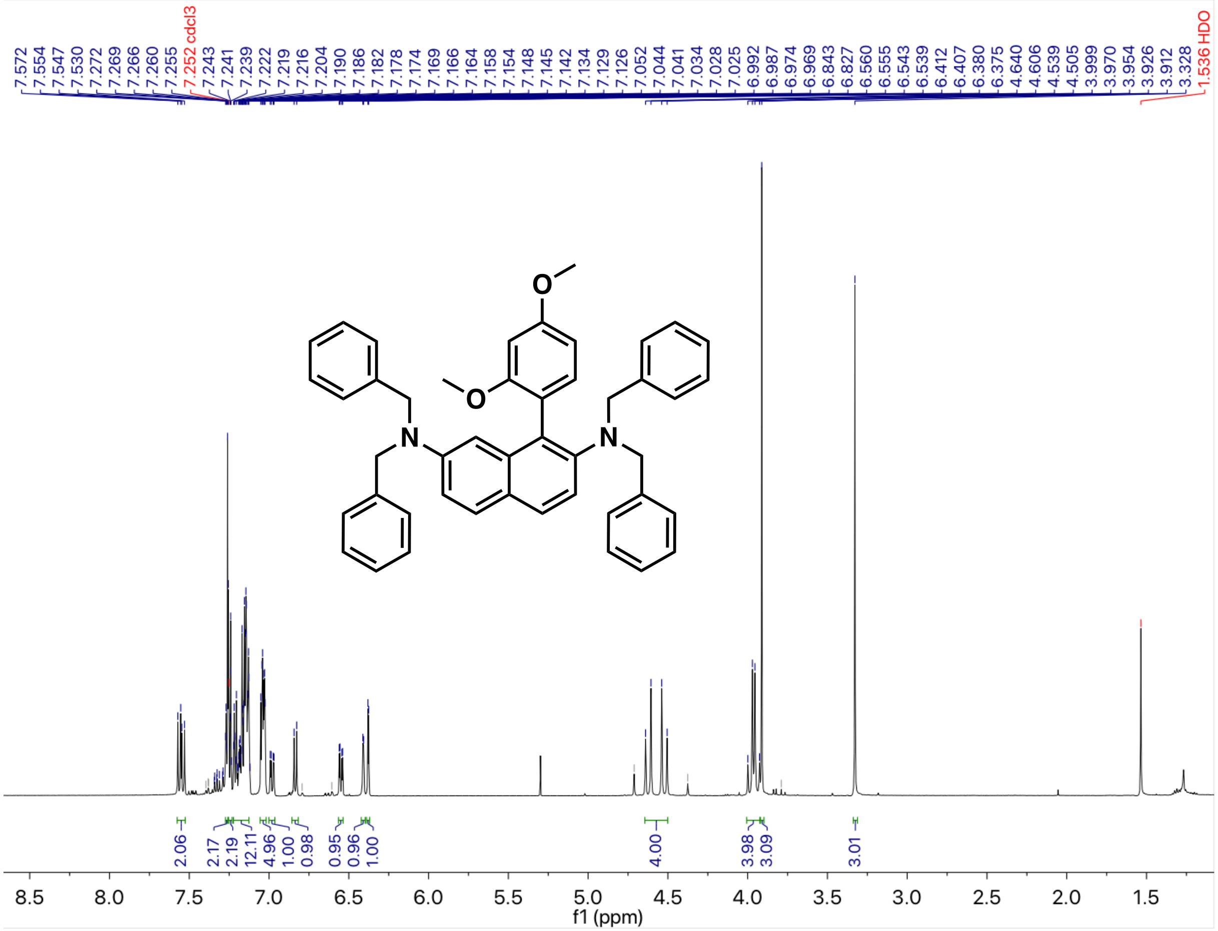
7.572
7.554
7.547
7.530
7.272
7.269
7.266
7.260
7.255
7.252 cdcl3
7.243
7.241
7.239
7.222
7.219
7.216
7.204
7.190
7.186
7.182
7.178
7.174
7.169
7.166
7.164
7.158
7.154
7.148
7.145
7.142
7.134
7.129
7.126
7.052
7.044
7.041
7.034
7.028
7.025
6.992
6.987
6.974
6.969
6.843
6.827
6.560
6.555
6.543
6.539
6.412
6.407
6.380
6.375
4.640
4.606
4.539
4.505
3.999
3.970
3.954
3.926
3.912
3.328
1.536 HDO
O
O
N
N
2.06
2.17
2.19
12.11
4.96
1.00
0.98
0.95
0.96
1.00
4.00
3.98
3.09
3.01
8.5
8.0
7.5
7.0
6.5
6.0
5.5
5.0
4.5
4.0
3.5
3.0
2.5
2.0
1.5
f1 (ppm)

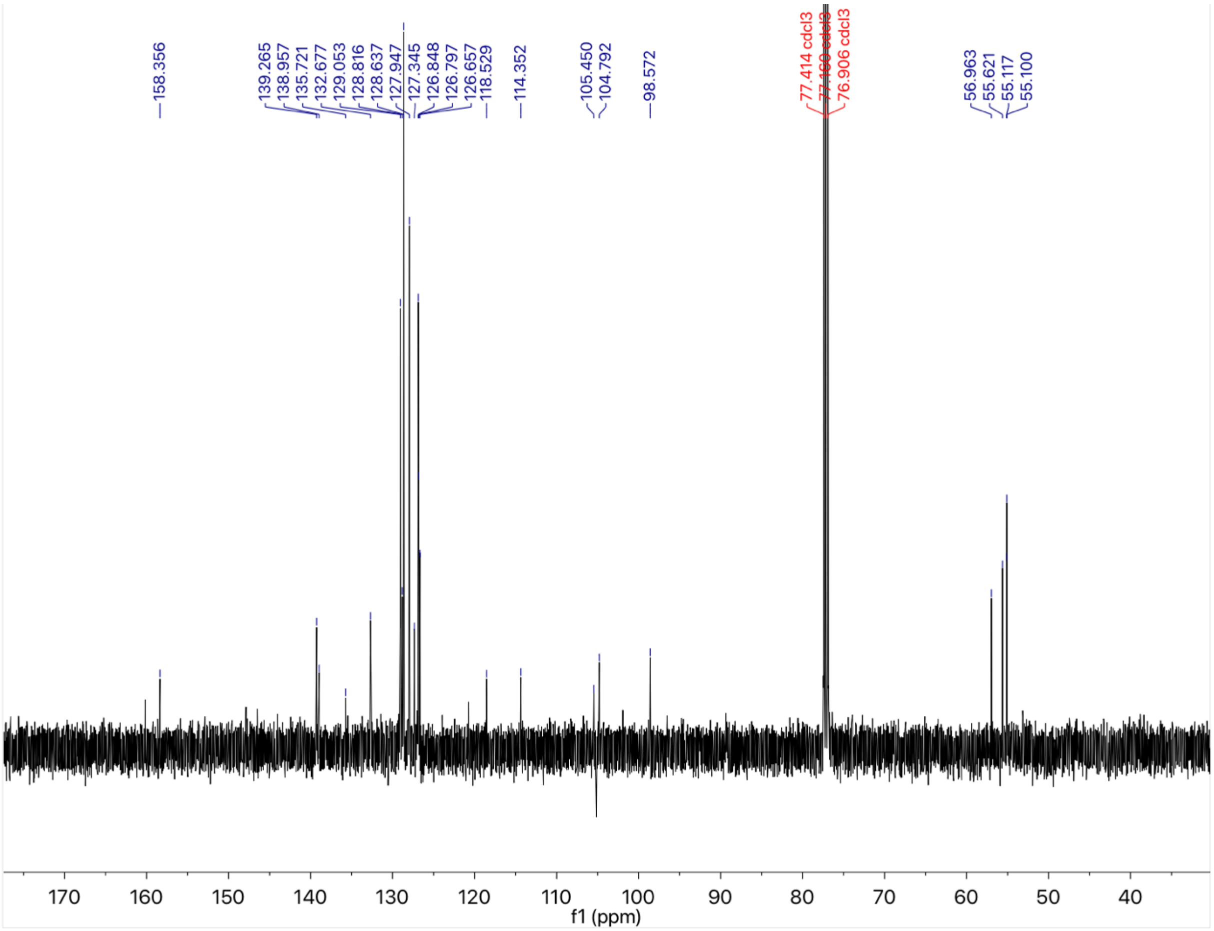
158.356
139.265
138.957
135.721
132.677
129.053
128.816
128.637
127.947
127.345
126.848
126.797
126.657
118.529
114.352
105.450
104.792
98.572
77.414 cdcl3
77.160 cdcl3
76.906 cdcl3
56.963
55.621
55.117
55.100
170
160
150
140
130
120
110
100
90
80
70
60
50
40
f1 (ppm)

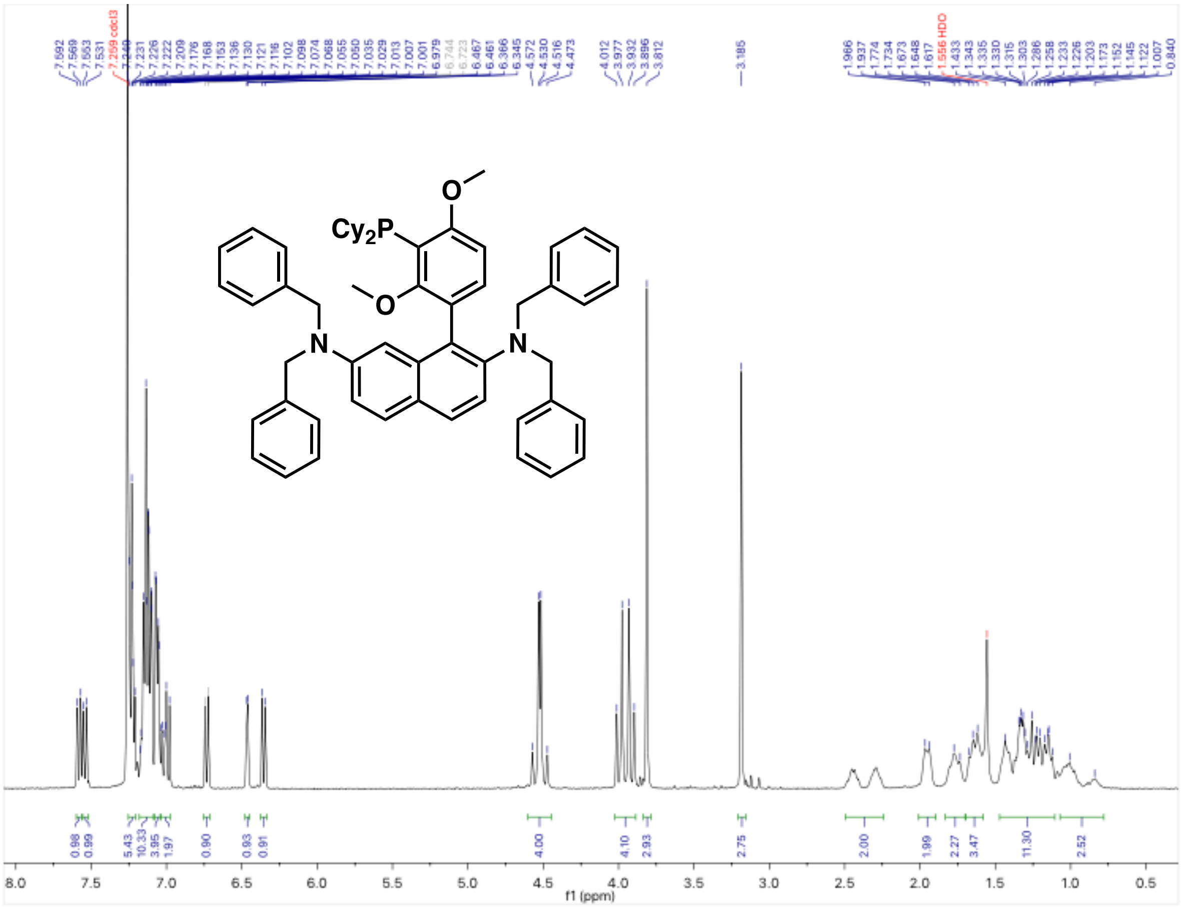

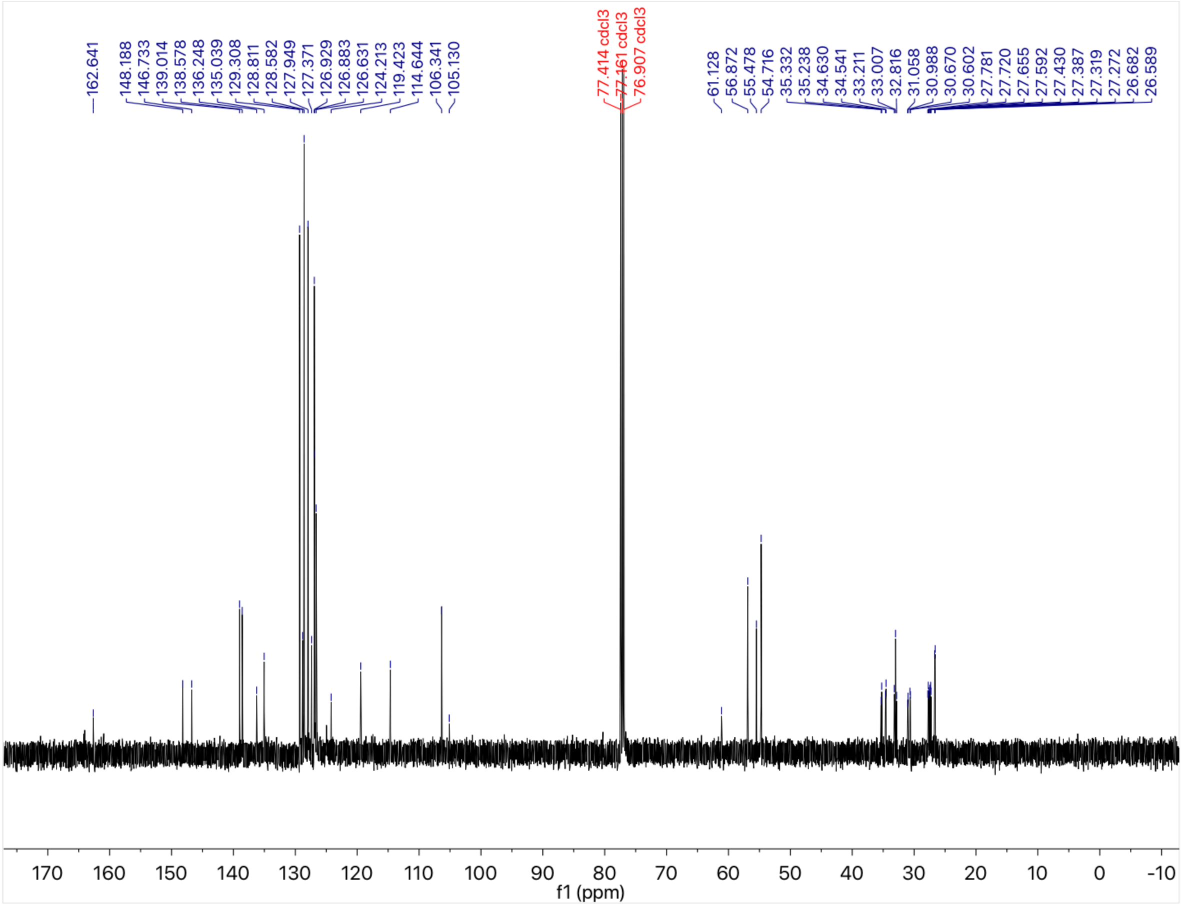

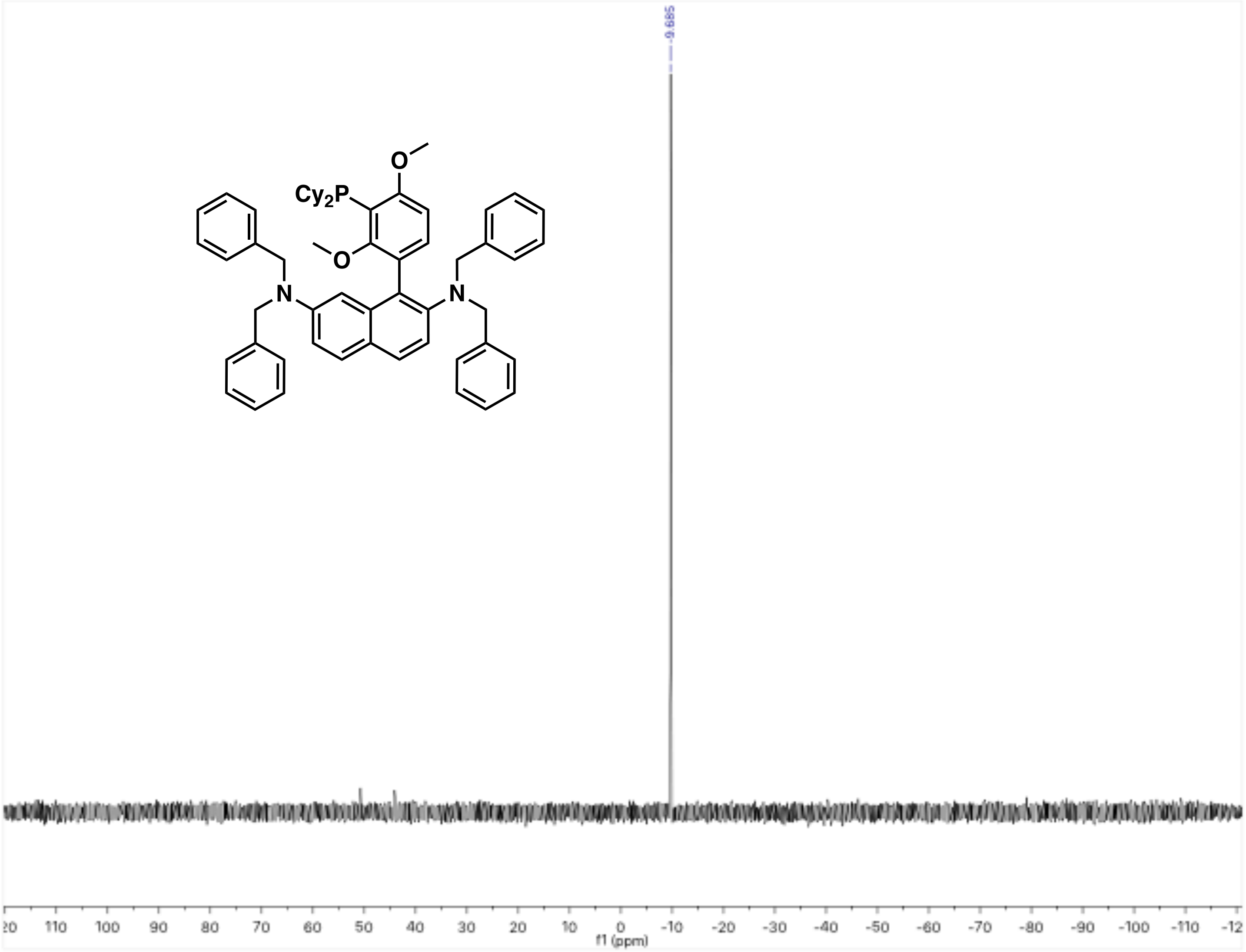
-9.685
Cy2P
O
O
N
N
20
110
100
90
80
70
60
50
40
30
20
10
0
-10
-20
-30
-40
-50
-60
-70
-80
-90
-100
-110
-12
f1 (ppm)

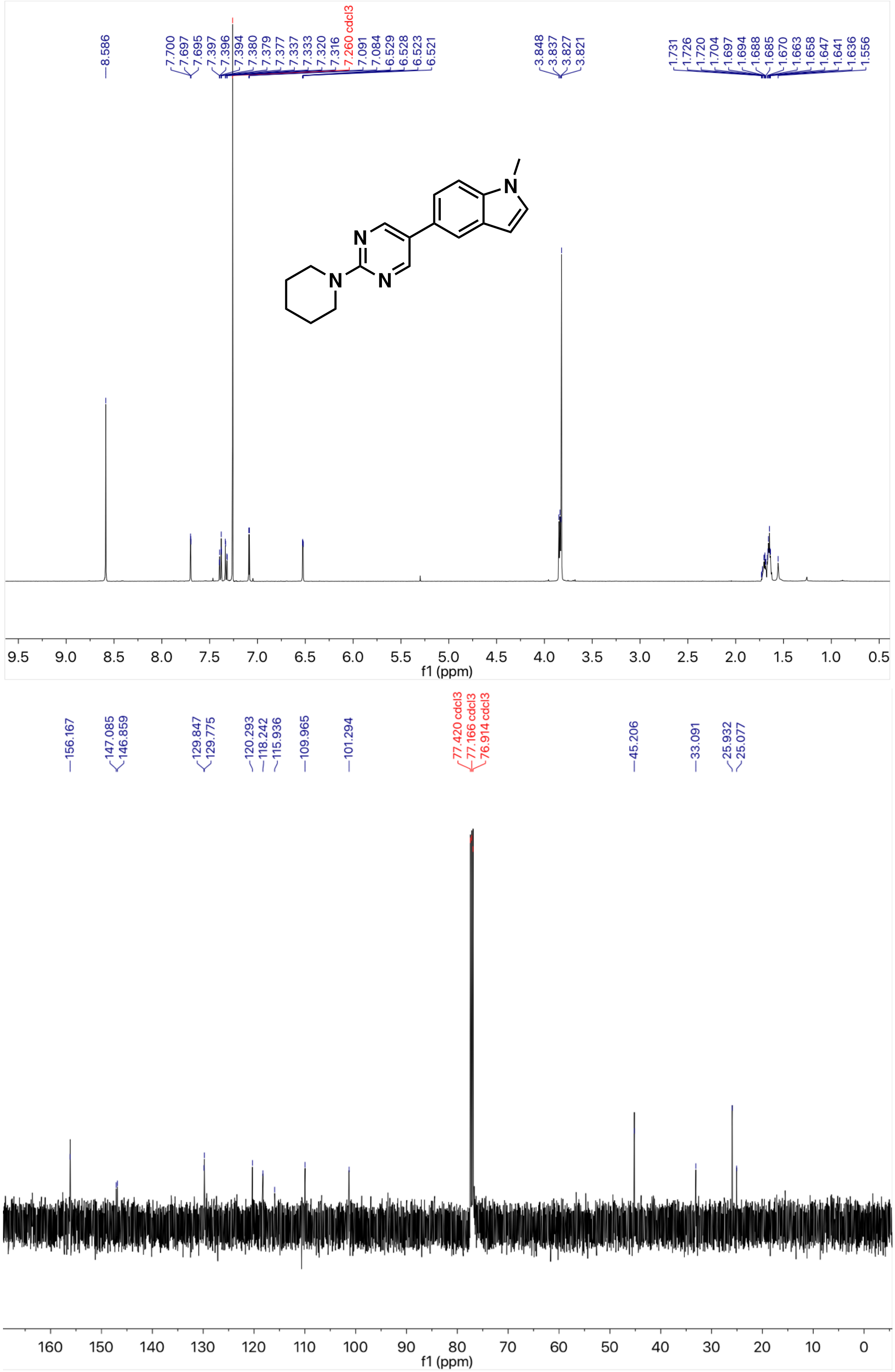

8.586
7.700
7.697
7.695
7.397
7.396
7.394
7.380
7.379
7.377
7.337
7.333
7.320
7.316
7.260 cdcl3
7.091
7.084
6.529
6.528
6.523
6.521
3.848
3.837
3.827
3.821
1.731
1.726
1.720
1.704
1.697
1.694
1.688
1.685
1.670
1.663
1.658
1.647
1.641
1.636
1.556
N
N
N
N
f1 (ppm)
156.167
147.085
146.859
129.847
129.775
120.293
118.242
115.936
109.965
101.294
77.420 cdcl3
77.166 cdcl3
76.914 cdcl3
45.206
33.091
25.932
25.077
f1 (ppm)

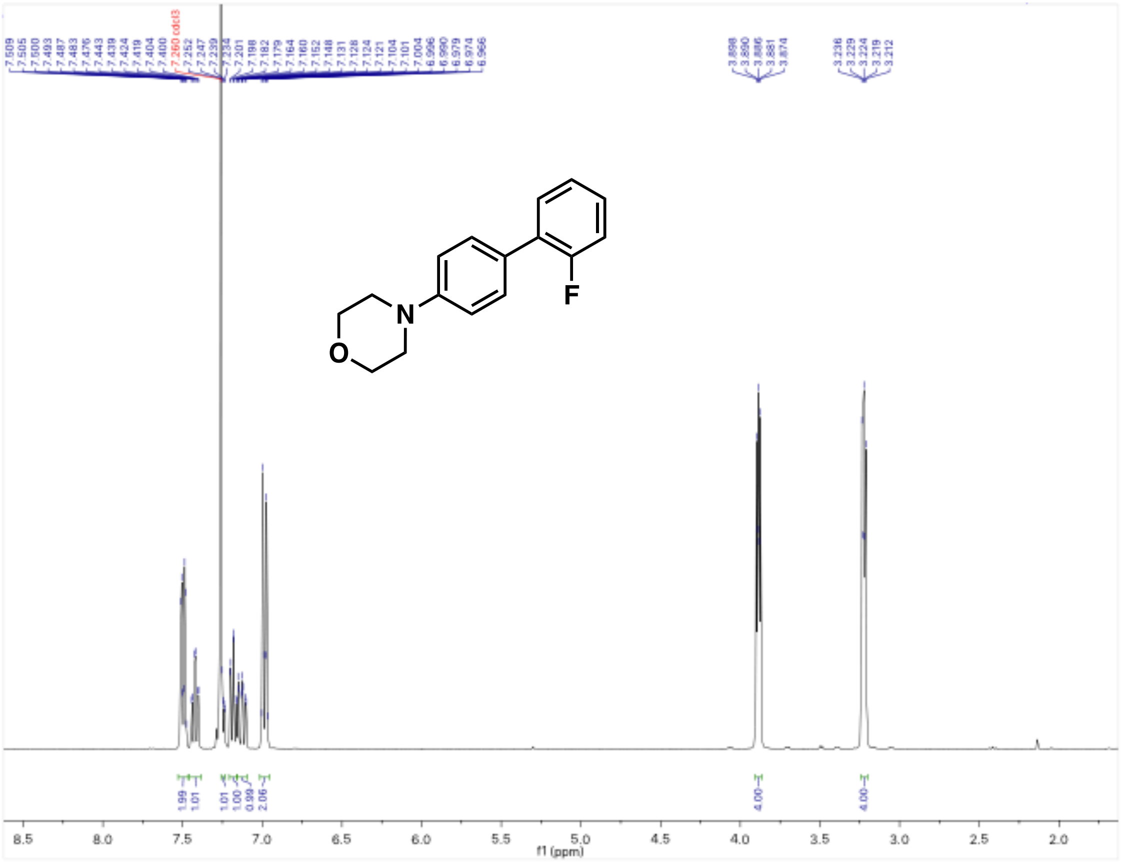

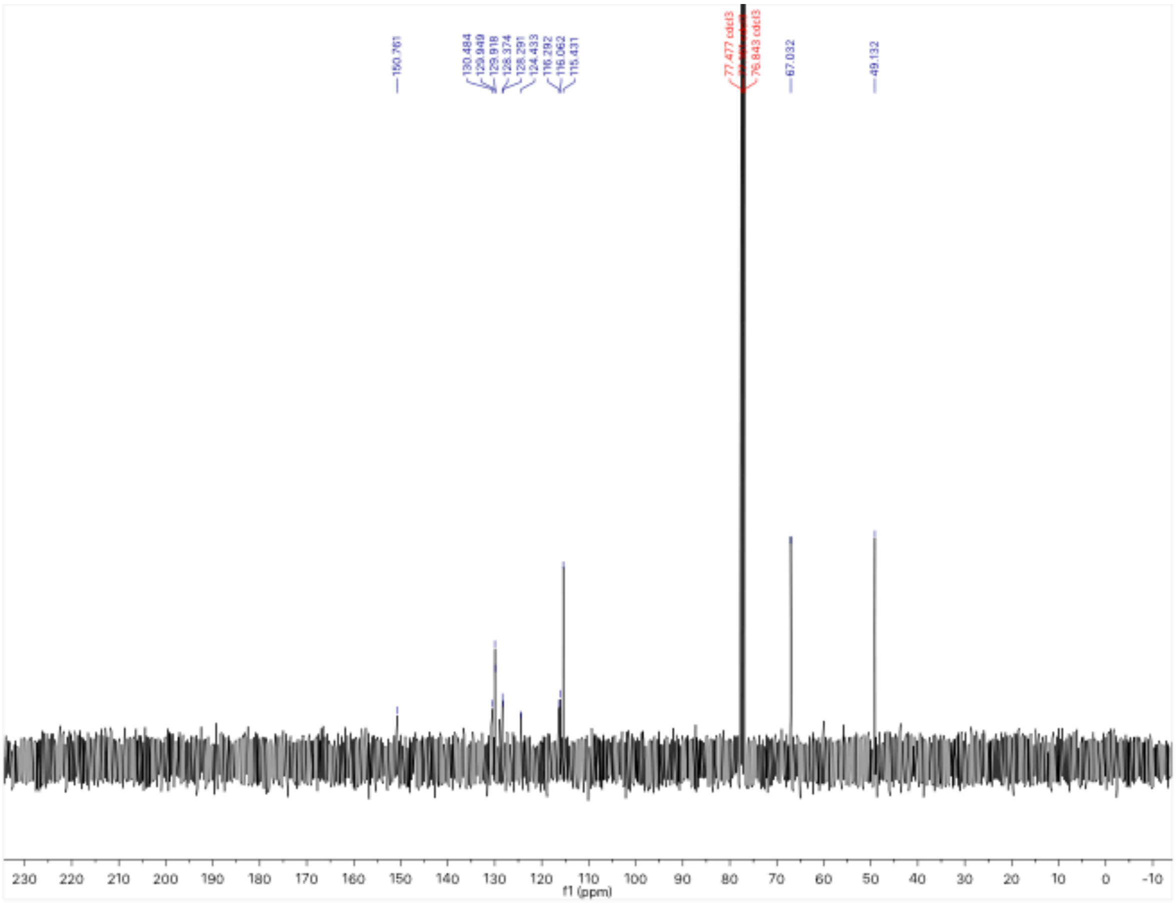

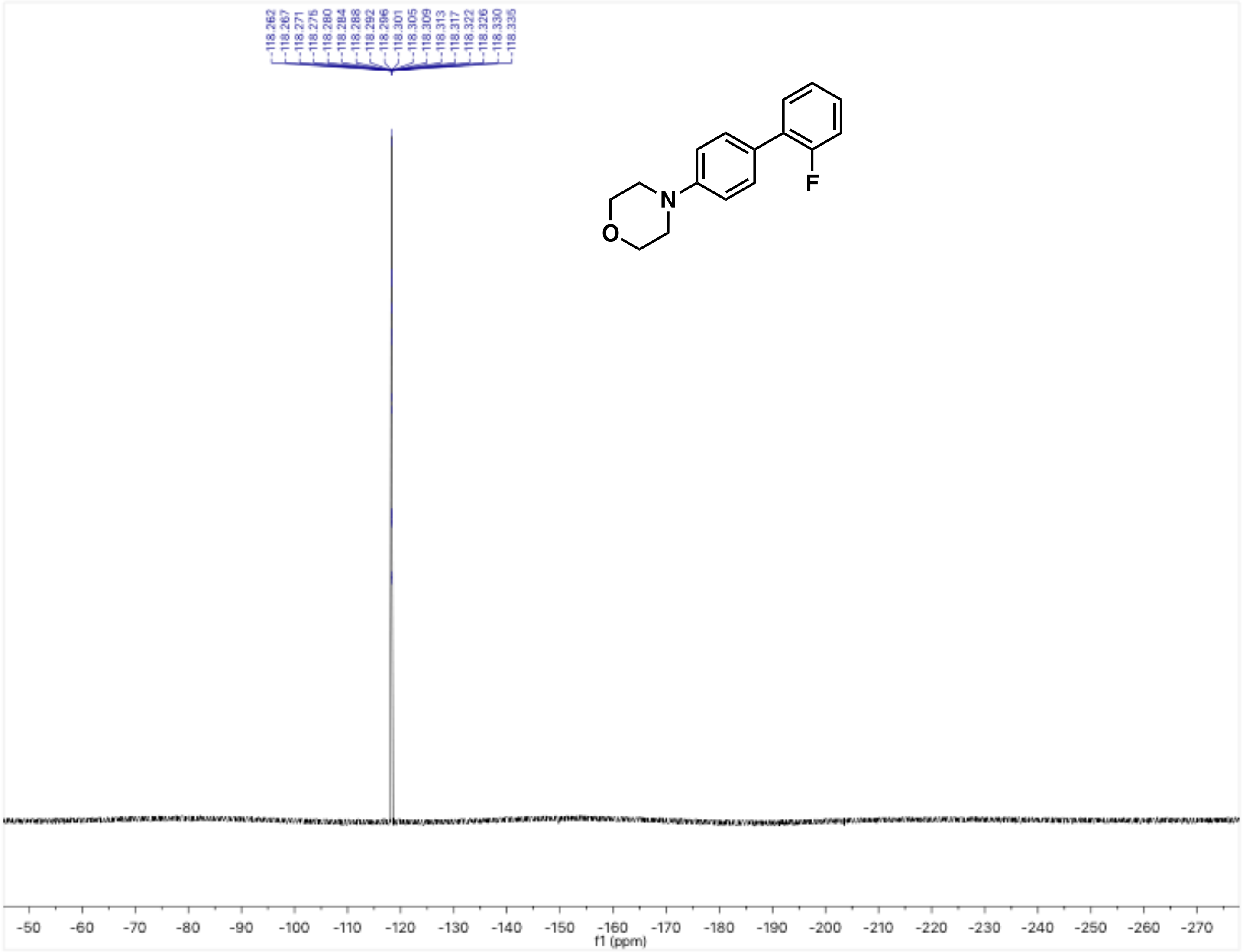

-118.262
-118.267
-118.271
-118.275
-118.280
-118.284
-118.288
-118.292
-118.296
-118.301
-118.305
-118.309
-118.313
-118.317
-118.322
-118.326
-118.330
-118.335
N
O
F
-50 -60 -70 -80 -90 -100 -110 -120 -130 -140 -150 -160 -170 -180 -190 -200 -210 -220 -230 -240 -250 -260 -270
f1 (ppm)

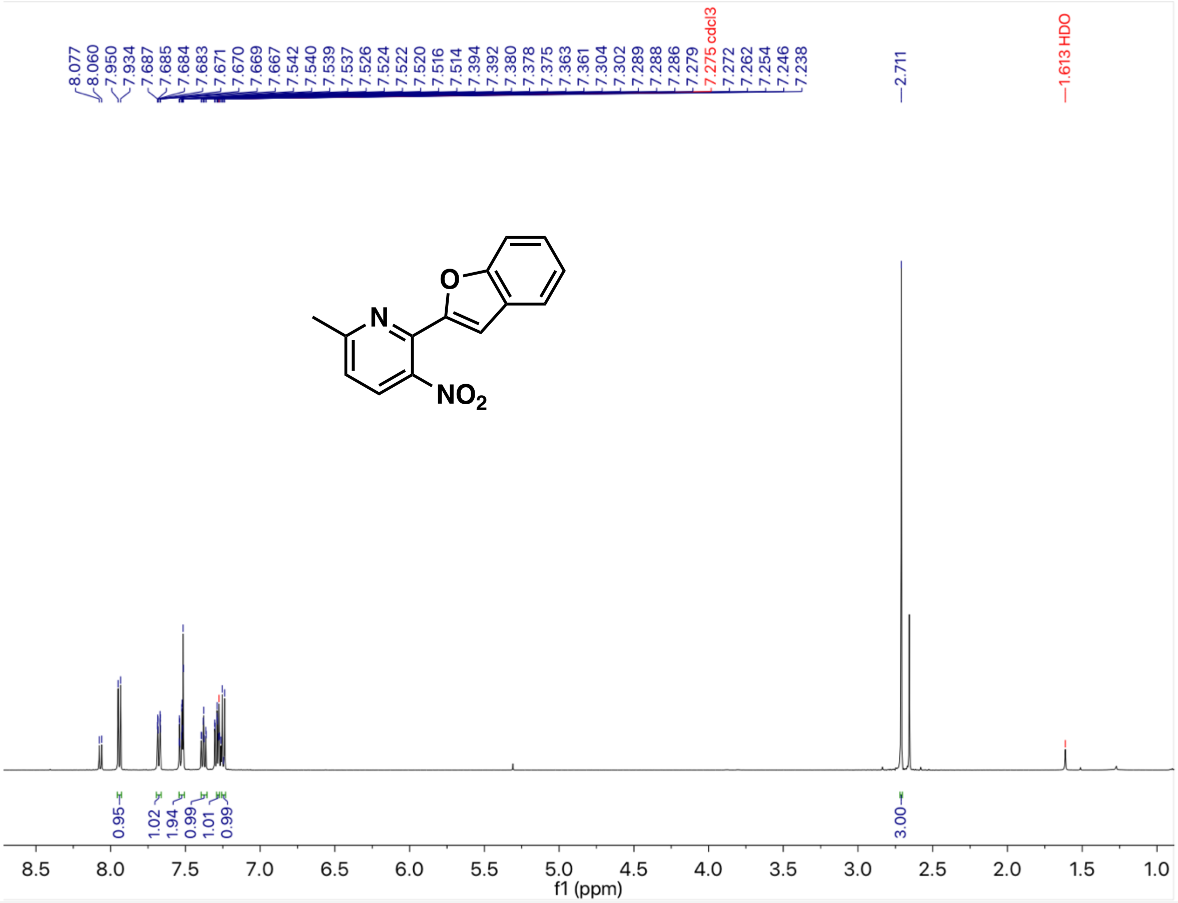
8.077
8.060
7.950
7.934
7.687
7.685
7.684
7.683
7.671
7.670
7.669
7.667
7.542
7.540
7.539
7.537
7.526
7.524
7.522
7.520
7.516
7.514
7.394
7.392
7.380
7.378
7.375
7.363
7.361
7.304
7.302
7.289
7.288
7.286
7.279
7.275 cdcl3
7.272
7.262
7.254
7.246
7.238
2.711
1.613 HDO
N
O
NO_2
0.95
1.02
1.94
0.99
1.01
0.99
3.00
8.5 8.0 7.5 7.0 6.5 6.0 5.5 5.0 4.5 4.0 3.5 3.0 2.5 2.0 1.5 1.0
f1 (ppm)

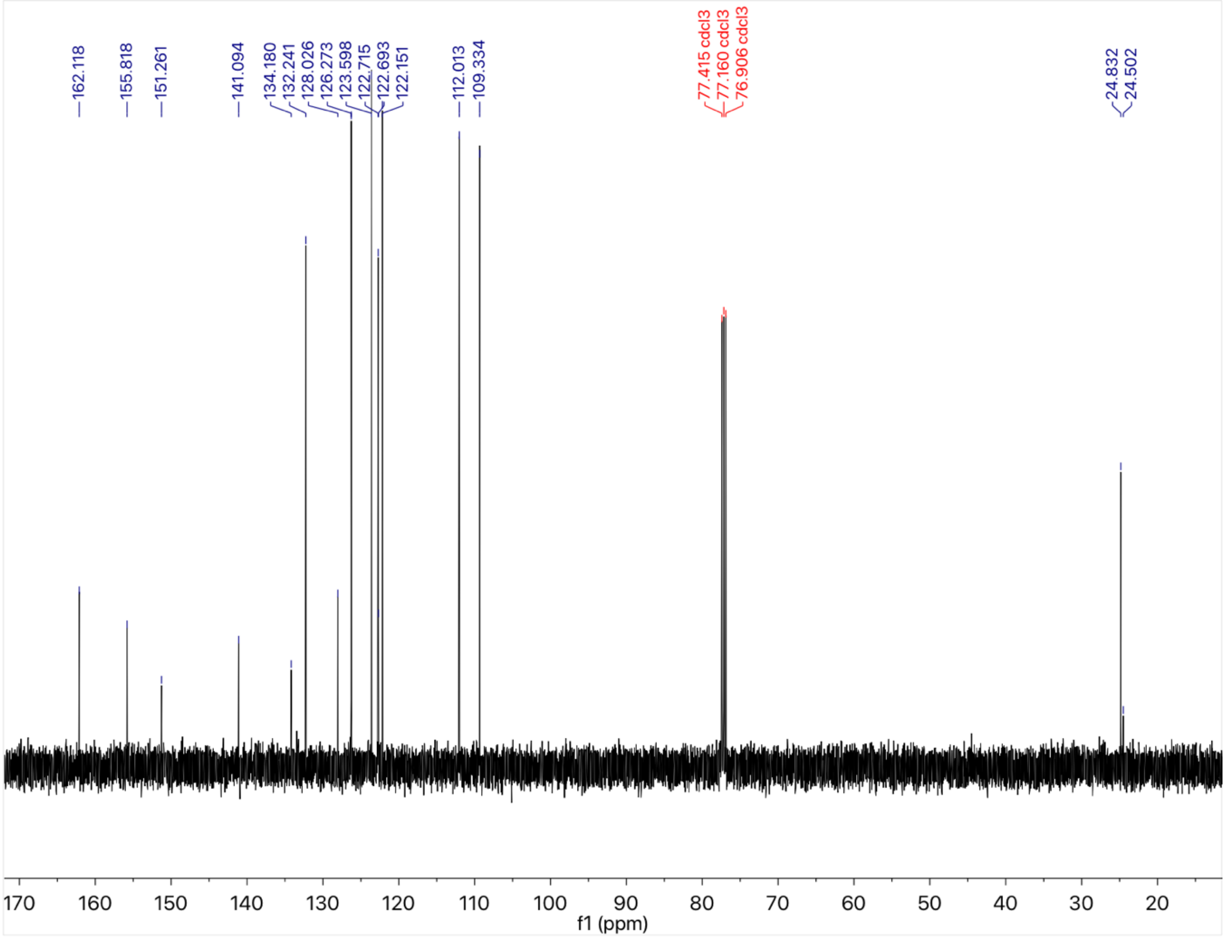
162.118
155.818
151.261
141.094
134.180
132.241
128.026
126.273
123.598
122.715
122.693
122.151
112.013
109.334
77.415 cdcl3
77.160 cdcl3
76.906 cdcl3
24.832
24.502
170 160 150 140 130 120 110 100 90 80 70 60 50 40 30 20
f1 (ppm)

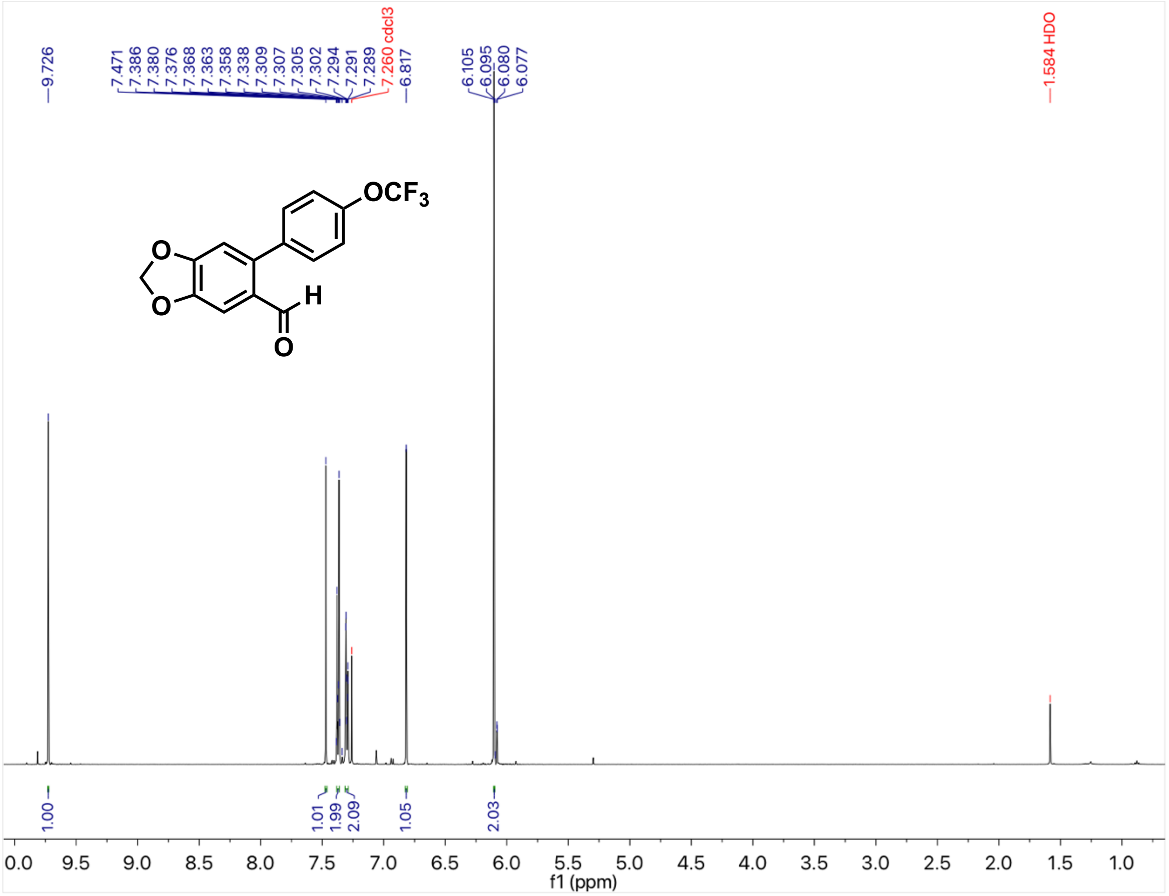
9.726
7.471
7.386
7.380
7.376
7.368
7.363
7.358
7.338
7.309
7.307
7.305
7.302
7.294
7.291
7.289
7.260 cdcl3
6.817
6.105
6.095
6.080
6.077
1.584 HDO
OCF3
O
O
H
O
1.00
1.01
1.99
2.09
1.05
2.03
f1 (ppm)

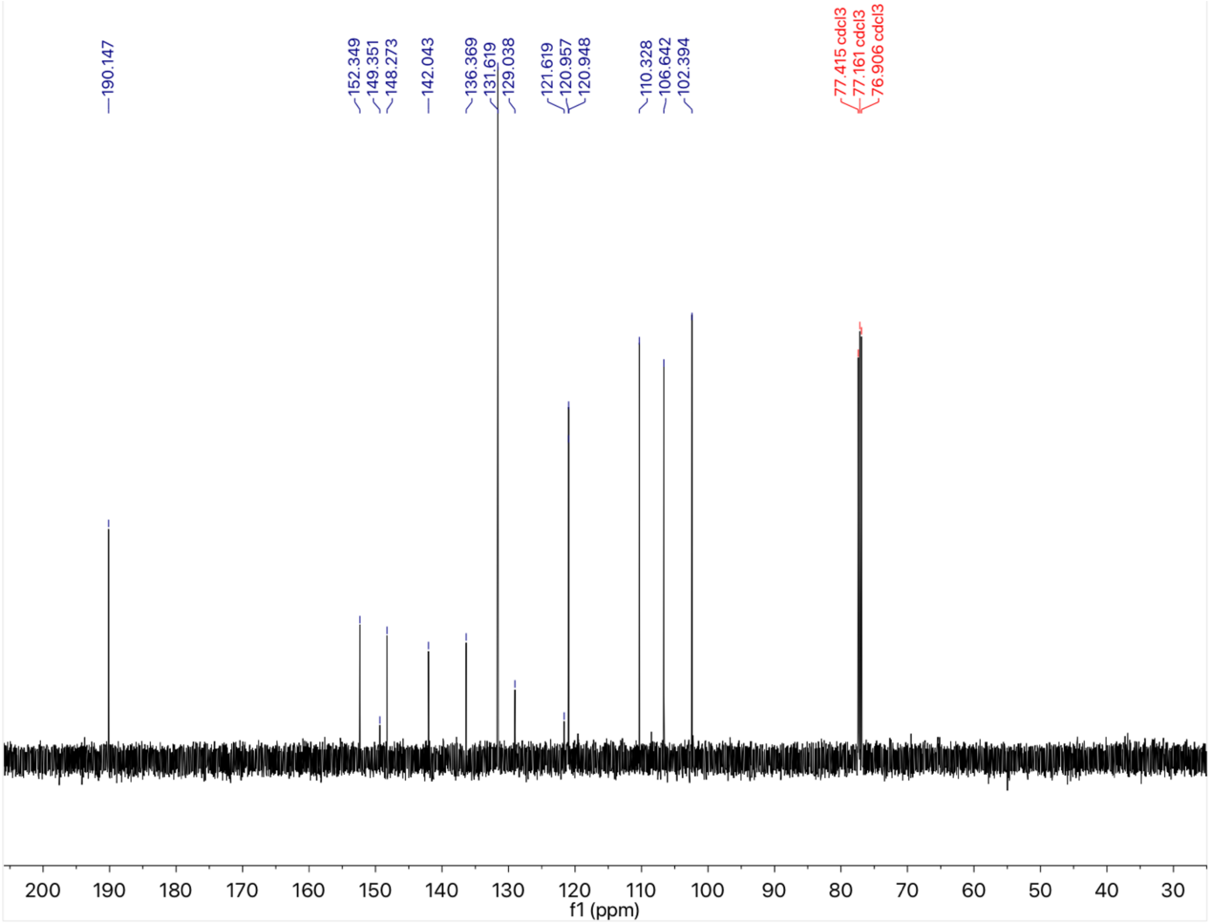
190.147
152.349
149.351
148.273
142.043
136.369
131.619
129.038
121.619
120.957
120.948
110.328
106.642
102.394
77.415 cdcl3
77.161 cdcl3
76.906 cdcl3
f1 (ppm)

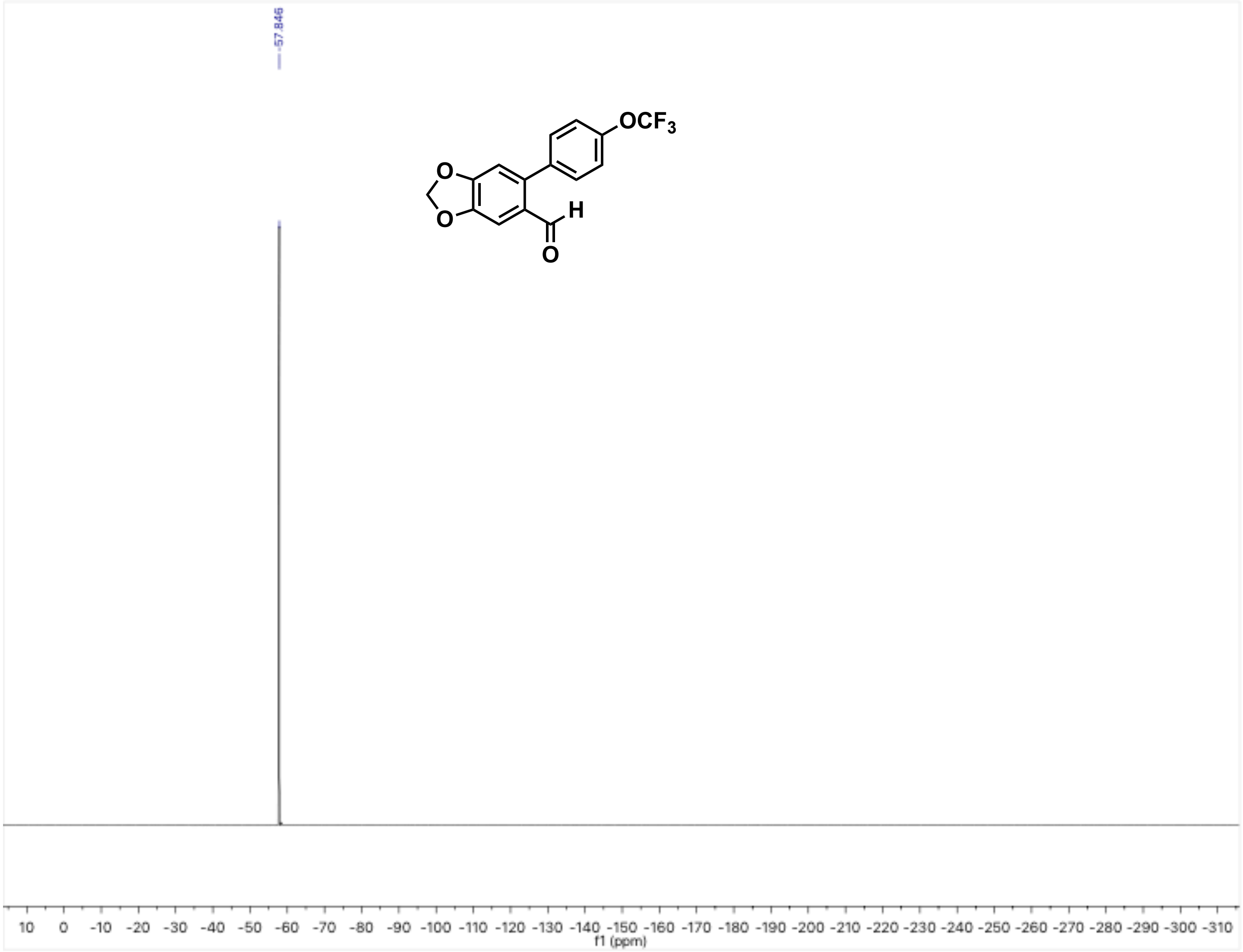
-57.846
OCF3
H
O
O
O
10 0 -10 -20 -30 -40 -50 -60 -70 -80 -90 -100 -110 -120 -130 -140 -150 -160 -170 -180 -190 -200 -210 -220 -230 -240 -250 -260 -270 -280 -290 -300 -310
f1 (ppm)

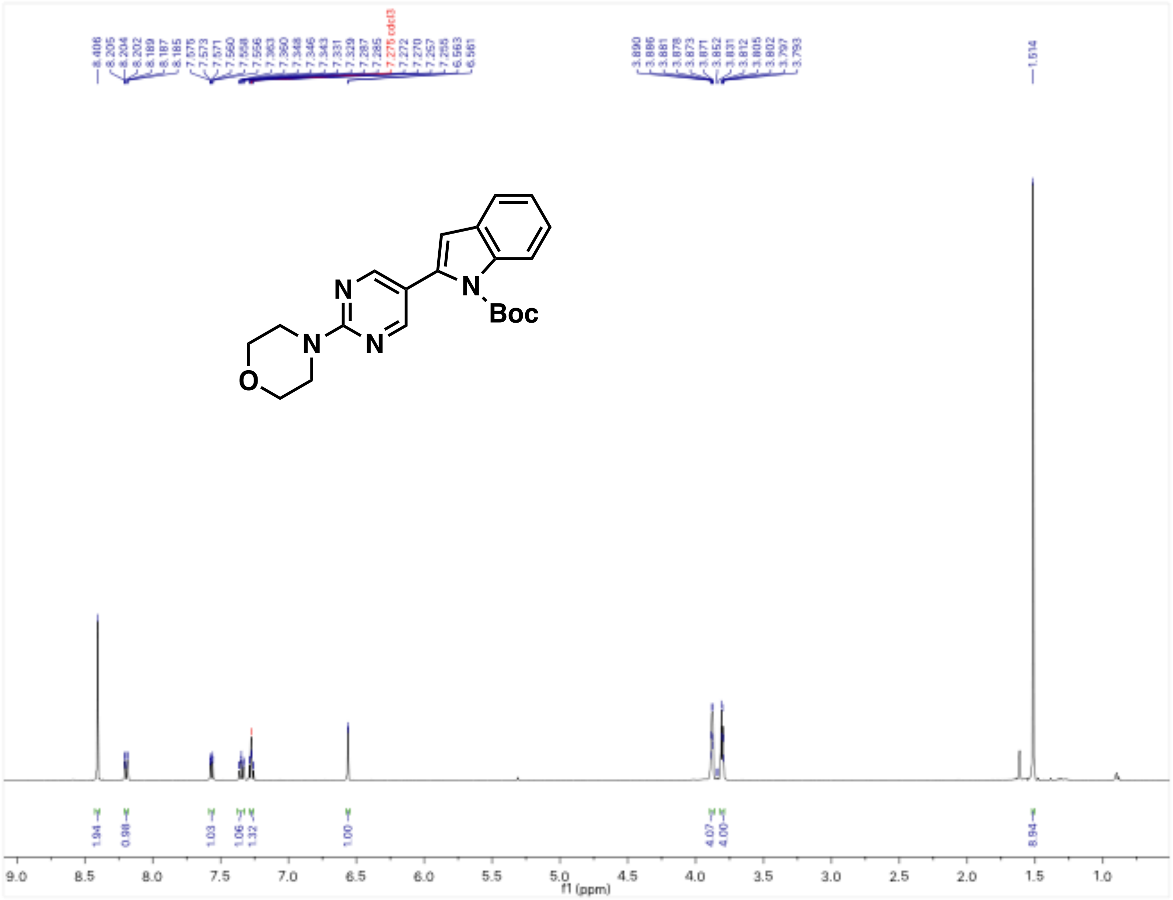
8.406
8.205
8.204
8.202
8.189
8.187
8.185
7.575
7.573
7.571
7.560
7.558
7.556
7.363
7.360
7.348
7.346
7.343
7.331
7.329
7.287
7.285
7.275 cdcl3
7.272
7.270
7.257
7.255
6.563
6.561
3.890
3.886
3.881
3.878
3.873
3.871
3.852
3.831
3.812
3.805
3.802
3.797
3.793
1.514
N
N
N
O
N
Boc
1.94
0.98
1.03
1.06
1.32
1.00
4.07
4.00
8.94
9.0 8.5 8.0 7.5 7.0 6.5 6.0 5.5 5.0 4.5 4.0 3.5 3.0 2.5 2.0 1.5 1.0
f1 (ppm)

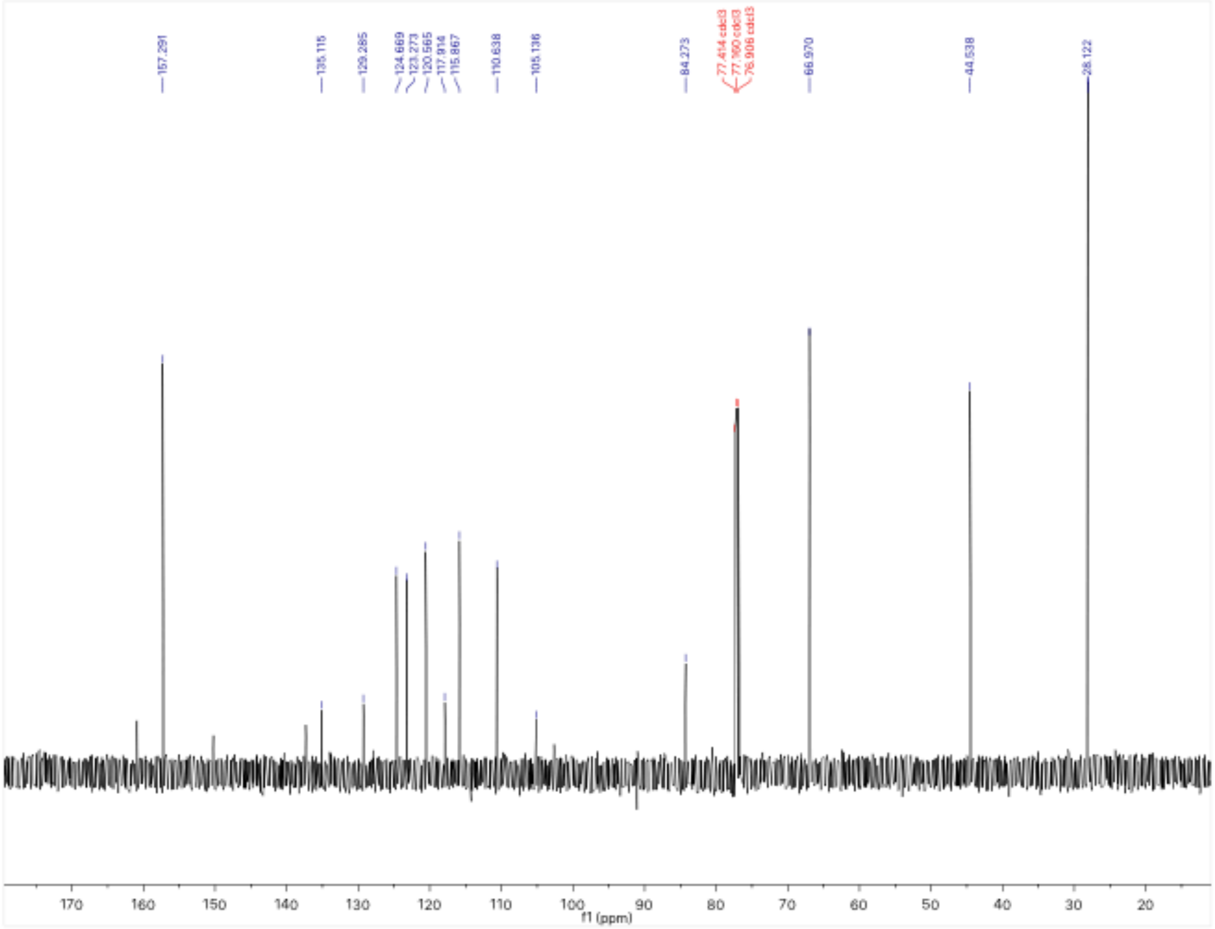
157.291
135.115
129.285
124.669
123.273
120.565
117.914
115.867
110.638
105.136
84.273
77.414 cdcl3
77.160 cdcl3
76.906 cdcl3
66.970
44.538
28.122
170 160 150 140 130 120 110 100 90 80 70 60 50 40 30 20
f1 (ppm)

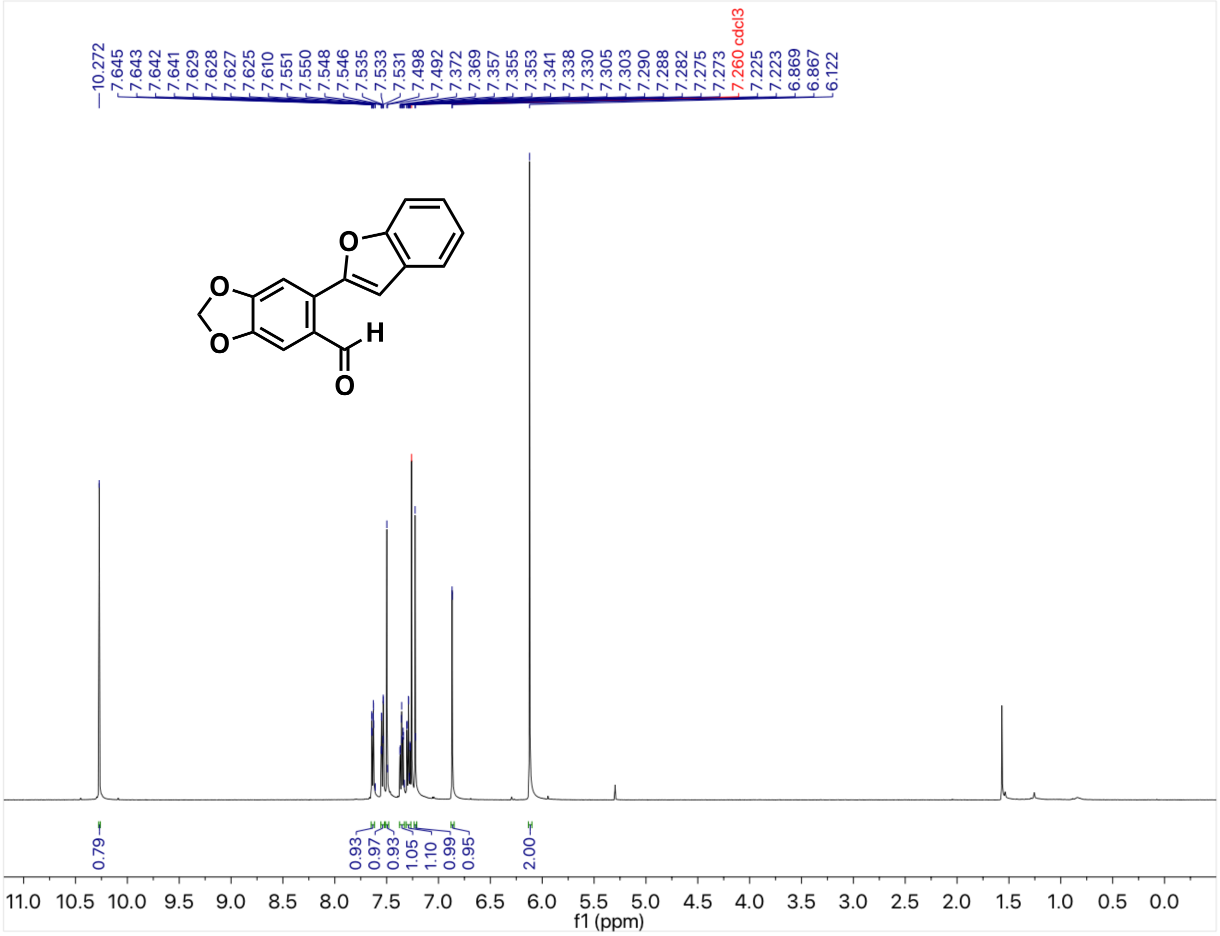
10.272
7.645
7.643
7.642
7.641
7.629
7.628
7.627
7.625
7.610
7.551
7.550
7.548
7.546
7.535
7.533
7.531
7.498
7.492
7.372
7.369
7.357
7.355
7.353
7.341
7.338
7.330
7.305
7.303
7.290
7.288
7.282
7.275
7.273
7.260 cdcl3
7.225
7.223
6.869
6.867
6.122
O
O
O
H
O
0.79
0.93
0.97
0.93
1.05
1.10
0.99
0.95
2.00
11.0 10.5 10.0 9.5 9.0 8.5 8.0 7.5 7.0 6.5 6.0 5.5 5.0 4.5 4.0 3.5 3.0 2.5 2.0 1.5 1.0 0.5 0.0
f1 (ppm)

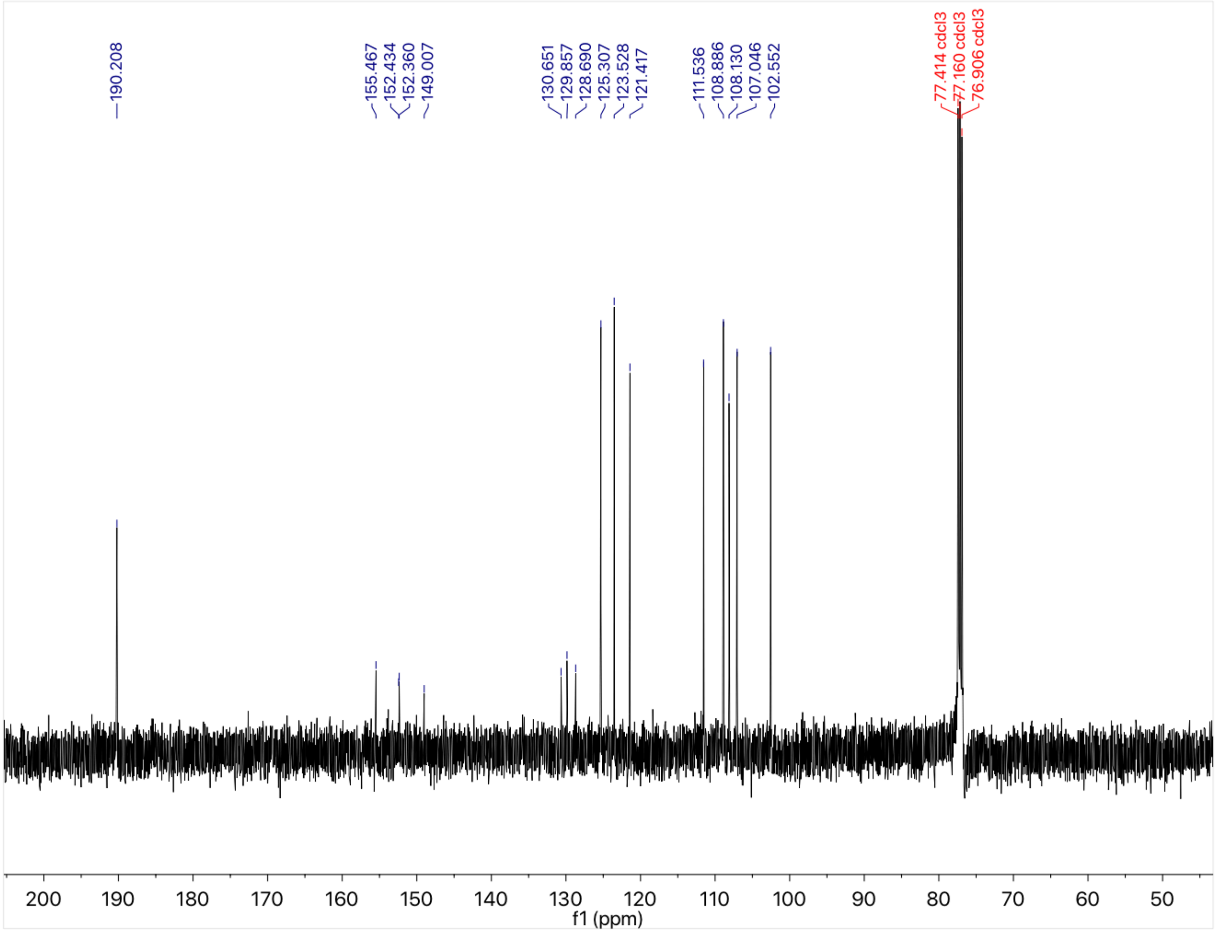
190.208
155.467
152.434
152.360
149.007
130.651
129.857
128.690
125.307
123.528
121.417
111.536
108.886
108.130
107.046
102.552
77.414 cdcl3
77.160 cdcl3
76.906 cdcl3
200 190 180 170 160 150 140 130 120 110 100 90 80 70 60 50
f1 (ppm)

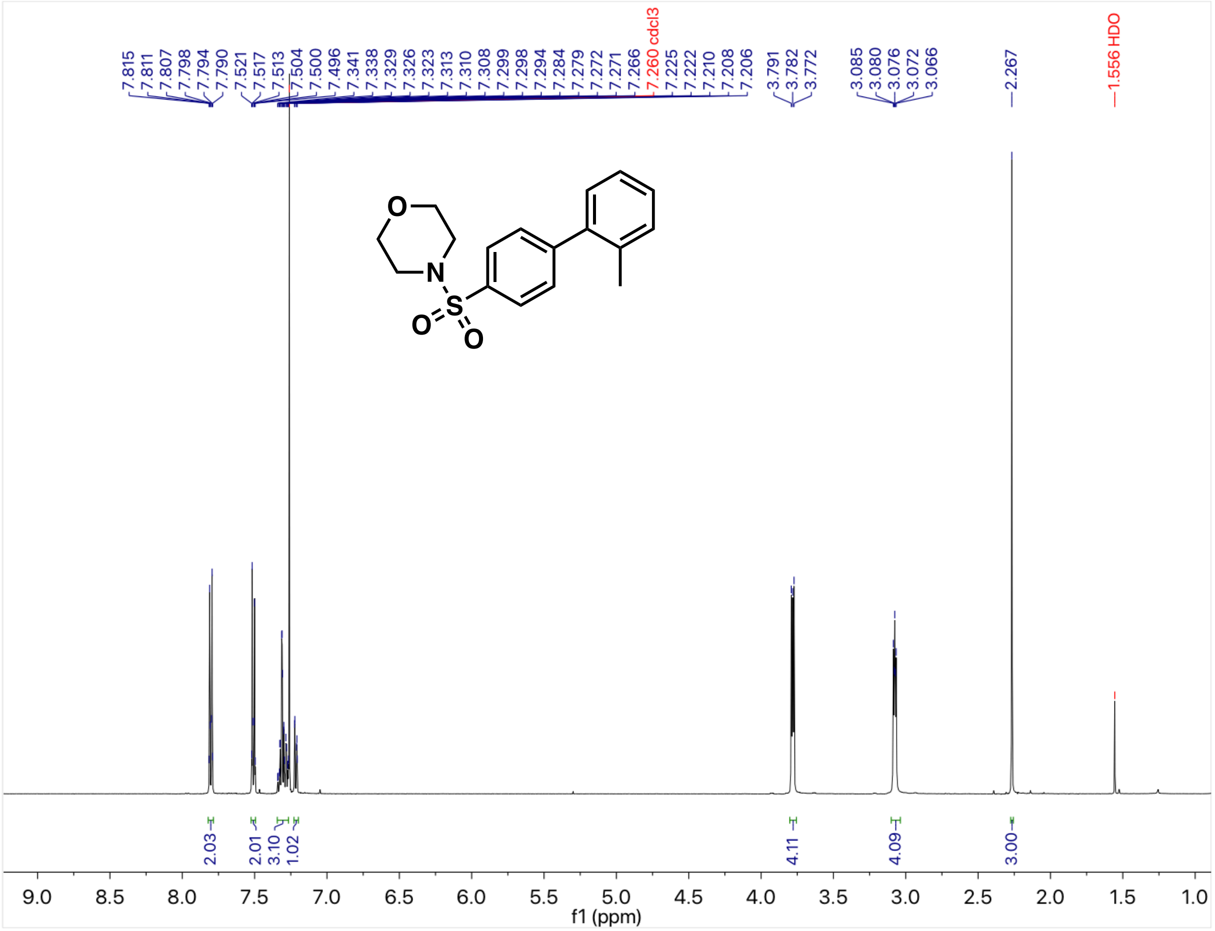
7.815 7.811 7.807 7.798 7.794 7.790 7.521 7.517 7.513 7.504 7.500 7.496 7.341 7.338 7.329 7.326 7.323 7.313 7.310 7.308 7.299 7.298 7.294 7.284 7.279 7.272 7.271 7.266
7.260 cdcl3
7.225 7.222 7.210 7.208 7.206
3.791 3.782 3.772
3.085 3.080 3.076 3.072 3.066
2.267
1.556 HDO
O
N
S
O
O
2.03
2.01
3.10
1.02
4.11
4.09
3.00
9.0 8.5 8.0 7.5 7.0 6.5 6.0 5.5 5.0 4.5 4.0 3.5 3.0 2.5 2.0 1.5 1.0
f1 (ppm)

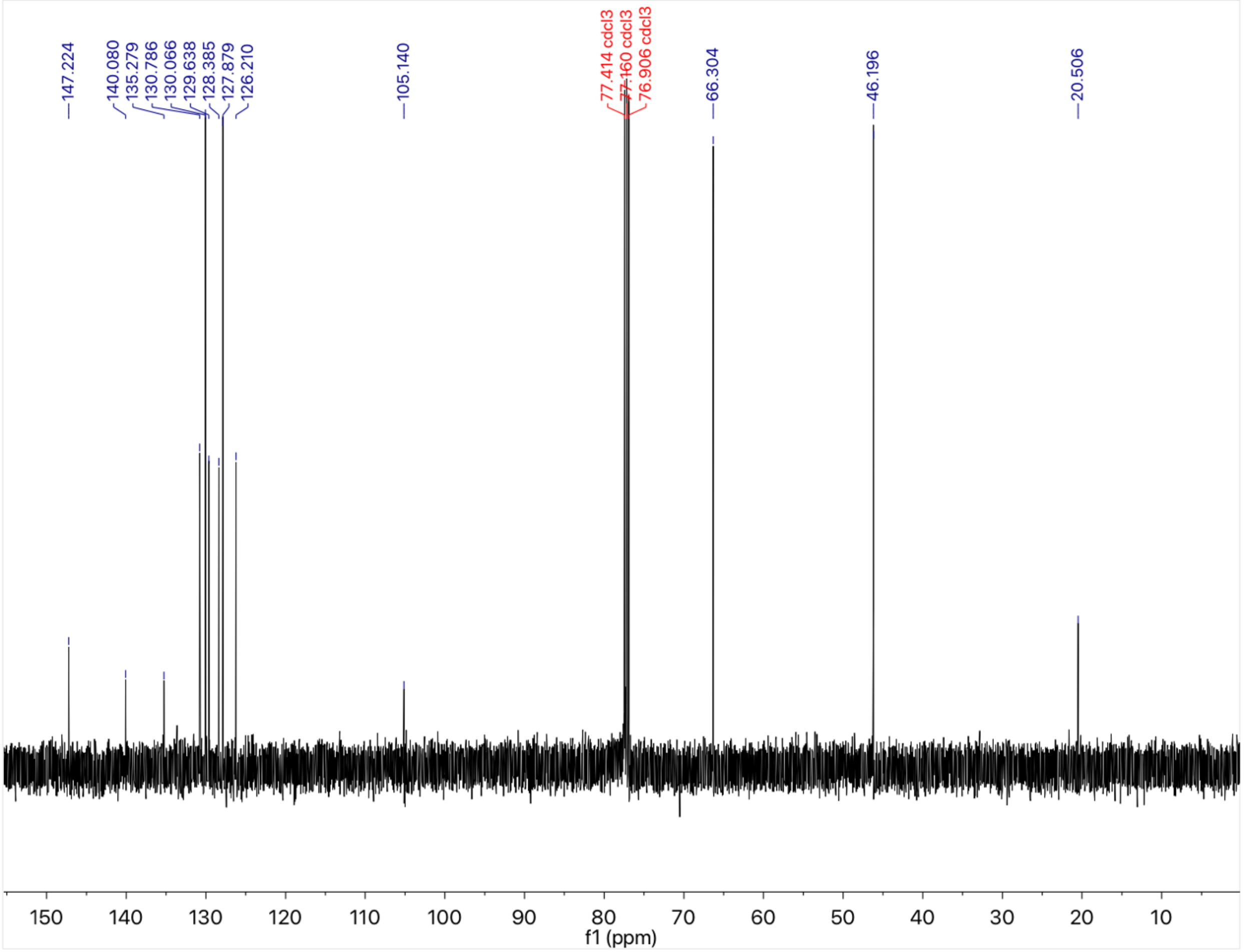
147.224
140.080 135.279 130.786 130.066 129.638 128.385 127.879 126.210
105.140
77.414 cdcl3
77.160 cdcl3
76.906 cdcl3
66.304
46.196
20.506
150 140 130 120 110 100 90 80 70 60 50 40 30 20 10
f1 (ppm)

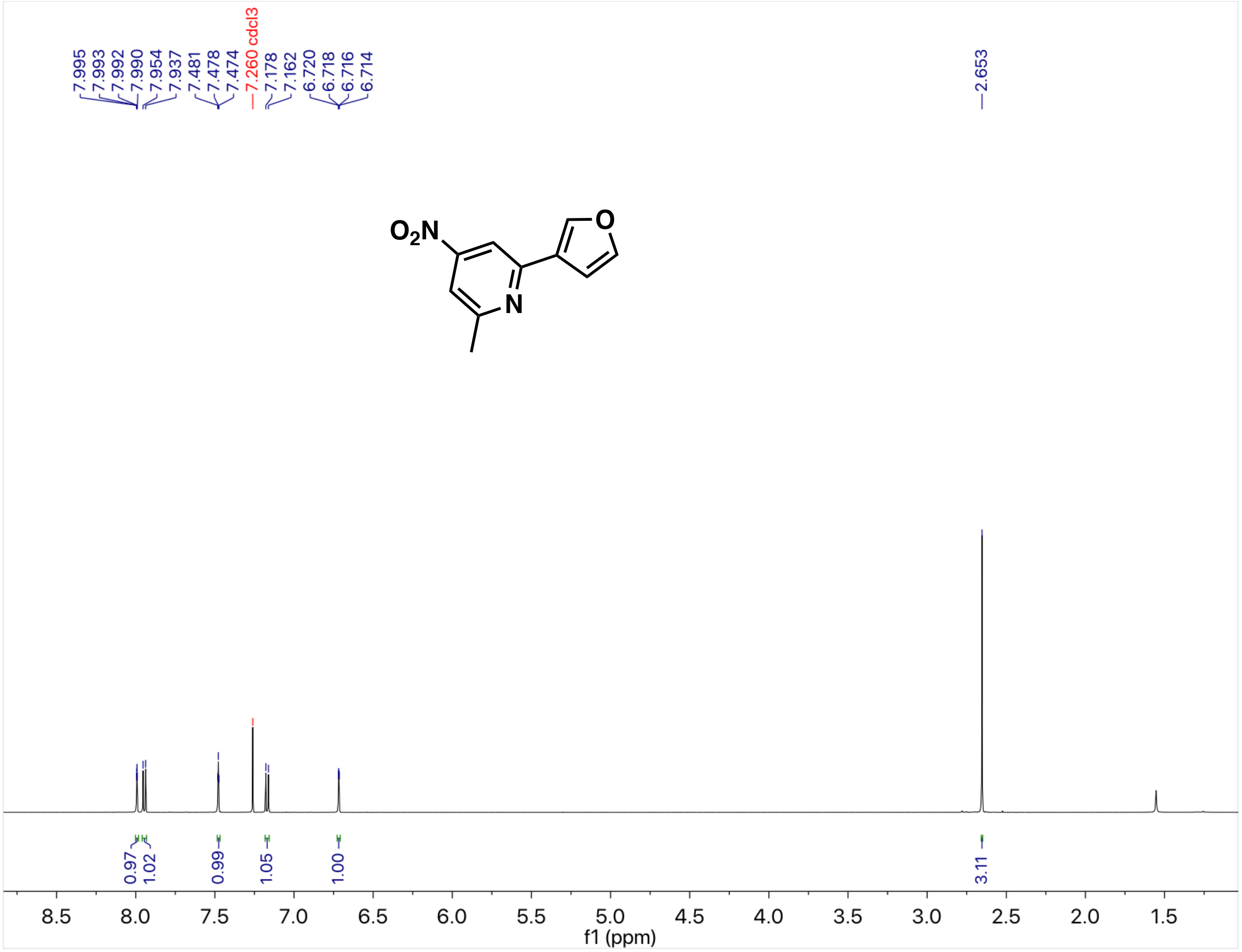
7.995
7.993
7.992
7.990
7.954
7.937
7.481
7.478
7.474
7.260 cdcl3
7.178
7.162
6.720
6.718
6.716
6.714
2.653
O_2N
N
O
0.97
1.02
0.99
1.05
1.00
3.11
8.5 8.0 7.5 7.0 6.5 6.0 5.5 5.0 4.5 4.0 3.5 3.0 2.5 2.0 1.5
f1 (ppm)

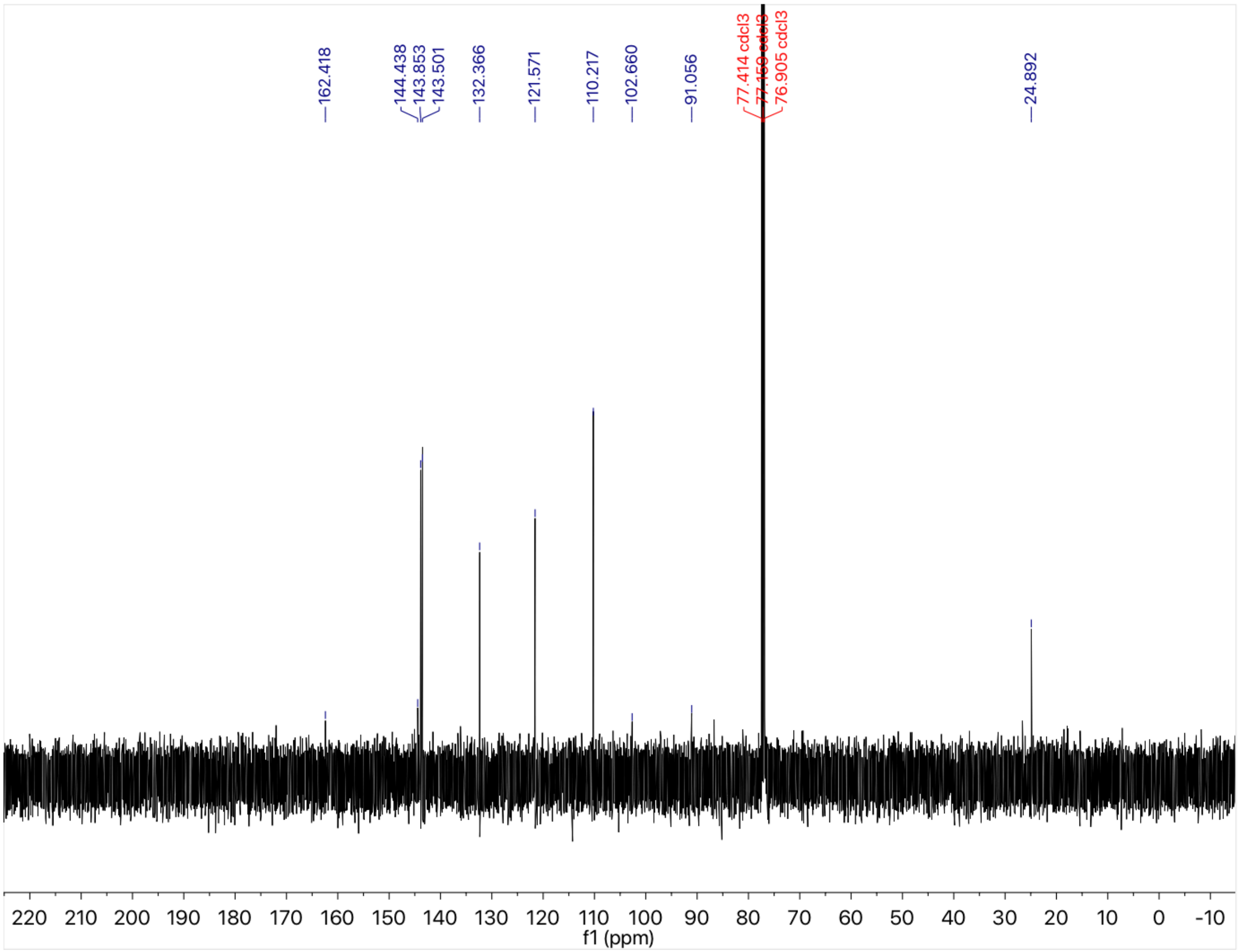
162.418
144.438
143.853
143.501
132.366
121.571
110.217
102.660
91.056
77.414 cdcl3
76.905 cdcl3
24.892
220 210 200 190 180 170 160 150 140 130 120 110 100 90 80 70 60 50 40 30 20 10 0 -10
f1 (ppm)

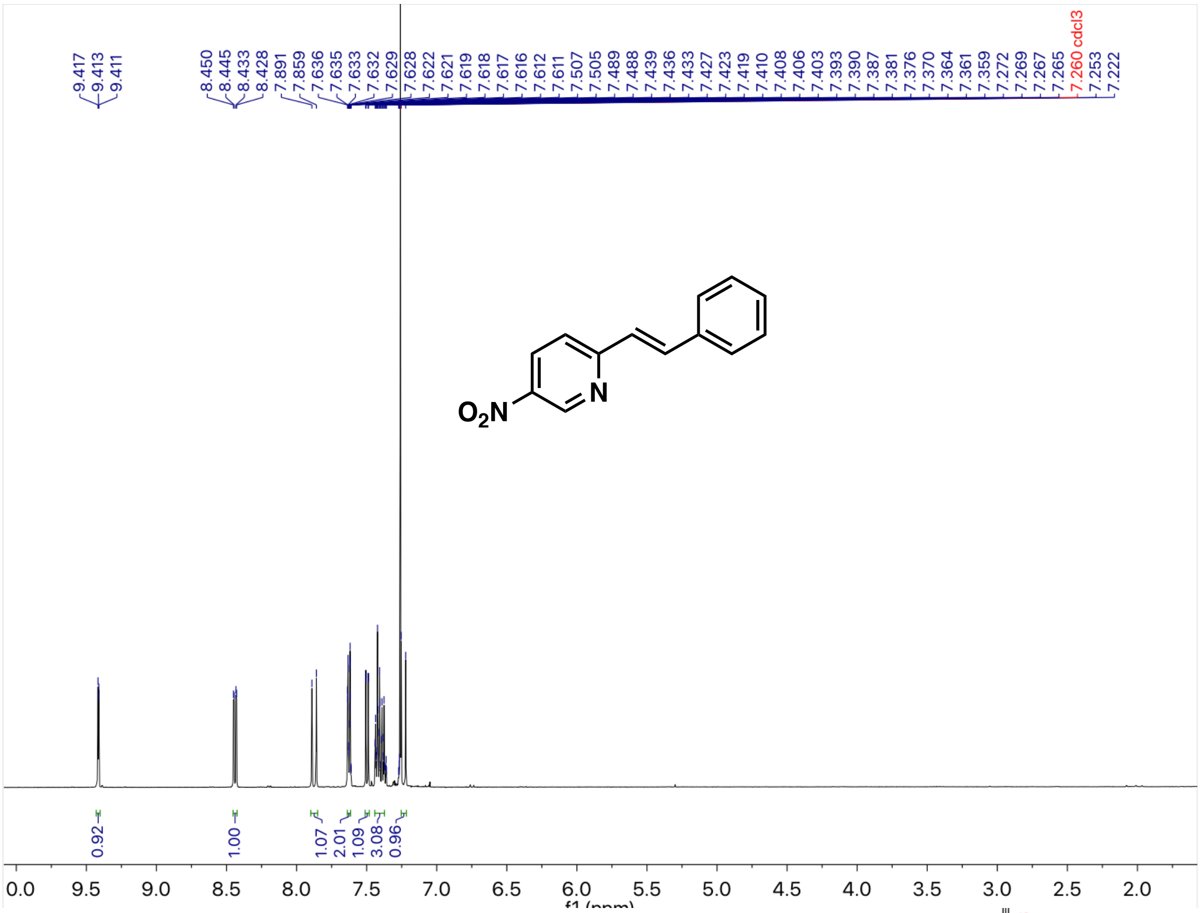
9.417
9.413
9.411
8.450
8.445
8.433
8.428
7.891
7.859
7.636
7.635
7.633
7.632
7.629
7.628
7.622
7.621
7.619
7.618
7.617
7.616
7.612
7.611
7.507
7.505
7.489
7.488
7.439
7.436
7.433
7.427
7.423
7.419
7.410
7.408
7.406
7.403
7.393
7.390
7.387
7.381
7.376
7.370
7.364
7.361
7.359
7.272
7.269
7.267
7.265
7.260 cdcl3
7.253
7.222
O2N
N
0.92
1.00
1.07
2.01
1.09
3.08
0.96
9.5
9.0
8.5
8.0
7.5
7.0
6.5
6.0
5.5
5.0
4.5
4.0
3.5
3.0
2.5
2.0

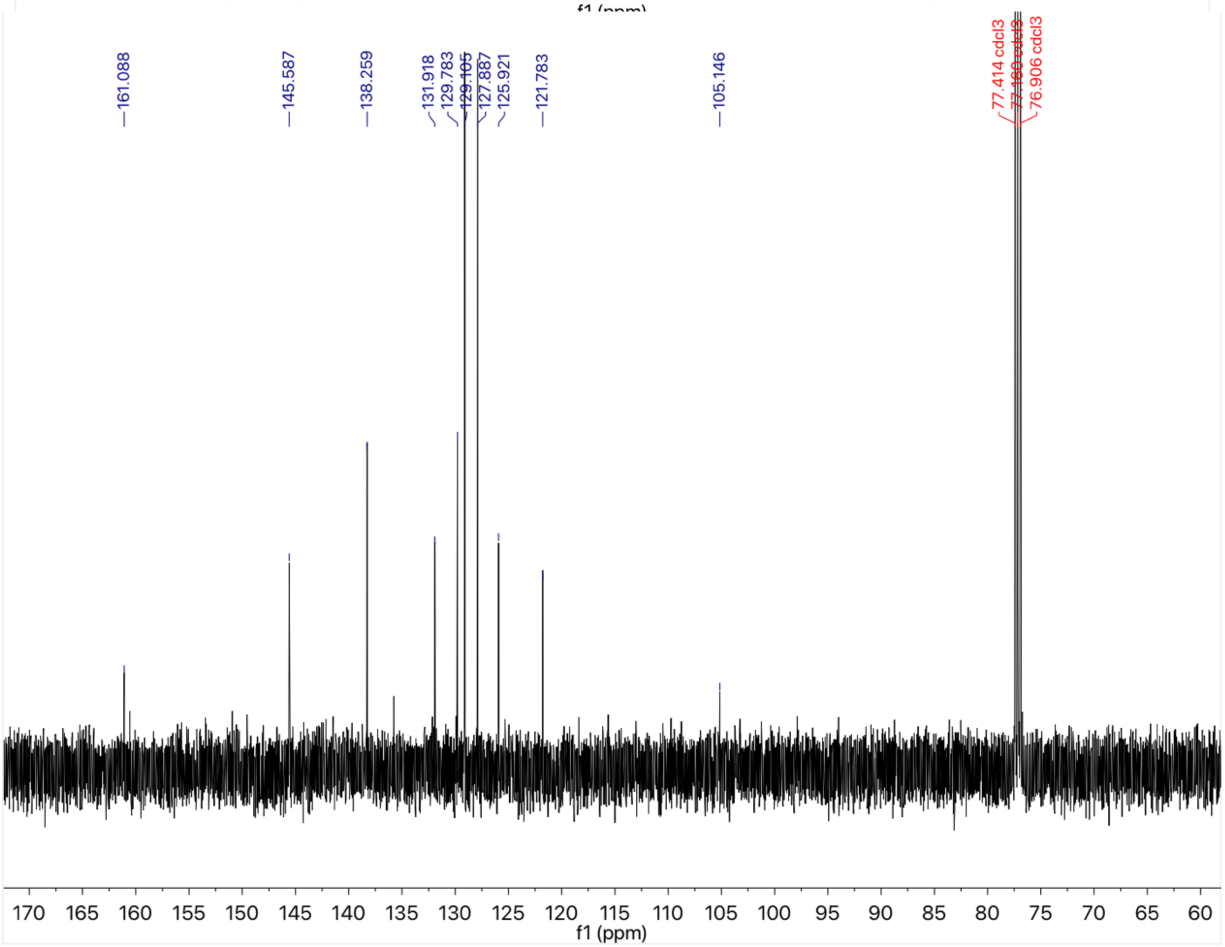
161.088
145.587
138.259
131.918
129.783
129.105
127.887
125.921
121.783
105.146
77.414 cdcl3
77.160 cdcl3
76.906 cdcl3
170
165
160
155
150
145
140
135
130
125
120
115
110
105
100
95
90
85
80
75
70
65
60
f1 (ppm)

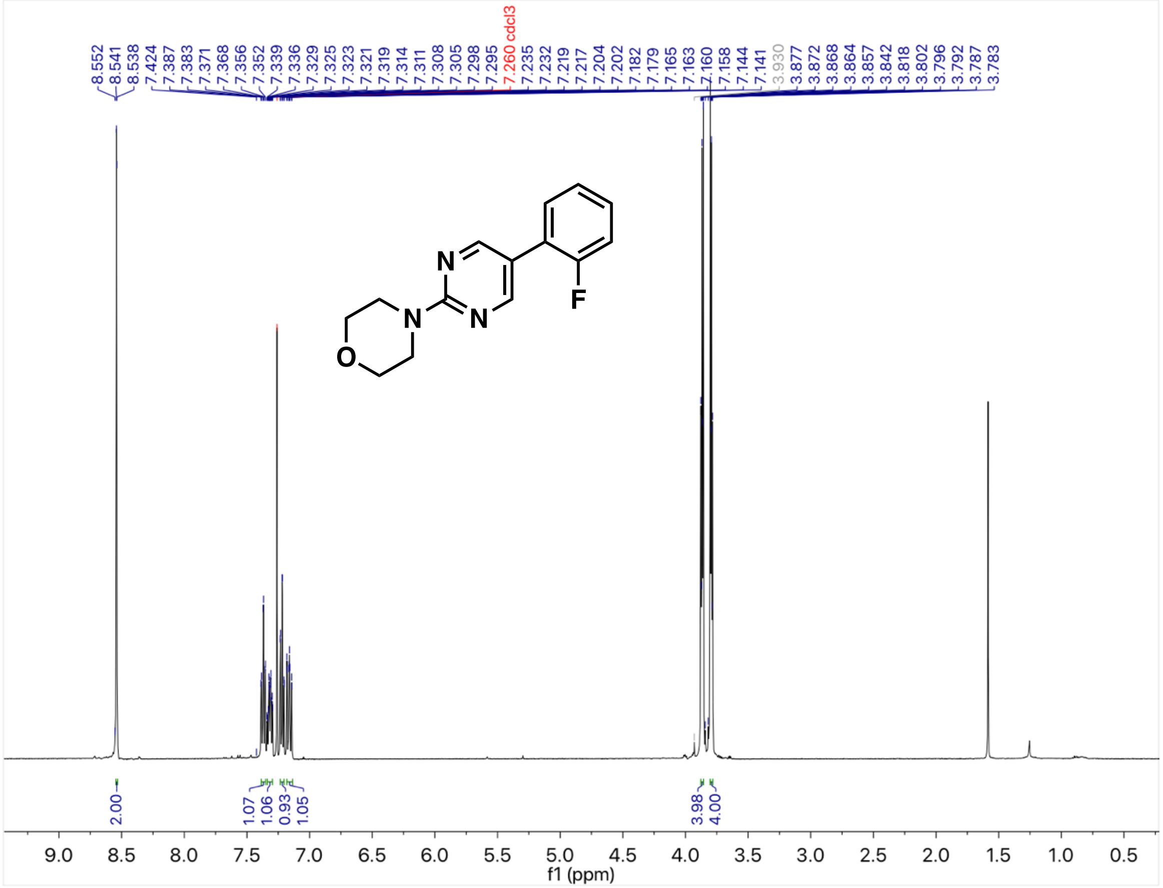
8.552
8.541
8.538
7.424
7.387
7.383
7.371
7.368
7.356
7.352
7.339
7.336
7.329
7.325
7.323
7.321
7.319
7.314
7.311
7.308
7.305
7.298
7.295
7.260 cdcl3
7.235
7.232
7.219
7.217
7.204
7.202
7.182
7.179
7.165
7.163
7.160
7.158
7.144
7.141
3.930
3.877
3.872
3.868
3.864
3.857
3.842
3.818
3.802
3.796
3.792
3.787
3.783
N
N
N
O
F
2.00
1.07
1.06
0.93
1.05
3.98
4.00
9.0 8.5 8.0 7.5 7.0 6.5 6.0 5.5 5.0 4.5 4.0 3.5 3.0 2.5 2.0 1.5 1.0 0.5
f1 (ppm)

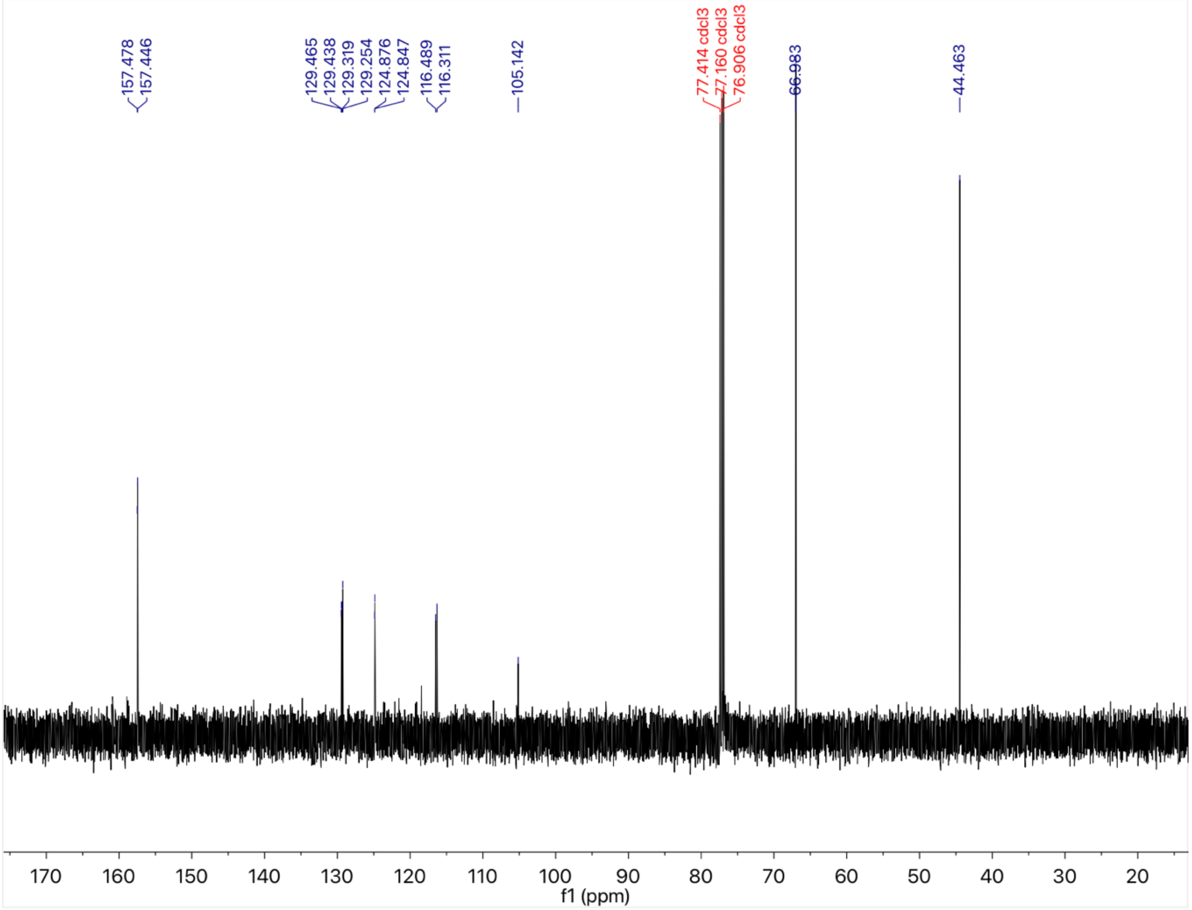
157.478
157.446
129.465
129.438
129.319
129.254
124.876
124.847
116.489
116.311
105.142
77.414 cdcl3
77.160 cdcl3
76.906 cdcl3
66.983
44.463
170 160 150 140 130 120 110 100 90 80 70 60 50 40 30 20
f1 (ppm)

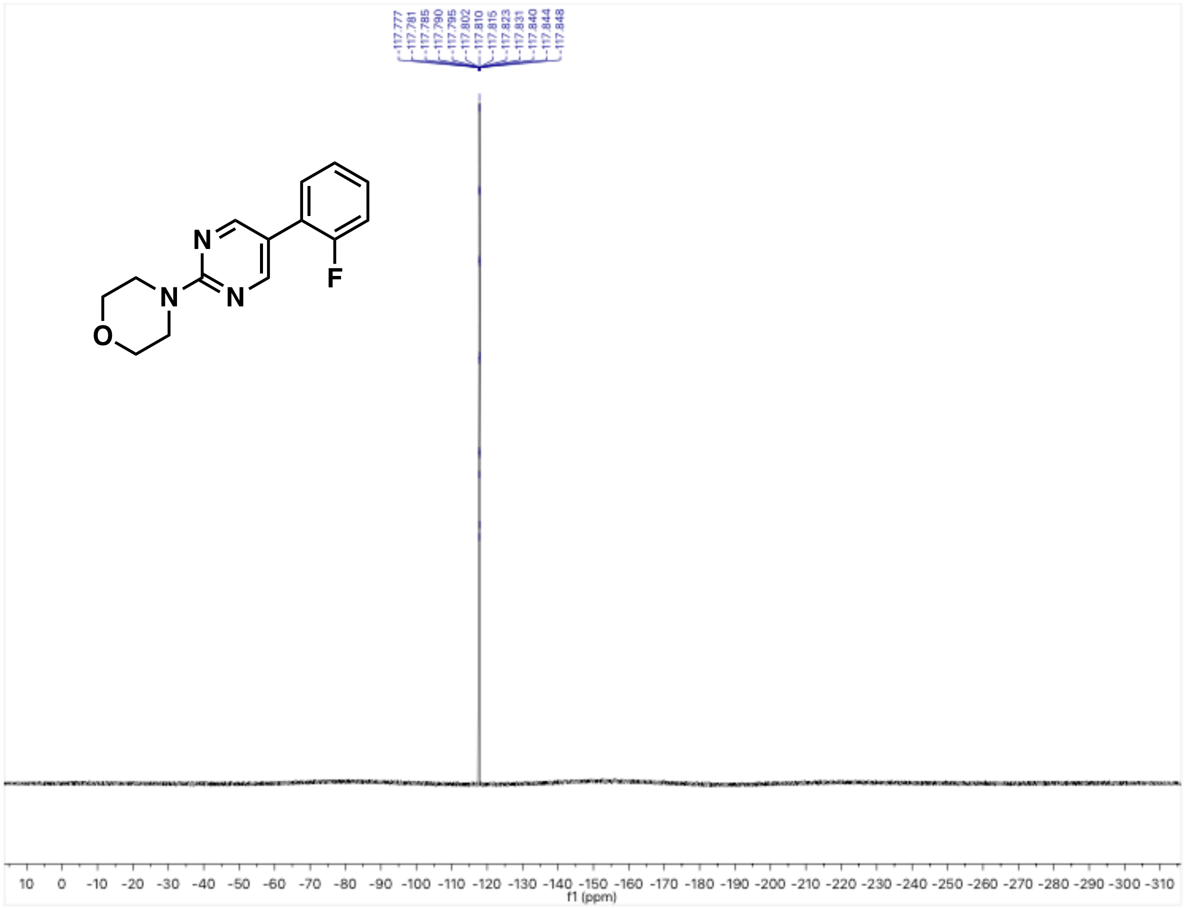

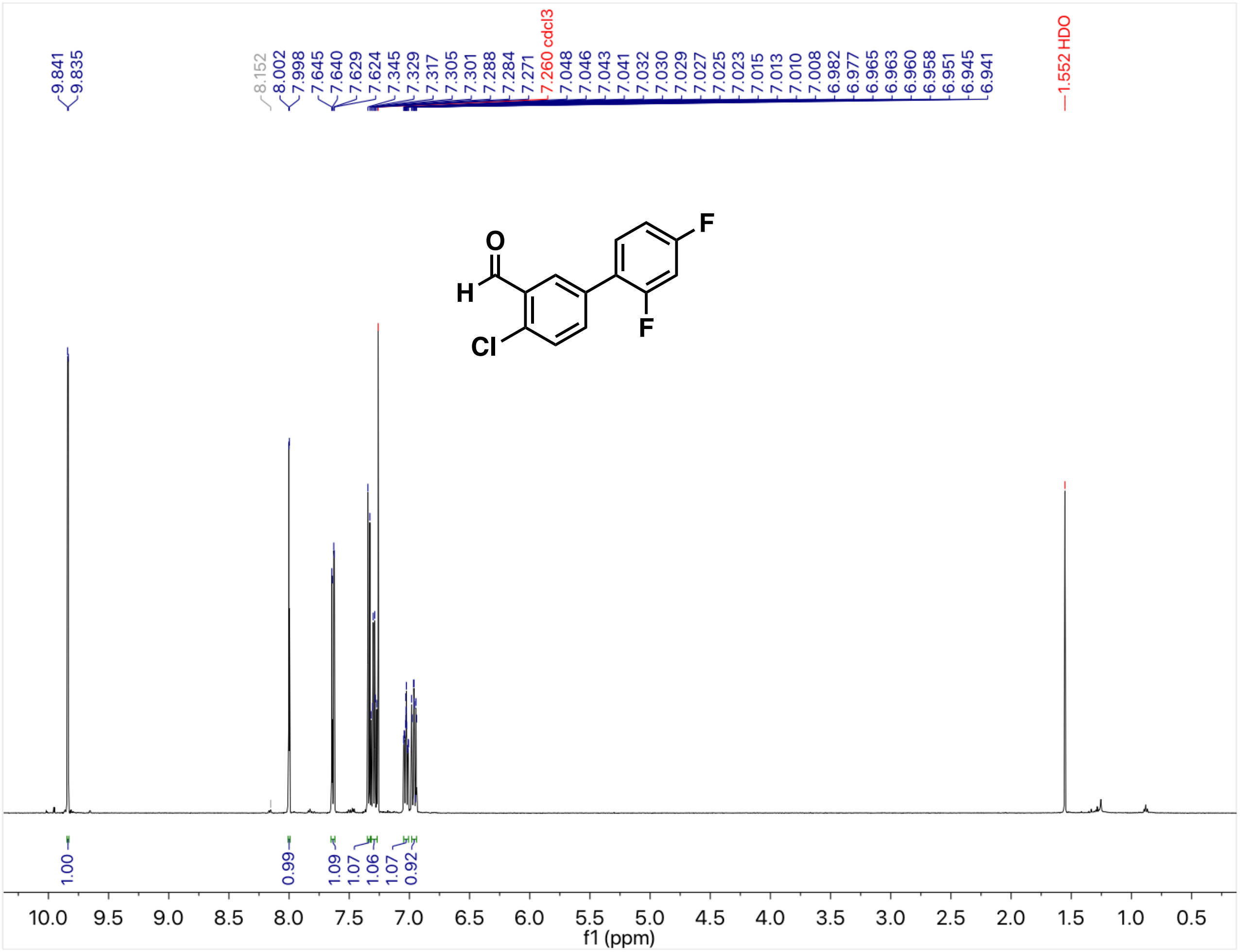
9.841
9.835
8.152
8.002
7.998
7.645
7.640
7.629
7.624
7.345
7.329
7.317
7.305
7.301
7.288
7.284
7.271
7.260 cdcl3
7.048
7.046
7.043
7.041
7.032
7.030
7.029
7.027
7.025
7.023
7.015
7.013
7.010
7.008
6.982
6.977
6.965
6.963
6.960
6.958
6.951
6.945
6.941
1.552 HDO
O
H
Cl
F
F
1.00
0.99
1.09
1.07
1.06
1.07
0.92
10.0 9.5 9.0 8.5 8.0 7.5 7.0 6.5 6.0 5.5 5.0 4.5 4.0 3.5 3.0 2.5 2.0 1.5 1.0 0.5
f1 (ppm)

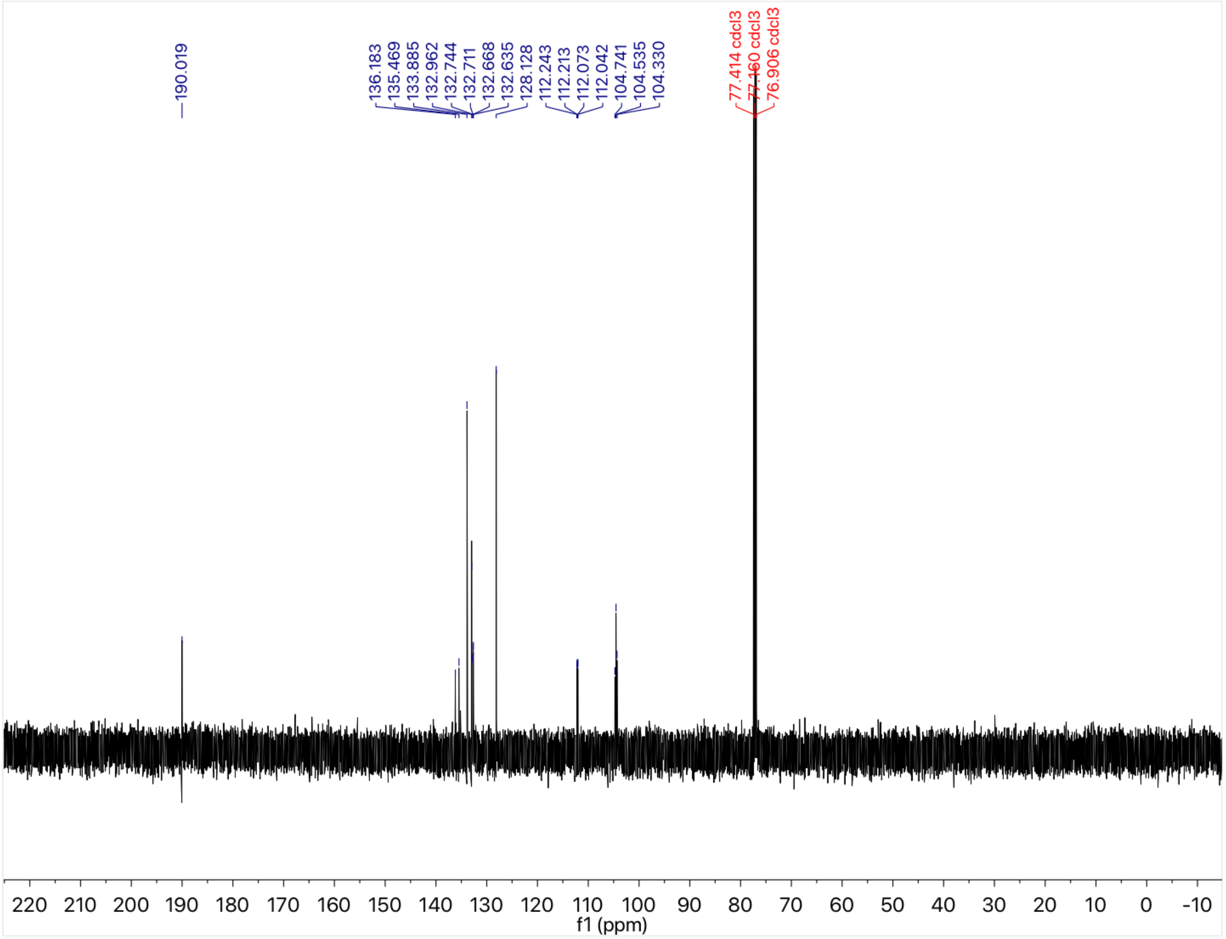
190.019
136.183
135.469
133.885
132.962
132.744
132.711
132.668
132.635
128.128
112.243
112.213
112.073
112.042
104.741
104.535
104.330
77.414 cdcl3
77.160 cdcl3
76.906 cdcl3
220 210 200 190 180 170 160 150 140 130 120 110 100 90 80 70 60 50 40 30 20 10 0 -10
f1 (ppm)

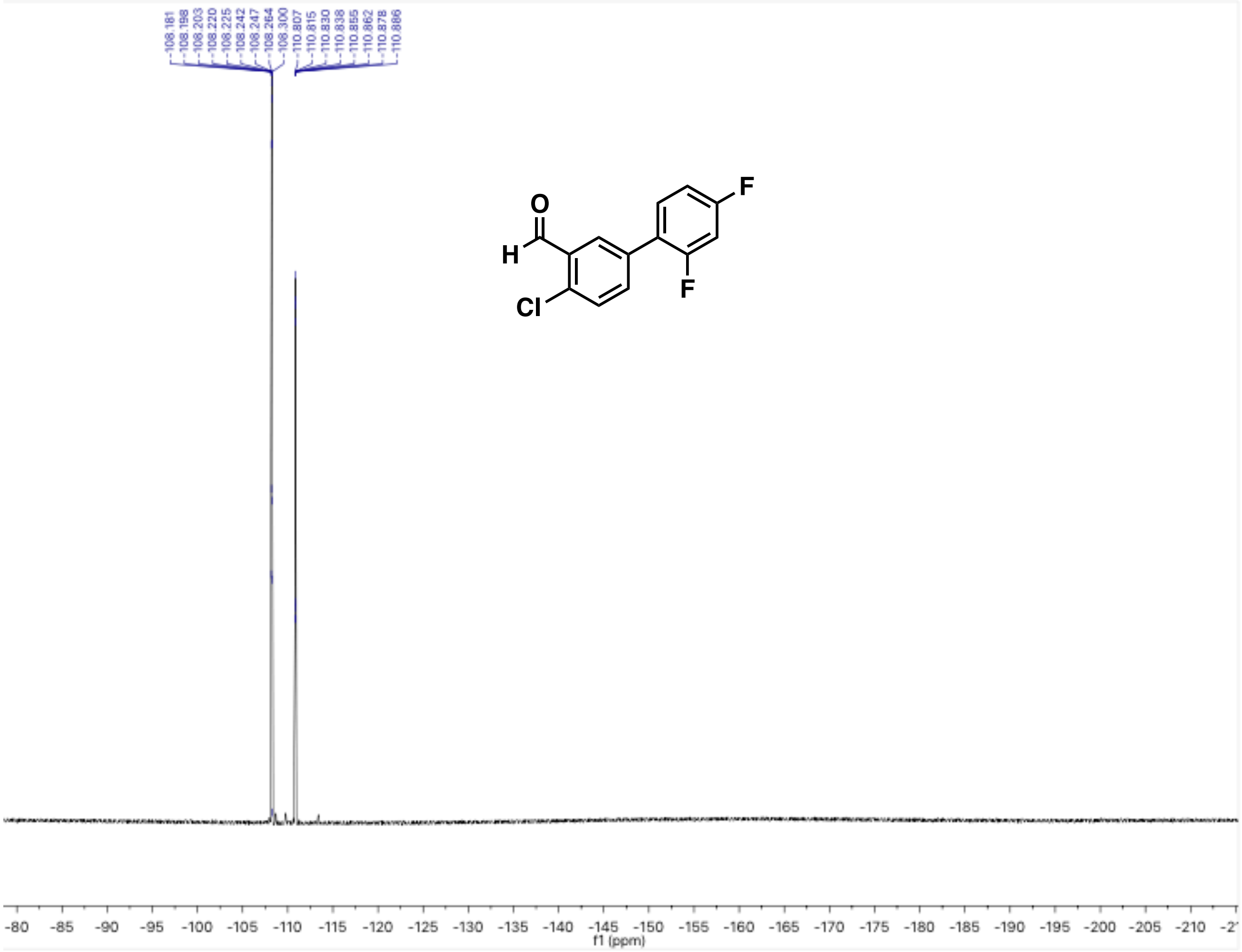

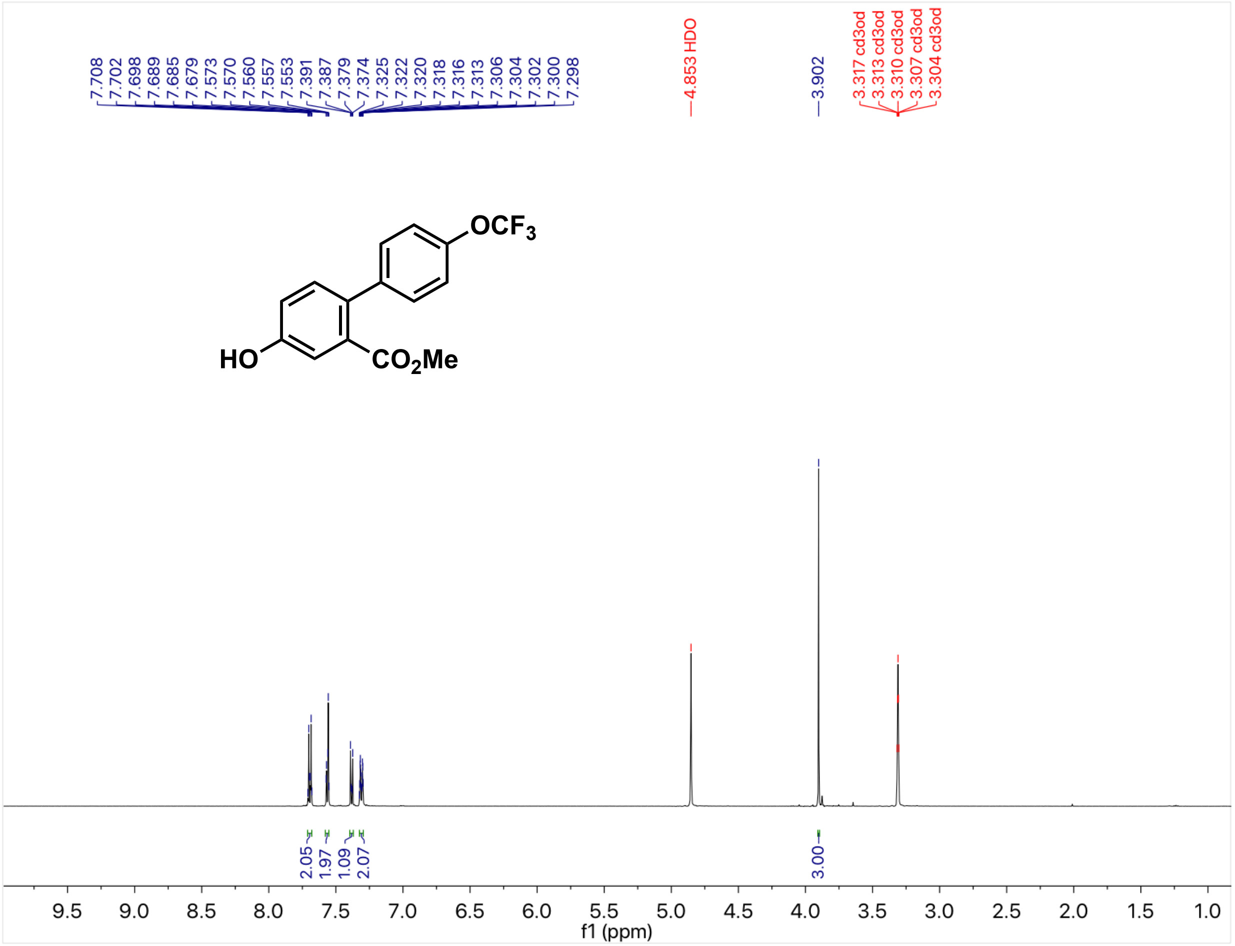
7.708
7.702
7.698
7.689
7.685
7.679
7.573
7.570
7.560
7.557
7.553
7.391
7.387
7.379
7.374
7.325
7.322
7.320
7.318
7.316
7.313
7.306
7.304
7.302
7.300
7.298
4.853 HDO
3.902
3.317 cd3od
3.313 cd3od
3.310 cd3od
3.307 cd3od
3.304 cd3od
OCF3
HO
CO2Me
2.05
1.97
1.09
2.07
3.00
9.5
9.0
8.5
8.0
7.5
7.0
6.5
6.0
5.5
5.0
4.5
4.0
3.5
3.0
2.5
2.0
1.5
1.0
f1 (ppm)

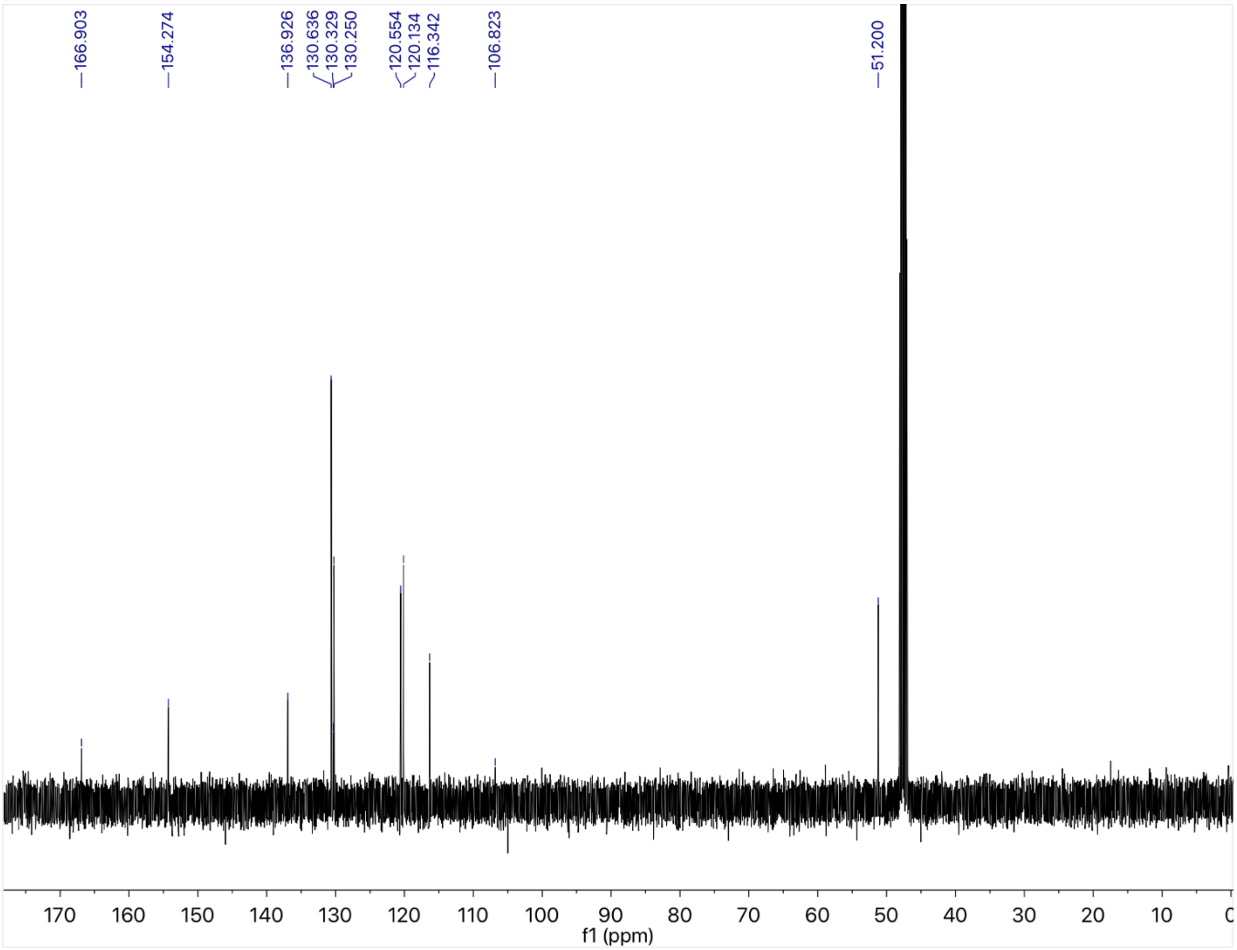
166.903
154.274
136.926
130.636
130.329
130.250
120.554
120.134
116.342
106.823
51.200
170
160
150
140
130
120
110
100
90
80
70
60
50
40
30
20
10
f1 (ppm)

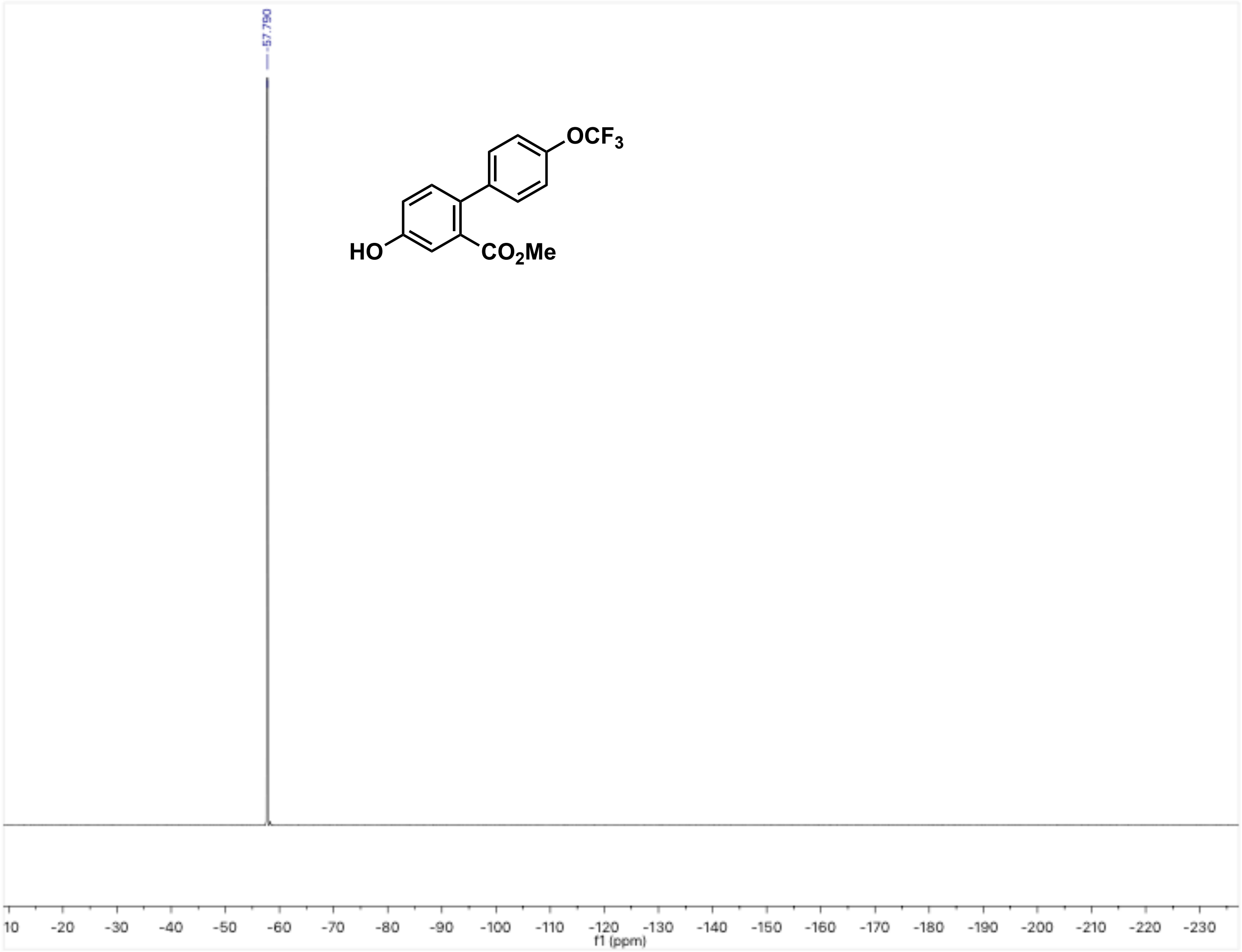

-57.790
OCF3
HO
CO2Me
10 -20 -30 -40 -50 -60 -70 -80 -90 -100 -110 -120 -130 -140 -150 -160 -170 -180 -190 -200 -210 -220 -230
f1 (ppm)

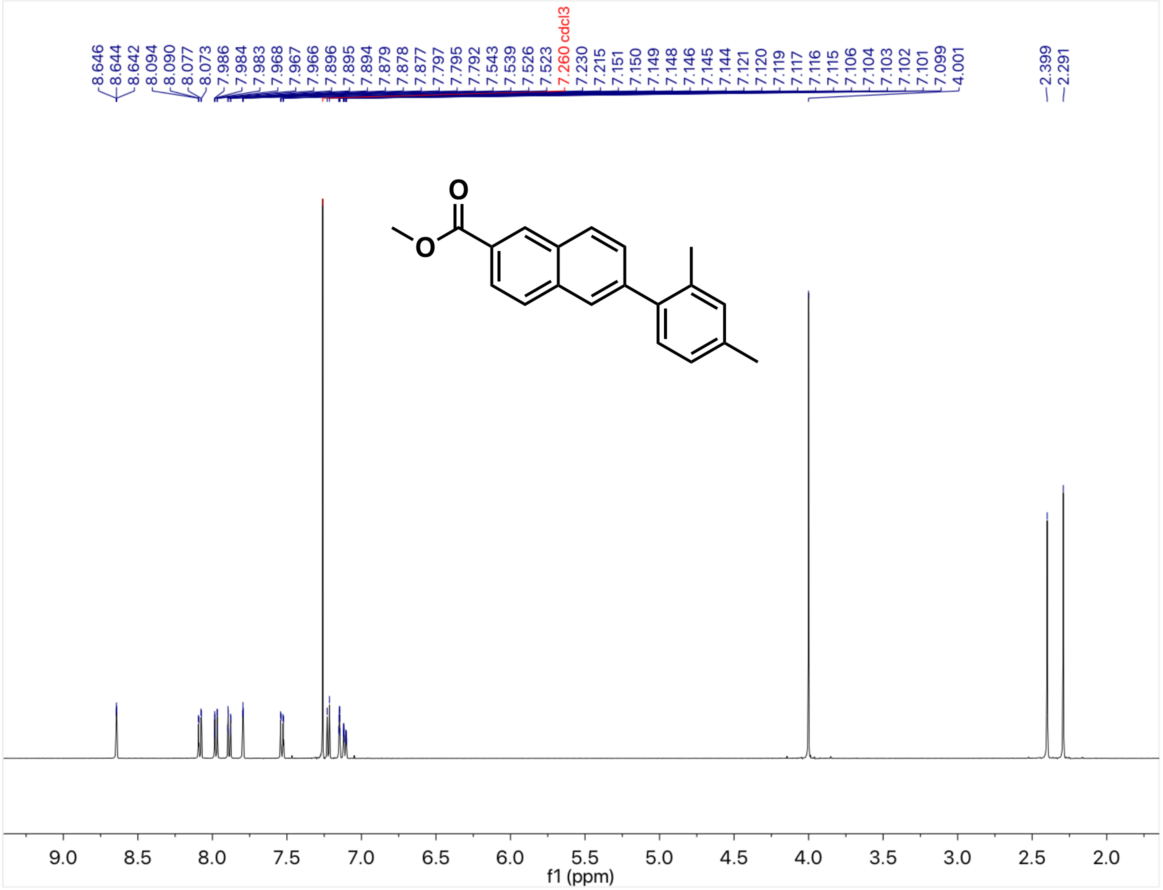
8.646
8.644
8.642
8.094
8.090
8.077
8.073
7.986
7.984
7.983
7.968
7.967
7.966
7.896
7.895
7.894
7.879
7.878
7.877
7.797
7.795
7.792
7.543
7.539
7.526
7.523
7.260 cdcl3
7.230
7.215
7.151
7.150
7.149
7.148
7.146
7.145
7.144
7.121
7.120
7.119
7.117
7.116
7.115
7.106
7.104
7.103
7.102
7.101
7.099
4.001
2.399
2.291
O
O
9.0 8.5 8.0 7.5 7.0 6.5 6.0 5.5 5.0 4.5 4.0 3.5 3.0 2.5 2.0
f1 (ppm)

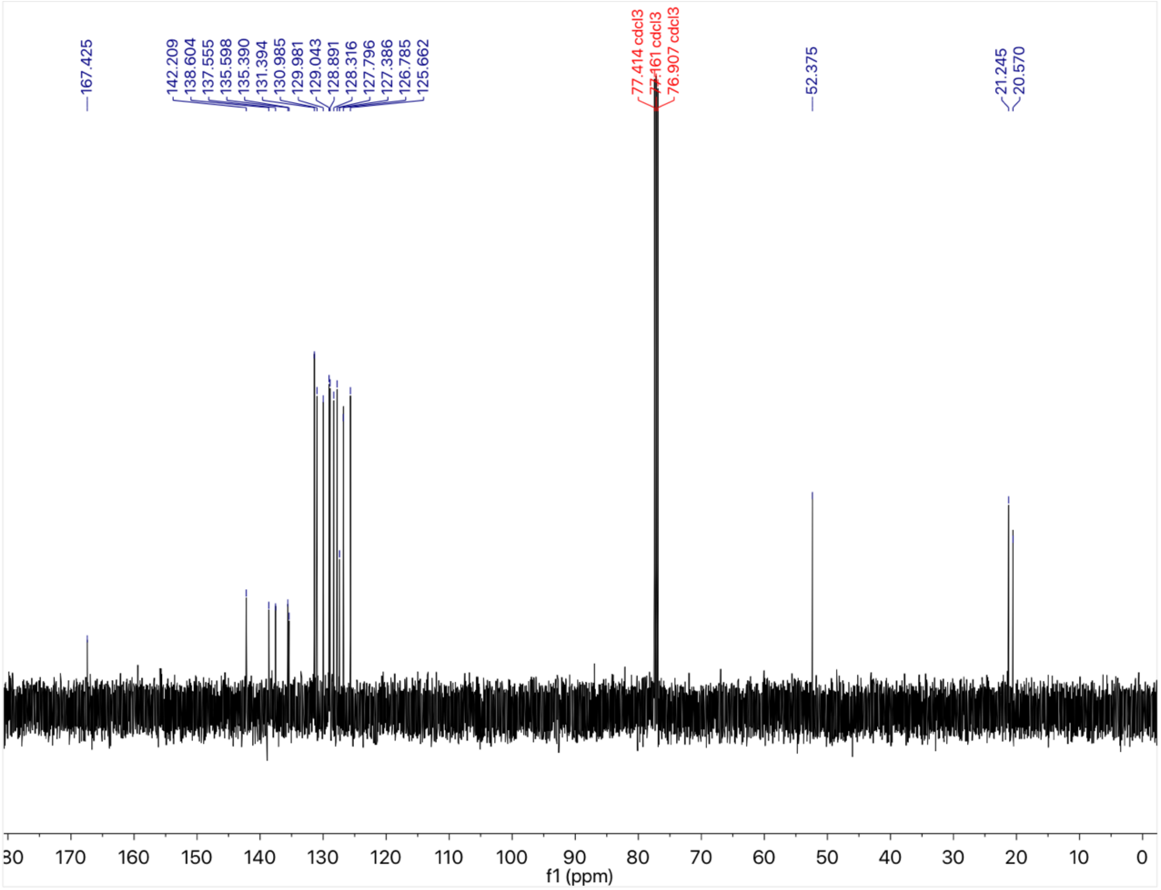
167.425
142.209
138.604
137.555
135.598
135.390
131.394
130.985
129.981
129.043
128.891
128.316
127.796
127.386
126.785
125.662
77.414 cdcl3
77.161 cdcl3
76.907 cdcl3
52.375
21.245
20.570
30 170 160 150 140 130 120 110 100 90 80 70 60 50 40 30 20 10 0
f1 (ppm)

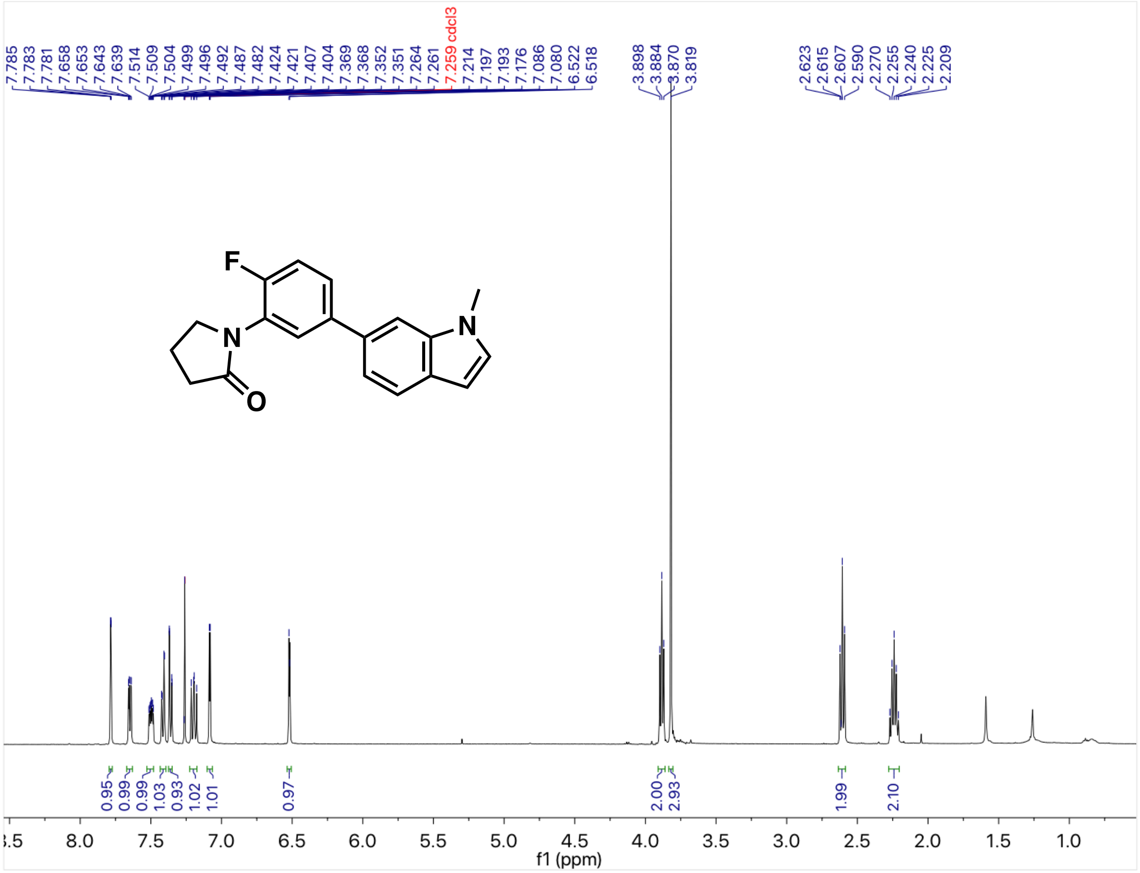
7.785 7.783 7.781 7.658 7.653 7.643 7.639 7.514 7.509 7.504 7.499 7.496 7.492 7.487 7.482 7.424 7.421 7.407 7.404 7.369 7.368 7.352 7.351 7.264 7.261 7.259 cdcl3 7.214 7.197 7.193 7.176 7.086 7.080 6.522 6.518
3.898 3.884 3.870 3.819
2.623 2.615 2.607 2.590 2.270 2.255 2.240 2.225 2.209
F
N
O
N
0.95 0.99 0.99 1.03 0.93 1.02 1.01 0.97 2.00 2.93 1.99 2.10
8.0 7.5 7.0 6.5 6.0 5.5 5.0 4.5 4.0 3.5 3.0 2.5 2.0 1.5 1.0
f1 (ppm)

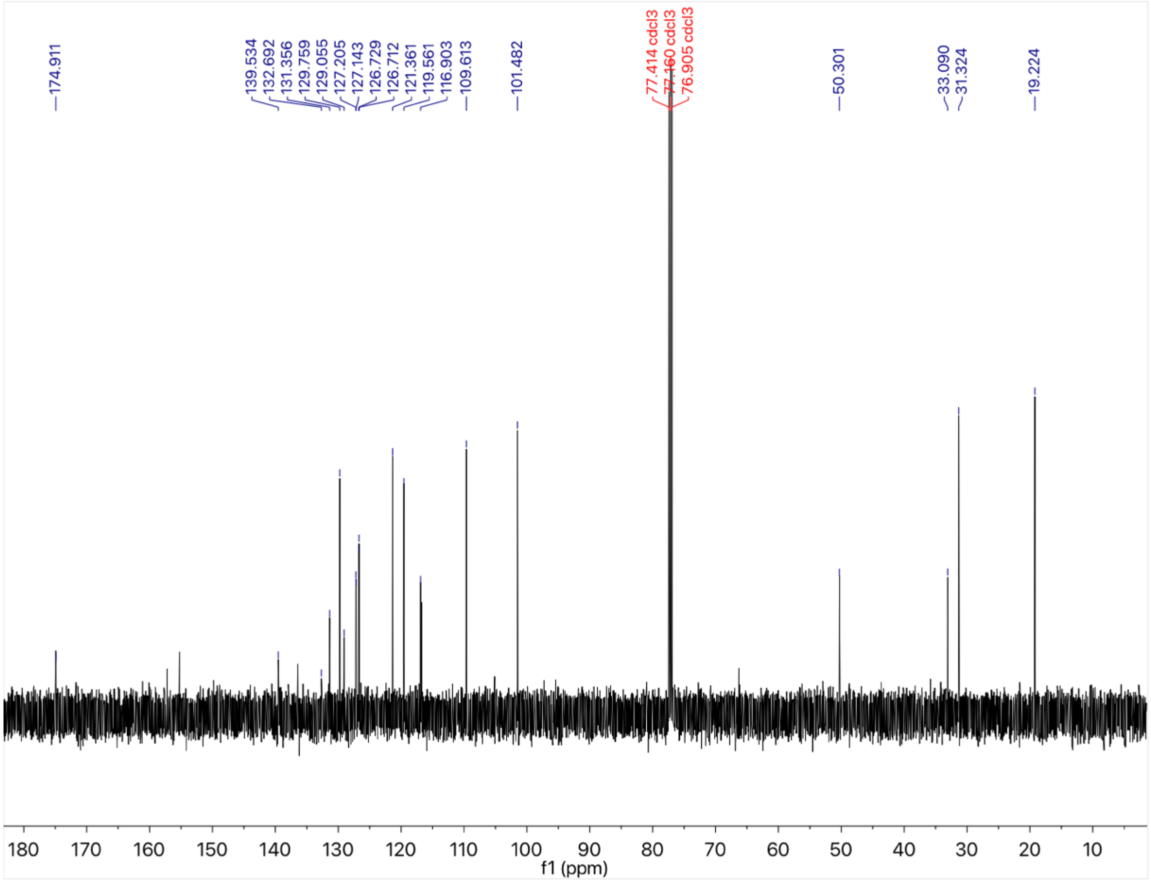
174.911
139.534 132.692 131.356 129.759 129.055 127.205 127.143 126.729 126.712 121.361 119.561 116.903 109.613
101.482
77.414 cdcl3 77.160 cdcl3 76.905 cdcl3
50.301
33.090 31.324
19.224
180 170 160 150 140 130 120 110 100 90 80 70 60 50 40 30 20 10
f1 (ppm)

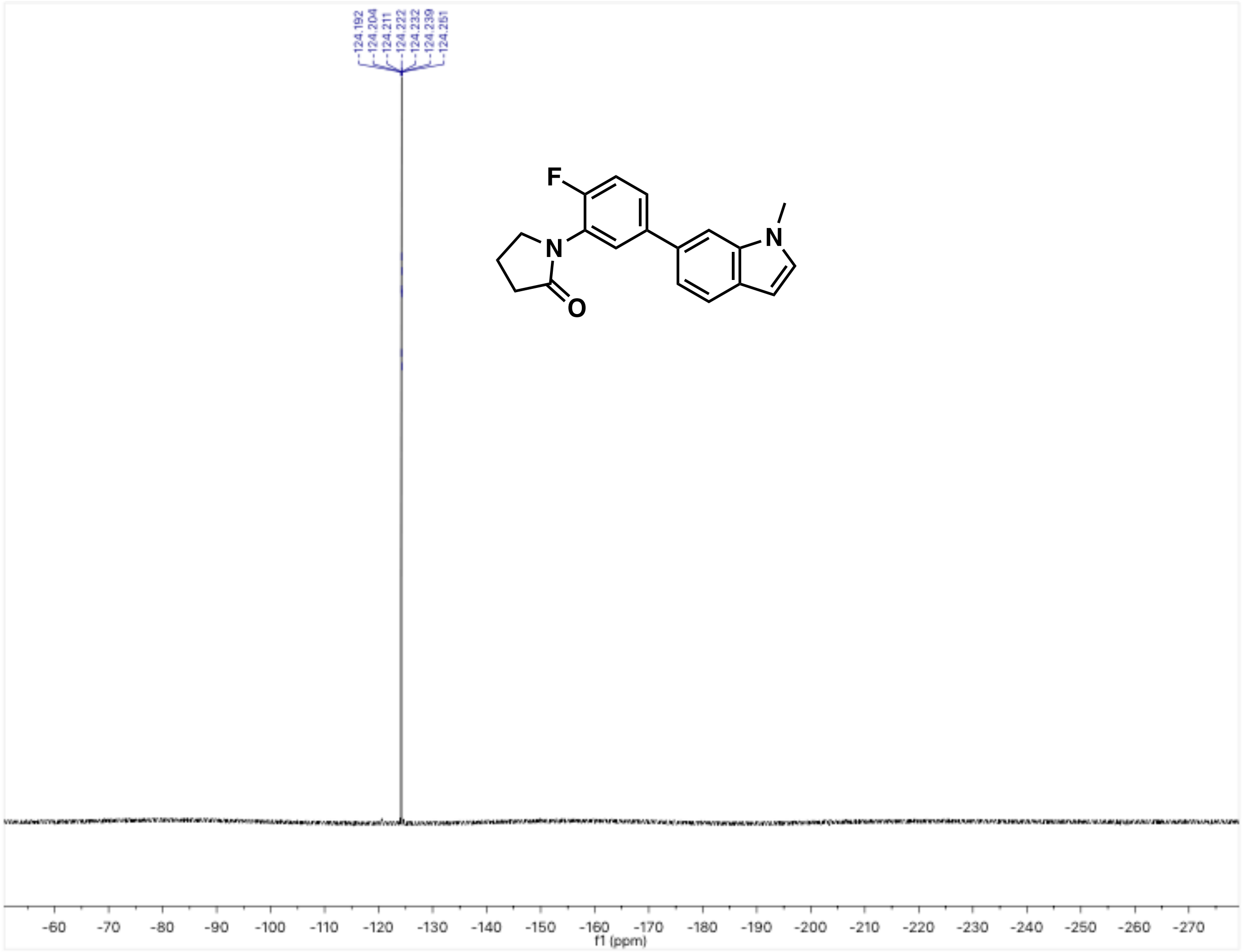

-124.192
-124.204
-124.211
-124.222
-124.232
-124.239
-124.251
F
N
O
N
-60 -70 -80 -90 -100 -110 -120 -130 -140 -150 -160 -170 -180 -190 -200 -210 -220 -230 -240 -250 -260 -270
f1 (ppm)

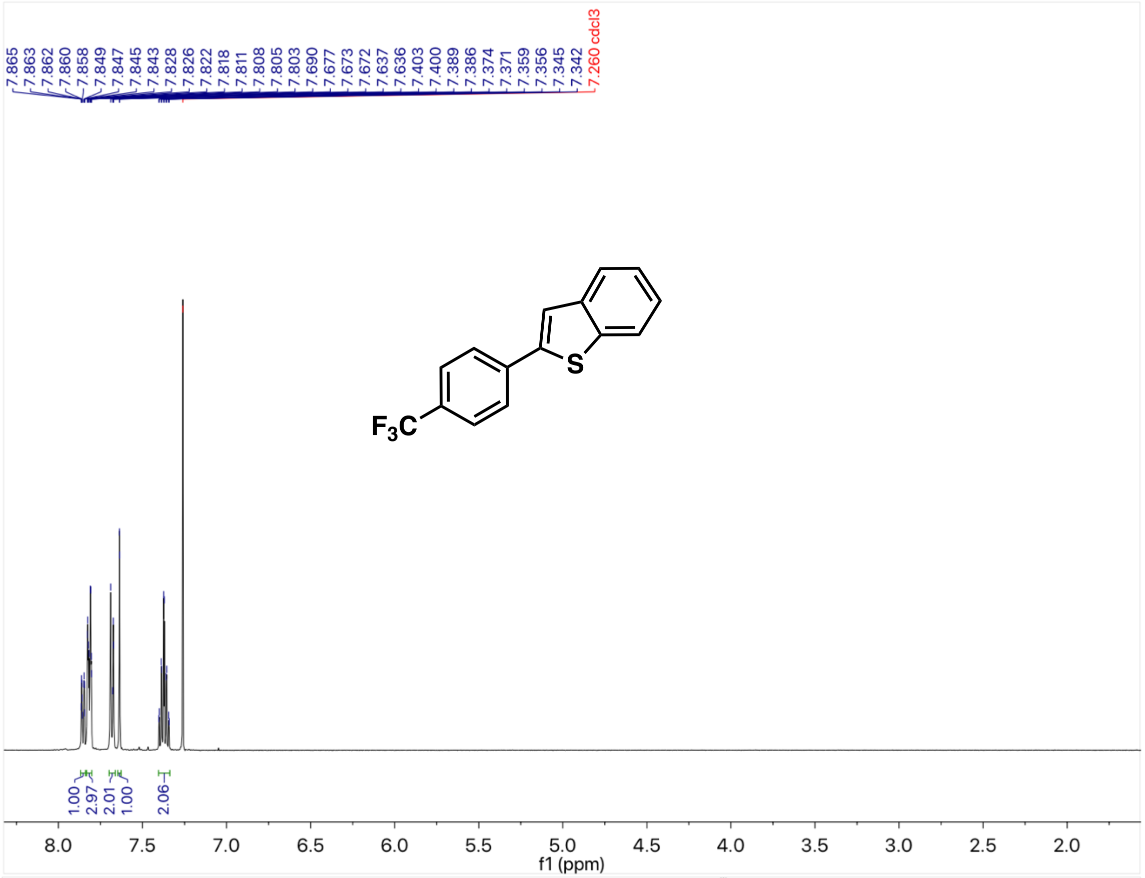
7.865
7.863
7.862
7.860
7.858
7.849
7.847
7.845
7.843
7.828
7.826
7.822
7.818
7.811
7.808
7.805
7.803
7.690
7.677
7.673
7.672
7.637
7.636
7.403
7.400
7.389
7.386
7.374
7.371
7.359
7.356
7.345
7.342
7.260 cdcl3
F3C
S
1.00
2.97
2.01
1.00
2.06
8.0
7.5
7.0
6.5
6.0
5.5
5.0
4.5
4.0
3.5
3.0
2.5
2.0
f1 (ppm)

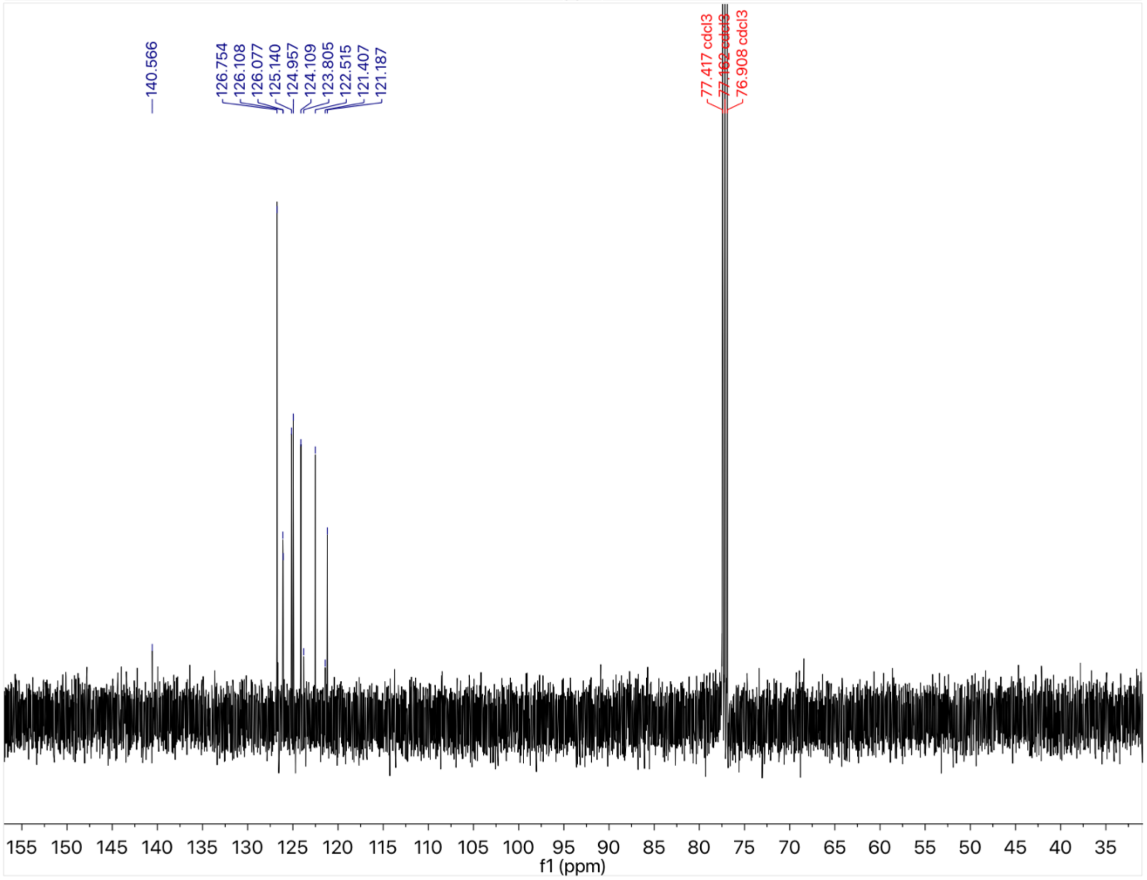
140.566
126.754
126.108
126.077
125.140
124.957
124.109
123.805
122.515
121.407
121.187
77.417 cdcl3
77.162 cdcl3
76.908 cdcl3
155
150
145
140
135
130
125
120
115
110
105
100
95
90
85
80
75
70
65
60
55
50
45
40
35
f1 (ppm)

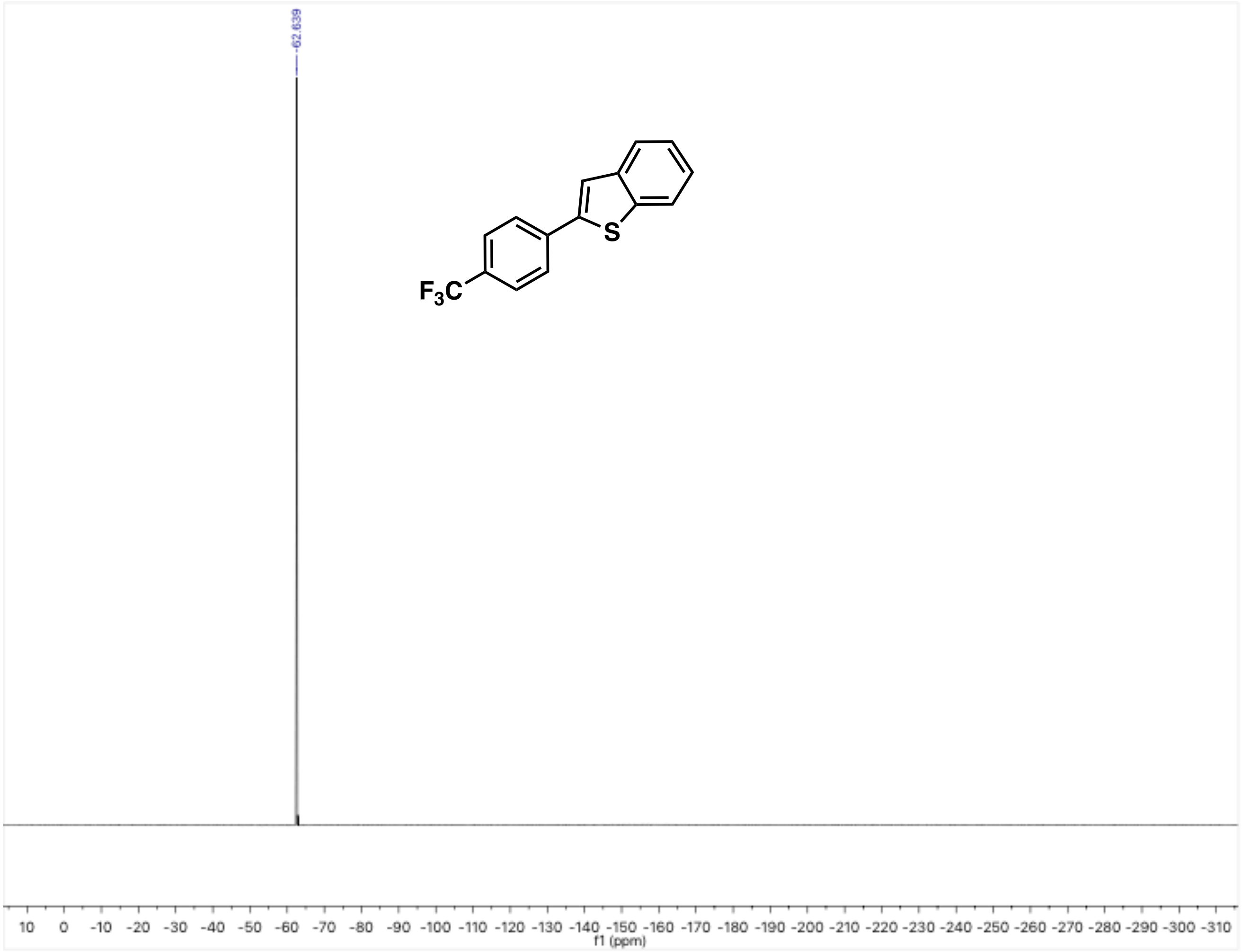
-62.639
F3C
S
10 0 -10 -20 -30 -40 -50 -60 -70 -80 -90 -100 -110 -120 -130 -140 -150 -160 -170 -180 -190 -200 -210 -220 -230 -240 -250 -260 -270 -280 -290 -300 -310
f1 (ppm)

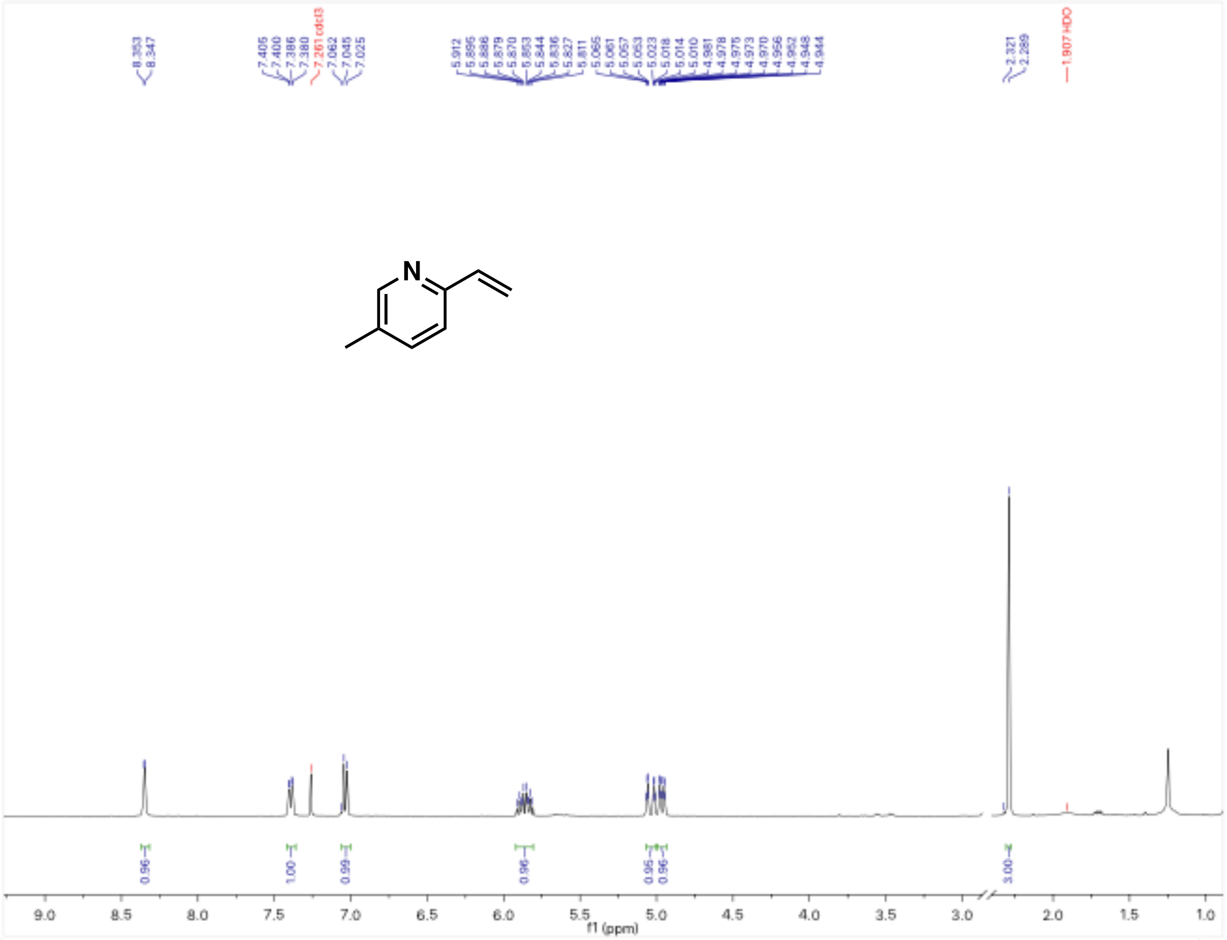
8.353
8.347
7.405
7.400
7.386
7.380
7.261 cdcl3
7.062
7.045
7.025
5.912
5.896
5.888
5.879
5.870
5.863
5.853
5.844
5.836
5.827
5.811
5.065
5.061
5.057
5.053
5.023
5.018
5.014
5.010
4.981
4.978
4.975
4.973
4.970
4.956
4.952
4.948
4.944
2.321
2.289
1.907 HDO
N
0.96
1.00
0.99
0.96
0.95
0.96
3.00
f1 (ppm)

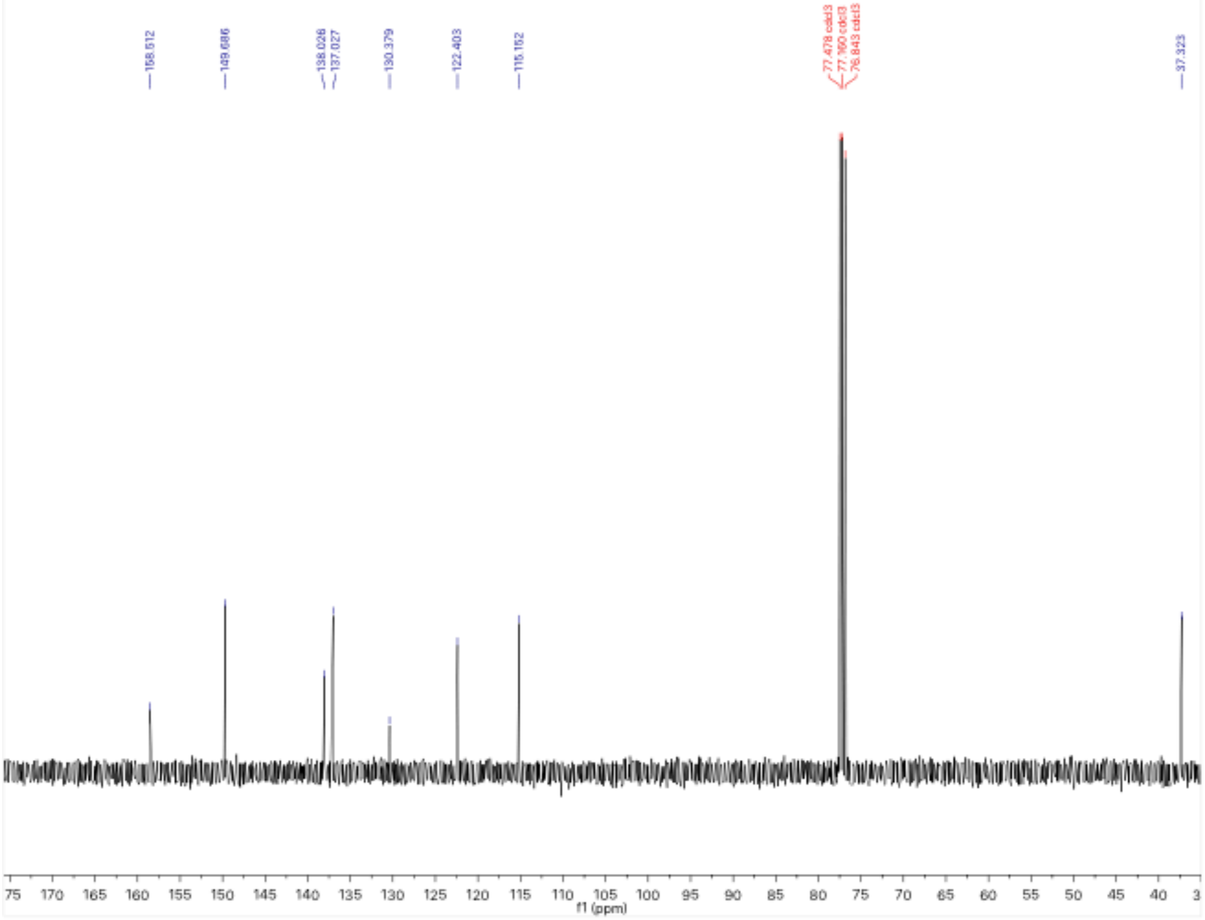
158.512
149.686
138.026
137.027
130.379
122.403
115.152
77.478 cdcl3
77.160 cdcl3
76.843 cdcl3
37.323
f1 (ppm)

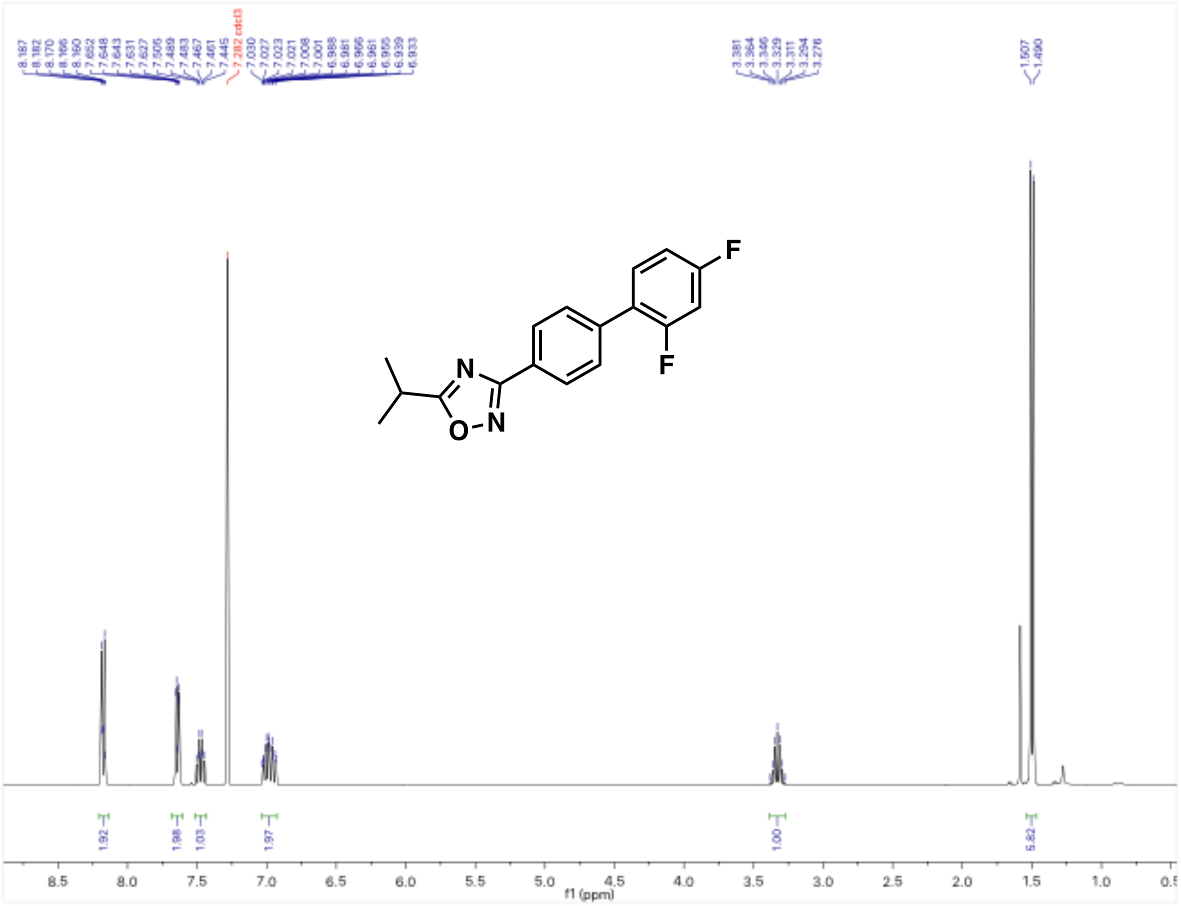

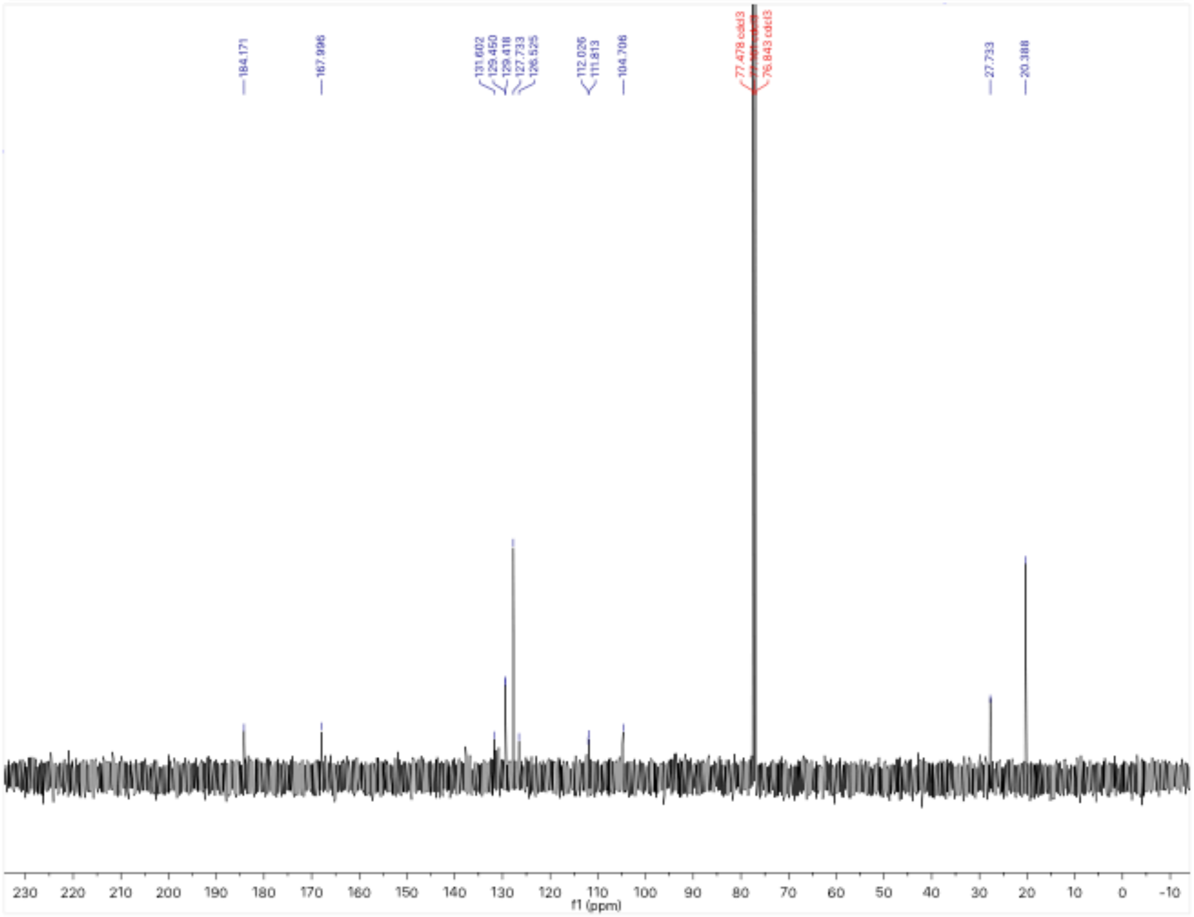

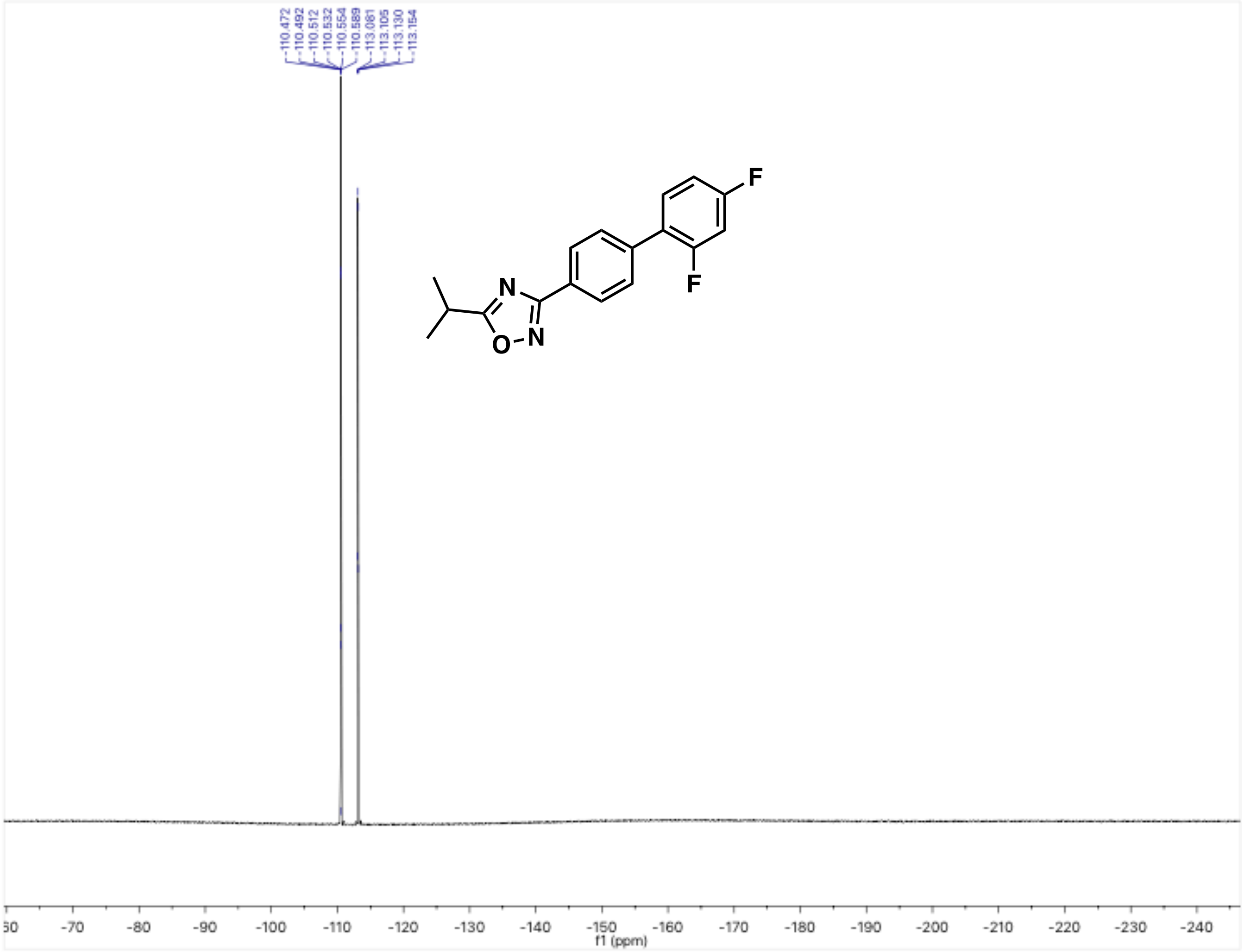

-110.472
-110.492
-110.512
-110.532
-110.554
-110.589
-113.081
-113.105
-113.130
-113.154
N
O
N
F
F
-70
-80
-90
-100
-110
-120
-130
-140
-150
-160
-170
-180
-190
-200
-210
-220
-230
-240
f1 (ppm)

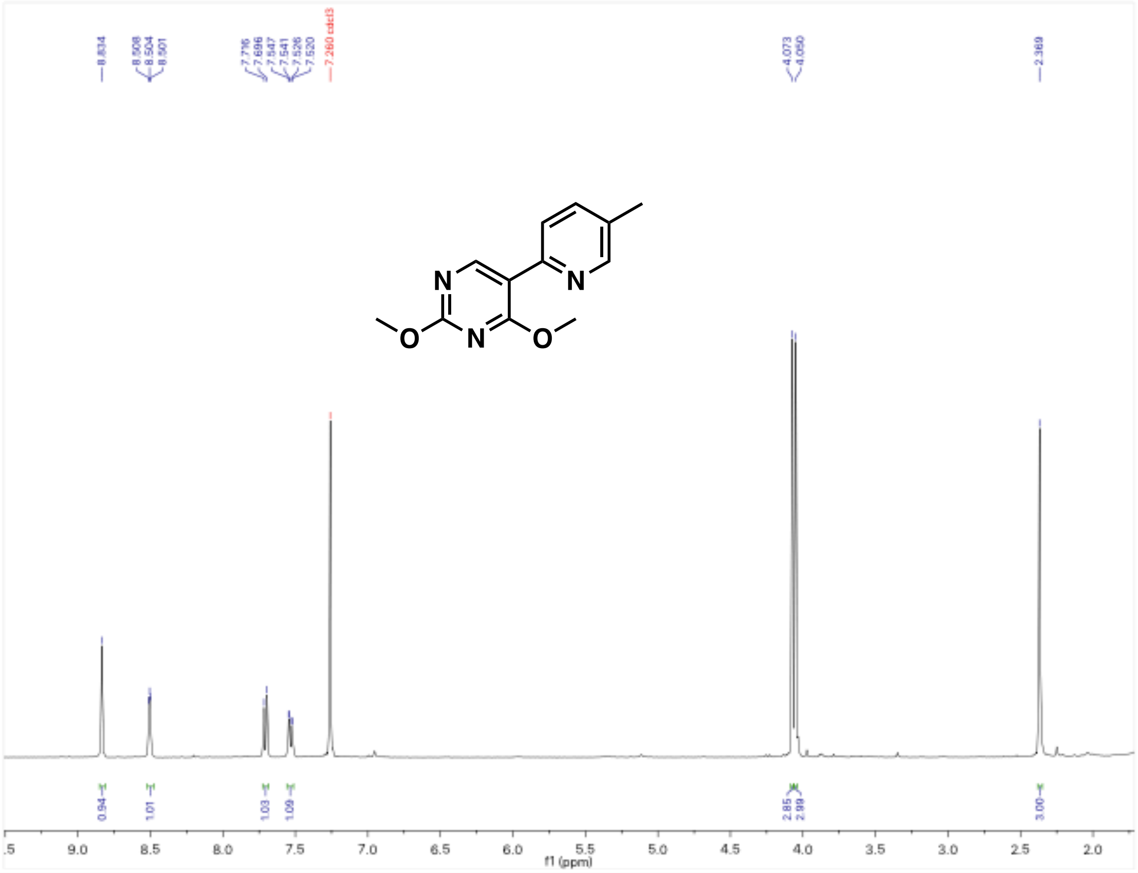

8.834
8.506
8.504
8.501
7.716
7.696
7.547
7.541
7.526
7.520
7.260 cdcl3
4.073
4.050
2.369
N
N
N
O
O
0.94
1.01
1.03
1.09
2.85
2.88
3.00
f1 (ppm)

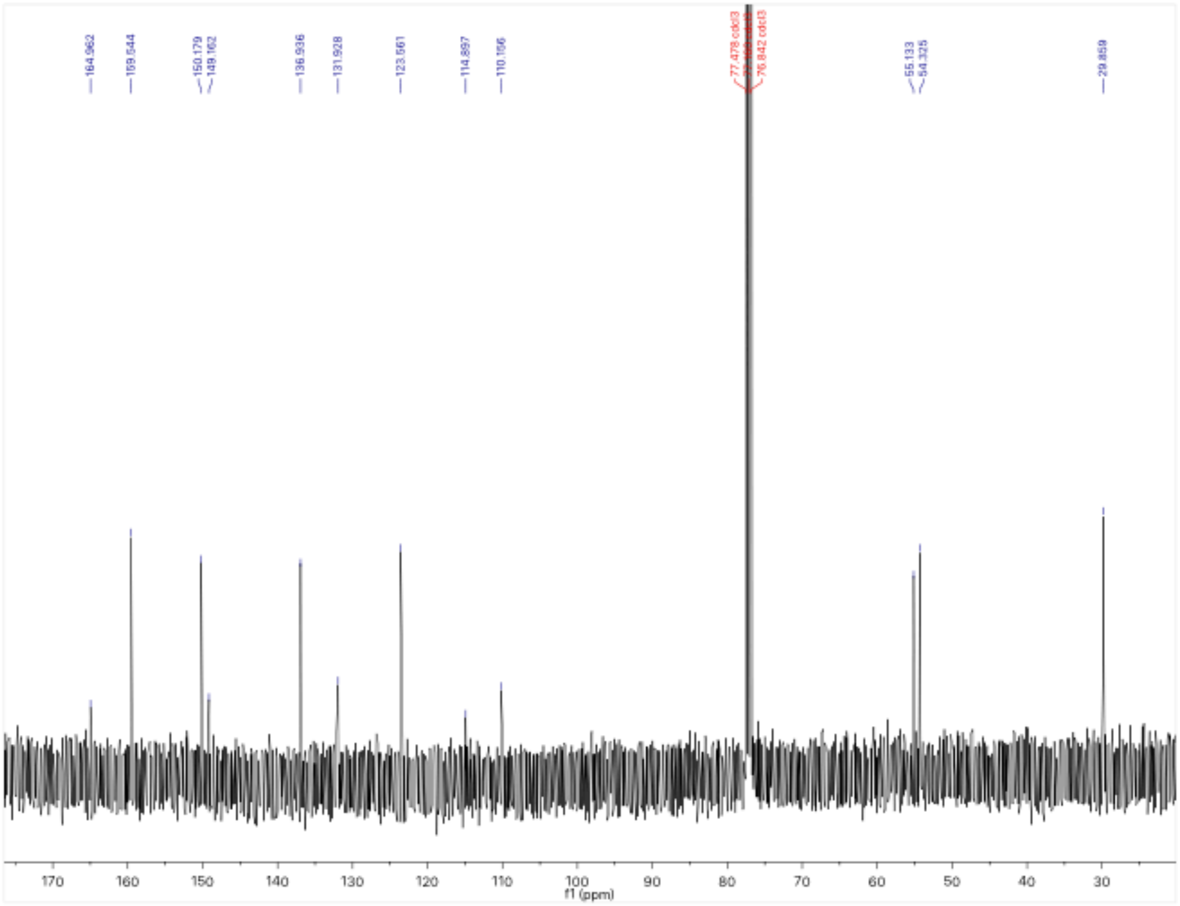

164.962
159.544
150.179
149.162
136.936
131.928
123.561
114.897
110.156
77.478 cdcl3
76.842 cdcl3
55.133
54.325
29.859
f1 (ppm)

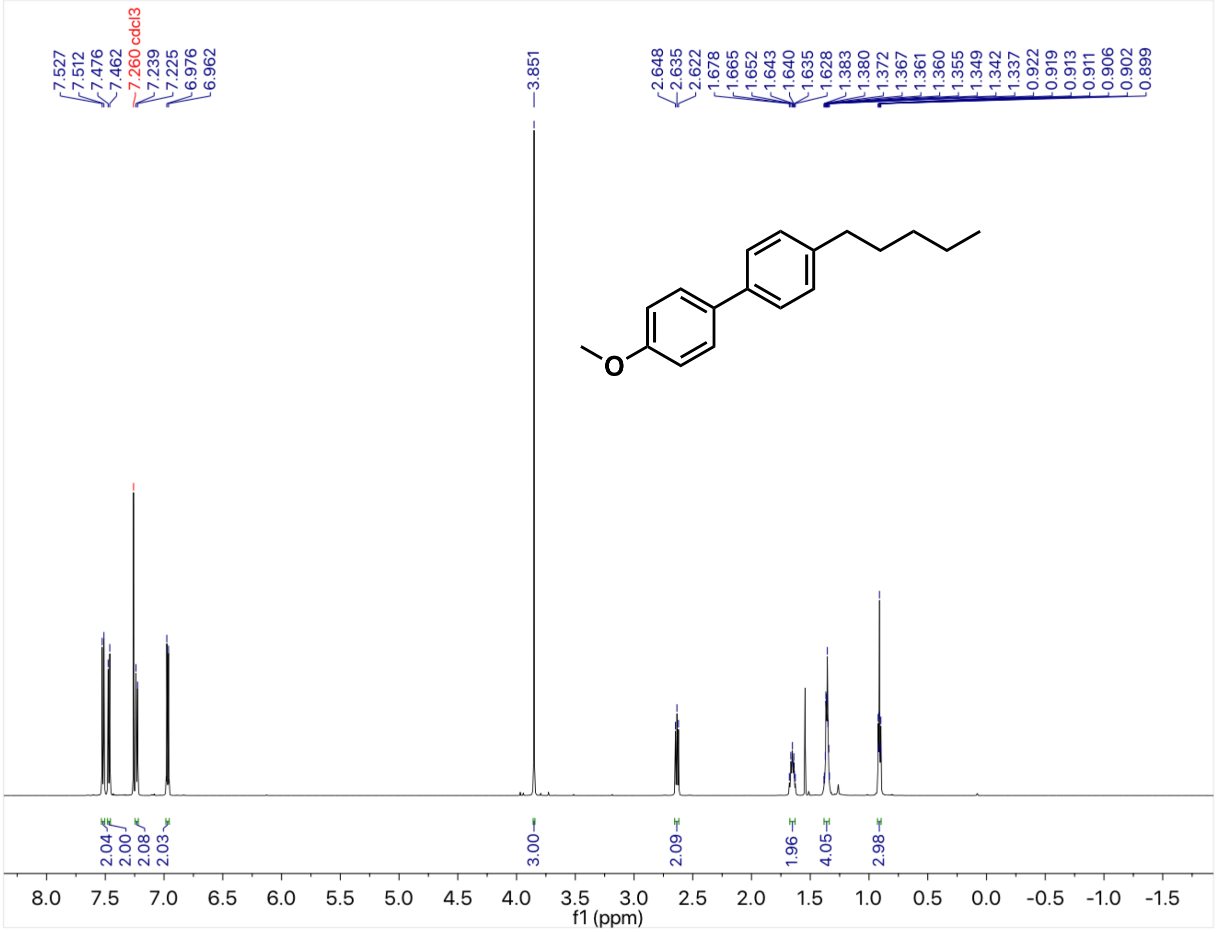
7.527
7.512
7.476
7.462
7.260 cdcl3
7.239
7.225
6.976
6.962
3.851
2.648
2.635
2.622
1.678
1.665
1.652
1.643
1.640
1.635
1.628
1.383
1.380
1.372
1.367
1.361
1.360
1.355
1.349
1.342
1.337
0.922
0.919
0.913
0.911
0.906
0.902
0.899
O
2.04
2.00
2.08
2.03
3.00
2.09
1.96
4.05
2.98
8.0 7.5 7.0 6.5 6.0 5.5 5.0 4.5 4.0 3.5 3.0 2.5 2.0 1.5 1.0 0.5 0.0 -0.5 -1.0 -1.5
f1 (ppm)

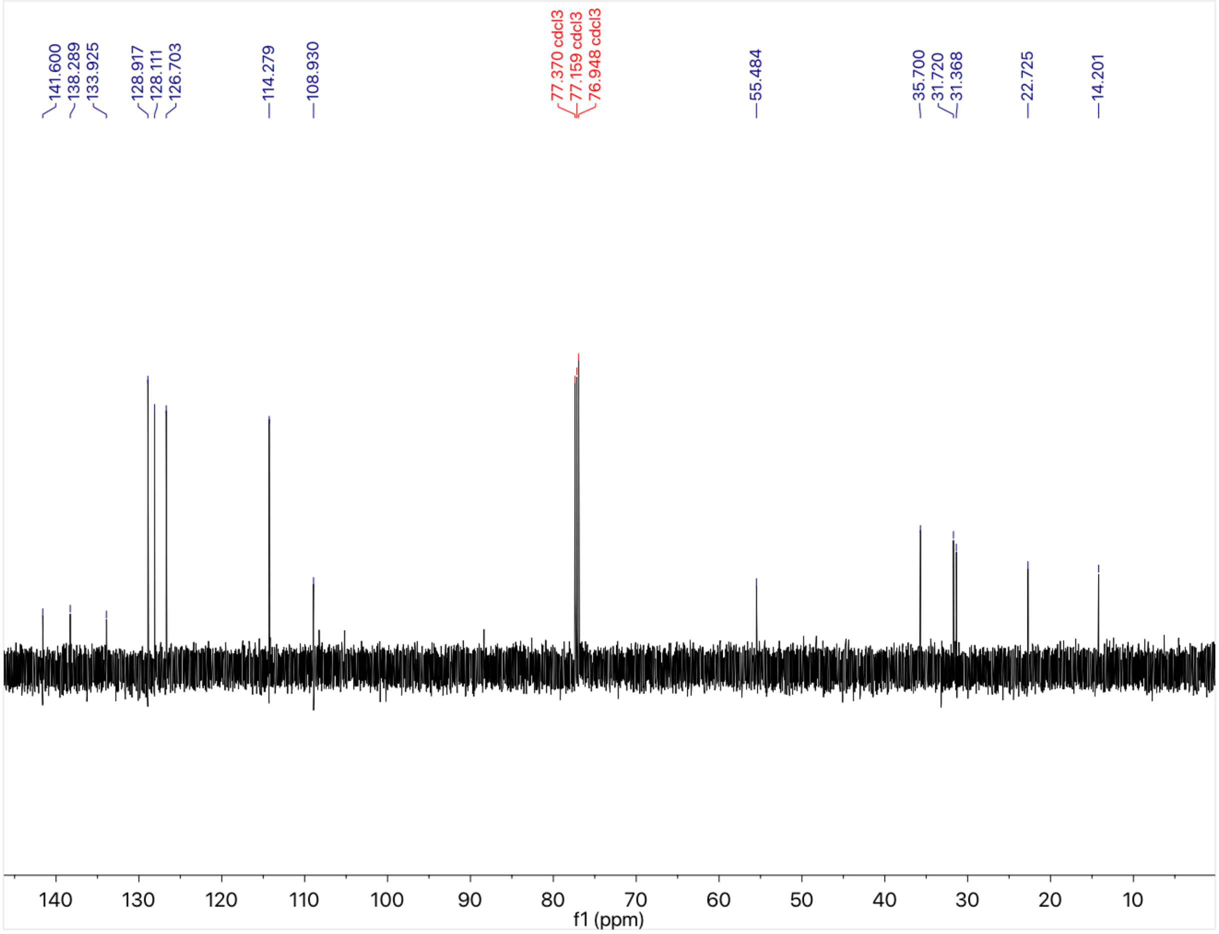
141.600
138.289
133.925
128.917
128.111
126.703
114.279
108.930
77.370 cdcl3
77.159 cdcl3
76.948 cdcl3
55.484
35.700
31.720
31.368
22.725
14.201
140 130 120 110 100 90 80 70 60 50 40 30 20 10
f1 (ppm)

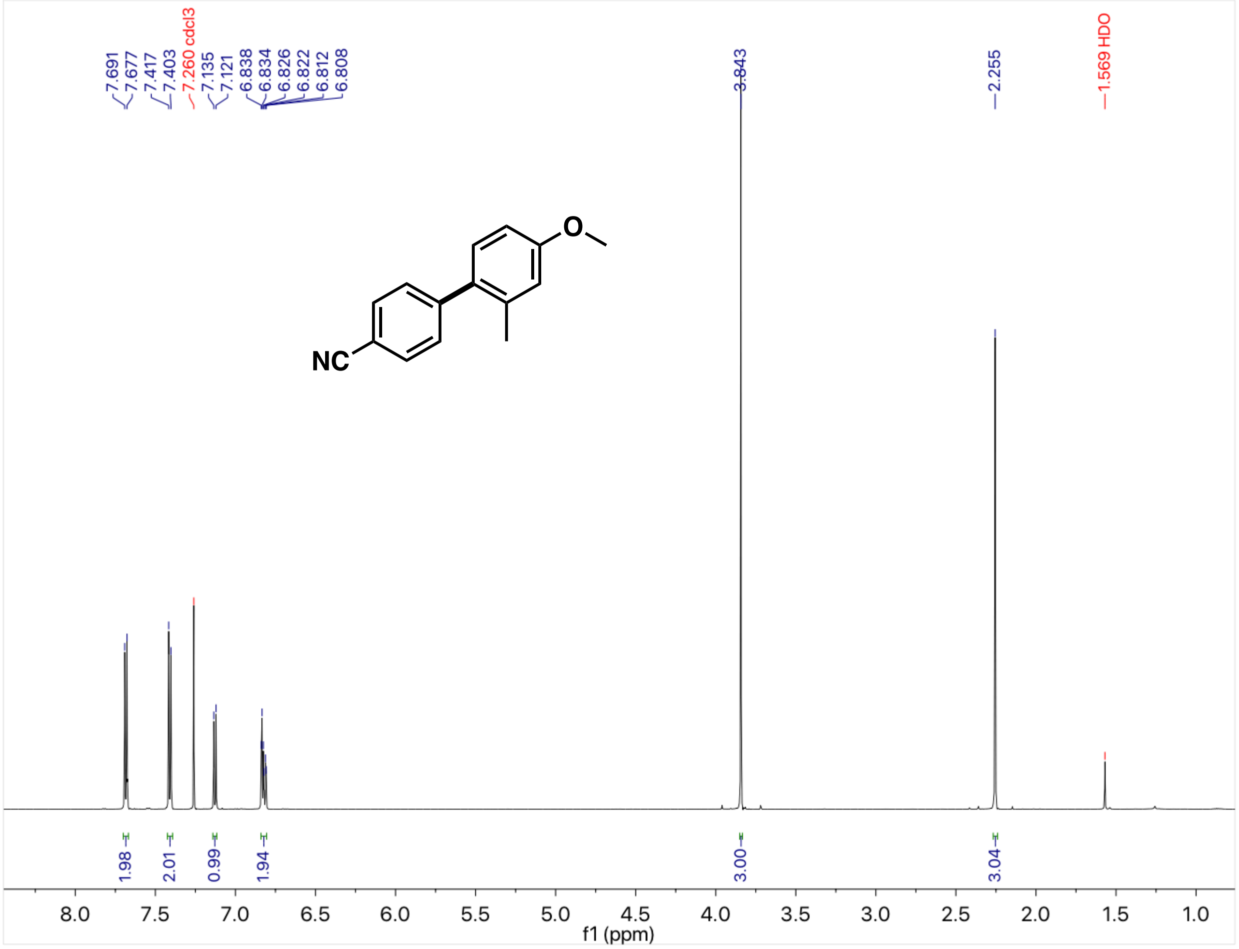
7.691
7.677
7.417
7.403
7.260 cdcl3
7.135
7.121
6.838
6.834
6.826
6.822
6.812
6.808
3.843
2.255
1.569 HDO
NC
O
1.98
2.01
0.99
1.94
3.00
3.04
8.0 7.5 7.0 6.5 6.0 5.5 5.0 4.5 4.0 3.5 3.0 2.5 2.0 1.5 1.0
f1 (ppm)

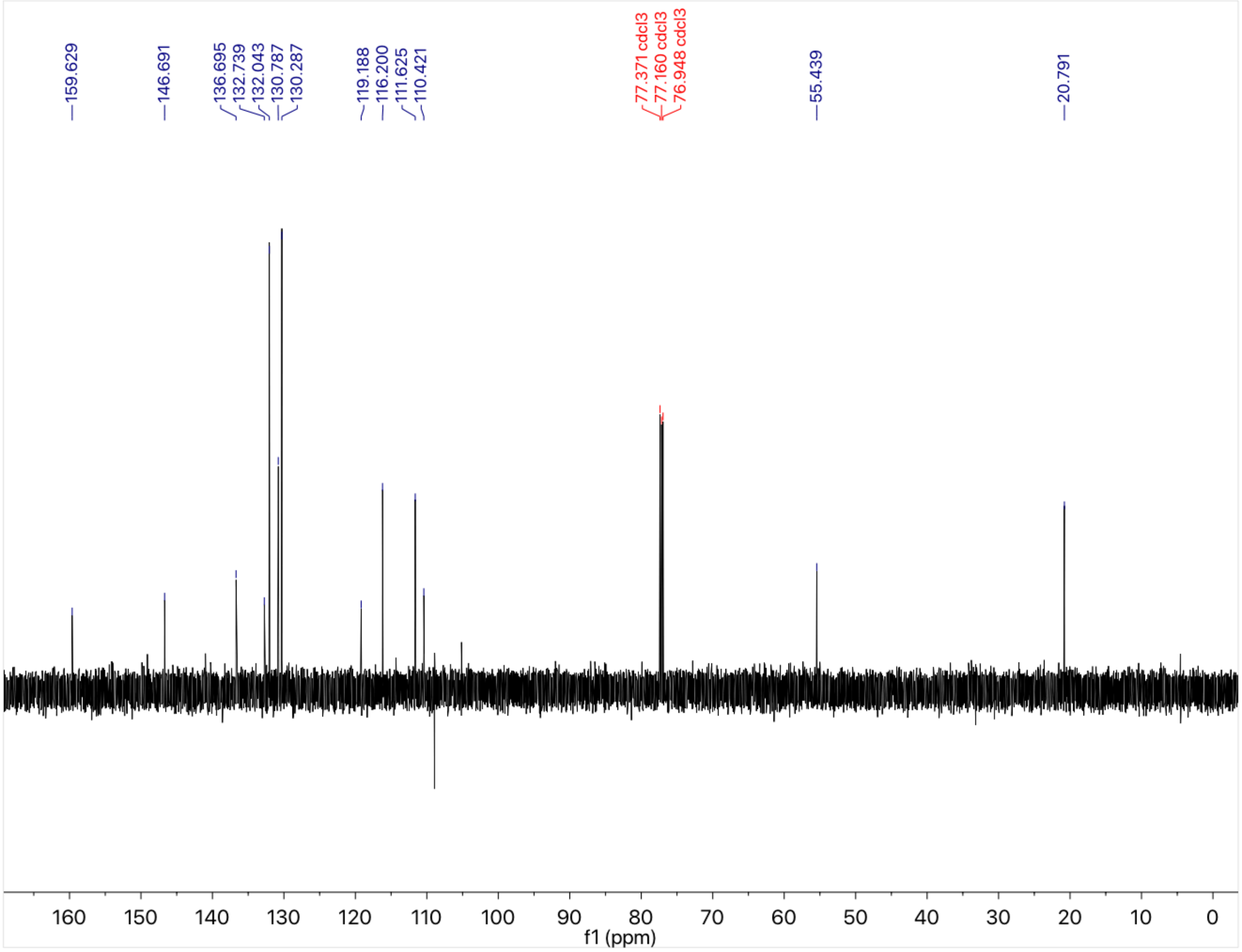
159.629
146.691
136.695
132.739
132.043
130.787
130.287
119.188
116.200
111.625
110.421
77.371 cdcl3
77.160 cdcl3
76.948 cdcl3
55.439
20.791
160 150 140 130 120 110 100 90 80 70 60 50 40 30 20 10 0
f1 (ppm)

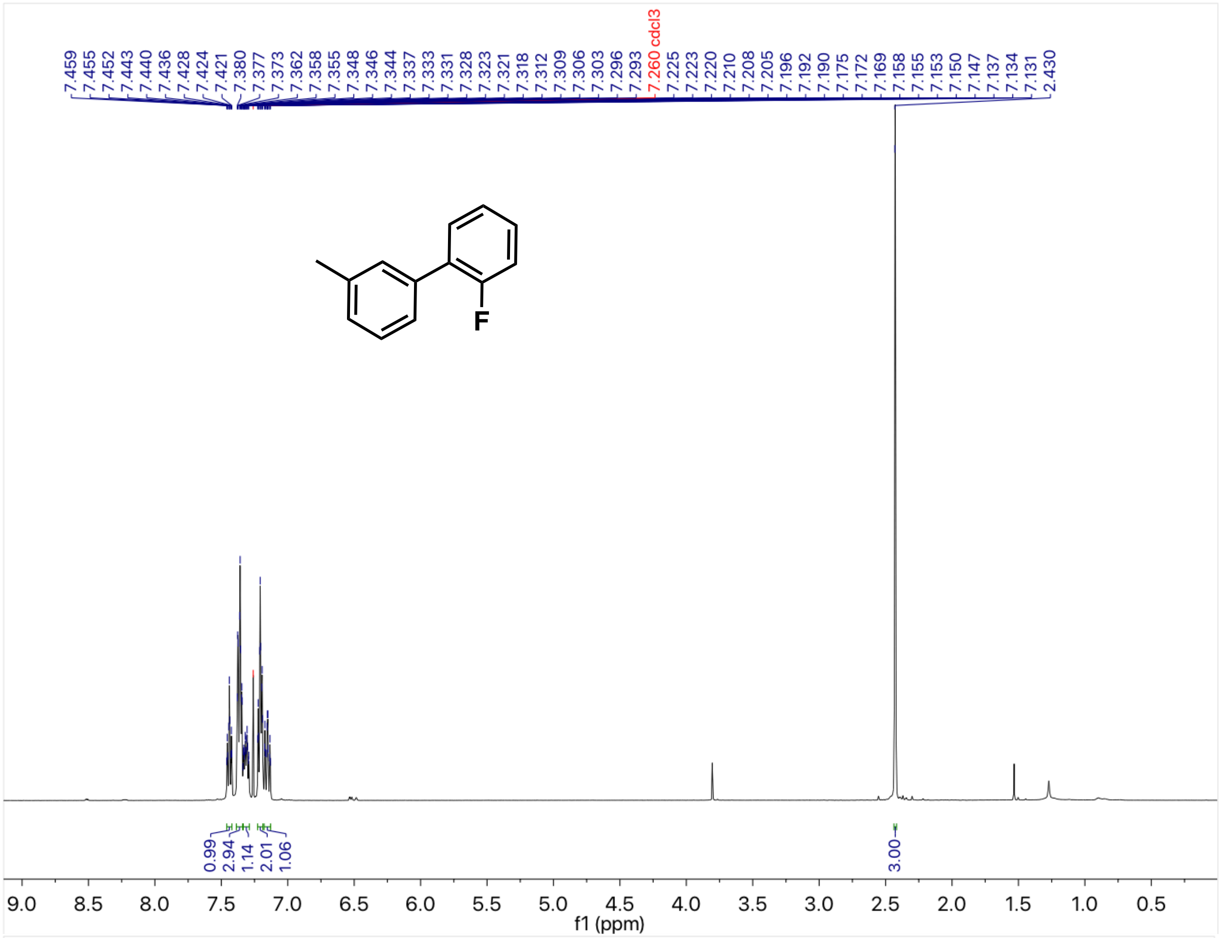
7.459
7.455
7.452
7.443
7.440
7.436
7.428
7.424
7.421
7.380
7.377
7.373
7.362
7.358
7.355
7.348
7.346
7.344
7.337
7.333
7.331
7.328
7.323
7.321
7.318
7.312
7.309
7.306
7.303
7.296
7.293
7.260 cdcl3
7.225
7.223
7.220
7.210
7.208
7.205
7.196
7.192
7.190
7.175
7.172
7.169
7.158
7.155
7.153
7.150
7.147
7.137
7.134
7.131
2.430
F
0.99
2.94
1.14
2.01
1.06
3.00
9.0 8.5 8.0 7.5 7.0 6.5 6.0 5.5 5.0 4.5 4.0 3.5 3.0 2.5 2.0 1.5 1.0 0.5
f1 (ppm)

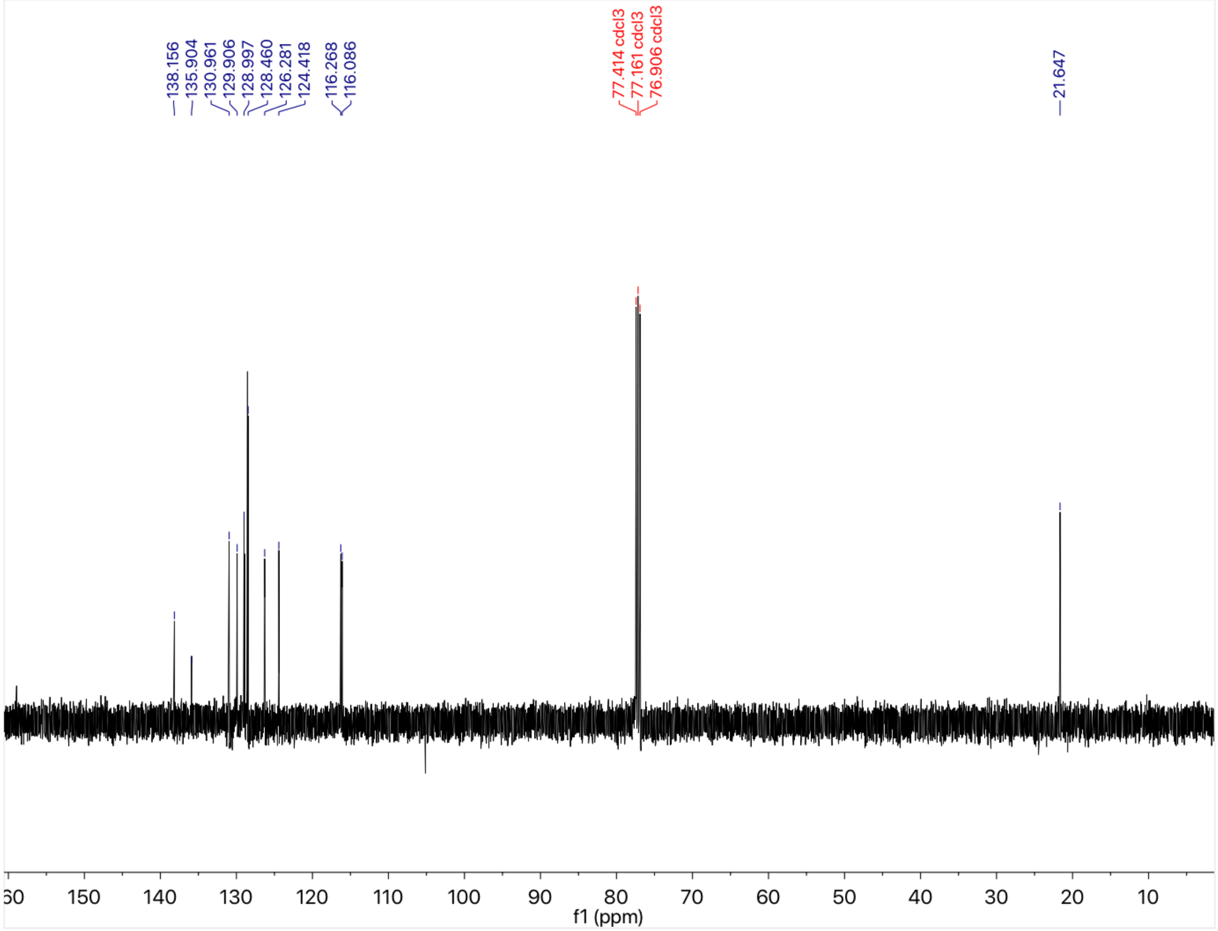
138.156
135.904
130.961
129.906
128.997
128.460
126.281
124.418
116.268
116.086
77.414 cdcl3
77.161 cdcl3
76.906 cdcl3
21.647
150 140 130 120 110 100 90 80 70 60 50 40 30 20 10
f1 (ppm)

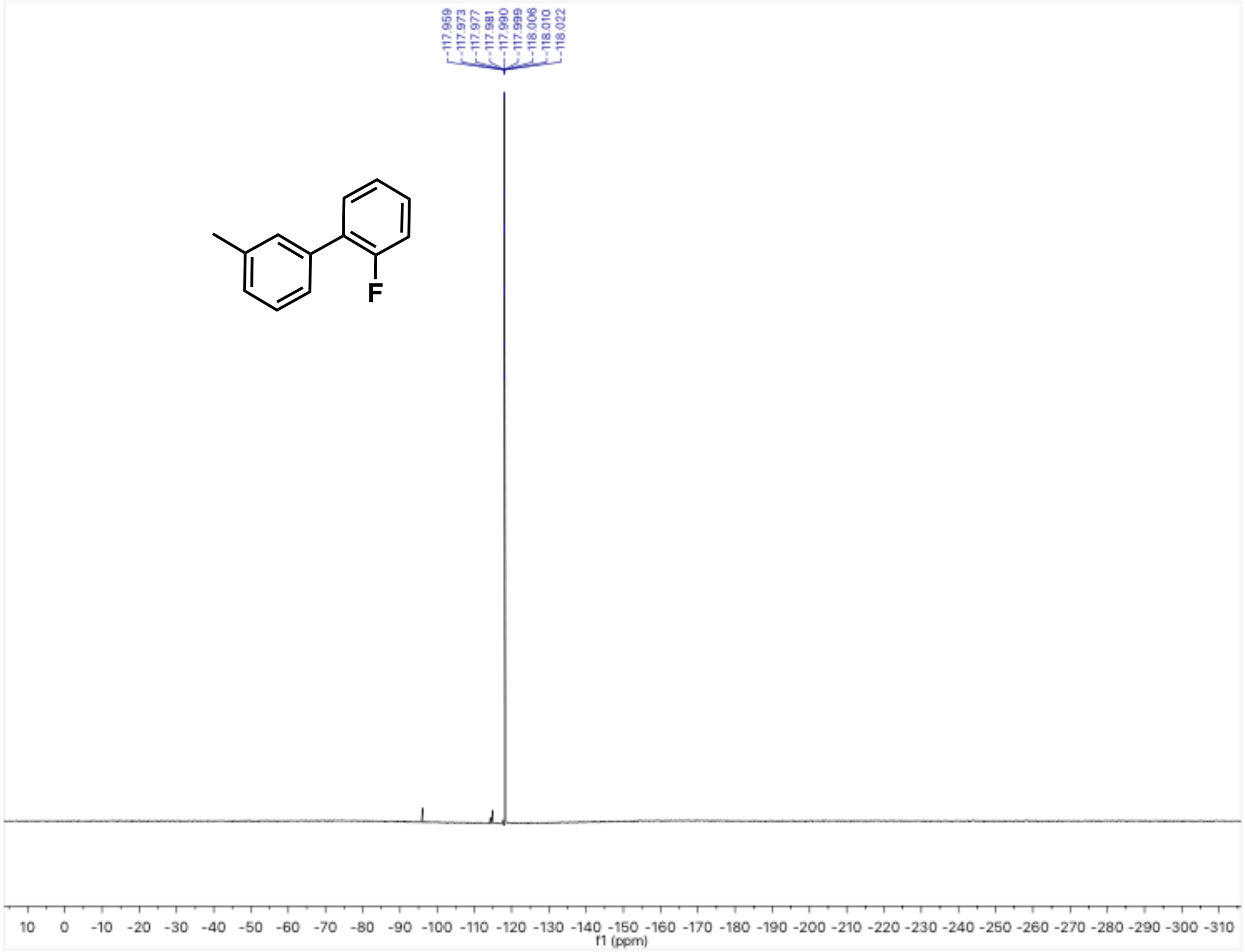

-117.959
-117.973
-117.977
-117.981
-117.990
-117.999
-118.006
-118.010
-118.022
F
10 0 -10 -20 -30 -40 -50 -60 -70 -80 -90 -100 -110 -120 -130 -140 -150 -160 -170 -180 -190 -200 -210 -220 -230 -240 -250 -260 -270 -280 -290 -300 -310
f1 (ppm)

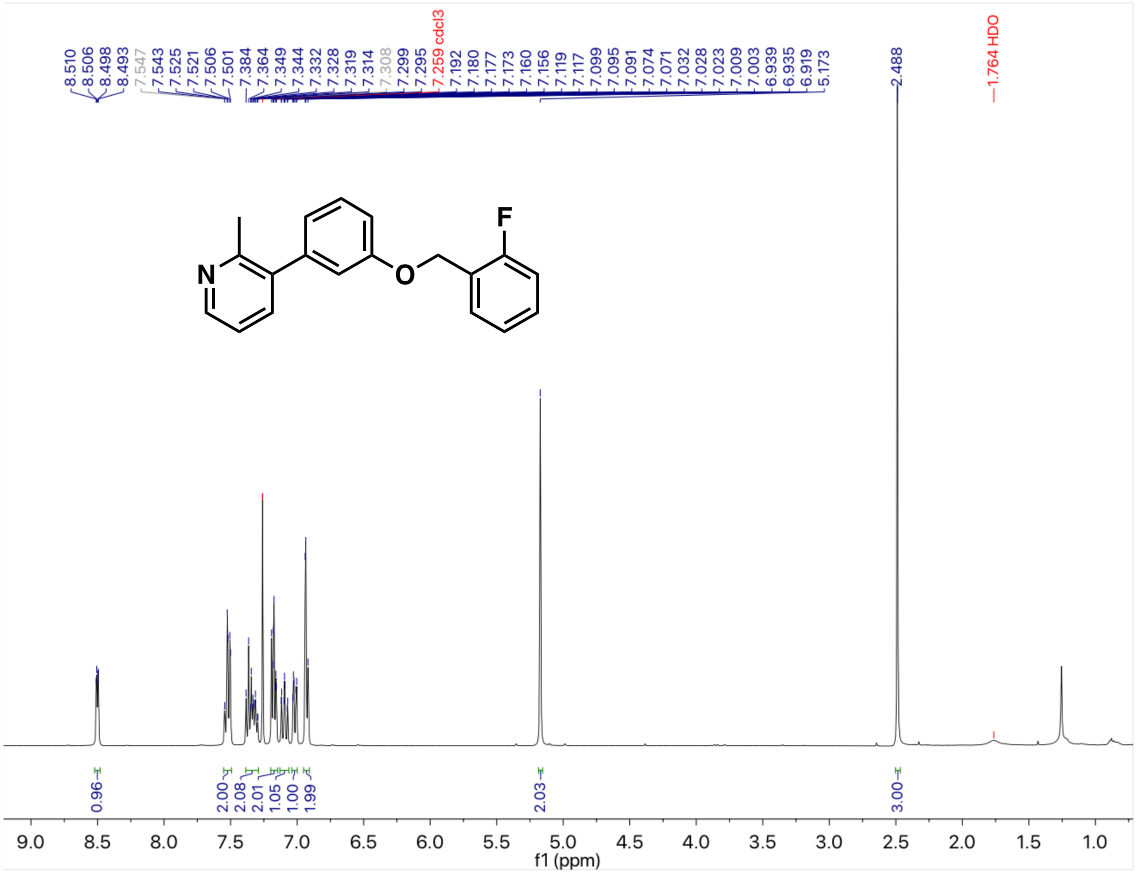
8.510
8.506
8.498
8.493
7.547
7.543
7.525
7.521
7.506
7.501
7.384
7.364
7.349
7.344
7.332
7.328
7.319
7.314
7.308
7.299
7.295
7.259 cdcl3
7.192
7.180
7.177
7.173
7.160
7.156
7.119
7.117
7.099
7.095
7.091
7.074
7.071
7.032
7.028
7.023
7.009
7.003
6.939
6.935
6.919
5.173
2.488
1.764 HDO
F
N
O
0.96
2.00
2.08
2.01
1.05
1.00
1.99
2.03
3.00
9.0 8.5 8.0 7.5 7.0 6.5 6.0 5.5 5.0 4.5 4.0 3.5 3.0 2.5 2.0 1.5 1.0
f1 (ppm)

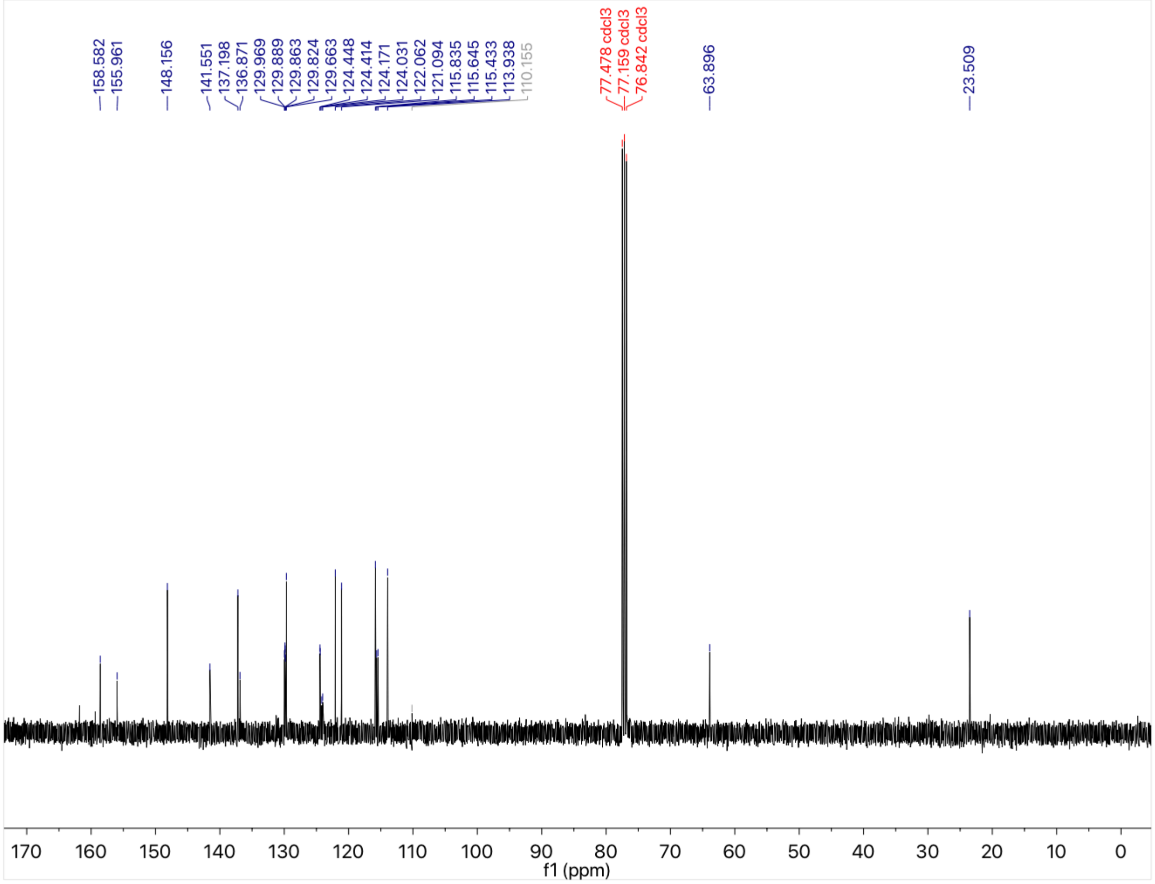
158.582
155.961
148.156
141.551
137.198
136.871
129.969
129.889
129.863
129.824
129.663
124.448
124.414
124.171
124.031
122.062
121.094
115.835
115.645
115.433
113.938
110.155
77.478 cdcl3
77.159 cdcl3
76.842 cdcl3
63.896
23.509
170 160 150 140 130 120 110 100 90 80 70 60 50 40 30 20 10 0
f1 (ppm)

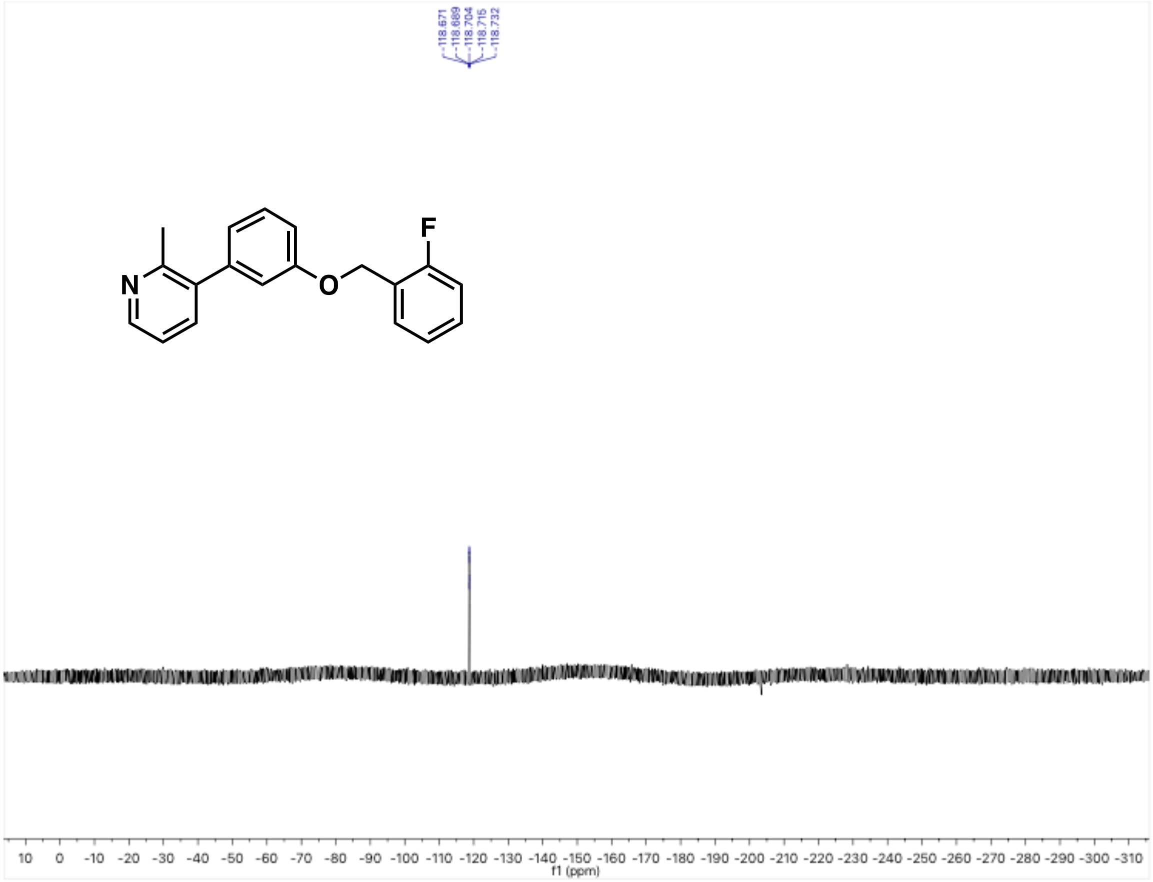
-118.671
-118.689
-118.704
-118.715
-118.732
F
N
O
10 0 -10 -20 -30 -40 -50 -60 -70 -80 -90 -100 -110 -120 -130 -140 -150 -160 -170 -180 -190 -200 -210 -220 -230 -240 -250 -260 -270 -280 -290 -300 -310
f1 (ppm)

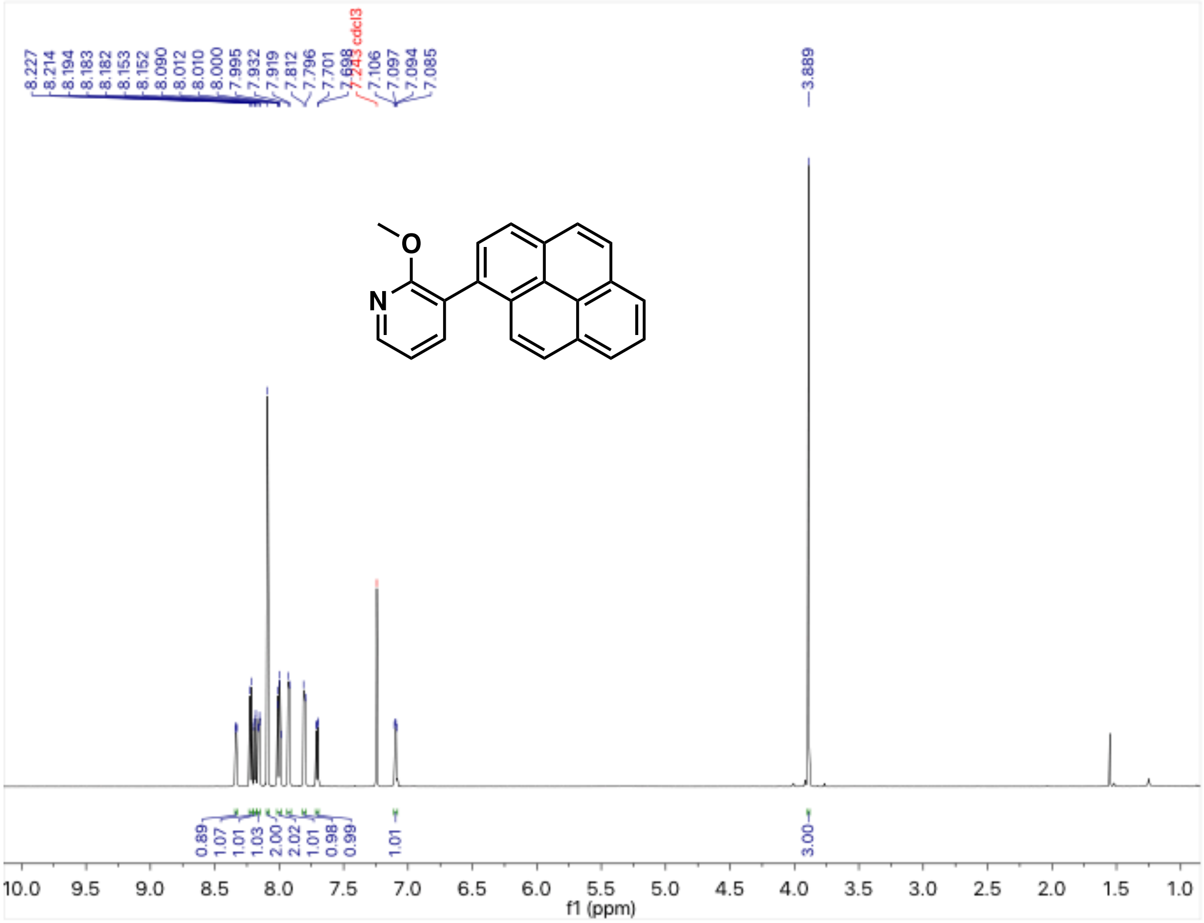
8.227
8.214
8.194
8.183
8.182
8.153
8.152
8.090
8.012
8.010
8.000
7.995
7.932
7.919
7.812
7.796
7.701
7.698
7.243 cdcl3
7.106
7.097
7.094
7.085
3.889
O
N
0.89
1.07
1.01
1.03
2.00
2.02
1.01
0.98
0.99
1.01
3.00
10.0 9.5 9.0 8.5 8.0 7.5 7.0 6.5 6.0 5.5 5.0 4.5 4.0 3.5 3.0 2.5 2.0 1.5 1.0
f1 (ppm)

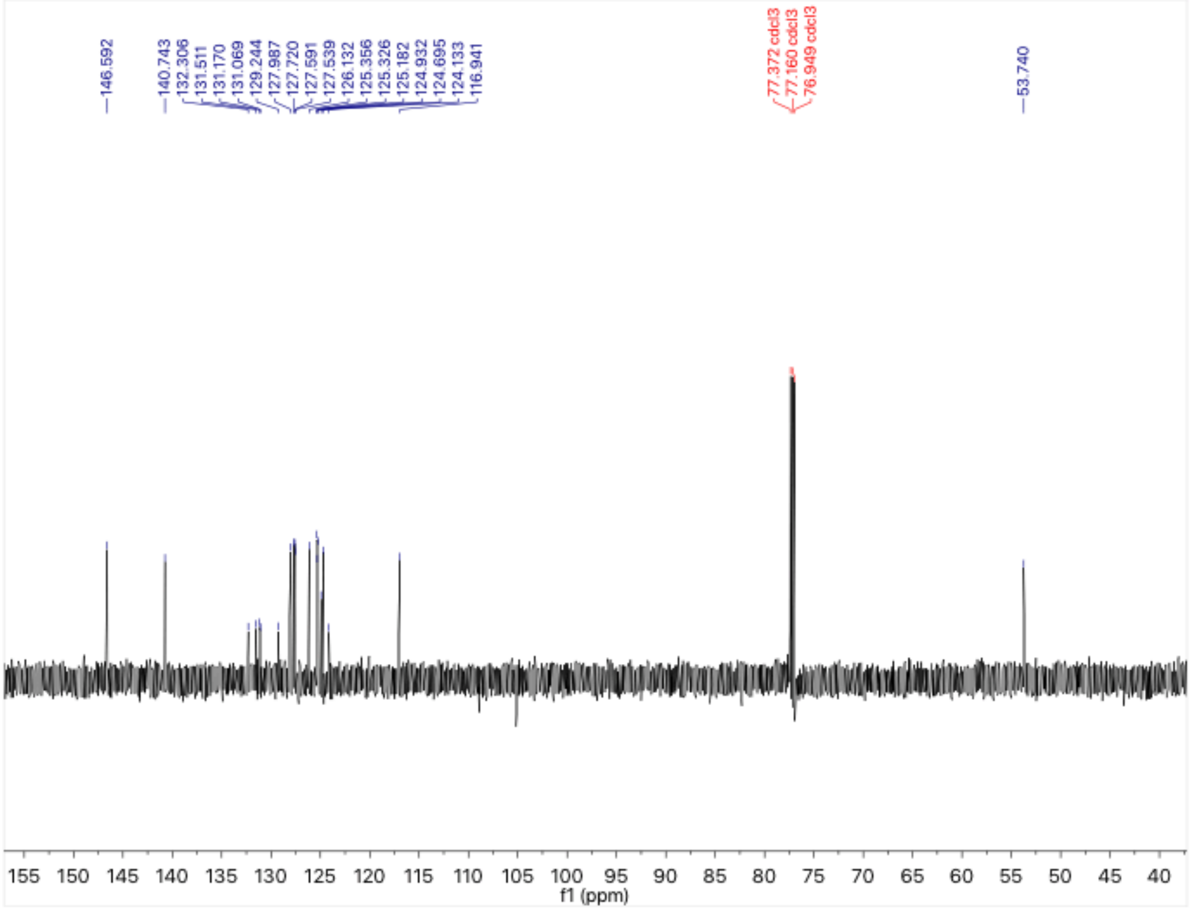
146.592
140.743
132.306
131.511
131.170
131.069
129.244
127.987
127.720
127.591
127.539
126.132
125.356
125.326
125.182
124.932
124.695
124.133
116.941
77.372 cdcl3
77.160 cdcl3
76.949 cdcl3
53.740
155 150 145 140 135 130 125 120 115 110 105 100 95 90 85 80 75 70 65 60 55 50 45 40
f1 (ppm)

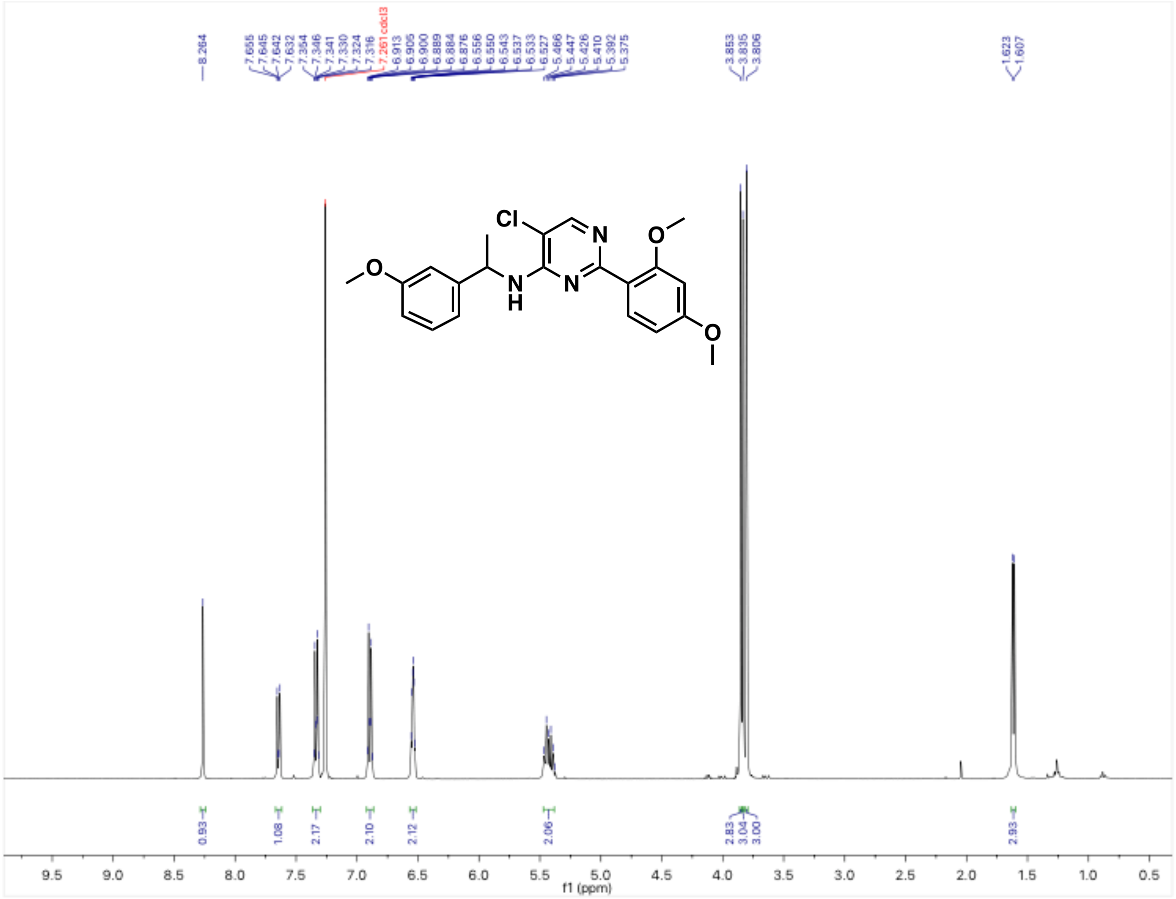

8.264
7.655
7.645
7.642
7.632
7.354
7.346
7.341
7.330
7.324
7.316
7.261 cdcl3
6.913
6.905
6.900
6.889
6.884
6.876
6.556
6.550
6.543
6.537
6.533
6.527
5.466
5.447
5.426
5.410
5.392
5.375
3.853
3.835
3.806
1.623
1.607
Cl
N
O
N
H
N
O
O
0.93
1.08
2.17
2.10
2.12
2.06
2.83
3.04
3.00
2.93
9.5 9.0 8.5 8.0 7.5 7.0 6.5 6.0 5.5 5.0 4.5 4.0 3.5 3.0 2.5 2.0 1.5 1.0 0.5
f1 (ppm)

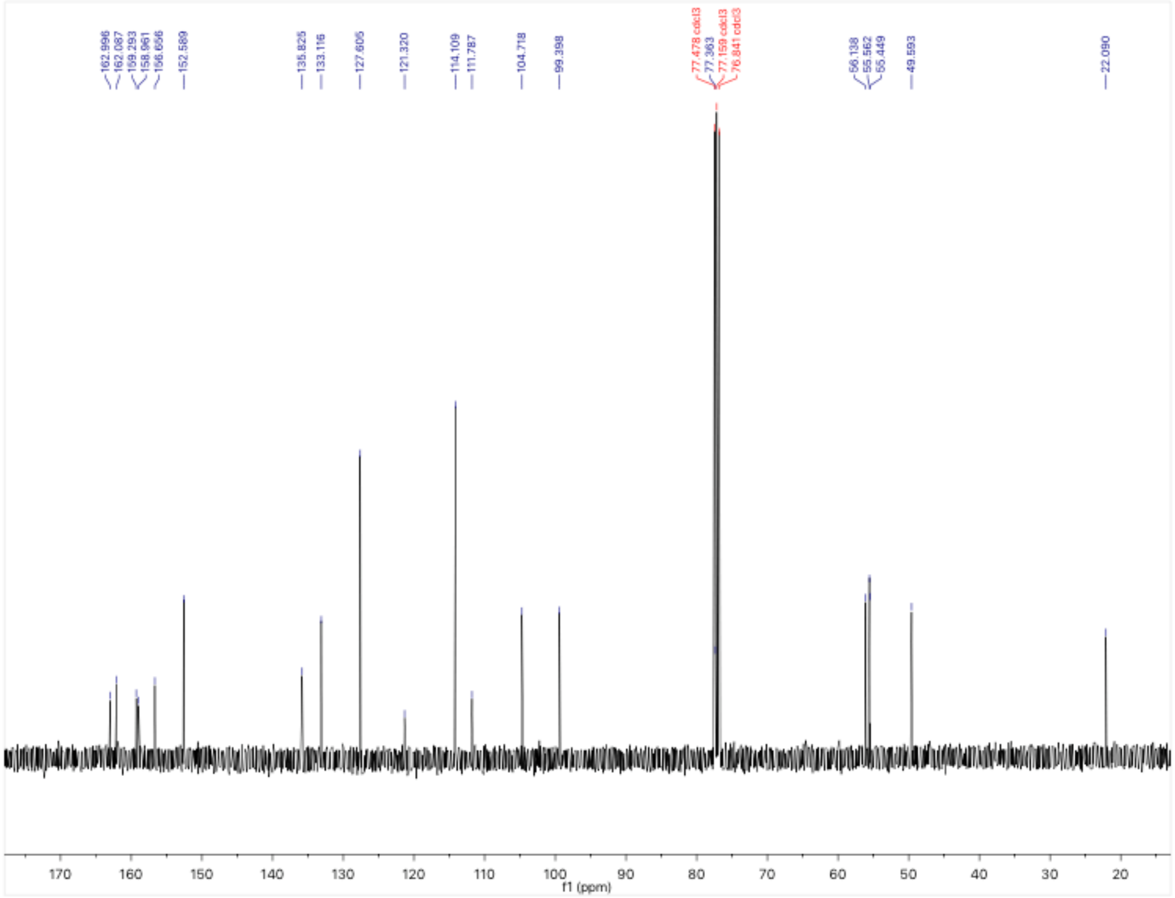

162.996
162.087
159.293
158.961
156.656
152.589
135.825
133.116
127.605
121.320
114.109
111.787
104.718
99.398
77.478 cdcl3
77.363
77.159 cdcl3
76.841 cdcl3
56.138
55.562
55.449
49.593
22.090
170 160 150 140 130 120 110 100 90 80 70 60 50 40 30 20
f1 (ppm)

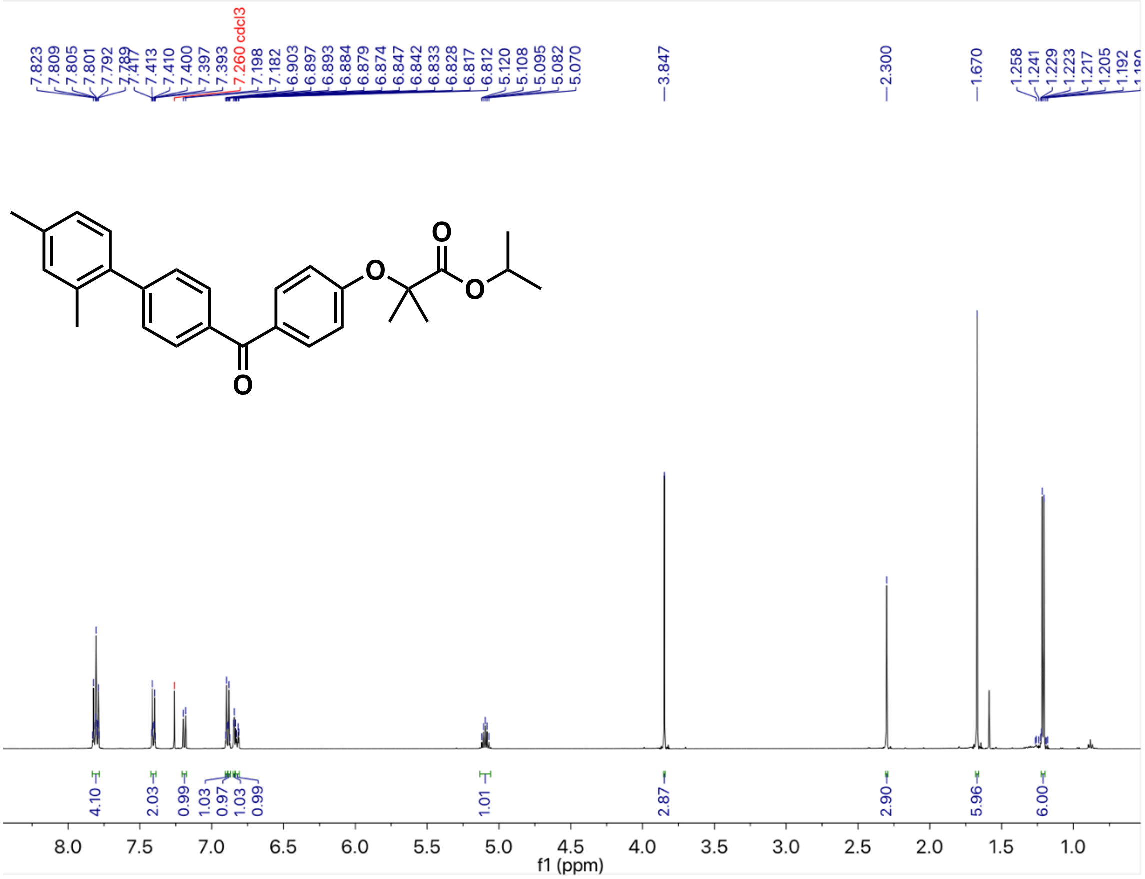
7.823
7.809
7.805
7.801
7.792
7.789
7.417
7.413
7.410
7.400
7.397
7.393
7.260 cdcl3
7.198
7.182
6.903
6.897
6.893
6.884
6.879
6.874
6.847
6.842
6.833
6.828
6.817
6.812
5.120
5.108
5.095
5.082
5.070
3.847
2.300
1.670
1.258
1.241
1.229
1.223
1.217
1.205
1.192
O
O
O
O
4.10
2.03
0.99
1.03
0.97
1.03
0.99
1.01
2.87
2.90
5.96
6.00
8.0 7.5 7.0 6.5 6.0 5.5 5.0 4.5 4.0 3.5 3.0 2.5 2.0 1.5 1.0
f1 (ppm)

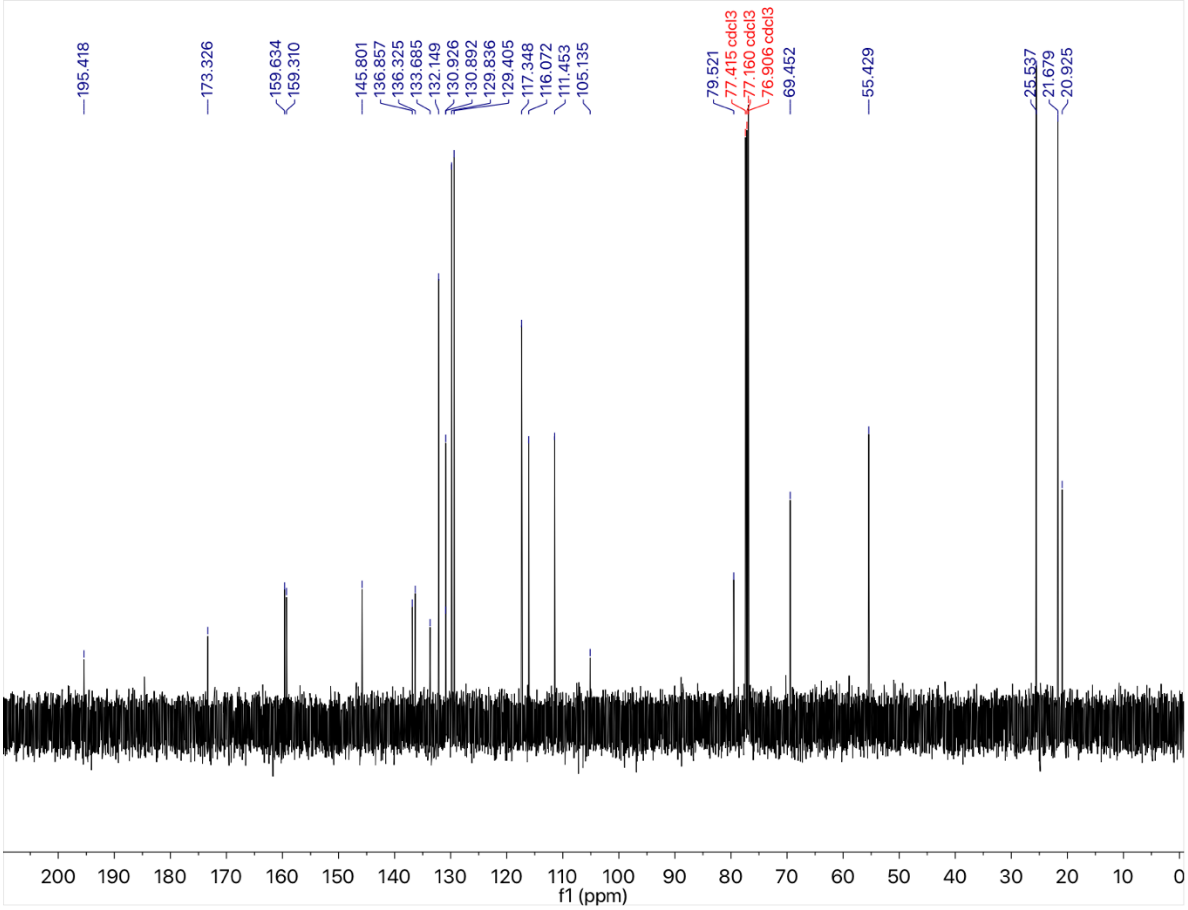
195.418
173.326
159.634
159.310
145.801
136.857
136.325
133.685
132.149
130.926
130.892
129.836
129.405
117.348
116.072
111.453
105.135
79.521
77.415 cdcl3
77.160 cdcl3
76.906 cdcl3
69.452
55.429
25.537
21.679
20.925
200 190 180 170 160 150 140 130 120 110 100 90 80 70 60 50 40 30 20 10 0
f1 (ppm)

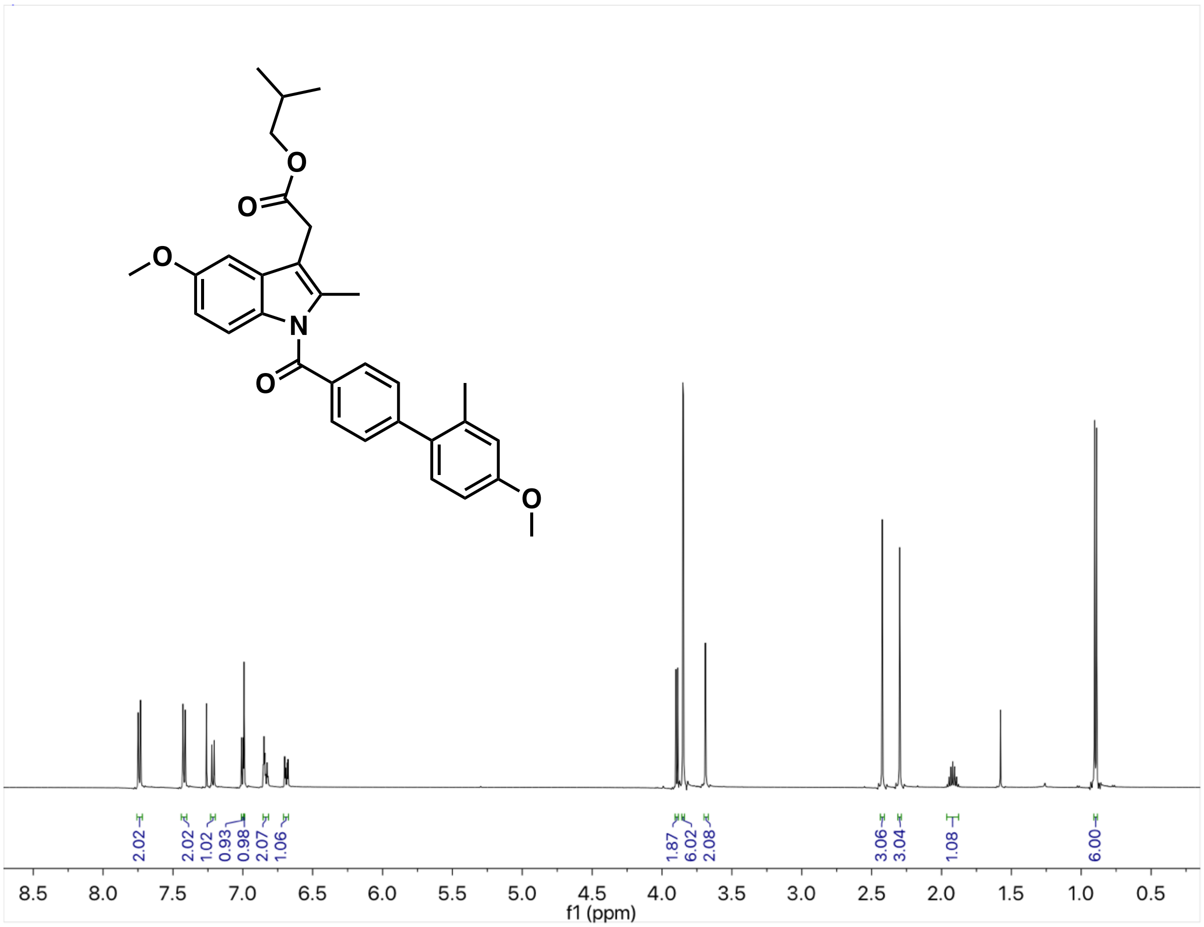

2.02
2.02
1.02
0.93
0.98
2.07
1.06
1.87
6.02
2.08
3.06
3.04
1.08
6.00
8.5 8.0 7.5 7.0 6.5 6.0 5.5 5.0 4.5 4.0 3.5 3.0 2.5 2.0 1.5 1.0 0.5
f1 (ppm)

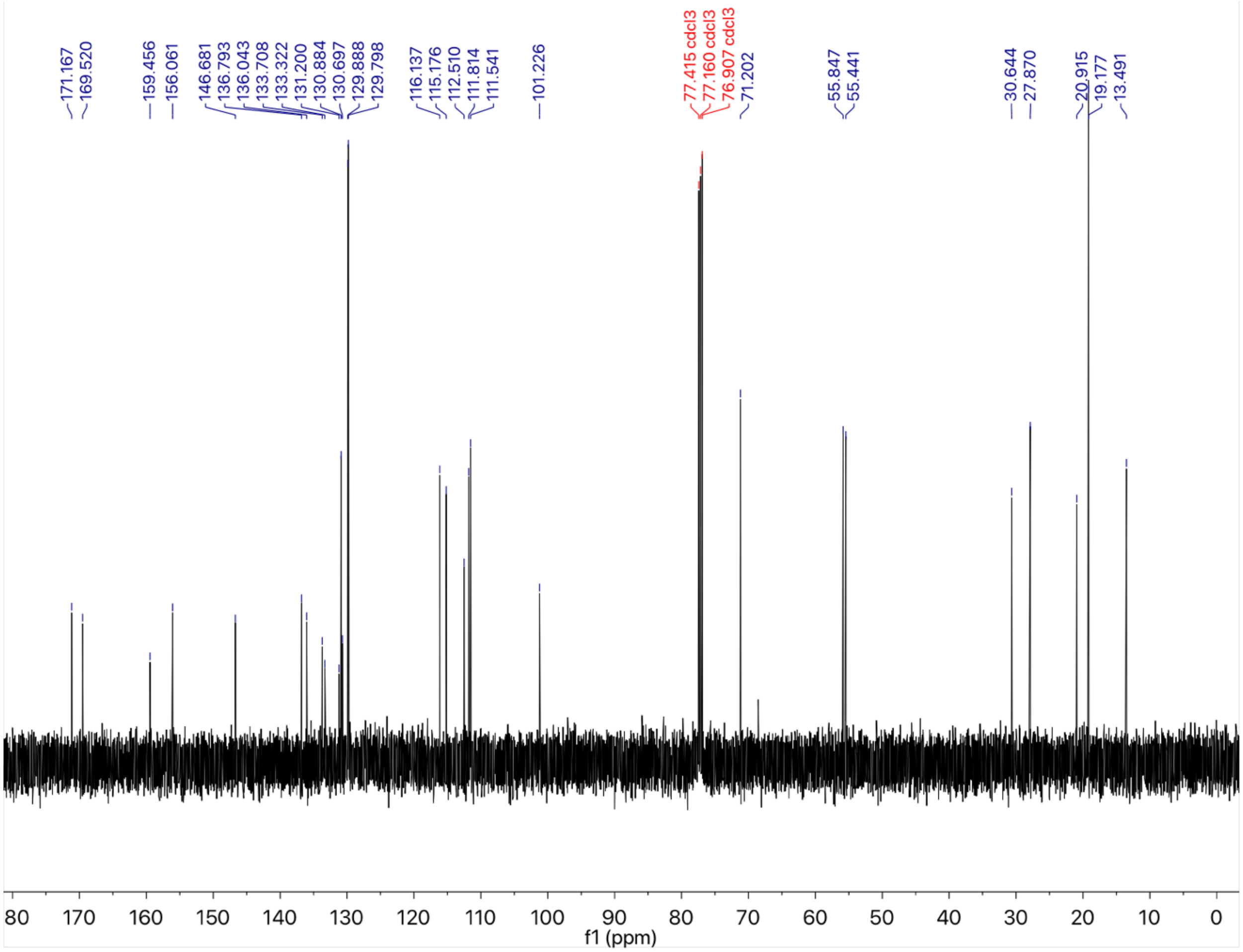

171.167
169.520
159.456
156.061
146.681
136.793
136.043
133.708
133.322
131.200
130.884
130.697
129.888
129.798
116.137
115.176
112.510
111.814
111.541
101.226
77.415 cdcl3
77.160 cdcl3
76.907 cdcl3
71.202
55.847
55.441
30.644
27.870
20.915
19.177
13.491
80 170 160 150 140 130 120 110 100 90 80 70 60 50 40 30 20 10 0
f1 (ppm)

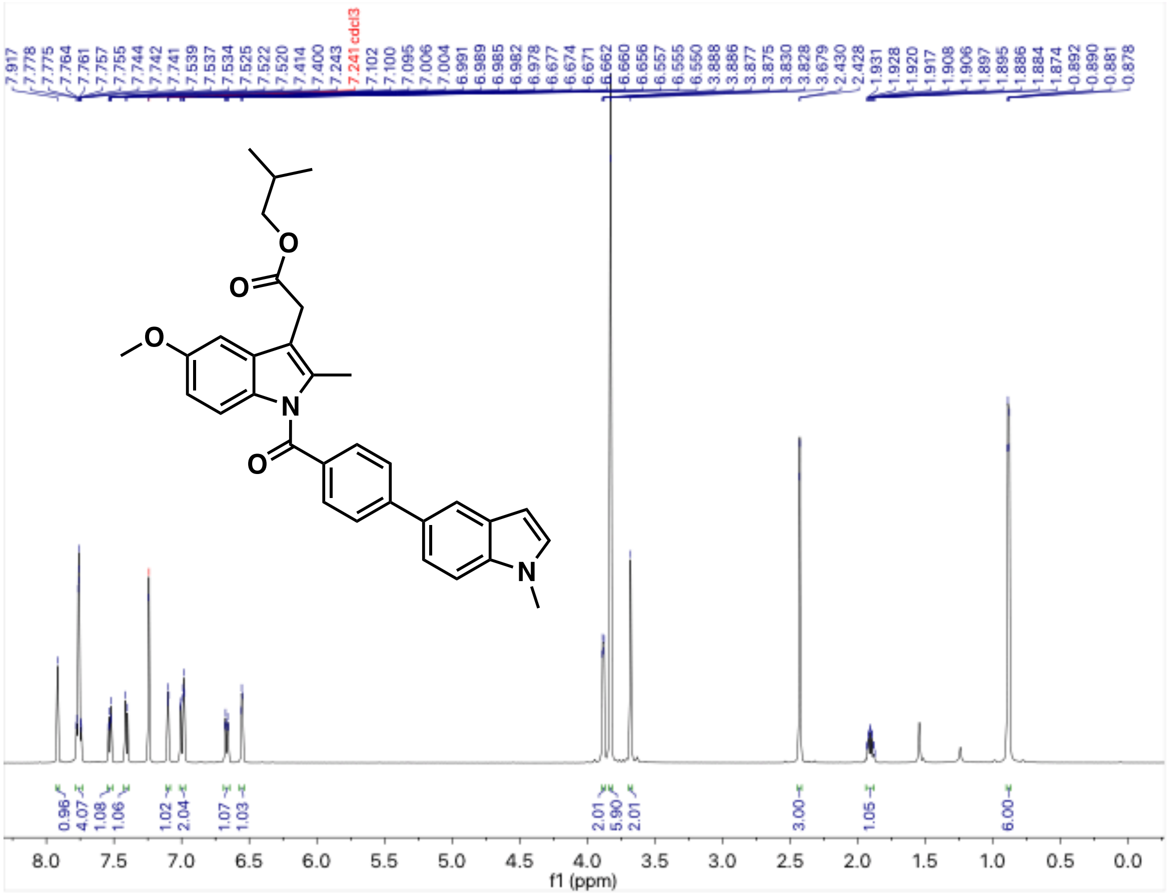

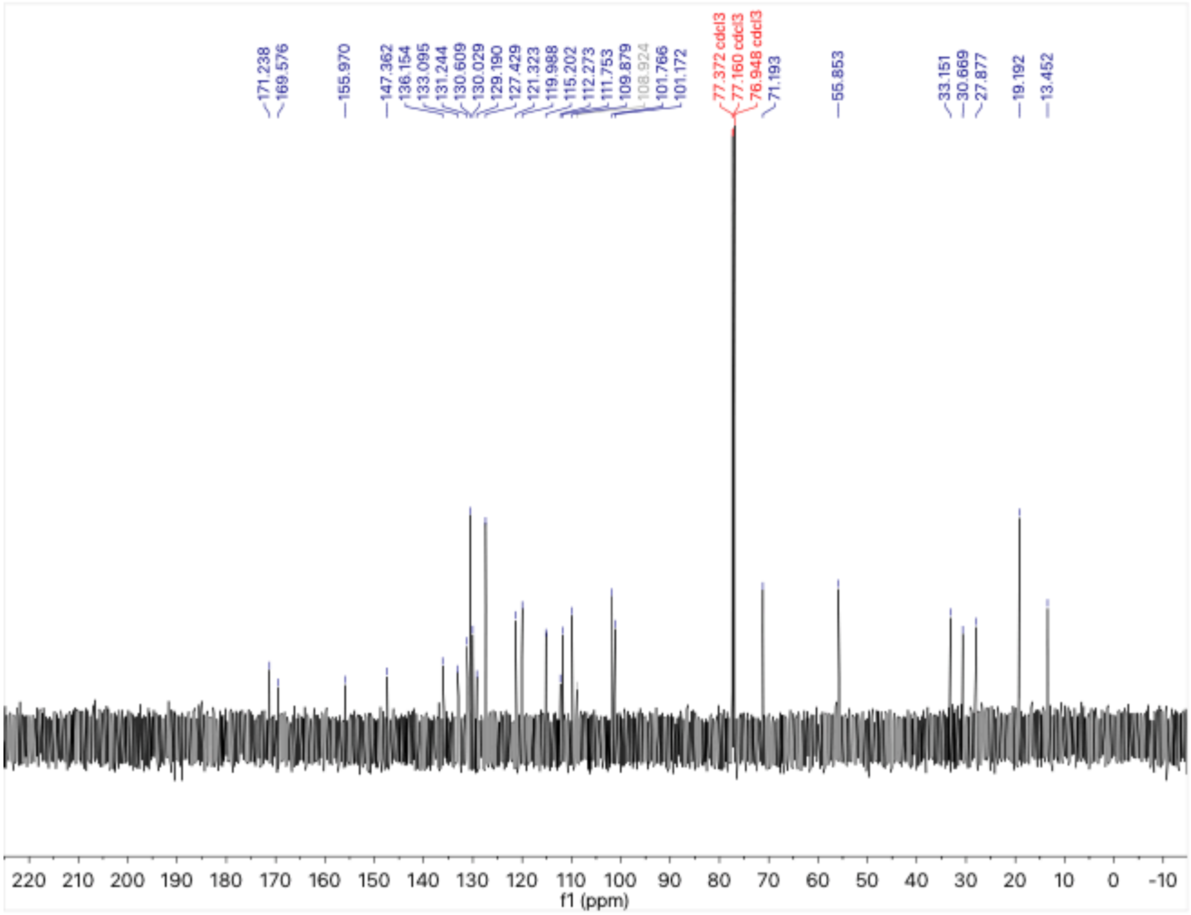

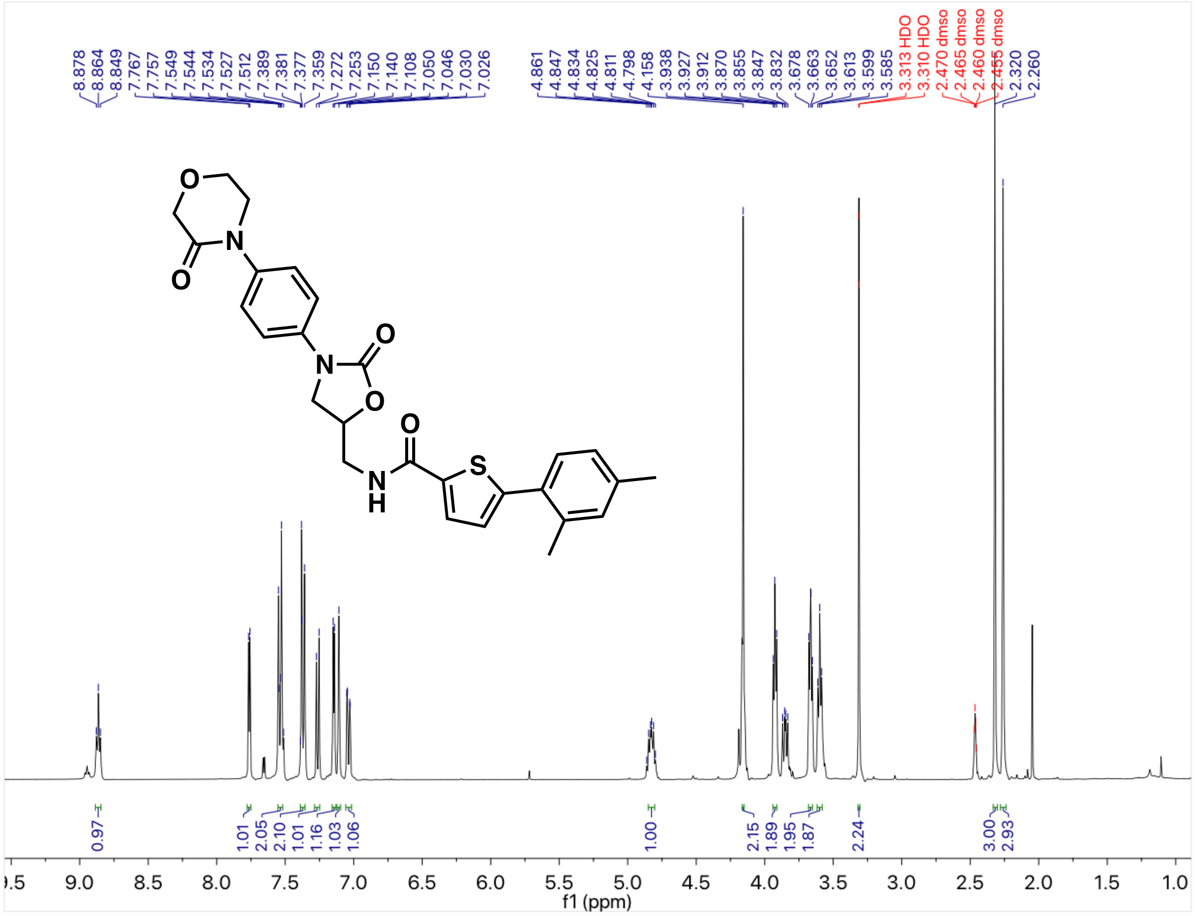
8.878
8.864
8.849
7.767
7.757
7.549
7.544
7.534
7.527
7.512
7.389
7.381
7.377
7.359
7.272
7.253
7.150
7.140
7.108
7.050
7.046
7.030
7.026
4.861
4.847
4.834
4.825
4.811
4.798
4.158
3.938
3.927
3.912
3.870
3.855
3.847
3.832
3.678
3.663
3.652
3.613
3.599
3.585
3.313 HDO
3.310 HDO
2.470 dmso
2.465 dmso
2.460 dmso
2.455 dmso
2.320
2.260
O
N
O
N
O
O
O
S
N
H
0.97
1.01
2.05
2.10
1.01
1.16
1.03
1.06
1.00
2.15
1.89
1.95
1.87
2.24
3.00
2.93
9.0 8.5 8.0 7.5 7.0 6.5 6.0 5.5 5.0 4.5 4.0 3.5 3.0 2.5 2.0 1.5 1.0
f1 (ppm)

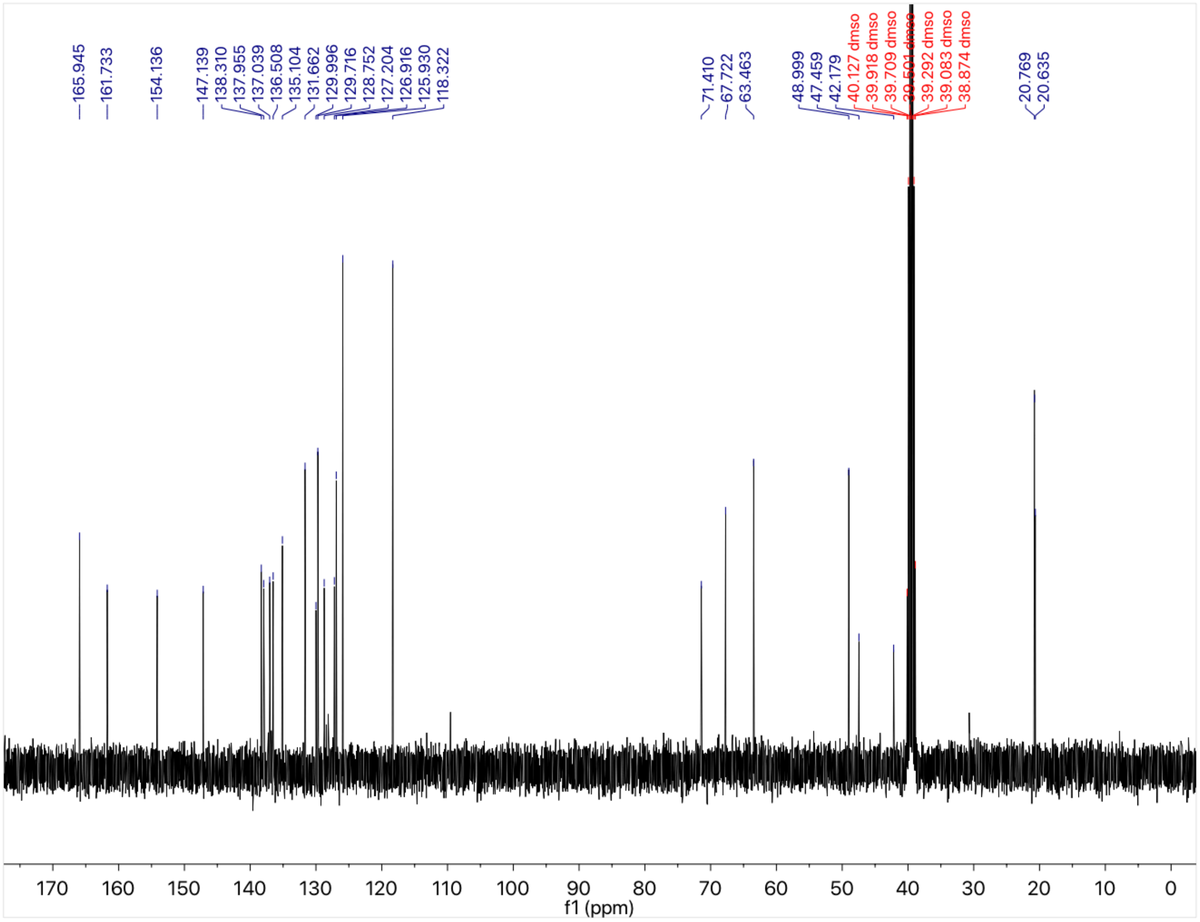
165.945
161.733
154.136
147.139
138.310
137.955
137.039
136.508
135.104
131.662
129.996
129.716
128.752
127.204
126.916
125.930
118.322
71.410
67.722
63.463
48.999
47.459
42.179
40.127 dmso
39.918 dmso
39.709 dmso
39.501 dmso
39.292 dmso
39.083 dmso
38.874 dmso
20.769
20.635
170 160 150 140 130 120 110 100 90 80 70 60 50 40 30 20 10 0
f1 (ppm)

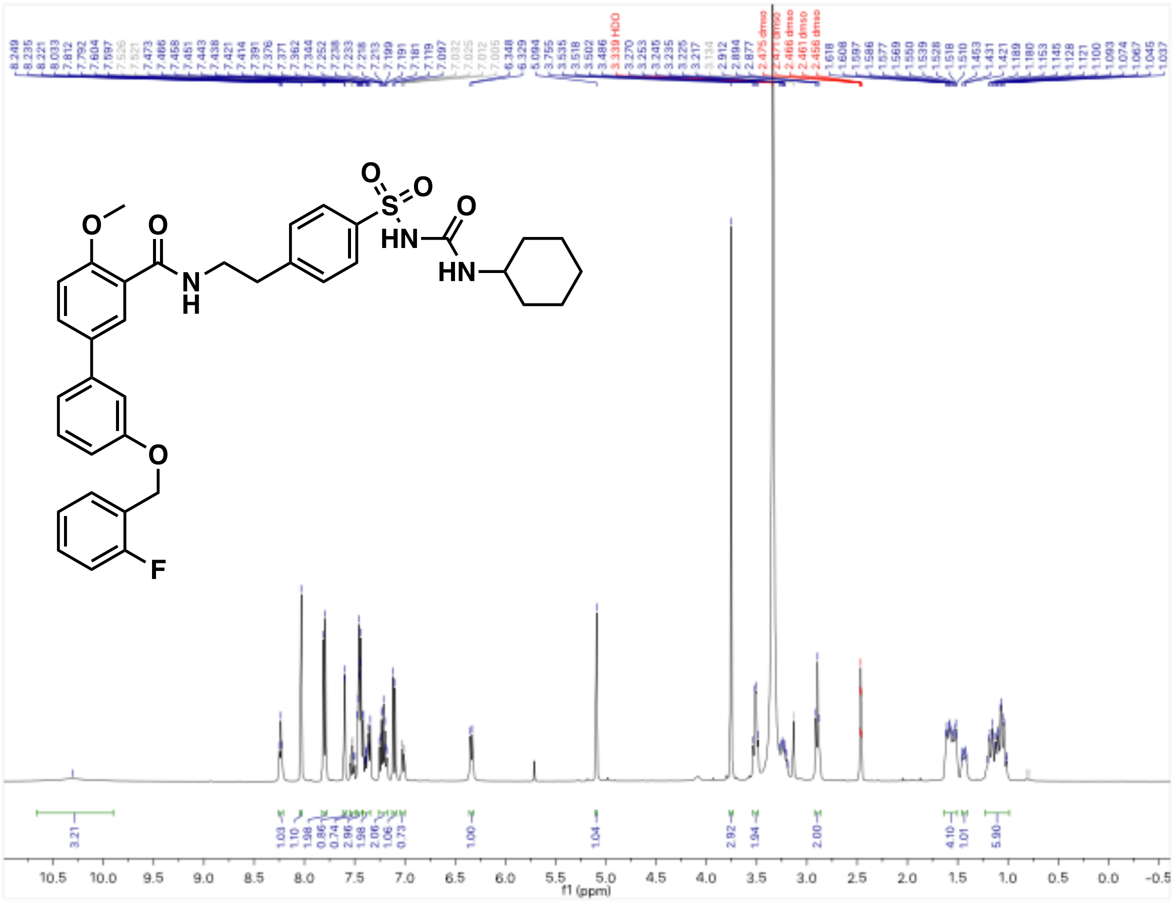

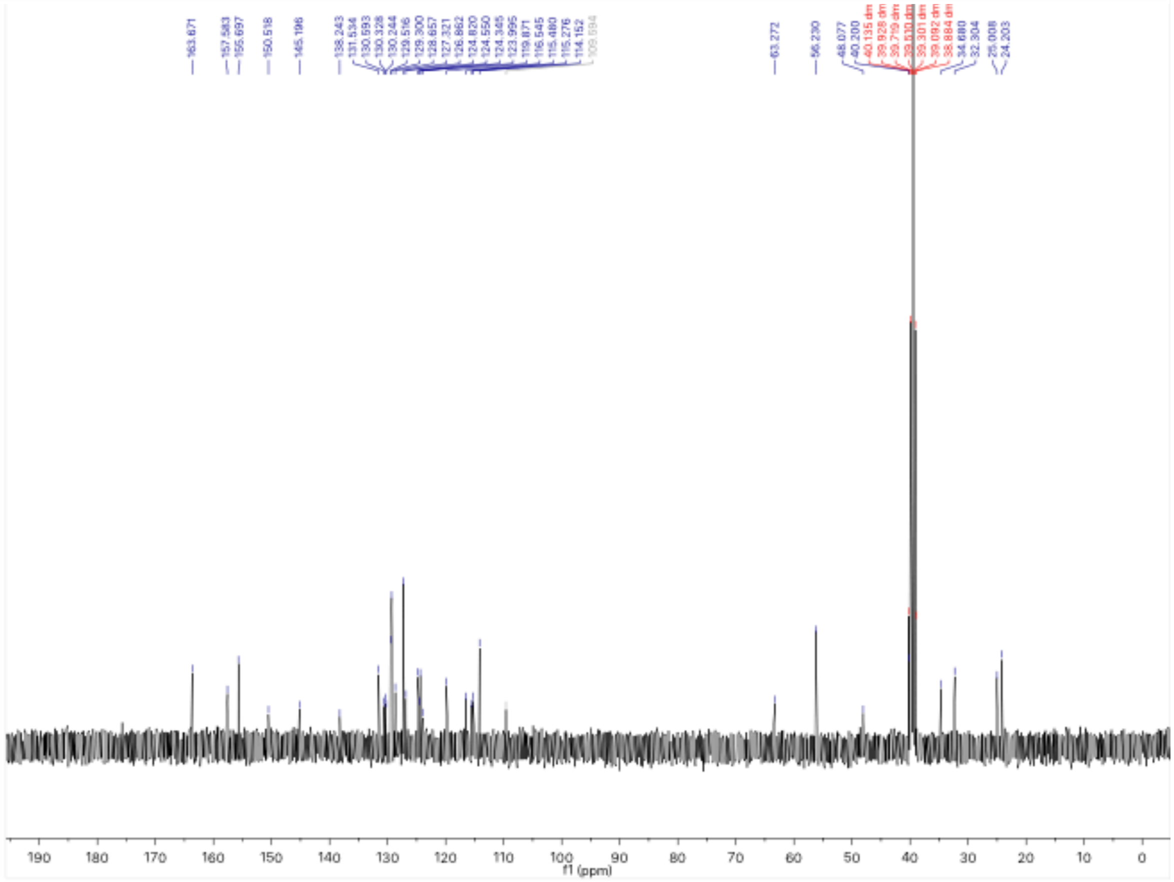

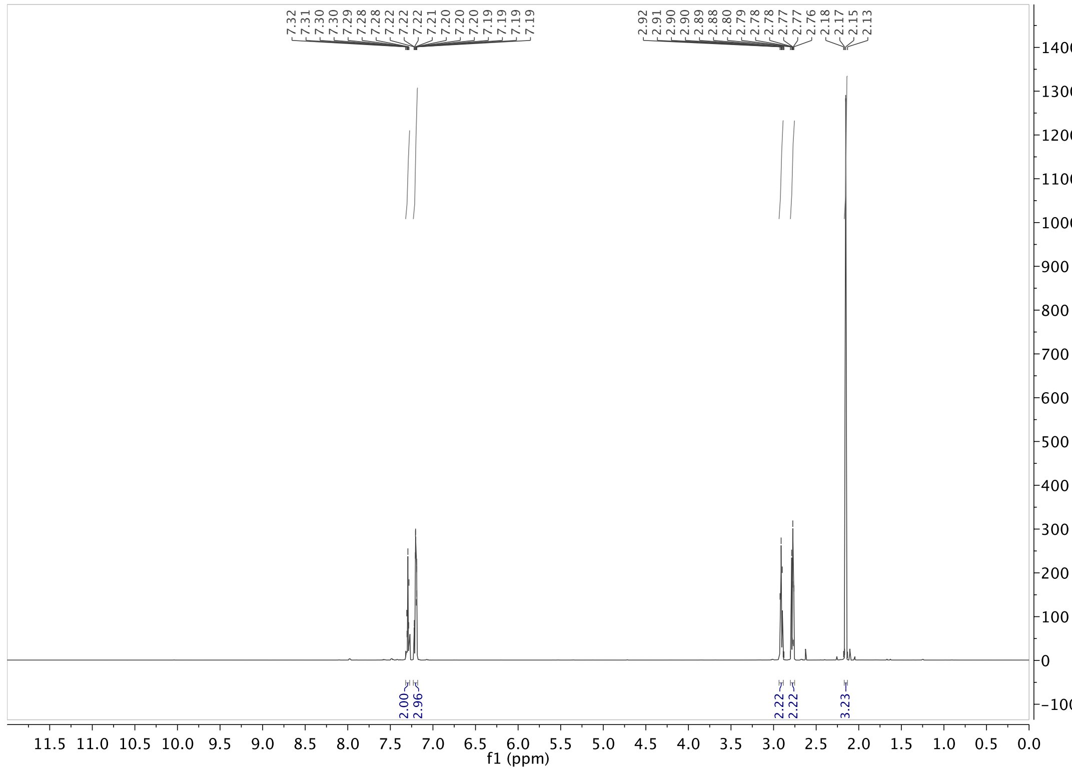

Fig. 1: ¹H NMR spectra of benzylacetone

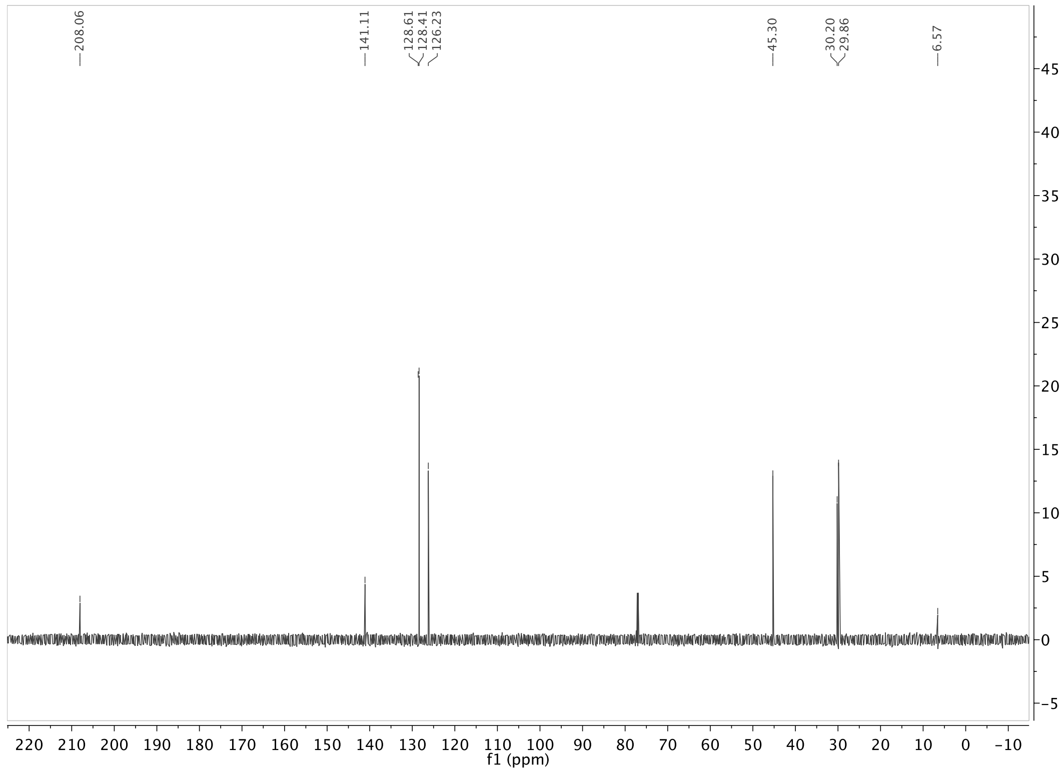

Fig. 2: ¹³C NMR spectra of benzylacetone

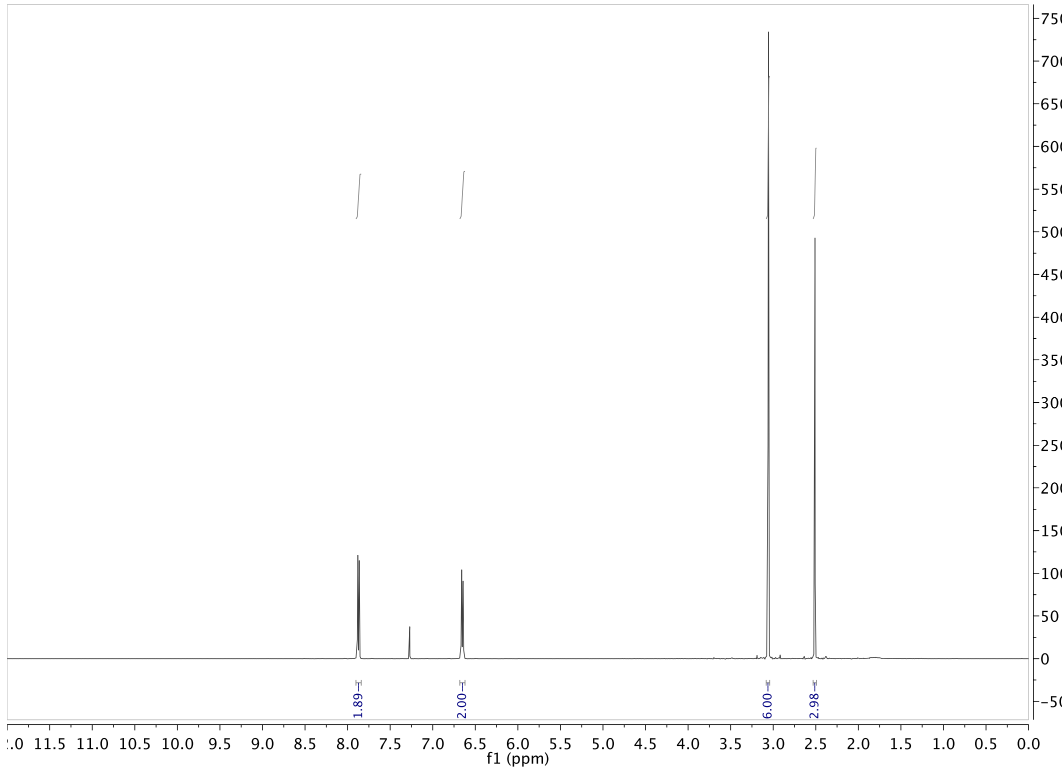

Fig. 3: ¹H NMR spectra of N-dimethylaminoacetophenone

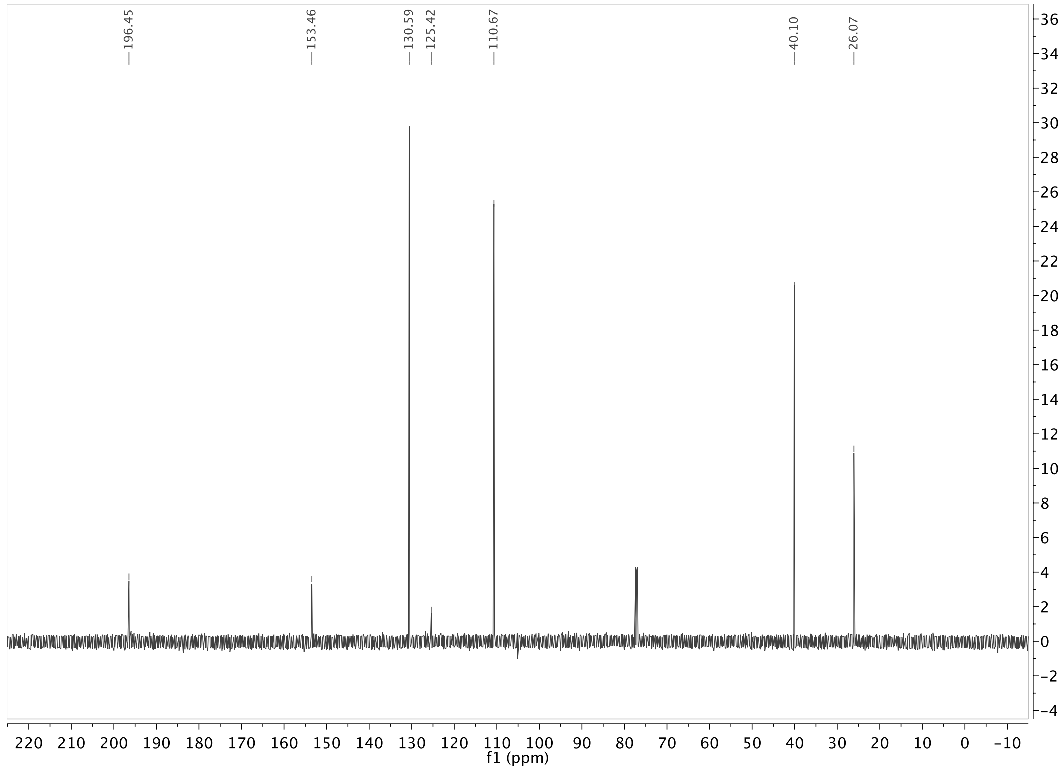

Fig. 4: ¹³C NMR spectra of N-dimethylaminoacetophenone

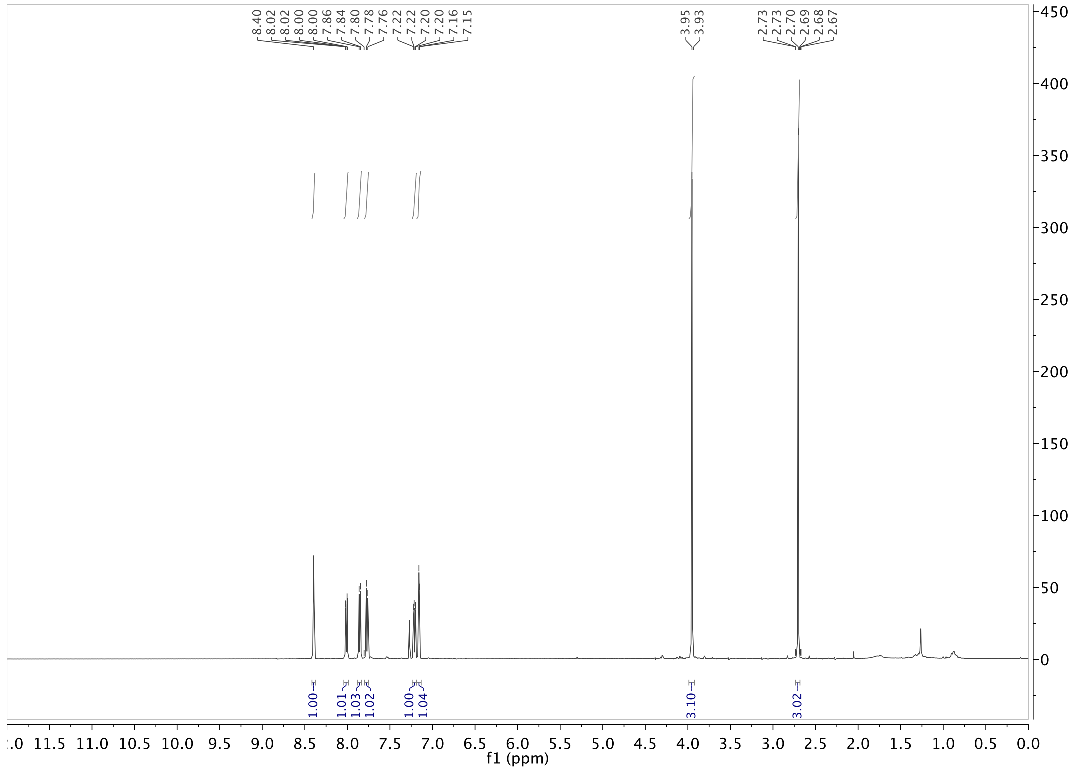

Fig. 5: 1H NMR spectra of 1-(6-methoxynaphthalen-2-yl)ethan-1-one

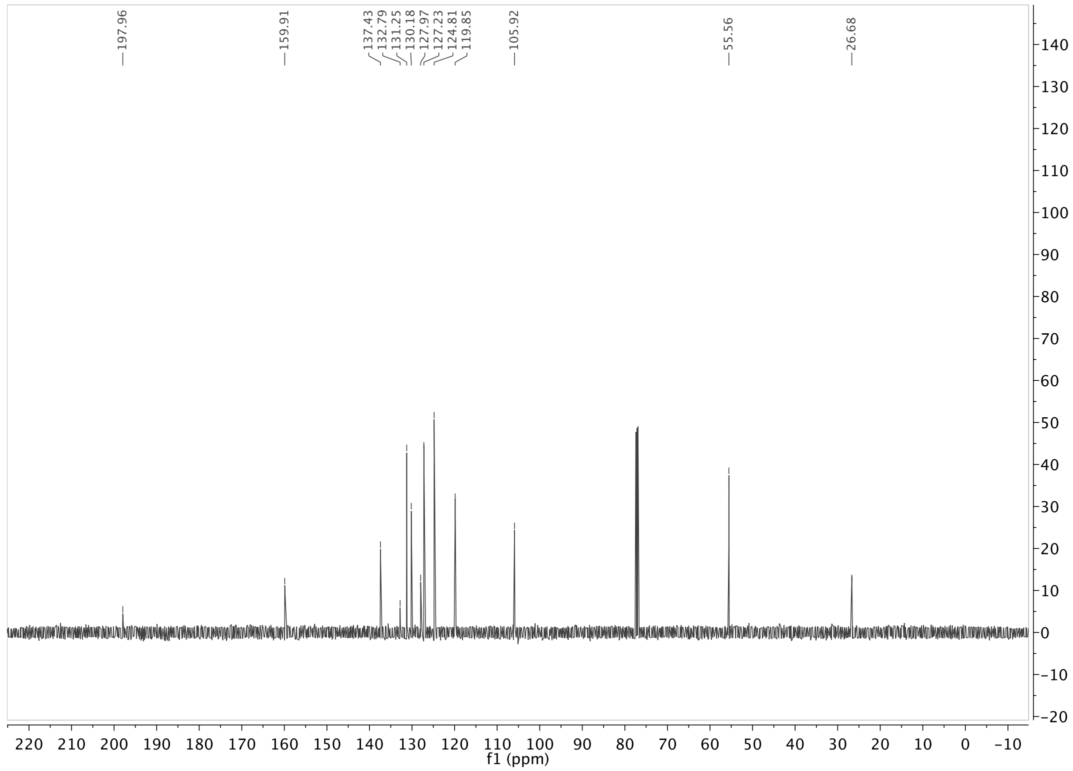

Fig. 6: ^{13}C NMR spectra of 1-(6-methoxynaphthalen-2-yl)ethan-1-one

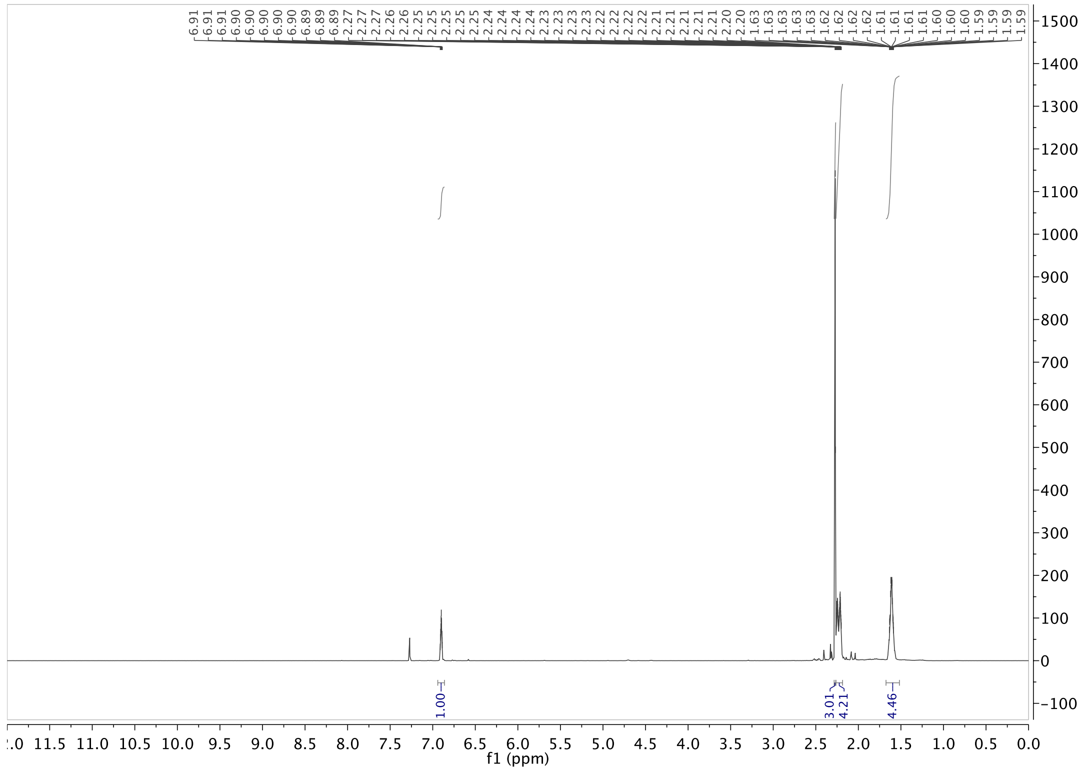

Fig. 7 :¹H NMR spectra of 1-acetyl-1-cyclohexene

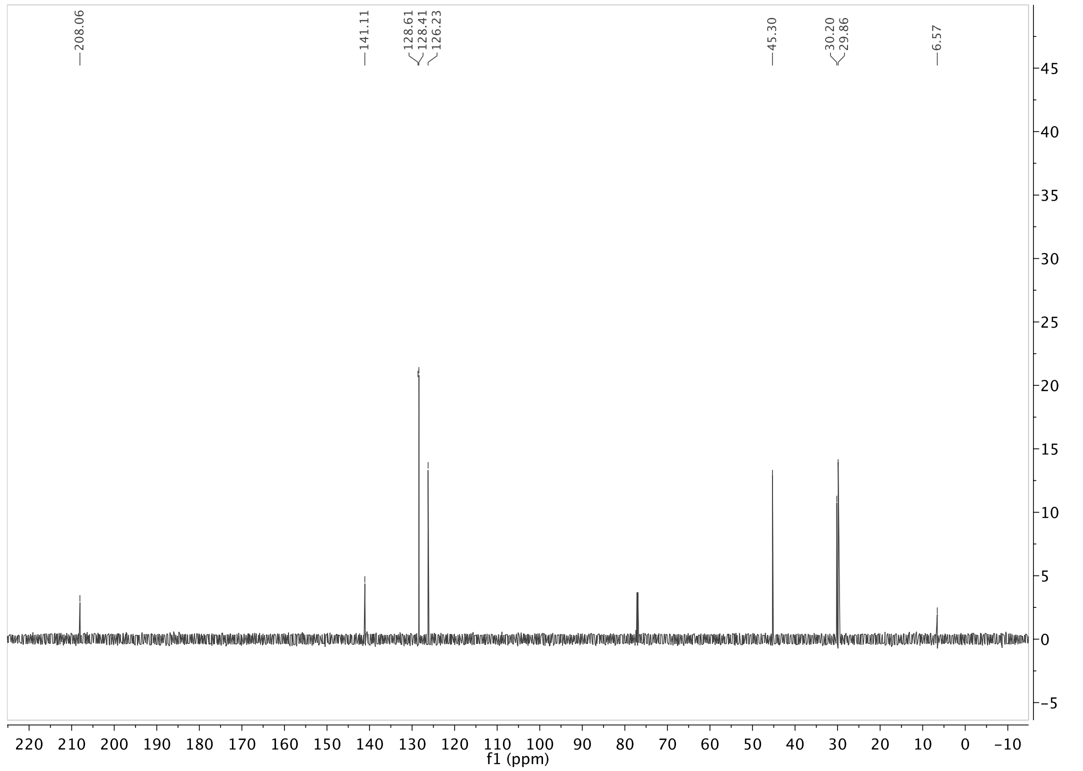

Fig. 8: ¹³C NMR spectra of 1-acetyl-1-cyclohexene

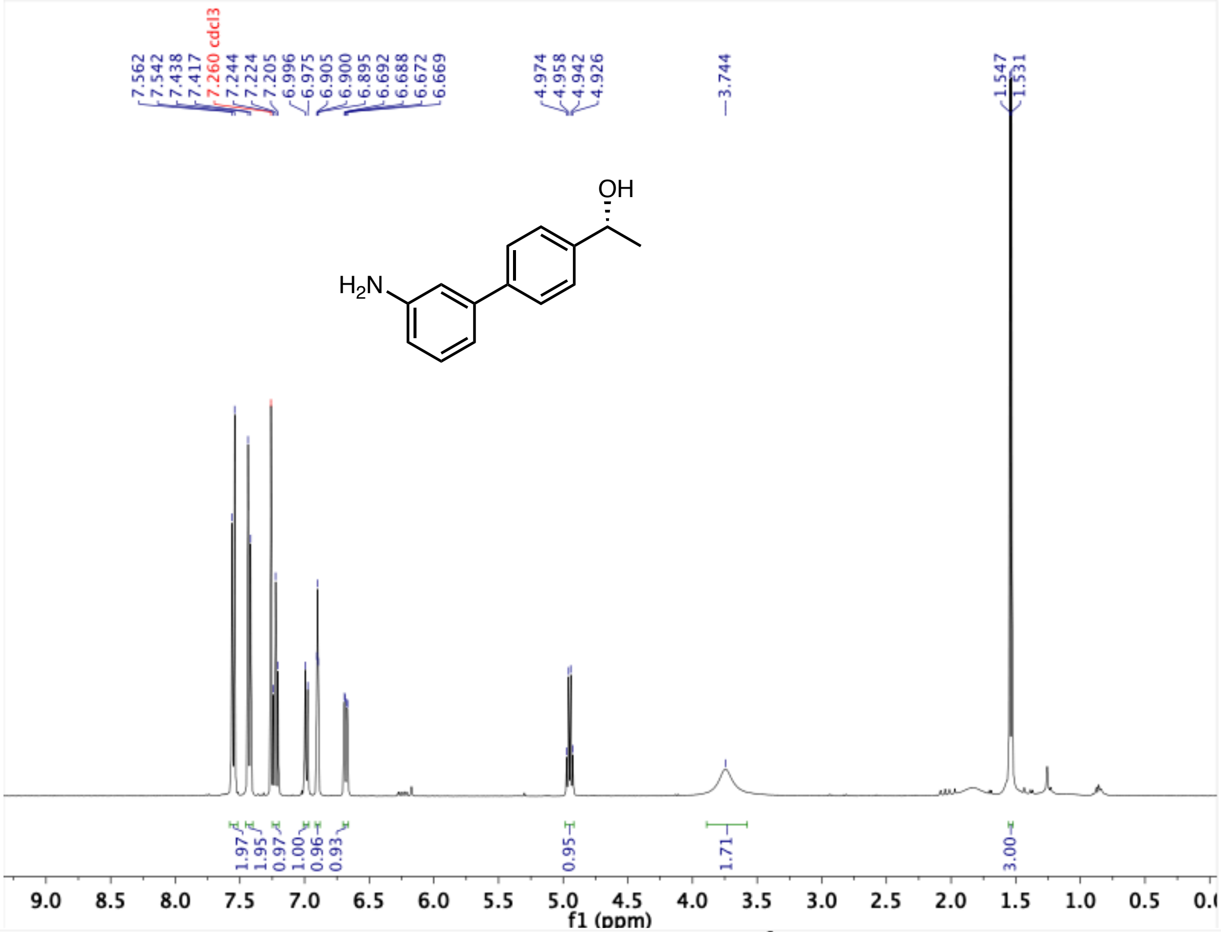
7.562
7.542
7.438
7.417
7.260 cdcl3
7.244
7.224
7.205
6.996
6.975
6.905
6.900
6.895
6.692
6.688
6.672
6.669
4.974
4.958
4.942
4.926
3.744
1.547
1.531
OH
H2N
1.97
1.95
0.97
1.00
0.96
0.93
0.95
1.71
3.00
9.0 8.5 8.0 7.5 7.0 6.5 6.0 5.5 5.0 4.5 4.0 3.5 3.0 2.5 2.0 1.5 1.0 0.5
f1 (ppm)

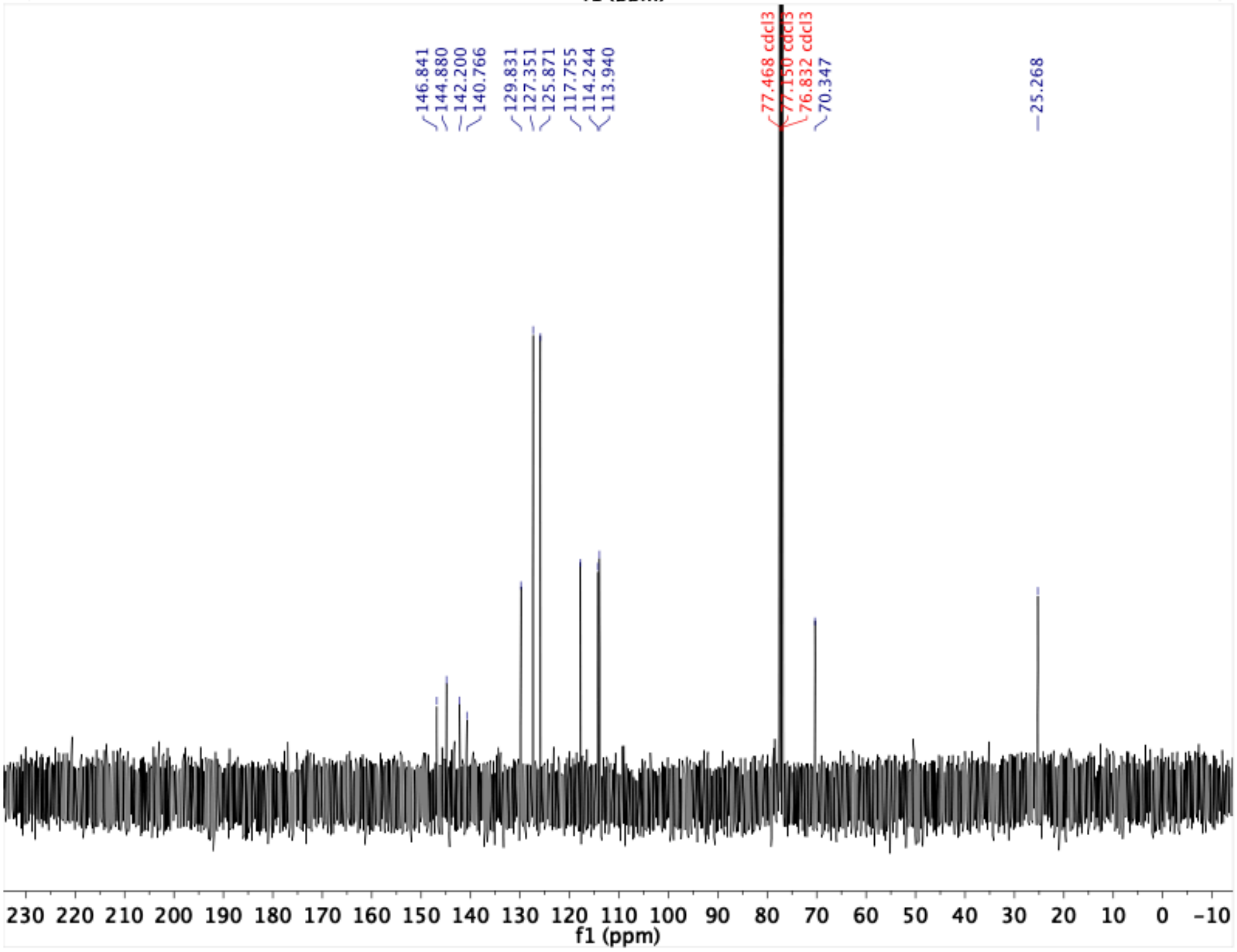
146.841
144.880
142.200
140.766
129.831
127.351
125.871
117.755
114.244
113.940
77.468 cdcl3
77.150 cdcl3
76.832 cdcl3
70.347
25.268
230 220 210 200 190 180 170 160 150 140 130 120 110 100 90 80 70 60 50 40 30 20 10 0 −10
f1 (ppm)
